# ^18^F‐Radiopharmaceutical Diversification Enabled by Deaminative Cross‐Electrophile Couplings

**DOI:** 10.1002/anie.202522650

**Published:** 2025-12-04

**Authors:** Isabella F. Ogilvy, Joseph Ford, Sebastiano Ortalli, Evelien Renders, Thomas R. Hayes, Shuanglong Liu, Inne Mortiers, Anastasia Nikolopoulou, Alexandre M. Sorlin, Andrés A. Trabanco, Matthew Tredwell, Peter J. J. A. Buijnsters, Rhys Salter, Véronique Gouverneur

**Affiliations:** ^1^ Department of Chemistry, University of Oxford Chemistry Research Laboratory 12 Mansfield Road Oxford OX1 3TA UK; ^2^ Global Discovery Chemistry Johnson & Johnson Turnhoutseweg 30 Beerse 2340 Belgium; ^3^ Global Discovery Chemistry Johnson & Johnson La Jolla California 92121 USA; ^4^ Global Discovery Chemistry Johnson & Johnson Spring House Pennsylvania 19477 USA; ^5^ Global Discovery Chemistry Johnson & Johnson Janssen‐Cilag S.A. Toledo E‐45007 Spain; ^6^ Wales Research and Diagnostic PET Imaging Centre Cardiff University University Hospital of Wales Heath Park Cardiff CF14 4XN UK; ^7^ School of Chemistry Cardiff University Main Building Park Place Cardiff CF10 3AT UK

**Keywords:** Cross‐coupling, Fluorine, High‐throughput screening, Radiochemistry, Synthetic methods

## Abstract

The development of ^18^F‐labelled radiotracers is of vital importance for (pre)clinical positron emission tomography (PET) imaging and to guide drug discovery campaigns. State‐of‐the‐art approaches often require labour‐intensive preparation of highly functionalised radiolabelling precursors. This bottleneck impedes analogue generation for optimal imaging and exploration of radiochemical space. To this end, we disclose a nickel‐mediated aryl (C)*sp* ^2^‐(C)*sp* ^3^ cross‐coupling with amine‐derived alkyl 2,4,6‐triphenylpyridinium salts as coupling partners amenable to radiosynthesis. The method was applied to primary and secondary 2,4,6‐triphenylpyridinium salts in radiochemical conversion (RCC) up to 86% and a high‐throughput experimentation (HTE) assay proved crucial for expedient ligand evaluation. A late‐stage diversification case study from a sole precursor achieved six ^18^F‐labelled GSK‐3 kinase inhibitor analogues, one being prepared in up to gigabecquerel (GBq) quantities in a (semi)automated two‐step protocol applied across three commercial radiosynthesis platforms.

## Graphical abstract

A general Ni‐mediated (C)*sp^2^*–(C)*sp^3^* cross‐coupling expedites access to ^18^F‐radiopharmaceuticals, essential for positron emission tomography imaging and drug discovery. This late‐stage diversification approach was implemented across three user‐friendly automated protocols affording ^18^F‐radiotracers in sufficient quantities for imaging. Tailored high‐throughput experimentation rapidly discovers radiochemically applicable reaction conditions.

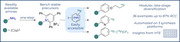

## Introduction

**Figure 1:**
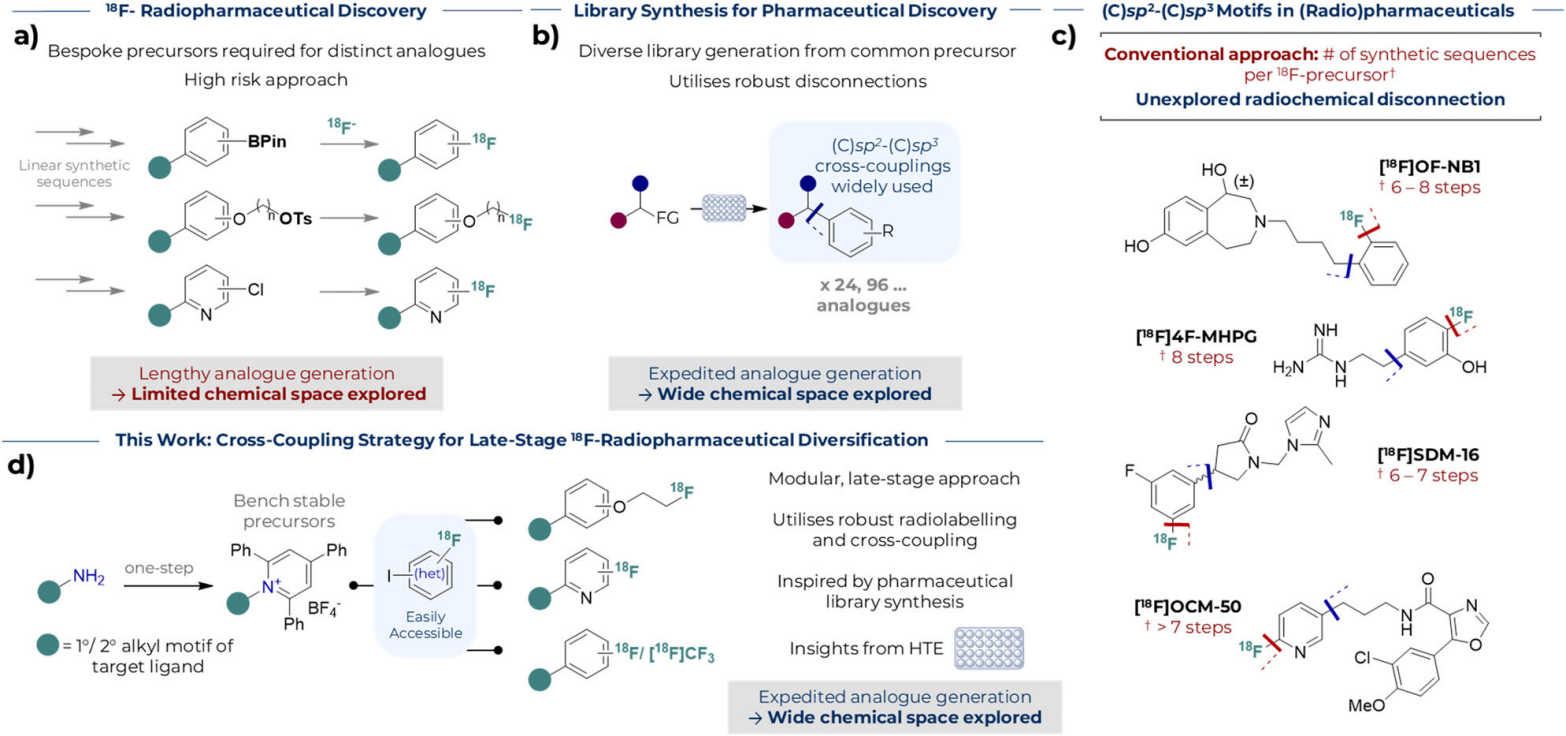
Overview of (radio)pharmaceutical discovery, (C)*sp^2^*–(C)*sp^3^* motifs in radiopharmaceuticals, and this work. a) State‐of‐the‐art approach to ^18^F‐labelled radiopharmaceutical discovery via linear synthetic routes to different analogues limits ^18^F‐chemical space. b) library approach for accessing analogues for pharmaceutical discovery highlighting prevalence of (C)*sp* ^2^–(C)*sp* ^3^ cross‐couplings; c) possible aryl (C)*sp* ^2^–(C)*sp* ^3^ radiosynthetic disconnections in existing ^18^F‐labelled radiopharmaceuticals along with the number of synthetic sequences per analogue precursor for direct radiofluorination; d) this work: a nickel‐mediated coupling of alkyl pyridinium salts and ^18^F‐fluoro(hetero)aryl iodides enables facile diversification to access ^18^F‐labelled radiotracers.

Positron Emission Tomography (PET) imaging relies on positron‐emitting radionuclides for non‐invasive in vivo interrogation of biological processes in real time.^[^ ^1^ ^]^ This technique is routinely used for clinical diagnosis and offers insights that inform drug discovery campaigns through studies such as receptor occupancy and biodistribution.^[^ ^2^ ^]^ Fluorine‐18 is a highly desirable isotope for such applications due to its advantageous decay properties (*t*_1/2 _= 109.8 min; 97% β^+^ decay; 635 keV).^[^ ^3^ ^]^ To access ^18^F‐labelled radiopharmaceuticals, practitioners often apply well‐established reactions including direct nucleophilic ^18^F‐fluorination of onium or nitro precursors for ^18^F‐fluoro(hetero)arenes, Cu‐mediated approaches from aryl boron/tin precursors, or S*_N_* 2 precursors for the synthesis of ^18^F‐fluoroalkyl compounds (Figure 1a).^[^ ^4^, ^5^, ^6^, ^7^ ^]^ While such established methods are robust, challenges arise when multiple structurally and electronically differentiated ^18^F‐labelled radiotracer analogues are required to identify the best candidate for PET studies. First, each structural analogue requires the synthesis of a distinct labelling precursor, often via lengthy and linear synthetic routes. Furthermore, highly functionalised and complex molecular structures present in modern (radio)pharmaceuticals can complicate established radiolabelling strategies and precursor synthesis.^[^ ^8^ ^]^ Therefore, generation of ^18^F‐labelled radiopharmaceutical analogues from a single precursor or method is typically not feasible, with state‐of‐the‐art workflows being high‐risk as well as time‐ and resource‐intensive. Such challenges lead to an overreliance on radiolabelled prosthetic groups or motifs that are readily accessed, which may result in significant structural deviations from lead structures optimised for target binding.^[^ ^9^ ^]^ Taken together, these factors represent a significant barrier to PET imaging with novel ^18^F‐labelled radiotracers.

This state‐of‐play contrasts with pharmaceutical discovery, whereby numerous structural analogues are routinely synthesised in a library format from a common precursor by employing robust disconnections and readily available building blocks (Figure 1b).^[^ ^10^, ^11^, ^12^ ^]^ Recent developments in aryl (C)*sp^2^*–(C)*sp^3^* cross‐couplings have been transformative to rapidly access diverse structures and accelerate the design‐make‐test‐analyse cycle of drug discovery.^[^ ^13^, ^14^ ^]^ Such couplings have not been disclosed in ^18^F‐radiochemistry beyond ^18^F‐(poly)fluoromethylation, despite their potential to accelerate access to PET radioligands for clinical diagnosis and drug discovery campaigns (Figure 1c).^[^ ^15^, ^16^, ^17^ ^]^ Herein, we provide a solution to expedite development of ^18^F‐labelled radiopharmaceuticals with a robust nickel‐mediated aryl (C)*sp* ^2^–(C)*sp* ^3^ cross‐coupling reaction between alkyl 2,4,6‐triphenylpyridinium salts and ^18^F‐labelled fluoro(hetero)aryl iodides (Figure 1d).

## Results and Discussion

### Reaction Development

Alkyl 2,4,6‐triphenylpyridinium salts derived from ubiquitous amines were identified as highly suitable coupling partners because they can be introduced via condensation or building block approaches and are amenable to a range of reductive processes (*e*.*g*., photochemical or stoichiometric reductants).^[^ ^18^, ^19^, ^20^, ^21^, ^22^, ^23^, ^24^, ^25^, ^26^ ^]^ Such charged substrates may also enable facile HPLC purification compared to neutral reagents, such as alkyl halides.^[^ ^27^ ^]^

**Scheme 1:**
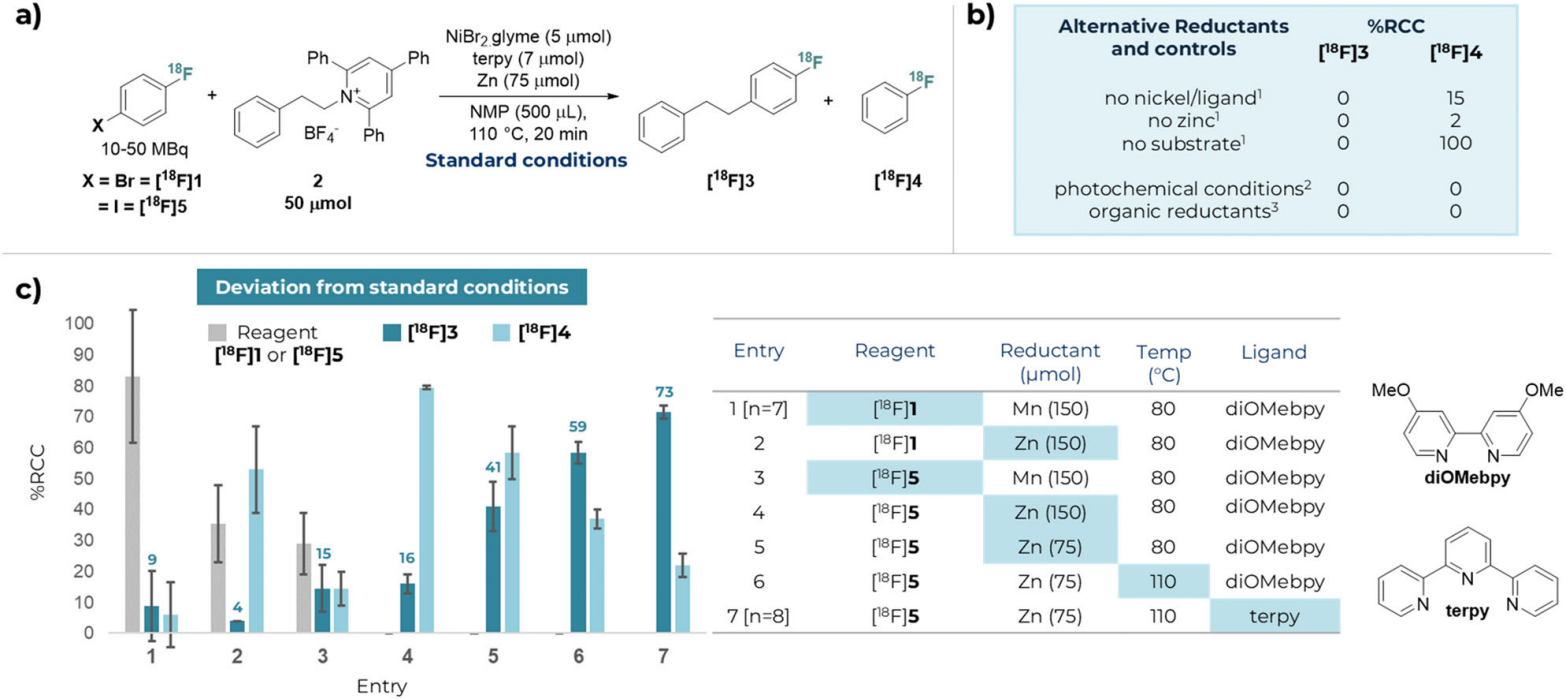
Optimisation and control reactions of aryl (C)*sp^2^*‐(C)*sp^3^* cross‐coupling of primary alkyl 2,4,6‐triphenylpyridinium tetrafluoroborate **2** with [^18^F]1‐halo‐4‐fluoroarenes [^18^F]**1** and [^18^F]**5**. ^a)^Standard Conditions. ^b)^Alternative reductants and controls. ^c)^Optimisation reactions with deviations from standard conditions (Scheme 1a). ^1^ Deviation from standard conditions (Scheme 1a). ^2^ See Supporting Information Section 1.7 for conditions screened ^3^ see Supporting Information Section 1.6.4 for organic reductants screened. % = %RCC = % radiochemical conversion as determined by RadioHPLC. *n* = 2 unless otherwise stated.

**Scheme 2:**
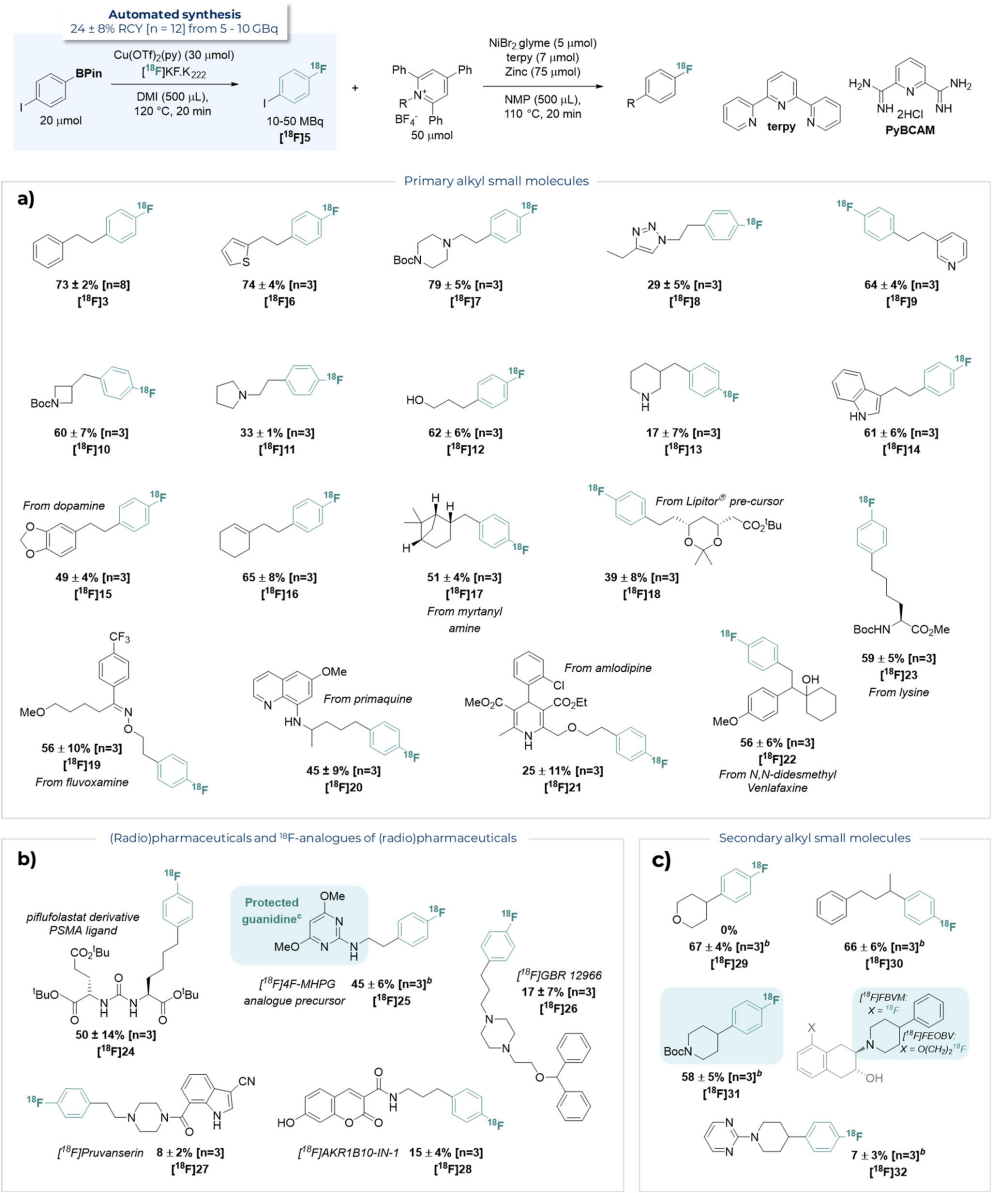
Alkyl coupling partner scope. ^a)^ % = %RCC = % radiochemical conversion as determined by RadioHPLC. ^b)^ Ligand used = PyBCAM (Pyridine‐2,6‐bis(carboximidamide) dihydrochloride). ^c )^2,4,6‐triphenylpyridinium precursor accessed in two steps from commercial starting material.

We began our studies by investigating nickel‐catalysed (C)*sp^2^*–(C)*sp^3^* cross‐electrophile couplings with [^18^F]1‐bromo‐4‐fluorobenzene ([^18^F]**1**), readily prepared by Cu‐mediated radiofluorination of the corresponding boron pinacol ester and purified by semi‐preparative HPLC for small‐scale screening reactions (Scheme 2).^[^ ^28^ ^]^ Applying the metallophotoredox conditions, disclosed by Yi et. al., with alkyl 2,4,6‐triphenylpyridinium salt **2** no cross‐coupling product ([^18^F]**3**) was observed.^[^ ^22^ ^]^ Alternative photochemical conditions using electron donor–acceptor mediated reduction were equally unsuccessful (Scheme 1b), highlighting the challenges associated with translating established chemical reactivity to ^18^F‐radiochemistry.^[^ ^23^ ^]^ We next investigated stoichiometric reductants. Seminal reports from the groups of Watson, Martin, and Rueping demonstrated manganese is an efficient reductant for nickel‐catalysed cross‐coupling of aryl bromides and alkyl pyridinium salts.^[^ ^18^, ^19^, ^20^ ^]^ Direct application of analogous radiochemical conditions afforded [^18^F]**3** in an average RCC of 9% (Scheme 1c, entry 1), along with unreacted [^18^F]**1**, and undesired reduction product [^18^F]fluorobenzene ([^18^F]**4**). However, this result was highly irreproducible, with RCCs in the range of 0–27% (9% ± 11, *n *= 7, Scheme 1c, entry 1, see Supporting Information **Section 1.6.3**). Further optimisation of this system using manganese delivered no improvement, prompting consideration of alternative reductants. Established organic reductants, such as tetrakis(dimethylamino)ethylene (TDAE), were found to be ineffective under radiochemical cross‐coupling conditions, despite reliable non‐radiochemical reactivity (Scheme 1b).^[^ ^29^, ^30^ ^]^ When zinc was used as a reductant, as demonstrated by Ni et. al. and others, desired product [^18^F]**3** was formed in 4% RCC along with significant amounts of [^18^F]**4** (53% RCC) (Scheme 1c, entry 2).^[^ ^29^, ^31^, ^32^ ^]^ Having performed extensive investigations with [^18^F]**1**, the nature of the ^18^F‐labelled aryl halide was evaluated next. [^18^F]1‐Iodo‐4‐fluorobenzene ([^18^F]**5**) was reactive with both Mn and Zn reductants, but Mn was dismissed further due to issues with reproducibility (Scheme 1c, entries 3–4). With Zn as the reductant, [^18^F]**5** was consumed quantitatively; however, [^18^F]**3** was formed in 16% RCC along with [^18^F]**4** in 80% RCC (Scheme 1c, entry 4). These data highlight the challenges in the development of radiochemical transformations; reliable non‐radioactive reactivity can result in no or suboptimal reactivity when analogous radiochemical conditions are trialled.

Thorough optimisation was required to attenuate the formation of undesired reduction product [^18^F]**4**. Reduction of Zn loading from 150 to 75 µmol delivered [^18^F]**3** in 41% RCC (Scheme 1c, entry 5). Increasing the reaction temperature from 80 to 110 °C (Scheme 1c, entry 6) and changing the ligand from 4–4′‐dimethoxy‐2–2′‐bipyridine (diOMebpy) to 2,2′:6′,2′′‐terpyridine (terpy) were further beneficial and led to the formation of [^18^F]**3** in 73 ± 2% RCC [*n *= 8] (Scheme 1c, entry 7).

Control reactions were conducted to determine the source of formation of [^18^F]**4**. In the presence of either Zn or NiBr_2_.glyme/terpy with model substrate **2**, no formation of product [^18^F]**3** and minimal formation of [^18^F]**4** was observed (15% and 2% RCC, respectively). In contrast, complete conversion of [^18^F]**5** to [^18^F]**4** (100% RCC) were observed in the presence of both Zn and NiBr_2_.glyme/terpy, in the absence of the pyridinium salt **2**. This observation is supported by previous studies in nickel catalysis, which suggest this reduction byproduct arises from in situ formation of an aryl zinc species from the aryl halide, that then undergoes subsequent protodezincation.^[^ ^32^, ^33^ ^]^ This is in line with predominant formation of [^18^F]**4** observed in our system when zinc is used (Scheme 1c, entry 4). For completeness, alkyl halide and tosylate precursors were evaluated as alternative alkyl coupling partners under our optimal conditions, but alkyl 2,4,6‐triphenylpyridinium salt **2** demonstrated superior reactivity and reproducibility (see Supporting Information **Section 1.7**).^[^ ^27^ ^]^

### Substrate Scope

With optimal conditions in hand, a broad range of primary alkyl 2,4,6‐triphenylpyridinium tetrafluoroborate salts were found to be competent coupling partners (Scheme 2). Medicinally relevant heterocycles, such as thiophene ([^18^F]**6**), piperazine ([^18^F]**7**), triazole ([^18^F]**8**), pyridine ([^18^F]**9**), azetidine ([^18^F]**10**), and pyrrolidine ([^18^F]**11**), were tolerated in up to 79% RCC. Unprotected nucleophilic functionalities such as alcohols ([^18^F]**12**), phenols ([^18^F]**28**), secondary amines ([^18^F]**13**), and indoles ([^18^F]**14**), were also compatible with our reaction. An electron‐rich dopamine derivative was effective, forming [^18^F]**15** in 49% RCC. However, a substrate featuring a Ni/Zn‐sensitive, electron‐poor nitro group was not tolerated (see Supporting Information **Section 1.8** for further unsuccessful substrates). Despite the radical nature of this process, an alkene‐containing substrate was successfully converted to the corresponding radiolabelled product [^18^F]**16** in 65% RCC. Structurally complex alkyl coupling partners derived from myrtanyl amine ([^18^F]**17**) and a Lipitor precursor ([^18^F]**18**) were obtained from cross‐coupling with [^18^F]**5**. Several complex drugs such as fluvoxamine ([^18^F]**19**), primaquine ([^18^F]**20**), amlodipine ([^18^F]**21**) and *N,N*‐didesmethyl venlafaxine ([^18^F]**22**) were amenable to derivatisation with [^18^F]**5** in 25–56% RCC. Additionally, lysine residues were successfully derivatised utilising this technology, as demonstrated by [^18^F]**23** and an analogue of PSMA radioligand piflufolastat ([^18^F]**24**). We also applied this methodology to further examples of known (radio)pharmaceuticals (Scheme 2b). A protected analogue of the cardiac sympathetic nerve radiotracer [^18^F]4F‐MHPG ([^18^F]**25**) was formed in 45% RCC.^[^ ^34^ ^]^ The dopamine transport inhibitor [^18^F]GBR 12 996 ([^18^F]**26**) and an isotopologue of 5‐HT_2A_ receptor antagonist pruvanserin ([^18^F]**27**) were accessed in 17% and 8% RCC, respectively.^[^ ^35^ ^]^ Additionally, [^18^F]AKR1B10‐IN‐1 ([^18^F]**28**), an inhibitor of Aldo–Keto Reductase 1B10, was prepared in 15% RCC. Notably, the developed Ni‐mediated cross‐coupling tolerates pharmaceutically relevant motifs that typically inhibit Cu‐mediated deborylative fluorination reactions including pyridine ([^18^F]**9**), pyrimidine ([^18^F]**25** and [^18^F]**32**), pyrrolidine ([^18^F]**11**), unprotected secondary aniline ([^18^F]**20**), and unprotected piperidine ([^18^F]**13**). This demonstrates the value of this disconnection to build in complex and (radio)pharmaceutically relevant motifs as a post‐labelling step.^[^ ^8^ ^]^

**Scheme 3:**
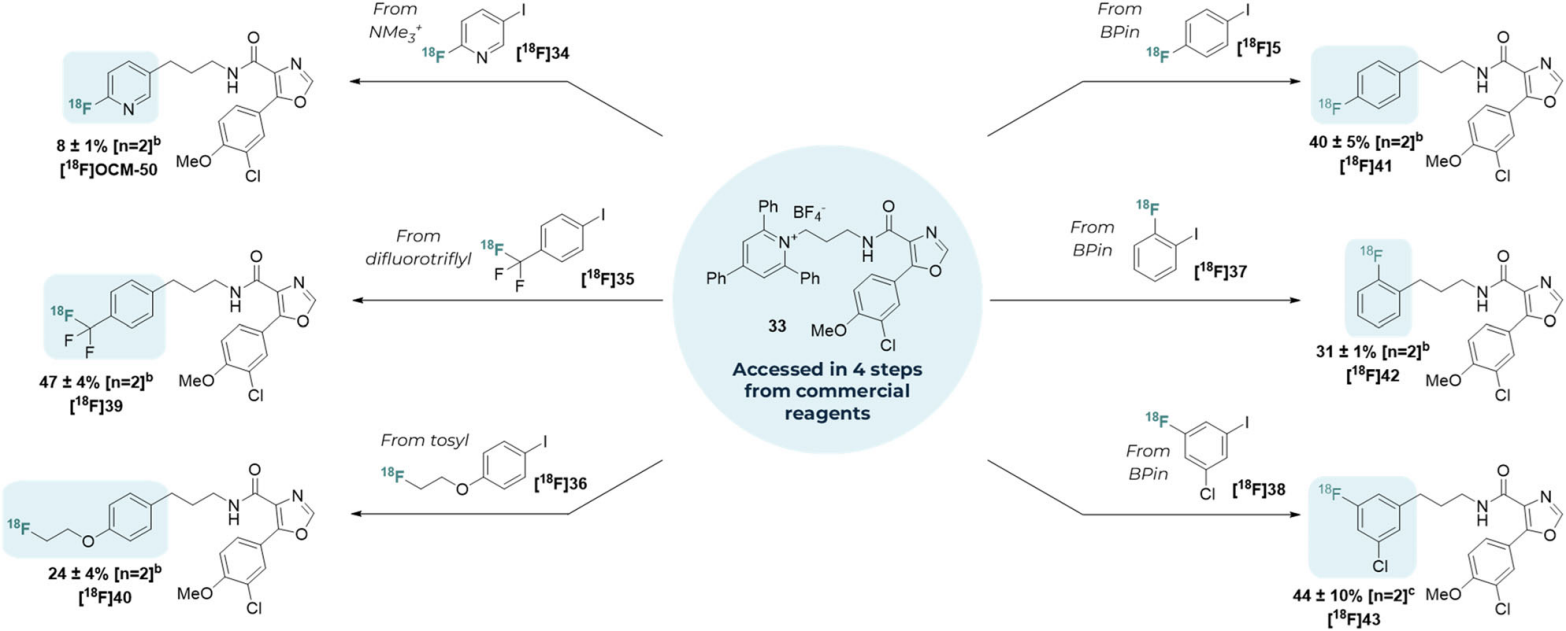
Late‐stage diversification approach for accessing ^18^F‐GSK‐3 kinase inhibitor analogues. ^a)^ % = %RCC = % radiochemical conversion as determined by RadioHPLC. ^b)^ and ^c)^ Standard conditions (Scheme 1a) used with ligand specified as follows: ^(b)^ Ligand used = terpyridine; ^(c)^ ligand used = PyBCAM (Pyridine‐2,6‐bis(carboximidamide) dihydrochloride).

**Scheme 4:**
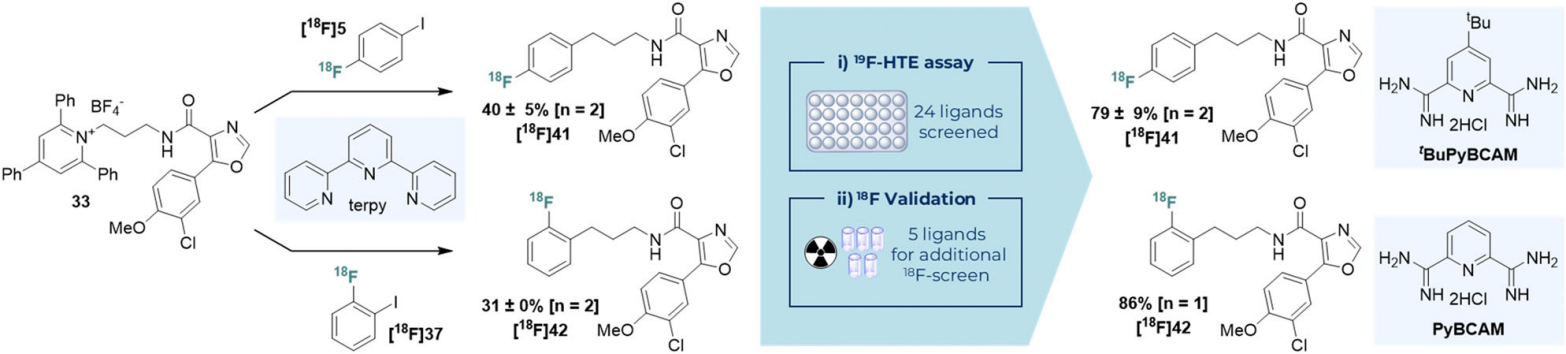
^19^F‐HTE ligand assay validation and improved ^18^F‐cross‐coupling conditions. ^a)^% = %RCC = % radiochemical conversion as determined by RadioHPLC. Conditions as described in Scheme 1a with ligand varied as specified.

Having demonstrated feasibility for primary alkyl pyridinium tetrafluoroborate substrates, we turned our attention to substrates derived from secondary amines (Scheme 2c). Initial attempts applying our optimised conditions to the alkyl pyridinium salt precursor of [^18^F]**29**, derived from tetrahydro‐*2H*‐pyran‐4‐amine, resulted in no desired product. While several groups have reported the cross‐coupling of more challenging secondary alkyl 2,4,6‐triphenylpyridinium salts under non‐radiochemical systems, adaptations to reaction conditions such as decreased reaction temperature or slow addition of substrate were found to be unsuitable for radiochemical cross‐coupling.^[^ ^18^, ^19^, ^32^ ^]^ Further screening led to the identification of amidine‐based ligands, such as PyBCAM, as highly privileged for secondary alkyl substrates, forming [^18^F]**29** in 67% RCC (see Supporting Information **Section 1.6.7**). Several secondary alkyl substrates could now successfully undergo cross‐coupling with [^18^F]**5** in 7–67% RCC, including a branched aliphatic secondary substrate leading to [^18^F]**30**, and two piperidine‐derived substrates, producing [^18^F]**31** − a core motif of vesicular acetylcholine transporter (VAChT) radiotracers [^18^F]FEOBV and [^18^F]FBVM − and [^18^F]**32**.

### Late‐Stage Diversification Case Study

With reaction conditions applicable to both primary and secondary substrates in hand, we turned our attention to varying the ^18^F‐labelled iodoarene partner. As a case study, we focused on the generation of radiolabelled analogues of PF‐367, a glycogen synthase kinase 3 (GSK‐3) inhibitor.^[^ ^36^ ^]^ GSK‐3 deregulation has been implicated in neurological diseases, such as Alzheimer's disease (AD), and therefore GSK‐3 inhibitors are sought after ^18^F‐labelled radiotracers for the clinical diagnosis of AD.^[^ ^37^, ^38^, ^39^ ^]^ A selective GSK‐3β inhibitor was therefore selected for late‐stage radiochemical diversification. This compound has been the subject of extensive structure‐activity relationship (SAR) studies in order to identify suitable ^11^C/^18^F‐labelled radiotracer analogues that can penetrate the blood–brain barrier.^[^ ^40^ ^]^

Common 2,4,6‐triphenylpyridinium intermediate **33** was readily accessed in four steps in 32% overall yield (up to 10 mmol). Following this, six representative ^18^F‐radiolabelled (hetero)aryl halides containing (radio)pharmaceutically ubiquitous fluorinated motifs ([^18^F]**5**, [^18^F]**34**, [^18^F]**35**, [^18^F]**36**, [^18^F]**37** and [^18^F]**38**) was explored as coupling partners. These labelled reagents were conveniently prepared from a range of precursors applying known radiochemistry (see Supporting Information **Section 1.10**) and subsequently reacted with **33** applying our optimised reaction conditions. The protocol rapidly yielded six ^18^F‐GSK 3 inhibitor analogues ([^18^F]OCM‐50, [^18^F]**39**, [^18^F]**40**, [^18^F]**41**, [^18^F]**42** and [^18^F]**43**) in moderate to good conversion (8–47% RCC) from a single precursor, including those featuring [^18^F]2‐fluoropyridyl ([^18^F]OCM‐50), ^18^F‐trifluoromethyl ([^18^F]**39**) and ^18^F‐fluoroethoxy motifs ([^18^F]**40**) (Scheme 3).^[^ ^41^, ^42^ ^]^ Since the RCCs obtained using our model conditions did not exceed 47%, further investigation ensued. Varying the reaction temperature or Zn loading with these different ^18^F‐labelled fluoro(hetero)aryl halide coupling partners did not lead to further improvement in RCC (see Supporting Information **1.10.4**). We noted that one single ligand—terpy or PyBCAM—was not optimal across a panel of ^18^F‐labelled fluoro(hetero)aryl iodides ([^18^F]**5**, [^18^F]**35**, [^18^F]**36**) in reactions with model substrate **2** (see Supporting Information **Section 1.10.3**). These data highlighted the importance of the ligand for efficient cross‐coupling with alternative ^18^F‐labelled fluoro(hetero)aryl iodides and prompted investigation of further applicable ligands.

### HTE Ligand Selection

To expedite the discovery of efficient ligands, a non‐radioactive high throughput experimentation (HTE) assay was developed, specifically tailored to radiochemical reactions by accounting for stoichiometry differences, and subsequently validated with radiochemical control studies. Twenty‐four possible ligands were evaluated for the coupling of **33** with eight non‐radioactive fluorine‐containing (hetero)aryl iodides (see Supporting Information **Section 3.3**). This HTE assay led to the identification of four ligands selected for generality across the sample set (bpyCAM, *^t^* BuPyBCAM, PyBCAM, *^t^* BubpyCAM^CN^). [^18^F]2‐Fluoro‐1‐iodobenzene ([^18^F]**37**) and [^18^F]4‐fluoro‐1‐iodobenzene ([^18^F]**5**) were selected for radiochemical validation with their respective optimal ligands identified in HTE since variation of fluorine substitution is prevalent in the design of novel radioligands and ^18^F‐SAR studies (e.g., [^18^F]OB‐NB1).^[^ ^43^ ^]^ Validation of the top 5 ligands for **5** and **37** identified by HTE led to improved RCCs of both [^18^F]**41** and [^18^F]**42** from 40% to 79% and from 31% to 86%, respectively (Scheme 4). Additionally, inefficient ligands identified in HTE were found to exhibit analogously low reactivity under radiochemical conditions, corroborating the valuable insights that can be gained from our developed ^19^F‐HTE assay for ^18^F‐reactivity (see Supporting Information **Section 3.3.4**).

### Automated Synthesis

**Scheme 5:**
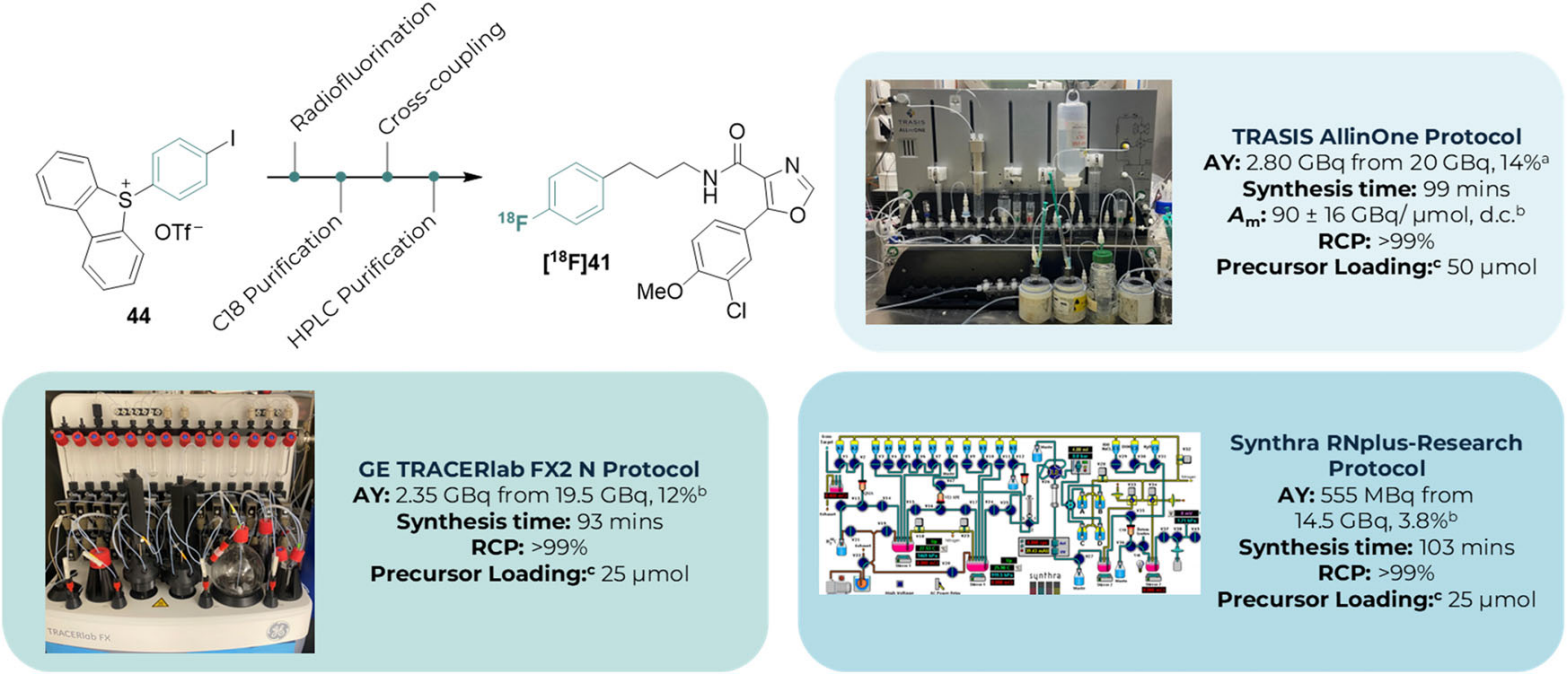
Automation of two‐step, 1‐HPLC process across three commercial automated synthesis platforms. ^a)^ Using *^t^* BuPyBCAM as ligand. ^b)^ Using PyBCAM as ligand. ^c)^ Precursor = 2,4,6‐triphenylpyridinium precursor (**33**). AY = activity yield. n.d.c = non‐decay corrected. *A*_m_ = molar activity. RCP = radiochemical purity. d.c. = decay‐corrected to the end‐of synthesis.

For a new radiosynthetic method to be useful for PET imaging studies, ideally it would provide GBq‐quantities of the target tracer in suitable molar activity (*A*_m_), formulated for injection.

(Pre)clinical radiochemistry facilities most often rely on commercial automated synthesis platforms for accessing these formulated radiopharmaceuticals. We therefore sought to demonstrate automation of the developed two‐step process (Scheme 5). A semi‐automated process was first developed to prepare [^18^F]**41** using a TRASIS AllinOne automated synthesiser. To eliminate the need for two separate HPLC purification steps (*i.e*., HPLC purification of the ^18^F‐labelled reagent and of the product), we explored the use of onium radiolabelling precursors, which could enable purification of [^18^F]**5** by solid‐phase extraction cartridges (e.g., C18) instead of HPLC. The use of 5‐(4‐iodophenyl)‐5*H*‐dibenzo[*b,d*]thiophen‐5‐ium triflate (**44**)—available in a single step from iodobenzene—as the radiolabelling precursor and PyBCAM as the ligand gave the desired product [^18^F]**41** isolated in an activity yield (AY) of 1.81 GBq from 20 GBq of starting activity (9%), non‐decay corrected (n.d.c.) in >99% radiochemical purity (RCP) and an average *A*_m_ of 90 GBq/µmol.^[^ ^44^ ^]^ Using *^t^* Bu‐PyBCAM, the optimal ligand identified by HTE, in place of PyBCAM delivered [^18^F]**41** in an improved AY of 2.80 GBq from 20 GBq of starting radioactivity (14% n.d.c.), which is sufficient for (pre)clinical imaging studies.

This protocol was further adapted to two additional widely adopted automated synthesisers—a GE TRACERlab FX2 N and a Synthra RNplus‐Research—in a fully automated program even with reduced loadings of **33** (25 µmol). The GE TRACERlab FX2 N protocol gave the desired product [^18^F]**41** isolated in an AY of 2.35 GBq from 19 GBq (12%, n.d.c.) in > 99% radiochemical purity (RCP). The Synthra RNplus‐Research protocol yielded 555 MBq of [^18^F]**41** from 14.5 GBq (3.8% AY, n.d.c.). These results serve as a proof‐of‐concept for preclinical translation of this automated method for immediate use by radiochemists.

## Conclusion

We have developed an aryl (C)*sp^2^*‐(C)*sp^3^* cross‐coupling between amine‐derived redox‐active primary and secondary alkyl pyridinium salts and ^18^F‐labelled fluoro(hetero)aryl iodides. The process is applicable to complex biorelevant targets with excellent functional group tolerance, offering radiochemists a novel strategy to accelerate analogue generation for diagnostic and drug discovery campaigns. A late‐stage diversification of a GSK 3 inhibitor provided expedient access to six structurally diverse ^18^F‐labelled analogues from a single 2,4,6‐triphenylpyridinium precursor. Suitably tailored and radiochemically validated ^19^F‐HTE studies enabled rapid evaluation of ligands prior to radiochemical adoption. Additionally, our reaction was amenable to two‐step (semi‐)automated protocols on TRASIS AllinOne, GE TRACERlab FX2 N, and Synthra RNplus‐Research radiosynthesisers with a single HPLC purification. Radiochemists can now apply aryl (C)*sp^2^*‐(C)*sp^3^* cross‐couplings of ubiquitous primary and secondary amines to expedite the synthesis of ^18^F‐labelled radiotracers for diagnostic and drug discovery campaigns.

## Supporting Information

The authors have cited additional references within the Supporting Information.^[45–67]^

## Conflict of Interests

The authors declare no conflict of interest.

## Supporting information



Supporting Information

## Data Availability

The data that support the findings of this study are available in the Supporting Information of this article.
