# Supplementary material for: 18F‐Radiopharmaceutical Diversification Enabled by Deaminative Cross‐Electrophile Couplings
Source: Angew Chem Int Ed Engl. 2025 Dec 4;65(4):e22650. doi: 10.1002/anie.202522650 (PMC12828466; doi:10.1002/anie.202522650)

Supplementary Information for

^{18}F-Radiopharmaceutical Diversification Enabled by Deaminative Cross-Electrophile Couplings

Isabella F. Ogilvy[1], Joseph Ford[1], Sebastiano Ortalli[1], Evelien Renders[2], Thomas R. Hayes[3], Shuanglong Liu[3], Inne Mortiers[2], Anastasia Nikolopoulou[4], Alexandre M. Sorlin[4], Andrés A. Trabanco[5], Matthew Tredwell[6], Peter J. J. A. Buijnsters[2], Rhys Salter[4], and Véronique Gouverneur[1]*

[1]*Chemistry Research Laboratory, Department of Chemistry, 12 Mansfield Road, Oxford, OX1 3TA, United Kingdom.*

[2]*Johnson & Johnson, Global Discovery Chemistry, Turnhoutseweg 30, 2340, Beerse, Belgium*

[3]*Johnson & Johnson, Global Discovery Chemistry, La Jolla, California 92121, United States.*

[4]*Johnson & Johnson, Global Discovery Chemistry, Spring House, Pennsylvania 19477, United States.*

[5]*Johnson & Johnson, Global Discovery Chemistry, Janssen-Cilag, S.A., E-45007 Toledo, Spain.*

[6]*Wales Research and Diagnostic PET Imaging Centre, Cardiff University, University Hospital of Wales, Heath Park, Cardiff CF14 4XN, United Kingdom. School of Chemistry, Cardiff University, Main Building, Park Place, Cardiff CF10 3AT, United Kingdom.*

*Correspondence should be addressed to Véronique Gouverneur at veronique.gouverneur@chem.ox.ac.uk

Contents

1. Radiochemistry 3
1.1 General Experimental Details 3
1.2 HPLC Columns and Eluent Systems 4
1.3 Automated Synthesis of [^{18}F]5 from Aryl Boronic Ester using a Trasis AllinOne Synthesizer 5
1.4 Manual Radiosynthesis of [^{18}F]1 and [^{18}F]5 from Aryl Boronic Esters 8
1.5 General Procedure for Screening Scale Deaminative Cross-Coupling with [^{18}F]5 9
1.6 Optimization of Deaminative Cross-Coupling with ^{18}F-Radiolabelled Aryl Halides 11
1.7 Investigation of Alternative Alkyl Coupling Partners and Reduction Methods for Cross-Coupling with ^{18}F-Fluoroaryl Halides 24
1.8 Unsuccessful Alkyl Pyridinium Coupling Partners 27
1.9 Radiosynthesis of ^{18}F-Radiolabelled Aryl Halide Coupling Partners 28
1.10 Automated Syntheses of ^{18}F-Radiolabelled Aryl Halide Coupling Partners on TRASIS AllinOne 33
1.11 TRASIS AllinOne Semi-Automated Synthesis of [^{18}F]41 41
1.12 Considerations for Adapting 2-Step Radiofluorination/Cross-Coupling to GE TRACERlab FX2 N and Synthra RNPlus Research 50
1.13 GE TRACERlab FX2 N Automated Synthesis of [^{18}F]41 51
1.14 Synthra RNPlus Research Automated Synthesis of [^{18}F]5-(3-chloro-4-methoxyphenyl)-*N*-(3-(4-fluorophenyl)propyl)oxazole-4-carboxamide 57
1.15 Radiotrace Overlays and Radiochemical Conversions 62
2.1 General Information for Synthesis 86
2.2 Preparation of Alkyl 2,4,6-Triphenylpyridinium Salts 88
2.3 Preparation of Aryl Iodide Radiolabelling Precursors 108
2.4 Preparation of Reference Compounds 110
3 High-Throughput Experimentation (HTE) 135
3.1 General Information for HTE 135
3.2 General Method for HTE Assay 136
3.3 HTE Ligand Screens 138
3.4 High-Throughput Synthesis and Purification for Characterisation of Products Observed in HTE 143
4 References 148
5 NMR Spectra of Novel Compounds 153

1. Radiochemistry

1.1 General Experimental Details

Caution! Due to the spontaneous fission and β^+ and γ particle emission from fluorine-18, the radioisotope sample represents a serious health hazard. All studies with fluorine-18 were conducted in a radiation laboratory equipped with appropriate lead-shielded hot cells.

See "General information for synthesis" for the preparation and storage of chemicals.

Radiochemical conversion (%RCC) as determined by RadioHPLC by integration of the desired ^{18}F-product relative to the total peak area for all radioactive species observed. Identity of the ^{18}F-product was confirmed by matching of the UV-HPLC retention time of an authentic ^{19}F-reference. Activity yield (AY) is the non-decay corrected (n.d.c.) isolated activity of radiochemical product from given starting activity. All molar activities are decay corrected until the end of synthesis (EOS) unless stated otherwise.

For manual radiosynthesis of ^{18}F-radiolabelled aryl iodides and cross-coupling optimization: [^{18}F]Fluoride was produced by Alliance Medical (UK), Invicro (UK) or PETIC (UK) via the $^{18}O_{(p,n)}{}^{18}F$ reaction and delivered as [^{18}F]fluoride in [^{18}O]water. Radiosynthesis and azeotropic drying were performed on a NanoTek® automated microfluidic device (Advion). Analytical HPLC analysis was performed on a Dionex Ultimate 3000 dual channel HPLC system equipped with a shared autosampler, parallel UV-detectors and LabLogic NaI/PMT-radiodetectors with Flow-RAM analogue output. Due to the separation of the modules the radio-signal is offset by 0.1-0.3 minutes from the UV signal.

For automated radiosynthesis of ^{18}F-radiolabelled (heteroaryl)aryl iodides and scope entries: [^{18}F]Fluoride was produced in an IBA Cyclon 18/9 cyclotron using the $^{18}O_{(p,n)}{}^{18}F$ reaction. A TRASIS AllinOne radiosynthesizer (TRASIS) was used for all automated synthesis including semi-preparative purification of the radiolabelled reagents and products using the integrated HPLC system (including UV and Radiodetectors). Analytical Radio-high-performance liquid (radio-HPLC) chromatography was performed on an Agilent 1200 equipped with a LabLogic gamma-RAM Model 4 detector. Due to the separation of the modules, the radio-signal is offset by 0.1 minute from the UV signal.

1.2 HPLC Columns and Eluent Systems

Preparative HPLC column conditions for (semi-)automated and manual synthesis protocols are specified in the relevant sections.

Analytical HPLC Condition A [standard] – Agilent Zorbax Eclipse Plus C18 80Å 150 x 4.6 mm LC column. **Eluent =** 0 to 1 min [isocratic, 25% MeCN]; 1 to 10 min [linear gradient, 25% - 95% MeCN]; 10 to 16 min [isocratic, 95% MeCN]; 16 to 18 min [linear gradient, 95% - 25% MeCN]; 18 to 20 min [isocratic, 25% MeCN] isocratic. **Flow rate** = 1.0 mL/min. **Temperature** = 25 °C. **Wavelength** = 220 nm unless otherwise specified.

Analytical HPLC Condition B [5% – 95% gradient] – Agilent Zorbax Eclipse Plus C18 80Å 150 x 4.6 mm LC column. **Eluent** = 0 to 1 min [isocratic, 5% MeCN]; 1 to 10 min [linear gradient, 5% - 95% MeCN]; 10 to 16 min [isocratic, 95% MeCN]; 16 to 18 min [linear gradient, 95% - 5% MeCN]; 18 to 20 min [isocratic, 5% MeCN] isocratic. **Flow rate** = 1.0 mL/min. **Temperature** = 25 °C. **Wavelength** = 220 nm unless otherwise specified.

Analytical HPLC Condition C [5% – 95% and TFA] – Agilent Zorbax Eclipse Plus C18 80Å 150 x 4.6 mm LC column. **Eluent** = 0 to 1 min [isocratic, 5% MeCN+0.1% TFA]; 1 to 10 min [linear gradient, 5% - 95% MeCN+0.1% TFA]; 10 to 16 min [isocratic, 95% MeCN+0.1% TFA]; 16 to 18 min [linear gradient, 95% - 5% MeCN+0.1% TFA]; 18 to 20 min [isocratic, 5% MeCN+0.1% TFA] isocratic. **Flow rate** = 1.0 mL/min. **Temperature** = 25 °C. **Wavelength** = 220 nm unless otherwise specified.

Analytical Condition D [5% – 95% Chromolith] Chromolith® Performance RP-18 end capped 100-4.6 HPLC column. **Eluent** = 0 to 1 min [isocratic, 5% MeCN]; 1 to 10 min [linear gradient, 5% - 95% MeCN]; 10 to 16 min [isocratic, 95% MeCN]; 16 to 18 min [linear gradient, 95% - 5% MeCN]; 18 to 20 min [isocratic, 5% MeCN] isocratic. **Flow rate** = 1.0 mL/min. **Temperature** = 25 °C. **Wavelength** = 220 nm unless otherwise specified.

Analytical Condition E [20-minute standard gradient]– Agilent Zorbax Eclipse Plus C18 80Å 150 x 4.6 mm LC column. **Eluent =** 0 to 1 min [isocratic, 25% MeCN]; 1 to 21 min [linear gradient, 25% - 95% MeCN]; 21 to 26 min [isocratic, 95% MeCN]; 26 to 28 min [linear gradient, 95% - 25% MeCN]; 28 to 30 min [isocratic, 25% MeCN] isocratic. **Flow rate** = 1.0 mL/min. **Temperature** = 25 °C. **Wavelength** = 220 nm unless otherwise specified.

Analytical HPLC Condition F [standard] – Phenomenex SynergiTM 4 µm Hydro RP 80Å 150 x 4.6 mm LC column. **Eluent =** 0 to 1 min [isocratic, 25% MeCN]; 1 to 10 min [linear gradient, 25% - 95% MeCN]; 10 to 14 min [isocratic, 95% MeCN]; 14 to 17 min [linear gradient, 95% - 25% MeCN]; 17 to 19.4 min [isocratic, 25% MeCN] isocratic. **Flow rate** = 1.0 mL/min. **Temperature** = 25 °C. **Wavelength** = 220 nm unless otherwise specified.

1.3 Automated Synthesis of [^{18}F]5 from Aryl Boronic Ester using a Trasis AllinOne Synthesizer

The radiosynthesis of [^{18}F]1-fluoro-4-iodobenzene ([^{18}F]**5**) was automated on a TrasisAllinOne synthesizer utilizing a prebuilt cassette (Figure S1).

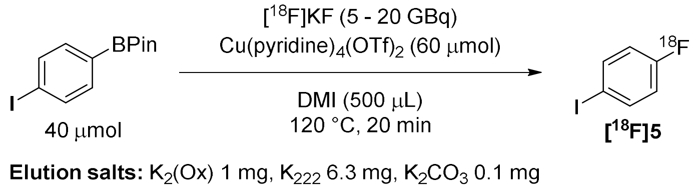

Cassette Preparation:

Position 2: Vial containing K_2(oxalate).H_2O (1 mg), K_2CO_3 (0.1 mg), K_{222} (6.3 mg) in H_2O (110 μL) and MeCN (500 μL).

Position 5: Waters Sep-Pak AccellPlus QMA Carbonate Plus Light Cartridge preconditioned with H_2O (10 mL).

Position 8: MeCN (10 mL) solvent reservoir for azeotropic drying of [^{18}F]fluoride.

Position 9: 2-(4-Iodophenyl)-4,4,5,5-tetramethyl-1,3,2-dioxaborolane (13 mg, 40 μmol) in anhydrous DMI (500 μL).

Position 10: Tetrakis(pyridine)copper(II) triflate (41 mg, 60 μmol) in DMI (500 μL).

Position 17: Anhydrous NMP (10 mL) solvent reservoir for elution of **[^{18}F]5** post-HPLC.

Position 33: Waters Sep-Pak C18 Plus cartridge preconditioned with EtOH (10 mL) and then H_2O (10 mL).

Position 34: H_2O (25 mL) reservoir for reaction dilution prior to HPLC loading.

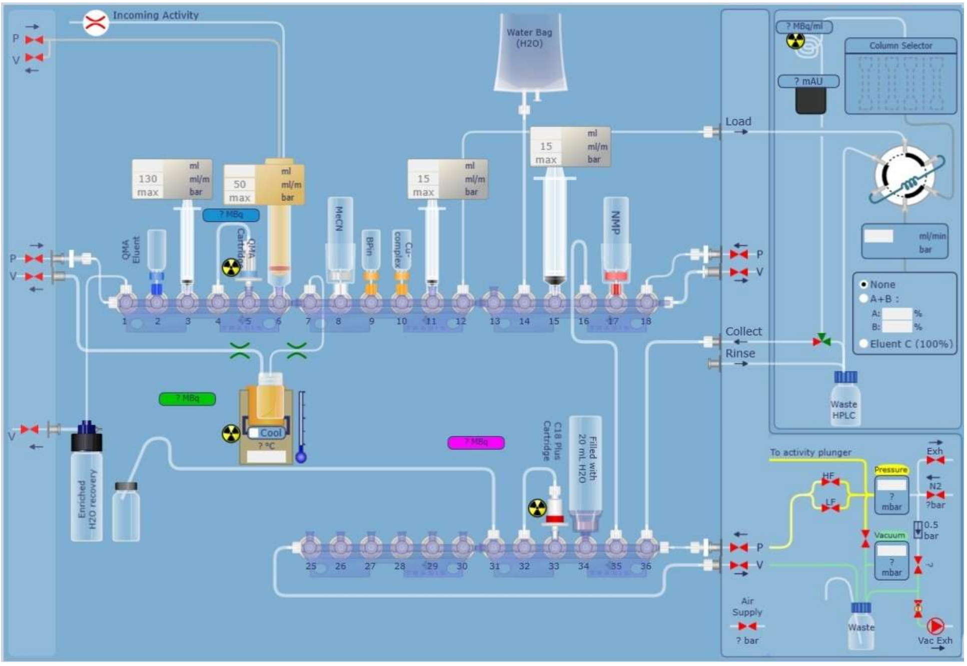

Figure S1: Cassette layout for the automated synthesis of [^{18}F]**5** on the Trasis AllinOne Platform. The figure was generated using the Trasis software.

Method

Reaction: [^{18}F]Fluoride in [^{18}O]H_2O was received from the cyclotron and subsequently trapped on a QMA cartridge (position 5). The elution salts (position 2) were passed through the QMA cartridge eluting [^{18}F]fluoride which was dried azeotropically in the reaction vial by heating to 120 °C with MeCN under a flow of N_2. Tetrakis(pyridine)copper(II) triflate (position 10) and 2-(4-iodophenyl)-4,4,5,5-tetramethyl-1,3,2-dioxaborolane (position 9) were transferred into the reaction vial and heated to 120 °C for 20 minutes.

Purification and Reformulation: Dilution of the reaction mixture with water was done prior to loading of the HPLC sample loop. Purification of the crude reaction mixture was achieved by semi-preparative reverse phase HPLC t_R = 10.5 - 12.5 min. A 1:1 mixture of the isolated product eluate in H_2O was loaded onto a C18 cartridge (position 33). The C18 cartridge was subsequently washed with H_2O (10 mL) followed by an air purge. [^{18}F]**5** was eluted from the C18 cartridge with NMP (2 mL) into a vial and the activity yield of the collected product measured. The solution [^{18}F]**5** was used as a stock solution for performing screening reactions.

Semi-preparative purification of [^{18}F]5 – Phenomenex 5 μm 250 x 10 mm LC column. Eluent = isocratic 65:35 MeCN:H_2O. Flow rate = 4.0 mL/min. Temperature = 25 °C. Wavelength = 254 nm.

Table S1: Activity Yields of [^{18}F]**5** by TRASIS AllinOne Automated Synthesis. Isolated RCY.

Entry	Starting Activity (GBq)	Activity Yield (AY) (GBq)	RCY (%)
**1**	20	6.8	34
**2**	10	2.8	28
**3**	10	2.1	21
**4**	10	3.1	31
**5**	10	2.1	21
**6**	10	1.9	19
**7**	5	1	20
**8**	5	1.9	38
**9**	10	1.08	11
**10**	5	1.57	31
**11**	5	0.80	16
**12**	5	1.1	22
**RCY (%) Average:**	24	**RCY (%) Standard Deviation:**	8

1.4 Manual Radiosynthesis of [^{18}F]1 and [^{18}F]5 from Aryl Boronic Esters

The synthesis of ^{18}F-radiolabelled aryl halide coupling partners was adapted from work by Gouverneur and co-workers.[28]

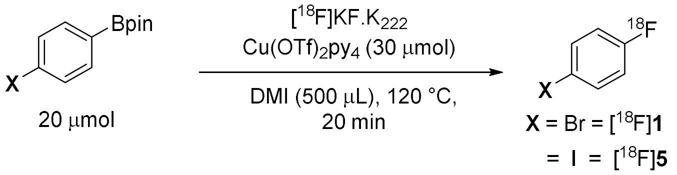

[^{18}F]Fluoride was separated from ^{18}O-enriched-water using an anion exchange cartridge [Waters Sep-Pak AccellPlus QMA Carbonate Plus Light Cartridge, activated with H_2O (10 mL) prior to use] and released with a solution of Kryptofix (6.3 mg), $K_2C_2O_4$ (1.0 mg) and K_2CO_3 (0.1 mg) in MeCN/H_2O (0.6 mL, 5:1, v/v), which was concentrated by azeotropic drying with additional MeCN (3 x 0.7 mL) in a 5 mL v-vial equipped with a stirrer bar.

Once drying was complete, a solution of Aryl Bpin precursor (20 µmol) and Cu(OTf)$_2$py$_4$ (20.3 mg, 30 µmol) in DMI (500 µL) was added by syringe. The vial was then purged with air (20 mL), and the mixture was stirred at 120 °C for 20 mins. Once this time had elapsed, the vial was left to cool, and the reaction mixture was diluted with H_2O (4 mL). The mixture was then taken up in a syringe and loaded onto a C18 cartridge [Waters Sep-Pak C18 Plus Cartridge, activated with EtOH (10 mL) and H_2O (10 mL) prior to use]. The cartridge was then eluted with MeCN (1.2 mL), and the eluate was transferred to the HPLC sample loop. The crude material was purified by semi-preparative HPLC (MeCN:H_2O = 65:35, Flow = 4 mL/min, Phenomenex Gemini LC column, 250 x 10 mm). The fraction containing the ^{18}F-radiolabelled product was collected into a vial containing H_2O (50 mL). The vial was shaken, and the liquid was passed over a C-18 cartridge [Waters Sep-Pak C18 Plus Cartridge, activated with EtOH (10 mL) and H_2O (10 mL) prior to use]. Air (5 mL) was then passed over the cartridge to remove residual H_2O and it was then eluted with NMP (1.5 mL) to yield the ^{18}F-radiolabelled aryl halide coupling partner.

[^{18}F]1: Precursor = 2-(4-bromophenyl)-4,4,5,5-tetramethyl-1,3,2-dioxaborolane (5.6 mg, 20 µmol). **AY** = 5 ± 1% [n = 3], starting activity ranging from 1.52 to 13.5 GBq *(reformulation unoptimized)*. t_R = 10 - 12 mins.

[^{18}F]5: Precursor = 2-(4-iodophenyl)-4,4,5,5-tetramethyl-1,3,2-dioxaborolane (6.6 mg, 20 µmol). **AY** = 27 ± 10% [n = 4], starting activity ranging from 6.75 to 20 GBq. t_R = 10.5 - 12.5 min.

1.5 General Procedure for Screening Scale Deaminative Cross-Coupling with [^{18}F]5

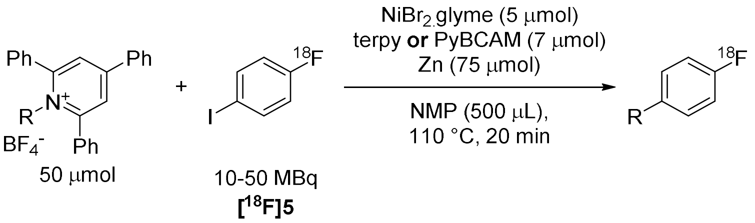

A stock solution of $NiBr_2$.glyme and ligand in anhydrous NMP was prepared in a 14 mL septum-sealed vial, as below.

	**Nickel Source**	**Ligand**	**Solvent**
**Stock solution**	$NiBr_2$.glyme<br>(30.9 mg, 100 µmol)	2,2':6',2''-terpyridine<br>(32.7 mg, 140 µmol)<br>**or**<br>Pyridine-2,6-bis(carboximidamide) dihydrochloride<br>(33.0 mg, 140 µmol)	*N*-Methyl pyrrolidine<br>(10 mL)
**Reaction mixture**<br>(500 µL of stock solution)	$NiBr_2$.glyme<br>(1.5 mg, 5 µmol)	2,2':6',2''-terpyridine<br>(1.6 mg, 7 µmol)<br>**or**<br>Pyridine-2,6-bis(carboximidamide) dihydrochloride<br>(1.7 mg, 7 µmol)	*N*-Methyl pyrrolidine<br>(500 µL)

The stock solution was used for no more than nineteen reactions to account for loss of solution in the dead volume of syringes. The stock solution was degassed by fitting a vent needle through the septum and bubbling N_2 through the solution using a N_2-filled balloon while sonicating the vial for 1 minute. The solution was prepared on day of use and stored with an N_2-filled balloon attached to a needle fitted through the septum.

Into an oven-dried, 4 mL flat-bottomed vial with a magnetic stir bar was weighed zinc powder (5 mg, 75 µmol), followed by the addition of the triphenylpyridinium tetrafluoroborate salt (50 µmol). The nickel stock solution (500 µL/reaction) was added to the reaction vial. The reaction vial was sealed with an open-top polypropylene screw-top cap fitted with a PTFE/silicone septum. [^{18}F]**5** (10-50 MBq) in NMP (approx. 50 µL) was subsequently added to the reaction vial and the mixture stirred at 110 °C for 20 minutes. The reaction vial was allowed to cool to room temperature and the reaction mixture was then diluted with MeCN (300 µL) and an aliquot was analysed by RadioHPLC. If TFA was required as an additive for the analytical HPLC eluent system, the reaction mixture was diluted with MeCN + 0.1% TFA (300 µL).

Considerations for Reaction Set-up:

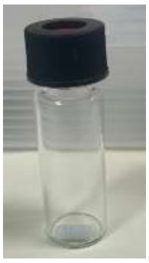

The standard set-up used was as follows: Flat bottom 4 mL vial (1-dram, 45 x 14.75 mm) with open-top polypropylene screw-top cap fitted with PTFE/silicone septum and cylindrical stirrer bar. (Pictured right).

This was chosen due to the heterogenous reaction mixture. However, standard 3- or 5-mL V-vials were shown to give comparable results. Stirring was required for the reaction to proceed (Table S2).

Table S2: Consideration of stirring and reaction vial on cross-coupling of **2** and [^{18}F]**5**. All entries n = 1.

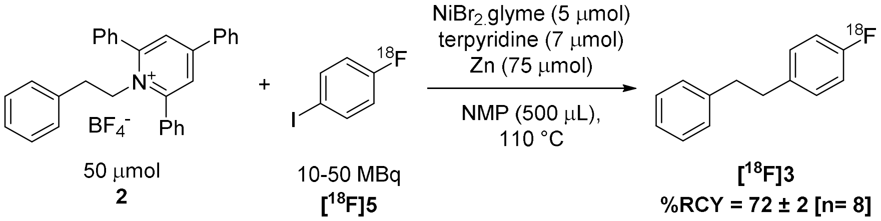

Entry	Deviation from Optimized Conditions	%RCC of [^{18}F]3
1	No stirring	37
2	No stirring (V-Vial)	15
3	V-vial + stirring	62
4	V-vial + spin vane	56

1.6 Optimization of Deaminative Cross-Coupling with ^{18}F-Radiolabelled Aryl Halides

1.6.1 General Procedure for Optimization of Deaminative Cross-Coupling with [^{18}F]1 or [^{18}F]5

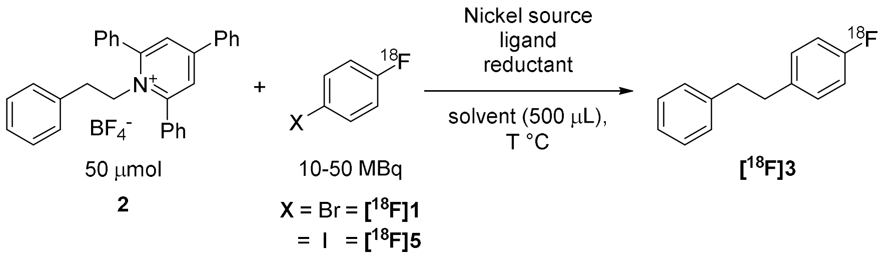

A stock-solution of nickel salt and ligand in anhydrous solvent was prepared in an oven-dried vial sealed with a suba-seal or PTFE cap with teflon septum and degassed for 5-10 minutes by sparging with N_2 gas while sonicating the vial. To an oven-dried, 4 mL flat-bottomed vial with a magnetic stir bar was added the reductant and alkyl 2,4,6-triphenylpyridinium salt (**3**). The vial was sealed by an open-top polypropylene screw-top cap fitted with a PTFE/silicone septum. Nickel stock solution was added to the reaction vial containing the substrate and reductant. For screening of the nickel sources and ligands, the nickel source and ligand were weighed – a stock solution was not used. ^{18}F-Radiolabelled aryl halide coupling partner (10-50 MBq) in NMP (approx. 50 μL) was dispensed into this vial and stirred at the stated temperature for 20 minutes. The reaction mixture was then diluted with MeCN (500 μL) and an aliquot of the crude reaction mixture analyzed by RadioHPLC (Conditions A – F Section 1.2).

1.6.2 Exemplary crude RadioHPLC trace superimposed with UV-HPLC trace of authentic samples

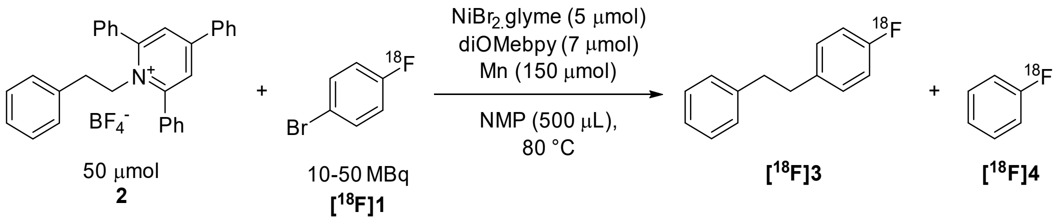

Figure S2: Representative UV and Radio chromatogram trace overlays of [^{18}F]**1**, [^{18}F]**3**, and [^{18}F]**4** with their corresponding non-radioactive references.

Analytical HPLC condition F. Wavelength for measurement of **4** was 200 nm.

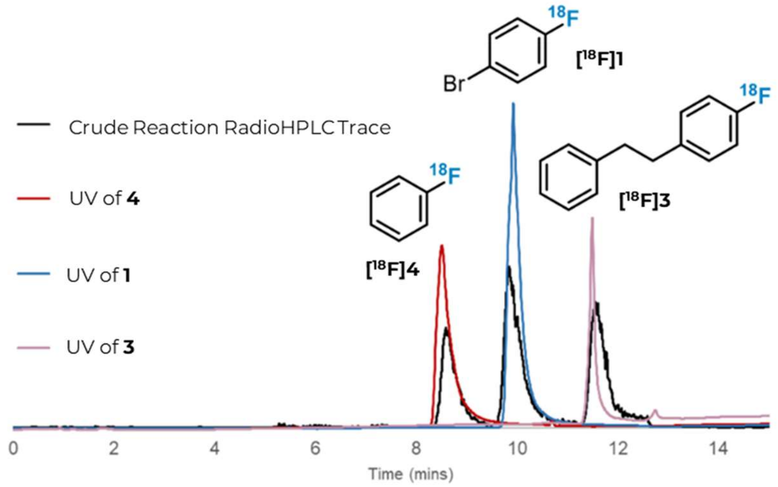

1.6.3 Table of Values for Manuscript Scheme 1 Bar Graph

Scheme S1: Manuscript Scheme 1 Bar Graph. DiOMebpy = 4-4'-Dimethoxy-2-2'-bipyridine Terpy = 2,2';6',2"-terpyridine.

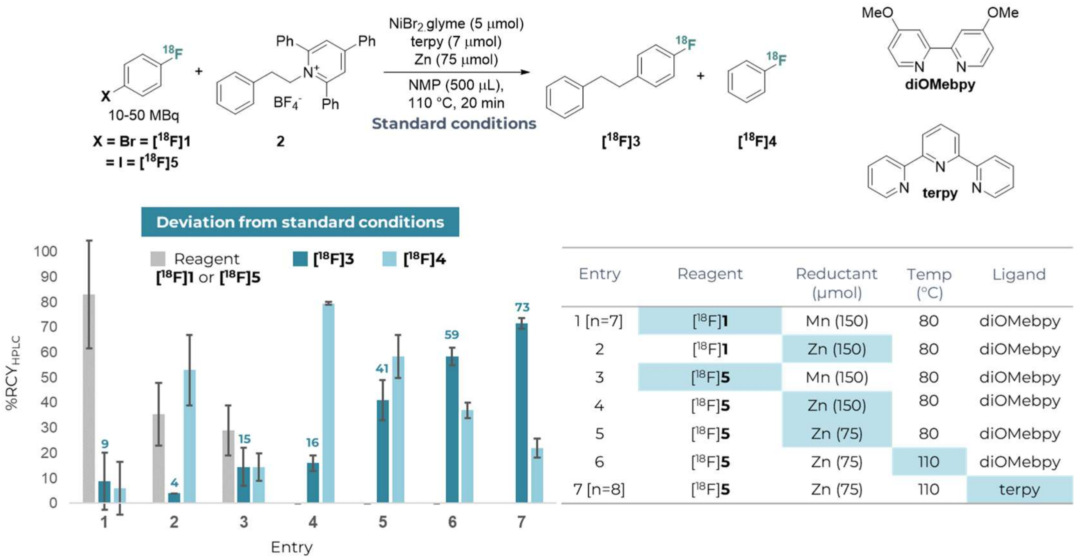

Table S3: Entries and error values for Manuscript Scheme 1 Bar Graph. 1.# = individual experimental results for Entry 1 all n = 1.

Entry	%RCC [18F]3	%RCC [18F]4	%RCC [18F]1 or 5
**1** [n = 7]	9 ± 11	6 ± 10	83 ± 21
**1.1**	0	0	100
**1.2**	26	29	45
**1.3**	7	0	82
**1.4**	0	0	100
**1.5**	0	0	100
**1.6**	2	0	98
**1.7**	27	13	57
**2**	4 ± 0	53 ± 14	36 ± 13
**3**	15 ± 8	15 ± 6	29 ± 10
**4**	16 ± 3	80 ± 1	0 ± 0
**5**	41 ± 8	59 ± 9	0 ± 0
**6**	59 ± 4	37 ± 3	0 ± 0
**7** [n = 8]	73 ± 2	22 ± 4	0 ± 0

1.6.4 Optimization of Radiochemical Cross-coupling of Primary Alkyl 2,4,6-Triphenylpyridinium Salts with ^{18}F-Fluoroaryl Halides

Table S4: Optimization of reductants in deaminative cross-coupling with [^{18}F]**1**. All entries n = 2 unless specified. TDAE = Tetrakis(dimethylamino)ethylene. DBTTF = Dibenzotetrathiafulvalene.

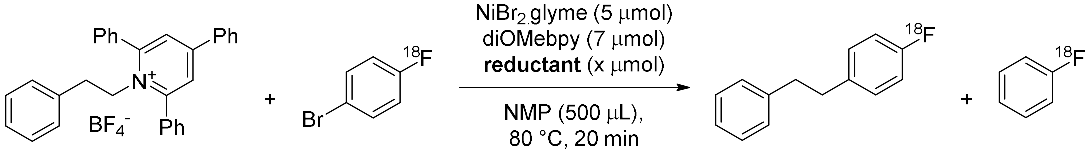

50 μmol **2** | 10-50 MBq **[^{18}F]1** | **[^{18}F]3** | **[^{18}F]4**

Entry	Reductant	Reductant µmol	%RCC [^{18}F]3	%RCC [^{18}F]4	%RCC [^{18}F]1
**1** [n=7]	Mn	150	9 ± 11	6 ± 10	83 ± 21
**2** [n=6]	Mn	300	20 ± 18	17 ± 20	63 ± 38
**3** [n=1]	Mn	75	33	60	0
**4** [n=2]	Mn	600	24 ± 24	24 ± 24	53 ± 47
**5**	TDAE	150	0 ± 0	0 ± 0	100 ± 0
**6**	DBTTF	150	0 ± 0	0 ± 0	100 ± 0
**7**	Zn	150	4 ± 0	53 ± 14	36 ± 13

Table S5: Optimization of reductants in deaminative cross-coupling with [^{18}F]**5**. All entries n = 2. TDAE = Tetrakis(dimethylamino)ethylene. DBTTF = Dibenzotetrathiafulvalene. TTF = Tetrathiafulvalene.

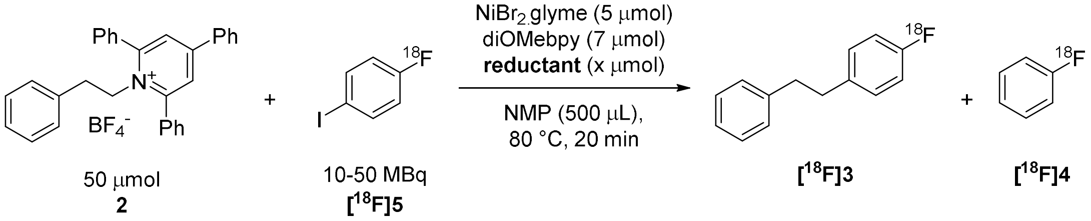

Entry	Reductant	Reductant µmol	%RCC [^{18}F]3	%RCC [^{18}F]4	%RCC [^{18}F]5
**1**	Mn	150	15 ± 8	15 ± 6	29 ± 10
**2**	Zn	150	16 ± 3	80 ± 1	0 ± 0
**3**	Zn	75	41 ± 8	59 ± 9	0 ± 0
**4**	Zn	50	35 ± 11	61 ± 16	0 ± 0
**5**	Zn	37.5	31 ± 6	69 ± 6	0 ± 0
**6**	TDAE	150	0 ± 0	0 ± 0	100 ± 0
**7**	DBTTF	150	0 ± 0	1 ± 0	99 ± 0
**8**	TTF	150	0 ± 0	1 ± 0	99 ± 0

Table S6: Optimization of solvent in deaminative cross-coupling with [^{18}F]**5**. All entries n = 2 unless specified. [1] Ligand = terpy; reaction performed at 110 °C.

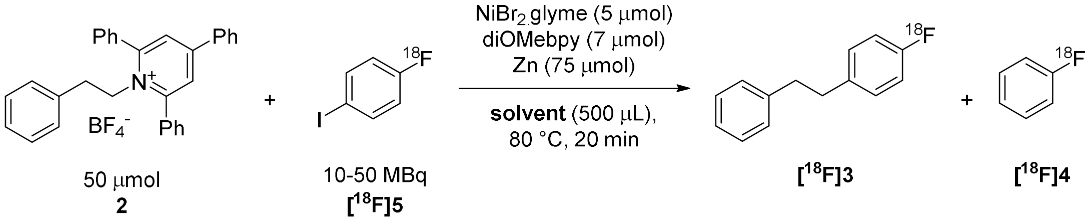

Entry	Solvent	%RCC [^{18}F]3	%RCC [^{18}F]4	%RCC [^{18}F]5
**1**	NMP	41 ± 8	59 ± 9	0 ± 0
**2**	THF	22 ± 4	28 ± 28	22 ± 22
**3**	DMA	26 ± 4	68 ± 6	0 ± 0
**4**	DMI	20 ± 8	30 ± 2	0 ± 0
**5**	DMF	34 ± 6	58 ±15	0 ± 0
**6** [n = 1]	DMSO[1]`	26	39	14
**7** [n = 1]	MeCN[1]	3	19	78

Table S7: Optimization of nickel source/ligand loading in deaminative cross-coupling with [^{18}F]**5**. All entries n = 2.

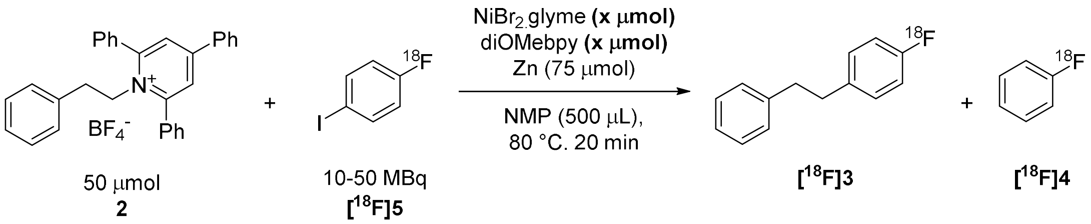

Entry	Ni : Ligand Loading (µmol)	%RCC [^{18}F]3	%RCC [^{18}F]4	%RCC [^{18}F]5
**1**	5 : 7	41 ± 8	59 ± 9	0 ± 0
**2**	2.5 : 3.5	12 ± 1	89 ± 1	0 ± 0
**3**	10 : 14	23 ± 4	40 ± 9	0 ± 0

Table S8: Optimization of temperature using diOMebpy as ligand in deaminative cross-coupling with [^{18}F]**5**. All entries n = 2.

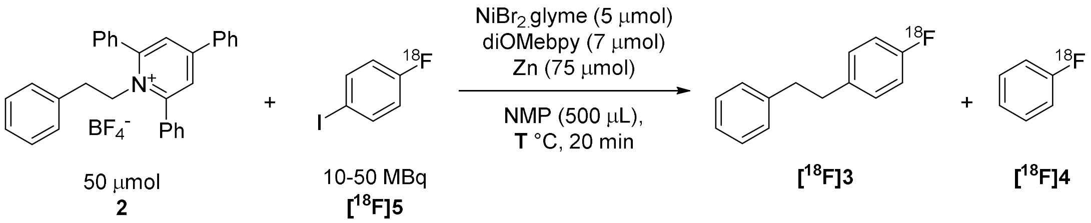

Entry	Temperature (°C)	%RCC [^{18}F]3	%RCC [^{18}F]4	%RCC [^{18}F]5
**1**	60	13 ± 4	50 ± 17	24 ± 24
**2**	80	41 ± 8	59 ± 9	0 ± 0
**3**	90	48 ± 6	52 ± 5	0 ± 0
**4**	100	50 ± 8	45 ± 3	0 ± 0
**5**	110	59 ± 4	37 ± 3	0 ± 0
**6**	120	58 ± 3	43 ± 3	0 ± 0

Table S9: Optimization of temperature using terpy as ligand in deaminative cross-coupling with [^{18}F]**5**. All entries n = 2 unless specified. Terpy = 2,2':6',2''-Terpyridine

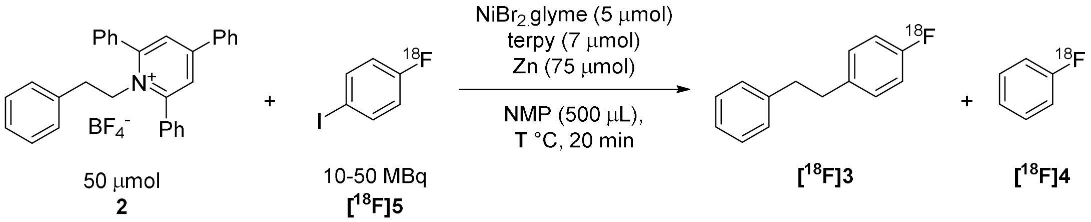

Entry	Temperature (°C)	%RCC [^{18}F]3	%RCC [^{18}F]4	%RCC [^{18}F]5
**1** [n=1]	21	4	10	53
**2**	50	13 ± 6	21 ± 6	27 ± 16
**3**	80	64 ± 2	18 ± 6	0
**4** [n=8]	110	73 ± 2	22 ± 4	0

Table S10: Optimization of nickel source ligand in deaminative cross-coupling with [^{18}F]**5**. All entries n = 2. Ni(COD)(DQ) = Bis(1,5-cyclooctadiene)(duroquinone) nickel(0).

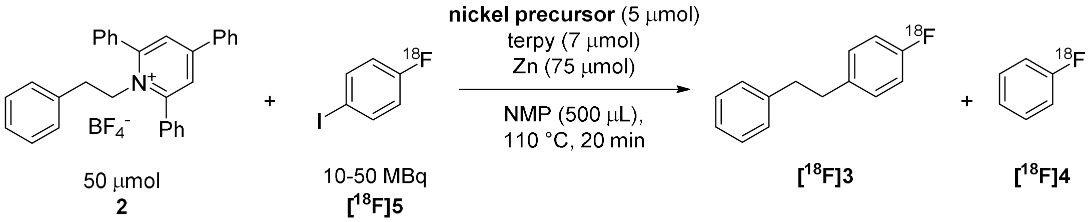

Entry	Ni Precursor	%RCC [^{18}F]3	%RCC [^{18}F]4	%RCC [^{18}F]5
**1**	$NiBr_2$.glyme	59 ± 4	37 ± 3	0 ± 0
**2**	$Ni(acac)_2$	10 ± 2	91 ± 2	0 ± 0
**3**	$Ni(OAc)_2.4H_2O$	2 ± 0	98 ± 0	0 ± 0
**4**	Ni(COD)(DQ)	1 ± 0	46 ± 0	53 ± 1

Table S11: Optimization of reaction concentration in deaminative cross-coupling with [^{18}F]**5**. All entries n = 2.

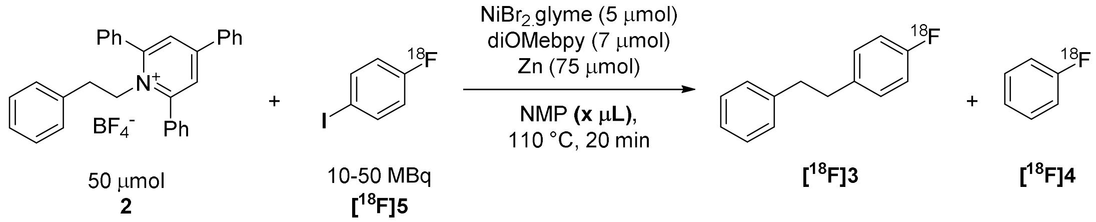

Entry	Volume of NMP (µL)	%RCC [^{18}F]3	%RCC [^{18}F]4	%RCC [^{18}F]5
**1**	500	59 ± 4	37 ± 3	0 ± 0
**2**	1000 [0.5x concentration]	43 ± 6	40 ± 6	0 ± 0
**3**	250 [2x concentration]	7 ± 1	45 ± 3	0 ± 0

Table S12: Altering Substrate Loading, Reaction Scale, and Concentration of Model Conditions in deaminative cross-coupling with [^{18}F]**5**. All entries n = 1 unless specified. [1]Everything in reaction scaled down except reagent. [2]Reaction performed in a 3 mL V-Vial.

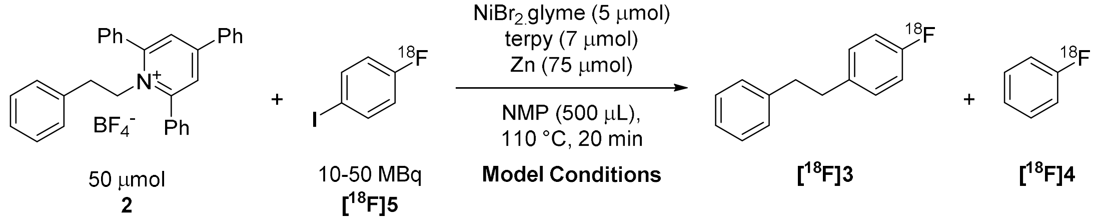

Entry	Deviation from model conditions	%RCC [^{18}F]3	%RCC [^{18}F]4	%RCC [^{18}F]5
**1** [n=8]	None	73 ± 2	22 ± 4	0
**2**	25 µmol substrate (1/2 loading)	5	45	46
**3**	12.5 µmol substrate (1/4 loading)	2	45	51
**4**	0.5x reaction scale[1]	46	22	0
**5**	0.2x reaction scale[1]	Trace	33	47
**6**	1 mL NMP[2] (0.5x concentration)	69	-	-
**7**	1.5 mL NMP[2] (0.33x concentration)	69	-	-
**8**	0.5x reaction scale in 500 µL NMP[2] (0.5x concentration)	47	-	-
**9**	0.2x reaction scale in 500 µL NMP[2] (0.2x concentration)	Trace	-	-

1.6.5 Ligand Structures for Radiochemical Optimization

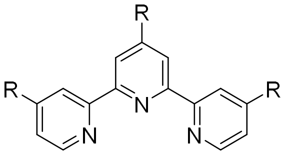

R = ^{t}Bu = ^{t}Bu-terpy
R = H = terpy

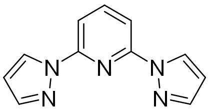

L1

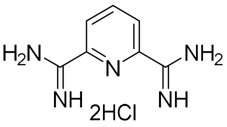

PyBCAM.HCl

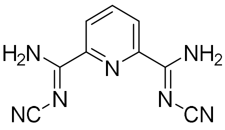

PyBCAM^{CN}

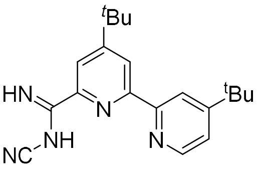

^{t}BubpyCAM^{CN}

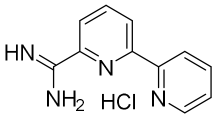

bpyCAM.HCl

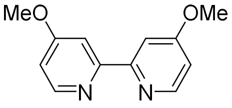

diOMebpy

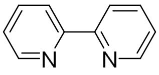

bpy

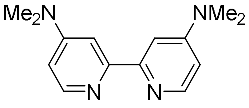

di-NMe$_2$bpy

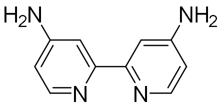

di-NH$_2$bpy

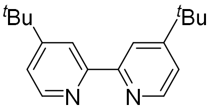

dtbbpy

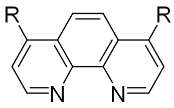

R = Ph = BPhen
R = H = Phen

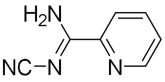

PyCAM^{CN}

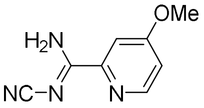

L2

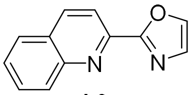

L3

1.6.6 Ligand Optimization for Radiochemical Cross-coupling of Primary Alkyl 2,4,6-Triphenylpyridinium Salts with [^{18}F]5

Table S13: Preliminary ligand screen in deaminative cross-coupling with [^{18}F]**5**. All entries n = 2 unless specified.

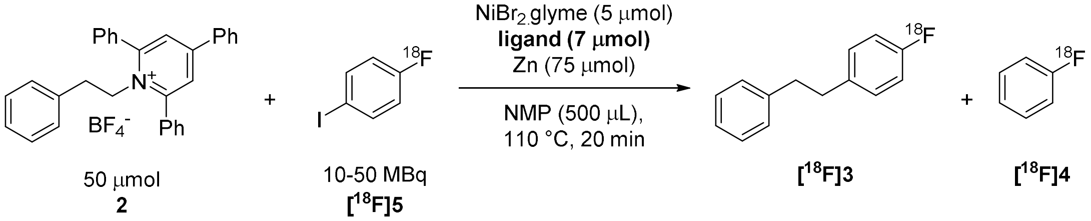

Entry	Ligand	%RCC [^{18}F]3	%RCC [^{18}F]4	%RCC [^{18}F]5
**1**	diOMe-bpy	59 ± 4	37 ± 3	0 ± 0
**2**	BPhen	55 ± 5	35 ± 4	0 ± 0
**3**	phen	49 ± 2	44 ± 1	0 ± 0
**4** [n=8]	terpy	73 ± 2	22 ± 4	0 ± 0
**5**	^{t}Bu-terpy	66 ± 3	7 ± 1	0 ± 0

Table S14: Subsequent ligand screen in deaminative cross-coupling with [^{18}F]**5**. All entries n = 1 unless specified.

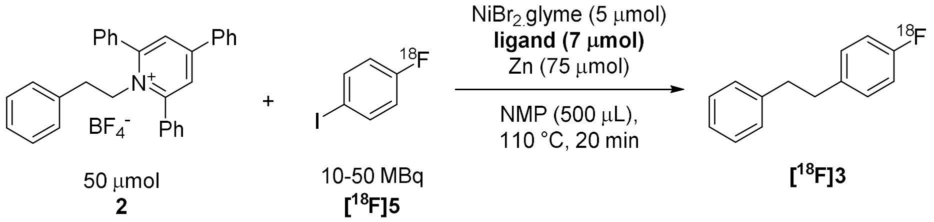

Entry	Ligand	%RCC [^{18}F]3
**1**	PyBCAM^{CN}	52
**2**	^{t}BubpyCAM^{CN}	35
**3** [n = 2]	PyBCAM.HCl	87 ± 2
**4**	L1	32
**5**	BpyCAM.HCl	57
**6**	L2	14
**7**	PyCAM^{CN}	61
**8**	di-NMe$_2$bpy	21
**9**	L3	0
**10**	di-NH$_2$bpy	12
**11**	dtbbpy	17
**12**	bpy	0

1.6.7 Optimization of Deaminative Cross-coupling of Secondary Alkyl 2,4,6-triphenylpyridinium salts with [^{18}F]5

Table S15: Optimization of Deaminative Cross-coupling of Secondary Alkyl 2,4,6-triphenylpyridinium salts with [^{18}F]5. All entries n = 1 unless specified. Loading of ligand was always 7 μmol. *This entry was performed after the identification of ^{t}BuPyBCAM as a highly competent ligand for the cross-coupling of 2,4,6-triphenylpyridinium salts by HTE.

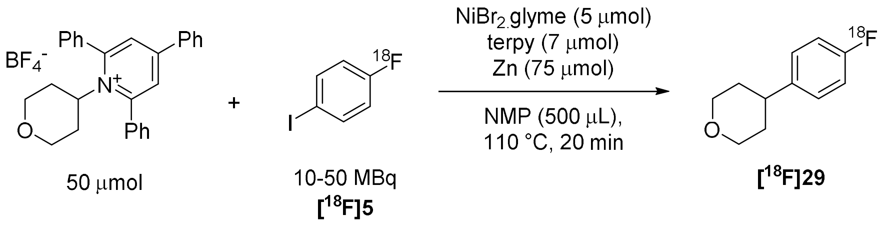

Entry	Deviation from above conditions	%RCC [^{18}F]29
**1**	None	0
**2**	diOMebpy as ligand	0
**3**	di(NMe$_2$)bpy as ligand	0
**4**	Bpy as ligand	0
**5**	^{t}BuCAM^{CN} as ligand	0
**6**	PyBCam^{CN} as ligand	6
**7**	L2 as ligand	0
**8**	PyCam^{CN} as ligand	9
**9**	Dtbbpy as ligand	0
**10** [n = 3]	PyBCAM.HCl as ligand	67 ± 4
**11**	bpyCAM.HCl as ligand	14
**12**	50 °C	0
**13*** [n = 2]	^{t}BuPyBCAM.HCl as ligand	79 ± 4

1.6.8 Control Reactions

Table S16: Control Reactions at 80 °C using diOMebpy as ligand. All entries n = 1 unless specified.

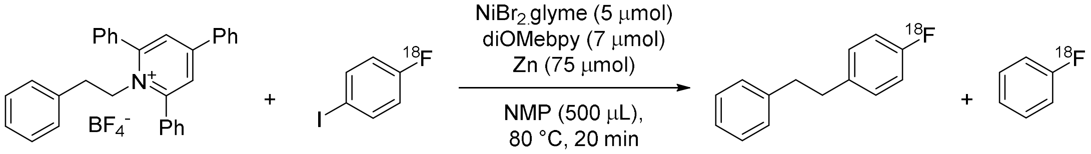

50 μmol **2** | 10-50 MBq **[18F]5** | **[18F]3** | **[18F]4**

Entry	Deviation from above conditions	%RCC [18F]3	%RCC [18F]4	%RCC [18F]5
**1** [n=2]	None	41 ± 8	59 ± 9	0 ± 0
**2**	No Zinc	0	6	94
**3**	No Ni/Ligand	0	15	85
**4**	No substrate/Zinc	-	6	94
**5**	No substrate/Ni/Ligand	-	4	96
**6**	No substrate	-	41	15

Table S17: Control Reactions at 110 °C using terpy as ligand. All entries n = 1 unless specified.

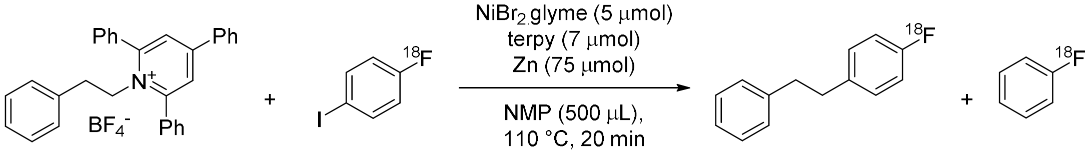

50 μmol **2** | 10-50 MBq **[18F]5** | **[18F]3** | **[18F]4**

Entry	Deviation from above conditions	%RCC [18F]3	%RCC [18F]4	%RCC [18F]5
**1** [n=8]	terpy	73 ± 2	22 ± 4	0 ± 0
**2**	No Zinc	0	2	98
**3**	No substrate/Zinc	0	2	98
**4**	No substrate	0	100	0

1.6.9 Time Course

The model reaction was set up four times as described in **Section 1.5** and reactions stopped at various time points (2 mins, 5 mins, 10 mins, 20 mins).

Figure S3: Time Course of Model Conditions. All entries n = 1.

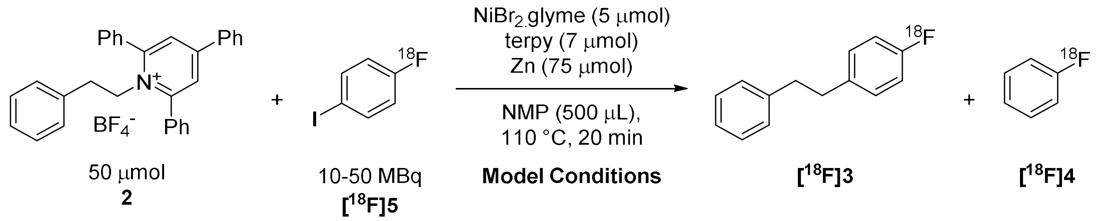

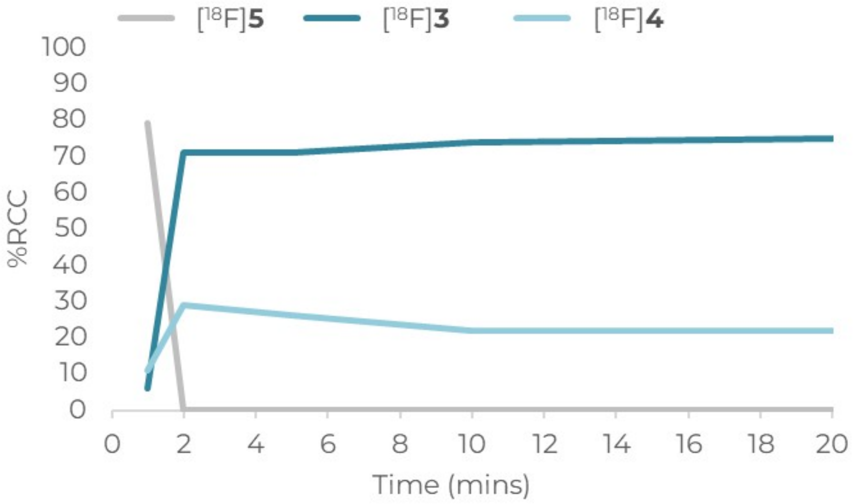

1.7 Investigation of Alternative Alkyl Coupling Partners and Reduction Methods for Cross-Coupling with ^{18}F-Fluoroaryl Halides

1.7.1 General Set-up for Photochemical Reactions

Photochemical reactions were performed in 4 mL glass vials in a HepatoChem EvoluChem PhotoRedOx Box™ using an EvoluChem 18 W blue LED lamp (λ = 450 nm) with fan cooling unless otherwise specified.

1.7.2 Investigation of Photo-mediated Methods for Radiochemical Deaminative Cross-couplings

Table S18: Metallophotoredox Cross-coupling of Alkyl 2,4,6-Triphenylpyridinium salts with [^{18}F]1. All entries n = 1.

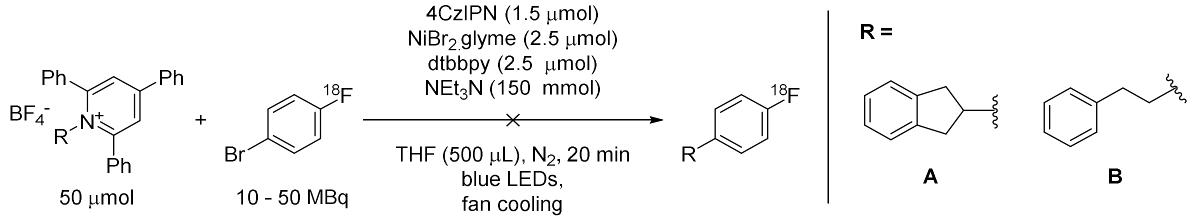

Entry	Substrate	RCC % of Respective Product
**1**	**A**	0
**2**	**B**	0

Conditions are adapted from the analogous non-radiochemical reaction.[22]

2,4,6-Triphenyl pyridinium tetrafluoroborate salt (0.05 mmol), 4CzIPN (1.2 mg, 1.5 μmol), $NiBr_2$·glyme (1 mg, 2.5 μmol) and dtbppy (0.7 mg, 2.5 μmol) were added to an oven dried 4-mL glass vial with stirrer bar and sealed with a PTFE cap lined with teflon septum. To the mixture was added anhydrous THF (500 μL), followed by anhydrous Et_3N (150 μmol, 15 μL). **[^{18}F]1** (10-50 MBq) in THF (approx. 50 μL) was dispensed into the reaction mixture and the vial was degassed by bubbling N_2 through the reaction mixture for 10 seconds. The vial was subjected to irradiation by blue light with fan cooling for 20 minutes. After this time, the reaction mixture was diluted with MeCN (500 μL) and an aliquot was analysed by RadioHPLC.

Table S19: Photoactive Electron Donor-Acceptor (EDA) Complex Mediated Cross-coupling of Alkyl 2,4,6-Triphenylpyridinium salts with [^{18}F]5. All entries n = 1.

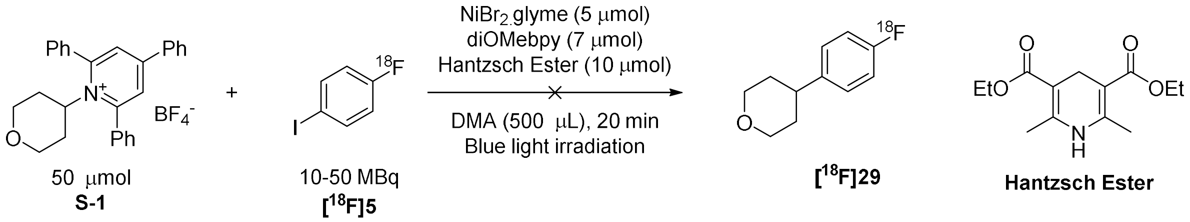

Entry	%RCC [^{18}F]29
**1**	0

Conditions are adapted from the analogous non-radiochemical reaction.[23]

2,4,6-Triphenyl-1-(tetrahydro-*2H*-pyran-4-yl)pyridin-1-ium tetrafluoroborate (**S-1**, 50 µmol), diethyl 1,4-dihydro-2,6-dimethyl-3,5-pyridinedicarboxylate (Hantzsch Ester, 25 mg, 10 µmol), $NiBr_2$·glyme (1.6 mg, 5 µmol) and diOMebpy (1.5 mg, 7 µmol) were added to an oven dried 4-mL glass vial with stirrer bar and sealed with a PTFE cap lined with teflon septum. To the mixture was added anhydrous DMA (500 µL). **[^{18}F]5** (10-50 MBq) in NMP (approx. 50 µL) was dispensed into the reaction mixture and the vial degassed by bubbling N_2 through the reaction mixture for 10 seconds. The vial was subjected to blue light with fan cooling for 20 minutes. After this time, the reaction mixture was diluted with MeCN (500 µL) and an aliquot was analysed by RadioHPLC.

1.7.3 Radiochemical Cross-Electrophile Couplings with [^{18}F]5

Reactions were set-up as described in Section 1.5.

Table S20: Cross-Electrophile Coupling of Primary and Secondary Alkyl Halides and Tosylates with **[^{18}F]5**. All entries n = 2 unless specified.

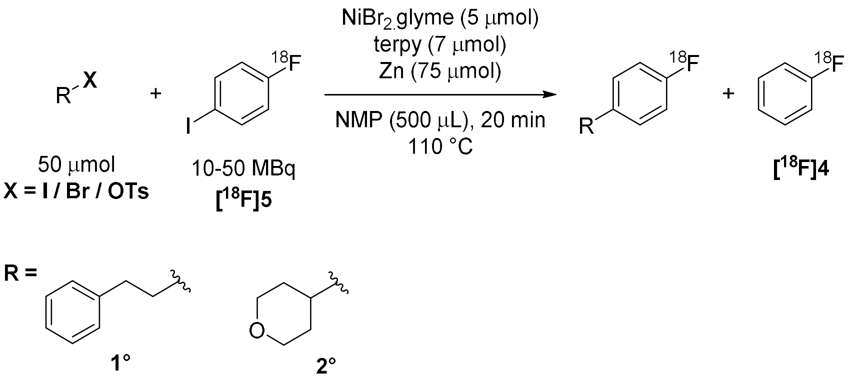

Entry	Substrate	Leaving Group	Deviation from above	%RCC of Product	%RCC [^{18}F]4	%RCC [^{18}F]5
**1** [n = 3]	1°	Br	-	22 ± 14	34 ± 6	28 ± 28
**2** [n = 3]	1°	I	-	26 ± 7	37 ± 12	26 ± 20
**3** [n = 1]	1°	OTs	-	21	24	0
**4**	2°	Br	-	34 ± 22	42 ± 4	25 ± 25
**5**	2°	I	-	17 ± 2	40 ± 1	30 ± 12
**6**	1°	I	PyBCAM as ligand	54 ± 18	12 ± 3	-
**7**	2°	I	PyBCAM as ligand	56 ± 1	22 ± 1	-

1.8 Unsuccessful Alkyl Pyridinium Coupling Partners

Figure S4: Alkyl 2,4,6-triphenylpyridinium coupling partners that led to no desired product under the model reaction conditions (Section 1.5) with **[^{18}F]5**.

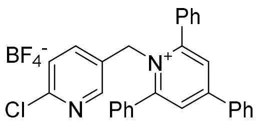

S-2

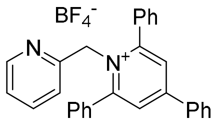

S-3

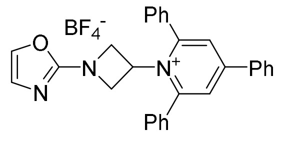

S-4

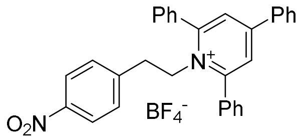

S-5

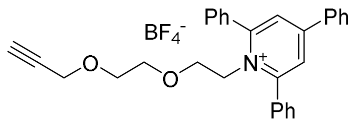

S-6

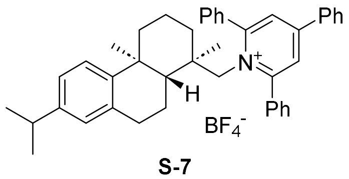

S-7

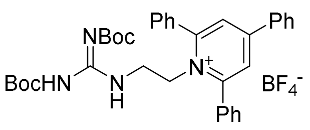

S-8

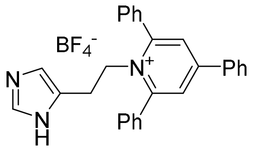

S-9

Benzylic and 3-azetidine *N*-substituents (**S-2**, **S-3**, **S-4**) on the 2,4,6-triphenylpyridinum salt were unsuccessful in the model radiochemical cross-coupling.

Nitro and free alkyne substituents (**S-5**, **S-6**) were also unsuccessful in the radiochemical cross-coupling, which is in-line with known incompatibilities of nickel catalysed cross-electrophile couplings.[27]

Sterically encumbered leelamine derivative (**S-7**) led to no observable product under the model reaction conditions alongside basic motifs such as *N,N'*-dibocguanidine (**S-8**) and free imidazole (**S-9**) substrates.

1.9 Radiosynthesis of ^{18}F-Radiolabelled Aryl Halide Coupling Partners

1.9.1 General Method for Radiofluorination Screening Reactions with [^{18}F]KF

[^{18}F]KF elution and drying was performed using a Trasis AllinOne automated synthesizer. [^{18}F]Fluoride was separated from ^{18}O-enriched-water using an anion exchange cartridge (Waters Sep-Pak AccellPlus QMA Carbonate Plus Light Cartridge, activated with H_2O (10.0 mL) prior to use) and released with a solution of K_{222} (7.5 mg) and K_2CO_3 (1.5 mg) in MeCN/H_2O (0.75 mL, 4:1, v/v). The solution was dried over a period of 20 min by azeotropic drying using MeCN (3 x 700 µL) under a flow of N_2 at 110 °C.

To an oven-dried 3 mL glass V-vial equipped with a magnetic stirrer bar was added the radiolabelling precursor, full-batch conditions (elution salts as additives), and solvent. The vial was sealed with a septum and [^{18}F]KF.K_{222} solution (5-20 MBq) in MeCN (approx. 20 µL). The reaction was heated to the temperature specified for 20 minutes. The reaction mixture was cooled to room temperature and subsequently diluted with MeCN/H_2O (200 µL, 3:1 v/v). An aliquot of this mixture was subsequently analysed by RadioHPLC (HPLC Condition A, Section 1.2).

Alternative experimental methods were used as specified.

1.9.2 ^{18}F-Radiolabelled Aryl Halide Coupling Partners: Radiolabelling Validation and Optimization

Table S21: Small scale radiosynthesis of **[^{18}F]1** and **[^{18}F]5** from Boronic Pinacol Esters. All entries n = 1. Full batch conditions: K_2(oxalate) (1 mg), K_{222} (6.3 mg), K_2CO_3 (0.1 mg).

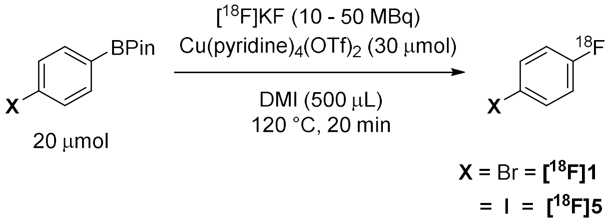

Entry	Product	%RCC of Product
**1**	**[^{18}F]1**	55
**2**	**[^{18}F]5**	53

Table S22: Small-scale radiosynthesis of **[18F]35**. All entries n = 1. Full batch conditions: K_{222} (7.5 mg), K_2CO_3 (1.5 mg).

Radiolabelling method adapted from previously published conditions.[46] TBAB = Tetrabutylammonium bromide.

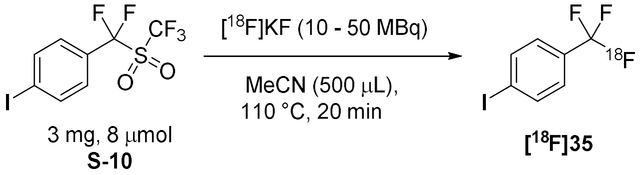

Entry	Deviation from above	%RCC of [18F]35
**1**	None	31
**2**	2.5 mg TBAB	25

Scheme S2: Small scale radiosynthesis of **[18F]37**. n = 1. Full batch conditions: K_2(oxalate) (1 mg), K_{222} (6.3 mg), K_2CO_3 (0.1 mg).

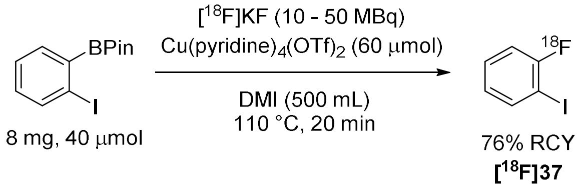

Scheme S3: Small scale radiosynthesis of **[18F]36**. n = 1. Full batch conditions: K_{222} (7.5 mg), K_2CO_3 (1.5 mg).

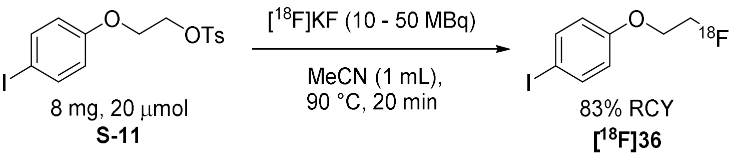

Table S23: Small scale radiosynthesis of **[^{18}F]34**. All entries n = 1. Full batch conditions: K_{222} (7.5 mg), K_2CO_3 (1.5 mg).

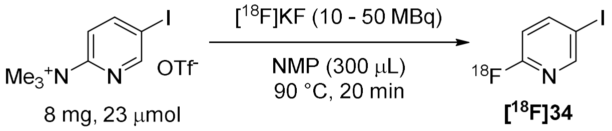

Entry	Deviation from above	%RCC of [^{18}F]34
**1**	None	93
**2**	DMI	89
**3**	DMA	33

Table S24: Radiosynthesis of **[^{18}F]5** from (4-iodophenyl)diphenylsulfonium triflate. All entries are n = 1. Full batch conditions, additives specified for each entry.

Methods adapted from previously published conditions. [48,49]

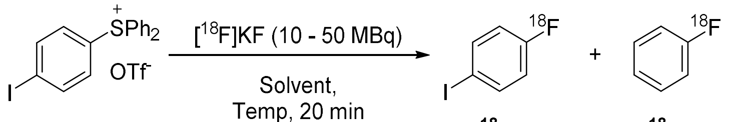

Entry	Method	Substrate Loading (mg)	Solvent (µL)	Temp. (°C)	Additives	%RCC of [^{18}F]5	%RCC of [^{18}F]4
**1**	General	7	DMA (300)	90	K_{222} (15 mg), K_2CO_3 (2.8 mg)	28	20
**2**	General	7	DMI (300)	90	K_{222} (15 mg), K_2CO_3 (2.8 mg)	18	32
**3**	A	7	MeCN (1000)	85	K_{222} (15 mg), K_2CO_3 (2.8 mg)	7	0
**4**	A	7	DMSO (500)	110	K_{222} (11 mg), $KHCO_3$ (3 mg)	30	9
**5**	A	7	NMP (500)	110	K_{222} (11 mg), $KHCO_3$ (3 mg)	29	12
**6**	B	5	DMSO (500)	110	K_{222} (5.6 mg) $KHCO_3$ (1.5 mg)	57	-
**7**	B	5	NMP (500)	110	K_{222} (5.6 mg) $KHCO_3$ (1.5 mg)	30	-

Method A: [^{18}F]KF (10 – 50 MBq) in MeCN (~ 10 µL) was dispensed into a vial containing additives and dried under a flow of N_2 while heating to 90 °C. (4-iodophenyl)diphenylsulfonium triflate (7 mg) was dissolved in the reaction solvent and dispensed into the same vial and heated at the specified temperature for 20 mins. Analysis of the reaction mixture was performed as describe in the general method (Section 1.9.1).

Method B: K_{222} (5.6 mg) and $KHCO_3$ (1.5 mg) in a mixture of 85: 15 MeCN:H_2O (500 µL) were added to the reaction vial. [^{18}F]KF (10 – 50 MBq) in MeCN (~ 10 µL) was dispensed into the same vial and the mixture dried azeotropically by heating to 90 °C under a flow of N_2. MeCN (500 µL) was added to the vial and the same drying process repeated. (4-Iodophenyl)diphenylsulfonium triflate (5 mg) in the reaction solvent was dispensed into the same vial and heated at the specified temperature for 20 mins. Analysis of the reaction mixture followed that of the general method (Section 1.9.1).

Table S24: Radiosynthesis of **[^{18}F]5** from 5-(4-iodophenyl)-5*H*-dibenzo[*b*,*d*]thiophen-5-ium triflate. All entries are n = 1. Full batch conditions, salts specified for each entry.

Methods adapted from previously published conditions.[44,49]

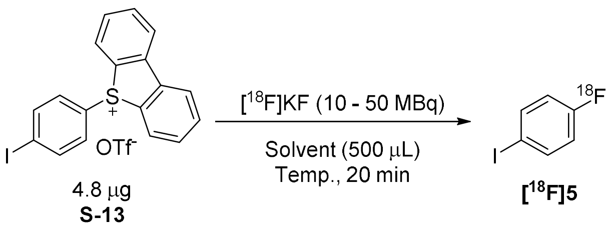

Entry	Method	Solvent (500 µL)	Temp. (°C)	Additives	%RCC of Product
**1**	General	MeCN	90	K_{222} (7.5 mg), K_2CO_3 (1.5 mg)	0
**2**	General	MeCN	110	K_{222} (7.5 mg), K_2CO_3 (1.5 mg)	14
**3**	C	MeCN	110	K_{222} (7.5 mg), K_2CO_3 (1.5 mg)	0
**4**	C	DMSO	110	K_{222} (7.5 mg), K_2CO_3 (1.5 mg)	0
**5**	C	NMP	110	K_{222} (7.5 mg), K_2CO_3 (1.5 mg)	3
**6**	D	MeCN	110	None added	0
**7**	D	NMP	110	None added	0
**8**	D	DMSO	110	None added	50

Radiochemical conversion (%RCC) determined by RadioHPLC. n = 1. Full batch conditions, salts specified for each entry.

Method C: [^{18}F]KF (10 – 50 MBq) in MeCN (~ 10 µL) was dispensed into a vial containing additives and dried under a flow of N_2 while heating to 90 °C. 5-(4-Iodophenyl)-5*H*-dibenzo[*b*,*d*]thiophen-5-ium triflate (**S-13**, 4.8 mg) was dissolved in the reaction solvent and dispensed into the same vial and heated at the specified temperature for 20 mins. Analysis of the reaction mixture followed that of the general method (Section 1.9.1).

Method D: A Chromafix PS-HCO_3 ^{18}F separation cartridge was preconditioned with an aqueous K_2(oxalate) solution (3 mL, 10 mg/ mL) and H_2O (2 mL). The cartridge was subsequently washed with anhydrous MeCN (1 mL) and air (2 mL). **S-13** (4.8 mg) in MeOH

(0.5 mL) was eluted through the cartridge into a 5 mL V-vial followed by MeCN (0.5 mL) and air (1 mL). The eluted substrate was dried in the V-Vial under a flow of N_2 while heating to 90 °C. MeCN (1 mL) and azeotropically dried in the same manner twice. The reaction solvent was added and [^{18}F]KF (10 – 50 MBq) in MeCN (~ 10 μL) dispensed into the reaction mixture, which was heated to the specified temperature for 20 minutes. Analysis of the reaction mixture followed that of the general method (Section 1.9.1).

1.10 Automated Syntheses of ^{18}F-Radiolabelled Aryl Halide Coupling Partners on TRASIS AllinOne

1.10.1 Automated Synthesis of [^{18}F]37

As described for the synthesis of **[^{18}F]5** using 2-(2-iodophenyl)-4,4,5,5-tetramethyl-1,3,2-dioxaborolane as the precursor (Section 1.3).

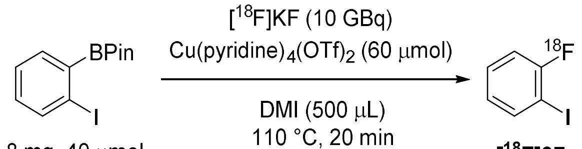

[^{18}F]37

Activity Yield = 1.42 GBq from 10 GBq, 14% (n.d.c).

Semi-preparative purification of [^{18}F]37 – Phenomenex Gemini LC column, 250 x 10 mm. **Eluent** = isocratic, 65:35 MeCN:H_2O. **Flow rate** = 4.0 mL/min. **Temperature** = 25 °C. **Wavelength** = 254 nm. $\boldsymbol{t_R}$ = 9.5 – 12 mins.

1.10.2 Automated Synthesis of [^{18}F]38

As described in for the synthesis of **[^{18}F]5** from 2-(3-chloro-5-iodophenyl)-4,4,5,5-tetramethyl-1,3,2-dioxaborolane (**S-53**) as the precursor (Section 1.3).

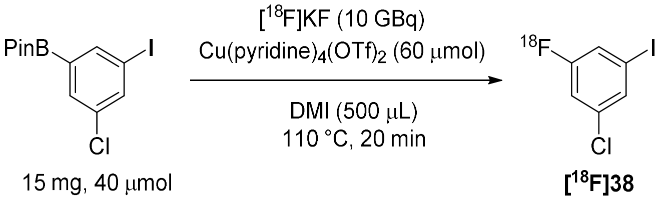

[^{18}F]38

Activity Yield = 300 MBq from 10 GBq, 3% (n.d.c).

Semi-preparative purification of [^{18}F]37 – Phenomenex Gemini LC column, 250 x 10 mm. **Eluent** = isocratic, 65:35 MeCN:H_2O. **Flow rate** = 4.0 mL/min. **Temperature** = 25 °C. **Wavelength** = 254 nm. $\boldsymbol{t_R}$ = 13 – 16 mins.

1.10.3 General Automated Synthesis of ^{18}F-Fluorinated Aryl Iodides

The following ^{18}F-fluorinated aryl iodides were produced *via* the same automated synthesis program on a TrasisAllinOne synthesizer utilizing a prebuilt cassette (Figure 5): **[^{18}F]34**, **[^{18}F]35**, and **[^{18}F]36**.

Cassette Preparation:

Position 2: Vial containing K_2CO_3 (1.5 mg), K_{222} (7.5 mg) in H_2O (110 µL) and MeCN (500 µL)

Position 3: Waters Sep-Pak AccellPlus QMA Carbonate Plus Light Cartridge preconditioned with H_2O (10 mL)

Position 8: MeCN (10 mL) solvent reservoir for azeotropic drying of [^{18}F]fluoride

Position 9: Radiolabelling precursor in reaction solvent – amounts specified for each precursor below.

Position 17: Anhydrous NMP (10 mL) solvent reservoir for elution of ^{18}F-fluoro(hetero)aryl iodide post-HPLC

Position 33: Waters Sep-Pak C18 Plus cartridge preconditioned with EtOH (10 mL) and then H_2O (10 mL)

Position 34: H_2O (25 mL) reservoir for reaction dilution prior to HPLC loading

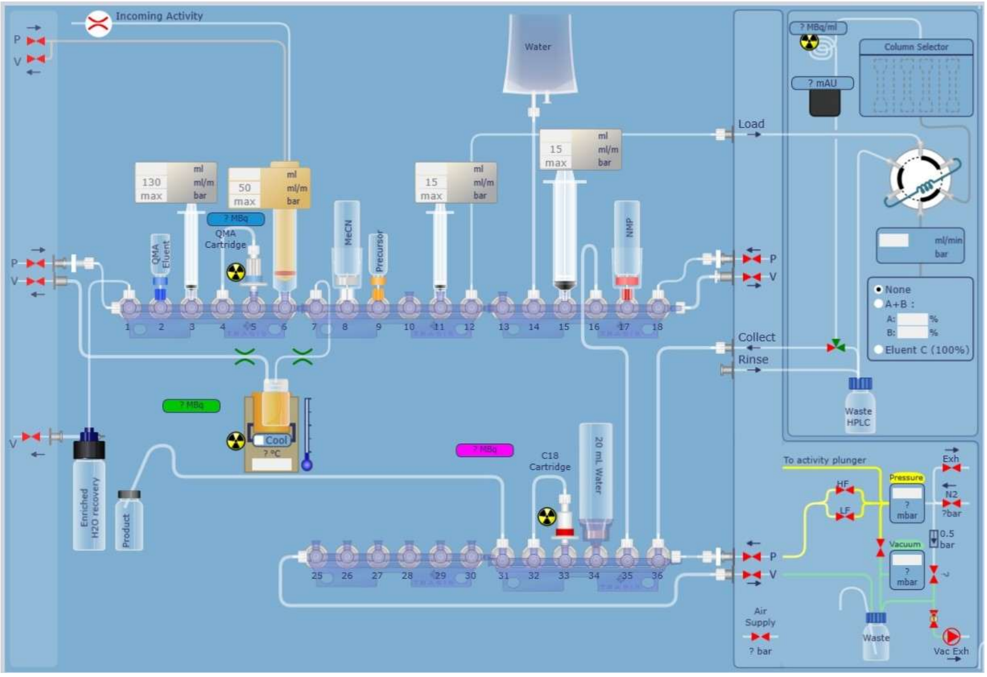

Figure S5: Cassette layout used for the automated syntheses of **[^{18}F]34**, **[^{18}F]35**, and **[^{18}F]36** on the Trasis AllinOne Platform. The figure was generated using the Trasis software.

Method:

Fluorination Reaction: [^{18}F]Fluoride in [^{18}O]H_2O was received from the cyclotron and subsequently trapped on a QMA cartridge (position 3). The elution salts (position 2) were passed through the QMA cartridge eluting [^{18}F]fluoride which was dried azeotropically in the reaction vial by heating to 120 °C with MeCN under a flow of N_2. The radiolabelling precursor (position 9) was dispensed in the reaction vial and heated to the specified temperature for 20 minutes.

Purification and Reformulation: Dilution of the reaction mixture with H_2O was done prior to loading of the HPLC sample loop. Purification of the crude reaction mixture was achieved by semi-preparative reverse phase HPLC. A 1:1 mixture of the isolated product eluate in H_2O was loaded onto a C18 cartridge (position 33). The C18 cartridge was subsequently washed with H_2O (10 mL) followed by an air purge. The ^{18}F-radiolabelled product was eluted from the C18 cartridge with NMP (2 mL) into a vial and the activity yield of the collected product measured. The solution containing the ^{18}F-radiolabelled product was used as a stock solution for performing screening reactions.

1.10.3A Automated synthesis of [^{18}F]34

Automated synthesis was performed as described in Section 1.10.2 from 5-iodo-*N,N,N*-trimethylpyridin-2-aminium (**S-12**, 8.2 mg, 23 μmol).

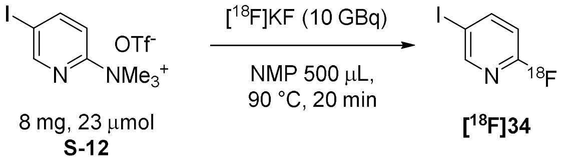

Activity Yield: 1.09 GBq from 10 GBq, 11%, (n.d.c).

Semi-preparative purification of [^{18}F]34 – Phenomenex Gemini LC column, 250 x 10 mm. **Eluent** = isocratic, 50:50 MeCN:H_2O. **Flow rate** = 4.0 mL/min. **Temperature** = 25 °C. **Wavelength** = 254 nm. $\boldsymbol{t_R}$ = 9.5 – 13 min.

1.10.3B Automated synthesis of [^{18}F]35

Automated synthesis was performed as described in Section 1.10.2 from 1-(difluoro((trifluoromethyl)sulfonyl)methyl)-4-iodobenzene (**S-10**, 3 mg, 8 μmol).

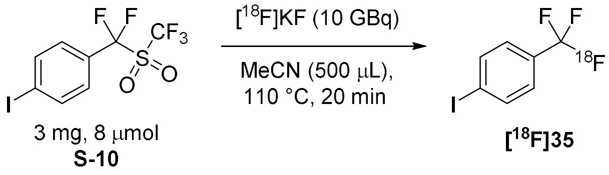

Activity Yield: 67 MBq from 10 GBq, 1%, (n.d.c).

Semi-preparative purification of [^{18}F]35 – Phenomenex Gemini LC column, 250 x 10 mm. **Eluent** = isocratic, 70:30 MeCN:H_2O. **Flow rate** = 4.0 mL/min. **Temperature** = 25 °C. **Wavelength** = 254 nm. $\boldsymbol{t_R}$ = 11.5 – 12.5 min.

1.10.3C Automated synthesis of [^{18}F]36

Automated synthesis was performed as described in Section 1.10.2 from 2-(4-iodophenoxy)ethyl 4-methylbenzenesulfonate (**S-11**, 8.4 mg, 20 µmol)

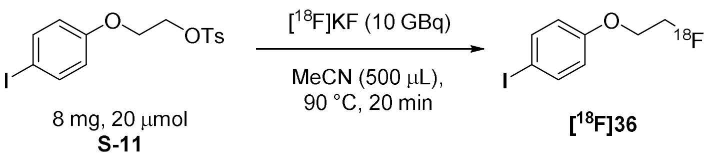

Activity Yield: 3.49 GBq from 10 GBq, 35%, (n.d.c).

Semi-preparative purification of [^{18}F]36 – Phenomenex Gemini LC column, 250 x 10 mm. **Eluent** = isocratic, 60:40 MeCN:H_2O. **Flow rate** = 4.0 mL/min. **Temperature** = 25 °C. **Wavelength** = 254 nm. $\boldsymbol{t_R}$ = 14 – 16 min.

1.10.3 One-Pot Radiolabelling/ Cross-Coupling Protocols

One-pot radiolabelling/cross-coupling protocols using aryl BPin, heteroaryl trimethylammonium, or aryl sulfonium precursors were trialled. Although the radiolabelling proceeded, cross-coupling in the presence of unreacted radiolabelling precursor was not successful.

Radiolabelling procedures were performed with reduced solvent (300 μL) and the crude reaction mixture added to a secondary vial containing the cross-coupling components in 200 μL of reaction solvent such that the total reaction volume was 500 μL.

Aliquots of the crude radiolabelling reaction mixture were taken to evaluate the %RCC for this step.

The general radiolabelling method (Section 1.9.1) was used for all precursors.

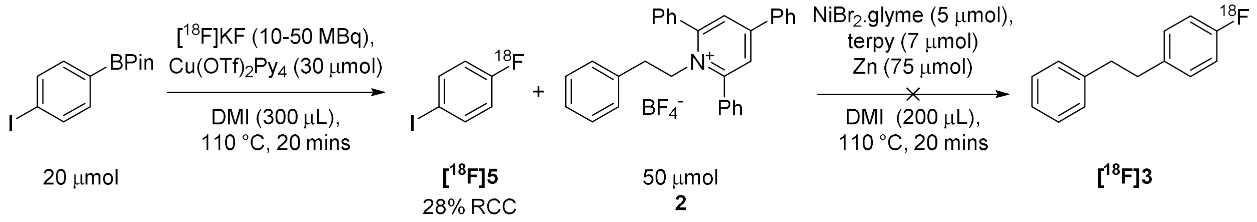

Full batch conditions, the following salts were added to the radiofluorination step:
$K_2(Ox)$ (1 mg), K_{222} (6.3 mg), K_2CO_3 (0.1 mg)

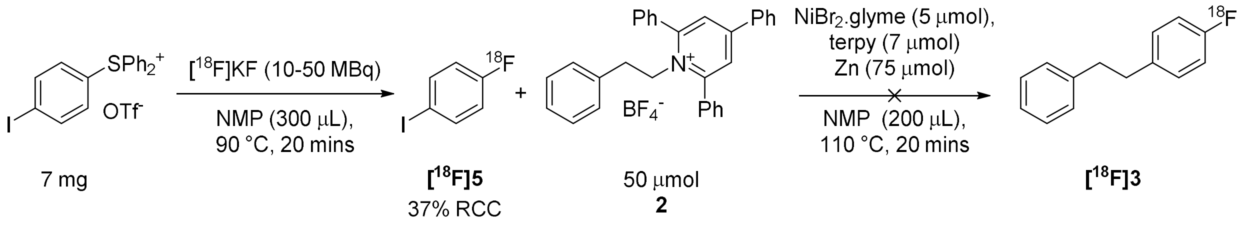

Full batch conditions, the following salts were added to the radiofluorination step:
K_{222} (15 mg), K_2CO_3 (2.8 mg)

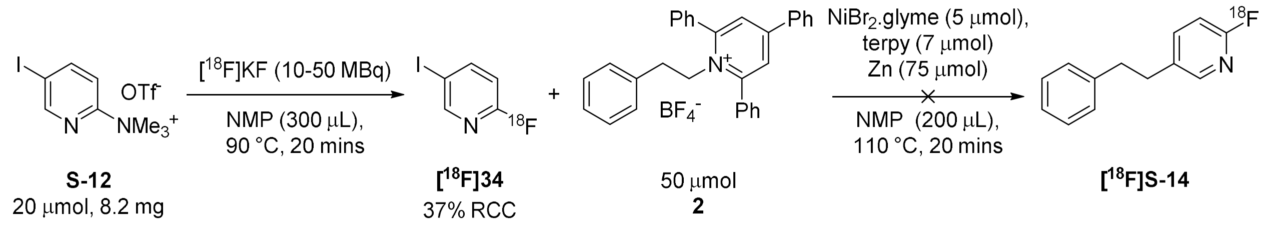

Full batch conditions, the following salts were added to the radiofluorination step:
K_{222} (7.5 mg), K_2CO_3 (1.5 mg)

1.10.4 Cross-Coupling Optimization with ¹⁸F-Fluoro(Hetero)aryl Iodide Coupling Partners

Table S25: Cross-coupling optimization of varied ^{18}F-fluoro(hetero)aryl iodide coupling partners with model substrate (**2**). Entries are %RCC of the corresponding cross-coupling product (Scheme S4) and are *n*= 2 unless indicated. * *n* = 3. ♦ *n* = 1. # *n* = 8. Highest yielding condition is highlighted in blue.

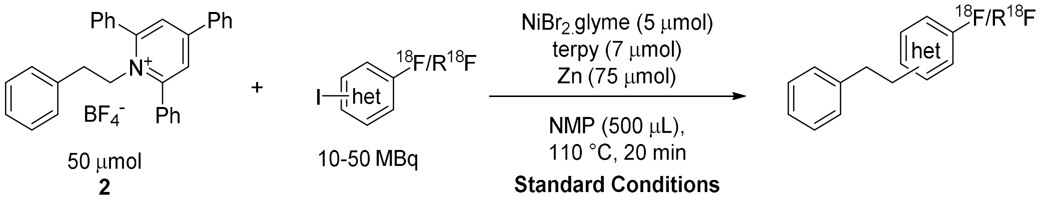

	Deviation from Standard Conditions								
	Temperature (°C)						Zinc Loading		Ligand
**¹⁸F-Fluoro(Hetero)aryl Iodide**	**RT**	**50**	**65**	**80**	**95**	**110**	**15 µmol**	**45 µmol**	**PyBCAM**
**[¹⁸F]5**	4♦	13	-	64	-	72#	31	35	87
**[¹⁸F]34**	-	-	19	22	26	33*	22	29	-
**[¹⁸F]35**	-	-	26	50	56	54*	-	-	50
**[¹⁸F]36**	-	-	27	63	69	70*	-	-	34
**[¹⁸F]37**	-	-	-	45	42	43*	-	-	-

Scheme S4: Structures ^{18}F-fluoro(hetero)aryl iodide coupling partners **[¹⁸F]5**, **[¹⁸F]34**, **[¹⁸F]35**, **[¹⁸F]36**, and [¹⁸F]37 and respective products for **Table 25**.

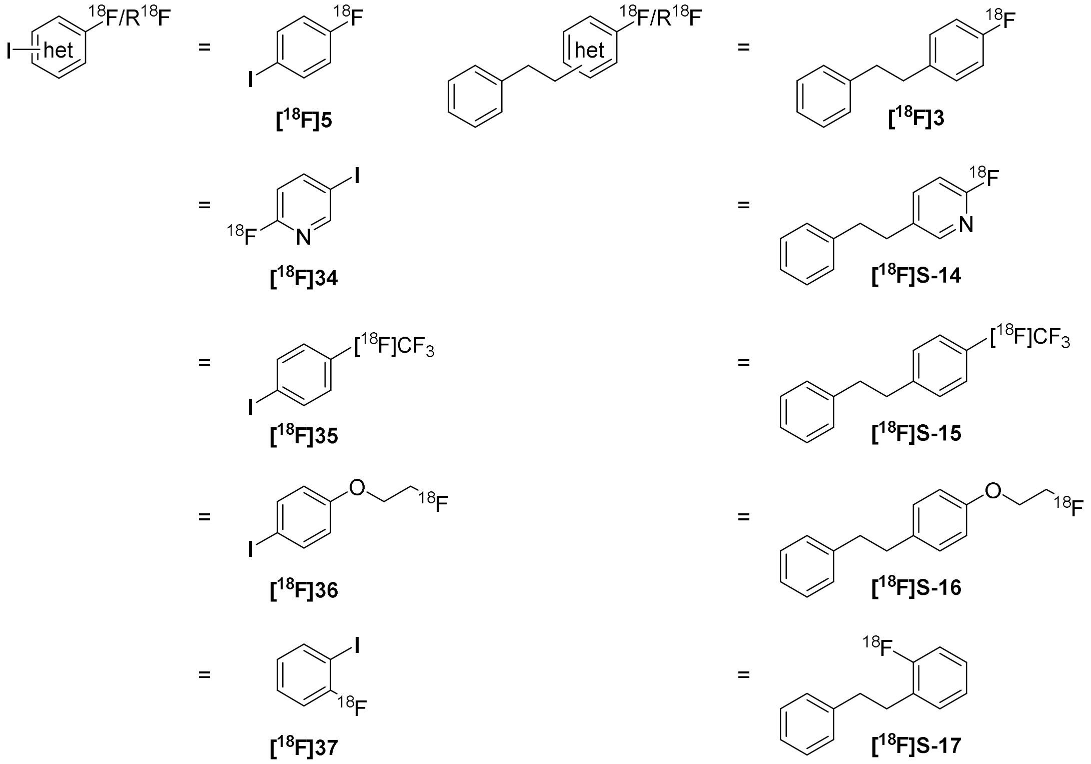

Temperature had a significant effect on the %RCC of the desired cross-coupled product. However, **Table 25** putatively indicates different reagents have different "onset" temperatures, whereby increasing the temperature beyond this point offers little to no improvement in the respective %RCC. For example, the highest %RCC of the corresponding cross-coupled products for **[^{18}F]37**, **[^{18}F]35**, and **[^{18}F]36** were achieved at 80 °C, 95 °C, and 110 °C respectively. Therefore, performing the cross-coupling of all new ^{18}F-labelled fluoro(hetero)aryl iodides would be best attempted at 110 °C, however, lower temperatures may be viable for alternative ^{18}F-labelled fluoro(hetero)aryl iodides.

Reduction of zinc loading offered no improvement/was detrimental to the %RCC of corresponding products in the cross-couplings of **[^{18}F]5** and **[^{18}F]34**. Thus, it was deemed an unviable parameter to optimize when attempting additional ^{18}F-labelled fluoro(hetero)aryl iodides in the developed cross-coupling.

PyBCAM as a ligand was only beneficial to the %RCC of the cross-coupling of **[^{18}F]5**. When used as the ligand for reagents **[^{18}F]35** and **[^{18}F]36**, PyBCAM offered no improvement or was detrimental to the %RCC.

From this data we concluded the ligand would serve as the most important factor to investigate when applying the method to additional ^{18}F-labelled fluoro(hetero)aryl iodides.

See **Section 3.3.4 for** radiochemical ligand screening of **33** with **[^{18}F]5** and **[^{18}F]37** .

1.11 TRASIS AllinOne Semi-Automated Synthesis of [18F]41

1.11.1 TRASIS AllinOne Method and HPLC Conditions

The semi-automated radiosynthesis of [18F]-5-(3-chloro-4-methoxyphenyl)-*N*-(3-(4-fluorophenyl)propyl)oxazole-4-carboxamide (**[18F]41,** Scheme S5) was performed on a TRASIS AllInOne synthesizer using a pre-built cassette.

Scheme S5: General scheme for TRASIS AllinOne automated synthesis of **[18F]41** from cartridge purification and subsequent cross-coupling of **[18F]5**.

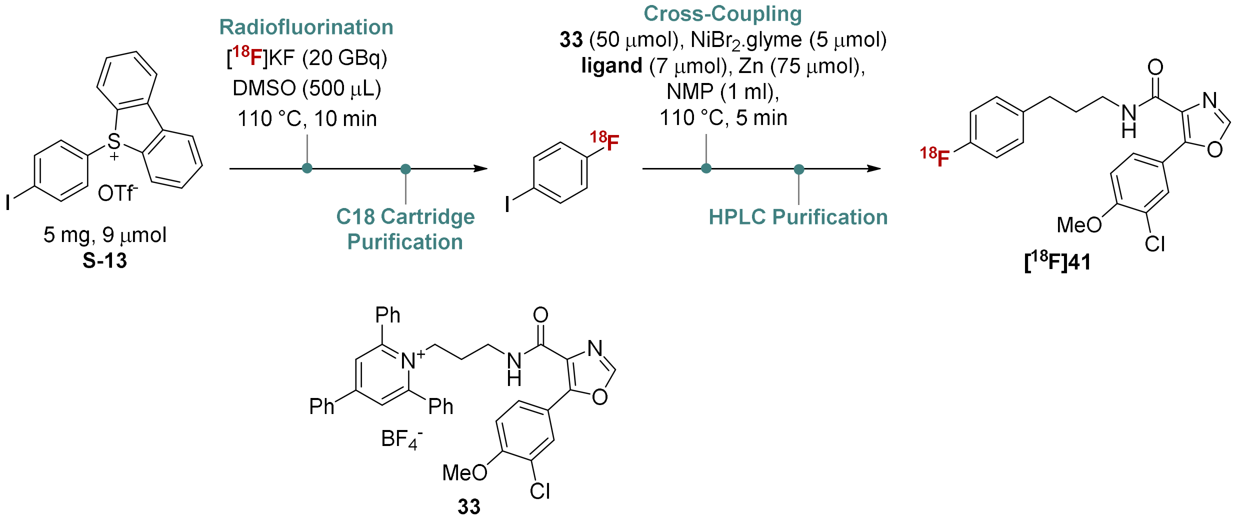

Semi-preparative purification of [18F]44 – Phenomenex Gemini LC column, 250 x 10 mm. **Eluent** = isocratic 65:35 MeCN:H_2O. **Flow rate** = 4.0 mL/min. **Temperature** = 25 °C. **Wavelength** = 254 nm. $\boldsymbol{t_R}$ = 20 min

Cassette and Vial Preparation:

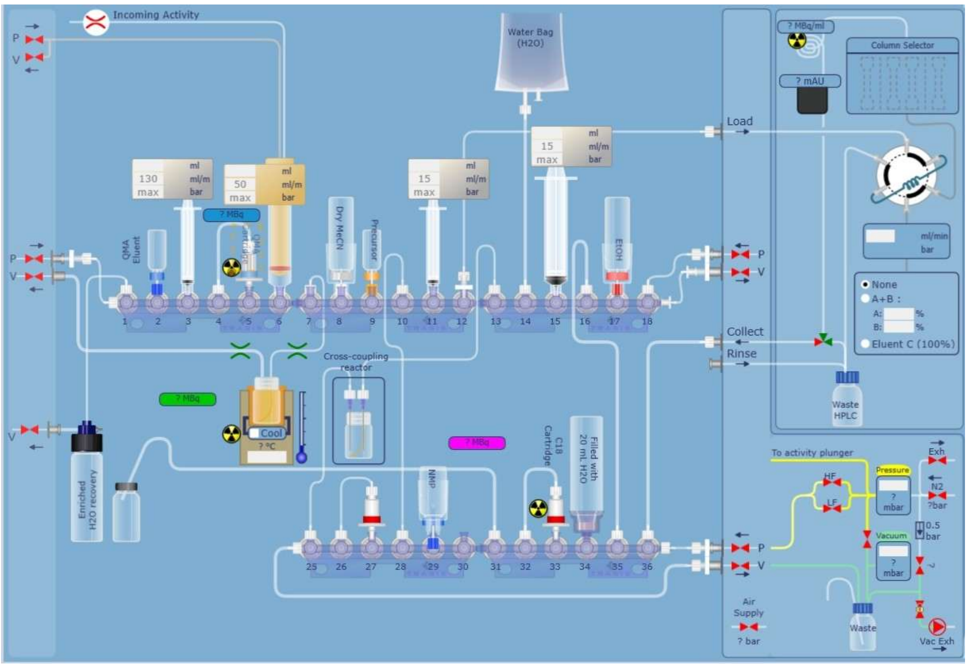

Position 2: Vial containing $KHCO_3$ (1.5 mg), K_{222} (5.6 mg), in MeCN:H_2O (500 µL, 85:15 v/v).

Position 3: Waters Sep-Pak AccellPlus QMA Carbonate Plus Light Cartridge [WAT023525] preconditioned as follows in the order as written: NaOH (aq, 1 M, 5 mL), H_2O (10 mL), K_2CO_3 (aq., 1 M, 1 mL), H_2O (10 mL), air (10 mL).

Position 8: MeCN (10 mL) solvent reservoir for azeotropic drying of [^{18}F]fluoride.

Position 9: 5-(4-iodophenyl)-5H-dibenzo[*b,d*]thiophen-5-ium triflate (**S-13**, 5 mg, 9 µmol) in anhydrous DMSO (500 µL).

Position 12: Filter placed on cassette. Hydrophilic syringe filter is used to prevent overpressure on the cassette. [Whatman UniFLO 25 mm, 0.2 µm PES filter media, catalogue # 9914-2502].

Position 13: This line has a needle fitted to the end and is **only placed into the cross-coupling reactor once the cross-coupling reaction has taken place.**

Position 17: MeCN (10 mL) solvent reservoir for elution of **[^{18}F]41**.

Position 25: This line has a needle fitted to the end and is placed into the cross-coupling reactor prior to the automation taking place.

Position 27: Waters Sep-Pak C18 Plus (Waters SKU WAT020515) cartridge preconditioned with EtOH (10 mL) and then H_2O (10 mL).

Position 29: NMP (10 mL) solvent reservoir for cartridge elution of **[^{18}F]5**.

Position 33: Waters Sep-Pak C18 Plus (Waters SKU WAT020515) cartridge preconditioned with EtOH (10 mL) and then H_2O (10 mL).

Position 34: H_2O (25 mL) reservoir for dilution of collected HPLC eluate before cartridge purification and reformulation.

Additional Notes about Set-up:

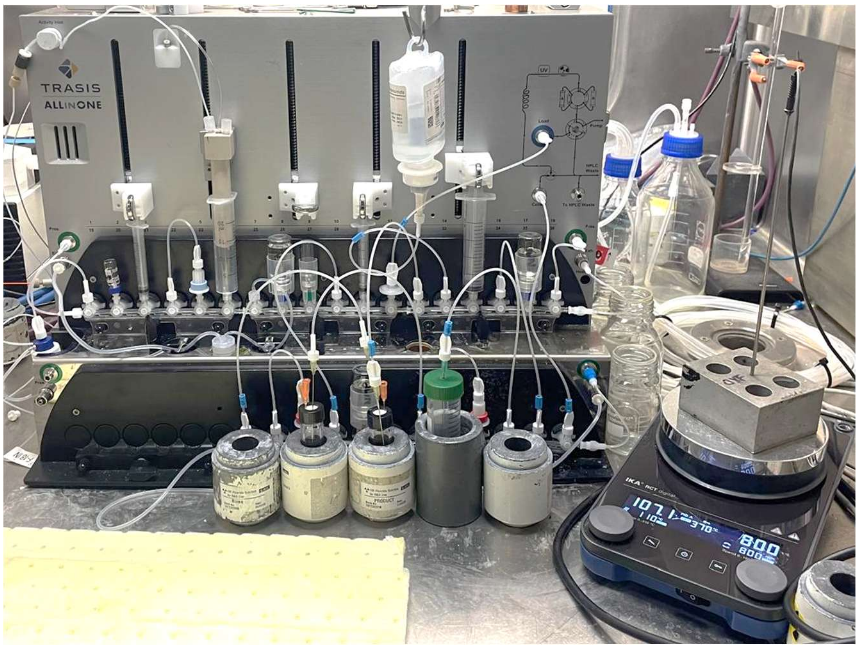

A secondary reactor (5 mL V-vial) was used for cross-coupling reaction (see lines at positions 13 and 25) was charged with the following: stirrer bar, 1-(3-(5-(3-chloro-4-methoxyphenyl)oxazole-4-carboxamido)propyl)-2,4,6-triphenylpyridin-1-ium tetrafluoroborate (**33**, 50 μmol, 34 mg), Zn (75 μmol, 5 mg), $NiBr_2$.glyme (5 μmol, 1.5 mg), Ligand (7 μmol, 1.7 mg) and fitted with a vent needle. A hot-plate was set to 110 °C and 800 rpm prior to the automated process starting and placed so the cross-coupling reactor can be moved with ease between the lead pot in front of the synthesizer to the heating block on the hot-plate. An additional V-vial was placed in a lead pot to be used for storing the needles connected to the lines as well as additional vent needles.

Method:

Fluorination Reaction: [^{18}F]Fluoride in [^{18}O]H_2O was received from the cyclotron and subsequently trapped on a QMA cartridge (position 3). The elution solution (position 2) was passed through the QMA cartridge eluting [^{18}F]fluoride, which was dried azeotropically in the reaction vial by heating to 90 °C with MeCN under a flow of N_2. **S-13** (5 mg, 9 μmol) in anhydrous DMSO (500 μL) (position 9) was dispensed in the reaction vial and heated to 110 °C for 10 minutes.

[^{18}F]5 C18 purification and cross-coupling: Dilution of the radiofluorination reaction mixture with H_2O was performed prior to C18 cartridge (position 27) loading. The C18 cartridge was then washed with NMP (0.3 mL), which was not collected, and subsequently purged with air. Additional NMP (1 mL) was washed through the same C18 cartridge, which was eluted directly into the cross-coupling reactor *via* line 25. Line 25 and vent needle were removed manually from the cross-coupling reactor and the vial was moved onto the hot plate. The reaction was heated at 110 °C and stirred for 5 minutes, after which it was removed to a lead pot to cool for 5 minutes.

[^{18}F]41 Purification and Reformulation: Line 13 was placed into the cross-coupling reactor along with a vent needle. The reaction mixture was diluted with H_2O and transferred to the HPLC sample loop. Purification of the crude reaction mixture was achieved by semi-preparative reverse-phase HPLC (**Section 1.11.1**). The collected eluate was mixed with an equal volume of H_2O in the 20 mL syringe and loaded onto a C18 cartridge (position 33) in three cycles. The C18 cartridge was subsequently washed with H_2O (10 mL) and purged with air. [^{18}F]**41** was eluted from the C18 cartridge with MeCN (2 mL) into a vial and the activity of collected [^{18}F]**41** was measured in a dose calibrator.

1.11.2 TRASIS AllinOne Run 1: Synthesis of [^{18}F]41 using PyBCAM as Ligand

The semi-automated radiosynthesis of (**[^{18}F]41**, Scheme S6) was performed on a TRASIS AllinOne synthesizer using a prebuilt cassette.

Method as described in **Section 1.11.1** using PyBCAM as ligand (7 µmol, 1.7 mg).

Scheme S6: TRASIS AllinOne automated synthesis of **[^{18}F]41** from cartridge purification and subsequent cross-coupling of **[^{18}F]5** using PyBCAM as ligand.

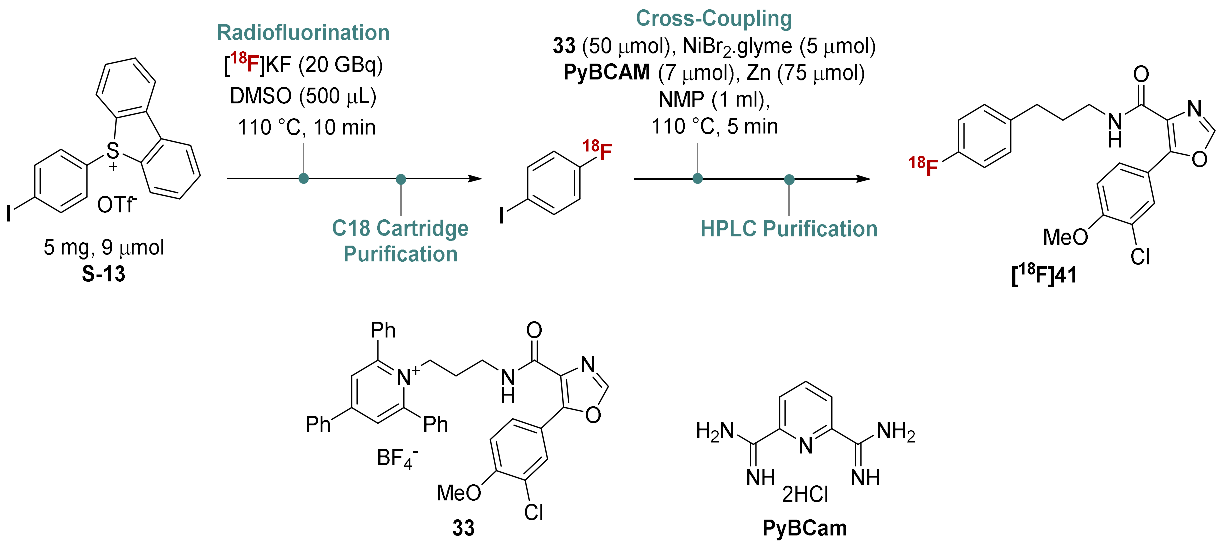

Activity yield: 1.82 GBq from 20 GBq, 9% n.d.c.

Synthesis time: 99 minutes

Radiochemical Purity (RCP): > 99%

1.11.3 TRASIS AllinOne Run 1: Semi-Preparative HPLC Trace for Purification of [^{18}F]5-(3-chloro-4-methoxyphenyl)-*N*-(3-(4-fluorophenyl)propyl)oxazole-4-carboxamide

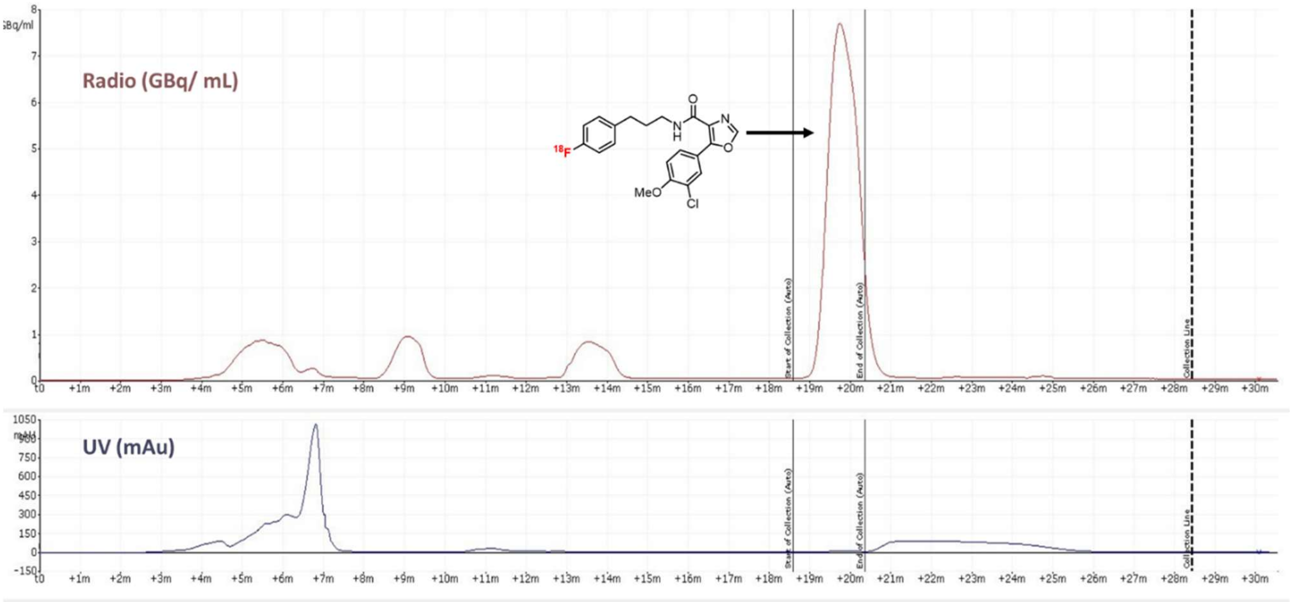

1.11.4 TRASIS AllinOne Run 1: Quality Control of Purified [^{18}F]41 Analytical HPLC Trace

Analytical HPLC Condition A

See **Section 1.15** for overlay of crude screening scale reaction with non-radioactive reference compound, **41**.

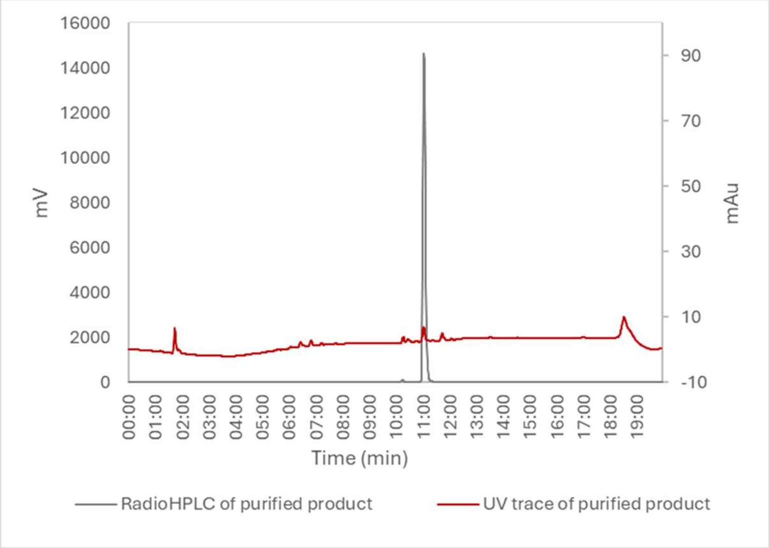

1.11.5 TRASIS AllinOne Run 1: Molar Activity Calculation of Purified [^{18}F]41

Molar activity (A_M) calculation: Molar activity was determined on a radiochemically and chemically pure sample of HPLC-purified **[^{18}F]41**. An aliquot (ca. 20 MBq) of **[^{18}F]41** was transferred to an HPLC vial and diluted with MeCN (approx. 0.5 mL). This sample was then analysed in triplicate by analytical HPLC. The time of injection and activity before and after injection on the HPLC were recorded to determine the injected activity.

Determination of the amount of non-radioactive material was achieved by integration of the UV-HPLC peak corresponding to **41**. A calibration curve of non-radioactive sample was prepared to quantify the amount of non-radioactive **41** present in the sample. Molar activity was then calculated.

Calibration curve: 5-(3-chloro-4-methoxyphenyl)-*N*-(3-(4-fluorophenyl)propyl)oxazole-4-carboxamide (**41**, 2 mg, 5 µmol) was dissolved in MeCN (1 mL). 8 solutions were prepared in a 1:5 serial dilution. These were analysed by HPLC [10 µL injections from the stock solutions, (HPLC conditions = Condition A) and the integrals of the peaks].

Figure S5: HPLC calibration curve for calculating molarity of **[^{18}F]41.**

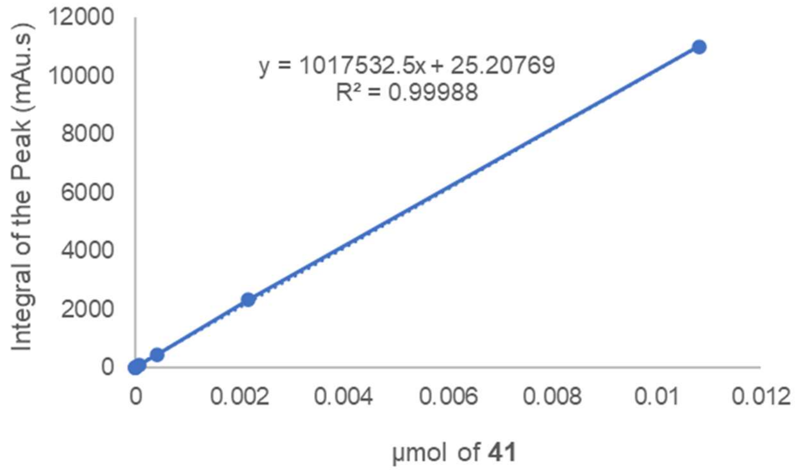

Table S26: Molar Activity Calculation of **[^{18}F]41**. EOS = End of synthesis. d.c. = decay corrected. n.d.c. = non-decay corrected

Measurement	Time after EOS (mins)	Activity Injected (MBq, n.d.c.)	Peak Area (mAu*s)	µmol of 41 injected	A_M (GBq/ µmol, d.c. EOS)
**1**	24	0.4	19.0	6.1×10^{-6}	77.0
**2**	47	0.41	18.2	6.9×10^{-6}	79.9
**3**	70	0.27	21.4	3.7×10^{-6}	112.2
				Average A_M:	90 ± 16

1.11.6 TRASIS AllinOne Run 1: Quality Control of Purified [^{18}F]41 – Residual Nickel

HPLC-purified **[^{18}F]41** was subjected to semi-quantitative analysis of residual nickel present once the sample had decayed fully.

Quantofix® Semi-quantitative test strips were used for the determination of the concentration of residual nickel (Gradation 0-10-25-50-100-250-500-1000 Ni^{2+}); no colour change was detected, which corresponds to a residual Ni^{2+} concentration of less than 10 mg/L (i.e. <20 µg in the 2 mL sample of decayed **[^{18}F]41**).

Figure S6: Photograph of semi-quantitative test (Quantofix® test strips) used to determine residual nickel content. Top 3 strips: formulated final product **[^{18}F]41**. Bottom strip = Stock solution of $NiBr_2$.glyme diluted to concentration of 250 mg/ L.

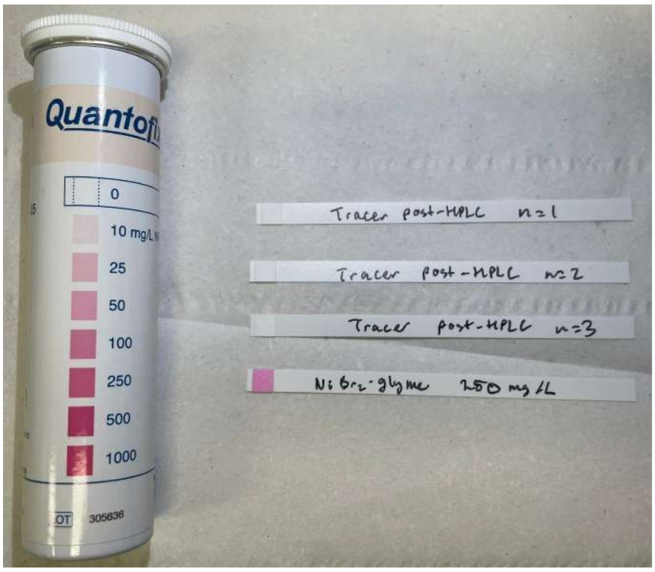

1.11.7 TRASIS AllinOne Run 2: Synthesis of [^{18}F]41 using ^{t}BuPyBCAM as Ligand

The semi-automated radiosynthesis of (**[^{18}F]41**, Scheme S7) was performed on a TRASIS AllInOne synthesizer using a prebuilt cassette.

Method as described in **Section 1.11.1** using ^{t}BuPyBCAM as ligand (7 µmol, 2 mg).

Scheme S7: TRASIS AllinOne automated synthesis of **[^{18}F]41** from cartridge purification and subsequent cross-coupling of **[^{18}F]5** using PyBCAM as ligand.

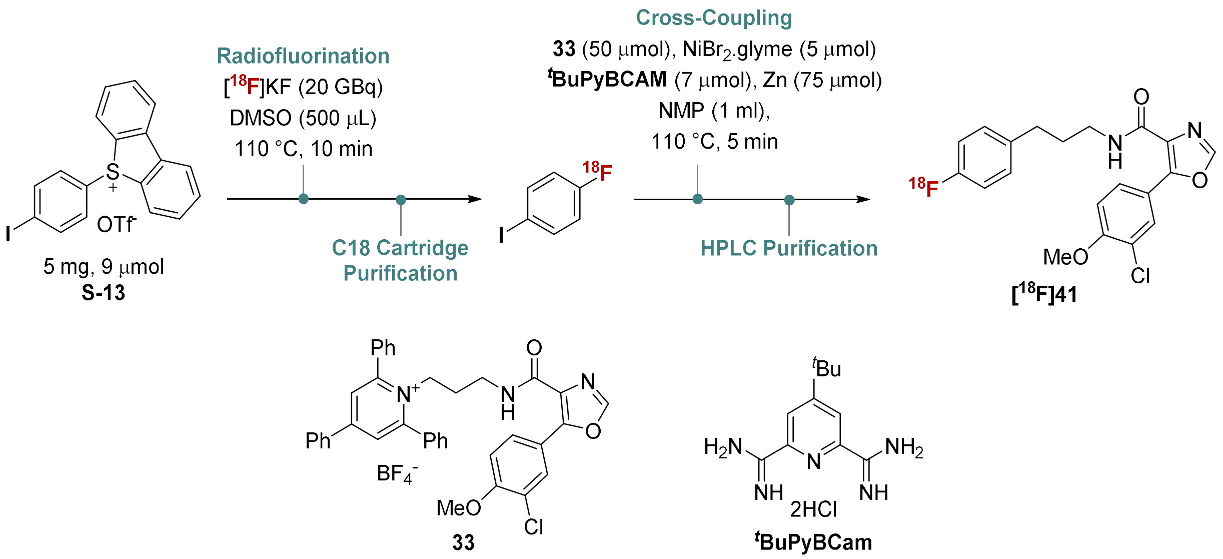

Activity yield: 2.80 GBq from 20 GBq, 14% n.d.c.

Synthesis time: 99 minutes

RCP: > 99%

1.12 Considerations for Adapting 2-Step Radiofluorination/Cross-Coupling to GE TRACERlab FX2 N and Synthra RNPlus Research

Heat-Transfer: In the semi-automated protocol developed on the TRASIS AllinOne, the second reactor was not used due to lack of stirring capability. Therefore, the second reaction took place on a pre-heated external IKA RCT digital. As pre-heating of the secondary reactors on both GE TRACERlab FX2 N and Synthra RNPlus is not feasible, longer reaction times were used to ensure the reaction is heated sufficiently to 110 °C.

Heterogenous Reaction Considerations: Across all three tested automated synthesis platforms, no pressure build-up was observed in the reaction. Zinc appeared to remain on the flask walls. It can therefore be concluded that zinc does not block liquid transfer lines. For the TRASIS AllinOne system, a hydrophilic filter unit [Whatman UniFLO 25 mm, 0.2 μm PES filter media, catalogue # 9914-2502] was fitted to the HPLC load line to prevent zinc entering the HPLC system and reduce pressure build-up in the loading step.

1.13 GE TRACERlab FX2 N Automated Synthesis of [^{18}F]41

1.13.1 GE TRACERlab FX2 N General Information and HPLC conditions

[^{18}F]Fluoride was produced by PETNET Solutions, Inc. (USA) via the $^{18}O(p,n)^{18}F$ reaction and delivered as [^{18}F]fluoride in [^{18}O]H_2O.

A TRACERlab FX2 N (GE HealthCare) was used for fully automated synthesis including semi-preparative purification of the radiolabelled product using the integrated HPLC system.

Analytical Radio-high-performance liquid chromatography (radio-HPLC) was performed on Agilent 1200 equipped with Eckert & Ziegler Flow-Count FC-3200 Detector System.

HPLC Conditions

Analytical Reverse Phase HPLC: Phenomenex Gemini C18 column (5 μm, 4.6 mm x 250 mm). **Mobile Phase:** solvent (A) H_2O + 0.1% TFA, solvent (B) MeCN + 0.1% TFA. **Flow rate:** 1 mL/min. **UV wavelength:** 254 nm. **Gradient:** 0 - 16 min = 65% B, 16-20 min = 65-95% B, 20-21 min = 95-65% B, 21-22 min = 65 % B.

Semi-Preparative Reverse Phase HPLC: Agilent Eclipse XDB-C18 column (5 μm, 9.4 mm x 250 mm)**. Mobile phase:** MeCN:H_2O 60/40 (Isocratic). **Flow rate** = 4 mL/min. **UV wavelength:** = 254 nm.

1.13.2 GE TRACERlab FX2 N Run: Synthesis of [^{18}F]41 using PyBCAM as Ligand

The automated radiosynthesis of (**[^{18}F]41**, Scheme S8) was performed on a GE TRACERlab FX2 automated synthesizer using PyBCAM as ligand and reduced loadings of both fluorination and cross-coupling reaction components.

Scheme S8: GE TRACERlab FX2 automated synthesis of **[^{18}F]41** from cartridge purification and subsequent cross-coupling of **[^{18}F]5** using PyBCAM as ligand.

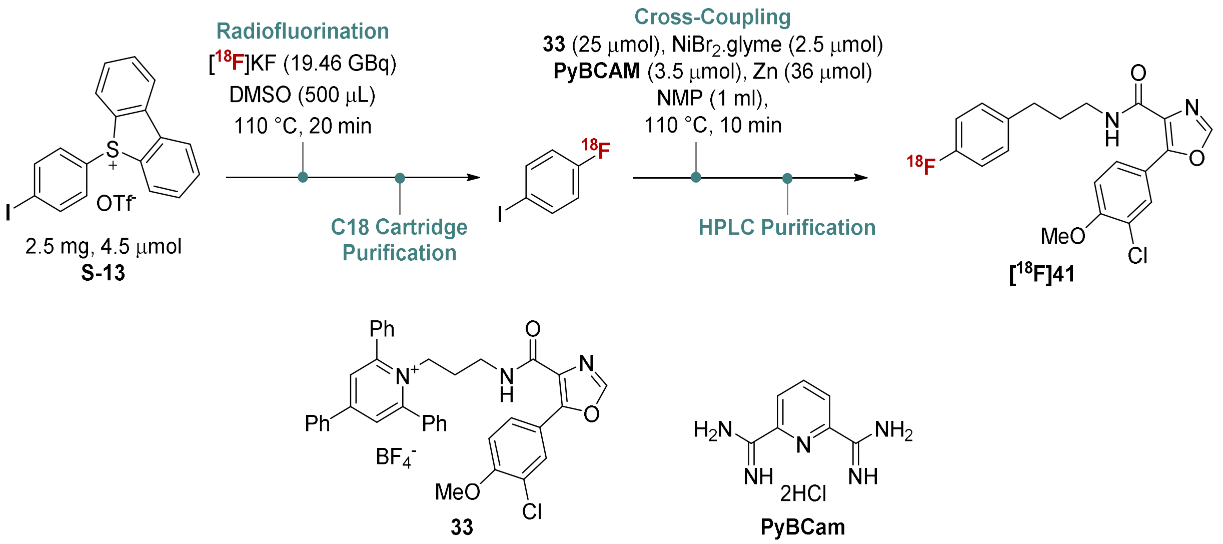

Vial Preparation:

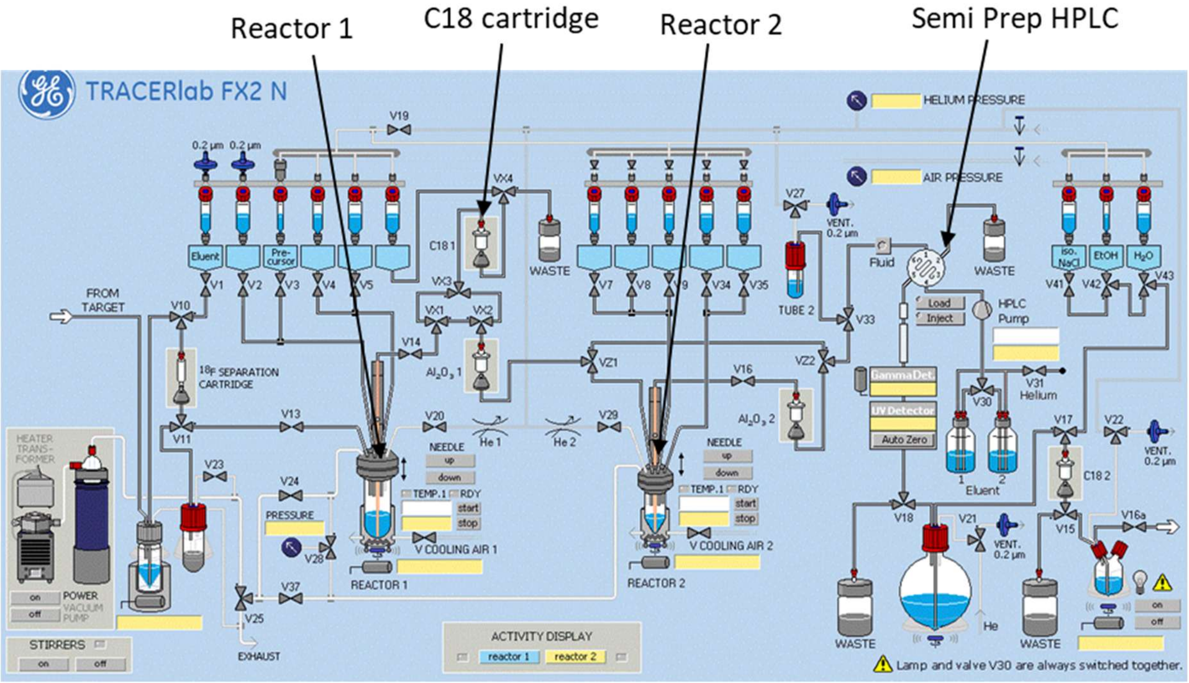

Vial 1 Elution solution: K_{222} (5.6 mg) and $KHCO_3$ (1.5 mg) were dissolved in 0.5 mL; 85:15 v/v MeCN /H_2O

Vial 2 Anhydrous MeCN (1 mL)

Vial 3 **S-13** (2.5 mg) in 0.5 mL anhydrous DMSO

Vial 4 H_2O (10 mL)

Vial 5 Anhydrous NMP (1 mL) [C18 elution]

Reactor 2 **33** (17 mg, 25 μmol), $NiBr_2$.glyme (0.8 mg), PyBCAM (0.8 mg), activated zinc (2.3 mg) along with spin vane (*not* a cylindrical stirrer bar).

Wash 1 H_2O (0.5 mL)

Wash 2 H_2O (1.5 mL)

Dilution Flask H_2O (35 mL)

Vial 12 saline (0.9%, 4.5 mL)

Vial 13 EtOH (0.5 mL)

Vial 14 H_2O (10 mL) [C18 wash]

Cartridge Preparation:

QMA SepPak chloride 46 mg (Waters SKU WAT186004540) cartridge was preconditioned with the following in the order given: aq. NaOH (1M, 5 mL), H_2O (10 mL), aq. K_2CO_3 (1M, 1 mL), H_2O (10 mL), and flushed with air (10 mL).

Both C18 SepPak 130 mg (Waters SKU WAT023501) cartridges were preconditioned with EtOH (10 mL) followed by H_2O (10 mL) and flushed with air.

Method

Fluorination Reaction: [^{18}F]Fluoride (19.46 GBq in 5.6 mL of [^{18}O]H_2O) was trapped on the QMA cartridge. Elution solution (Vial 1) was passed through the QMA cartridge eluting [^{18}F]KF which was dried azeotropically in Reactor 1 by heating to 85 °C with MeCN under vacuum and a flow of N_2 for 4 minutes. Anhydrous MeCN (1 mL) was added, and the evaporation procedure was repeated at 110 °C for 1.5 min. The fluorination reaction vial was cooled down to 40°C. **S-13** (2.5 mg, 4.5 μmol) in DMSO (0.5 mL) was added to reaction vial and the reaction mixture heated at 110 °C for 20 min.

[^{18}F]5 C18 purification and cross-coupling: The mixture was cooled down to 40°C and diluted with H_2O (10 mL) and the diluted crude reaction mixture passed through a C18 cartridge. NMP (1 mL) was passed through the C18 cartridge eluting [^{18}F]4-fluoroiodobenzene into Reactor 2 (containing cross-coupling reaction components, see above). Reactor 2 was heated to 110 °C for 10 minutes and then cooled down to 30 °C.

[^{18}F]41 Purification and Reformulation: H_2O (0.5 mL + 1.5 mL) to Reactor 2 and purified by semi-preparative HPLC. The fraction containing purified **[^{18}F]41** was collected in a flask containing H_2O (35 mL) and then passed through a SepPak Light C18 cartridge which was subsequently washed with H_2O (10 mL). **[^{18}F]41** was eluted from C-18 cartridge with EtOH (0.5 mL) and the resulting solution diluted with saline (0.9%, 4.5 mL) and transferred to the final product vial.

Activity yield: 2.35 GBq from 19.5 GBq, 12% n.d.c.

Synthesis time: 93 minutes

Radiochemical Purity (RCP): > 99%

1.13.2 GE TRACERlab FX2 N: Semi-Preparative HPLC Trace for Purification of [^{18}F]41

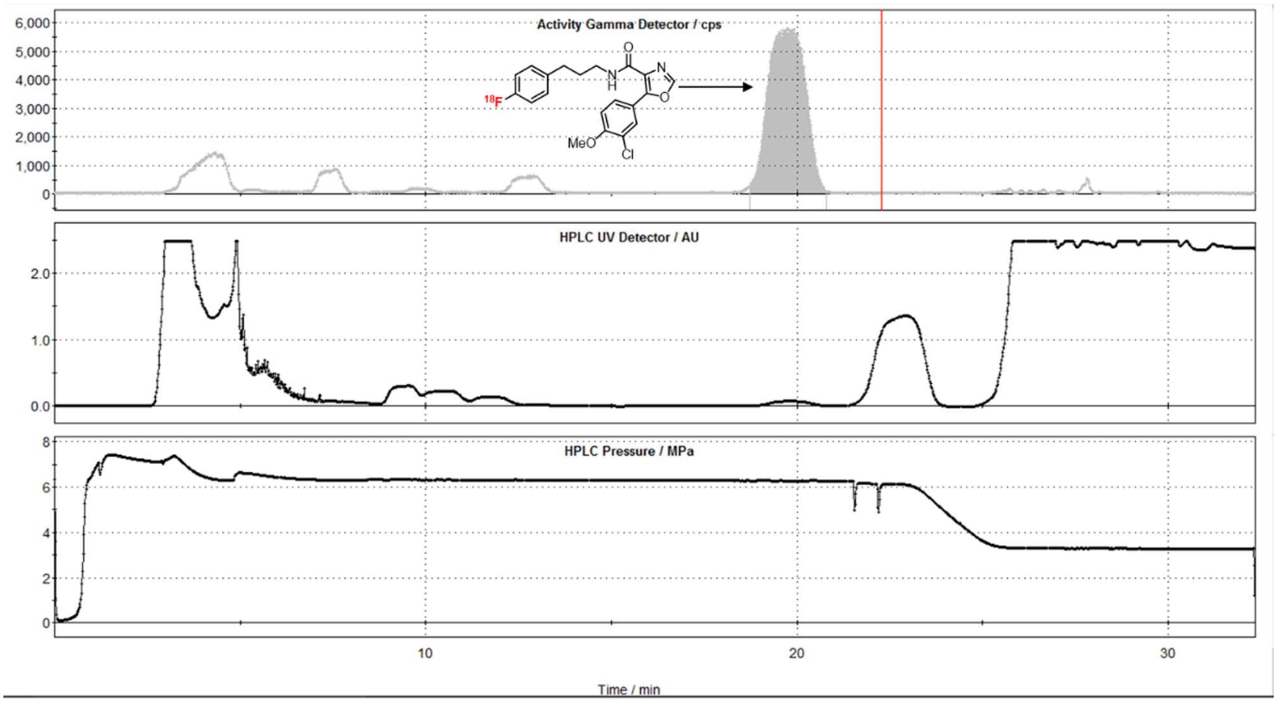

1.13.3 GE TRACERlab FX2 N: Quality Control of [^{18}F]41 Analytical HPLC Traces

An aliquot of non-radioactive reference of product **41** was mixed into a purified sample of **[^{18}F]41**. Radiochemical purity (RCP) was determined to be > 99%.

Figure S7: Co-injection of **[^{18}F]41** with **41** on analytical HPLC

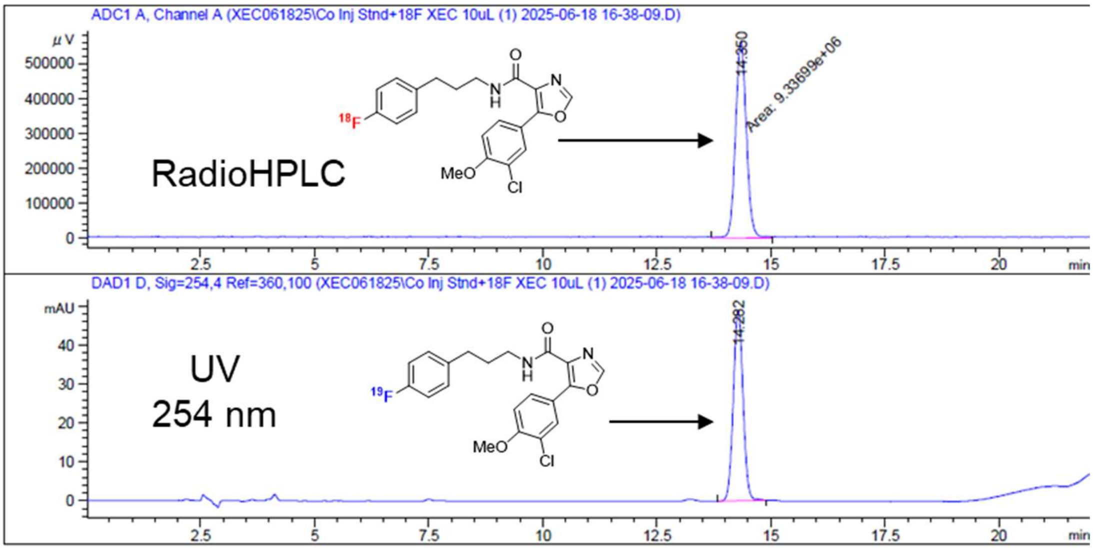

Figure S8: Analytical RadioHPLC of purified **[^{18}F]41**

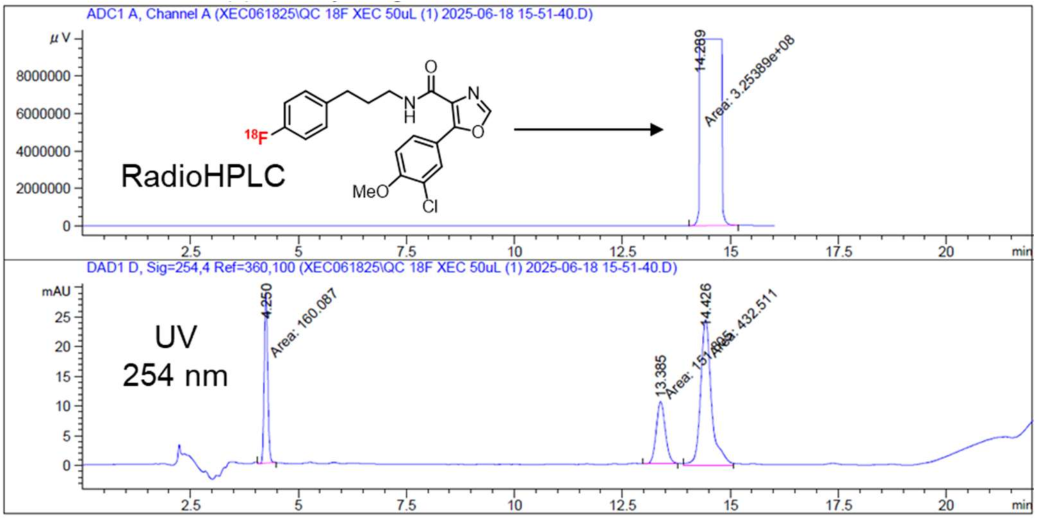

1.14 Synthra RNPlus Research Automated Synthesis of [^{18}F]5-(3-chloro-4-methoxyphenyl)-*N*-(3-(4-fluorophenyl)propyl)oxazole-4-carboxamide

1.14.1 Synthra RNPlus Research General Information and HPLC conditions

[^{18}F]Fluoride was produced by PETNET Solutions, Inc. (USA) via the ^{18}O(p,n)^{18}F reaction and delivered as [^{18}F]fluoride in [^{18}O]H_2O.

A Synthra RNPlus Research (Synthra GmbH) was used for fully automated synthesis including semi-preparative purification of the radiolabelled product using the integrated preparative HPLC system.

Analytical Radio-high-performance liquid chromatography (radio-HPLC) was performed on Agilent 1260 Infinity II equipped with a GABi NOVA (Elysia-Raytest) gamma detector.

HPLC Conditions

Analytical HPLC condition: Phenomenex Kinetex EVO C18 column (5 µm, 150 x 4.6mm). **Mobile phase:** solvent (A) MeCN + 0.1% TFA, solvent (B) H_2O + 0.1% TFA. **Flow rate:** 1 mL/min. **UV wavelength:** 254 nm. **Gradient:** 0-1 min = 5% B, 1-7 min = 5-95% B, 7-10 min = 95 % B, 10-10.5 min = 95-5% B, 10.5-12 min = 5% B.

Semi-Preparative Reverse Phase HPLC: Agilent Zorbax Eclipse C18 Column (5 µm, 9.4 mm x 250 mm). **Mobile phase:** MeCN:H_2O 60/40 (Isocratic). **Flow rate** = 4 mL/min. **UV wavelength:** = 254 nm.

1.14.2 Synthra RNPlus Research Method

The automated radiosynthesis of **[^18^F]41** (Scheme S8) was performed on a Synthra RNPlus Research automated synthesizer.

Scheme S9: Synthra RNPlus Research automated synthesis of **[^{18}F]41** from cartridge purification and subsequent cross-coupling of **[^{18}F]5** using PyBCAM as ligand.

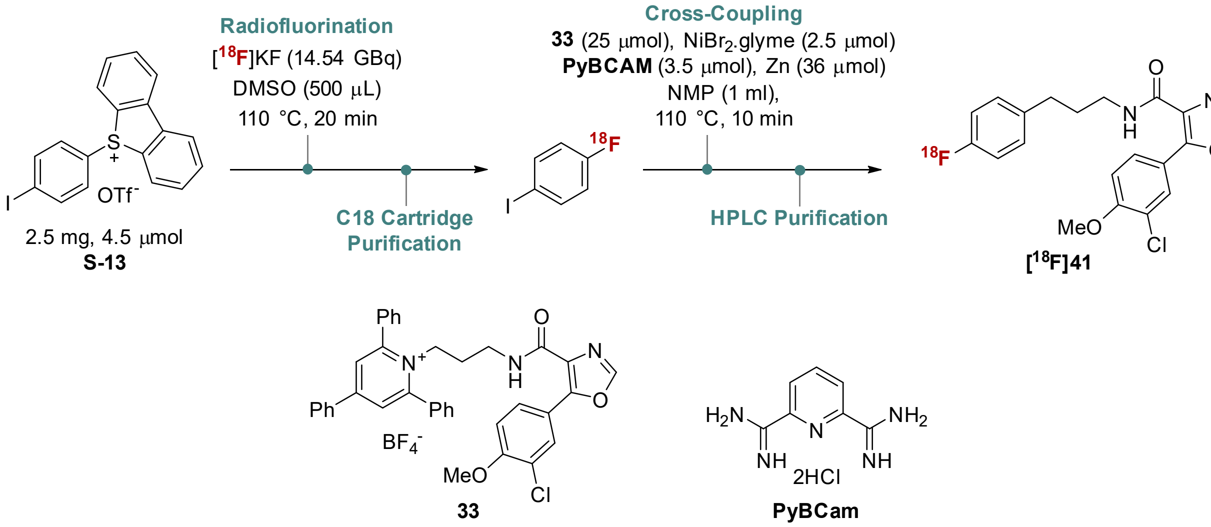

Vial Preparation:

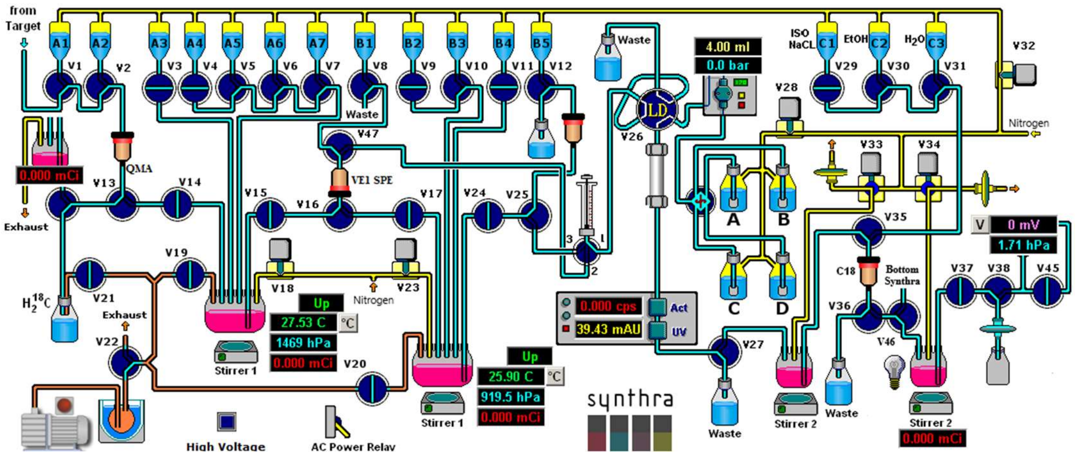

QMA: Waters Sep-Pak Accell Plus QMA Carbonate Plus Light Cartridge (Waters SKU 186004540) cartridge, preconditioned with H_2O (10 mL).

VE1 SPE: Waters Sep-Pak C18 Plus Short Cartridge (Waters SKU WAT020515), preconditioned with MeCN (10 mL), H_2O (10 mL), and air (10 mL).

Reformulation cartridge: Waters Sep-Pak C18 Plus Light Cartridge (Waters SKU WAT023501), preconditioned with MeCN (10 mL) and H_2O (10 mL).

Position A1: Vial containing $KHCO_3$ (1.5 mg), K_{222} (5.6 mg), in MeCN:H_2O (500 µL, 85:15 v/v).

Position A2: MeCN (0.7 mL).

Position A3: 5-(4-iodophenyl)-5H-dibenzo[*b,d*]thiophen-5-ium triflate (**S-13**) (2.5 mg, 5 µmol) in anhydrous DMSO (500 µL).

Position A4: H_2O (4 mL).

Position B1: NMP (1 mL)

Position B2: H_2O (2 mL) and MeCN (0.5 mL)

Position C1: Saline (0.9%, 4.5 mL)

Position C2: EtOH (0.5 mL)

Position C3: H_2O (5 mL)

HPLC collection dilution flask: H_2O (30 mL)

Second Reactor: Activated zinc (2.5 mg), **33** (17.6 mg, 25 µmol), PyBCAM (2.2 mg) and $NiBr_2$.glyme (1.7 mg).

Method

Fluorination Reaction: [^{18}F]fluoride (14.54 GBq in 5.6 mL of [^{18}O]H_2O) was trapped on the QMA cartridge. Elution solution [Position A1] was passed through the QMA cartridge eluting [^{18}F]KF into reactor 1 which was subsequently dried azeotropically under reduced pressure and a stream of N_2 at 90 °C. MeCN (700 µL) [Position A2] was added and the drying repeated at 100 °C. The reactor was then cooled to 70 °C and **S-13** (2.5 mg, 5 µmol) in anhydrous DMSO (500 µL) [Position A3] subsequently added and heated to 110 °C for 20 min.

[^{18}F]5 C18 purification and cross-coupling: The reactor was cooled to 40 °C and H_2O (4 mL) [Position A4] was added followed by transfer of the diluted fluorination reaction mixture through the C18 Plus Short cartridge. The cartridge was dried by flowing N_2 for 5 minutes. C18 elution using NMP (1 mL) [Position B2] into Reactor 2 released [^{18}F]-4-fluoroiodobenzene. Reactor 2 was heated to 110 °C for 10 minutes.

[^{18}F]41 Purification and Reformulation: H_2O (2 mL) and MeCN (0.5 mL) [Position B2] were added to the reactor and loaded onto the semi-preparative HPLC ($\boldsymbol{t_R}$ = 22 min). The desired peak was collected into the HPLC collection flask which was mixed and then transferred through the C18 Plus Light cartridge. The cartridge was washed with H_2O (5 mL) [Position C3] and dried for 2 minutes by a pressure of N_2. The purified product was eluted from the C18 cartridge by EtOH (0.5 mL) [Position C2] followed by Saline (0.9%, 4.5 mL) [Position C1].

The formulated product was then transferred out of the synthesizer into a product vial in 10 % EtOH in saline.

Activity yield: 555 MBq from 14.54 GBq, 3.8% n.d.c.

Synthesis time: 103 minutes

Radiochemical Purity (RCP): >99%

1.14.3 Synthra RNPlus Research: Semi-Preparative HPLC Trace for Purification of [^{18}F]41

Retention time = 22 mins.

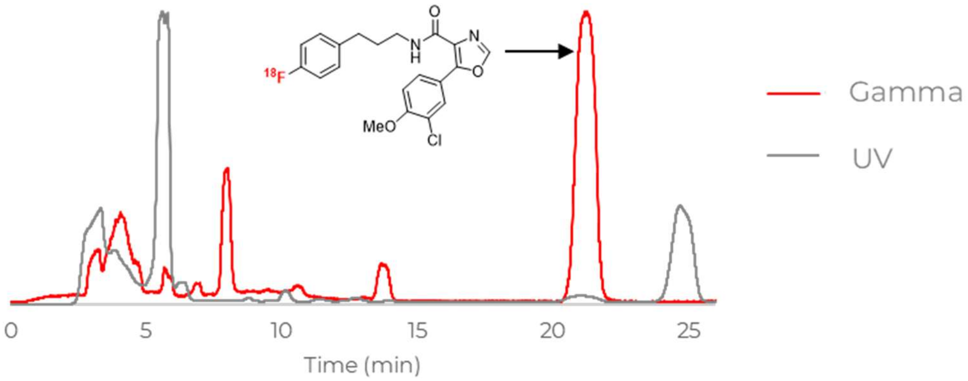

1.14.4 Synthra RNPlus Research: Quality Control of [^{18}F]41 – Analytical HPLC Traces

Figure S9: Co-injection of **[^{18}F]41** with **41** on analytical HPLC

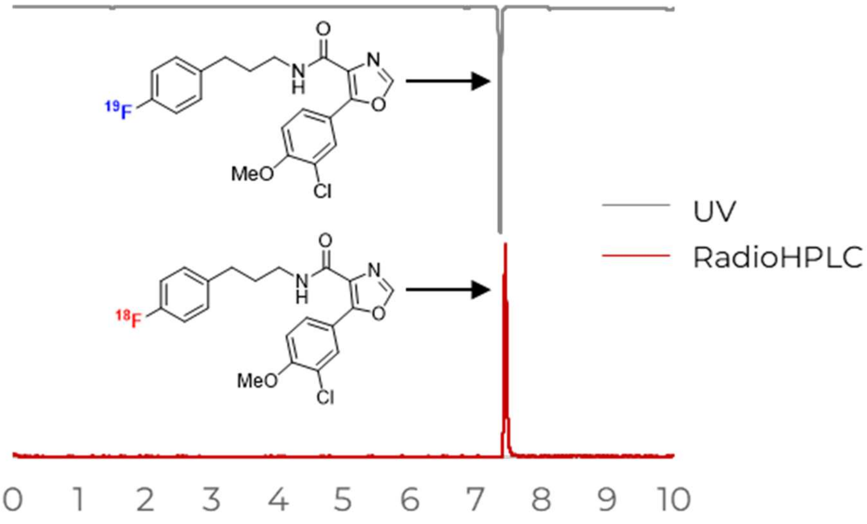

Figure S10: Analytical RadioHPLC of purified **[^{18}F]41**

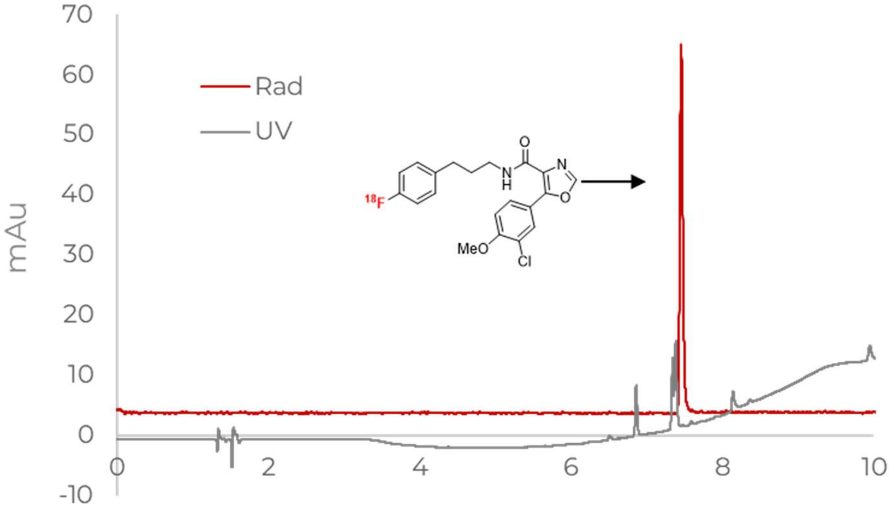

1.15 Radiotrace Overlays and Radiochemical Conversions

All radiotrace overlays use **Analytical HPLC Condition A** (**Section 1.2**) unless otherwise specified.

1.15.1 Entries with [^{18}F]5 as the coupling partner

All RCC (%) values are using the conditions specified in Scheme 2 and Scheme 3 in the manuscript.

[^{18}F]1-fluoro-4-phenethylbenzene ([^{18}F]3)

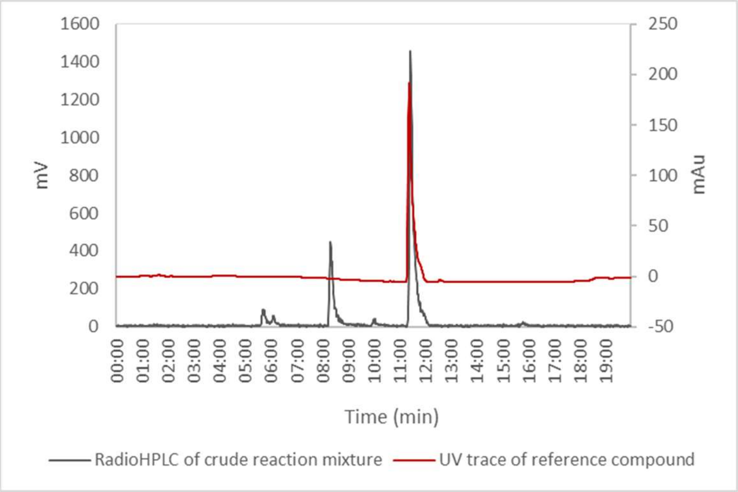

Entry	RCC (%)
**1**	74
**2**	75
**3**	71
**4**	76
**5**	71
**6**	72
**7**	73
**8**	68
**Average**	73 ± 2

[^{18}F]2-(4-Fluorophenethyl)thiophene ([^{18}F]6)

Analytical HPLC Condition F

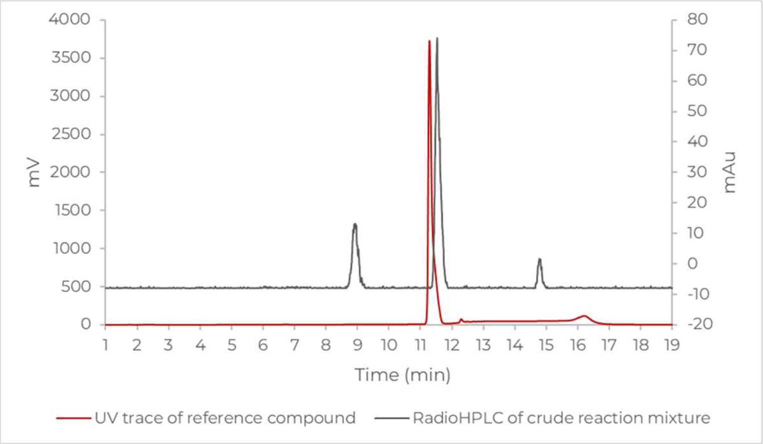

Entry	RCC (%)
**1**	73
**2**	70
**3**	80
**Average**	74 ± 4

[^{18}F]*tert*-butyl 4-(4-fluorophenethyl)piperazine-1-carboxylate ([^{18}F]7)

Analytical HPLC Condition C. HPLC Wavelength = 200 nm

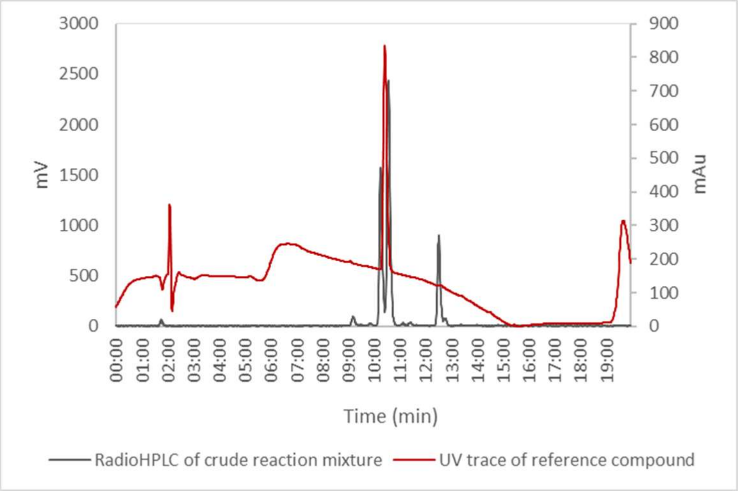

Entry	RCC (%)
**1**	76
**2**	75
**3**	86
**Average**	79 ± 5

[^{18}F]-4-ethyl-1-(4-fluorophenethyl)-*1H*-1,2,3-triazole ([^{18}F]8)

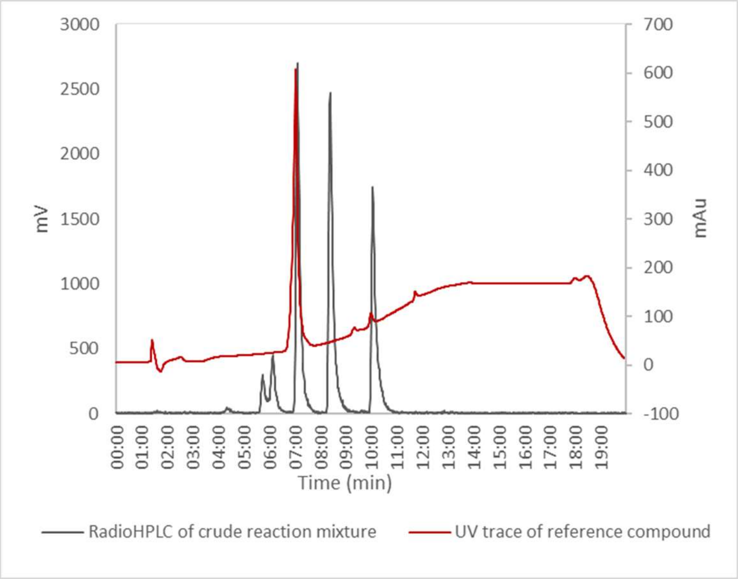

Entry	RCC (%)
**1**	33
**2**	32
**3**	22
**Average**	29 ± 5

[^{18}F]-3-(4-fluorophenethyl)pyridine ([^{18}F]9)

Analytical HPLC Condition C

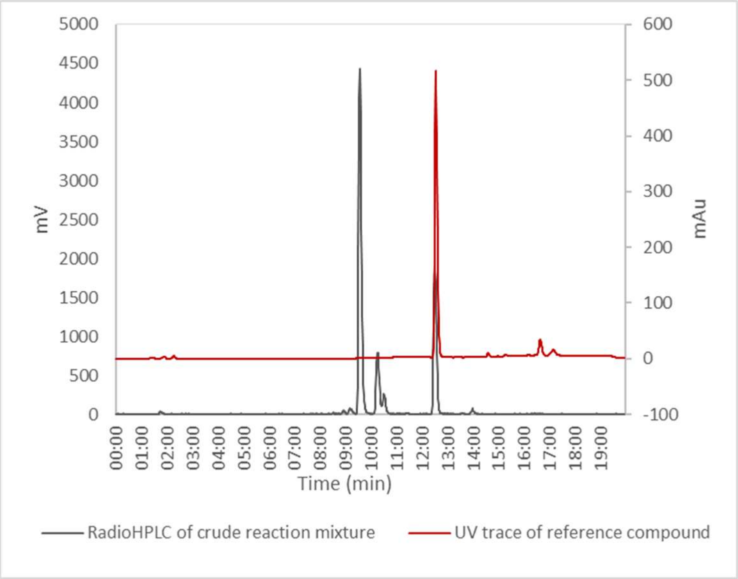

Entry	RCC (%)
**1**	58
**2**	64
**3**	69
**Average**	64 ± 4

[^{18}F]*tert*-butyl 3-(4-fluorobenzyl)azetidine-1-carboxylate ([^{18}F]10)

HPLC Wavelength = 207 nm

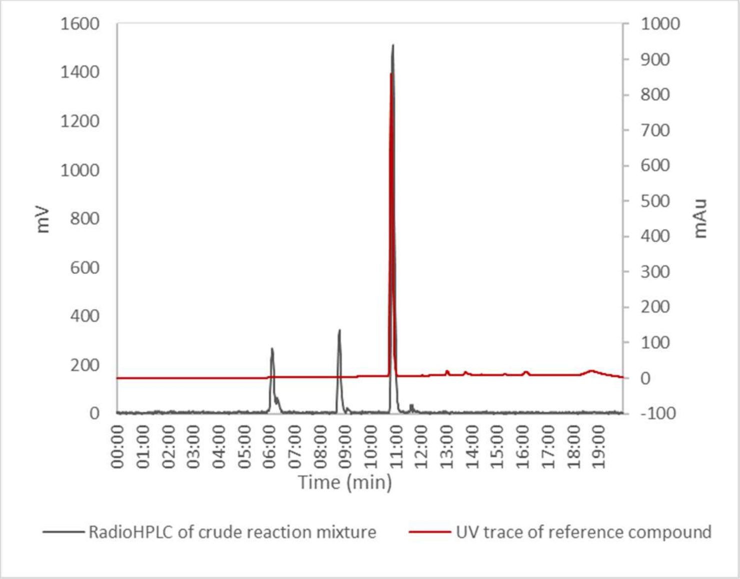

Entry	RCC (%)
**1**	69
**2**	55
**3**	55
**Average**	60 ± 7

[^{18}F]1-(4-fluorophenethyl)pyrrolidine ([^{18}F]11)

Analytical HPLC Condition C

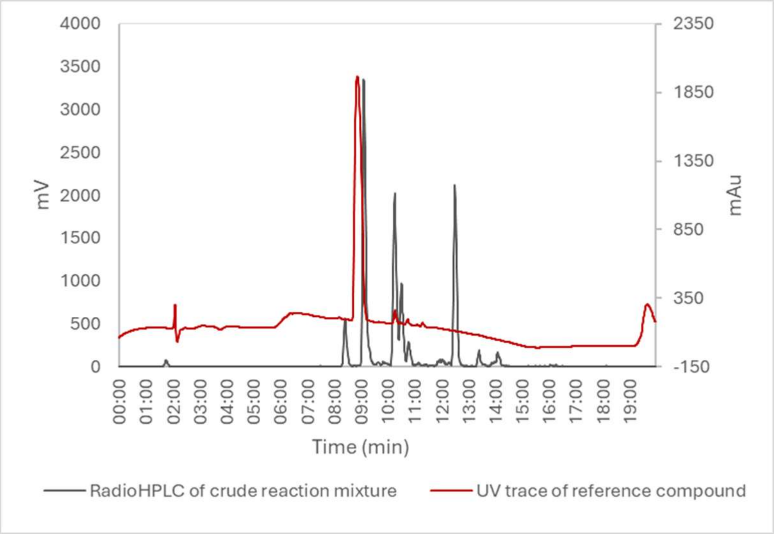

Entry	RCC (%)
**1**	33
**2**	34
**3**	32
**Average**	33 ± 1

[^{18}F]-3-(4-fluorophenyl)propan-1-ol ([^{18}F]12)

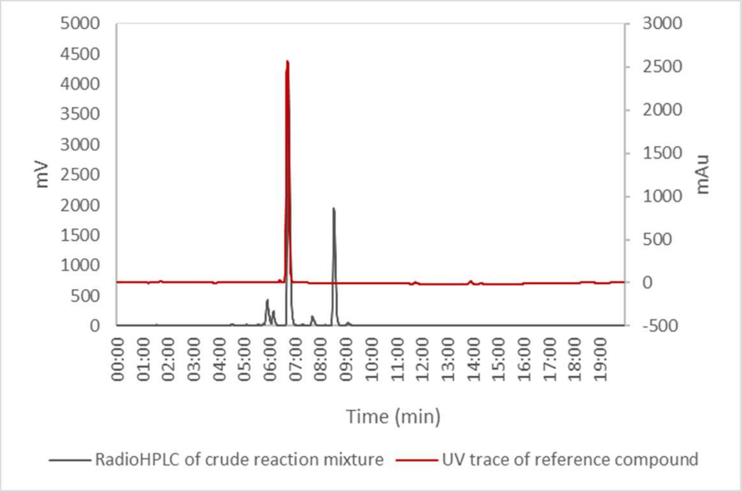

Entry	RCC (%)
**1**	71
**2**	56
**3**	60
**Average**	62 ± 6

[^{18}F]3-(4-fluorobenzyl)piperidine ([^{18}F]13)

Analytical HPLC Condition C

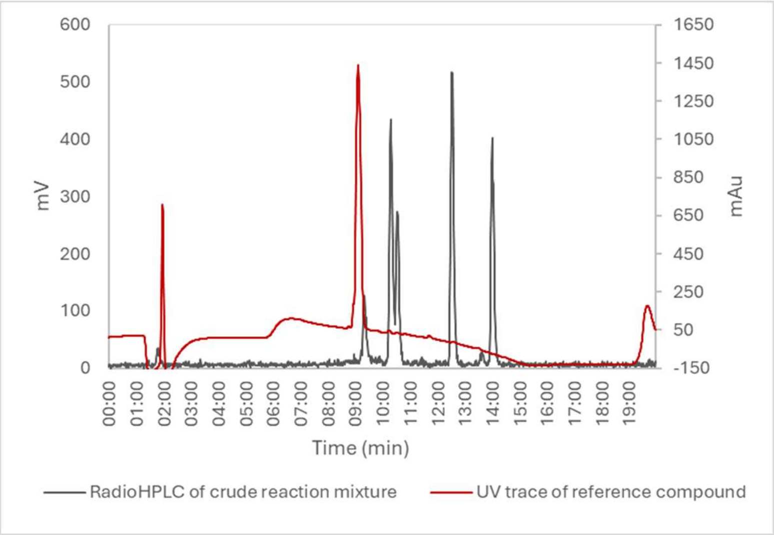

Entry	RCC (%)
**1**	22
**2**	22
**3**	8
**Average**	17 ± 7

[^{18}F]3-(4-Fluorophenethyl)-*1H*-indole ([^{18}F]14)

Analytical HPLC Condition F

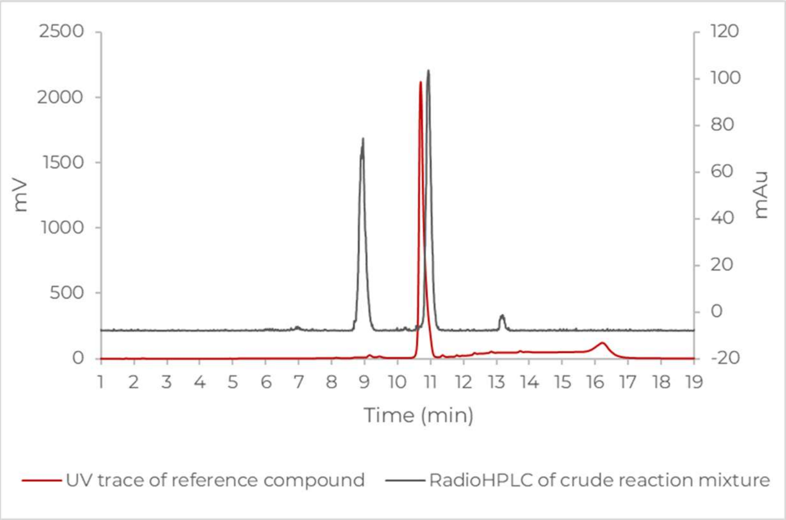

Entry	RCC (%)
**1**	52
**2**	66
**3**	64
**Average**	61 ± 6

[¹⁸F]5-(4-fluorophenethyl)benzo[*d*][1,3]dioxole ([¹⁸F]15)

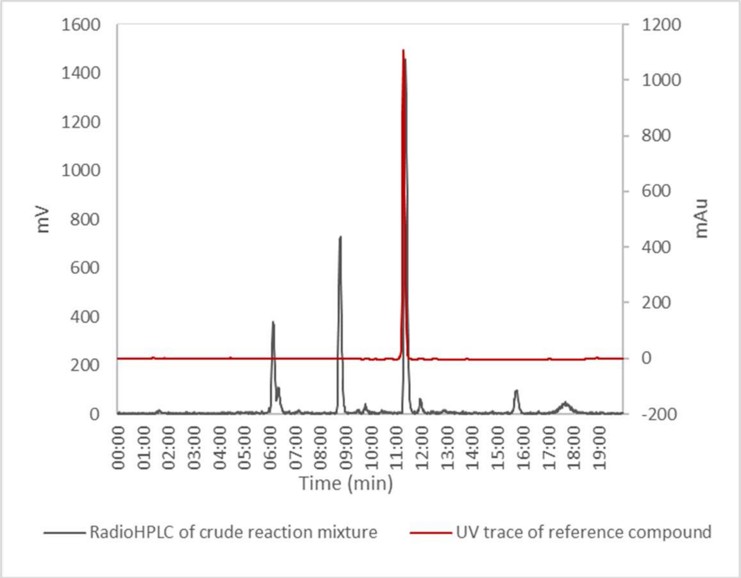

Entry	RCC (%)
**1**	44
**2**	54
**3**	48
**Average**	49 ± 4

[¹⁸F]1-(2-(cyclohex-1-en-1-yl)ethyl)-4-fluorobenzene ([¹⁸F]16)

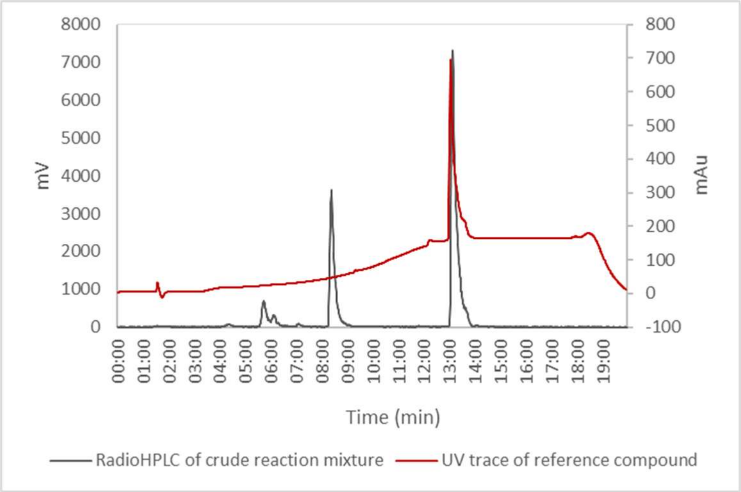

Entry	RCC (%)
**1**	76
**2**	57
**3**	62
**Average**	65 ± 8

[^{18}F](1*S*,2*S*,5*S*)-2-(4-fluorobenzyl)-6,6-dimethylbicyclo[3.1.1]heptane ([^{18}F]17)

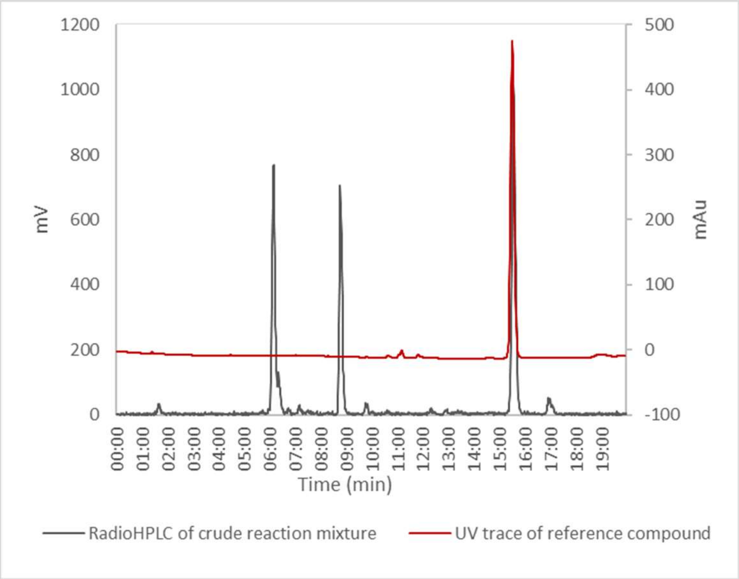

Entry	RCC (%)
**1**	54
**2**	55
**3**	45
**Average**	51 ± 4

[^{18}F]*tert*-butyl 2-((4*R*,6*R*)-6-(4-fluorophenethyl)-2,2-dimethyl-1,3-dioxan-4-yl)acetate ([^{18}F]18)

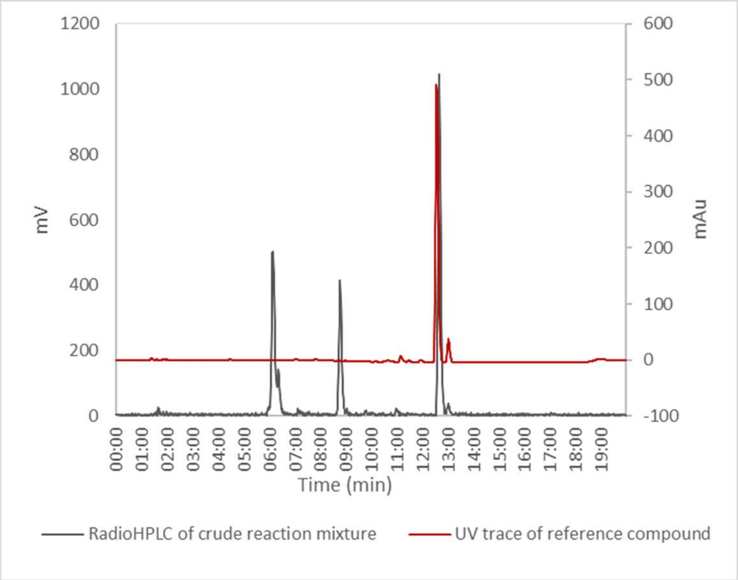

Entry	RCC (%)
**1**	38
**2**	30
**3**	49
**Average**	39 ± 8

[^{18}F]-5-methoxy-1-(4-(trifluoromethyl)phenyl)pentan-1-one *O*-(4-fluorophenethyl) oxime ([^{18}F]19)

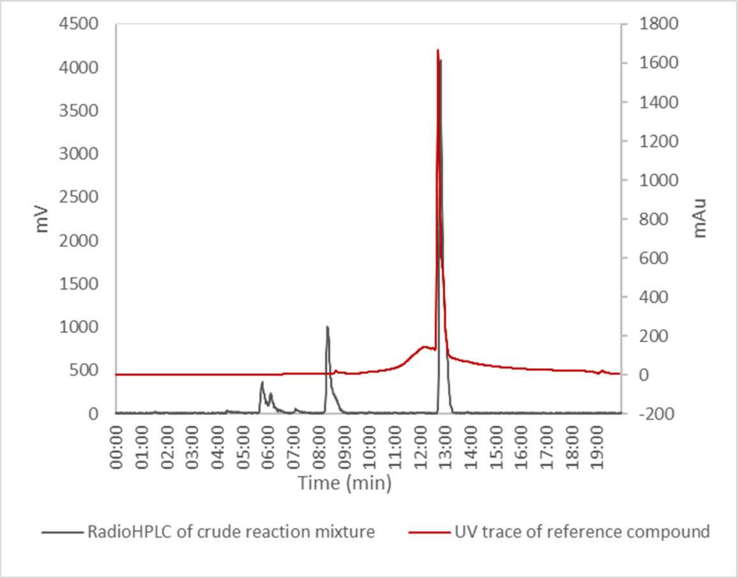

Entry	RCC (%)
**1**	68
**2**	43
**3**	57
**Average**	56 ± 10

[^{18}F]-*N*-(5-(4-fluorophenyl)pentan-2-yl)-6-methoxyquinolin-8-amine ([^{18}F]20)

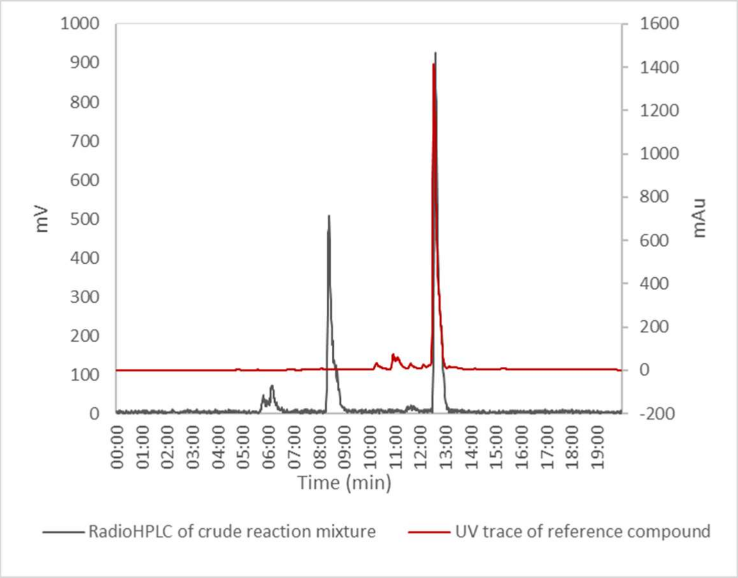

Entry	RCC (%)
**1**	45
**2**	57
**3**	34
**Average**	45 ± 9

[^{18}F]-3-ethyl 5-methyl 4-(2-chlorophenyl)-2-((4-fluorophenethoxy)methyl)-6-methyl-1,4-dihydropyridine-3,5-dicarboxylate ([^{18}F]21)

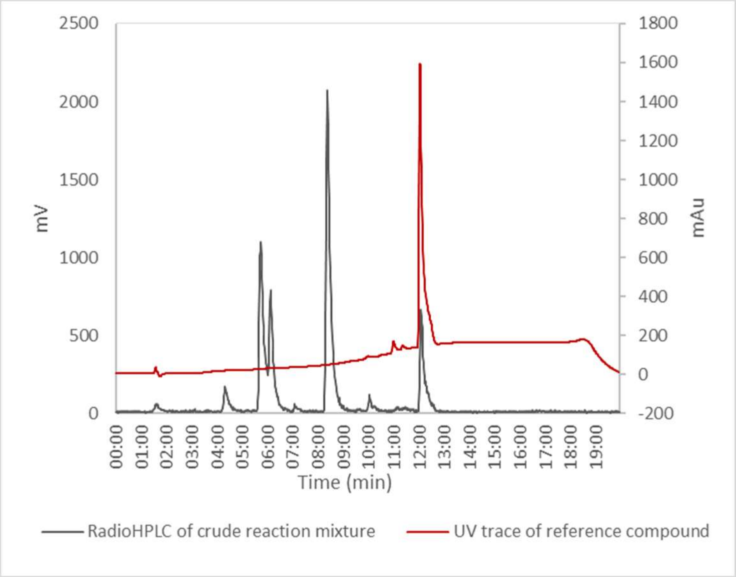

Entry	RCC (%)
**1**	39
**2**	23
**3**	13
**Average**	25 ± 11

[^{18}F]-1-(2-(4-fluorophenyl)-1-(4-methoxyphenyl)ethyl)cyclohexan-1-ol ([^{18}F]22)

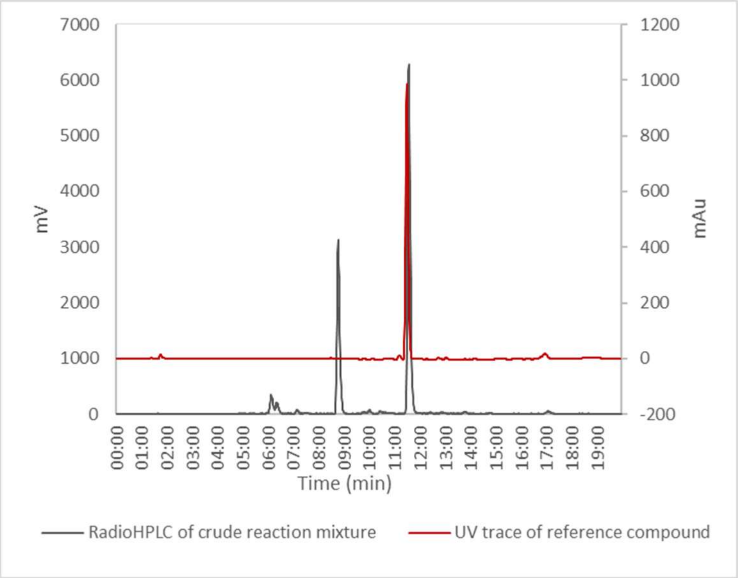

Entry	RCC (%)
**1**	63
**2**	55
**3**	49
**Average**	56 ± 6

[^{18}F]Methyl (*S*)-2-((*tert*-butoxycarbonyl)amino)-6-(4-fluorophenyl)hexanoate ([^{18}F]23)

Analytical HPLC Condition F

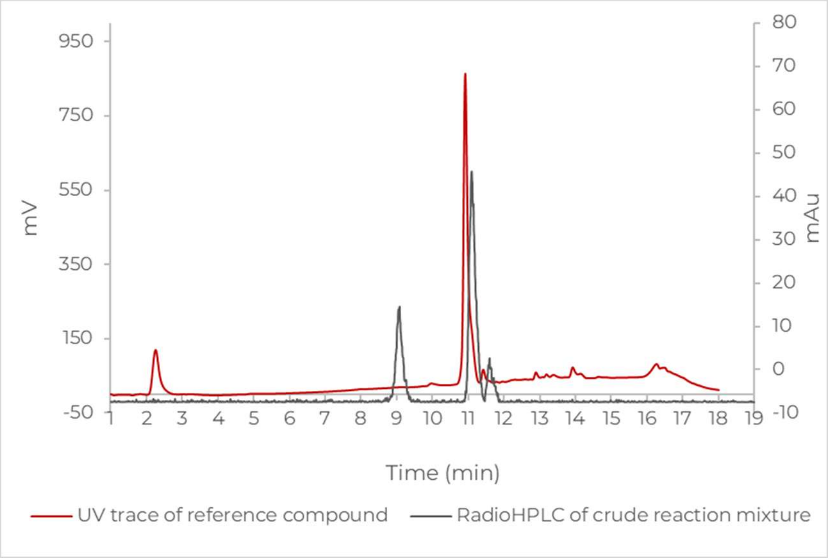

Entry	RCC (%)
**1**	63
**2**	52
**3**	63
**Average**	59 ± 5

[^{18}F]*di-tert*-butyl (((*S*)-1-(tert-butoxy)-6-(4-fluorophenyl)-1-oxohexan-2-yl)carbamoyl)-*L*-glutamate ([^{18}F]24)

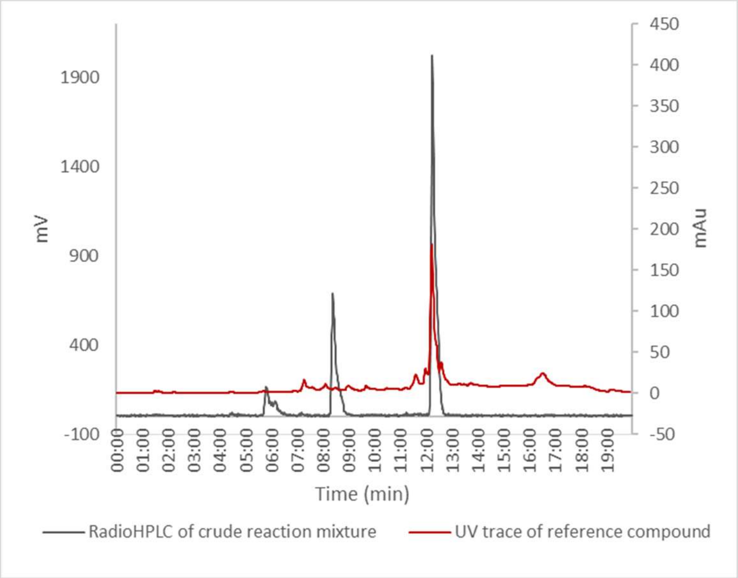

Entry	RCC (%)
**1**	31
**2**	65
**3**	53
**Average**	50 ± 14

[^{18}F]*N*-(4-fluorophenethyl)-4,6-dimethoxypyrimidin-2-amine ([^{18}F]25)

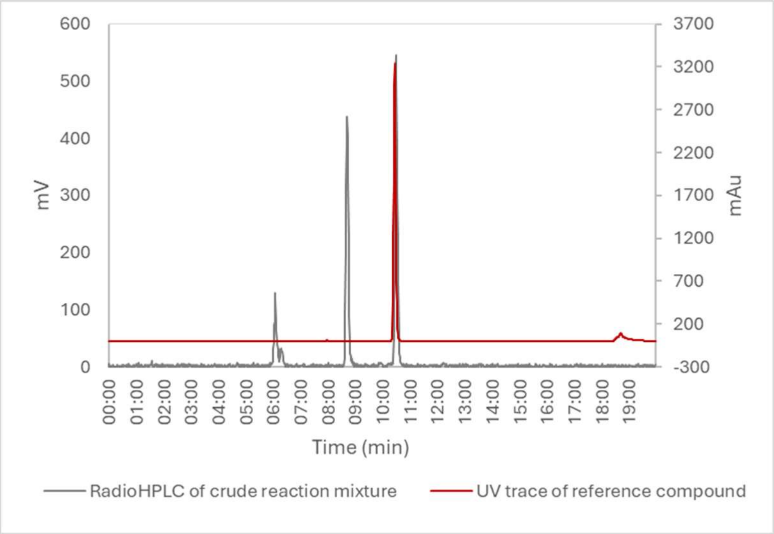

Entry	RCC (%)
**1**	49
**2**	49
**3**	37
**Average**	45 ± 6

[^{18}F]1-(2-(benzhydryloxy)ethyl)-4-(3-(4-fluorophenyl)propyl)piperazine ([^{18}F]26)

Analytical HPLC Condition C

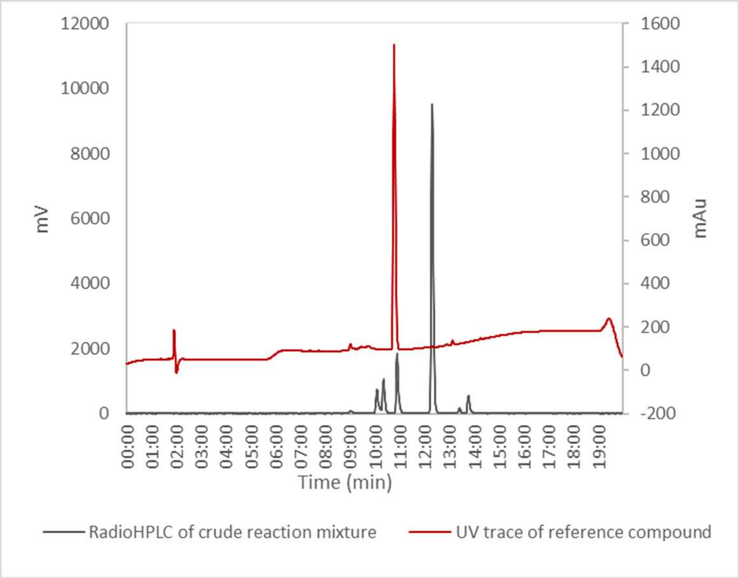

Entry	RCC (%)
**1**	27
**2**	13
**3**	11
**Average**	17 ± 7

[18F]Pruvanserin ([18F]27)

Analytical HPLC Condition C

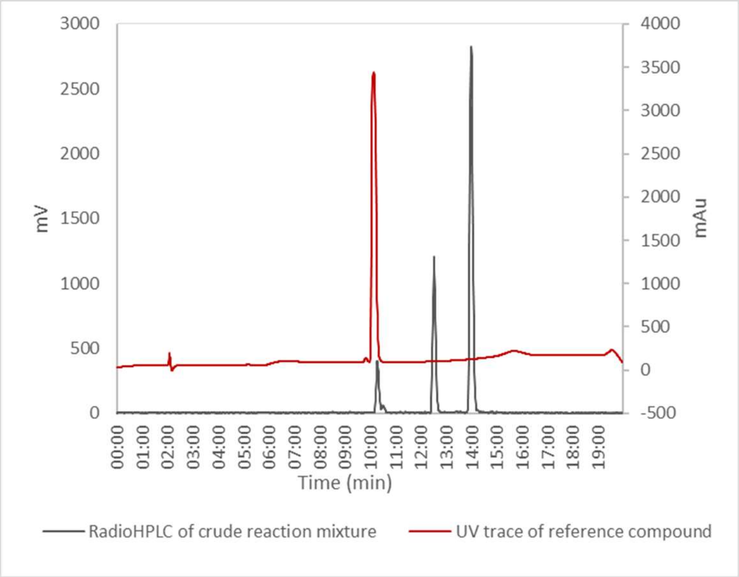

Entry	RCC (%)
**1**	8
**2**	5
**3**	10
**Average**	8 ± 2

[18F]*N*-(3-(4-fluorophenyl)propyl)-7-hydroxy-2-oxo-*2H*-chromene-3-carboxamide ([18F]28)

Analytical HPLC Condition D

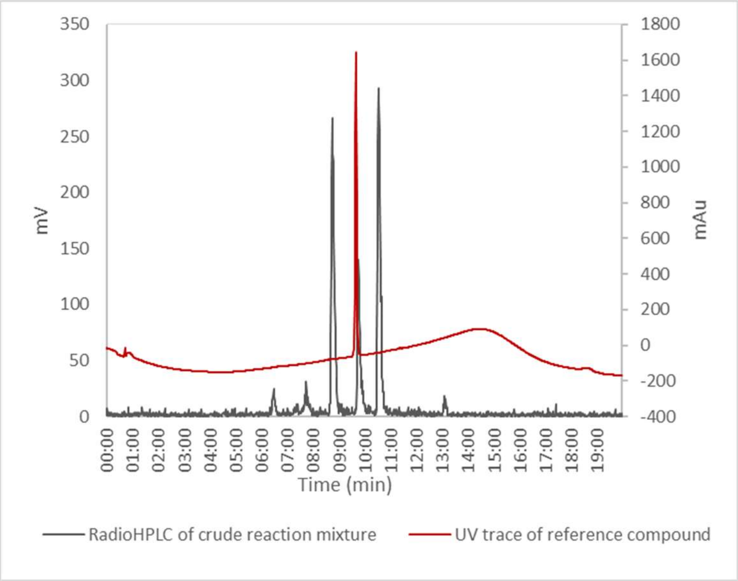

Entry	RCC (%)
**1**	14
**2**	10
**3**	20
**Average**	15 ± 4

[^{18}F]4-(4-fluorophenyl)tetrahydro-*2H*-pyran ([^{18}F]29)

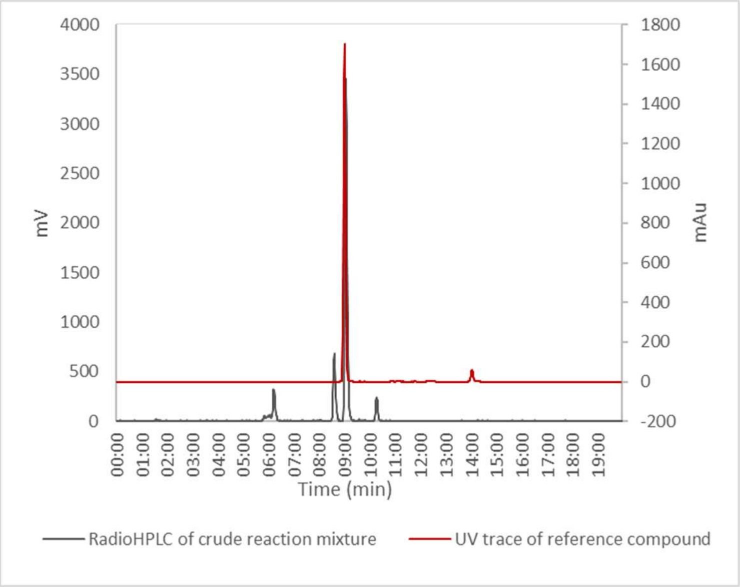

Entry	RCC (%)
**1**	68
**2**	71
**3**	62
**Average**	67 ± 4

[^{18}F]1-fluoro-4-(4-phenylbutan-2-yl)benzene ([^{18}F]30)

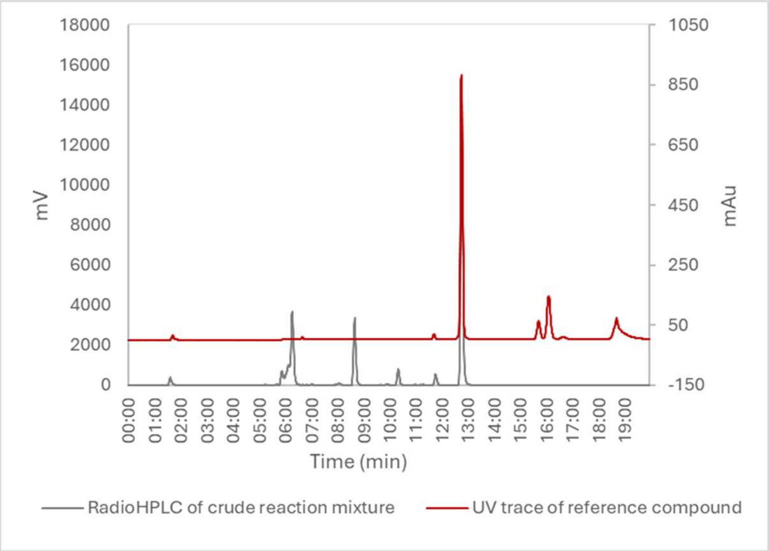

Entry	RCC (%)
**1**	58
**2**	71
**3**	69
**Average**	66 ± 6

[^{18}F]*tert*-butyl 4-(4-fluorophenyl)piperidine-1-carboxylate ([^{18}F]31)

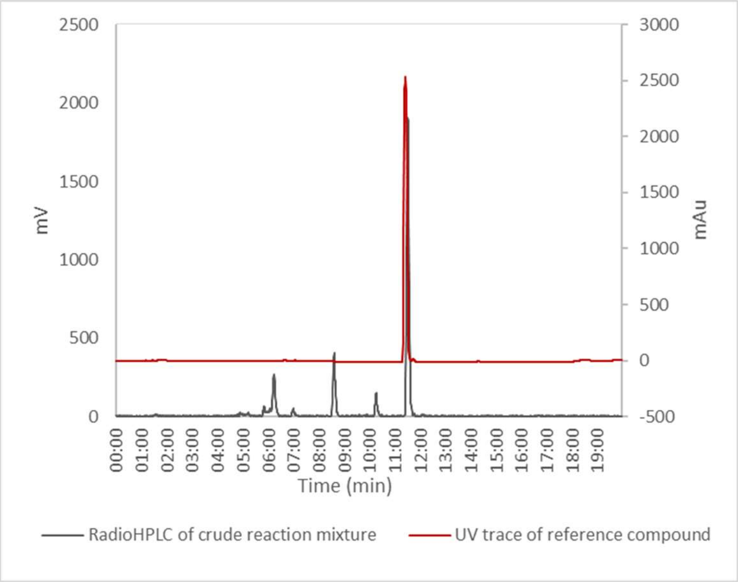

Entry	RCC (%)
**1**	54
**2**	56
**3**	65
**Average**	58 ± 5

[^{18}F]2-(4-(4-fluorophenyl)piperidin-1-yl)pyrimidine ([^{18}F]32)

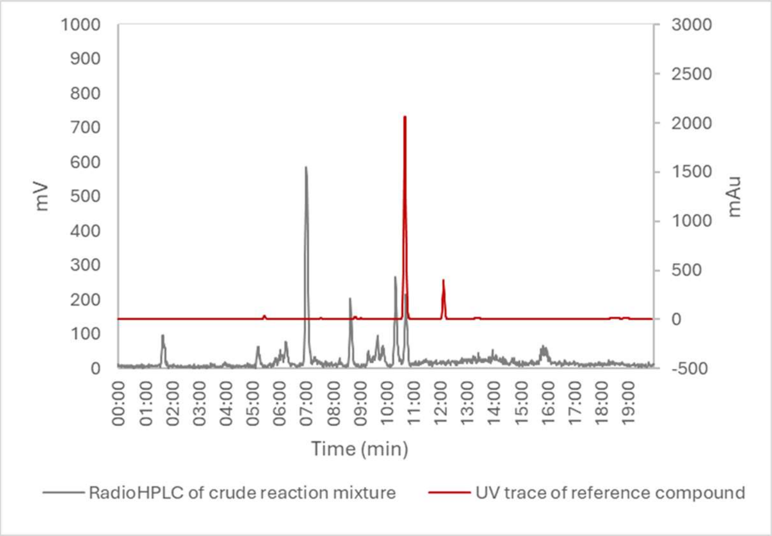

Entry	RCC (%)
**1**	11
**2**	5
**3**	6
**Average**	7 ± 3

[^{18}F]5-(3-chloro-4-methoxyphenyl)-*N*-(3-(4-fluorophenyl)propyl)oxazole-4-carboxamide ([^{18}F]41)

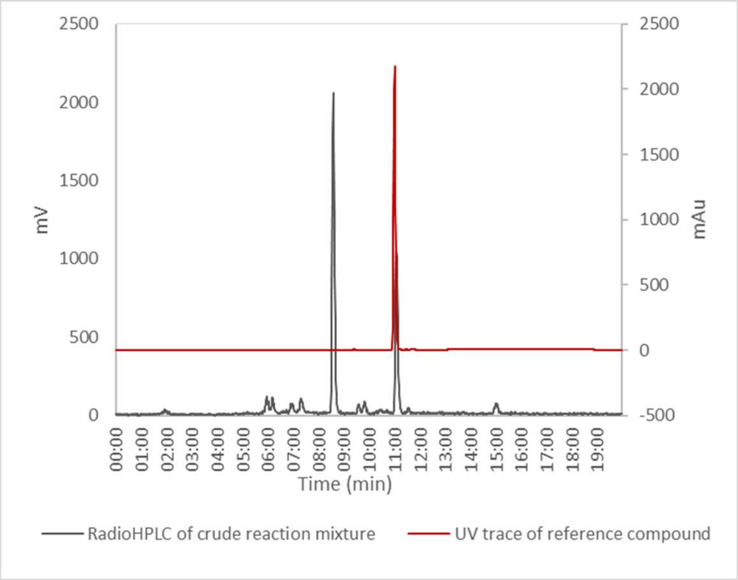

Entry	RCC (%)
**1**	44
**2**	35
**Average**	40 ± 5

1.15.2 Entries with Diversified ^{18}F-Labelled Fluoro(Hetero)aryl Iodides

Semi-preparative HPLC Purified [^{18}F]2-fluoro-iodobenzene ([^{18}F]37) Quality Check

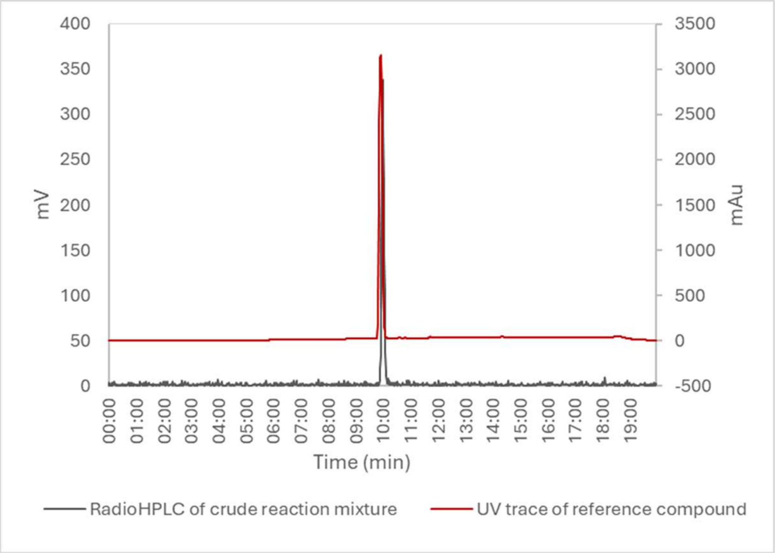

[^{18}F]1-fluoro-2-phenethylbenzene ([^{18}F]S-17)

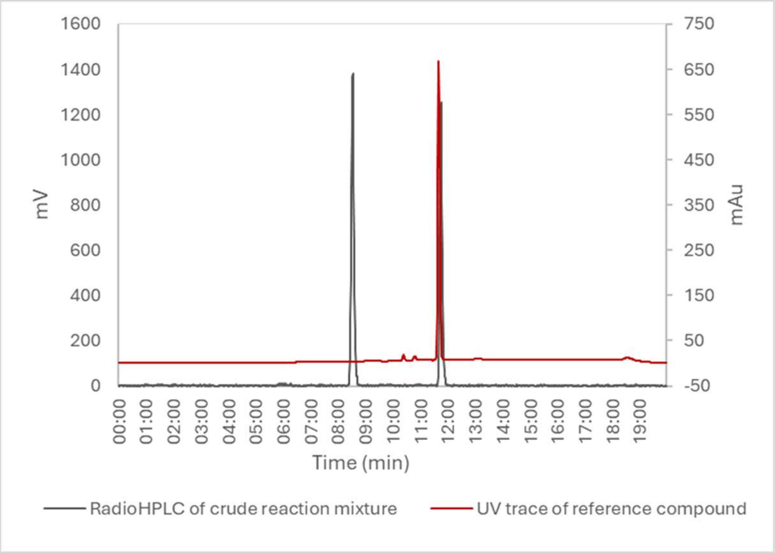

Entry	RCC (%)
**1**	46
**2**	44
**3**	40
**Average**	43 ± 3

[¹⁸F]5-(3-chloro-4-methoxyphenyl)-*N*-(3-(2-fluorophenyl)propyl)oxazole-4-carboxamide ([¹⁸F]42)

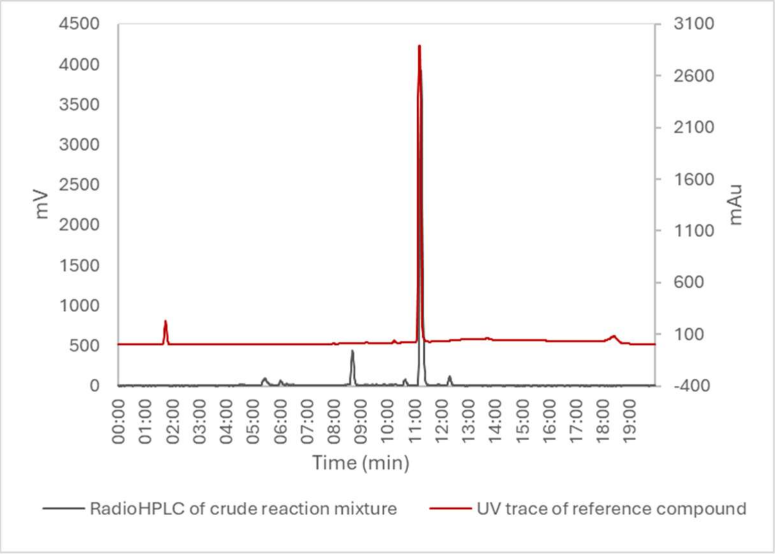

Entry	RCC (%)
**1**	31
**2**	30
**Average**	31 ± 1

Semi-preparative HPLC Purified [^{18}F]2-fluoro-5-iodopyridine ([^{18}F]34) Quality check

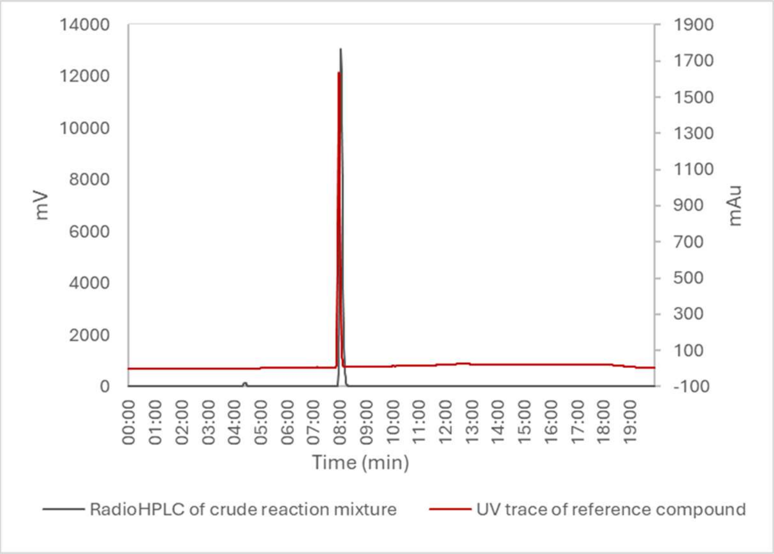

[^{18}F]2-fluoro-5-phenethylpyridine ([^{18}F]S-14)

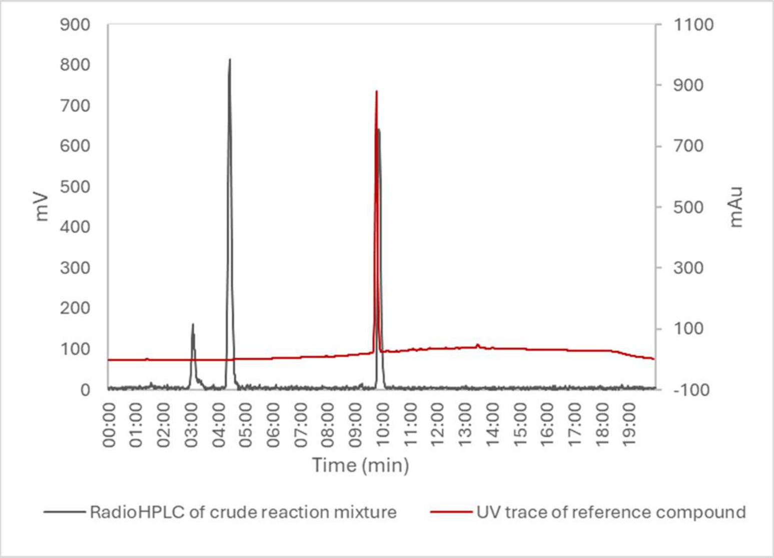

Entry	RCC (%)
**1**	31
**2**	36
**3**	33
**Average**	33 ± 2

[^{18}F]5-(3-chloro-4-methoxyphenyl)-*N*-(3-(6-fluoropyridin-3-yl)propyl)oxazole-4-carboxamide ([^{18}F]OCM-50)

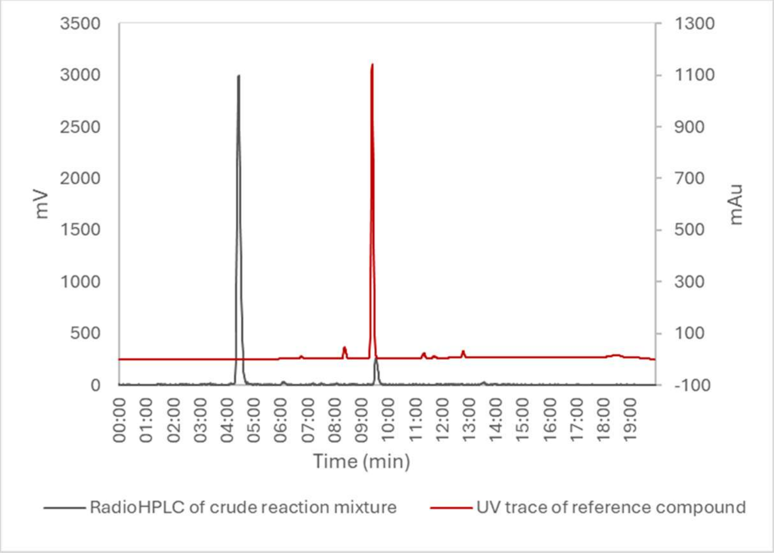

Entry	RCC (%)
**1**	7
**2**	8
**Average**	8 ± 1

Semi-preparative HPLC Purified [^{18}F]1-iodo-4-(trifluoromethyl)benzene ([^{18}F]35) Quality Check

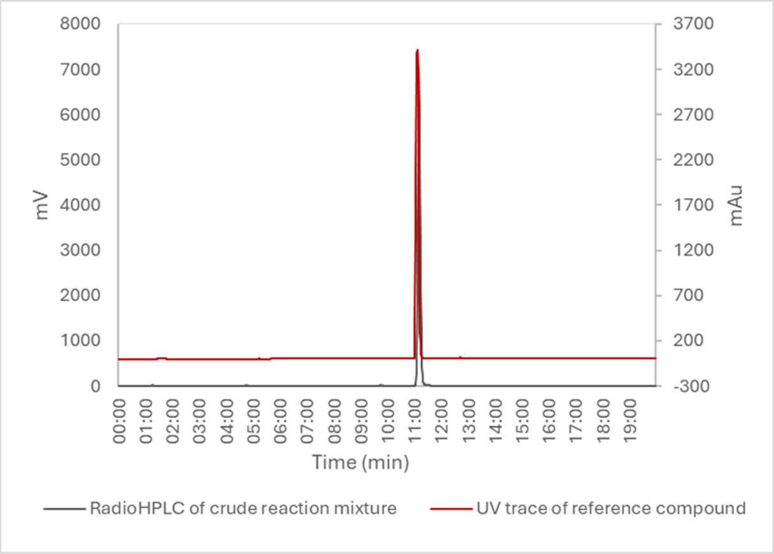

[^{18}F]1-phenethyl-4-(trifluoromethyl)benzene ([^{18}F]S-15)

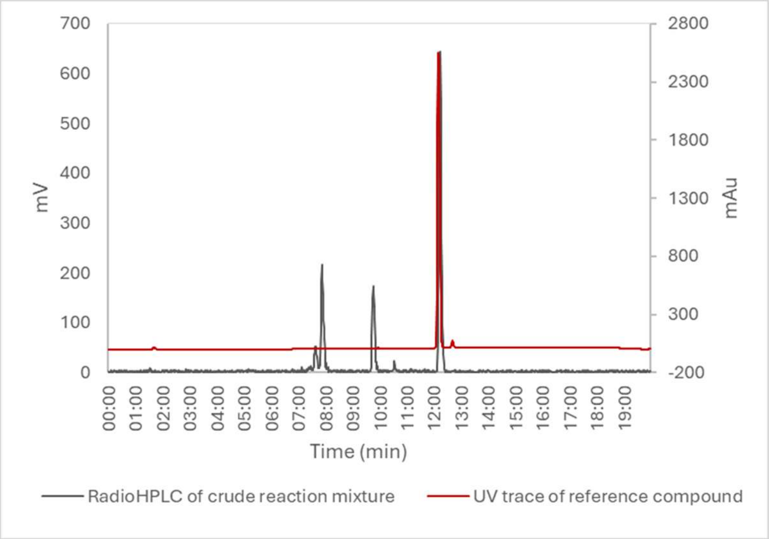

Entry	RCC (%)
**1**	52
**2**	55
**3**	54
**Average**	54 ± 1

[^{18}F]5-(3-chloro-4-methoxyphenyl)-*N*-(3-(4-(trifluoromethyl)phenyl)propyl)oxazole-4-carboxamide ([^{18}F]39)

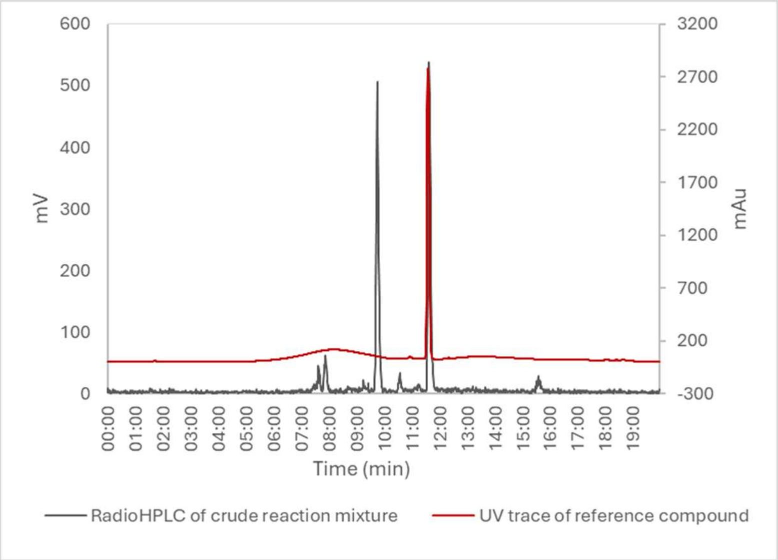

Entry	RCC (%)
**1**	51
**2**	43
**Average**	47 ± 4

Semi-preparative HPLC Purified [^{18}F]1-(2-fluoroethoxy)-4-iodobenzene ([^{18}F]36) Quality Check

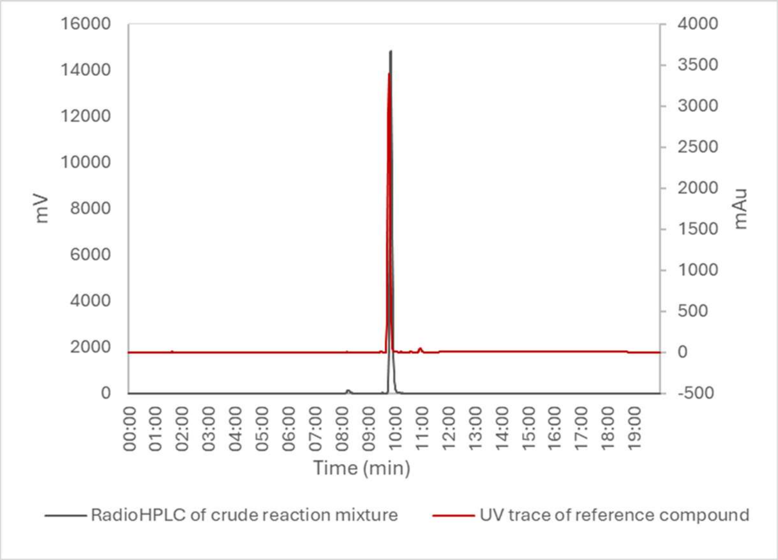

[^{18}F]1-(2-fluoroethoxy)-4-phenethylbenzene ([^{18}F]S-16)

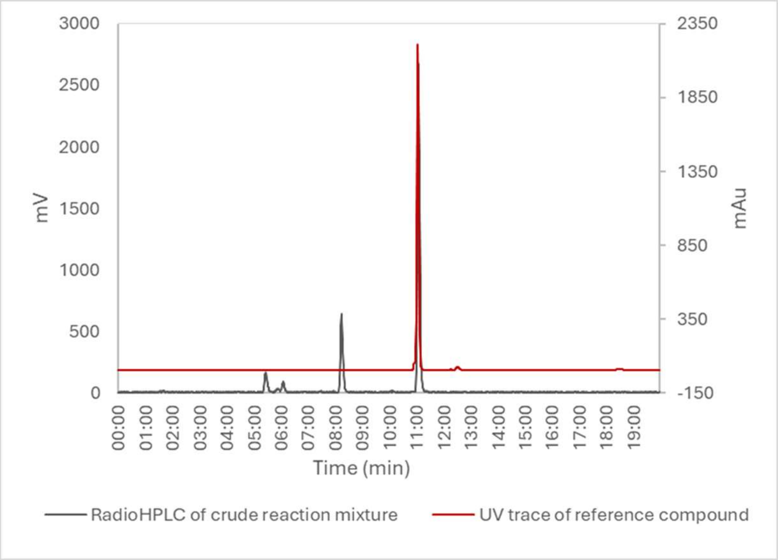

Entry	RCC (%)
**1**	68
**2**	72
**3**	69
**Average**	70 ± 2

[^{18}F]5-(3-chloro-4-methoxyphenyl)-*N*-(3-(4-(2-fluoroethoxy))propyl)oxazole-4-carboxamide ([^{18}F]40)

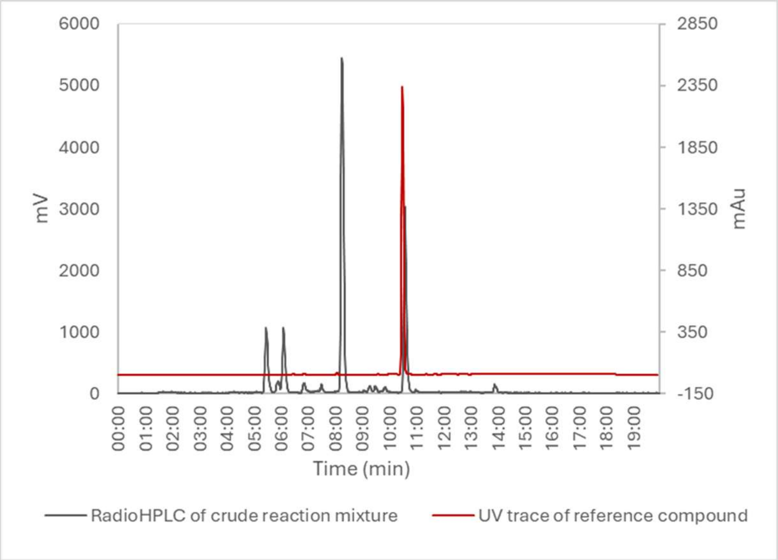

Entry	RCC (%)
**1**	20
**2**	27
**Average**	24 ± 4

Semi-preparative HPLC Purified [^{18}F]1-chloro-3-fluoro-5-iodobenzene ([^{18}F]38) Quality Check

Analytical HPLC Condition E

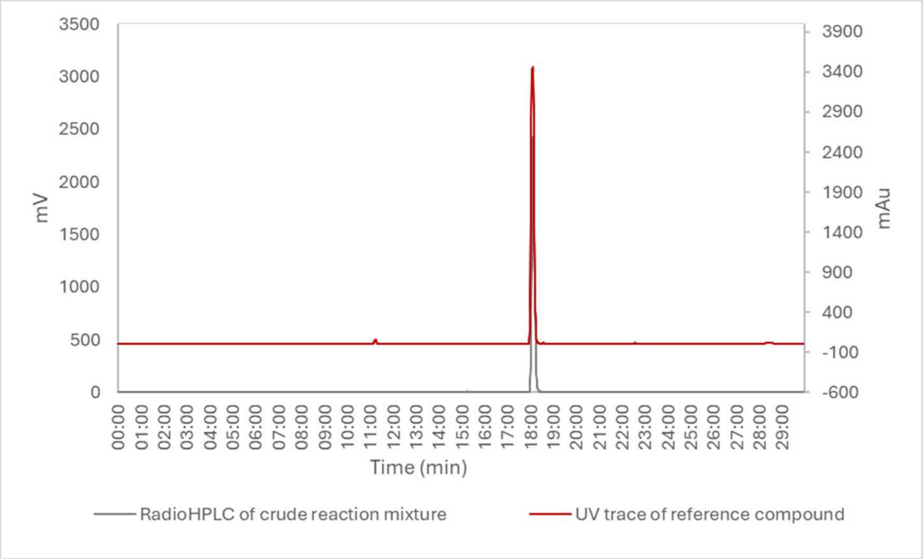

[^{18}F]5-(3-chloro-4-methoxyphenyl)-*N*-(3-(3-chloro-5-fluorophenyl)propyl)oxazole-4-carboxamide ([^{18}F]43)

Analytical HPLC Condition E

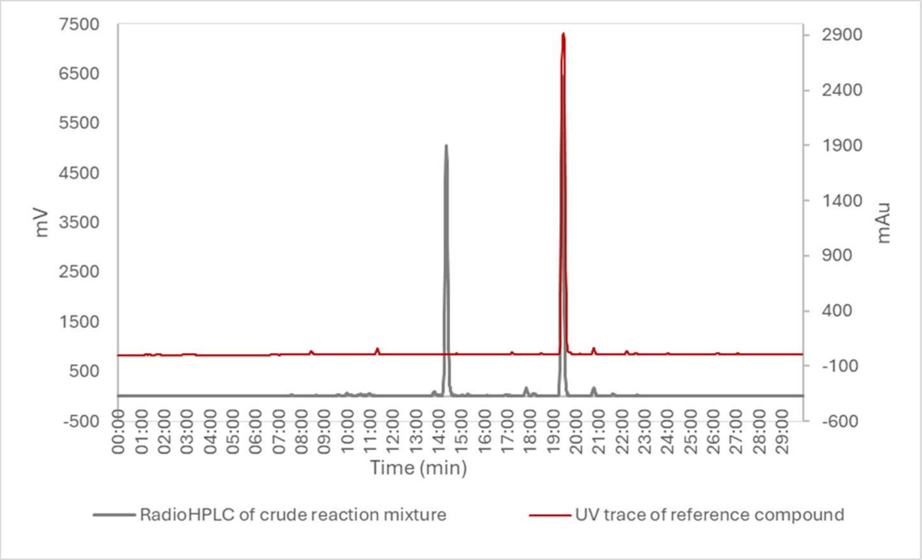

Entry	RCC (%)
**1**	54
**2**	34
**Average**	44 ± 10

2.1 General Information for Synthesis

Unless stated otherwise all chemicals were used as received. Extra dry solvents (99.9% with Acroseal) [*N*-methyl pyrrolidine (NMP); Ethanol (EtOH); 1,3-Dimethyl-2-imidazolidinone (DMI); *N,N*-Dimethylacetamide (DMA); Tetrahydrofuran (THF); *N,N*-Dimethylformamide (DMF); Dimethyl sulfoxide (DMSO); Acetonitrile (MeCN)] were purchased from Thermo-Scientific and an inert atmosphere maintained using a balloon of N_2 gas before withdrawing from the bottle.

Chemicals were purchased from Acros, Alfa Aesar, Doug Discovery (Fluorochem), BLD Pharma, Combi-Blocks, or Sigma Aldrich. Zinc (powder, <150 µm, >99% trace metals basis) was activated prior to use by stirring in 1M HCl (aq.) for 1 hour and then filtered and washed with H_2O (20 mL), EtOH (20 mL), and Et_2O (2 x 20 mL) and subsequently dried on high vacuum for 12-16 h. Tetrakis(dimethylamino)ethylene (TDAE) was stored in an N_2-filled Schlenk flask. Manganese, powder, − 325 mesh, ≥99% trace metals basis was purchased from Sigma Aldrich or Alfa Aesar and stored under ambient conditions. $NiBr_2$.glyme was purchased from Sigma Aldrich and stored under argon in a Schlenk flask for the synthesis of reference compounds. When $NiBr_2$.glyme was required for use in the radiochemistry facility, the desired amount was decanted from the Schlenk flask into a vial and stored under ambient conditions.

Reactions were monitored by thin-layer chromatography (TLC) on silica gel pre-coated aluminium sheets (Merck Kieselgel 60 F254 plates). Visualization was accomplished by irradiation with UV light (254 nm or 366 nm) or staining with ninhydrin, or cerium ammonium molybdate stains. Column chromatography was performed using Merck silica gel (60, particle size 0.040-0.063 mm) with solvent specified or was performed over pre-packed RediSep® silica gel eluted with *n*-heptane:ethyl acetate (EA) or Methanol/dichloromethane (DCM) mixtures using a CombiFlash Rf 200 automatic flash chromatography system.

All NMR spectra were recorded on Bruker AVIIIHD400, AVIIIHD500, NEO600, or DPX-400 MHz spectrometers. NMR spectra were processed using MestReNova (Version 14.3.2). Unless otherwise specified, NMR spectra were acquired at 298K. ^{1}H and ^{13}C NMR spectra are reported as chemical shifts (δ) in parts per million (ppm) relative to the solvent peak or to tetramethylsilane (TMS) if $CDCl_3$ + 0.03 % TMS was used. ^{19}F NMR spectra are referenced relative to $CFCl_3$ in the corresponding solvent. Coupling constants (J) are reported in units of hertz (Hz). The following abbreviations are used to describe multiplicities; s (singlet), d (doublet), t (triplet), q (quartet), m (multiplet), br. (broad).

High resolution mass spectra (HRMS) were recorded using a Thermo Exactive High-Resolution Orbitrap FT-MS or a Waters RDa bench-top TOF spectrometer used with an Acquity LC system in direct infusion (loop injection) mode for electrospray ionization (ESI), or an Agilent 7200 Accurate Mass Q-TOF GC-MS connected to a 7890 GC system for electron impact ionization (GC-EI), with the mode specified in all cases. After trying all available MS ionization methods, some compounds were found to not be readily ionizable and therefore no HRMS data obtained.

Infrared spectra were recorded as the neat compound (neat) or as an evaporated solution (thin layer film) using a Bruker Tensor 27 FTIR spectrometer. Absorptions are reported in wavenumber (cm^{-1}). Melting points of solids were measured on a Griffin apparatus and are uncorrected.

2.2 Preparation of Alkyl 2,4,6-Triphenylpyridinium Salts

The following compounds were prepared as described previously in literature reports (Figure S11):

1-phenethyl-2,4,6-triphenylpyridin-1-ium Tetrafluoroborate[21] (**2**)

2,4,6-triphenyl-1-(tetrahydro-*2H*-pyran-4-yl)pyridin-1-ium Tetrafluoroborate[19] (**S-1**)

2,4,6-triphenyl-1-(pyridin-2-ylmethyl)pyridin-1-ium Tetrafluoroborate[18] (**S-3**)

1-(((1R,4aS,10aR)-7-isopropyl-1,4a-dimethyl-1,2,3,4,4a,9,10,10a-octahydrophenanthren-1-yl)methyl)-2,4,6-triphenylpyridin-1-ium Tetrafluoroborate[25] (**S-7**)

(*S*)-1-(5-((tert-butoxycarbonyl)amino)-6-methoxy-6-oxohexyl)-2,4,6-triphenylpyridin-1-ium Tetrafluoroborate[25] (**S-18**)

1-(2-(*1H*-indol-3-yl)ethyl)-2,4,6-triphenylpyridin-1-ium Tetrafluoroborate[25] (**S-19**)

2,4,6-triphenyl-1-(2-(thiophen-2-yl)ethyl)pyridin-1-ium Tetrafluoroborate[25] (**S-20**)

1-(2-(cyclohex-1-en-1-yl)ethyl)-2,4,6-triphenylpyridin-1-ium Tetrafluoroborate[25] (**S-21**)

1-(2-(4-(*tert*-butoxycarbonyl)piperazin-1-yl)ethyl)-2,4,6-triphenylpyridin-1-ium Tetrafluoroborate[21] (**S-22**)

1-(3-hydroxypropyl)-2,4,6-triphenylpyridin-1-ium Tetrafluoroborate[18] (**S-23**)

1-(2-((4-(2-chlorophenyl)-3,5-bis(ethoxycarbonyl)-6-methyl-1,4-dihydropyridin-2-yl)methoxy)ethyl)-2,4,6-triphenylpyridin-1-ium Tetrafluoroborate[18] (**S-24**)

1-(3-((6-methoxyquinolin-8-yl)amino)butyl)-2,4,6-triphenylpyridin-1-ium Tetrafluoroborate[19] (**S-25**)

(*E*)-1-(2-(((5-methoxy-1-(4-(trifluoromethyl)phenyl)pentylidene)amino)oxy)ethyl)-2,4,6-triphenylpyridin-1-iumTetrafluoroborate[49] (**S-26**)

2,4,6-triphenyl-1-(2-(pyridin-3-yl)ethyl)pyridin-1-ium Tetrafluoroborate[50] (**S-27**)

1-((1-(tert-butoxycarbonyl)azetidin-3-yl)methyl)-2,4,6-triphenylpyridin-1-ium Tetrafluoroborate[21] (**S-28**)

1-(2-((*4R,6R*)-6-(2-(tert-butoxy)-2-oxoethyl)-2,2-dimethyl-1,3-dioxan-4-yl)ethyl)-2,4,6-triphenylpyridin-1-ium Tetrafluoroborate[21] (**S-29**)

1-(((*1S,2R,5S*)-6,6-dimethylbicyclo[3.1.1]heptan-2-yl)methyl)-2,4,6-triphenylpyridin-1-ium Tetrafluoroborate[50] (**S-30**)

1-(2-(benzo[*d*][1,3]dioxol-5-yl)ethyl)-2,4,6-triphenylpyridin-1-ium Tetrafluoroborate[21] (**S-31**)

1-(1-(*tert*-butoxycarbonyl)piperidin-4-yl)-2,4,6-triphenylpyridin-1-ium Tetrafluoroborate[19] (**S-32**)

2,4,6-triphenyl-1-(4-phenylbutan-2-yl)pyridin-1-ium Tetrafluoroborate[19] (**S-33**)

Figure S11: Structures of 2,4,6-triphenykpyridinium salts prepared as described previously in literature reports

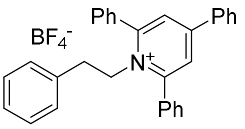

2

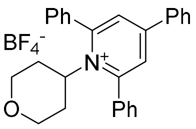

S-1

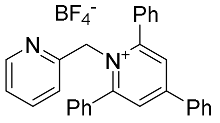

S-3

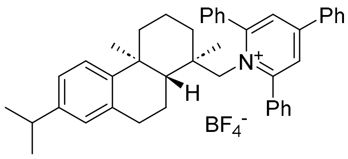

S-7

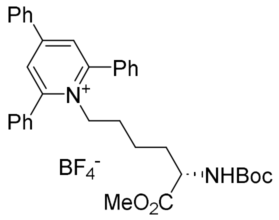

S-21

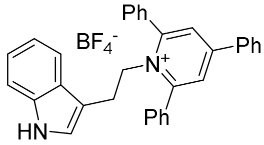

S-20

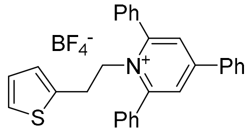

S-19

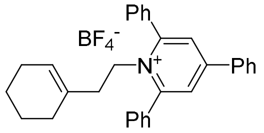

S-18

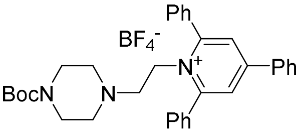

S-22

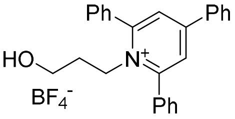

S-23

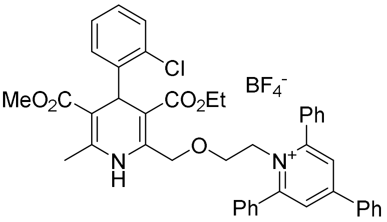

S-24

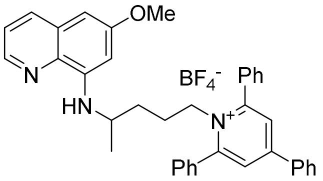

S-25

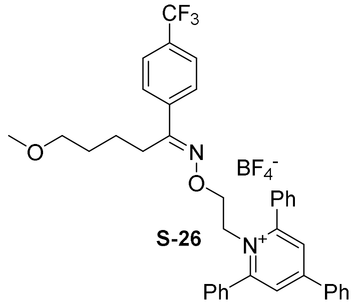

S-26

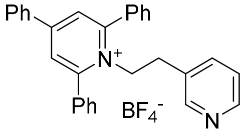

S-27

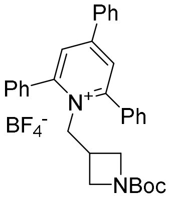

S-28

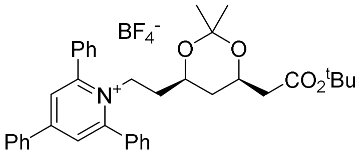

S-29

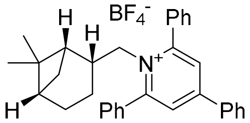

S-30

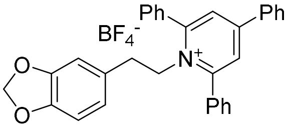

S-31

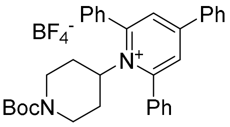

S-32

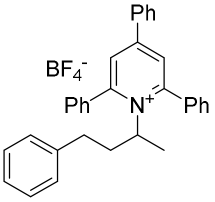

S-33

2.2.1 General Procedure A: Preparation of Alkyl 2,4,6-Triphenylpyridinium Salts

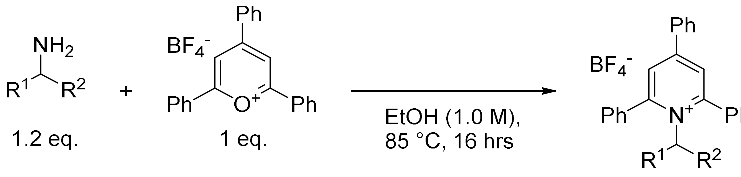

Adapted from Watson and co-workers.[18]

The amine (1.2 eq.) was added to a suspension of 2,4,6-triphenylpyrylium tetrafluoroborate (1.0 eq.) and EtOH (1.0 M) in an aluminium foil-covered Schlenk flask fitted with a reflux condenser. The mixture was heated at reflux in an oil bath at 85 °C for 16 h. The mixture was cooled to room temperature and diluted with Et_2O (2–3x volume of EtOH used). If product precipitation occurred at this point, the solid was filtered, washed with Et_2O (3 x 25 mL) and hexane (3 x 25 mL), and dried under high vacuum.

If the product failed to precipitate at this point and sonication in Et_2O resulted in the formation of a gel, then Et_2O (10 mL) was decanted over a fritted filter and this process of sonicating and decanting repeated with Et_2O (2 x 10 mL) followed by hexane (2 x 10mL). The Et_2O/hexane filtrates were discarded and the remaining solids dissolved in MeCN and washed through the same frit. Co-evaporation under reduced pressure of the MeCN filtrate with hexane to dryness typically resulted in a foam at this point which was sonicated in hexane until a pulverized solid was obtained. This was then filtered and washed as above and dried under high vacuum.

Should impurities be present at this point the solid was dissolved in a minimum amount of MeCN and triturated with Et_2O and sonicated followed by filtering, washing, and drying under vacuum as above. Additional purification required column chromatography eluting with DCM:acetone, elution conditions specified for each compound.

The corresponding amine hydrochloride salts can also be used following a modified procedure: Et_3N (1.2 eq.) was added to a mixture of the corresponding alkyl ammonium hydrochloride salt (1.2 eq.) and EtOH (1.0 M). After stirring the mixture for 30 min at room temperature, 2,4,6-triphenylpyrylium tetrafluoroborate (1 eq.) was added. From this point forward, the same procedure was followed as for alkyl amines described above; however, prior to washing the solid product with EtOH and/or Et_2O, the mixture was washed with H_2O (3 x 25 mL) to remove $Et_3N{\cdot}HCl$.

2.2.2 General Procedure B: Room Temperature Preparation of Alkyl Pyridinium Salts

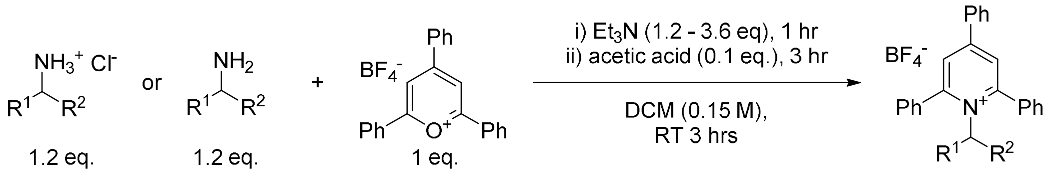

Adapted from Katritzky et. al.[24]

Performed under ambient conditions. To a suspension of the 2,4,6-triphenylpyrylium tetrafluoroborate (1 eq.) in DCM (0.15 M) were added dropwise a mixture of amine or amine hydrochloride (1.2 eq.) and triethylamine (1.2 eq. for amine, 2.4 eq. for hydrochloride salt, 3.6 eq. for dihydrochloride salt). If specified, 4 Å powdered molecular sieves (50 mg per 1 mmol) were added. The resulting solution was stirred for 1 hour at room temperature. After this time, acetic acid (0.1 eq.) was added and stirred at room temperature for 3 h.

A solid was precipitated from the reaction mixture using Et_2O (10 volumes) and filtered over celite. The Et_2O filtrate was discarded. The resulting gel-like residue at bottom of flask was dissolved in DCM and poured through the same celite frit. The DCM filtrate was concentrated and washed with H_2O (1 x 3 mL), followed by brine (2 x 3 mL). The organic phase was dried over Na_2SO_4, filtered, and the solvent removed. Should further purification be required at this stage, the mixture was purified by column chromatography [DCM:acetone].

2.2.3 General Procedure C: Preparation of Alkyl Pyridinium Salts from Boc-Protected Amines

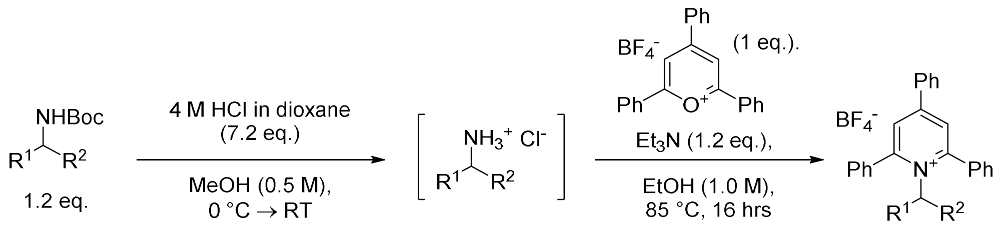

Boc-protected primary alkyl amine (1.2 eq.) was dissolved in MeOH (0.5M) and cooled to 0 °C in a one-necked round bottom flask under ambient atmosphere. 4 M HCl in dioxane (7.2 eq.) was added. After 15 minutes, the cooling bath was removed, and the mixture was stirred at room temperature until fully deprotected (consumption of starting material monitored by TLC). After this time, the solvent was removed under reduced pressure and the resulting hydrochloride salt carried forward to the next step with no further purification.

Under ambient atmosphere, to the hydrochloride salt in the same flask was added anhydrous EtOH (1 M) added followed by 2,4,6-triphenylpyrylium tetrafluoroborate (1 eq.). Anhydrous triethylamine (2.4 eq.) was then added dropwise to the mixture and the flask fitted with a reflux condenser and heated to 85 °C for 15 h.

After this time, the reaction mixture was cooled to room temperature and excess Et_2O (~10 volumes) was added. The suspension was filtered over a pad of celite. The resulting residue was dissolved in a minimum amount of DCM (1 to 5 mL) and excess Et_2O was added (~5 volumes) and decanted over the same frit. This process was repeated until no residue remained in the flask (3 or 4 times). The celite pad was washed with Et_2O (10 mL) and hexane (10 mL) and the Et_2O/ hexane filtrate discarded. The solids remaining on the celite pad were dissolved in DCM and the filtrate was concentrated under reduced pressure followed by purification by column chromatography.

2.2.4 Characterisation Data for Alkyl 2,4,6-Triphenylpyridinium Salts

1-(2-(4-Ethyl-1*H*-1,2,3-triazol-1-yl)ethyl)-2,4,6-triphenylpyridin-1-ium Tetrafluoroborate (S-34)

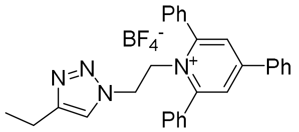

Prepared according to **General Procedure A** on a 1.49 mmol scale from 2-(4-Ethyl-*1H*-1,2,3-triazol-1-yl)ethylamine. After diluting the reaction mixture with Et_2O (10 mL) and sonication, the solid was filtered and washed with Et_2O (2 x 25 mL) and hexane (2 x 25 mL) and then dried on high vacuum resulting in the title compound as an off-white powder (732 mg, 1.41 mmol, 95% yield).

^{1}H NMR (400 MHz, DMSO-*d*$_6$) δ 8.47 (s, 2H), 8.29 (d, *J* = 7.0 Hz, 2H), 7.76 – 7.58 (m, 13H), 7.30 (s, 1H), 5.02 – 4.95 (m, 2H), 4.26 – 4.18 (m, 2H), 2.56 (q, *J* = 7.6 Hz, 2H), 1.12 (t, *J* = 7.6 Hz, 3H).

^{13}C NMR (101 MHz, DMSO-*d*$_6$) δ 156.7, 155.0, 148.8, 132.9, 132.7, 132.6, 131.2, 129.6, 129.4, 129.2, 128.9, 126.0, 122.0, 54.5, 47.2, 18.3, 13.5.

^{19}F NMR (470 MHz, DMSO-*d*$_6$) δ -148.3 (BF_4^- minor), -148.3 (BF_4^- major).

FTIR (thin layer film) ν (cm^{-1}) = 3597, 2926, 2367, 1728, 1623, 1062, 767.

m.p. 119 – 123 °C

HRMS: (ESI+) *m/z* calculated for $C_{29}H_{27}N_4$ requires *m/z* 431.2230 ([M-BF_4^-]$^+$). Found *m/z* 431.2236.

1-((*S*)-6-(*tert*-Butoxy)-5-(3-((*S*)-1,5-di-tert-butoxy-1,5-dioxopentan-2-yl)ureido)-6-oxohexyl)-2,4,6-triphenylpyridin-1-ium Tetrafluoroborate (S-35)

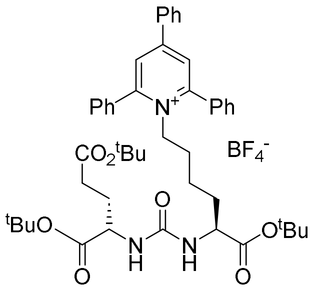

Prepared according to **General Procedure A** on a 0.40 mmol scale. Once the reaction was complete, the reaction solvent was removed under reduced pressure and co-evaporated with hexane (30 mL x3). The resulting solid was sonicated in Et_2O (30 mL) and decanted over a fritted filter under vacuum; this was repeated three times. The Et_2O filtrate was discarded and the same frit was washed with DCM (20 mL) which was combined with the solid residue in the flask and purified by column chromatography [DCM → 20:80 Acetone:DCM] resulting in a white foam (326 mg, 0.38 mmol, 95% yield).

^{1}H NMR (500 MHz, DMSO-*d*$_6$) δ 8.47 (s, 2H), 8.25 (dd, *J* = 7.0, 1.8 Hz, 2H), 7.87 – 7.81 (m, 4H), 7.72 – 7.67 (m, 6H), 7.67 – 7.64 (m, 1H), 7.61 (t, *J* = 7.4 Hz, 2H), 6.19 (d, *J* = 8.4 Hz, 1H), 6.05 (d, *J* = 8.3 Hz, 1H), 5.75 (s, 1H), 4.29 (dq, *J* = 18.2, 7.3 Hz, 2H), 3.99 (td, *J* = 8.6, 5.2 Hz, 1H), 3.70 (td, *J* = 8.1,

5.3 Hz, 1H), 2.25 – 2.12 (m, 2H), 1.89 – 1.79 (m, 1H), 1.68 – 1.58 (m, 1H), 1.38 (s, 18H), 1.31 (s, 9H), 1.14 (s, 1H), 1.11 – 1.01 (m, 1H), 0.99 – 0.89 (m, 1H), 0.80 – 0.66 (m, 2H).

^{13}C NMR (126 MHz, DMSO-*d*$_6$) δ 171.9, 171.7, 171.4, 156.9, 155.9, 154.2, 133.1, 132.9, 132.4, 130.9, 129.6, 129.2, 129.0, 128.7, 126.1, 80.6, 80.4, 79.7, 54.1, 52.5, 52.1, 30.8, 30.6, 29.6, 28.4, 27.7, 27.6, 27.6, 21.6.

^{19}F NMR (470 MHz, DMSO-*d*$_6$) δ -148.3 (BF_4^- minor), -148.3 (BF_4^- major).

FTIR (thin film) ν (cm^{-1}) = 2932, 1732, 1562, 1257, 1158, 1068, 764.

m.p. 99 – 103 °C

HRMS: (ESI+) *m/z* calculated for $C_{47}H_{60}N_3O_7$ requires *m/z* 778.4426 ([M-BF_4^-]$^+$). Found *m/z* 778.4449**.**

2,4,6-Triphenyl-1-(piperidin-2-ylmethyl)pyridin-1-ium Tetrafluoroborate (S-36)

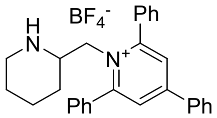

Prepared using **General Procedure A** on a 1 mmol scale from piperidin-2-ylmethanamine. After diluting the reaction mixture with Et_2O (10 mL) and sonication, the solid was filtered and washed with Et_2O (2 x 25 mL) and hexane (2 x 25 mL) and then dried on high vacuum resulting in the title compound as a yellow solid (408 mg, 0.83 mmol, 83% yield).

^{1}H NMR (400 MHz, CD_3CN) δ 8.18 (s, 2H), 8.05 – 7.96 (m, 2H), 7.84 – 7.73 (m, 4H), 7.73 – 7.57 (m, 9H), 4.56 (dd, *J* = 14.2, 4.6 Hz, 1H), 4.44 (dd, *J* = 14.2, 8.8 Hz, 1H), 2.69 – 2.60 (m, 1H), 2.42 – 2.31 (m, 1H), 2.20 – 2.09 (m, 1H), 1.53 – 1.42 (m, 1H), 1.36 – 1.24 (m, 1H), 1.07 – 0.86 (m, 3H), 0.64 – 0.50 (m, 1H).

^{13}C NMR (101 MHz, CD_3CN) δ 158.3, 156.4, 134.7, 134.5, 133.5, 132.1, 130.7, 130.6, 130.3, 129.4, 127.6, 60.6, 57.4, 46.9, 30.9, 26.8, 24.7.

^{19}F NMR (376 MHz, CD_3CN) δ -152.0 (BF_4^- minor), -152.1 (BF_4^- major).

FTIR (thin film) ν (cm^{-1}) = 3036, 2937, 1621, 1055, 761, 702.

m.p. 168 – 170 °C

HRMS: (ESI+) *m/z* calculated for [$C_{29}H_{29}N_2$] requires *m/z* 405.2325 [M-BF_4^-]$^+$. Found *m/z* 405.2338.

1-(2-(1-Hydroxycyclohexyl)-2-(4-methoxyphenyl)ethyl)-2,4,6-triphenylpyridin-1-ium Tetrafluoroborate (S-37)

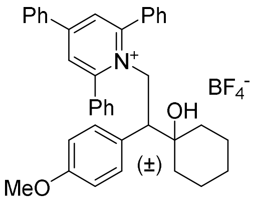

Prepared using **General Procedure A** on a 2 mmol scale from 1-[2-amino-1-(4-methoxyphenyl)ethyl]cyclohexanol hydrochloride. After diluting the reaction mixture with Et_2O (10 mL) and sonication, a gel remained at the bottom of the flask. The supernatant Et_2O was decanted and discarded and the residue concentrated under reduced pressure. Purification by column chromatography [DCM → 7:3 DCM:Acetone] resulted in the title compound as a yellow foam (1.16g, 1.84 mmol, 92% yield).

^{1}H NMR (600 MHz, DMSO-d_6, T = 373K) δ 8.16 (br. s, 2H), 8.09 – 8.04 (m, 2H), 7.87 (br. s, 4H), 7.72 (dd, J = 5.2, 2.9 Hz, 6H), 7.64 (t, J = 7.3 Hz, 1H), 7.59 (t, J = 7.5 Hz, 2H), 6.68 – 6.60 (m, 4H), 5.33 (dd, J = 14.3, 4.3 Hz, 1H), 5.10 (dd, J = 14.3, 11.0 Hz, 1H), 3.70 (s, 3H), 2.51 (dd, J = 6.7, 2.4 Hz, 1H – *signal overlaps with DMSO*), 1.30 – 1.12 (m, 4H), 1.00 – 0.88 (m, 2H), 0.83 – 0.75 (m, 2H), 0.69 (ddd, J = 13.6, 9.8, 3.8 Hz, 1H), 0.54 (ddd, J = 13.5, 9.9, 3.9 Hz, 1H).

^{13}C NMR (151 MHz, DMSO-d_6, T = 373K) δ 158.3, 157.3, 154.2, 133.1, 132.8, 131.8, 130.8, 129.9, 129.7, 129.0, 128.7, 128.0, 127.9, 125.4, 113.2, 71.3, 57.9, 54.8, 52.5, 35.5, 35.4, 24.4, 20.9, 20.6.

^{19}F NMR (376 MHz, DMSO-d_6) δ -148.3 (BF_4^- minor), -148.3 (BF_4^- major).

FTIR (thin film) ν (cm^{-1}) = 3529, 3035, 2938, 1619, 1250, 1059, 758.

m.p. 133 – 136 °C

HRMS: (ESI+) *m/z* calculated for $C_{38}H_{38}NO_2$ requires *m/z* 540.2897 (M-BF_4^-]$^+$). Found *m/z* 540.2904.

2,4,6-Triphenyl-1-(2-(pyrrolidin-1-yl)ethyl)pyridin-1-ium tetrafluoroborate (S-38)

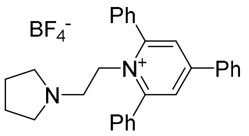

Prepared using **General Procedure A** on a 2 mmol scale from 2-(pyrrolidin-1-yl)ethan-1-amine. Purification by column chromatography [DCM → 6:4 DCM:Acetone] resulted in the title compound as a yellow solid (300 mg, 0.61 mmol, 30% yield).

^{1}H NMR (400 MHz, CD_3CN) δ 8.18 (s, 2H), 8.04 – 7.97 (m, 2H), 7.79 – 7.73 (m, 4H), 7.72 – 7.56 (m, 9H), 4.55 (t, J = 6.9 Hz, 2H), 2.40 (t, J = 6.9 Hz, 2H), 1.92 – 1.87 (m, 4H), 1.53 – 1.45 (m, 4H).

^{13}C NMR (101 MHz, CD_3CN) δ 158.0, 156.3, 134.6, 134.2, 133.5, 132.1, 130.7, 130.3, 130.2, 129.4, 127.5, 55.1, 54.7, 54.3, 24.1.

^{19}F NMR (376 MHz, CD_3CN) δ -151.8 (BF_4^- minor), -151.8 (BF_4^- major).

FTIR (thin film) ν (cm^{-1}) = 3032, 1624, 1055, 765, 701, 667.

m.p. 159 – 162 °C

HRMS: (ESI+) *m/z* calculated for $C_{29}H_{29}N_2$ requires *m/z* 405.2325 $[M\text{-}BF_4^-]^+$. Found *m/z* 405.2338.

Scheme S10: Multistep synthesis of 1-(2-((4,6-dimethoxypyrimidin-2-yl)amino)ethyl)-2,4,6-triphenylpyridin-1-ium Tetrafluoroborate (**S-40**)

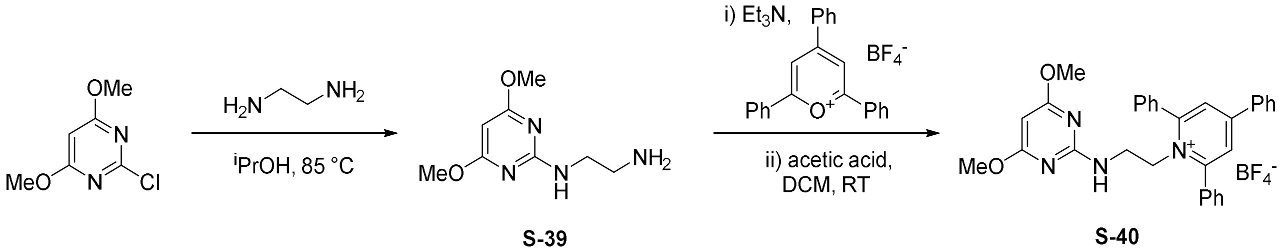

N^1-(4,6-Dimethoxypyrimidin-2-yl)ethane-1,2-diamine (S-39)

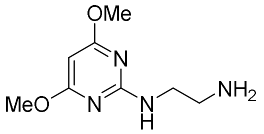

Prepared as previously described in literature.[51]

1-(2-((4,6-Dimethoxypyrimidin-2-yl)amino)ethyl)-2,4,6-triphenylpyridin-1-ium tetrafluoroborate (S-40)

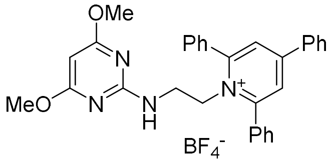

Prepared using **General Procedure B** on a 1.05 mmol scale from N^1-(4,6-dimethoxypyrimidin-2-yl)ethane-1,2-diamine (**S-39**). Purification by column chromatography [DCM → 6:4 DCM:Acetone] resulted in the title compound as a yellow foam (300 mg, 0.61 mmol, 30% yield).

^{1}H NMR (400 MHz, $CDCl_3$) δ 7.92 (s, 2H), 7.86 – 7.70 (m, 6H), 7.62 – 7.45 (m, 9H), 6.07 (br. s, 1H), 5.31 (s, 1H), 5.12 (s, 2H), 3.88 (br. s, 3H), 3.54 (br. s, 3H), 3.34 (s, 2H).

^{13}C NMR (101 MHz, $CDCl_3$) δ 171.7, 161.2, 157.5, 156.2, 134.4, 133.2, 132.1, 130.8, 129.8, 129.6, 129.3, 128.3, 127.1, 79.3, 54.4, 53.7, 41.6.

^{19}F NMR (377 MHz, $CDCl_3$) δ -152.7 (BF_4^- minor), -152.8 (BF_4^- major).

FTIR (thin film) ν (cm^{-1}) = 1587, 1356, 1163, 1053, 754.

m.p. 120 – 126 °C

HRMS: (ESI+) *m/z* calculated for $C_{31}H_{29}N_4O_4$ requires *m/z* 489.2285 $[M\text{-}BF_4^-]^+$. Found *m/z* 489.2279.

1-(1-(Oxazol-2-yl)azetidin-3-yl)-2,4,6-triphenylpyridin-1-ium tetrafluoroborate (S-4)

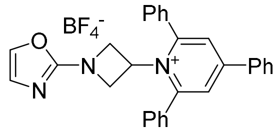

Prepared using **General Procedure B** on a 2.52 mmol scale from 1-(1,3-oxazol-2-yl)azetidin-3-amine dihydrochloride. Purification by column chromatography [DCM → 6:4 DCM:Acetone] resulted in the title compound as a yellow foam (550 mg, 1.31 mmol, 52% yield).

^{1}H NMR (500 MHz, $CDCl_3$) δ 7.95 (dd, *J* = 7.4, 1.9 Hz, 4H), 7.88 (s, 2H), 7.83 – 7.76 (m, 2H), 7.64 – 7.49 (m, 9H), 7.02 (s, 1H), 6.55 (s, 1H), 6.25 (p, *J* = 7.4 Hz, 1H), 3.69 (t, *J* = 8.4 Hz, 2H), 3.13 – 3.06 (m, 2H).

^{13}C NMR (126 MHz, $CDCl_3$) δ 161.2, 157.1, 156.7, 134.1, 133.8, 132.6, 132.6, 132.3, 130.1, 130.0, 129.6, 128.3, 126.6, 126.5, 58.5, 57.2.

^{19}F NMR (470 MHz, $CDCl_3$) δ -152.3 (BF_4^- minor), -152.4 (BF_4^- major).

FTIR (thin film) ν (cm^{-1}) = 1619, 1402, 1056, 751, 701.

m.p. 170 – 174 °C

HRMS: (ESI+) *m/z* calculated for $[C_{29}H_{24}N_3O]^+$ requires *m/z* 430.1914 ($[M-BF_4]^+$). Found *m/z* 430.1914.

1-((6-Chloropyridin-3-yl)methyl)-2,4,6-triphenylpyridin-1-ium tetrafluoroborate (S-2)

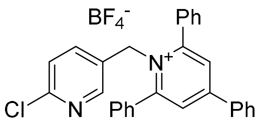

Prepared according to **General Procedure A** on a 2.5 mmol scale from 5-(aminomethyl)-2-chloropyridine. Purification by column chromatography [DCM → 40% acetone in DCM] resulted in a green foam (839 mg, 1.61 mmol, 64% yield).

^{1}H NMR (500 MHz, $CDCl_3$) δ 7.86 (s, 2H), 7.69 (dd, *J* = 20.0, 7.4 Hz, 6H), 7.55 – 7.41 (m, 9H), 7.34 (s, 1H), 7.11 (d, *J* = 8.1 Hz, 1H), 7.01 (d, *J* = 6.9 Hz, 1H), 5.76 (s, 2H).

^{13}C NMR (126 MHz, $CDCl_3$) δ 157.3, 157.0, 151.4, 147.6, 137.6, 133.8, 132.6, 132.6, 131.5, 129.8, 129.6, 129.2, 128.7, 128.3, 127.0, 124.8, 55.3.

^{19}F NMR (470 MHz, $CDCl_3$) δ -152.1 (BF_4^- minor), -152.2 (BF_4^- major).

FTIR (thin film) ν (cm^{-1}) = 1622, 1460, 1056, 756, 702.

m.p. 116 – 120 °C

HRMS: (ESI+) *m/z* calculated for $[C_{29}H_{22}ClN_2]^+$ requires *m/z* 433.1466 ($[M-BF_4]^+$). Found *m/z* 433.1467.

1-(2-(*1H*-Imidazol-5-yl)ethyl)-2,4,6-triphenylpyridin-1-ium tetrafluoroborate (S-9)

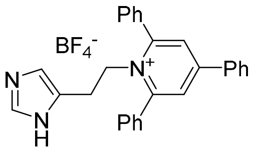

Prepared using **General Procedure A** on a 2.7 mmol scale from histamine. The solid was filtered, washed with Et_2O (3 x 25 mL) and hexane (3 x 25 mL), and dried under high vacuum resulting in the title compound as a white solid (1.085 g, 2.22 mmol, 82% yield).

^{1}H NMR (500 MHz, DMSO-d_6) δ 11.74 (s, 1H), 8.43 (s, 2H), 8.28 – 8.23 (m, 2H), 7.81 – 7.72 (m, 4H), 7.73 – 7.63 (m, 9H), 7.61 (dd, *J* = 8.3, 6.5 Hz, 2H), 7.36 (s, 1H), 6.39 (s, 1H), 4.63 (t, *J* = 7.4 Hz, 2H).

^{13}C NMR (126 MHz, DMSO-d_6) δ 156.4, 154.1, 135.1, 134.7, 133.2, 133.1, 132.4, 130.9, 129.6, 129.2, 129.0, 128.7, 125.9, 112.9, 55.0, 27.2.

^{19}F NMR (470 MHz, DMSO-d_6) δ -148.2 (BF_4^- minor), -148.3 (BF_4^- major).

FTIR (thin film) ν (cm^{-1}) = 1622, 1220, 1057, 770, 702.

m.p. 214 – 219 °C

HRMS: (ESI+) m/z calculated for $[C_{28}H_{24}N_3]^+$ requires m/z 402.1965 ($[M-BF_4^-]^+$). Found m/z 402.1968.

2,4,6-Triphenyl-1-(2-(2-(prop-2-yn-1-yloxy)ethoxy)ethyl)pyridin-1-ium tetrafluoroborate (S-6)

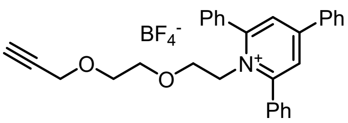

Prepared using **General Procedure A** on a 2 mmol scale from 2-(2-(prop-2-yn-1-yloxy)ethoxy)ethan-1-amine. The solid was filtered, washed with Et_2O (3 x 25 mL) and hexane (3 x 25 mL), and dried under high vacuum resulting in the title compound as a pale orange solid (666 mg, 1.28 mmol, 64% yield).

^{1}H NMR (400 MHz, $CDCl_3$) δ 7.88 (s, 2H), 7.87 – 7.81 (m, 4H), 7.81 – 7.77 (m, 2H), 7.62 – 7.57 (m, 6H), 7.56 – 7.50 (m, 3H), 4.85 (t, *J* = 5.6 Hz, 2H), 4.03 (d, *J* = 2.4 Hz, 2H), 3.47 – 3.40 (m, 2H), 3.32 (t, *J* = 5.5 Hz, 2H), 3.27 – 3.20 (m, 2H), 2.39 (t, *J* = 2.4 Hz, 1H).

^{13}C NMR (101 MHz, $CDCl_3$) δ 157.7, 155.9, 134.1, 133.2, 132.4, 131.2, 129.9, 129.7, 129.5, 128.2, 126.6, 79.4, 75.0, 70.3, 68.9, 67.9, 58.4, 54.1.

^{19}F NMR (377 MHz, $CDCl_3$) δ -153.0 (BF_4^- minor), -153.1 (BF_4^- major).

FTIR (thin film) ν (cm^{-1}) = 1622, 1566, 1217, 1056, 893. 752, 702, 667.

m.p. 114 – 115 °C

HRMS: (ESI+) *m/z* calculated for $[C_{30}H_{28}NO_2]^+$ requires *m/z* 434.2115 ($[M-BF_4^-]^+$). Found *m/z* 434.2117.

Scheme S11: Multistep synthesis of 1-(2-(2,3-bis(tert-butoxycarbonyl)guanidino)ethyl)-2,4,6-triphenylpyridin-1-ium tetrafluoroborate (**S-8**)

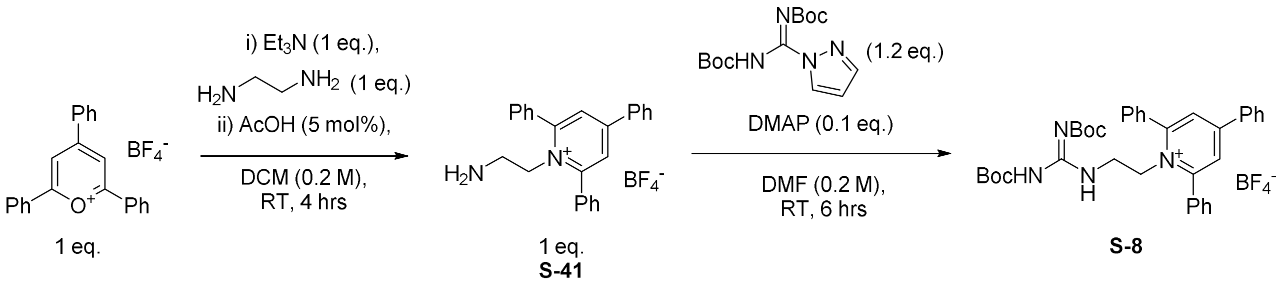

1-(2-Aminoethyl)-2,4,6-triphenylpyridin-1-ium tetrafluoroborate (S-41)

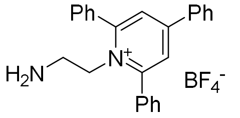

Prepared as previously described in literature.[24]

1-(2-(2,3-Bis(*tert*-butoxycarbonyl)guanidino)ethyl)-2,4,6-triphenylpyridin-1-ium tetrafluoroborate (S-8)

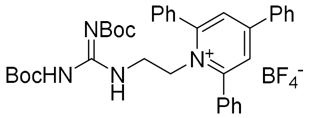

In a 2-neck flask under N_2, 1-(2-aminoethyl)-2,4,6-triphenylpyridin-1-ium tetrafluoroborate (88 mg, 0.2 mmol, 1 eq.) in DMF (1 mL) had DMAP (2.5 mg, 20 μmol, 0.1 eq.) added followed by *N,N'*-di-Boc-*1H*-pyrazole-1-carboxamidine (75 mg, 0.24 mmol, 1.2 eq.) and stirred for 6 h. After this time, the crude reaction mixture was decanted into a mixture of Et_2O:hexane (1:1, 600 mL) and a solid crashed out. The mixture was decanted over celite and the Et_2O/hexane filtrate discarded. The solids remaining on the celite filter were washed through with DCM and the solvent concentrated under reduced pressure. Purification by column chromatography [DCM → 70:30 DCM:acetone] resulted in the title compound as a white foam (52 mg, 76 μmol, 40% yield).

^{1}H NMR (500 MHz, $CDCl_3$) δ 11.18 (s, 1H), 8.06 – 7.92 (m, 4H), 7.82 (s, 2H), 7.80 – 7.74 (m, 2H), 7.63 – 7.47 (m, 10H), 5.00 – 4.94 (m, 2H), 3.34 (q, J = 6.7 Hz, 2H), 1.53 (s, 9H), 1.20 (s, 9H).

^{13}C NMR (126 MHz, $CDCl_3$) δ 162.7, 158.0, 156.6, 155.4, 152.7, 133.9, 133.4, 132.5, 131.2, 129.9, 129.7, 129.5, 128.1, 126.5, 84.2, 79.4, 54.44, 39.0, 28.1, 28.1.

^{19}F NMR (470 MHz, $CDCl_3$) δ -153.0 (BF_4^- minor), -153.1 (BF_4^- major).

FTIR (thin film) ν (cm^{-1}) = 1725, 1621, 1415, 1369, 1328, 1134, 1060, 754.

m.p. 124 – 129 °C

HRMS: (ESI+) *m/z* calculated for $C_{36}H_{41}N_4O_4$ requires *m/z* 593.3122 $[M-BF_4^-]^+$. Found *m/z* 593.3126.

1-(4-Nitrophenethyl)-2,4,6-triphenylpyridin-1-ium tetrafluoroborate (S-5)

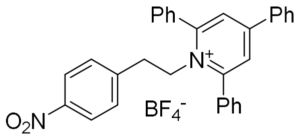

Prepared using **General Procedure A** on a 2 mmol scale from 4-nitrophenethylamine.HCl. The solid was filtered, washed with Et_2O (3 x 25 mL) and hexane (3 x 25 mL), and dried under high vacuum resulting in the title compound as an off-white solid (782 mg, 1.44 mmol, 72% yield).

^{1}H NMR (400 MHz, $CDCl_3$) δ 7.86 – 7.75 (m, 7H), 7.68 – 7.53 (m, 7H), 7.50 (t, *J* = 7.4 Hz, 1H), 7.40 (t, *J* = 7.6 Hz, 2H), 6.41 (d, *J* = 8.2 Hz, 2H), 4.55 (t, *J* = 8.1 Hz, 1H), 2.82 (t, *J* = 8.1 Hz, 1H).

^{13}C NMR (101 MHz, CD_3CN) δ 157.6, 156.7, 148.3, 144.3, 134.5, 133.7, 133.6, 132.3, 130.8, 130.5, 130.4, 130.1, 129.4, 127.8, 124.8, 56.1, 35.8.

^{19}F NMR (376 MHz, CD_3CN) δ -151.7 (BF_4^- minor), -151.8 (BF_4^- major).

FTIR (thin film) ν (cm^{-1}) = 1624, 1348, 1056, 752, 704.

m.p. 119 – 124 °C

HRMS: (ESI+) *m/z* calculated for $C_{31}H_{25}N_2O_2$ requires *m/z* 457.1911 $[M-BF_4^-]^+$. Found *m/z* 457.1914.

2,4,6-Triphenyl-1-(1-(pyrimidin-2-yl)piperidin-4-yl)pyridin-1-ium tetrafluoroborate (S-42)

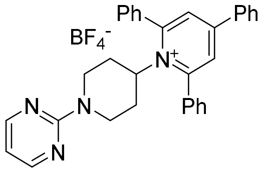

Prepared using **General Procedure B** using 4Å molecular sieves on a 1.2 mmol scale from 1-(pyrimidin-2-yl)piperidin-4-amine dihydrochloride. The resulting solid was filtered, washed with Et_2O (3 x 25 mL) and hexane (3 x 25 mL). Purification by column chromatography [DCM:acetone 95:5 → 60:40] resulted in the title compound as an off-white solid (618 mg, 1.11 mmol, 93% yield).

^{1}H NMR (500 MHz, $CDCl_3$) δ 8.17 (d, *J* = 4.7 Hz, 2H), 7.81 (s, 2H), 7.77 – 7.68 (m, 6H), 7.55 – 7.48 (m, 7H), 7.45 (dd, *J* = 8.2, 6.7 Hz, 2H), 6.47 (t, *J* = 4.7 Hz, 1H), 4.94 (tt, *J* = 12.5, 3.2 Hz, 1H), 4.54 (dt, *J* = 13.6, 2.3 Hz, 2H), 2.29 (ddd, *J* = 13.9, 11.8, 2.2 Hz, 2H), 2.19 – 2.13 (m, 2H), 1.78 (qd, *J* = 12.3, 4.1 Hz, 2H).

^{13}C NMR (126 MHz, $CDCl_3$) δ 161.3, 157.6, 157.3, 155.5, 134.1, 133.8, 132.1, 131.0, 129.7, 129.4, 129.0, 128.4, 128.4, 110.6, 70.3, 44.7, 32.1.

^{19}F NMR (470 MHz, $CDCl_3$) δ -152.9 (BF_4^- minor), -152.9 (BF_4^- major).

FTIR (thin film) ν (cm^{-1}) = 2981, 2625, 1621, 1584, 1479, 1450, 1357, 1056, 751, 706.

m.p. 134 – 139 °C

HRMS: (ESI+) *m/z* calculated for $C_{32}H_{29}N_4$ requires *m/z* 469.2387 $[M-BF_4^-]^+$. Found *m/z* 469.2374.

Scheme S12: Multistep synthesis of 1-(2-(4-(3-cyano-1*H*-indole-7-carbonyl)piperazin-1-yl)ethyl)-2,4,6-triphenylpyridin-1-ium tetrafluoroborate (**S-45**)

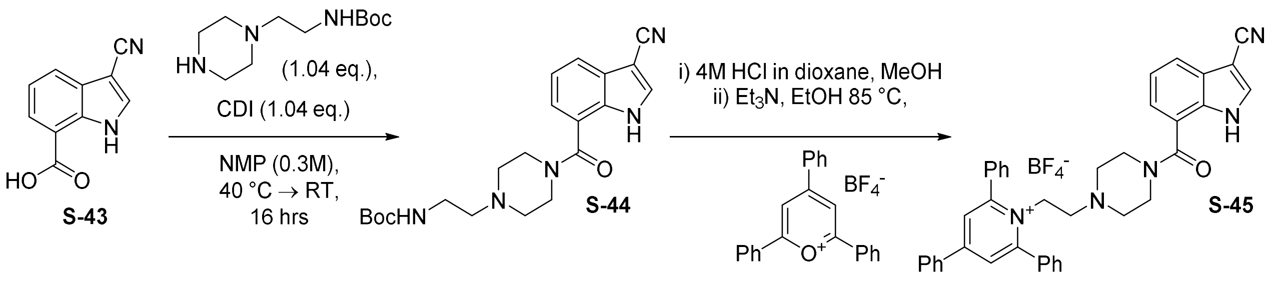

3-Cyano-1*H*-indole-7-carboxylic acid (**S-43**)

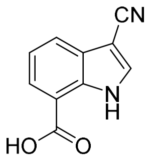

Prepared as previously described in literature from methyl 3-cyano-*1H*-indole-7-carboxylate.

***Tert*-butyl (2-(4-(3-cyano-*1H*-indole-7-carbonyl)piperazin-1-yl)ethyl)carbamate (S-44)**

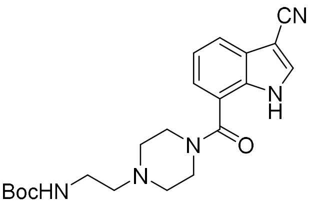

Method adapted from previously published route.[52]

In a 2-neck flask under N_2, 3-cyano-1*H*-indole-7-carboxylic acid (242 mg, 1.44 mmol, 1 eq.) was dissolved in NMP (2.3 mL) and heated to 80 °C until fully dissolved. The mixture was cooled to 40 °C and CDI (243 mg, 1.5 mmol, 1.04 eq.) was added and the mixture stirred at room temperature for 1 hour. After this time, 1-(2-*N*-Boc-aminoethyl)piperazine (394 mg, 1.5 mmol, 1.04 eq.) in NMP (2.3 mL) was added and the reaction mixture stirred for 16 h. Upon completion, the reaction mixture was diluted with Et_2O (10 mL) and washed with brine (3 x 10 mL). The organic fraction was dried over sodium sulfate, filtered, and solvent removed under reduced pressure. Purification by column chromatography [DCM → 8% MeOH in DCM] resulted in a white foam (403 mg, 1.015 mmol, 70% yield).

^{1}H NMR (500 MHz, CDCl$_3$) δ 10.13 (s, 1H), 7.84 (d, *J* = 7.8 Hz, 1H), 7.69 (d, *J* = 2.8 Hz, 1H), 7.35 – 7.26 (m, 2H), 4.93 (s, 1H), 3.76 (s, 4H), 3.25 (s, 2H), 2.55 – 2.48 (m, 6H), 1.45 (s, 9H).

^{13}C NMR (126 MHz, CDCl$_3$) δ 168.3, 156.1, 134.3, 133.0, 128.5, 123.3, 122.3, 121.2, 118.2, 115.6, 87.6, 79.5, 57.4, 53.1, 37.2, 29.8, 28.6.

FTIR (thin film) ν (cm^{-1}) = 2917, 2360, 2224, 1670, 1622, 1435, 1269, 1165, 910, 731.

m.p. 56 – 60 °C

HRMS: (ESI+) *m/z* calculated for $C_{21}H_{28}N_5O_3$ requires *m/z* 398.2187 $[M+H]^+$. Found *m/z* 398.2183.

1-(2-(4-(3-Cyano-1*H*-indole-7-carbonyl)piperazin-1-yl)ethyl)-2,4,6-triphenylpyridin-1-ium Tetrafluoroborate (S-45)

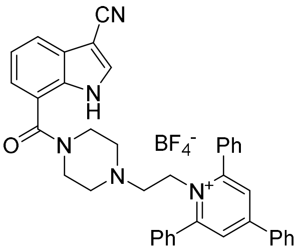

Prepared using **General Procedure C** on a 0.42 mmol scale from tert-butyl (2-(4-(3-cyano-*1H*-indole-7-carbonyl)piperazin-1-yl)ethyl)carbamate (**S-44**). Purification by column chromatography [DCM:acetone 95:5 → 60:40] resulted in the title compound as a yellow foam (252 mg, 0.37 mmol, 89% yield).

^{1}H NMR (600 MHz, DMSO) δ 12.01 (s, 1H), 8.44 (s, 2H), 8.27 – 8.20 (m, 3H), 8.18 (d, *J* = 3.1 Hz, 1H), 7.94 – 7.87 (m, 4H), 7.75 – 7.65 (m, 6H), 7.62 (t, *J* = 7.5 Hz, 2H), 7.27 (t, *J* = 7.6 Hz, 1H), 7.17 (d, *J* = 7.2 Hz, 1H), 4.60 (t, *J* = 7.2 Hz, 2H), 3.28 (br. s, 4H), 2.46 (br. s, 2H), 2.01 (br. s, 4H).

^{13}C NMR (151 MHz, DMSO, T = 353K) δ 165.88, 156.21, 154.27, 134.73, 132.82, 132.58, 131.98, 131.45, 130.65, 129.14, 129.07, 128.72, 128.25, 127.25, 126.55, 125.68, 121.45, 120.88, 120.41, 119.46, 115.14, 84.74, 78.74, 54.72, 51.31.

^{19}F NMR (471 MHz, CDCl$_3$) δ -150.2 (BF_4^- minor), -150.3 (BF_4^- major).

FTIR (thin film) ν (cm^{-1}) = 2931, 2220, 1621, 1436, 1216, 763.

m.p. 145 – 150 °C

HRMS: (ESI+) *m/z* calculated for $C_{39}H_{34}N_5O$ requires *m/z* 588.2758 $[M-BF_4^-]^+$. Found *m/z* 588.2763.

Scheme S13: Multistep synthesis of 1-(3-(5-(3-chloro-4-methoxyphenyl)oxazole-4-carboxamido)propyl)-2,4,6-triphenylpyridin-1-ium Tetrafluoroborate (**33**)

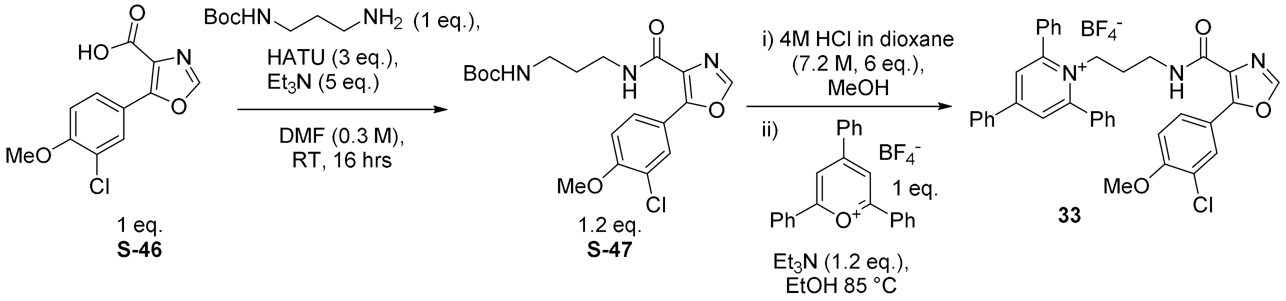

5-(3-Chloro-4-methoxyphenyl)oxazole-4-carboxylic acid (S-46)

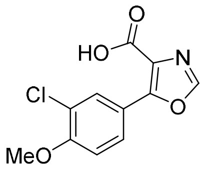

Prepared as previously described in literature from 3-chloro-4-methoxybenzoic acid.[40]

Tert-butyl (3-(5-(3-chloro-4-methoxyphenyl)oxazole-4-carboxamido)propyl)carbamate (S-47)

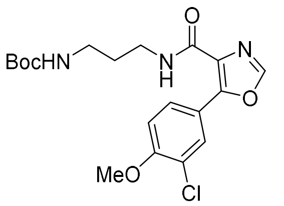

Under ambient conditions, to a solution of 5-(3-chloro-4-methoxyphenyl)oxazole-4-carboxylic acid (**S-46**) (6.448 g, 25.42 mmol, 1 eq.) in anhydrous DMF (85 mL, 0.3M) was added HATU (29 g, 76.3 mmol, 3 eq.) and *tert*-butyl(3-aminopropyl)carbamate (4.43 g, 25.42 mmol, 1 eq.). Triethylamine (17.7 mL, 127.1 mmol, 5 eq.) was added dropwise and the mixture stirred at room temperature for 16 h.

Upon completion, H_2O (10 mL) was added, and the mixture was extracted with EtOAc (3 x 10 mL). The combined organics were washed with brine (3 x 10 mL) and subsequently dried over sodium sulfate, filtered, and the solvent concentrated under reduced pressure. Purification by column chromatography [20% EtOAc in heptane → 40% EtOAc in heptane] resulted in a white solid (10.24 g, 24.98 mmol, 98% yield).

^{1}H NMR (400 MHz, $CDCl_3$) δ 8.36 (dd, *J* = 8.7, 2.1 Hz, 1H), 8.31 (d, *J* = 2.2 Hz, 1H), 7.80 (s, 1H), 7.52 (br. s, 1H), 7.01 (d, *J* = 8.8 Hz, 1H), 4.92 (br. s, 1H), 3.96 (s, 3H), 3.50 (q, *J* = 6.5 Hz, 2H), 3.21 (q, *J* = 6.3 Hz, 2H), 1.76 (p, *J* = 6.5 Hz, 2H), 1.45 (s, 9H).

^{13}C NMR (101 MHz, $CDCl_3$) δ 161.6, 156.4, 156.3, 151.6, 147.8, 130.0, 128.6, 128.3, 122.6, 120.6, 111.7, 79.4, 56.4, 37.7, 36.3, 30.5, 28.6.

FTIR (thin film) ν (cm^{-1}) = 3367, 2979, 1703, 1662, 1522, 1265, 1172, 1069, 761.

m.p. 136 – 140 °C

HRMS: (ESI+) *m/z* calculated for $C_{19}H_{25}ClN_3O_5$ requires *m/z* 410.1477 $[M+H]^+$. Found *m/z* 410.1480.

1-(3-(5-(3-Chloro-4-methoxyphenyl)oxazole-4-carboxamido)propyl)-2,4,6-triphenylpyridin-1-ium Tetrafluoroborate (33)

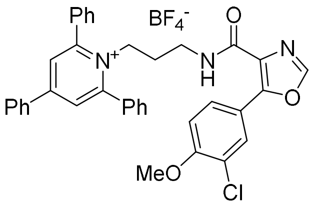

Prepared according to **General Procedure C** from *tert*-butyl (3-(5-(3-chloro-4-methoxyphenyl)oxazole-4-carboxamido)propyl)carbamate (**S-47**) on a 10 mmol scale. Purification by column chromatography [DCM → DCM:MeOH 95:5] resulted in a white foam (7.31 g, 10.63 mmol, 43% yield).

^{1}H NMR (400 MHz, CDCl$_3$) δ 8.37 (d, *J* = 2.2 Hz, 1H), 8.22 (dd, *J* = 8.7, 2.2 Hz, 1H), 7.88 (s, 2H), 7.76 (d, *J* = 7.7 Hz, 7H), 7.58 – 7.44 (m, 7H), 7.38 (t, *J* = 7.5 Hz, 2H), 7.04 – 7.02 (m, 2H), 4.64 – 4.55 (m, 2H), 3.98 (s, 3H), 2.99 (q, *J* = 6.1 Hz, 2H), 1.90 – 1.78 (m, 2H).

^{13}C NMR (101 MHz, CDCl$_3$) δ 161.3, 156.8, 156.4, 156.2, 151.4, 147.6, 134.1, 132.6, 132.3, 131.0, 130.0, 129.8, 129.4, 129.0, 128.3, 128.3, 127.8, 127.0, 122.7, 120.5, 111.8, 56.4, 53.3, 36.0, 30.3.

^{19}F NMR (376 MHz, CDCl$_3$) δ -153.1 (BF_4^- minor), -153.2 (BF_4^- major).

FTIR (thin film) ν (cm^{-1}) = 3035, 2926, 1623, 1518, 1604, 763.

m.p. 112 – 115 °C

HRMS: (ESI+) *m/z* calculated for $C_{37}H_{31}ClN_3O_3$ requires *m/z* 600.2048 $[M-BF_4^-]^+$. Found *m/z* 600.2021.

Scheme S14: Multistep synthesis of 1-(3-(7-hydroxy-2-oxo-*2H*-chromene-3-carboxamido)propyl)-2,4,6-triphenylpyridin-1-ium Tetrafluoroborate (**S-49**)

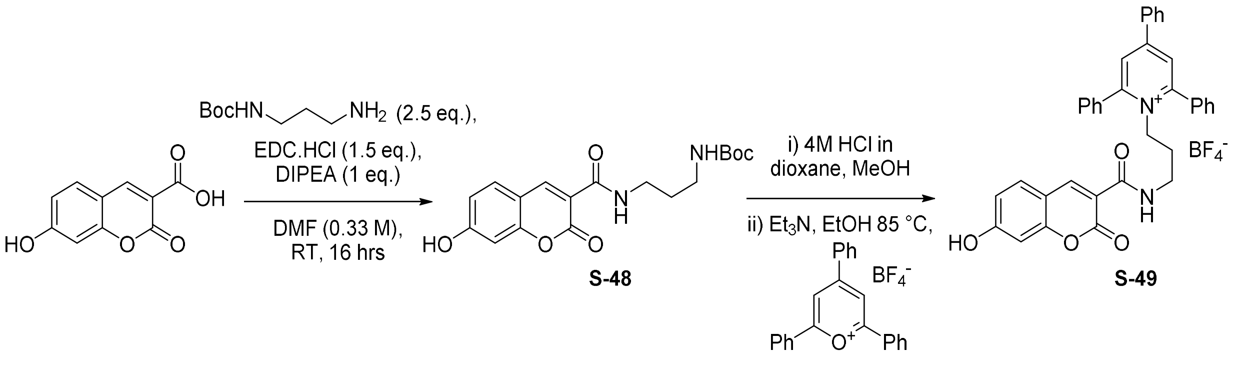

Tert-butyl (3-(7-hydroxy-2-oxo-_2H_-chromene-3-carboxamido)propyl)carbamate (S-48)

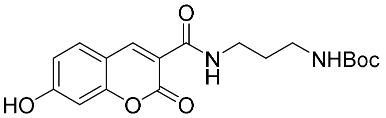

In a 2-neck flask under N_2, 7-hydroxy-2-oxo-*2H*-chromene-3-carboxylic acid (206 mg, 1 mmol, 1 eq.), *tert*-butyl-3-aminopropylcarbamate (436 mg, 2.5 mmol, 2.5 eq.) and EDC.HCl (288 mg, 1.5 mmol, 1.5 eq.) were dissolved in DMF (3 mL, 0.33 M) and DIPEA (100 µL, 1 mmol, 1 eq.) was added. The reaction mixture was stirred at room temperature for 16 h. Upon completion, H_2O (10 mL) was added and the mixture extracted with DCM (3 x 25 mL). The combined organics were washed with brine (3 x 25 mL), dried over sodium sulfate, filtered, and the solvent removed under reduced pressure. Purification by column chromatography [100% DCM → 8% MeOH in DCM] resulted in the title compound as an off-white solid (163 mg, 0.45 mmol, 45% yield).

^{1}H NMR (400 MHz, DMSO-*d*$_6$) δ 11.03 (s, 1H), 8.77 (s, 1H), 8.67 (t, *J* = 5.8 Hz, 1H), 7.81 (d, *J* = 8.7 Hz, 1H), 6.90 – 6.82 (m, 2H), 6.80 (d, *J* = 2.1 Hz, 1H), 3.32 – 3.28 (m, 2H), 2.96 (q, *J* = 6.6 Hz, 2H), 1.60 (p, *J* = 6.7 Hz, 2H), 1.37 (s, 9H).

^{13}C NMR (101 MHz, DMSO-*d*$_6$) δ 163.6, 161.6, 160.9, 156.3, 155.6, 148.0, 132.0, 114.3, 113.8, 111.1, 101.8, 77.5, 37.4, 36.7, 29.6, 28.2.

FTIR (thin film) ν (cm^{-1}) = 3419, 2936, 2487, 1702, 1614, 1255, 1121, 978, 796.

m.p. 200 – 202 °C

HRMS: (ESI+) *m/z* calculated for $C_{18}H_{23}N_2O_6$ requires *m/z* 363.1551 [M+H]$^+$. Found *m/z* 363.1551.

1-(3-(7-Hydroxy-2-oxo-_2H_-chromene-3-carboxamido)propyl)-2,4,6-triphenylpyridin-1-ium Tetrafluoroborate (S-49)

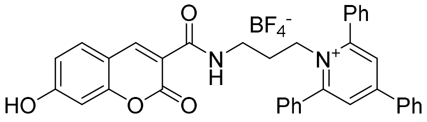

Prepared according to **General Procedure C** on a 0.4 mmol scale from *tert*-butyl (3-(7-hydroxy-2-oxo-2H-chromene-3-carboxamido)propyl)carbamate (**S-48**). Upon completion of the reaction, the mixture was diluted with Et_2O (30 mL) resulting in an orange gel. The Et_2O supernatant was decanted and discarded. The residue was dissolved in the minimum volume of DCM (5 mL) and diluted with Et_2O (30 mL). The mixture was sonicated and the Et_2O decanted and discarded. This process was repeated 3 times, and the mixture was then decanted over a celite pad on a fritted filer. The resulting solid was washed with water (15 mL), Et_2O (15 mL), and hexane (15 mL). The aqueous and organic filtrates were disposed of. The solid remaining in the flask was dissolved in DCM and then this solution was passed over the celite pad and collected. Removal of DCM under reduced pressure resulted in the title compound as an orange foam (243 mg, 0.38 mmol, 95% yield).

^{1}H NMR (600 MHz, DMSO-d_6) δ 8.53 (s, 1H), 8.41 (s, 2H), 8.25 (t, *J* = 6.1 Hz, 1H), 8.22 – 8.17 (m, 2H), 7.85 – 7.79 (m, 4H), 7.73 (d, *J* = 8.7 Hz, 1H), 7.62 – 7.52 (m, 8H), 7.51 – 7.44 (m, 2H), 6.80 (dd, *J* = 8.6, 2.2 Hz, 1H), 6.68 (d, *J* = 2.2 Hz, 1H), 4.51 – 4.34 (m, 2H), 2.89 (q, *J* = 6.0 Hz, 2H), 1.61 (ddt, *J* = 11.2, 7.8, 3.9 Hz, 2H).

^{13}C NMR (151 MHz, DMSO-d_6) δ 166.6, 161.8, 160.9, 156.8, 156.0, 154.0, 147.8, 133.0, 132.9, 132.3, 131.9, 130.5, 129.5, 129.1, 128.9, 128.6, 126.0, 115.6, 110.9, 110.0, 102.0, 53.1, 35.6, 29.5.

^{19}F NMR (471 MHz, DMSO-d_6) δ -148.2 (BF_4^- minor), -148.3 (BF_4^- major).

FTIR (thin film) ν (cm^{-1}) = 3344, 2956, 1706, 1621, 1537, 1222, 1060, 764.

m.p. 164 – 167 °C

HRMS: (ESI+) *m/z* calculated for $C_{36}H_{29}N_2O_4$ requires *m/z* 553.2122 [M-BF_4^-]$^+$. Found *m/z* 553.2120.

Scheme S15: Multistep synthesis of 1-(3-(4-(2-(benzhydryloxy)ethyl)piperazin-1-yl)propyl)-2,4,6-triphenylpyridin-1-ium Tetrafluoroborate (**S-52**)

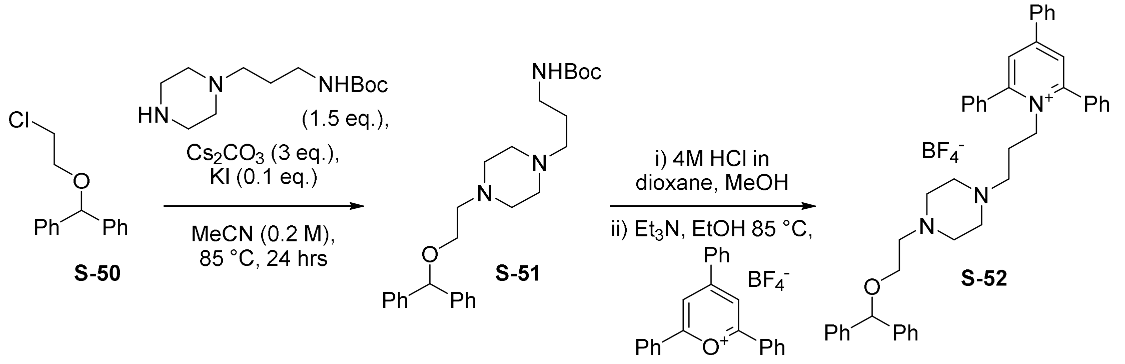

((2-Chloroethoxy)methylene)dibenzene (S-50)

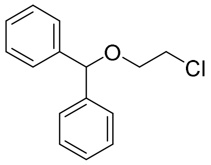

Prepared as previously described in literature from 1,1-diphenylmethanol.[53]

Tert-butyl (3-(4-(2-(benzhydryloxy)ethyl)piperazin-1-yl)propyl)carbamate dibenzene (S-51)

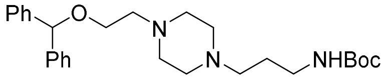

Under an N_2 atmosphere, Cs_2CO_3 (4 g, 12 mmol, 3 eq.), KI (72 mg, 0.4 mmol, 0.1 eq.), and *tert*-butyl (3-(piperazin-1-yl)propyl)carbamate (1.46 mg, 6 mmol, 1.5 eq.) were added to a Schlenk flask charged with stirrer bar. ((2-Chloroethoxy)methylene)dibenzene (1 g, 4 mmol, 1 eq.) was dissolved MeCN (8 mL, 0.5 M) and added to the reaction vessel. The mixture was heated to reflux at 85 °C for

24 h. Purification by column chromatography [DCM → 5% MeOH in DCM] resulted in the title compound as a yellow oil (1 g, 2.21 mmol, 54% yield).

^{1}H NMR (400 MHz, CDCl$_3$) δ 7.36 – 7.27 (m, 8H), 7.26 – 7.20 (m, 2H), 5.48 (br. s, 1H), 5.37 (s, 1H), 3.60 (t, *J* = 6.0 Hz, 2H), 3.18 (d, *J* = 5.9 Hz, 2H), 2.68 (t, *J* = 6.0 Hz, 2H), 2.63 – 2.33 (m, 10H), 1.65 (p, *J* = 6.6 Hz, 2H), 1.43 (s, 9H).

^{13}C NMR (101 MHz, CDCl$_3$) δ 156.2, 142.4, 128.5, 127.6, 127.1, 84.1, 67.1, 58.0, 57.0, 53.8, 53.3, 40.1, 31.7, 28.6, 26.4, 22.8, 14.3.

FTIR (thin film) ν (cm^{-1}) = 3348, 2978, 2815, 1708, 1508, 1274, 1168, 760, 703.

HRMS: (ESI+) *m/z* calculated for $C_{27}H_{40}N_3O_3$ requires *m/z* 454.3064 [M+H]$^+$. Found *m/z* 454.3065.

1-(3-(4-(2-(Benzhydryloxy)ethyl)piperazin-1-yl)propyl)-2,4,6-triphenylpyridin-1-ium Tetrafluoroborate (S-52)

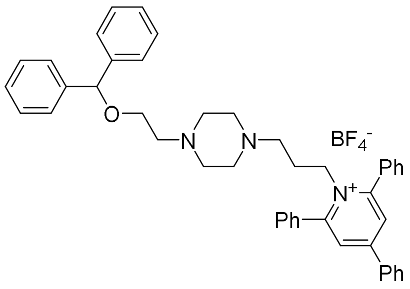

Prepared according to **General Procedure C** on a 0.73 mmol scale from *tert*-butyl (3-(4-(2-(benzhydryloxy)ethyl)piperazin-1-yl)propyl)carbamate (**S-51**). Upon completion of the reaction, the mixture was diluted with Et_2O (30 mL) resulting in an orange gel. Purification by column chromatography [DCM → 40% Acetone in DCM] resulted in the desired product as a yellow solid (62 mg, 0.09 mmol, 12% yield).

^{1}H NMR (500 MHz, CDCl$_3$) δ 7.87 (s, 2H), 7.82 – 7.73 (m, 6H), 7.57 (t, *J* = 7.7 Hz, 5H), 7.54 – 7.46 (m, 4H), 7.38 – 7.29 (m, 8H), 7.25 (d, *J* = 5.3 Hz, 2H), 5.38 (s, 1H), 4.54 – 4.47 (m, 2H), 3.63 (s, 2H), 2.74 (s, 2H), 2.43 (br. s, 4H), 1.99 – 1.83 (m, 6H), 1.62 (s, 2H).

^{13}C NMR (126 MHz, CDCl$_3$) δ 156.6, 156.1, 142.1, 134.2, 132.8, 132.3, 131.2, 129.9, 129.5, 129.2, 128.6, 128.3, 127.7, 127.1, 127.0, 84.1, 66.1, 57.6, 54.2, 53.8, 53.2, 51.3, 26.7.

^{19}F NMR (470 MHz, CDCl$_3$) δ -152.7 (BF_4^- minor), -152.7 (BF_4^- major).

FTIR (thin film) ν (cm^{-1}) = 3034, 2922, 1624, 1061, 764.

m.p. 83 – 87 °C

HRMS: (ESI+) *m/z* calculated for $C_{45}H_{46}N_3O$ requires *m/z* 644.3635 [M-BF_4^-]$^+$. Found *m/z* 644.3651.

2.3 Preparation of Aryl Iodide Radiolabelling Precursors

The following compounds were prepared as described previously in literature reports:

1-(difluoro((trifluoromethyl)sulfonyl)methyl)-4-iodobenzene[46] **(S-10)**

5-(4-iodophenyl)-*5H*-dibenzo[*b,d*]thiophen-5-ium Triflate[44] **(S-13)**

2-(3-chloro-5-iodophenyl)-4,4,5,5-tetramethyl-1,3,2-dioxaborolane[54] **(S-53)**

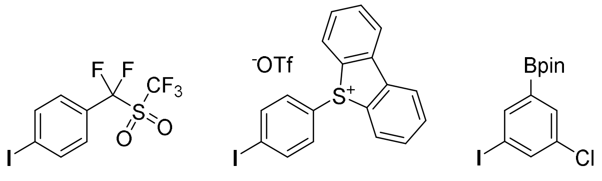

2.3.1 Characterisation Data of Aryl Iodide Radiolabelling Precursors

Scheme S16: Multistep synthesis of 5-iodo-*N,N,N*-trimethylpyridin-2-aminium Triflate (**S-12**)

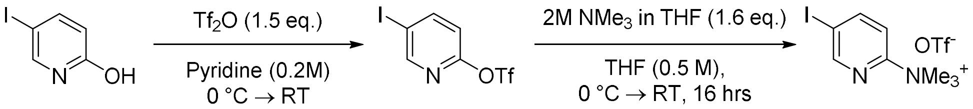

The title compound was prepared by adapting a known literature procedure.[55]

5-Iodopyridin-2-yl trifluoromethanesulfonate (S-54)

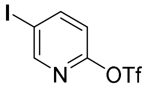

5-Iodopyridin-2-yl trifluoromethanesulfonate was prepared as previously described in literature from 5-iodopyridin-2-ol on a 4.5 mmol scale. The resulting compound was used in the subsequent step following column chromatography (Hexane → 10% EtOAc) which resulted in a colourless liquid (1.46 g, 4.1 mmol, 91% yield).

5-Iodo-*N,N,N*-trimethylpyridin-2-aminium Triflate (S-12)

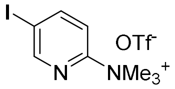

5-Iodopyridin-2-yl trifluoromethanesulfonate (**S-54**) (1. g, 4.1 mmol) was dissolved in anhydrous THF (4.8 mL) and cooled to 0 °C. NMe_3 in THF (2M, 3.4 mL, 6.8 mmol, 1.6 eq.) was added dropwise and the cooling bath removed. The mixture was left to stir for 16 h and the resulting solid was filtered and washed with diethyl ether (2 x 10 mL). The compound was dissolved in chloroform (5 mL) and filtered over celite. The chloroform filtrate had solvent removed under reduced pressure resulting in the title compound as a white solid (927 mg, 2.25 mmol, 55% yield).

^{1}H NMR (500 MHz, $CDCl_3$) δ 8.74 (d, J = 2.2 Hz, 1H), 8.36 (dd, J = 8.7, 2.2 Hz, 1H), 7.95 (d, J = 8.7 Hz, 1H), 3.68 (s, 9H).

{^{19}F}^{13}C NMR (126 MHz, $CDCl_3$) δ 156.1, 155.0, 149.6, 119.4, 117.1, 95.8, 55.5.

^{19}F NMR (471 MHz, $CDCl_3$) δ -78.5 (triflate -CF_3).

FTIR (thin film) ν (cm^{-1}) 2961, 2929, 1729, 1491, 1469, 1277, 1164, 1074, 766.

m.p. 122 – 125 °C

HRMS: (ESI+) *m/z* calculated for $C_8H_{12}IN_2$ requires *m/z* 263.0040 [M-OTf^-]$^+$. Found *m/z* 263.0036.

2-(4-Iodophenoxy)ethyl 4-methylbenzenesulfonate (S-11)

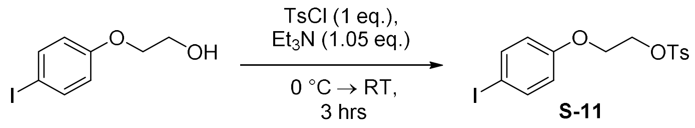

In a 2-neck flask under N_2 was added 2-(4-iodophenoxy)ethanol (100 mg, 0.38 mmol, 1 eq.) and DCM (1.5 mL) and the mixture cooled to 0 °C. Et_3N (55 µL, 0.4 mmol, 1.05 eq.) were added followed by tosyl chloride (72.5 mg, 0.38 mmol, 1 eq.). The ice bath was removed and the mixture left to stir at room temperature for 3 h. Purification by column chromatography [10% Et_2O in pentane → 60% Et_2O in pentane] resulted in the title compound as a white solid (130 mg, 0.31 mmol, 82% yield).

^{1}H NMR (400 MHz, $CDCl_3$) δ 7.81 – 7.78 (m, 2H), 7.56 – 7.47 (m, 2H), 7.33 (d, J = 8.0 Hz, 2H), 6.60 – 6.51 (m, 2H), 4.37 – 4.33 (m, 2H), 4.13 – 4.09 (m, 2H), 2.45 (s, 3H).

^{13}C NMR (101 MHz, $CDCl_3$) δ 158.0, 145.2, 138.4, 132.9, 130.0, 128.1, 117.1, 83.7, 68.0, 65.6, 21.8.

FTIR (thin film) ν (cm^{-1}) = 3067, 1588, 1486, 1359, 1246, 1177, 1022, 934, 819, 735, 665.

m.p. 99 – 103 °C

HRMS: Not found

2.4 Preparation of Reference Compounds

The following reference compounds were purchased from commercial suppliers:

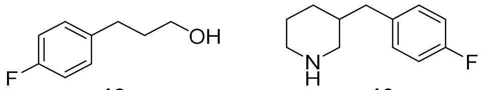

12 **13**

The following compounds were prepared as described previously in literature reports:

1-(4-fluorophenethyl)pyrrolidine[56] (**11**)

4-(4-fluorophenyl)tetrahydro-*2H*-pyran[57] (**29**)

tert-butyl 4-(4-fluorophenyl)piperidine-1-carboxylate (**31**)

1-(2-fluoroethoxy)-4-iodobenzene[58] (**36**)

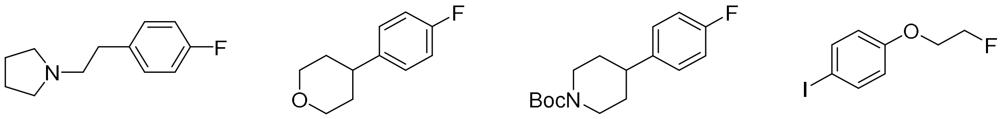

11 **29** **31** **36**

2.4.1 General Procedure D: Reductive Cross-Coupling of Alkyl Pyridinium Salts with Aryl bromides

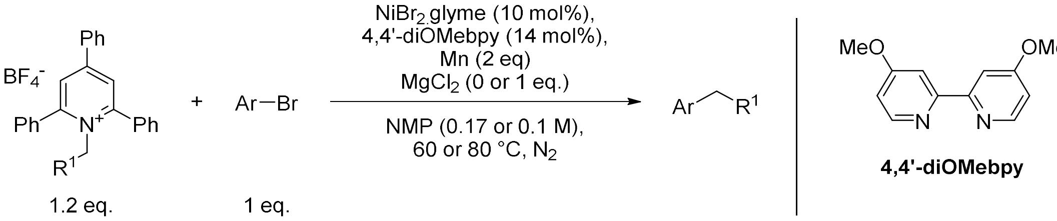

This procedure was adapted from previously published procedures by Watson and co-workers as well as Martin and co-workers.[54,55]

A stock solution of $NiBr_2$·glyme (10 mol%), 4,4'-dimethoxy-2,2'-bipyridine (14 mol %), and *N*-Methyl-2-pyrrolidone (NMP) (0.17 M or 0.1M) was prepared in a flame-dried Schlenk flask under a flow of N_2 gas. Under an N_2 atmosphere, the stock solution was degassed by bubbling N_2 through the solution.

To a separate flame-dried Schlenk flask were added manganese powder (2 eq. unless otherwise specified), alkyl pyridinium salt (1.2 eq.), and aryl bromide (1.0 eq.), if solid and an additive of $MgCl_2$ (1 eq.) (if used). The flask was then evacuated and refilled with N_2 (x 3). If the aryl bromide was a liquid, it was added *via* syringe at this point under a flow of N_2. The nickel/ligand stock solution was next added, and the flask was sealed. The resultant mixture was then stirred at the specified temperature and time.

The mixture was then allowed to cool to room temperature and diluted with ethyl acetate (10 mL) and was filtered over celite. The filter cake was washed with ethyl acetate (4 x 10 mL). The filtrate was then washed with a sat. solution of NaCl (aq., 4 x 25mL) and the organics dried with sodium sulfate and the solvent removed. The crude cross-coupled product was then purified *via* silica gel chromatography.

Slow addition: If slow addition by syringe pump is specified the reaction is set-up as above in NMP (4.0 mL) excluding the aryl bromide and pyridinium salt and heated to 80 °C. The alkyl pyridinium salt (1.0 mmol, 1.0 eq.) and aryl bromide (1.1 mmol, 1.1 eq.) were combined separately under N_2 in NMP (2.0 mL) and degassed. The resulting mixture was transferred to an N_2-purged 3 mL syringe and added steadily over 3 h *via* syringe pump. The resulting mixture was then stirred at 80 °C between 12 –24 h and the work-up and purification followed as above.

2.4.2 General Procedure E: Reductive Cross-Coupling of Alkyl Halides with Aryl Halides

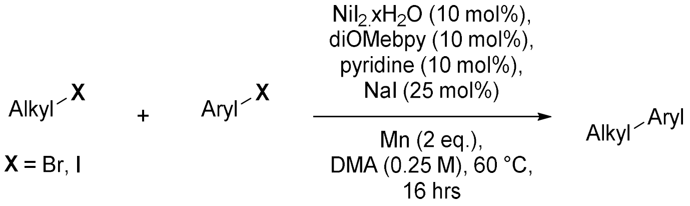

Method adapted from Weix and co-workers.[59]

The identity of the halide coupling partners and their respective stoichiometry is specified for each example.

Under ambient conditions, $NiI_2.xH_2O$ (10 mol%), diOMebpy (10 mol%), manganese (2 eq.) and, NaI (25 mol%) were added to a vial containing a stirrer bar along with alkyl halide (1 – 2 eq.) and aryl halide (1 eq.), if solid. Anhydrous DMA (0.25 M) was subsequently added followed by anhydrous pyridine (10 mol%) and if liquids, alkyl halide (1.2 – 2 eq.) and aryl halide (1 eq.). The vial was sealed with a septum lined screw cap, fitted with a vent needle, and sparged with N_2 from a balloon for 15 seconds under sonication. The reaction mixture was then heated to 60 °C for 16 h.

After this time, the reaction mixture was cooled to room temperature, diluted with EtOAc (3 volumes) and filtered over celite. The celite was washed with EtOAc (2 x 1 volume) and the combined organics were washed with brine (3 x 15 mL). The organics were dried over Na_2SO_4, filtered, and the solvent was removed under reduced pressure. Purification by column chromatography resulted in the desired cross-coupled product.

2.3.3 General Procedure F: Amide Coupling

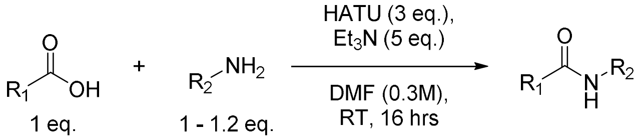

Under ambient conditions, to a solution of the relevant carboxylic acid (1 eq.) in anhydrous DMF (0.3M) was added HATU (3 eq.) and the relevant amine (1 eq.). Triethylamine (5 eq.) was added dropwise and the mixture stirred at room temperature for 16 h.

Upon completion, H_2O (3 volumes) was added, and this was then extracted with EtOAc (3 x 3 volumes). The combined organics were washed with brine (3 x 3 volumes) and subsequently dried over Na_2SO_4, filtered, and the solvent was concentrated under reduced pressure. Purification by column chromatography resulted in the desired amide.

2.4.4 Characterisation Data of Reference Compounds

Methyl (*S*)-2-((*tert*-butoxycarbonyl)amino)-6-(4-fluorophenyl)hexanoate (23)

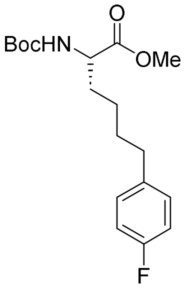

Prepared using **General Procedure D** from (*S*)-1-(5-((tert-butoxycarbonyl)amino)-6-methoxy-6-oxohexyl)-2,4,6-triphenylpyridin-1-ium tetrafluoroborate (**S-18)** (191.5 mg, 0.30 mmol, 1.4 eq.), 1-bromo-4-fluorobenzene (23 µL, 0.21 mmol, 1 eq.), and manganese (17 mg, 0.32 mmol, 1.5 eq.) at 60 °C. The total reaction concentration of NMP was 0.1 M (2 mL). After column chromatography [pentane → 90:10 pentane:Et_2O], the compound was isolated as a clear oil (55 mg, 0.162 mmol, 77% yield).

^{1}H NMR (600 MHz, $CDCl_3$) δ 7.10 (dd, *J* = 8.6, 5.4 Hz, 2H), 6.95 (t, *J* = 8.7 Hz, 2H), 4.98 (d, *J* = 8.5 Hz, 1H), 4.30 (d, *J* = 6.7 Hz, 1H), 3.72 (s, 3H), 2.57 (t, *J* = 7.7 Hz, 2H), 1.81 (br. s, 1H), 1.68 – 1.55 (m, 3H), 1.44 (s, 9H), 1.41 – 1.30 (m, 2H).

^{13}C NMR (151 MHz, $CDCl_3$) δ 173.5, 161.4 (d, *J* = 243.2 Hz), 155.5, 137.9 (d, *J* = 3.2 Hz), 129.8 (d, *J* = 7.7 Hz), 115.14 (d, *J* = 21.1 Hz), 80.1, 53.5, 52.4, 34.9, 32.8, 31.1, 28.5, 24.9.

{^{1}H}^{19}F NMR (565 MHz, $CDCl_3$) δ -118.0.

FTIR (thin layer film) ν (cm^{-1}) = 1744, 1710, 1510, 1439, 1223, 1167, 759.

HRMS: (ESI+) calculated for $C_{18}H_{27}FNO_4$ $[M+H]^+$. *m/z* 340.1919; found 340.1919.

3-(4-Fluorophenethyl)-*1H*-indole (14)

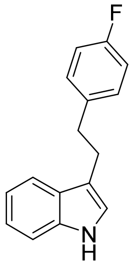

Prepared according to **General Procedure D** from 1-(2-(*1H*-indol-3-yl)ethyl)-2,4,6-triphenylpyridin-1-ium tetrafluoroborate (**S-19**) (127 mg, 0.24 mmol, 1.2 eq.), 1-bromo-4-fluorobenzene (57 µL, 0.2 mmol, 1 eq.), and $MgCl_2$ (95 mg, 1 mmol, 1 eq.) at 80 °C. The total reaction concentration of NMP was 0.1 M (6 mL). After column chromatography [Pentane → 95:5 Pentane:EtOAc], the title compound was isolated as an off-white solid (20 mg, 0.08 mmol, 40% yield).

^{1}H NMR (500 MHz, $CDCl_3$) δ 7.90 (br. s, 1H), 7.61 (d, *J* = 7.7 Hz, 1H), 7.37 (d, *J* = 8.0 Hz, 1H), 7.24 – 7.18 (m, 1H), 7.17 – 7.10 (m, 3H), 7.00 – 6.92 (m, 2H), 6.89 (d, *J* = 2.2 Hz, 1H), 3.10 – 3.02 (m, 2H), 3.03 – 2.96 (m, 2H).

^{13}C NMR (126 MHz, $CDCl_3$) δ 161.4 (d, *J* = 243.1 Hz), 138.1 (d, *J* = 3.2 Hz), 136.4, 130.0 (d, *J* = 7.7 Hz), 127.5, 122.1, 121.5, 119.4, 119.0, 116.0, 115.1 (d, *J* = 21.2 Hz), 111.2, 35.8, 27.5.

^{19}F NMR (471 MHz, $CDCl_3$) δ -117.8 (td, *J* = 8.9, 4.5 Hz).

Spectroscopic data are consistent with the literature known compound.[60]

2-(4-Fluorophenethyl)thiophene (6)

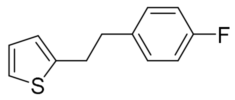

Prepared by **General Procedure D** (80 °C) from 2,4,6-triphenyl-1-(2-(thiophen-2-yl)ethyl)pyridin-1-ium tetrafluoroborate (**S-20**) and 1-bromo-4-fluorobenzene on a 0.2 mmol scale. Purification by column chromatography (100% Pentane) resulted in the product as a clear oil, (12 mg, 0.06 mmol, 29% yield).

^{1}H NMR (400 MHz, $CDCl_3$) δ 7.18 – 7.08 (m, 3H), 7.02 – 6.91 (m, 2H), 6.91 (dd, *J* = 5.1, 3.4 Hz, 1H), 6.77 – 6.73 (m, 1H), 3.16 – 3.07 (m, 2H), 2.96 (t, *J* = 7.8 Hz, 2H).

^{13}C NMR (126 MHz, $CDCl_3$) δ 161.4 (d, *J* = 243.8 Hz), 144.1, 136.7 (d, *J* = 3.2 Hz), 129.8 (d, *J* = 7.8 Hz), 126.7, 124.5, 123.2, 115.1 (d, *J* = 21.0 Hz), 37.3, 31.9.

^{19}F NMR (377 MHz, $CDCl_3$) δ -117.2 – -117.3 (m).

FTIR (neat) ν (cm^{-1}) = 2926.51, 1510.72, 1224.61, 830.18, 695.68.

HRMS: Not found.

1-(2-(Cyclohex-1-en-1-yl)ethyl)-4-fluorobenzene (16)

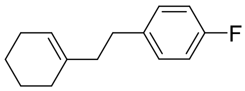

Prepared by **General Procedure D** (60 °C) from 1-(2-(cyclohex-1-en-1-yl)ethyl)-2,4,6-triphenylpyridin-1-ium Tetrafluoroborate (**S-21**) and 1-bromo-4-fluorobenzene on a 0.4 mmol scale. Purification by column chromatography (hexane) resulted in a clear oil (12 mg, 0.06 mmol, 15% yield).

^{1}H NMR (400 MHz, $CDCl_3$) δ 7.19 – 7.07 (m, 2H), 7.01 – 6.90 (m, 2H), 5.40 (br. s, 1H), 2.72 – 2.63 (m, 2H), 2.24 – 2.15 (m, 2H), 2.03 – 1.91 (m, 4H), 1.68 – 1.59 (m, 2H), 1.59 – 1.51 (m, 2H).

^{13}C NMR (101 MHz, $CDCl_3$) δ 161.3 (d, *J* = 242.7 Hz), 138.3 (d, *J* = 3.3 Hz), 137.1, 129.8 (d, *J* = 7.7 Hz), 121.7, 115.0 (d, *J* = 20.9 Hz), 40.2, 33.7, 28.6, 25.4, 23.1, 22.7.

^{19}F NMR (376 MHz, $CDCl_3$) δ -118.1 – -118.2 (m).

FTIR (neat) ν (cm^{-1}) 834, 1225, 1510, 1603, 2931.

HRMS: (GC-EI) *m/z* calculated for $C_{14}H_{17}F$ requires *m/z* 204.1309 ([M+]). Found *m/z* 204.1314.

Tert-butyl 4-(4-fluorophenethyl)piperazine-1-carboxylate (7)

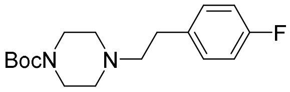

Prepared according to **General Procedure D** (80 °C) from 1-(2-(4-(*tert*-butoxycarbonyl)piperazin-1-yl)ethyl)-2,4,6-triphenylpyridin-1-ium tetrafluoroborate (**S-22**) and 1-bromo-4-fluorobenzene on a 0.2 mmol scale. Purification by column chromatography [Pentane → 3:7 EtOAc:Pentane] resulted in the title compound as a white solid (21 mg, 0.07 mmol, 35% yield).

^{1}H NMR (500 MHz, $CDCl_3$) δ 7.18 – 7.11 (m, 2H), 7.00 – 6.92 (m, 2H), 3.45 (t, *J* = 5.1 Hz, 4H), 2.80 – 2.73 (m, 2H), 2.60 – 2.53 (m, 2H), 2.45 (t, J = 5.1 Hz, 4H), 1.46 (s, 9H).

^{13}C NMR (126 MHz, $CDCl_3$) δ 161.55 (d, *J* = 243.8 Hz), 154.9, 135.9 (d, *J* = 3.2 Hz), 130.2 (d, *J* = 7.8 Hz), 115.3 (d, *J* = 21.2 Hz), 79.8, 60.6, 53.1, 43.7 (br. s), 32.8, 28.6.

^{19}F NMR (470 MHz, $CDCl_3$) δ -117.2 – -117.3 (m).

Spectroscopic data are consistent with the literature known compound.[61]

3-Ethyl 5-methyl 2-((4-fluorophenethoxy)methyl)-6-methyl-4-(*o*-tolyl)-1,4-dihydropyridine-3,5-dicarboxylate (21)

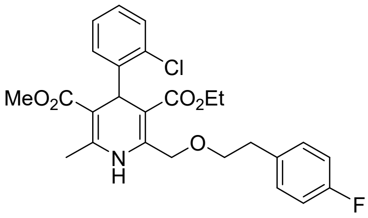

Prepared according to **General Procedure D** (60 °C) from 1-(2-((4-(2-chlorophenyl)-3,5-bis(ethoxycarbonyl)-6-methyl-1,4-dihydropyridin-2-yl)methoxy)ethyl)-2,4,6-triphenylpyridin-1-ium tetrafluoroborate (**S-24**) and 1-bromo-4-fluorobenzene. Purification by column chromatography [Pentane → 80:20 Pentane:EtOAc], resulted in the title compound an orange oil (50 mg, 0.1 mmol, 25% yield).

^{1}H NMR (400 MHz, $CDCl_3$) δ 7.32 (dd, *J* = 7.7, 1.7 Hz, 1H), 7.24 – 7.18 (m, 3H), 7.12 (td, *J* = 7.5, 1.4 Hz, 1H), 7.06 – 6.99 (m, 3H), 6.76 (br. s, 1H), 5.37 (s, 1H), 4.78 – 4.63 (m, 2H), 4.04 (qd, *J* = 7.1, 2.9 Hz, 2H), 3.76 (td, *J* = 6.4, 4.1 Hz, 2H), 3.60 (s, 3H), 2.93 (t, *J* = 6.4 Hz, 2H), 2.13 (s, 3H), 1.17 (t, *J* = 7.1 Hz, 3H).

^{13}C NMR (101 MHz, $CDCl_3$) δ 168.1, 167.3, 161.9 (d, J = 244.5 Hz),145.8, 145.4, 143.9, 134.5 (d, *J* = 3.2 Hz), 132.5, 131.6, 130.4 (d, *J* = 7.8 Hz), 129.4, 127.5, 126.9, 115.6 (d, *J* = 21.3 Hz), 104.0, 101.5, 72.1, 67.8, 59.9, 50.9, 37.4, 35.5, 19.4, 14.4.

^{19}F NMR (377 MHz, $CDCl_3$) δ -116.4 (ddd, *J* = 14.0, 8.8, 5.3 Hz).

FTIR (neat) ν (cm^{-1}) 3392, 3020. 1695, 1511, 1484, 1214, 1101, 759.

HRMS: (ESI+) *m/z* calculated for $C_{26}H_{28}ClFNO_5$ requires *m/z* 488.1635 ($[M+H]^+$). Found *m/z* 488.1625.

Di-*tert*-butyl (((*S*)-1-(tert-butoxy)-6-(4-fluorophenyl)-1-oxohexan-2-yl)carbamoyl)-*L*-glutamate (24)

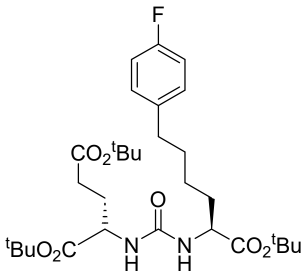

Prepared according to **General Procedure D** on a 0.17 mmol scale at 80 °C from of 1-((*S*)-6-(tert-butoxy)-5-(3-((*S*)-1,5-di-tert-butoxy-1,5-dioxopentan-2-yl)ureido)-6-oxohexyl)-2,4,6-triphenylpyridin-1-ium tetrafluoroborate (**S-35**) and 1-bromo-4-fluorobenzene. Slow addition of 2,4,6-triphenylpyridinium salt to the reaction mixture in half the total reaction volume (1 mL NMP). Purification by column chromatography [Pentane → 40% Et_2O in Pentane] resulted in the title compound as a yellow oil (32 mg, 0.06 mmol, 33% yield).

^{1}H NMR (500 MHz, $CDCl_3$) δ 7.09 (dd, *J* = 8.4, 5.6 Hz, 2H), 6.93 (t, *J* = 8.7 Hz, 2H), 5.10 (d, *J* = 8.0 Hz, 1H), 5.05 (d, *J* = 7.8 Hz, 1H), 4.38 – 4.28 (m, 2H), 2.55 (t, *J* = 7.6 Hz, 2H), 2.30 (ddt, *J* = 19.5, 16.5, 9.9 Hz, 3H), 2.06 (ddt, *J* = 11.2, 9.4, 6.0 Hz, 1H), 1.90 – 1.70 (m, 3H), 1.61 (dh, *J* = 23.7, 7.4 Hz, 3H), 1.46 (s, 9H), 1.42 (s, 9H), 1.42 (s, 9H).

^{13}C NMR (126 MHz, $CDCl_3$) δ 172.6, 172.6, 172.3, 161.3 (d, *J* = 243.0 Hz), 156.8, 138.1 (d, *J* = 3.1 Hz), 129.8 (d, *J* = 7.7 Hz), 115.1 (d, *J* = 21.0 Hz), 82.2, 81.9, 80.7, 53.6, 53.2, 35.0, 33.2, 31.7, 31.3, 30.5, 28.6, 28.2, 28.1, 28.1.

^{19}F NMR (470 MHz, $CDCl_3$) δ -118.1 (ddd, *J* = 14.2, 9.0, 5.5 Hz).

FTIR (thin film) ν (cm^{-1}) 2980, 2926, 2858, 1732, 1369, 1258, 1157, 760, 643, 614.

HRMS: (ESI+) *m/z* calculated for $C_{30}H_{48}FN_2O_7$ requires *m/z* 567.3440 ($[M+H]^+$). Found *m/z* 567.3438.

***N*-(5-(4-Fluorophenyl)pentan-2-yl)-6-methoxyquinolin-8-amine (20)**

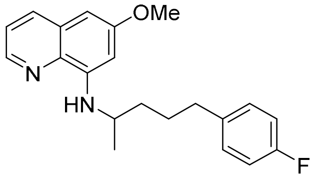

Prepared according to **General Procedure D** from 1-(3-((6-methoxyquinolin-8-yl)amino)butyl)-2,4,6-triphenylpyridin-1-ium tetrafluoroborate (**S-25**) (191 mg, 0.3 mmol, 1.2 eq.), 1-bromo-4-fluorobenzene (29 µL, 0.25 mmol, 1 eq.), and manganese (18 mg, 0.375 mmol, 1.5 eq.) at 80 °C. The reaction concentration of NMP was 0.1 M (2.5 mL). After column chromatography [Column 1: Hexane → 3% EtOAc; Column 2: DCM], the title compound was isolated as a brown oil (31 mg, 0.092 mmol, 31% yield).

^{1}H NMR (400 MHz, $CDCl_3$) δ 8.53 (d, *J* = 4.2 Hz, 1H), 7.92 (d, *J* = 8.2 Hz, 1H), 7.30 (dd, *J* = 8.2, 4.2 Hz, 1H), 7.11 (dd, *J* = 8.4, 5.6 Hz, 2H), 6.93 (t, *J* = 8.7 Hz, 2H), 6.33 (d, *J* = 2.5 Hz, 1H), 6.27 (d, *J* = 2.5 Hz, 1H), 6.01 (br. s, 1H), 3.89 (s, 3H), 3.63 (s, 1H), 2.62 (t, *J* = 6.9 Hz, 2H), 1.85 – 1.69 (m, 3H), 1.69 – 1.56 (m, 1H), 1.29 (d, *J* = 6.3 Hz, 3H).

^{13}C NMR (101 MHz, CDCl$_3$) δ 161.3 (d, *J* = 243.0 Hz), 159.7, 145.1, 144.3, 138.0 (d, *J* = 3.2 Hz), 135.5, 135.0, 130.1, 129.82 (d, *J* = 7.7 Hz), 121.9, 115.1 (d, *J* = 21.1 Hz), 96.9, 91.7, 55.4, 48.2, 36.3, 35.2, 28.2, 20.6.

^{19}F NMR (377 MHz, CDCl$_3$) δ -118.0 (tt, *J* = 9.0, 5.4 Hz).

FTIR (neat) ν (cm^{-1}) 2935, 1617, 1521, 1389, 1221, 1163, 822, 792.

HRMS: (ESI+) *m/z* calculated for $C_{21}H_{24}FN_2O$ requires *m/z* 339.1867 [M+H]$^+$. Found *m/z* 339.1868.

4-Ethyl-1-(4-fluorophenethyl)-1*H*-1,2,3-triazole (8)

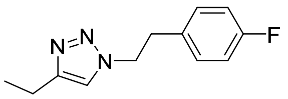

Prepared according to **General Procedure D** from 1-(2-(4-ethyl-1*H*-1,2,3-triazol-1-yl)ethyl)-2,4,6-triphenylpyridin-1-ium tetrafluoroborate (**S-34**) (248.8 mg, 0.4 mmol, 1 eq.), 1-bromo-4-fluorobenzene (46 µL, 0.4 mmol, 1 eq.), and manganese (34 mg, 0.6 mmol, 1.5 eq.) at 60 °C. The reaction concentration of NMP was 0.1 M (4 mL). After column chromatography [Pentane → 1:1 Pentane:EtOAc], the title compound was isolated as a white solid (32 mg, 0.15 mmol, 38% yield).

^{1}H NMR (400 MHz, CDCl$_3$) δ 7.06 – 7.01 (m, 2H), 7.00 (s, 1H), 6.99 – 6.93 (m, 2H), 4.51 (t, *J* = 7.2 Hz, 2H), 3.16 (t, *J* = 7.2 Hz, 2H), 2.70 (q, *J* = 7.6 Hz, 2H), 1.22 (t, *J* = 7.6 Hz, 3H).

^{13}C NMR (101 MHz, CDCl$_3$) δ 162.0 (d, *J* = 245.3 Hz),149.7, 133.0 (d, *J* = 3.3 Hz), 130.3 (d, *J* = 8.0 Hz), 120.7, 115.7 (d, *J* = 21.4 Hz), 51.6, 36.1, 19.1, 13.9.

^{19}F NMR (377 MHz, CDCl$_3$) δ -115.7 (tt, *J* = 8.7, 5.4 Hz).

FTIR (neat) ν (cm^{-1}) 1512, 1224, 1048, 829, 753.

m.p. 79 – 81 °C

HRMS: (ESI+) *m/z* calculated for $C_{12}H_{15}FN_3$ requires *m/z* 220.1245 ([M+H]$^+$). Found *m/z* 220.1236.

3-(4-Fluorophenethyl)pyridine (9)

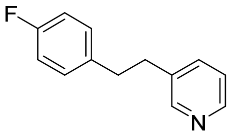

Prepared according to **General Procedure D** from 2,4,6-triphenyl-1-(2-(pyridin-3-yl)ethyl)pyridin-1-ium tetrafluoroborate (**S-27**) (280 mg, 0.56 mmol, 1.4 eq.), 1-bromo-4-fluorobenzene (44 µL, 0.4 mmol, 1 eq.), and manganese (34 mg, 0.6 mmol, 1.5 eq.) at 80 °C. The reaction concentration of NMP was 0.1

M (4 mL). After column chromatography [Hexane → 7:3 Hexane:EtOAc], the title compound was isolated as a clear oil (17 mg, 0.08 mmol, 21% yield).

^{1}H NMR (400 MHz, DMSO-*d*$_6$) δ 8.44 – 8.37 (m, 2H), 7.66 (dt, *J* = 7.8, 2.0 Hz, 1H), 7.32 (dd, *J* = 7.8, 4.8 Hz, 1H), 7.23 (dd, *J* = 8.6, 5.7 Hz, 2H), 7.08 (t, *J* = 8.9 Hz, 2H), 2.89 (s, 4H).

^{13}C NMR (101 MHz, DMSO-*d*$_6$) δ 160.7 (d, *J* = 241.2 Hz), 149.3, 146.8, 137.1 (d, *J* = 3.0 Hz), 136.9, 136.5, 130.2 (d, *J* = 7.9 Hz), 123.5, 114.9 (d, *J* = 20.9 Hz), 35.6, 34.0.

^{19}F NMR (376 MHz, DMSO-*d*$_6$) δ -117.3 – -117.4 (m).

Spectroscopic data are consistent with the literature known compound.[62]

(*E*)-5-Methoxy-1-(4-(trifluoromethyl)phenyl)pentan-1-one *O*-(4-fluorophenethyl) oxime (19)

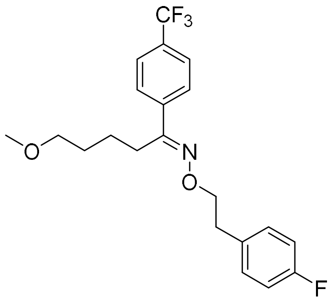

Prepared using **General Procedure D** on a 0.4 mmol scale from (*E*)-1-(2-(((5-methoxy-1-(4-(trifluoromethyl)phenyl) pentylidene)amino)oxy)ethyl)-2,4,6-triphenylpyridin-1-ium tetrafluoroborate (**S-26**) and 1-bromo-4-fluorobenzene. Purification by column chromatography [Column 1: Hexane → 10 % EtOAc; Column 2: Pentane → 10% Et_2O] yielded the title compound as a translucent yellow oil (48 mg, 0.12 mmol, 30% yield).

^{1}H NMR (500 MHz, CDCl$_3$) δ 7.73 (d, *J* = 8.2 Hz, 2H), 7.61 (d, *J* = 8.3 Hz, 2H), 7.20 (dd, *J* = 8.5, 5.5 Hz, 2H), 6.99 (t, *J* = 8.6 Hz, 2H), 4.39 (t, *J* = 6.7 Hz, 2H), 3.36 – 3.29 (m, 5H), 3.02 (t, *J* = 6.8 Hz, 1H), 2.73 (t, *J* = 7.5 Hz, 2H), 1.64 – 1.46 (m, 2H).

{^{19}F}^{13}C NMR (126 MHz, CDCl$_3$) δ 161.7 (d, *J* = 236.7 Hz), 157.4, 139.4, 134.5 (d, *J* = 2.1 Hz), 130.9, 130.5 (d, *J* = 5.4 Hz), 126.7, 125.5, 124.2, 115.3 (d, *J* = 16.2 Hz), 74.9, 72.4, 58.7, 35.1, 29.7, 26.4, 23.2.

n.b. {^{19}F}^{13}C NMR (126 MHz, $CDCl_3$) obtained with o3p set to -60.00 ppm, therefore, Ar-F *J*(^{19}F-^{13}C) is still observed as a doublet.

^{19}F NMR (471 MHz, CDCl$_3$) δ -62.7, -117.2 (ddd, *J* = 14.0, 8.8, 5.3 Hz).

FTIR (thin film) ν (cm^{-1}) 2939, 2872, 2360, 1603, 1326, 1224, 1169, 1127, 1069, 847, 761.

HRMS: (ESI+) *m/z* calculated for $C_{21}H_{24}F_4NO_2$ requires *m/z* 398.1738 ([M+H]$^+$). Found *m/z* 398.1719.

1-(2-(4-Fluorophenyl)-1-(4-methoxyphenyl)ethyl)cyclohexan-1-ol (22)

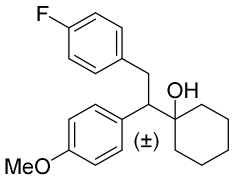

Prepared according to **General Procedure D** on a 0.4 mmol scale from 1-(2-(1-hydroxycyclohexyl)-2-(4-methoxyphenyl)ethyl)-2,4,6-triphenyl pyridin-1-ium tetrafluoroborate (**S-37**) and 1-bromo-4-fluorobenzene at 80 °C. Purification by column chromatography [Pentane → 7:3 Pentane:Et_2O] resulted in an orange oil (9 mg, 0.03 mmol, 7% yield).

^{1}H NMR (400 MHz, $CDCl_3$) δ 7.06 (d, *J* = 8.1 Hz, 2H), 6.93 – 6.83 (m, 2H), 6.83 – 6.72 (m, 4H), 3.76 (s, 3H), 3.24 (dd, *J* = 13.6, 3.2 Hz, 1H), 2.98 – 2.86 (m, 1H), 2.74 (dd, *J* = 11.7, 3.2 Hz, 1H), 1.79 (d, *J* = 13.1 Hz, 1H), 1.65 – 1.55 (m, 3H), 1.54 – 1.40 (m, 4H), 1.39 – 1.34 (m, 2H), 1.25 – 1.09 (m, 1H).

^{13}C NMR (101 MHz, $CDCl_3$) δ 161.1 (d, *J* = 242.7 Hz), 158.3, 137.3 (d, *J* = 3.3 Hz), 132.4, 130.8, 130.3 (d, *J* = 7.6 Hz), 114.8 (d, *J* = 20.9 Hz), 113.5, 73.4, 58.0, 55.2, 36.0, 35.9, 34.8, 25.8, 22.2, 22.1.

{^{1}H}^{19}F NMR (376 MHz, $CDCl_3$) δ -118.2.

FTIR (thin film) ν (cm^{-1}) = 3567, 2938, 1611, 1511, 1249, 760.

HRMS: Not found.

***Tert*-butyl 3-(4-fluorobenzyl)azetidine-1-carboxylate (10)**

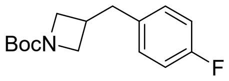

Prepared according to **General Procedure D** on a 0.4 mmol scale from1-((1-(tert-butoxycarbonyl)azetidin-3-yl)methyl)-2,4,6-triphenylpyridin-1-ium tetrafluoroborate (**S-28**) and 1-bromo-4-fluorobenzene at 60 °C. Purification by column chromatography [Pentane → 7:3 Pentane:Et_2O] resulted in a white solid (67 mg, 0.25 mmol, 63% yield).

^{1}H NMR (400 MHz, $CDCl_3$) δ 7.13 – 7.05 (m, 2H), 7.01 – 6.92 (m, 2H), 3.98 (t, *J* = 8.3 Hz, 2H), 3.62 (dd, *J* = 8.6, 5.3 Hz, 2H), 2.87 (d, *J* = 7.9 Hz, 2H), 2.82 – 2.71 (m, 1H), 1.43 (s, 9H).

^{13}C NMR (101 MHz, $CDCl_3$) δ 161.7 (d, *J* = 244.4 Hz), 156.6, 135.1 (d, *J* = 3.3 Hz), 129.9 (d, *J* = 7.9 Hz), 115.5 (d, *J* = 21.2 Hz), 79.5, 54.2, 39.6, 30.1, 28.6.

^{19}F NMR (376 MHz, $CDCl_3$) δ -116.7 – -116.9 (m).

FTIR (thin film) ν (cm^{-1}) 2970, 2885, 1687, 1510, 1413, 1367, 1221, 1159, 1137, 760.

m.p. 77 – 80 °C

HRMS: (ESI+) *m/z* calculated for $C_{15}H_{21}FNO_2$ requires *m/z* 266.1551 [M+H]$^+$. Found *m/z* 266.1552.

Tert-butyl 2-((4R,6R)-6-(4-fluorophenethyl)-2,2-dimethyl-1,3-dioxan-4-yl)acetate (18)

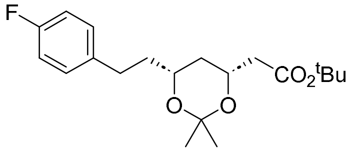

Prepared according to **General Procedure D** on a 0.4 mmol scale from 1-(2-((4*R*,6*R*)-6-(2-(*tert*-butoxy)-2-oxoethyl)-2,2-dimethyl-1,3-dioxan-4-yl)ethyl)-2,4,6-triphenylpyridin-1-ium tetrafluoroborate (**S-29**) and 1-bromo-4-fluorobenzene at 60 °C. Purification by column chromatography [Pentane → 7:3 Pentane:Et_2O] resulted in a clear oil (57 mg, 0.16 mmol, 40% yield).

^{1}H NMR (400 MHz, $CDCl_3$) δ 7.17 – 7.07 (m, 2H), 7.00 – 6.90 (m, 2H), 4.26 – 4.15 (m, 1H), 3.83 – 3.72 (m, 1H), 2.76 – 2.56 (m, 2H), 2.42 (dd, *J* = 15.1, 7.0 Hz, 1H), 2.28 (dd, J = 15.1, 6.2 Hz, 1H), 1.79 (dtd, *J* = 13.8, 8.5, 5.4 Hz, 1H), 1.66 (tdd, *J* = 9.3, 7.6, 4.6 Hz, 1H), 1.53 (dt, *J* = 12.7, 2.5 Hz, 1H), 1.44 (s, 9H), 1.41 (s, 3H), 1.41 – 1.37 (m, 3H), 1.29 – 1.13 (m, 1H).

^{13}C NMR (101 MHz, $CDCl_3$) δ 170.5, 161.4 (d, *J* = 243.3 Hz), 137.7 (d, *J* = 3.3 Hz), 130.0 (d, *J* = 7.7 Hz), 115.2 (d, *J* = 21.1 Hz), 98.9, 80.7, 67.7, 66.4, 42.9, 38.1, 36.7, 30.4, 30.3, 28.2, 19.9.

^{19}F NMR (376 MHz, $CDCl_3$) δ -117.8 – -117.9 (m).

FTIR (thin film) ν (cm^{-1}) 2991, 2941, 1732, 1511, 1381, 1369, 1261, 1222,1202, 1159, 953, 843, 763, 652.

HRMS: Not found.

(1S,2S,5S)-2-(4-Fluorobenzyl)-6,6-dimethylbicyclo[3.1.1]heptane (17)

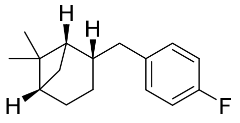

Prepared according to **General Procedure D** on a 0.4 mmol scale from1-(((1*S*,2*R*,5*S*)-6,6-dimethylbicyclo[3.1.1]heptan-2-yl)methyl)-2,4,6-triphenylpyridin-1-ium tetrafluoroborate (**S-30**) and 1-bromo-4-fluorobenzene at 80 °C. Purification by column chromatography [Hexane] resulted in a clear oil (41 mg, 0.18 mmol, 44% yield).

^{1}H NMR (400 MHz, $CDCl_3$) δ 7.09 (ddd, *J* = 8.4, 5.3, 2.5 Hz, 2H), 6.95 (t, *J* = 8.8 Hz, 2H), 2.73 – 2.58 (m, 2H), 2.35 – 2.25 (m, 3H), 1.98 – 1.81 (m, 4H), 1.60 – 1.49 (m, 1H), 1.20 (s, 3H), 1.13 (s, 3H), 0.85 (d, *J* = 9.6 Hz, 1H).

^{13}C NMR (101 MHz, $CDCl_3$) δ 161.3 (d, *J* = 242.9 Hz), 137.6 (d, *J* = 3.2 Hz), 130.4 (d, *J* = 7.7 Hz), 115.0 (d, *J* = 21.0 Hz), 45.3, 43.5, 43.5, 42.7, 41.6, 38.9, 33.9, 28.3, 26.6, 22.4.

^{19}F NMR (376 MHz, $CDCl_3$) δ -118.2 (tt, *J* = 8.8, 5.5 Hz).

FTIR (thin film) ν (cm^{-1}) = 3038, 2985, 2942, 2913, 1603, 1510, 1223, 825.

HRMS: Not found.

5-(4-Fluorophenethyl)benzo[*d*][1,3]dioxole (15)

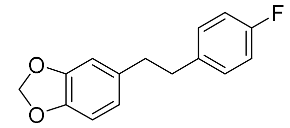

Prepared according to **General Procedure D** on a 0.4 mmol scale from 1-(2-(benzo[*d*][1,3]dioxol-5-yl)ethyl)-2,4,6-triphenylpyridin-1-ium tetrafluoroborate (**S-31**) and 1-bromo-4-fluorobenzene at 60 °C. Purification by column chromatography [Pentane → 95:5 Pentane:DCM] resulted in a white solid (19 mg, 78 μmol, 19% yield).

^{1}H NMR (400 MHz, CDCl$_3$) δ 7.09 (ddd, *J* = 8.3, 5.3, 2.5 Hz, 2H), 6.98 – 6.90 (m, 2H), 6.71 (d, *J* = 7.9 Hz, 1H), 6.65 (d, *J* = 1.5 Hz, 1H), 6.58 (dd, *J* = 7.9, 1.7 Hz, 1H), 5.92 (s, 2H), 2.90 – 2.76 (m, 4H).

^{13}C NMR (101 MHz, CDCl$_3$) δ 161.5 (d, *J* = 243.5 Hz), 147.7, 145.9, 137.3 (d, *J* = 3.1 Hz), 135.4, 130.0 (d, *J* = 7.8 Hz), 121.4, 115.2 (d, *J* = 21.1 Hz), 109.1, 108.3, 100.9, 37.9, 37.5.

^{19}F NMR (376 MHz, CDCl$_3$) δ -117.6 (ddd, *J* = 14.3, 8.8, 5.5 Hz).

FTIR (thin film) ν (cm^{-1}) 2362, 2342, 1509, 1491, 1246, 1042, 827, 765, 645.

m.p. 66 – 71 °C

HRMS: Not found.

2-Fluoro-5-phenethylpyridine (S-14)

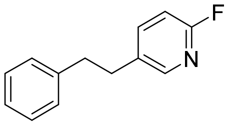

Prepared using **General Procedure D** on a 0.4 mmol scale from 1-phenethyl-2,4,6-triphenylpyridin-1-ium tetrafluoroborate (**2**) and 2-fluoro-5-iodopyridine at 80 °C with slow addition. Purification by column chromatography [Pentane → 8:2 Pentane:Et$_2$O] resulted in the title compound as a yellow oil (15 mg, 80 μmol, 19% yield).

^{1}H NMR (400 MHz, CDCl$_3$) δ 7.97 (s, 1H), 7.50 (td, *J* = 8.1, 2.5 Hz, 1H), 7.28 (*overlaps with CDCl$_3$,* t, *J* = 6.6 Hz, 2H), 7.20 (dd, *J* = 8.5, 6.1 Hz, 1H), 7.16 – 7.08 (m, 2H), 6.82 (dd, J = 8.4, 2.9 Hz, 1H), 2.92 (s, 4H).

^{13}C NMR (101 MHz, CDCl$_3$) δ 162.5 (d, *J* = 237.1 Hz), 147.3 (d, *J* = 14.4 Hz), 141.3 (d, *J* = 7.8 Hz), 140.6, 134.5 (d, *J* = 4.6 Hz), 128.6, 128.6, 126.4, 109.1 (d, *J* = 37.4 Hz), 37.6, 34.0.

^{19}F NMR (376 MHz, CDCl$_3$) δ -72.0 (d, *J* = 7.9 Hz).

FTIR (thin film) ν (cm^{-1}) = 2926, 2360, 1598, 1486, 1395, 1249.

HRMS: (ESI+) *m/z* calculated for $C_{13}H_{13}FN$ requires *m/z* 202.1027 [M+H]$^+$. Found *m/z* 202.1023.

1-Fluoro-2-phenethylbenzene (S-17)

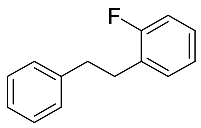

Prepared according to **General Procedure D** on a 0.4 mmol scale from 1-phenethyl-2,4,6-triphenylpyridin-1-ium tetrafluoroborate (**2**) (280 mg, 0.56 mmol, 1.4 eq.) and 1-bromo-2-fluorobenzene (44 μL, 0.4 mmol, 1 eq.) at 60 °C. Purification by column chromatography [100% Pentane] resulted in the title compound as a colourless crystalline solid (39 mg, 0.2 mmol, 49% yield).

^{1}H NMR (400 MHz, CDCl$_3$) δ 7.31 – 7.22 (m, 2H), 7.22 – 7.06 (m, 5H), 7.03 – 6.95 (m, 2H), 2.90 (dq, *J* = 9.2, 4.5 Hz, 4H).

^{13}C NMR (101 MHz, CDCl$_3$) δ 161.3 (d, *J* = 244.8 Hz), 141.7, 130.8 (d, *J* = 5.1 Hz), 128.8, 128.5, 128.5 (d, *J* = 11.4 Hz), 127.8 (d, *J* = 8.1 Hz), 126.1, 124.0 (d, *J* = 3.6 Hz), 115.3 (d, *J* = 22.1 Hz), 36.6, 31.4.

^{19}F NMR (376 MHz, CDCl$_3$) δ -118.9 – -119.0 (m).

FTIR (thin film) ν (cm^{-1}) = 2930, 2360, 2341, 1491, 1227, 760, 699.

m.p. 35 – 37 °C

HRMS: Not found.

***N*-(4-Fluorophenethyl)-4,6-dimethoxypyrimidin-2-amine (25)**

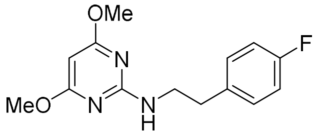

To a solution of 2-(4-fluorophenyl)ethan-1-amine (80 μL, 0.6 mmol, 1 eq.) in isopropanol (1 mL) was added triethylamine (0.3 mL, 2 mmol, 3 eq.) and 2-chloro-4,6-dimethoxy-pyrimidine (100 mg, 0.6 mmol, 1 eq.). The reaction mixture was stirred at reflux (120 °C) for 12 h. Purification by column chromatography [heptane → EtOAc] resulted in the title compound as a white solid (84 mg, 0.30 mmol, 50% yield).

^{1}H NMR (400 MHz, CDCl$_3$ + 0.03% TMS) δ 7.17 (ddd, *J* = 8.4, 5.3, 2.6 Hz, 2H), 7.04 – 6.94 (m, 2H), 5.41 (s, 1H), 4.95 (s, 1H), 3.85 (s, 6H), 3.68 – 3.58 (m, 2H), 2.88 (t, *J* = 7.1 Hz, 2H).

^{13}C NMR (101 MHz, CDCl$_3$) δ 172.4, 161.8, 161.7 (d, *J* = 244.1 Hz), 135.2 (d, *J* = 3.5 Hz), 130.3 (d, *J* = 7.9 Hz), 115.5 (d, *J* = 21.1 Hz), 78.9, 53.7, 43.0, 43.0, 35.4.

^{19}F NMR (377 MHz, CDCl$_3$) δ -116.9 – -117.1 (m).

FTIR (thin film) ν (cm^{-1}) = 2981, 2625, 1621, 1585, 1479, 1450, 1357, 1056, 751, 706.

m.p. 93 – 94 °C

HRMS: (ESI+) *m/z* calculated for $C_{14}H_{17}FN_3O_2$ requires *m/z* 278.1299 [M+H]$^+$. Found *m/z* 278.1302.

2-(4-(4-Fluorophenyl)piperidin-1-yl)pyrimidine (32)

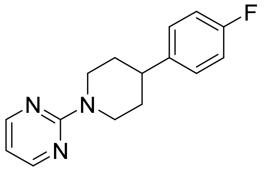

This procedure was adapted from MacMillan and co-workers.[63]

In an N_2 glove box, to a 20 mL screwcap vial charged with teflon magnetic stirring bar, was added 1-pyrimidin-2-ylpiperidin-4-ol (100 mg, 0.56 mmol, 1.75 eq.) followed by anhydrous dioxane (5 mL). 5,7-Di-tert-butyl-3-phenylbenzo[*d*]oxazol-3-ium trifluoromethanesulfonate (146 mg, 0.32 mmol, 1 eq,) was added portion-wise and the mixture was stirred for 10 minutes.

In a separate 20 mL screwcap vial charged with teflon magnetic stirring bar, a mixture of 1-bromo-4-fluorobenzene (35.0 µL, 0.32 mmol, 1 eq.), quinuclidine (62 mg, 0.56 mmol, 1.75 eq.), (4,4'-di-*tert*-butyl-2,2'-bipyridine)bis[(2-pyridinyl)phenyl]iridium(iii) hexafluorophosphate (4.4 mg, 4.78 µmol, 0.015 eq.), bis(1,1-dimethylethyl)-2,2'-bipyridine] nickel (II) dibromide (7.8 mg, 16 µmol, 0.05 eq.) and phthalimde (47 mg, 319 µmol, 1 eq.) in anhydrous DMSO (5.0 mL) was prepared. The first solution, containing the activated alcohol was added to the second solution containing the aryl bromide. The reaction vial was sealed with a septum-lined vial cap. The reaction mixture was irradiated on Lumidox II 450 nm LED modules for 2 h. The crude reaction mixture was concentrated under reduced pressure and then redissolved in EtOAc (5 mL) and washed with brine (3 x 5 mL). The mixture was dried over Na_2SO_4, filtered, and the solvent was removed under reduced pressure. Purification by column chromatography [heptane → EtOAc] resulted in the title compound as a clear oil (23 mg, 0.09 mmol, 28% yield).

^{1}H NMR (400 MHz, $CDCl_3$ + 0.03% TMS) δ 8.32 (d, *J* = 4.7 Hz, 2H), 7.17 (ddd, *J* = 8.4, 5.3, 2.5 Hz, 2H), 7.04 – 6.93 (m, 2H), 6.47 (t, *J* = 4.7 Hz, 1H), 4.92 (dt, *J* = 13.4, 2.2 Hz, 2H), 2.95 (td, *J* = 13.2, 2.5 Hz, 2H), 2.78 (tt, *J* = 12.2, 3.6 Hz, 1H), 1.97 – 1.87 (m, 2H), 1.66 (qd, *J* = 12.8, 4.2 Hz, 2H).

^{13}C NMR (101 MHz, $CDCl_3$ + 0.03% TMS) δ 161.8, 161.6 (d, *J* = 244.1 Hz), 157.9, 141.8 (d, *J* = 3.8 Hz), 128.3 (d, J = 8.1 Hz), 115.3 (d, *J* = 20.9 Hz), 109.7, 44.6, 42.5, 33.5.

^{19}F NMR (377 MHz, $CDCl_3$ + 0.03% TMS) δ -117.0 – -117.2 (m).

FTIR (thin film) ν (cm^{-1}) = 2933, 1585, 1546, 1509, 1459, 1361, 1229, 980, 833, 797, 774.

HRMS: (ESI+) *m/z* calculated for $C_{15}H_{17}FN_3$ requires *m/z* 258.1401 $[M\text{-}BF_4^-]^+$. Found *m/z* 258.1409.

1-Fluoro-4-(4-phenylbutan-2-yl)benzene (30)

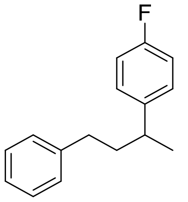

Method adapted from literature procedure.[23]

To a 20 mL crimp-cap vial charged with a stirrer bar was added 2,4,6-triphenyl-1-(4-phenylbutan-2-yl)pyridin-1-ium tetrafluoroborate (137 mg, 0.26 mmol, 1.3 equiv.), $NiBr_2$·glyme (6.2 mg, 20 µmol, 10 mol%), 4,4'-dimethoxy-2,2'-bipyridine (4.8 mg, 22 µmol, 11 mol%) and diethyl 1,4-dihydro-2,6-dimethyl-3,5-pyridinedicarboxylate (101 mg, 0.40 mmol, 2.0 equiv.). The vial was sealed with a septum cap and DMA (2.0 mL, 0.1 M) and 1-bromo-4-fluorobenzene (35 mg, 0.20 mmol, 1.0 equiv.) were added sequentially *via* syringe. The reaction vial was irradiated by blue LEDs (HepatoChem PhotoRedOx Box, equipped with an EvoluChem 18W blue LED lamp, λ = 450 nm) and stirred for 14 h with fan cooling. After this time had elapsed, the reaction mixture was diluted with EtOAc (10 mL) and washed with brine (2 x 5 mL). The combined organics were dried over $MgSO_4$, filtered, and removed under reduced pressure. The crude residue was purified by flash silica gel column chromatography [hexane → hexane:EtOAc, 50:1] resulted in the title compound as a colourless oil (18 mg, 78 µmol, 39% yield).

^{1}H NMR (500 MHz, $CDCl_3$) δ 7.30 – 7.23 (m, 2H), 7.20 – 7.08 (m, 5H), 7.03 – 6.95 (m, 2H), 2.77 – 2.66 (m, 1H), 2.56 – 2.45 (m, 2H), 1.89 (q, *J* = 7.6 Hz, 2H), 1.26 (d, *J* = 7.0 Hz, 3H).

^{13}C NMR (126 MHz, $CDCl_3$) δ 161.4 (d, *J* = 243.3 Hz), 143.0 (d, *J* = 2.8 Hz), 142.5, 128.5, 128.5, 128.5, 125.9, 115.2 (d, *J* = 20.9 Hz), 40.2, 38.9, 34.0, 22.8.

^{19}F NMR (471 MHz, $CDCl_3$) δ -117.6 – -117.7 (m).

HRMS: (GC-EI) m/z calculated for $C_{16}H_{17}F$ requires m/z 228.1309 $[M]^+$. Found m/z 228.1314.

The spectral data match that previously published in literature.[64]

Scheme S15: Multistep synthesis of 1-(2-fluoroethoxy)-4-phenethylbenzene (**S-16**)

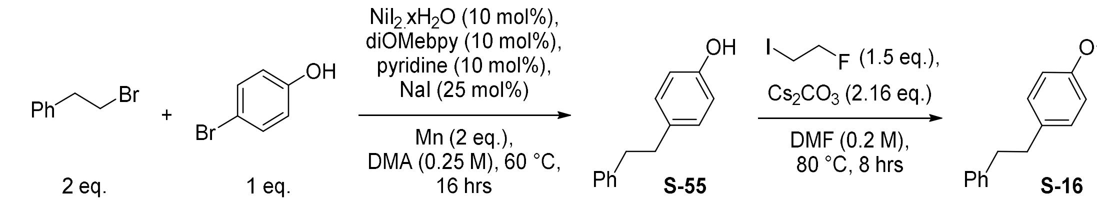

4-Phenethylphenol (S-55)

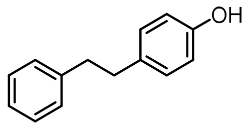

The title compound was prepared following **General Procedure E** on a 1 mmol scale from (2-bromoethyl)benzene (272 µL, 2 mmol, 2 eq.) and 4-bromophenol (173 mg, 1 mmol, 1 eq.). Purification by column chromatography [DCM] resulted in a white solid (103 mg, 0.52 mmol, 52% yield).

^{1}H NMR (400 MHz, $CDCl_3$) δ 7.31 – 7.25 (m, 3H), 7.20 – 7.15 (m, 2H), 7.06 – 7.01 (m, 2H), 6.78 – 6.73 (m, 2H), 2.88 (p, *J* = 3.3 Hz, 4H).

^{13}C NMR (101 MHz, $CDCl_3$) δ 153.8, 141.9, 134.2, 129.7, 128.6, 128.4, 126.0, 115.3, 38.3, 37.2.

The spectral data match that previously published in literature.[65]

1-(2-Fluoroethoxy)-4-phenethylbenzene (S-16)

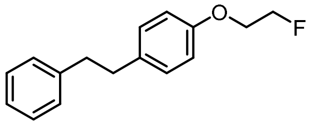

4-phenethylphenol (**S-55**) (74 mg, 0.37 mmol, 1 eq.) and Cs_2CO_3 (261 mg, 0.8 mmol, 2.16 eq.) were cycled under N_2 in a Schlenk flask. DMF (2 mL, 0.2 M) was added followed by 1-fluoro-2-iodoethane (50 µL, 0.6 mmol, 1.6 eq.). The flask was sealed and heated to 80 °C for 8 h. Upon completion, the reaction mixture was diluted with EtOAc (10 mL) and washed with brine (3 x 10 mL). The organics were dried over Na_2SO_4, filtered and solvent removed under reduced pressure. Purification by column chromatography [DCM] resulted in a white solid (73 mg, 0.3 mmol, 81% yield).

^{1}H NMR (500 MHz, $CDCl_3$) δ 7.28 (m, 2H, *overlaps with $CDCl_3$*), 7.18 (dd, *J* = 15.7, 7.2 Hz, 3H), 7.09 (d, *J* = 8.5 Hz, 2H), 6.85 (d, *J* = 8.6 Hz, 2H), 4.85 – 4.59 (m, 2H), 4.20 (dt, *J* = 27.8, 4.2 Hz, 2H), 2.87 (s, 4H).

^{13}C NMR (126 MHz, $CDCl_3$) δ 156.8, 141.9, 134.7, 129.6, 128.6, 128.5, 126.0, 114.7, 82.2 (d, *J* = 170.5 Hz), 67.3 (d, *J* = 20.6 Hz), 38.3, 37.2.

^{19}F NMR (377 MHz, $CDCl_3$) δ -223.9 (tt, *J* = 47.6, 27.8 Hz).

FTIR (thin film) ν (cm^{-1}) = 3028, 1512, 1454, 1247, 1079, 1055, 924, 824, 701.

m.p. 36 – 38 °C

HRMS: Not found.

1-Fluoro-4-phenethylbenzene (3)

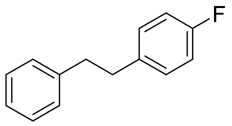

The title compound was prepared following **General Procedure E** on a 2 mmol scale from (2-iodoethyl)benzene (290 µL, 3 mmol, 1.5 eq.) and 1-fluoro-4-iodobenzene (230 µL, 2 mmol, 1 eq.) Purification by column chromatography [hexane] resulted in a crystalline white solid (206 mg, 1.03 mmol, 52% yield).

Spectroscopic data are consistent with the literature known compound.[65]

^{1}H NMR (500 MHz, $CDCl_3$) δ 7.28 (t, J = 7.4 Hz, 2H), 7.21 (d, J = 7.4 Hz, 1H), 7.16 (d, J = 8.4 Hz, 2H), 7.11 (d, J = 7.4 Hz, 2H), 6.96 (t, J = 8.8 Hz, 2H), 2.90 (s, 4H).

^{13}C NMR (126 MHz, $CDCl_3$) δ 161.5 (d, J = 243.4 Hz), 141.6, 137.4 (d, J = 3.2 Hz), 130.0 (d, J = 7.8 Hz), 128.6 (d, J = 15.3 Hz), 126.1, 125.8, 115.2 (d, J = 21.2 Hz), 38.2, 37.2.

^{19}F NMR (470 MHz, $CDCl_3$) δ -117.6 (tt, J = 9.4, 5.3 Hz).

1-Phenethyl-4-(trifluoromethyl)benzene (S-15)

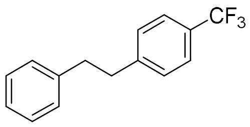

The title compound was prepared following **General Procedure E** on a 2 mmol scale from (2-bromoethyl)benzene (350 µL, 2.4 mmol, 1.2 eq.) and 4-iodotrifluoromethylbenzene (300 µL , 2 mmol, 1 eq.). Purification by column chromatography [hexane] resulted in a white solid (318 mg, 1.27 mmol, 64% yield).

^{1}H NMR (400 MHz, $CDCl_3$) δ 7.50 (d, J = 8.0 Hz, 2H), 7.30 – 7.10 (m, 7H), 3.00 – 2.85 (m, 4H).

{^{19}F}^{13}C NMR (126 MHz, $CDCl_3$) δ 145.8, 141.1, 128.9, 128.5, 128.4, 127.7, 126.2, 125.3, 123.4, 37.7, 37.6.

^{19}F NMR (376 MHz, $CDCl_3$) δ -62.2 (s).

Spectroscopic data are consistent with the literature known compound.[66]

7-(4-(4-Fluorophenethyl)piperazine-1-carbonyl)-1*H*-indole-3-carbonitrile (27)

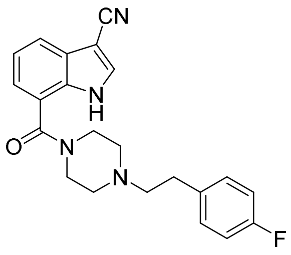

Prepared as previously described in literature on a 0.5 mmol scale.[57] Purification by column chromatography [EtOAc → 5% MeOH in EtOAc] resulted in a beige solid (121 mg, 0.32 mmol, 64% yield).

^{1}H NMR (400 MHz, $CDCl_3$) δ 10.32 (s, 1H), 7.96 (d, J = 7.8 Hz, 1H), 7.80 (s, 1H), 7.46 (d, J = 7.3 Hz, 1H), 7.43 – 7.37 (m, 2H), 7.30 – 7.22 (m, 1H), 7.09 (t, J = 8.5 Hz, 2H), 3.91 (s, 4H), 2.95 – 2.86 (m, 2H), 2.74 (dd, J = 16.0, 8.9 Hz, 7H).

{^{1}H}^{19}F NMR (377 MHz, $CDCl_3$) δ -117.0 (s).

FTIR (thin film) ν (cm^{-1}) = 3227, 3015, 2816, 2224, 1625, 1529, 1437, 1294, 1219, 761.

m.p. 153 – 157 °C

HRMS: (ESI+) *m/z* calculated for $C_{22}H_{22}FN_4O$ requires *m/z* 377.1772 $[M+H]^+$. Found *m/z* 377.1780.

5-(3-Chloro-4-methoxyphenyl)-*N*-(3-(4-fluorophenyl)propyl)oxazole-4-carboxamide (41)

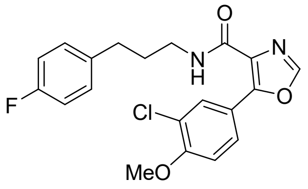

The title compound was prepared following **General Procedure F** on a 0.42 mmol scale from 5-(3-chloro-4-methoxyphenyl)oxazole-4-carboxylic acid (**S-46**) (1 eq.) and 3-(4-fluorophenyl)propan-1-amine.HCl (1.2 eq.). Purification by column chromatography [10% EtOAc in hexane → 30% EtOAc in hexane] resulted in a white solid (113 mg, 0.29 mmol, 69% yield).

^{1}H NMR (500 MHz, CDCl$_3$) δ 8.38 (dd, *J* = 8.7, 2.1 Hz, 1H), 8.33 (d, *J* = 2.1 Hz, 1H), 7.79 (s, 1H), 7.28 (s, 1H), 7.15 (dd, *J* = 8.3, 5.5 Hz, 2H), 7.03 – 6.92 (m, 3H), 3.95 (s, 3H), 3.46 (q, *J* = 6.8 Hz, 2H), 2.69 (t, *J* = 7.7 Hz, 2H), 1.94 (p, *J* = 7.4 Hz, 2H).

^{13}C NMR (126 MHz, CDCl$_3$) δ 161.5 (d, *J* = 243.6 Hz), 161.2, 156.4, 151.6, 147.7, 137.1 (d, *J* = 3.2 Hz), 130.0, 129.8 (d, *J* = 7.8 Hz), 128.6, 128.3, 122.7, 120.6, 115.3 (d, *J* = 21.1 Hz), 111.7, 56.4, 38.9, 32.6, 31.5.

^{19}F NMR (471 MHz, CDCl$_3$) δ -117.6 (ddd, *J* = 14.2, 8.9, 5.4 Hz).

FTIR (thin film) ν (cm^{-1}) = 3414, 2945, 1665, 1603, 1223, 822, 761.

m.p. 106 – 108 °C

HRMS: (ESI+) m/z calculated for $C_{20}H_{19}ClFN_2O_3$ requires m/z 389.1063 $[M+H]^+$. Found m/z 389.1055.

5-(3-Chloro-4-methoxyphenyl)-*N*-(3-(2-fluorophenyl)propyl)oxazole-4-carboxamide (42)

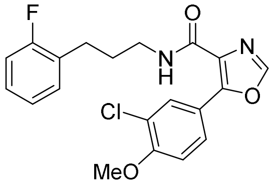

The title compound was prepared following **General Procedure F** on a 0.42 mmol scale from 5-(3-chloro-4-methoxyphenyl)oxazole-4-carboxylic acid (**S-46**) (1 eq.) and 3-(2-fluorophenyl)propan-1-amine (1.2 eq.). Purification by column chromatography [10% EtOAc in hexane → 30% EtOAc in hexane] resulted in a white solid (117 mg, 0.3 mmol, 72% yield).

^{1}H NMR (400 MHz, CDCl$_3$) δ 8.38 (dd, *J* = 8.7, 2.2 Hz, 1H), 8.33 (d, *J* = 2.2 Hz, 1H), 7.80 (s, 1H), 7.35 (s, 1H), 7.24 – 7.13 (m, 2H), 7.09 – 7.02 (m, 1H), 7.00 (d, *J* = 8.8 Hz, 2H), 3.95 (s, 3H), 3.47 (q, *J* = 6.9 Hz, 2H), 2.75 (t, *J* = 7.6 Hz, 2H), 1.96 (p, *J* = 7.3 Hz, 2H).

^{13}C NMR (101 MHz, CDCl$_3$) δ 161.3 (d, *J* = 244.5 Hz), 161.2, 156.3, 151.6, 147.7, 130.8 (d, *J* = 5.0 Hz), 130.0, 128.6, 128.4, 128.3 (d, *J* = 15.9 Hz), 127.9 (d, *J* = 8.1 Hz), 124.2 (d, *J* = 3.6 Hz), 122.7, 120.6, 115.4 (d, *J* = 22.2 Hz), 111.7, 56.4, 38.9, 30.1, 26.6.

^{19}F NMR (376 MHz, CDCl$_3$) δ -118.7 – -118.8 (m).

FTIR (thin film) ν (cm-1) = 3414, 3015, 1666, 1522, 1494, 1229, 1263, 1068, 761.

m.p. 96 – 99 °C

HRMS: (ESI+) m/z calculated for $C_{20}H_{19}ClFN_2O_3$ requires m/z 389.1063 [M+H]$^+$. Found m/z 389.1055.

5-(3-Chloro-4-methoxyphenyl)-*N*-(3-(3-chloro-5-fluorophenyl)propyl)oxazole-4-carboxamide (43)

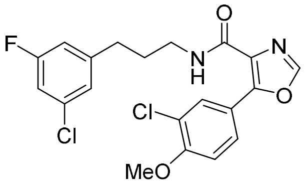

NiBr$_2$.glyme (7 mg, 22 µmol, 12 mol%), Zinc (22 mg, 0.34 mmol, 1.9 eq.), PyBCAM (7.2 mg, 30 µmol, 17 mol%) and 1-(3-(5-(3-chloro-4-methoxyphenyl)oxazole-4-carboxamido)propyl)-2,4,6-triphenyl pyridin-1-ium tetrafluoroborate (**33**) (144 mg, 0.22 mmol, 1.2 eq.), were added to a vial with subsequent addition of NMP (1.8 mL, 0.1M). The mixture was sealed with a septum, fitted with vent needle and was degassed by sparging with N_2 from a balloon while sonicating for 30 seconds. The vent needle was removed and 1-chloro-3-fluoro-5-iodobenzene (23 µL, 46 mg, 0.18 mmol, 1 eq.) was added under N_2. The N_2 balloon was removed, the septum wrapped in Teflon tape and parafilm and the vial heated to 110 °C for 16 h. The reaction mixture was then diluted with EtOAc (5 mL) and washed with brine (3 x 5 mL). The organic phase was dried over Na_2SO_4, filtered, and solvent removed under reduced pressure. Purification by column chromatography [DCM] resulted in a yellow solid (10 mg, 23 µmol, 13% yield).

^{1}H NMR (500 MHz, CDCl$_3$) δ 8.37 (dd, *J* = 8.7, 2.2 Hz, 1H), 8.33 (d, *J* = 2.2 Hz, 1H), 7.80 (s, 1H), 7.31 (s, 1H), 7.04 – 6.96 (m, 2H), 6.91 (dt, *J* = 8.5, 2.1 Hz, 1H), 6.82 (dd, *J* = 9.3, 1.7 Hz, 1H), 3.95 (s, 3H), 3.47 (q, *J* = 6.9 Hz, 2H), 2.72 – 2.65 (m, 2H), 1.95 (p, *J* = 7.5 Hz, 2H).

^{13}C NMR (126 MHz, CDCl$_3$) δ 162.9 (d, *J* = 249.1 Hz), 161.3, 156.4, 151.7, 147.7, 145.3 (d, *J* = 8.1 Hz), 135.0 (d, *J* = 11.0 Hz), 130.0, 128.6, 128.2, 124.6 (d, *J* = 3.0 Hz), 122.7, 120.5, 114.1 (d, *J* = 12.9 Hz), 113.9 (d, *J* = 9.1 Hz), 111.8, 38.8, 33.1, 30.9, 28.5.

^{19}F NMR (470 MHz, CDCl$_3$) δ -111.5 (t, *J* = 8.8 Hz).

FTIR (thin film) ν (cm^{-1}) = 2883, 2360, 1665, 1587, 1520, 1442, 1263, 1068, 851, 737, 640.

m.p. 120 – 124 °C

HRMS: (ESI+) *m/z* calculated for $C_{20}H_{18}Cl_2FN_2O_3$ requires *m/z* 423.0673 $[M+H]^+$. Found *m/z* 423.0667.

Tert-butyl (3-(4-(2-fluoroethoxy)phenyl)propyl)carbamate (S-56)

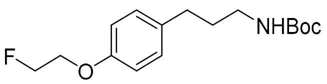

The title compound was prepared following **General Procedure E** on a 1 mmol scale from *tert*-butyl (3-bromopropyl)carbamate (1 eq.) and 1-(2-fluoroethoxy)-4-iodobenzene (**36**) (1 eq.). Purification by column chromatography [DCM → 3% Et_2O in DCM] resulted in a white crystalline solid (81 mg, 0.27 mmol, 27% yield).

^{1}H NMR (400 MHz, $CDCl_3$) δ 7.12 – 7.07 (m, 2H), 6.87 – 6.83 (m, 2H), 4.88 – 4.64 (m, 2H), 4.51 (br. s, 1H), 4.27 – 4.12 (m, 2H), 3.16 – 3.11 (m, 2H), 2.62 – 2.55 (m, 2H), 1.77 (p, *J* = 7.5 Hz, 2H), 1.44 (s, 9H).

^{13}C NMR (101 MHz, $CDCl_3$) δ 156.8, 156.1, 134.4, 129.5, 114.8, 82.1 (d, *J* = 170.5 Hz), 79.3, 67.3 (d, *J* = 20.6 Hz), 40.3, 32.3, 32.0, 28.5.

^{19}F NMR (377 MHz, $CDCl_3$) δ -223.9 (tt, *J* = 47.5, 27.8 Hz).

FTIR (thin film) ν (cm^{-1}) = 3347, 2928, 1705, 1511, 1367, 1254, 1176, 766.

m.p. 67 – 69 °C

HRMS: (ESI+) *m/z* calculated for $C_{16}H_{25}FNO_3$ requires *m/z* 298.1813 $[M+H]^+$. Found *m/z* 298.1808.

5-(3-Chloro-4-methoxyphenyl)-_N_-(3-(4-(2-fluoroethoxy)phenyl)propyl)oxazole-4-carboxamide (40)

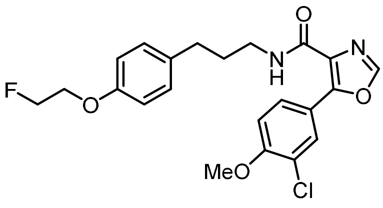

In a one-necked flask under ambient atmosphere, *tert*-butyl (3-(4-(2-fluoroethoxy)phenyl)propyl)carbamate (**S-56**) (59 mg, 0.2 mmol, 1 eq.) was dissolved in DCM (300 μL) and the resulting solution cooled to 0 °C. HCl in dioxane (300 μL, 4 M, 1.2 mmol, 6 eq.) was added. After 15 minutes, the cooling bath was removed and the mixture stirred for 2 h at room temperature. After this time, the solvent was removed under reduced pressure and the resulting hydrochloride salt carried forward to the next step without further purification.

The title compound was prepared following **General Procedure F** on a 0.2 mmol scale from 5-(3-chloro-4-methoxyphenyl)oxazole-4-carboxylic acid (**S-46**) (1 eq.) and 3-(4-(2-fluoroethoxy)phenyl)propan-1-amine hydrochloride (1 eq.). Purification by column

chromatography [10% EtOAc in hexane → 50% EtOAc in hexane] resulted in a white solid (49 mg, 0.11 mmol, 57% yield).

^{1}H NMR (400 MHz, CDCl$_3$) δ 8.38 (dd, *J* = 8.7, 2.2 Hz, 1H), 8.33 (d, *J* = 2.2 Hz, 1H), 7.79 (s, 1H), 7.27 (br. s, 1H), 7.16 – 7.08 (m, 2H), 7.00 (d, *J* = 8.8 Hz, 1H), 6.89 – 6.81 (m, 2H), 4.88 – 4.64 (m, 2H), 4.19 (ddd, *J* = 27.8, 4.9, 3.6 Hz, 2H), 3.95 (s, 3H), 3.46 (q, *J* = 7.0 Hz, 2H), 2.71 – 2.63 (m, 2H), 1.93 (p, *J* = 7.5 Hz, 2H).

^{13}C NMR (101 MHz, CDCl$_3$) δ 161.2, 156.9, 156.4, 151.6, 147.7, 134.3, 130.0, 129.5, 128.6, 128.4, 122.7, 120.6, 114.8, 111.8, 82.2 (d, *J* = 170.3 Hz), 67.3 (d, *J* = 20.6 Hz), 56.4, 39.0, 32.6, 31.5.

^{19}F NMR (377 MHz, CDCl$_3$) δ -223.9 (tt, *J* = 47.6, 27.9 Hz).

FTIR (thin film) ν (cm^{-1}) = 3404, 2925, 1666, 1512, 1262, 1069, 764.

m.p. 129 – 130 °C

HRMS: (ESI+) *m/z* calculated for $C_{22}H_{23}ClFN_2O_4$ requires *m/z* 433.1325 [M+H]$^+$. Found *m/z* 433.1314.

***Tert*-butyl (3-(6-fluoropyridin-3-yl)propyl)carbamate (S-57)**

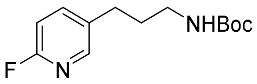

The title compound was prepared following **General Procedure E** on a 1 mmol scale from *tert*-butyl (3-bromopropyl)carbamate (1 eq.) and 5-fluoro-2-iodopyridine (1 eq.). Purification by column chromatography [DCM → 30% Et_2O in DCM] resulted in a clear oil (162 mg, 0.64 mmol, 32% yield).

^{1}H NMR (500 MHz, CDCl$_3$) δ 8.01 (s, 1H), 7.59 (td, *J* = 8.1, 2.3 Hz, 1H), 6.84 (dd, *J* = 8.3, 2.8 Hz, 1H), 4.61 (br. s, 1H), 3.15 (d, *J* = 6.2 Hz, 2H), 2.66 – 2.59 (m, 2H), 1.79 (dt, *J* = 14.3, 7.0 Hz, 2H), 1.43 (s, 9H).

^{13}C NMR (126 MHz, CDCl$_3$) δ 162.5 (d, *J* = 237.1 Hz), 156.1, 147.1 (d, *J* = 14.3 Hz), 141.1 (d, *J* = 7.7 Hz), 134.5 (d, *J* = 4.4 Hz), 109.3 (d, *J* = 37.4 Hz), 79.5, 40.1, 31.8, 29.3, 28.5.

^{19}F NMR (471 MHz, CDCl$_3$) δ -72.0 (s).

FTIR (thin film) ν (cm^{-1}) = 3343, 2978, 2937, 1697, 1529, 1486, 1251, 1173, 760.

HRMS: (ESI+) *m/z* calculated for $C_{13}H_{20}FN_2O_2$ requires *m/z* 255.1503 [M+H]$^+$. Found *m/z* 255.1498.

5-(3-Chloro-4-methoxyphenyl)-*N*-(3-(6-fluoropyridin-3-yl)propyl)oxazole-4-carboxamide (OCM-50)

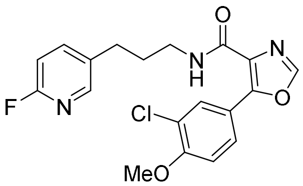

Under ambient conditions, *tert*-butyl (3-(6-fluoropyridin-3-yl)propyl)carbamate (**S-57**) (70 mg, 0.28 mmol, 1 eq.) dissolved in DCM (1 mL) and cooled to 0 °C. HCl in dioxane (450 µL, 4 M, 1.7 mmol, 6 eq.) was added. After 15 minutes, the cooling bath removed, and the mixture stirred for 2 h at room temperature. After this time, the solvent was removed under reduced pressure and the corresponding hydrochloride salt carried forward to the next step with no further purification.

The title compound was prepared as previously described in literature on a 0.2 mmol scale from 5-(3-chloro-4-methoxyphenyl)oxazole-4-carboxylic acid (**S-46**) (1 eq.) and 3-(6-fluoropyridin-3-yl)propan-1-amine hydrochloride (1 eq.).[40]

5-(3-Chloro-4-methoxyphenyl)-*N*-(3-(4-(trifluoromethyl)phenyl)propyl)oxazole-4-carboxamide (39)

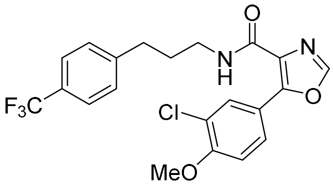

The title compound was prepared following **General Procedure F** on a 0.6 mmol scale from 5-(3-chloro-4-methoxyphenyl)oxazole-4-carboxylic acid (**S-46**) (1 eq.) and 3-(4-(trifluoromethyl)phenyl)propan-1-amine (1 eq.). Purification by column chromatography [20% EtOAc in hexane → 30% EtOAc in hexane] resulted in a white solid (238 mg, 0.54 mmol, 90% yield).

^{1}H NMR (400 MHz, CDCl$_3$) δ 8.39 – 8.31 (m, 2H), 7.77 (s, 1H), 7.52 (d, *J* = 8.0 Hz, 2H), 7.31 (d, *J* = 8.0 Hz, 2H), 7.31 – 7.25 (m, 1H, *overlapping*), 6.99 (d, *J* = 8.6 Hz, 1H), 3.94 (s, 3H), 3.48 (q, *J* = 6.9 Hz, 2H), 2.81 – 2.73 (m, 2H), 1.97 (p, *J* = 7.4 Hz, 2H).

^{13}C NMR (101 MHz, CDCl$_3$) δ 161.2, 156.4, 151.6, 147.7, 145.7, 130.0, 128.8, 128.6, 125.5 (q, *J* = 3.8 Hz), 128.5 (q, *J* = 32.3 Hz), 128.2, 124.4 (q, *J* = 271.9 Hz), 122.6, 120.5, 111.7, 56.3, 38.9, 33.3, 31.1.

^{19}F NMR (376 MHz, CDCl$_3$) δ -62.3 (s).

FTIR (thin film) ν (cm^{-1}) = 3516, 2943, 1666, 1522, 1328, 1123, 1068, 762.

m.p. 69 –72 °C

HRMS: (ESI+) *m/z* calculated for $C_{21}H_{19}ClF_3N_2O_3$ requires *m/z* 439.1031 $[M+H]^+$. Found *m/z* 439.1022.

_N_-(3-(4-Fluorophenyl)propyl)-7-hydroxy-2-oxo-_2H_-chromene-3-carboxamide (28)

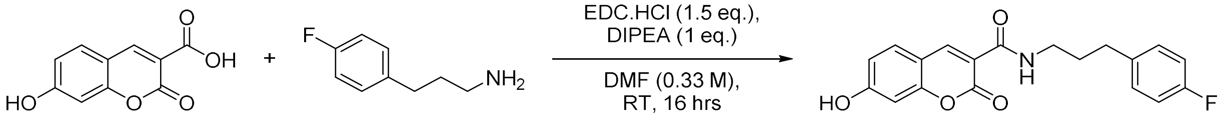

1 eq. 2.5 eq. **28**

In a 2-neck flask under N_2, 7-hydroxy-2-oxo-*2H*-chromene-3-carboxylic acid (103 mg, 0.5 mmol, 1 eq.), *tert*-butyl-3-aminopropylcarbamate (100 mg, 0.52 mmol, 1.05 eq.) and EDC.HCl (144 mg, 0.75 mmol, 1.5 eq.) were dissolved in DMF (1.5 mL, 0.33 M) and DIPEA (50 µL, 0.5 mmol, 1 eq.) was added. The reaction mixture was stirred at room temperature for 16 h. Upon completion, H_2O (10 mL) was added and the mixture extracted with DCM (3 x 25 mL). The combined organics were washed with brine (3 x 25 mL), dried over sodium sulfate, filtered, and the solvent removed under reduced pressure. Purification by column chromatography [100% DCM → 2% MeOH in DCM] and recrystallisation from EtOH resulted in the title compound as a clear crystalline solid (29 mg, 0.09 mmol, 18% yield).

^{1}H NMR (400 MHz, DMSO-*d*$_6$) δ 11.04 (s, 1H), 8.76 (s, 1H), 8.66 (t, *J* = 5.8 Hz, 1H), 7.81 (d, *J* = 8.7 Hz, 1H), 7.25 (ddd, *J* = 8.6, 5.4, 2.6 Hz, 2H), 7.14 – 7.03 (m, 2H), 6.87 (dd, *J* = 8.6, 2.3 Hz, 1H), 6.80 (d, *J* = 2.2 Hz, 1H), 3.31 (q, *J* = 6.8 Hz, 2H), 2.62 (t, *J* = 7.6 Hz, 2H), 1.81 (p, *J* = 7.3 Hz, 2H).

^{13}C NMR (101 MHz, DMSO-*d*$_6$) δ 163.6, 161.7, 161.0, 160.6 (d, *J* = 241.0 Hz), 156.2, 147.9, 137.6 (d, *J* = 3.0 Hz), 131.9, 130.0 (d, *J* = 7.9 Hz), 114.9 (d, *J* = 20.9 Hz), 114.3, 113.8, 111.1, 101.8, 38.5, 31.6, 30.8.

^{19}F NMR (376 MHz, DMSO-*d*$_6$) δ -117.7 (ddd, *J* = 14.8, 9.2, 5.6 Hz).

FTIR (thin film) ν (cm^{-1}) = 3325, 2980, 2886, 1715, 1620, 1548, 1378, 1224, 1145, 953.

m.p. 213 – 215 °C

HRMS: (ESI+) *m/z* calculated for $C_{19}H_{17}FNO_4$ requires *m/z* 342.1136 [M+H]$^+$. Found *m/z* 342.1129.

Scheme S16: Multistep synthesis of 1-(2-(benzhydryloxy)ethyl)-4-(3-(4-fluorophenyl)propyl)piperazine (**26**)

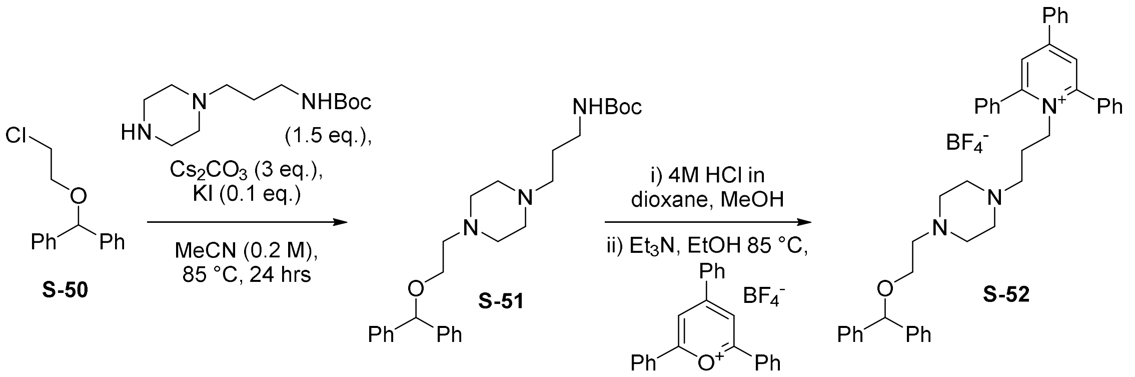

***Tert*-butyl 4-(3-(4-fluorophenyl)propyl)piperazine-1-carboxylate (S-58)**

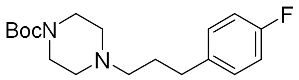

1-Boc-piperazine (224 mg, 1.2 mmol, 3 eq.), Cs_2CO_3 (196 mg, 1.2 mmol, 3 eq.), and KI (7 mg, 40 μmol, 0.1 eq.) were added to a Schlenk flask and cycled under N_2. MeCN (2 mL, 0.2 M) was added followed by 1-(3-bromopropyl)-4-fluorobenzene (62 μL, 0.4 mmol, 1 eq.). The mixture was heated to reflux at 85 °C for 24 h. Purification by column chromatography [5% MeOH in DCM] resulted in the title compound as a yellow oil (108 mg, 0.33 mmol, 84% yield).

^{1}H NMR (500 MHz, CDCl$_3$) δ 7.12 (dd, *J* = 8.4, 5.5 Hz, 2H), 6.95 (t, *J* = 8.7 Hz, 2H), 3.45 – 3.40 (m, 4H), 2.61 (t, *J* = 7.7 Hz, 2H), 2.39 – 2.31 (m, 6H), 1.78 (p, *J* = 7.6 Hz, 2H), 1.45 (s, 9H).

^{13}C NMR (126 MHz, CDCl$_3$) δ 161.4 (d, *J* = 243.3 Hz), 154.9, 137.7 (d, *J* = 3.2 Hz), 129.8 (d, *J* = 7.7 Hz), 115.2 (d, *J* = 21.0 Hz), 79.7, 57.9, 53.1, 43.4, 32.9, 28.7, 28.6.

^{19}F NMR (471 MHz, CDCl$_3$) δ -117.8 (q, *J* = 6.9 Hz).

FTIR (thin film) ν (cm^{-1}) = 2936, 2360, 1695, 1509, 1419, 1245, 1171, 1004, 832.

HRMS: (ESI+) *m/z* calculated for $C_{18}H_{28}FN_2O_2$ requires *m/z* 323.2129 $[M+H]^+$. Found *m/z* 323.2123.

1-(2-(Benzhydryloxy)ethyl)-4-(3-(4-fluorophenyl)propyl)piperazine (26)

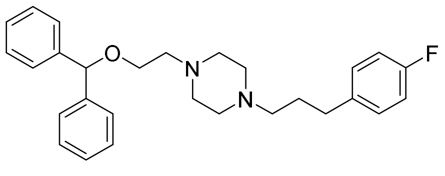

Tert-butyl 4-(3-(4-fluorophenyl)propyl)piperazine-1-carboxylate (**S-58**) (81 mg, 0.25 mmol, 1 eq.) was dissolved in DCM (1 mL) and cooled to 0 °C in a one-necked round bottom flask under ambient atmosphere. HCl in dioxane (375 μL, 4 M, 1.5 mmol, 6 eq.) was added. After 15 minutes, the cooling bath removed, and the mixture stirred for 2 h at room temperature. After this time, the solvent was removed under reduced pressure and the resulting hydrochloride salt carried forward to the next step with no further purification.

Cs_2CO_3 (244 mg, 0.75 mmol, 3 eq.) and KI (4 mg, 25 μmol, 0.1 mmol) were cycled under N_2 in a Schlenk flask. The piperazine.2HCl salt was dissolved in MeCN (625 μL,) and Et_3N (70 μL, 0.5 mmol, 2 eq.) and added to the solids. ((2-Chloroethoxy)methylene)dibenzene (**S-50**) (62 mg, 0.25 mmol, 1 eq.) was dissolved in MeCN (625 μL) and added to the same flask. The mixture was heated to reflux at 85 °C for 24 h. Purification by column chromatography [5% MeOH in DCM → 7% MeOH in DCM] resulted in the title compound as an orange oil (58 mg, 0.13 mmol, 52% yield).

^{1}H NMR (500 MHz, $CDCl_3$) δ 7.36 – 7.28 (m, 8H), 7.27 – 7.21 (m, 2H), 7.12 (dd, *J* = 8.4, 5.6 Hz, 2H), 6.95 (t, *J* = 8.7 Hz, 2H), 5.38 (s, 1H), 3.60 (t, *J* = 6.1 Hz, 2H), 2.69 (t, *J* = 6.1 Hz, 2H), 2.63 – 2.37 (m, 10H), 2.38 – 2.31 (m, 2H), 1.79 (p, *J* = 7.7 Hz, 2H).

^{13}C NMR (126 MHz, $CDCl_3$) δ 161.4 (d, *J* = 243.2 Hz), 142.4, 137.8 (d, *J* = 3.1 Hz), 129.8 (d, *J* = 7.7 Hz), 128.5, 127.5, 127.1, 115.1 (d, *J* = 21.1 Hz), 84.1, 67.1, 58.1, 58.0, 53.8, 53.4, 33.0, 28.8.

^{19}F NMR (470 MHz, $CDCl_3$) δ -117.8 – -117.9 (m).

FTIR (thin film) ν (cm^{-1}) = 2923, 2853, 2808, 2359, 1509, 1454, 1221, 1157, 1099, 1076, 1017, 822, 741, 701.

HRMS: (ESI+) *m/z* calculated for $C_{28}H_{34}FN_2O$ requires *m/z* 433.2650 $[M+H]^+$. Found *m/z* 433.2652.

(2-Fluoroethoxy)benzene (S-59)

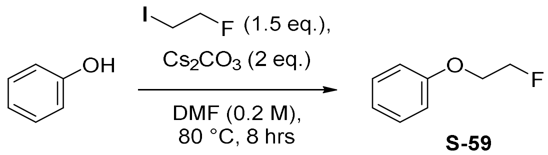

Phenol (38 mg, 0.4 mmol, 1 eq.) and Cs_2CO_3 (261 mg, 0.8 mmol, 2 eq.) were cycled under N_2 in a Schlenk flask. DMF (2 mL, 0.2 M) was added followed by 1-fluoro-2-iodoethane (50 μL, 0.6 mmol, 1.5 eq.). The flask was sealed and heated to 80 °C for 2 h. Upon completion, the reaction mixture was diluted with EtOAc (10 mL) and washed with brine (3 x 10 mL). The organics were dried over Na_2SO_4, filtered and solvent removed under reduced pressure. Purification by column chromatography [10% Et_2O in pentane] resulted in a white crystalline solid (73 mg, 0.3 mmol, 81% yield).

^{1}H NMR (400 MHz, $CDCl_3$) δ 7.34 – 7.27 (m, 2H), 6.99 (tt, *J* = 7.4, 1.1 Hz, 2H), 6.96 – 6.91 (m, 1H), 4.85 – 4.79 (m, 1H), 4.74 – 4.67 (m, 1H), 4.29 – 4.22 (m, 1H), 4.22 – 4.15 (m, 1H).

^{13}C NMR (101 MHz, $CDCl_3$) δ 158.5, 129.7, 121.4, 114.8, 82.1 (d, *J* = 170.6 Hz), 67.2 (d, *J* = 20.6 Hz).

The spectral data match those previously reported.[67]

3 High-Throughput Experimentation (HTE)

3.1 General Information for HTE

All reaction preparation was performed under ambient conditions. Zinc (powder, <150 µm, >99% trace metals basis) was activated as described in **Section 2.1** and stored under ambient conditions for up to 3 months. Anhydrous solvents were purchased from Sigma Aldrich (99.5%, Extra Dry, AcroSeal™).

Ultra-High Performance Liquid Chromatography (UPLC) measurement was performed using an HClassPlus UPLC® system from Waters® (Milford, MA, USA) equipped with a binary solvent delivery pump, an autosampler with flow-through needle injector, a column compartment with 4 column positions, a DAD detector, a SQD2 detector and a sample organizer module for sample introduction into the autosampler. **UPLC Analytical method: flow:** 0.8 m/L; **temperature:** 55 °C, **solvents: A** (0.1 % formic acid in H_2O/ MeCN) and **B** (MeCN). Initial conditions: 0% **B**. 0 min to 2.10 min [Linear gradient 0% to 95% **B**]; 2.10 min to 3.0 min [Linear gradient 95% to 100% **B**]; 3.0 min to 3.50 min [Linear gradient 100% to 95% **B**]. **Column:** XBridge C18, 1.7 µm, 2.1 x 50 mm. Flow from the column was brought through the DAD detector (210-400nm) to the SQD2 Mass Spectrometer (MS) which was measuring in ESI positive and negative mode. Data acquisition was performed with Masslynx v4.2 and processed with Analytical studio (Virscidian).

Para-dox® Standard 96-position Parallel Synthesis Reaction Block (SKU: 96960) sealed with PFA film, rubber mat, and metal top cover was used for HTE-assay. 1 mL vials (8 x 20 mm) with stir bars (1.6 x 4.8 mm) were used. Heating and stirring was accomplished by heat blocks (VP 741DCE, deep chamber, 220 volts, V&P Scientific Inc.) placed on top of a tumble stirring unit (VP 710E5X, Vertical Tumble Stirrer, Double Stack Servo Motor, 5 SLAS Positions, Manual Control, V&P Scientific Inc.) to ensure even heating and stirring of all reactions in the parallel synthesis reaction blocks. Chronect® Quantos weighing robot was used to pre-weigh zinc and **33** into parallel reactor vials. Tecan Freedom EVO-2 liquid handler was used to prepare UPLC-MS samples. Genevac Series 3i HT-6 or HT-12 was used for solvent evaporation.

3.2 General Method for HTE Assay

The reaction, work-up, and analytical method were optimised such that there is a differential in stoichiometry between the fluorinated (hetero)aryl iodide coupling partner and 2,4,6-triphenylpyridinium tetrafluoroborate coupling partner (**33**), while in the limits of UPLC-MS analysis. The relative ratio of **33** and every other component of the reaction mixture (*i.e.* $NiBr_2$.glyme, PyBCAM, Zinc, NMP) is analogous to the developed radiochemical reaction, but at 1/5 scale to conserve material of **33**. This results in a 4-fold excess of **33** *c.f.* fluorinated (hetero)aryl iodide coupling partner.

Process Overview:

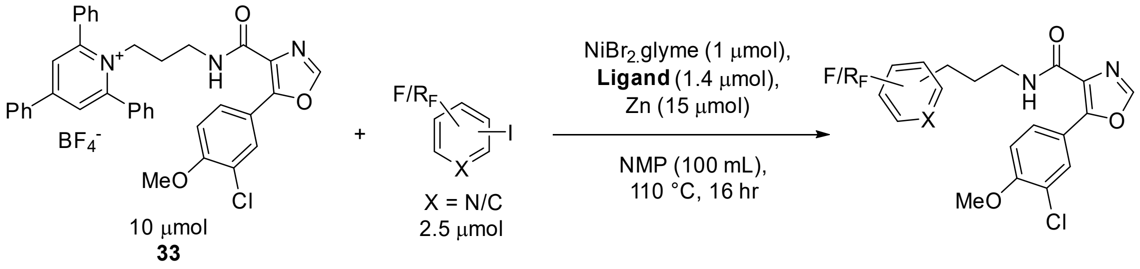

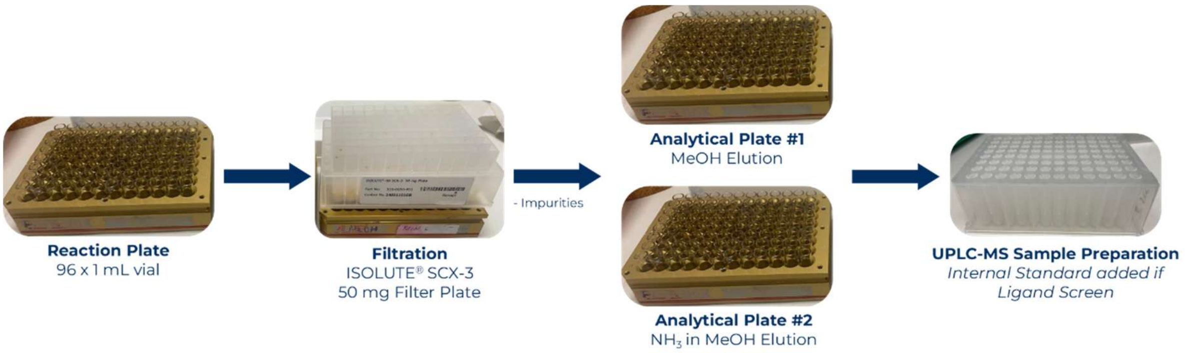

Stock solution preparation:

12 stock solutions of $NiBr_2$.glyme (3.1 mg, 10 μmol) and ligand (14 μmol) were weighed into a 1 mL vial equipped with stirrer bar. Anhydrous NMP (500 μL) was subsequently added and the mixture sealed with a PTFE vial cap with a Teflon septum. If a heterogenous mixture occurred, the mixture was sonicated until fully solubilized (up to 30s). [50 μL of stock solution per reaction = $NiBr_2$.glyme (1 μmol), PyBCAM (1.4 μmol)].

Pre-weighed aryl iodides (10 μmol) were diluted in anhydrous NMP (200 μL). [50 μL/ reaction = fluoro(hetero)aryl iodide (2.5 μmol)].

Reaction Preparation:

1-(3-(5-(3-chloro-4-methoxyphenyl)oxazole-4-carboxamido)propyl)-2,4,6-triphenylpyridin-1-ium tetrafluoroborate (**33**, 7 mg, 10 μmol) and zinc (1 mg, 15 μmol) were weighed into reaction vials using a Chronect® Quantos weighing robot. Fluoro(Hetero)aryl halide stock

solution (50 µL) was dispensed into the reaction vials and the $NiBr_2$.glyme/ PyBCAM stock solution (50 µL) subsequently added, total reaction volume = 100 µL.

The reactions were sealed and heated to 110 degrees for 16 hours with stirring.

Reaction work-up:

n.b. ISOLUTE® SCX-3 Solid-Phase extraction media was found to be optimal for **33**, however, ISOLUTE® SCX and ISOLUTE® SCX-2 may be more suited to semi-purification of alternative 2,4,6-triphenylpyridinium substrates.

ISOLUTE®-96 SCX-3 50 mg Fixed Well Plate (Biotage, 533-0050-P01) was preconditioned with MeOH (1 mL). The reaction mixture was diluted with MeOH (800 µL) and loaded onto the filter plate dropwise, collecting the eluent. The filter plate was washed with MeOH (1 mL) and collected dropwise into the same plate as the eluent from loading the crude reaction mixture [Analytical Plate #1]. The collection plate was exchanged with a new one and the filter plate washed with NH_3 (3.5 M in MeOH, 1 mL) [Analytical Plate #2].

MeOH/ NH_3 was removed completely by Genevac. Analytical Plate #1 had MeCN (100 µL) added, and Analytical Plate #2 had MeCN (200 µL) added such that the total volume across both plates is 300 µL.

Internal standard stock solution: 4,4'-di-*tert*-butyl-1,1'-biphenyl (2.5 µmol/ 200 µL) in 1:4 DMSO: MeCN

Following this, further sample preparation was performed by Tecan Freedom EVO-2 liquid handler.

The MeCN diluted reaction mixture (300 µL) was diluted by internal standard stock solution (200 µL) and mixed. 10 µL of this mixture was sampled and diluted in MeCN (250 µL) resulting in the UPLC-MS sample.

Analytical Plate #1 was measured by UPLC-MS first. If no product *m/z* was observed, then the corresponding well of Analytical Plate #2 was sampled. Cation exchange cartridges trap basic residues, and therefore, should the fluoro(hetero)aryl iodide feature a basic residue, the resulting product of the cross-coupling will be trapped by the ISOLUTE®-96 SCX-3 filter media and only elute with NH_3.

3.3 HTE Ligand Screens

3.3.1 Ligand Selection

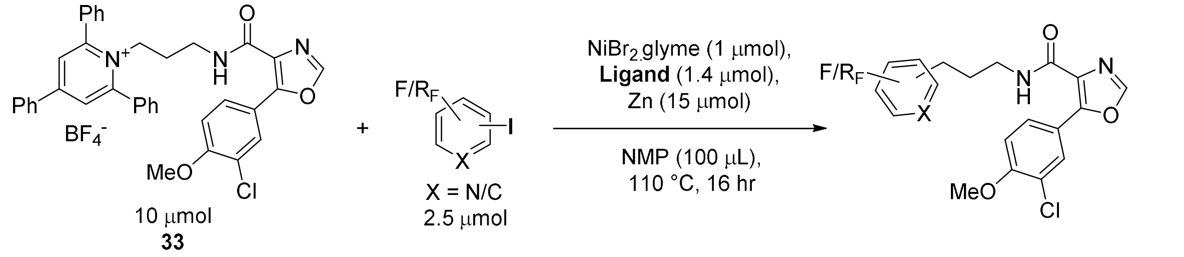

Ligands that achieved > 50% RCC in preliminary radiochemical screens (see SI 1.6.6) were included in the HTE assay. Commercially available ligands with prior reports in cross-electrophile couplings were prioritised.

Figure S12: Structure of ligands investigated in HTE screen organised by class.

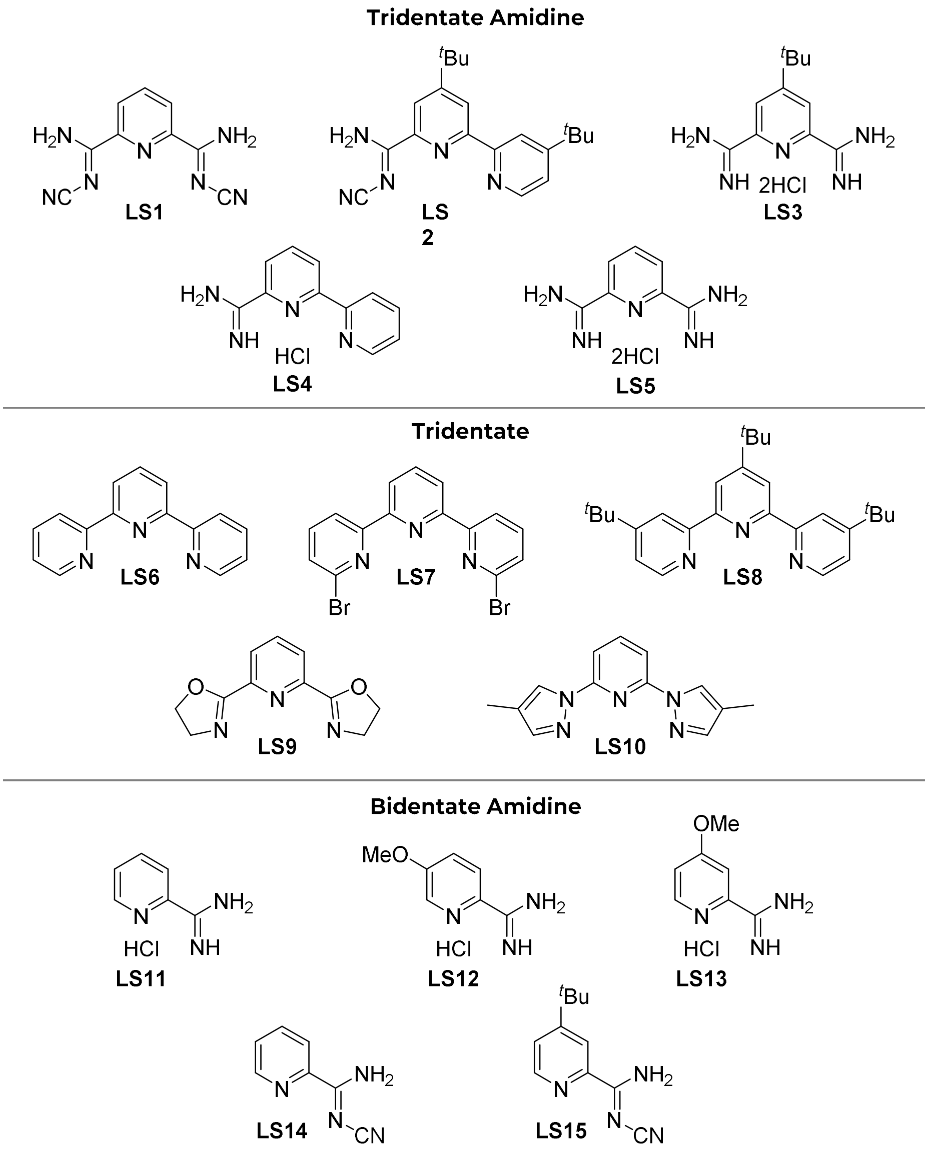

Figure S12 (continued): Structure of ligands investigated in HTE screen organised by class.

Phenathroline

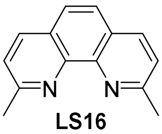

LS16

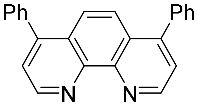

LS17

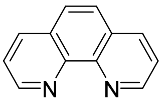

LS18

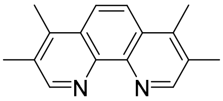

LS19

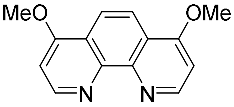

LS20

Bipyridine (bpy)

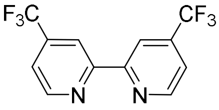

LS21

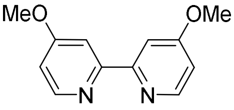

LS22

Bidentate

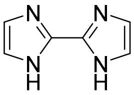

LS23

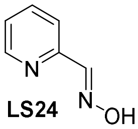

LS24

3.3.2 Ligand Screen Fluorinated Fluoro(hetero)aryl Iodides

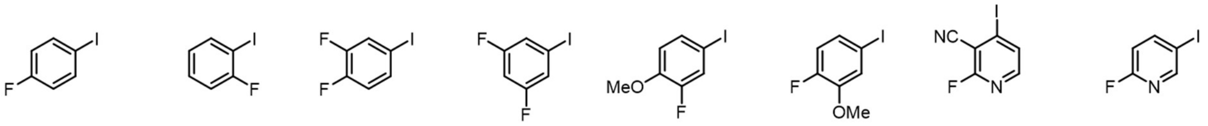

LS Ar-1 **LS Ar-2** **LS Ar-3** **LS Ar-4** **LS Ar-5** **LS Ar-6** **LS Ar-7** **LS Ar-8**

3.3.3 HTE Ligand Screen Results

Values in the heat map are % values as calculated using the equation below where LCA = area under the curve of the peak in the UPLC-MS chromatogram for the specified species. Product is the resulting cross-coupled product from **33** (**see Sections 3.2 and 3.3**) and the specified fluoro(hetero)aryl iodides in **Section 3.3.2** Reactions were set up and analysed as described in **Section 3.2** with an equimolar amount of 4,4'-di-tert-butyl-1,1'-biphenyl added as UPLC-MS internal standard.

$$\% = \frac{LCA_{product}}{LCA_{internal\ standard}} \times 100$$

Due to the varied UV absorbance of different products, numerical results should only be compared relatively across a row – *i.e.* same product formed - and do **not** correspond to the overall yield of the reaction.

Figure S15: Heat Map of High-Throughput Ligand Screening Results.

	Tridentate Amidine					Tridentate					Bidentate Amidine					Phenanthroline					bpy		bidentate	
	**LS1**	**LS2**	**LS3**	**LS4**	**LS5**	**LS6**	**LS7**	**LS8**	**LS9**	**LS10**	**LS11**	**LS12**	**LS13**	**LS14**	**LS15**	**LS16**	**LS17**	**LS18**	**LS19**	**LS20**	**LS21**	**LS22**	**LS23**	**LS24**
**LS Ar-1**	41	66	58	57	41	30	12	31	12	23	13	15	18	40	28	0	5	n/a	13	16	n/a	8	0	6
**LS Ar-2**	36	52	70	51	58	48	0	52	0	0	0	0	0	33	0	0	0	0	34	0	0	0	0	0
**LS Ar-3**	45	68	61	60	81	37	0	64	0	28	0	0	0	40	0	0	41	0	0	0	0	0	0	0
**LS Ar-4**	50	56	41	56	79	38	0	52	0	22	27	0	0	52	0	0	0	0	23	0	0	0	0	23
**LS Ar-5**	55	49	93	49	60	30	49	85	55	34	0	60	49	39	0	85	0	0	0	8	0	10	0	39
**LS Ar-6**	0	59	82	38	64	45	0	59	0	0	0	39	47	0	25	42	0	0	15	25	0	15	0	47
**LS Ar-7**	21	23	56	30	42	0	0	0	0	0	0	0	0	0	0	0	0	0	0	0	0	0	19	0
**LS Ar-8**	112	93	74	73	80	81	30	67	41	48	98	80	44	63	22	0	0	0	80	0	0	0	0	0

0 50 100

Percentile

3.3.4 Comparison of Radiochemical/ HTE Ligand Screens

3.3.4A Validation using [^{18}F]5/ LS Ar-1.

Radiochemical reactions were set up as described in **Section 1.5** and HTE conditions are as described in **Section 3.2** using **LS Ar-1**.

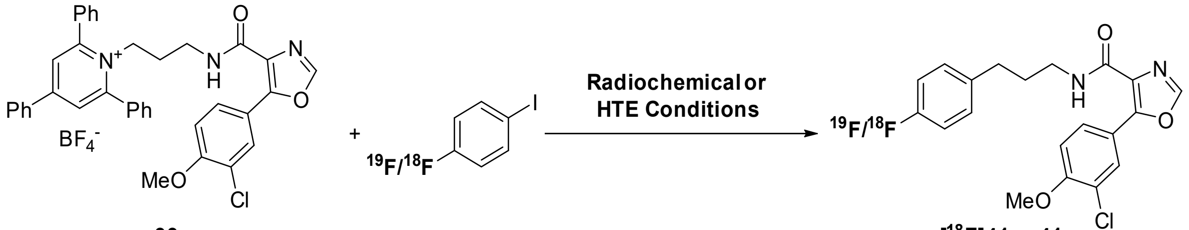

33 **[^{18}F]41** or **41**

	LS1	LS2	LS3	LS4	LS5	LS11	LS16	LS22
HTE	41	66	58	57	41	13	0	8
%RCY	**70**	41	**79***	**77**	**68***	33	2	18

**Positive Control** (LS1–LS5)
**Negative Control** (LS11, LS16, LS22)

n = 1 unless * then number is the average value of n = 2 runs.

The HTE value is as described in **Section 3.3.3.** %RCC = radiochemical conversion as determined by RadioHPLC.

Amidine ligands **LS1** through **LS5** were chosen as positive controls. 4 of these ligands resulted in high %RCCs of **[^{18}F]41** – except **LS2** which displayed only moderate %RCC of **[^{18}F]41**.

Bidentate **LS11** and **LS22** were chosen as negative controls and were demonstrated to result in relatively low to moderate amounts of product detected by HTE. Under analogous radiochemical conditions, **LS11** and **LS22** resulted in low to moderate %RCC of **[^{18}F]41**, 33% and 18% respectively.

Phenanthroline based **LS16** was chosen for validation as it demonstrated no conversion to the desired product by HTE and under analogous radiochemical conditions resulted in only 2% RCC of the desired product.

Overall, the trends of the HTE study showed strong alignment with the trends observed in analogous radiochemical reactions using [^{18}F]**5**. However, this demonstrates the best ligand

by HTE may not be optimal in the radiochemical reaction (e.g. **LS2**) and thus a small selection of the top performing ligands should be taken forward for radiochemical screens.

3.3.4B Validation using [^{18}F]37/ LS Ar-2.

Radiochemical reactions were set up as described in **Section 1.5** using **[^{18}F]37** as the aryl iodide coupling partner and HTE conditions are as described in **Section 3.2** using **LS Ar-2**.

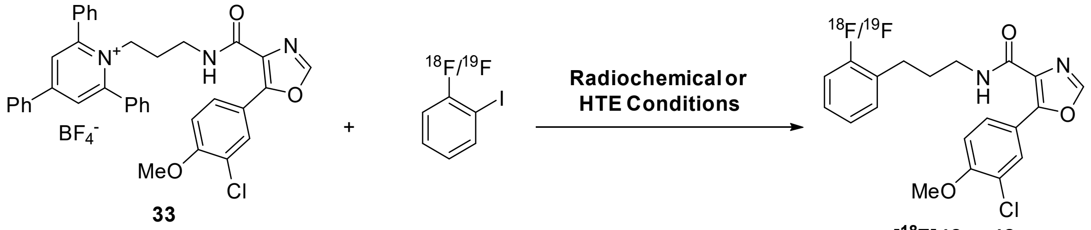

	LS2	LS3	LS4	LS5	LS8	LS11	LS22
HTE	52	70	51	58	52	0	0
%RCY	48	43	**79**	**86**	**71**	8	0

**Positive Control**
**Negative Control**

The HTE value is as described in **Section 3.3.3**. %RCC = radiochemical conversion as determined by RadioHPLC.

Amidine ligands **LS2** through **LS5** were chosen as positive controls. **LS8** is a tridentate pyridine ligand which interestingly was not optimal in the HTE screen using **[^{18}F]5**. All 5 of these ligands resulted in moderate to high %RCCs.

Bidentate **LS11** and **LS22** were chosen as negative controls and resulted in no product detected by HTE. Under analogous radiochemical conditions, **LS11** and **LS22** resulted in 8% and 0% RCC of **[^{18}F]42** respectively.

Overall, the trends of the HTE study showed strong alignment with the trends observed in analogous radiochemical reactions using **[^{18}F]37**. However, this demonstrates the best ligand by HTE may not be optimal in the radiochemical reaction (e.g. **LS3**) and thus a small selection of the top performing ligands should be taken forward for radiochemical screens.

3.4 High-Throughput Synthesis and Purification for Characterisation of Products Observed in HTE

3.4.1 General Information for High-Throughput Synthesis and Purification

Specifications from **Sections 2.1** and **3.1** apply to high throughput purification and characterisation with the differences noted below:

Para-dox® 2-dram, 24-Position Parallel Synthesis Reaction Block (SKU: 24017) sealed with PFA film, rubber mat, and metal top cover was used for HTE-assay. 2-dram vials (8 mL, 17 x 60 mm) with PTFE stir bars (10 x 3 mm) were used.

Chronect® Quantos weighing robot was used to pre-weigh zinc into parallel reactor vials.

Reversed Phase HPLC (RP-HPLC) was performed using a WatersLabomatic mass-directed preparative system equipped with a 2545 Waters Binary solvent pump, a 515 Waters make-up pump, 515 Waters ACD pump, a DAD detector, a column compartment, and a Labomatic Injector/collector platform (Labomatic Instruments Ag, Switzerland). The preparative runtime was 15 minutes.

Reversed Phase HPLC (RP-HPLC) Conditions:

Column: XBridge C18, 10 µm, 30 x 100 mm. Column flow was split 1/5000 to PDA and SQD2 by Waters flow splitter.

Binary pump: 43 m/L, ACD pump: 2 m/L; **make-up pump:** 1.0 m/L; **Temperature:** RT, **Solvents: A** (Formic acid, 25 mM 95% H2O + 5% MeCN) and **B** (MeCN). Column equilibration method was run with initial conditions for 2.0 min. Focused gradients were run to 100% B in 11.5 min, kept for 3.5 min.

The desired product was collected with a Labomatic fraction collector. Fractions were sampled by UPLC-MS (**Section 3.1**) acquisition was performed with Masslynx v4.2 and processed with Analytical studio (Virscidian). Pooled fractions were dried by Genevac and characterised by HRMS, ^{1}H NMR, and ^{19}F NMR **(Section 2.1).**

3.4.2 General Method for High Throughput Synthesis and Purification

Product compounds (**S-60** to **S-64**) from the HTE ligand screen were synthesised and purified as follows:

The stoichiometry of **33**, $NiBr_2$.glyme, PyBCAM, and zinc are in the same ratio as the HTE assay with scaled up molarity of the reaction and increased stoichiometry of fluorinated (hetero)aryl iodide to enable isolation of the product.

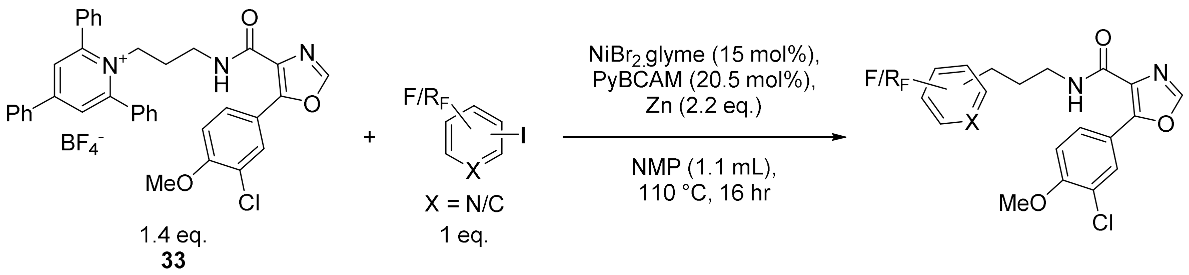

Stock solution preparation:

$NiBr_2$.glyme (102 mg, 0.3305 mmol) and pyridine-2,6-bis(carboximidamide) dihydrochloride (PyBCAM, 109 mg, 0.4618 mmol) were weighed into a 20 mL vial equipped with stirrer bar. Anhydrous NMP (13.5 mL) was subsequently added and the mixture sealed with a PTFE vial cap with a Teflon septum. The mixture was degassed by bubbling N_2 through the mixture from a balloon and a vent needle in the septum. The mixture was sonicated while degassing until fully solubilized (30 seconds to 1 minute). The mixture was stored under N_2 *via* N_2-filled balloon until dispensed. [450 μL/ reaction = $NiBr_2$.glyme (11 μmol), PyBCAM (15.4 μmol)].

1-(3-(5-(3-chloro-4-methoxyphenyl)oxazole-4-carboxamido)propyl)-2,4,6-triphenylpyridin-1-ium tetrafluoroborate (**33**, 2.020 g) was dissolved in anhydrous NMP (5.600 mL) and degassed and stored under N_2 as described above. [200 μL/ reaction = **33** (105 μmol)]

Reaction Preparation:

Zinc (11 mg, 2.2 eq., 165 μmol) was weighed into 2-dram reaction vials. Pre-weighed aryl iodides (75 μmol, 1 eq.) were diluted in anhydrous NMP (450 μL) and added to the reaction vial containing Zn. The stock solution of **33** (200 μL, 105 μmol, 1.4 eq.) and the $NiBr_2$.glyme/ PyBCAM stock solution [450 μL = $NiBr_2$.glyme (11 μmol), PyBCAM (15.4 μmol)] were added in that order.

The reactions were sealed and heated to 110 degrees for 16 hours with stirring.

Reaction work-up:

n.b. ISOLUTE® SCX-3 Solid-Phase extraction media was found to be optimal for ***33****, however, ISOLUTE® SCX and ISOLUTE® SCX-2 may be more suited to semi-purification of alternative 2,4,6-triphenylpyridinium substrates.*

DMSO (100 µL) was added to the reaction mixtures and NMP removed by Genevac. MeOH (1.4 mL) was added, and the mixtures sonicated to ensure the resulting gel was dissolved.

Biotage® Extrahera™ Classic was used to perform solid phase extraction using ISOLUTE® SCX-3 2 g/6 mL (Tabless) (Biotage 533-0200-CG) cartridges. The cartridges were preconditioned with MeOH. The crude reaction mixture was loaded manually onto the cartridges eluting dropwise. Automated elution with MeOH (2 x 3 mL) [Fraction #1] followed by with NH_3 (3.5 M in MeOH, 2 x 3 mL) [Fraction #2]. Solvents were removed by Genevac and redissolved in DMSO (100 µL) followed by MeCN (2 mL) and filtered prior to UPLC-MS sample preparation and high-throughput purification.

Analytical samples for Fraction #1 were measured by UPLC-MS first. If no product *m/z* was observed, then the corresponding well for analytical samples for Fraction #2 were then sampled. Cation exchange cartridges trap basic residues, and therefore, should the fluoro(hetero)aryl iodide feature a basic residue, the resulting product of the cross-coupling will be trapped by the ISOLUTE® SCX-3 filter media and only elute with NH_3.

High-throughput purification was performed as described in **Section 3.4.1**.

3.4.3 Characterisation Data for HT Purified Compounds

Yields are not reported as they are not representative of the overall yield of the process due to the high throughput purification process. ^{13}C NMRs, IRs, and melting points are not reported due to the low amounts of isolated product (< 10 mg).

5-(3-chloro-4-methoxyphenyl)-*N*-(3-(3,4-difluorophenyl)propyl)oxazole-4-carboxamide (S-60)

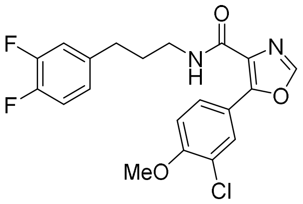

^{1}H NMR (400 MHz, CDCl$_3$) δ 8.36 (d, *J* = 8.7 Hz, 1H), 8.33 (d, *J* = 2.2 Hz, 1H), 7.79 (s, 1H), 7.30 (bs, 1H), 7.10 – 6.96 (m, 3H), 6.90 (ddt, *J* = 8.2, 3.8, 1.7 Hz, 1H), 3.95 (s, 3H), 3.46 (q, *J* = 6.8 Hz, 2H), 2.67 (t, *J* = 7.7 Hz, 2H), 1.93 (p, *J* = 7.4 Hz, 2H).

^{19}F NMR (377 MHz, CDCl$_3$) δ -138.1 – -138.4 (m), -142.0 – -142.2 (m).

HRMS: (ESI+) *m/z* calculated for $C_{20}H_{18}ClF_2N_2O_3$ requires *m/z* 407.0969 [M+H]$^+$. Found *m/z* 407.0984.

5-(3-chloro-4-methoxyphenyl)-*N*-(3-(3,5-difluorophenyl)propyl)oxazole-4-carboxamide (S-61)

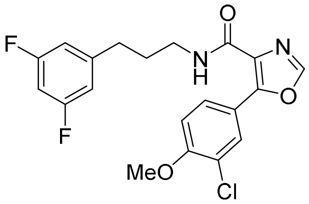

^{1}H NMR (400 MHz, CDCl$_3$) δ 8.37 (dd, *J* = 8.7, 2.2 Hz, 1H), 8.32 (d, *J* = 2.2 Hz, 1H), 7.80 (s, 1H), 7.31 (s, 2H), 7.00 (d, *J* = 8.8 Hz, 1H), 6.72 (dt, *J* = 6.9, 2.1 Hz, 2H), 6.67 – 6.59 (m, 1H), 3.95 (s, 4H), 3.47 (q, *J* = 6.8 Hz, 3H), 2.74 – 2.66 (m, 3H), 1.95 (p, *J* = 7.7 Hz, 2H).

^{19}F NMR (377 MHz, CDCl$_3$) δ -110.5 (t, *J* = 8.7 Hz).

HRMS: (ESI+) *m/z* calculated for $C_{20}H_{18}ClF_2N_2O_3$ requires *m/z* 407.0969 [M+H]$^+$. Found *m/z* 407.0975.

5-(3-chloro-4-methoxyphenyl)-*N*-(3-(3-fluoro-4-methoxyphenyl)propyl)oxazole-4-carboxamide (S-62)

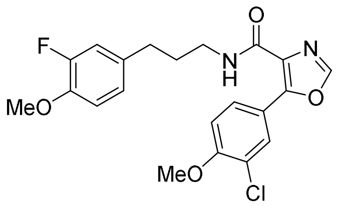

^{1}H NMR (400 MHz, CDCl$_3$) δ 8.38 (dd, *J* = 8.7, 2.2 Hz, 1H), 8.32 (d, *J* = 2.2 Hz, 1H), 7.79 (s, 1H), 7.28 (bs, 1H), 7.00 (d, *J* = 8.8 Hz, 1H), 6.95 – 6.84 (m, 3H), 3.95 (s, 3H), 3.86 (s, 3H), 3.46 (q, *J* = 6.8 Hz, 2H), 2.65 (t, *J* = 7.7 Hz, 2H), 1.92 (p, *J* = 7.5 Hz, 2H).

^{19}F NMR (377 MHz, CDCl$_3$) δ -135.5 (dd, *J* = 12.1, 7.6 Hz).

HRMS: (ESI+) *m/z* calculated for $C_{21}H_{21}ClFN_2O_4$ requires *m/z* 419.1168 [M+H]$^+$. Found *m/z* 419.1177.

5-(3-chloro-4-methoxyphenyl)-*N*-(3-(4-fluoro-3-methoxyphenyl)propyl)oxazole-4-carboxamide (S-63)

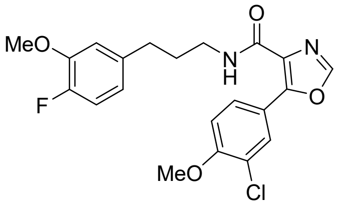

^{1}H NMR (400 MHz, $CDCl_3$) δ 8.39 – 8.32 (m, 2H), 7.79 (s, 1H), 7.29 (bs, 1H), 7.03 – 6.92 (m, 2H), 6.81 (dd, *J* = 8.2, 2.1 Hz, 1H), 6.71 (ddd, *J* = 8.2, 4.3, 2.1 Hz, 1H), 3.95 (s, 3H), 3.87 (s, 3H), 3.47 (q, *J* = 6.8 Hz, 2H), 2.68 (t, *J* = 7.7 Hz, 2H), 1.94 (p, *J* = 7.4 Hz, 2H).

^{19}F NMR (377 MHz, $CDCl_3$) δ -139.5 – -139.6 (m).

HRMS: (ESI+) *m/z* calculated for $C_{21}H_{21}ClFN_2O_4$ requires *m/z* 419.1168 [M+H]$^+$. Found *m/z* 419.1162.

5-(3-chloro-4-methoxyphenyl)-*N*-(3-(3-cyano-2-fluoropyridin-4-yl)propyl)oxazole-4-carboxamide (S-64)

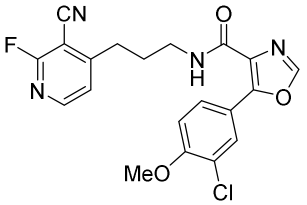

^{1}H NMR (500 MHz, $CDCl_3$) δ 8.35 – 8.32 (m, 2H), 8.30 (d, *J* = 5.2 Hz, 1H), 7.81 (s, 1H), 7.41 (s, 1H), 7.27 (bs, 1H, *overlapping with $CHCl_3$*), 7.01 (d, *J* = 9.3 Hz, 1H), 3.96 (s, 3H), 3.54 (q, *J* = 6.7 Hz, 2H), 2.99 (t, *J* = 7.6 Hz, 2H), 2.07 (p, *J* = 7.1 Hz, 2H).

^{19}F NMR (471 MHz, $CDCl_3$) δ -60.8 (s).

HRMS: (ESI+) *m/z* calculated for $C_{20}H_{17}ClFN_4O_3$ requires *m/z* 415.0968 [M+H]$^+$. Found *m/z* 415.0974.

4 References

[1] S. M. Ametamey, M. Honer, P. A. Schubiger, "Molecular Imaging with PET." *Chem. Rev.* **2008**, *108*, 1501–1516.

[2] J. K. Willmann, N. van Bruggen, L. M. Dinkelborg, S. S. Gambhir, "Molecular Imaging in Drug Development." *Nat. Rev. Drug Discov.* **2008**, *7,* 591–607.

[3] J. Rong, A. Haider, T. E. Jeppesen, L. Josephson, S. H. Liang, "Radiochemistry for Positron Emission Tomography." *Nat. Commun.* **2023**, *14*, 3257.

[4] O. Jacobson, D. O. Kiesewetter, X. Chen, "Fluorine-18 Radiochemistry, Labeling Strategies and Synthetic Routes." *Bioconjug. Chem.* **2015**, *26*, 1–18.

[5] M. Tredwell, V. Gouverneur, "^{18}F Labeling of Arenes." *Angew. Chem. Int. Ed.* **2012**, *51*, 11426–11437.

[6] K. J. Makaravage, A. F. Brooks, A. V. Mossine, M. S. Sanford, P. J. H. Scott, "Copper-Mediated Radiofluorination of Arylstannanes with [^{18}F]KF." *Org. Lett.* **2016**, *18*, 5440–5443.

[7] T. Gendron, K. Sander, K. Cybulska, L. Benhamou, P. K. B. Sin, A. Khan, M. Wood, M. J. Porter, E. Årstad, "Ring-Closing Synthesis of Dibenzothiophene Sulfonium Salts and Their Use as Leaving Groups for Aromatic ^{18}F-Fluorination." *J. Am. Chem. Soc.* **2018**, *140*, 11125–11132.

[8] N. J. Taylor, E. Emer, S. Preshlock, M. Schedler, M. Tredwell, S. Verhoog, J. Mercier, C. Genicot, V. Gouverneur, "Derisking the Cu-Mediated ^{18}F-Fluorination of Heterocyclic Positron Emission Tomography Radioligands." *J. Am. Chem. Soc.* **2017**, *139*, 8267–8276.

[9] R. Schirrmacher, B. Wängler, J. Bailey, V. Bernard-Gauthier, E. Schirrmacher, C. Wängler, "Small Prosthetic Groups in ^{18}F-Radiochemistry: Useful Auxiliaries for the Design of ^{18}F-PET Tracers." *Semin. Nucl. Med.* **2017**, *47*, 474–492.

[10] N. J. Castellino, A. P. Montgomery, J. J. Danon, M. Kassiou, "Late-Stage Functionalization for Improving Drug-like Molecular Properties." *Chem. Rev.* **2023**, *123*, 8127–8153.

[11] J. Boström, D.G. Brown, R. J. Young, G. M. Keserü, "Expanding the Medicinal Chemistry Synthetic Toolbox." *Nat. Rev. Drug Discov.* **2018**, *17*, 709–727.

[12] A. W. Dombrowski, A. L. Aguirre, A. Shrestha, K. A. Sarris, Y. Wang, "The Chosen Few: Parallel Library Reaction Methodologies for Drug Discovery." *J. Org. Chem.* **2022**, *87*, 1880–1897.

[13] A. W. Dombrowski, N. J. Gesmundo, A. L. Aguirre, K. A. Sarris, J. M. Young, A. R. Bogdan, M. C. Martin, S. Gedeon, Y. Wang, "Expanding the Medicinal Chemist Toolbox: Comparing Seven C(Sp^2)–C(Sp^3) Cross-Coupling Methods by Library Synthesis." *ACS Med. Chem. Lett.* **2020**, *11*, 597–604.

[14] W. Liu, J. Mulhearn, B. Hao, S. Cañellas, S. Last, J. E. Gómez, A. Jones, A. De Vera, K. Kumar, R. Rodríguez, L. Van Eynde, I. I. Strambeanu, S. E. Wolkenberg, "Enabling Deoxygenative C(Sp^2)-C(Sp^3) Cross-Coupling for Parallel Medicinal Chemistry." *ACS Med. Chem. Lett.* **2023**, *14*, 853–859.

[15] H. Doi, M. Goto, M Suzuki, "Pd^0-Mediated Rapid C-[^{18}F]Fluoromethylation by the Cross-Coupling Reaction of a [^{18}F]Fluoromethyl Halide with an Arylboronic Acid Ester: Novel Method for the Synthesis of a ^{18}F-Labeled Molecular Probe for Positron Emission Tomography." *Bull. Chem. Soc. Jpn.* **2012**, *85*, 1233–1238.

[16] J. B. I. Sap, C. F. Meyer, J. Ford, N. J. W. Straathof, A. B. Dürr, M. J. Lelos, S. J. Paisey, T. A. Mollner, S. M. Hell, A. A. Trabanco, C. Genicot, C. W. am Ende, R. S. Paton, M. Tredwell, V. Gouverneur, "[^{18}F]Difluorocarbene for Positron Emission Tomography." *Nature* **2022**, *606*, 102–108.

[17] M. Huiban, M. Tredwell, S. Mizuta, Z. Wan, X. Zhang, T. L. Collier, V. Gouverneur, J. A. Passchier, "Broadly Applicable [^{18}F]Trifluoromethylation of Aryl and Heteroaryl Iodides for PET Imaging." *Nat. Chem.* **2013**, *5*, 941–944.

[18] J. Liao, C. H. Basch, M. E.Hoerrner, M. R. Talley, B. P. Boscoe, J. W. Tucker, M.R. Garnsey, M. P. Watson, "Deaminative Reductive Cross-Electrophile Couplings of Alkylpyridinium Salts and Aryl Bromides." *Org. Lett.* **2019**, *21*, 2941–2946.

[19] R. Martin-Montero, V. R. Yatham, H. Yin, J. Davies, R. Martin, "Ni-Catalyzed Reductive Deaminative Arylation at Sp^3 Carbon Centers." *Org. Lett.* **2019**, *21*, 2947–2951.

[20] H. Yue, C. Zhu, L. Shen, Q. Geng, K. J. Hock, T. Yuan, L. Cavallo, M. Rueping. *Chem. Sci.,* **2019**, 10, 4430–4435

[21] C. H. Basch, J. Liao, J. Xu, J. J. Piane, M. P. Watson, "Harnessing Alkyl Amines as Electrophiles for Nickel-Catalyzed Cross Couplings *via* C–N Bond Activation." *J. Am. Chem. Soc.* **2017**, *139*, 5313–5316.

[22] J. Yi, S. O. Badir, L. M. Kammer, M. Ribagorda, G. A. Molander, "Deaminative Reductive Arylation Enabled by Nickel/Photoredox Dual Catalysis." *Org. Lett.* **2019**, *21*, 3346–3351.

[23] T. Yang, Y. Wei, M. J. Koh, "Photoinduced Nickel-Catalyzed Deaminative Cross-Electrophile Coupling for $C(Sp^2)$–$C(Sp^3)$ and $C(Sp^3)$–$C(Sp^3)$ Bond Formation." *ACS Catal.* **2021**, *11*, 6519–6525.

[24] A. R. Katritzky, R. T. Langthorne, R. C. Patel, G. Lhommet, "Transformations of Pyridiniums Derived from Amino-Alcohols and from Diamines: Novel Ring Closures." *Tetrahedron* **1981**, *37*, 2383–2390.

[25] J. Wu, L. He, A. Noble, V. K. Aggarwal, "Photoinduced Deaminative Borylation of Alkylamines." *J. Am. Chem. Soc.* **2018**, *140*, 10700–10704.

[26] A. R. Katritzky, O. A. Rubio, "Convenient Synthesis of *N*-Vinylpyridinium Perchlorate and a Study of *N*-Vinylpyridinium Cations as Michael Reaction Acceptors." *J. Org. Chem.* **1983**, *48*, 4017–4021.

[27] L. E. Ehehalt, O. M. Beleh, I. C. Priest, J. M. Mouat, A. K. Olszewski, B. N. Ahern, A. R. Cruz, B. K. Chi, A. J. Castro, K. Kang, J. Wang, D. J. Weix, "Cross-Electrophile Coupling: Principles, Methods, and Applications in Synthesis." *Chem. Rev.* **2024**, *124*, 13397–13569.

[28] M. Tredwell, S. M. Preshlock, N. J. Taylor, S. Gruber, M. Huiban, J. Passchier, J. Mercier, C. Génicot, V. Gouverneur, "A General Copper-Mediated Nucleophilic ^{18}F Fluorination of Arenes." *Angew. Chem. Int. Ed.* **2014**, *53*, 7751–7755.

[29] J. C. Twitty, Y. Hong, B. Garcia, S. Tsang, J. Liao, D. M. Schultz, J. Hanisak, S. L. Zultanski, A. Dion, D. Kalyani, M. P. Watson, "Diversifying Amino Acids and Peptides *via* Deaminative Reductive Cross-Couplings Leveraging High-Throughput Experimentation." *J. Am. Chem. Soc.* **2023**, *145*, 5684–5695.

[30] D. J. Charboneau, H. Huang, E. L. Barth, C. C. Germe, N. Hazari, B. Q. Mercado, M. R. Uehling, S. L. Zultanski, "Tunable and Practical Homogeneous Organic Reductants for Cross-Electrophile Coupling." *J. Am. Chem. Soc.* **2021**, *143*, 21024–21036.

[31] S. Ni, C.-X. Li, Y. Mao, J. Han, Y. Wang, H. Yan, Y. Pan, "Ni-catalyzed deaminative cross-electrophile coupling of Katritzky salts with halides via C—N bond activation" *Sci. Adv.* **2019**, 5, eaaw9516.

[32] Z.-M. Su, R. Deng, S. S. Stahl, "Zinc and Manganese Redox Potentials in Organic Solvents and Their Influence on Nickel-Catalysed Cross-Electrophile Coupling." *Nat. Chem.* **2024**, *16*, 2036–2043.

[33] A. N. Fohn, Y. Gao, S. Sproules, G. S. Nichol, C. M. Brennan, A. J. Robinson, G. C. Lloyd-Jones, "Kinetics and Mechanism of PPh_3/Ni-Catalyzed, Zn-Mediated, Aryl Chloride Homocoupling: Antagonistic Effects of $ZnCl_2$/Cl^-." *J. Am. Chem. Soc.* **2024**, *146*, 29913–29927.

[34] J. W. Shaw, L. Barbance, D. H. Grayson, I. Rozas, "Using *N*-Substituted-2-Amino-4,6-Dimethoxypyrimidines in the Synthesis of Aliphatic Guanidines." *Tetrahedron Lett.* **2015**, *56*, 4990–4992.

[35] P. van der Zee, W. Hespe, "Interaction between Substituted 1-[2-(Diphenylmethoxy)Ethyl] Piperazines and Dopamine Receptors." *Neuropharmacology* **1985**, *24*, 1171–1174.

[36] S. H. Liang, J. M. Chen, M. D. Normandin, J. S. Chang, G. C. Chang, C. K. Taylor, P. Trapa, M. S. Plummer, K. S. Para, E. L. Conn, L. Lopresti-Morrow, L. F. Lanyon, J. M. Cook, K. E. G. Richter, C. E. Nolan, J. B. Schachter, F. Janat, Y. Che, V. Shanmugasundaram, B. A. Lefker, B. E. Enerson, E. Livni, L. Wang, N. J. Guehl, D. Patnaik, F. F. Wagner, R. Perlis, E. B. Holson, S. J. Haggarty, G. El Fakhri, R. G. Kurumbail, N. Vasdev, "Discovery of a Highly Selective Glycogen Synthase Kinase-3 Inhibitor (PF-04802367) That Modulates Tau Phosphorylation in the Brain: Translation for PET Neuroimaging." *Angew. Chem. Int. Ed.* **2016**, *55*, 9601–9605.

[37] C. Hooper, R. Killick, S. Lovestone, "The GSK3 Hypothesis of Alzheimer's Disease." *J. Neurochem.* **2008**, *104*, 1433–1439.

[38] C. Ballatore, V. M.-Y. Lee, J. Q.Trojanowski, "Tau-Mediated Neurodegeneration in Alzheimer's Disease and Related Disorders." *Nat. Rev. Neurosci.* **2007**, *8*, 663–672.

[39] P. Lei, S. Ayton, A. I. Bush, P. A. Adlard, "GSK-3 in NeurodegenerativeDiseases." *Int. J. Alzheimer's Dis.* **2011**, *2011*, 189246.

[40] V. Bernard-Gauthier, A. V. Mossine, A. Knight, D. Patnaik, W.-N. Zhao, C. Cheng, H. S. Krishnan, L. L. Xuan, P. S. Chindavong, S. A. Reis, J. M. Chen, X. Shao, J. Stauff, J. Arteaga, P. Sherman, N. Salem, D. Bonsall, B. Amaral, C. Varlow, L. Wells, L. Martarello, S. Patel, S. H. Liang, R. G. Kurumbail, S. J. Haggarty, P. J. H. Scott, N. Vasdev, "Structural Basis for Achieving GSK-3β Inhibition with High Potency, Selectivity, and Brain Exposure for Positron Emission Tomography Imaging and Drug Discovery." *J. Med. Chem.* **2019**, *62*, 9600–9617.

[41] C. Varlow, A. V. Mossine, V. Bernard-Gauthier, P. J. H. Scott, N. Vasdev, "Radiofluorination of Oxazole-Carboxamides for Preclinical PET Neuroimaging of GSK-3." *J. Fluorine Chem.* **2021**, *245*, 109760.

[42] K. Smart, M. Q. Zheng, D. Holden, Z. Felchner, L. Zhang, Y. Han, J. Ropchan, R. E. Carson, N. Vasdev, Y. Huang, "In Vivo Imaging and Kinetic Modeling of Novel Glycogen Synthase Kinase-3 Radiotracers [^{11}C]OCM-44 and [^{18}F]OCM-50 in Non-Human Primates." *Pharmaceuticals* **2023**, *16*, 194.

[43] H. Ahmed, R. Wallimann, A. Haider, V. Hosseini, S. Gruber, M. Robledo, T. A. N. Nguyen, A. M. Herde, I. Iten, C. Keller, V. Vogel, R. Schibli, B. Wünsch, L. Mu, S. M. Ametamey, "Preclinical Development of ^{18}F-OF-NB1 for Imaging GluN2B-Containing N-Methyl-d-Aspartate Receptors and Its Utility as a Biomarker for Amyotrophic Lateral Sclerosis." *J. Nuc. Med.* **2021**, *62*, 259–265.

[44] P. Xu, D. Zhao, F. Berger, A. Hamad, J. Rickmeier, R. Petzold, M. Kondratiuk, K. Bohdan, T. Ritter, "Site-Selective Late-Stage Aromatic [^{18}F]Fluorination *via* Aryl Sulfonium Salts." *Angew. Chem. Int. Ed.* **2020**, *59*, 1956–1960.

[45] L. E. Ehehalt, O. M. Beleh, I. C. Priest, J. M. Mouat, A. K. Olszewski, B. N. Ahern, A. R. Cruz, B. K. Chi, A. J. Castro, K. Kang, J. Wang, D. J. Weix, "Cross-Electrophile Coupling: Principles, Methods, and Applications in Synthesis." *Chem. Rev.* **2024**, *124*, 13397–13569.

[46] R.-Y. Yang, X. Gao, K. Gong, J. Wang, X. Zeng, M. Wang, J. Han, B. Xu, "Synthesis of $ArCF_2X$ and [^{18}F]Ar-CF_3 via Cleavage of the Trifluoromethylsulfonyl Group." *Org. Lett.* **2022**, *24*, 164–168.

[47] J. D. Way, F. Wuest, "Automated Radiosynthesis of No-Carrier-Added 4-[^{18}F]Fluoroiodobenzene: A Versatile Building Block in ^{18}F Radiochemistry." *J. Label. Compd. Radiopharm.* **2014**, *57*, 104–109.

[48] K. Sander, T. Gendron, E. Yiannaki, K. Cybulska, T. L. Kalber, M. F. Lythgoe, E. Årstad, "Sulfonium Salts as Leaving Groups for Aromatic Labelling of Drug-like Small Molecules with Fluorine-18." *Sci. Rep.* **2015**, *5*, 9941.

[49] J. L. Douthwaite, R. Zhao, E. Shim, B. Mahjour, P. M. Zimmerman, T. Cernak, "Formal Cross-Coupling of Amines and Carboxylic Acids to Form Sp^3–Sp^2 Carbon–Carbon Bonds." *J. Am. Chem. Soc.* **2023**, *145*, 10930−10937.

[50] Z. Cai, R. Gu, W. Si, Y. Xiang, J. Sun, Y. Jiao, X. Zhang, "Photoinduced Allylic Defluorinative Alkylation of Trifluoromethyl Alkenes with Katritzky Salts under Catalyst- and Metal-Free Conditions." *Green Chem.* **2022**, *24*, 6830–6835.

[51] J. W. Shaw, L. Barbance, D. H. Grayson, I. Rozas, "Using N-Substituted-2-Amino-4,6-Dimethoxypyrimidines in the Synthesis of Aliphatic Guanidines." *Tetrahedron Lett.* **2015**, *56*, 4990–4992.

[52] H. Crassier, U. Eckert, H. Bottcher, A. Bathe, S. Emmert, "Process For The Preparation Of (3-Cyano-1H-Indol-7-yl) (4-(4-Fluorophenethyl) Piperazin-1-yl)-Methanone And Salts Thereof." US 20040063723 A1, April 1, **2004**.

[53] A. Buzas, A. Champagnac, A. Dehnel, G. Lavielle, M. Pommier, "Synthesis and Psychoanaleptic Properties of New Compounds Structurally Related to Diphenhydramine." *J. Med. Chem.* **1980**, *23*, 149–153.

[54] T. Ishiyama, J. Takagi, Y. Nobuta, N. Miyaura, "Iridium-catalyzed C-H borylation of arenes and heteroarenes: 1-chloro-3-iodo-5-(4,4,5,5-tetramethyl-1,3,2-dioxaborolan-

2-yl)benzene and 2-(4,4,5,5,-tetramethyl-1,3,2-dioxaborolan-2-yl)indole." *Org. Synth.* **2005**, *82*, 126.

[55] S. Humpert, M. A. Omrane, E. A. Urusova, L. Gremer, D. Willbold, H. Endepols, R. N. Krasikova, B. Neumaier, B. D. Zlatopolskiy, "Rapid ^{18}F-Labeling via Pd-Catalyzed S-Arylation in Aqueous Medium." *Chem. Commun.* **2021**, *57*, 3547–3550.

[56] N. J. Taylor, E. Emer, S. Preshlock, M. Schedler, M. Tredwell, S. Verhoog, J. Mercier, C. Genicot, V. Gouverneur, "Derisking the Cu-Mediated ^{18}F-Fluorination of Heterocyclic Positron Emission Tomography Radioligands." *J. Am. Chem. Soc.* **2017**, *139*, 8267–8276.

[57] P. Zhang, C. Le, D. W. C. MacMillan, "Silyl Radical Activation of Alkyl Halides in Metallaphotoredox Catalysis: A Unique Pathway for Cross-Electrophile Coupling." *J. Am. Chem. Soc.* **2016**, *138*, 8084–8087.

[58] Z. Chen, W. Mori, H. Fu, M. A. Schafroth, A. Hatori, T. Shao, G. Zhang, R. S. Van, Y. Zhang, K. Hu, M. Fujinaga, L. Wang, V. Belov, D. Ogasawara, P. Giffenig, X. Deng, J. Rong, Q. Yu, X. Zhang, M. I. Papisov, Y. Shao, T. L. Collier, J.-A. Ma, B. F. Cravatt, L. Josephson, M.-R. Zhang, S. H. Liang, "Design, Synthesis, and Evaluation of ^{18}F-Labeled Monoacylglycerol Lipase Inhibitors as Novel Positron Emission Tomography Probes." *J. Med. Chem.* **2019**, *62*, 8866–8872.

[59] D. A. Everson, R. Shrestha, D. J. Weix, "Nickel-Catalyzed Reductive Cross-Coupling of Aryl Halides with Alkyl Halides." *J. Am. Chem. Soc.* **2010**, *132*, 920–921.

[60] N. Sabat, W. Zhou, V. Gandon, X. Guinchard, G. Vincent, "Unbiased C3-Electrophilic Indoles: Triflic Acid Mediated C3-Regioselective Hydroarylation of N−H Indoles." *Angew. Chem. Int. Ed.* **2022**, *61*, e202204400.

[61] R. A. Lippa, D. J. Battersby, J. A. Murphy, T. N. Barrett, "Synthesis of Arylethylamines via C(Sp^3)–C(Sp^3) Palladium-Catalyzed Cross-Coupling." *J. Org. Chem.* **2021**, *86*, 3583–3604.

[62] S. Holovach, K. P. Melnykov, I. Poroshyn, R. T. Iminov, D. Dudenko, I. Kondratov, M. Levin, O. O. Grygorenko, "C-C Coupling through Nitrogen Deletion: Application to Library Synthesis." *Chem. Eur. J.* **2023**, *29*, e202203470.

[63] Z. Dong, D. W. C. MacMillan, "Metallaphotoredox-Enabled Deoxygenative Arylation of Alcohols." *Nature* **2021**, *598*, 451–456.

[64] B. Zhou, H. Sato, L. Ilies, E. Nakamura, "Iron-Catalyzed Remote Arylation of Aliphatic C–H Bond via 1,5-Hydrogen Shift." *ACS Catal.* **2018**, *8*, 8–11.

[65] C.-S. Yan, Y. Peng, X.-B. Xu, Y.-W. Wang, "Nickel-Mediated Inter- and Intramolecular Reductive Cross-Coupling of Unactivated Alkyl Bromides and Aryl Iodides at Room Temperature." *Chem. Eur. J.* **2012**, *18*, 6039–6048.

[66] T. Koyanagi, A. Herath, A. Chong, M. Ratnikov, A. Valiere, J. Chang, V. Molteni, J. Loren, "One-Pot Electrochemical Nickel-Catalyzed Decarboxylative Sp^2–Sp^3 Cross-Coupling." *Org. Lett.* **2019**, *21*, 816–820.

[67] V. Hugenberg, B. Riemann, S. Hermann, O. Schober, M. Schäfers, K. Szardenings, A. Lebedev, U. Gangadharmath, H. Kolb, J. Walsh, W. Zhang, K. Kopka, S. Wagner, "Inverse 1,2,3-Triazole-1-yl-Ethyl Substituted Hydroxamates as Highly Potent Matrix Metalloproteinase Inhibitors: (Radio)Synthesis, in Vitro and First in Vivo Evaluation." *J. Med. Chem.* **2013**, *56*, 6858–6870.

5 NMR Spectra of Novel Compounds

2-(4-Fluorophenethyl)thiophene (6) ^{1}H NMR (400 MHz, $CDCl_3$)

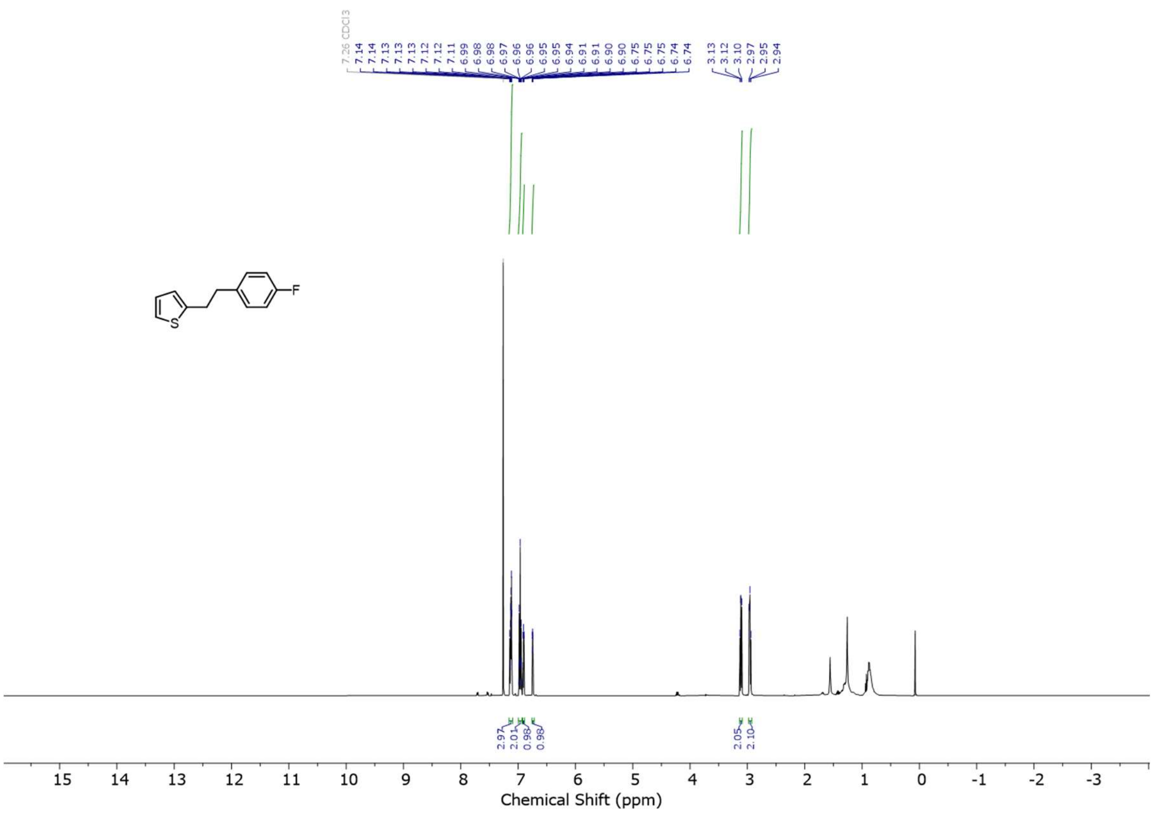

2-(4-Fluorophenethyl)thiophene (6) ^{13}C NMR (126 MHz, $CDCl_3$)

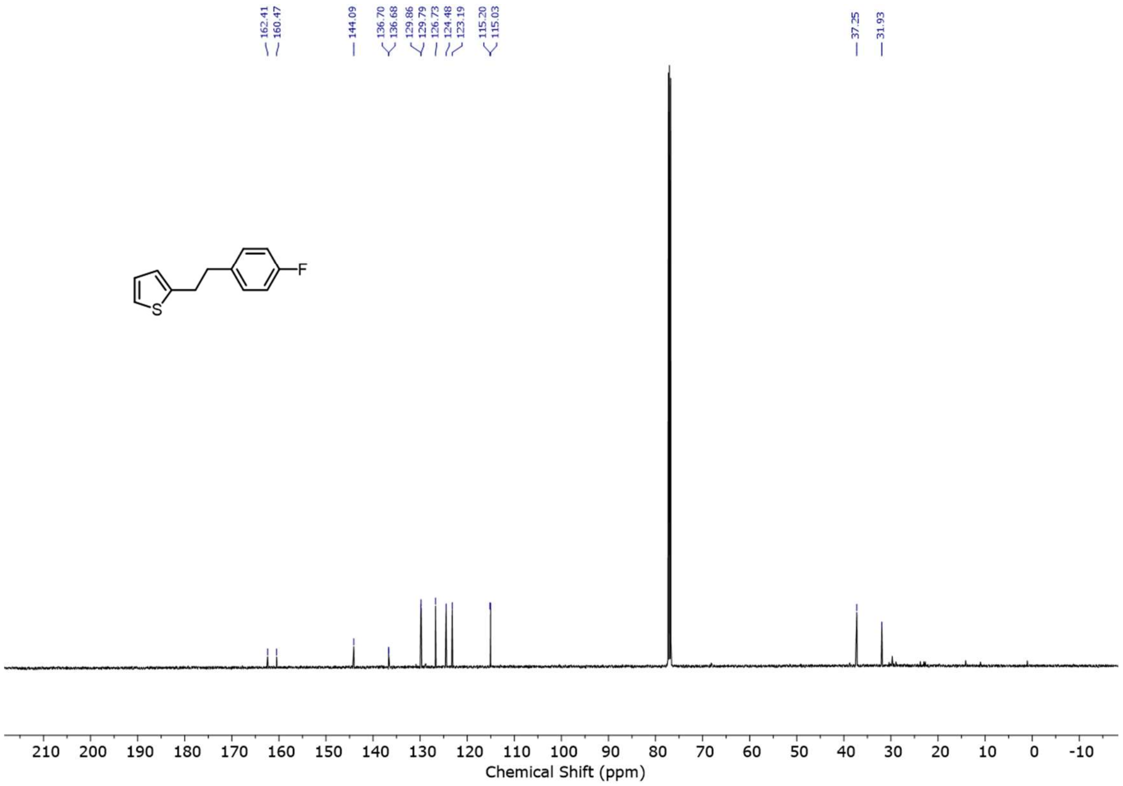

2-(4-Fluorophenethyl)thiophene (6) ^{19}F NMR (377 MHz, $CDCl_3$)

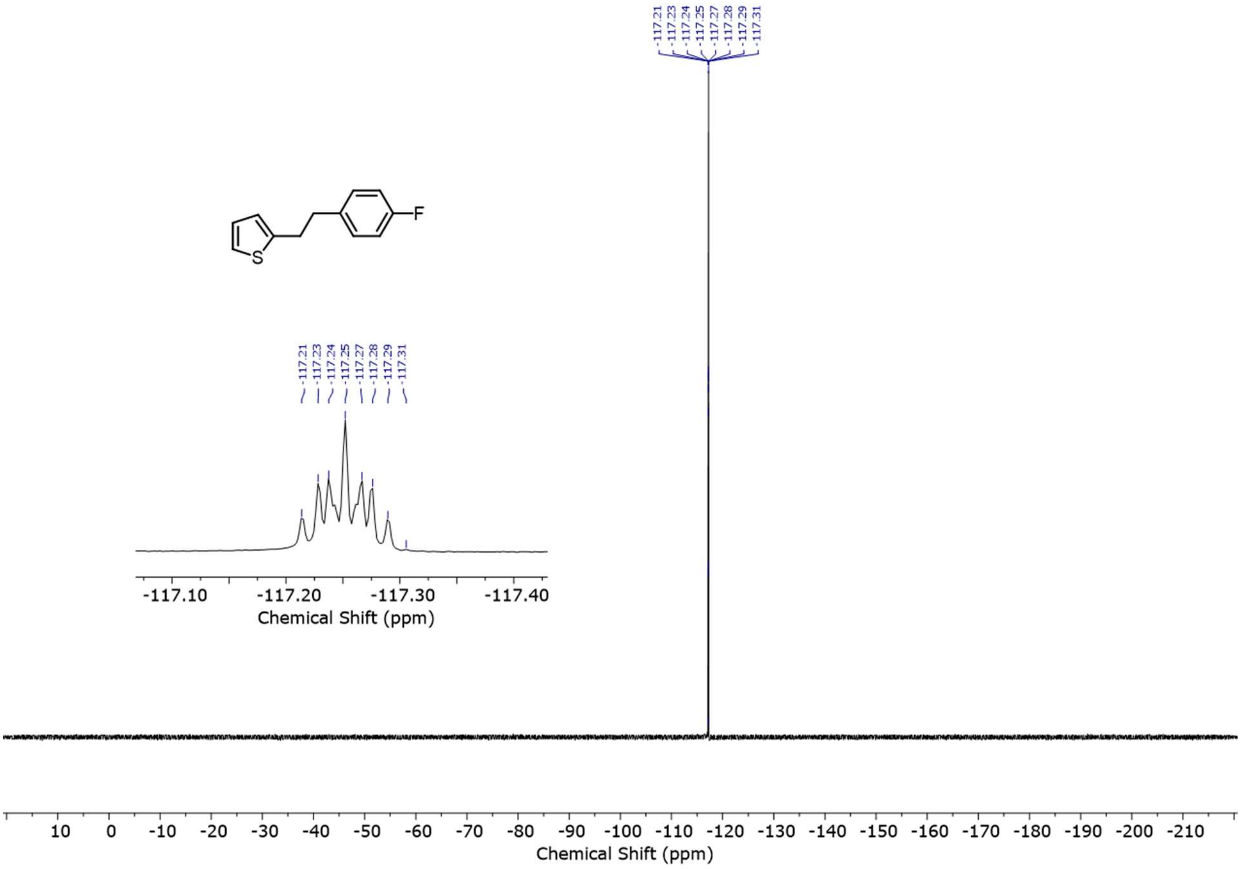

4-Ethyl-1-(4-fluorophenethyl)-1*H*-1,2,3-triazole (8) ^{1}H NMR (400 MHz, $CDCl_3$)

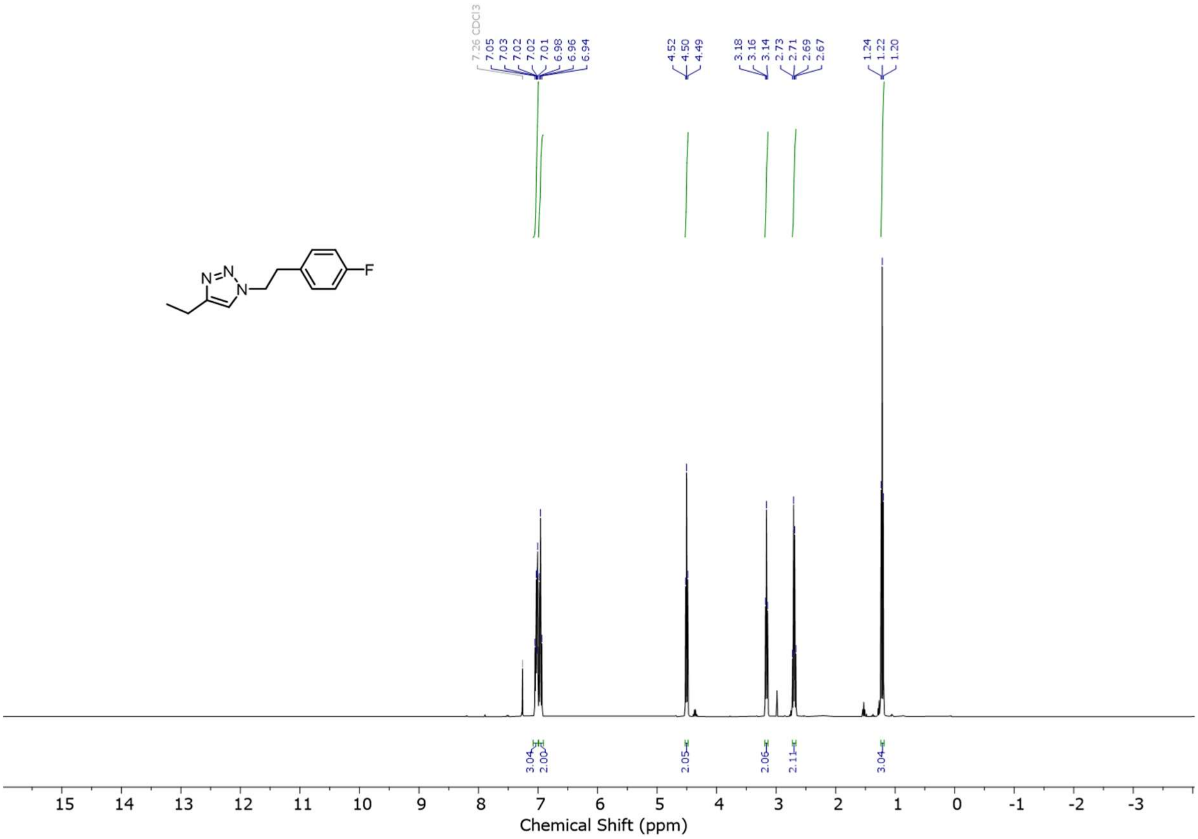

4-Ethyl-1-(4-fluorophenethyl)-1*H*-1,2,3-triazole (8) ^{19}F NMR (377 MHz, $CDCl_3$)

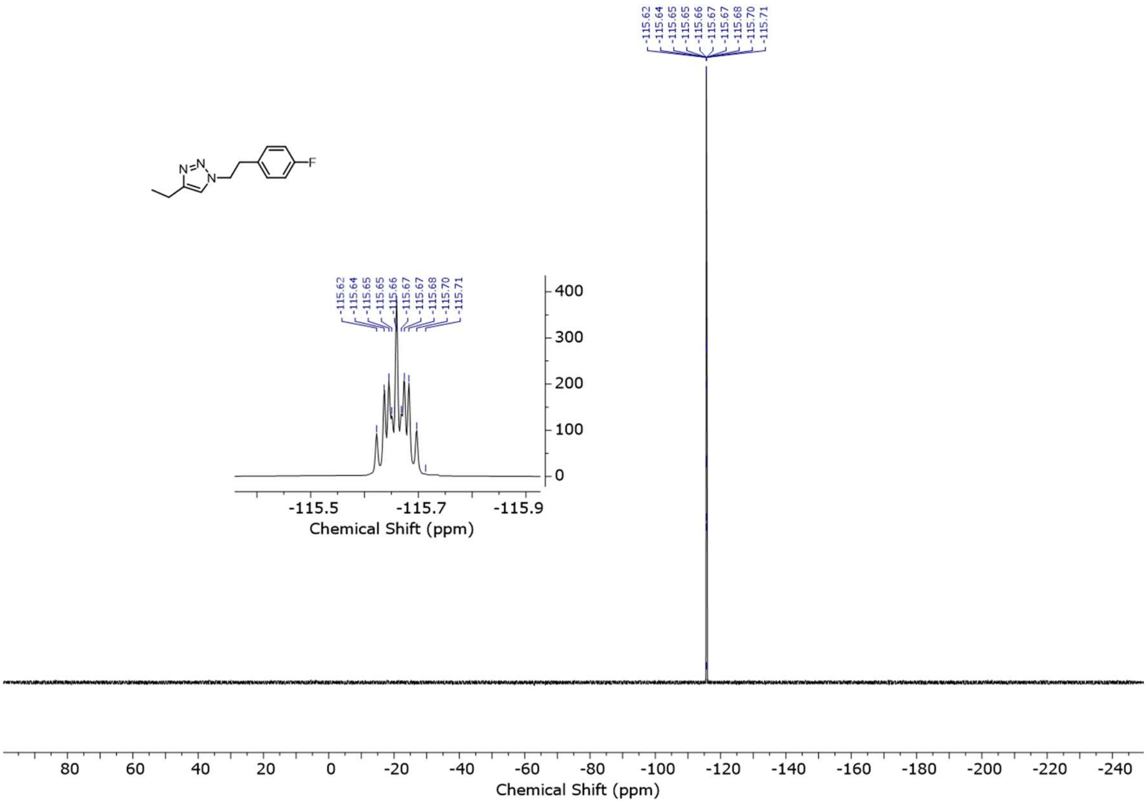

4-Ethyl-1-(4-fluorophenethyl)-1*H*-1,2,3-triazole (8) ^{13}C NMR (101 MHz, $CDCl_3$)

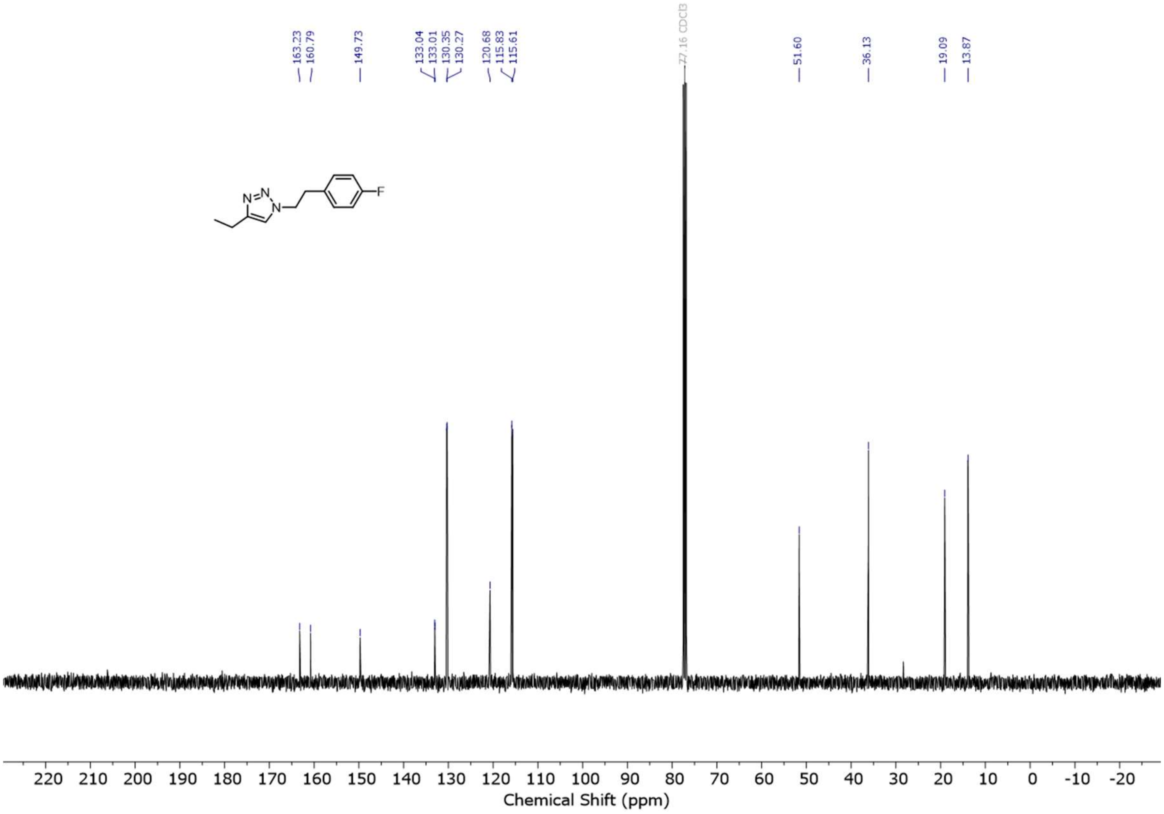

***tert*-Butyl 3-(4-fluorobenzyl)azetidine-1-carboxylate (10) ^{1}H NMR (400 MHz, $CDCl_3$)**

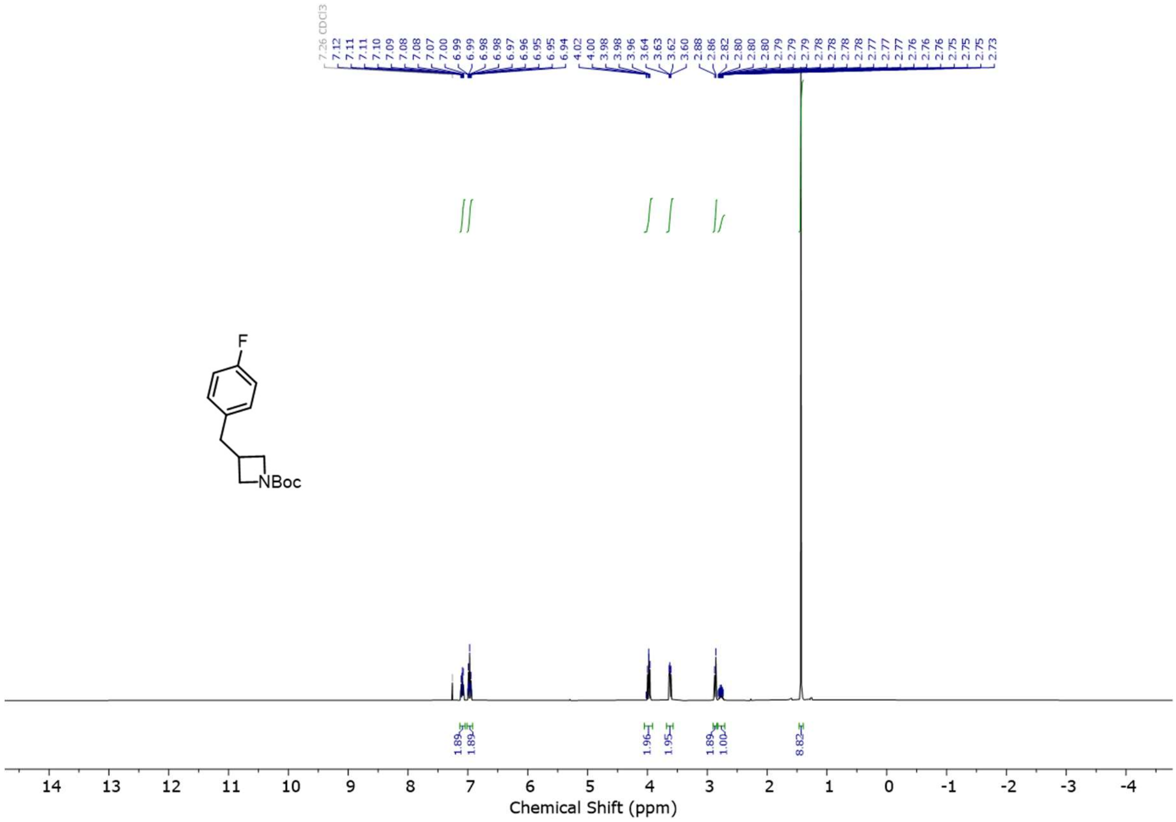

***tert*-Butyl 3-(4-fluorobenzyl)azetidine-1-carboxylate (10) ^{13}C NMR (101 MHz, $CDCl_3$)**

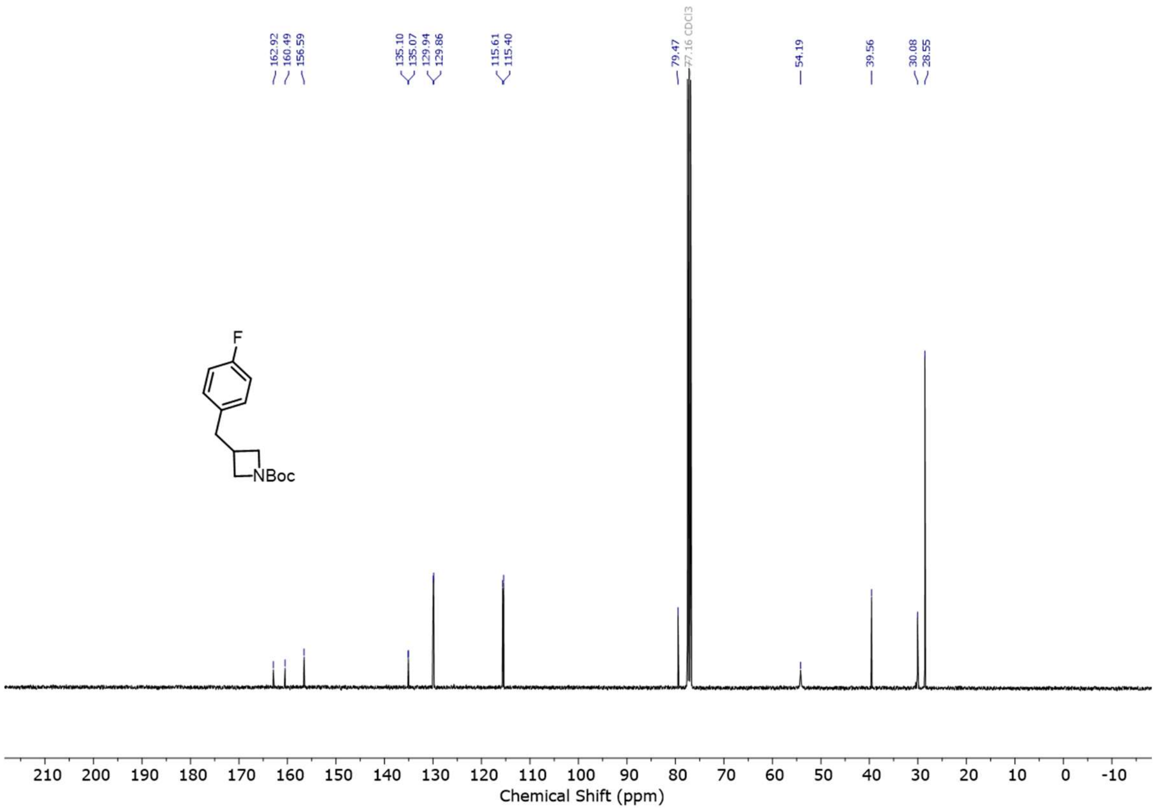

***tert*-Butyl 3-(4-fluorobenzyl)azetidine-1-carboxylate (10) ^{19}F NMR (376 MHz, $CDCl_3$)**

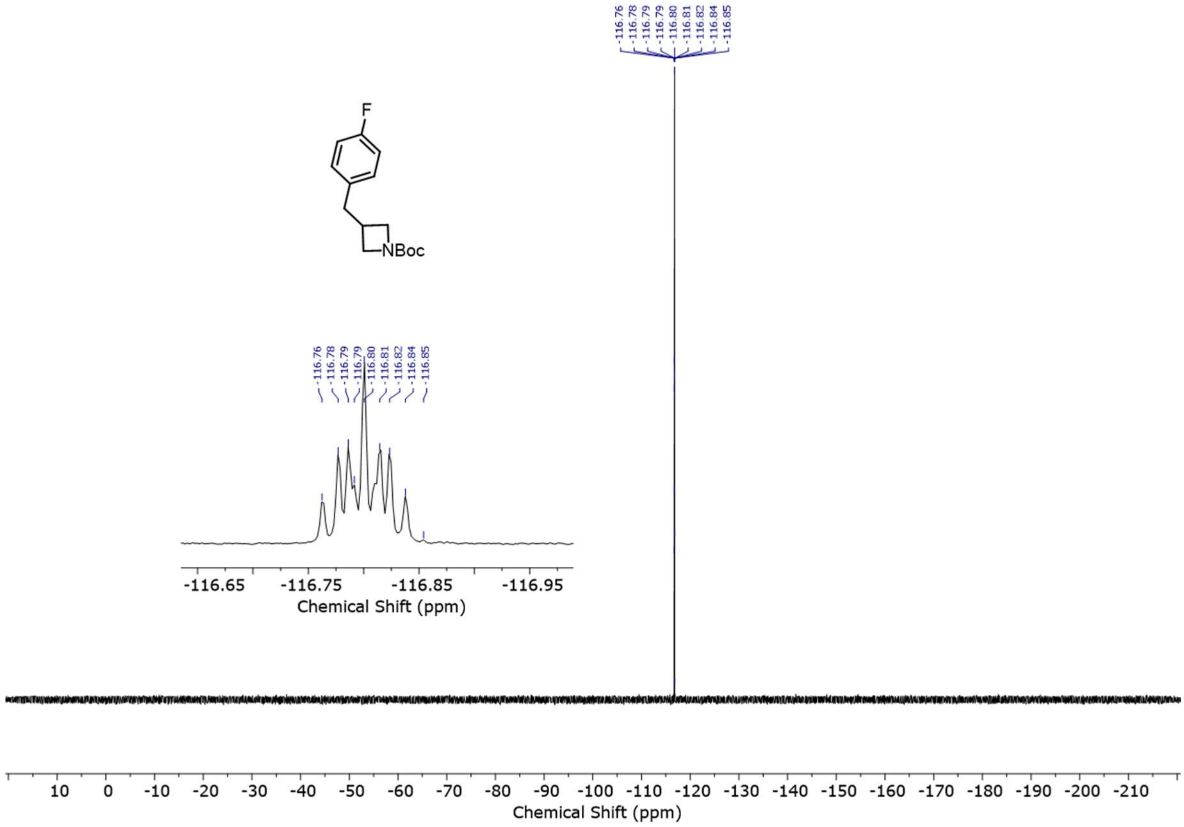

5-(4-Fluorophenethyl)benzo[*d*][1,3]dioxole (15) ^{1}H NMR (400 MHz, $CDCl_3$)

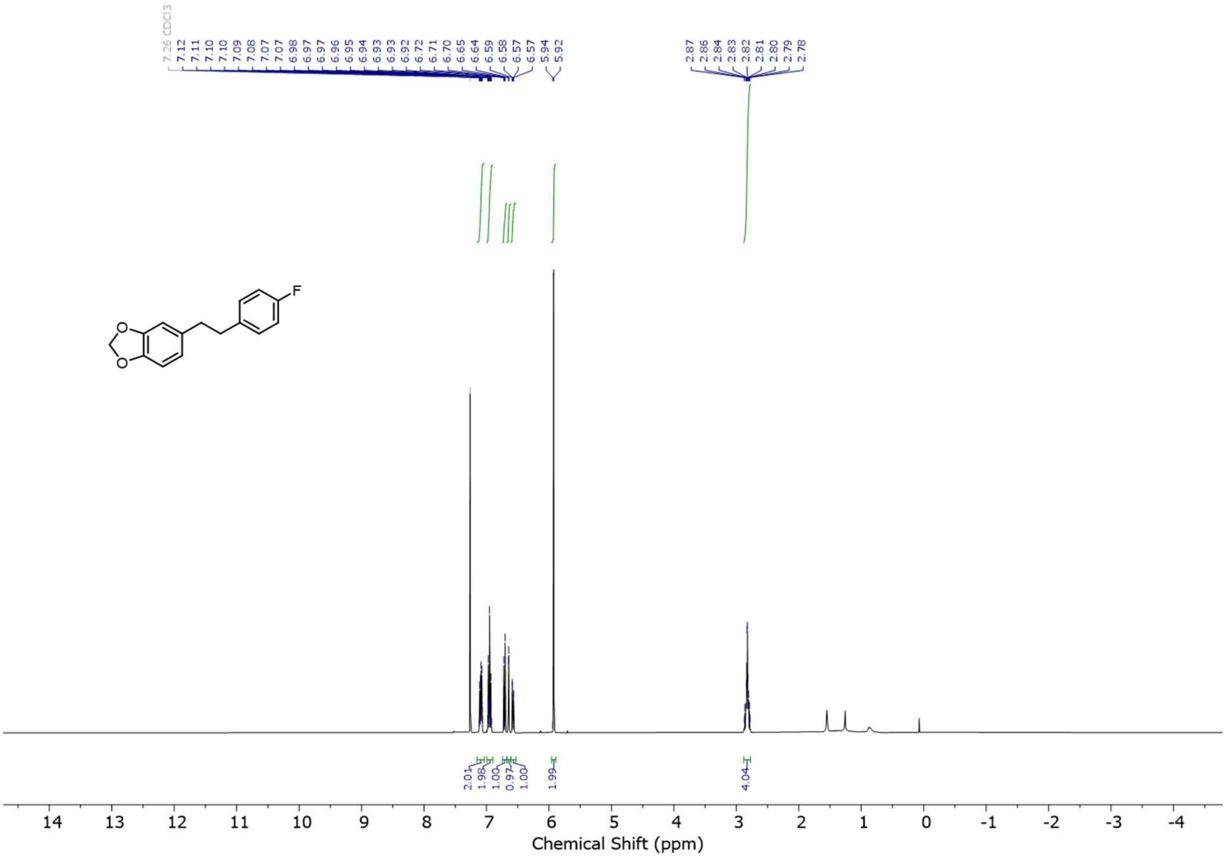

5-(4-Fluorophenethyl)benzo[*d*][1,3]dioxole (15) ^{13}C NMR (101 MHz, $CDCl_3$)

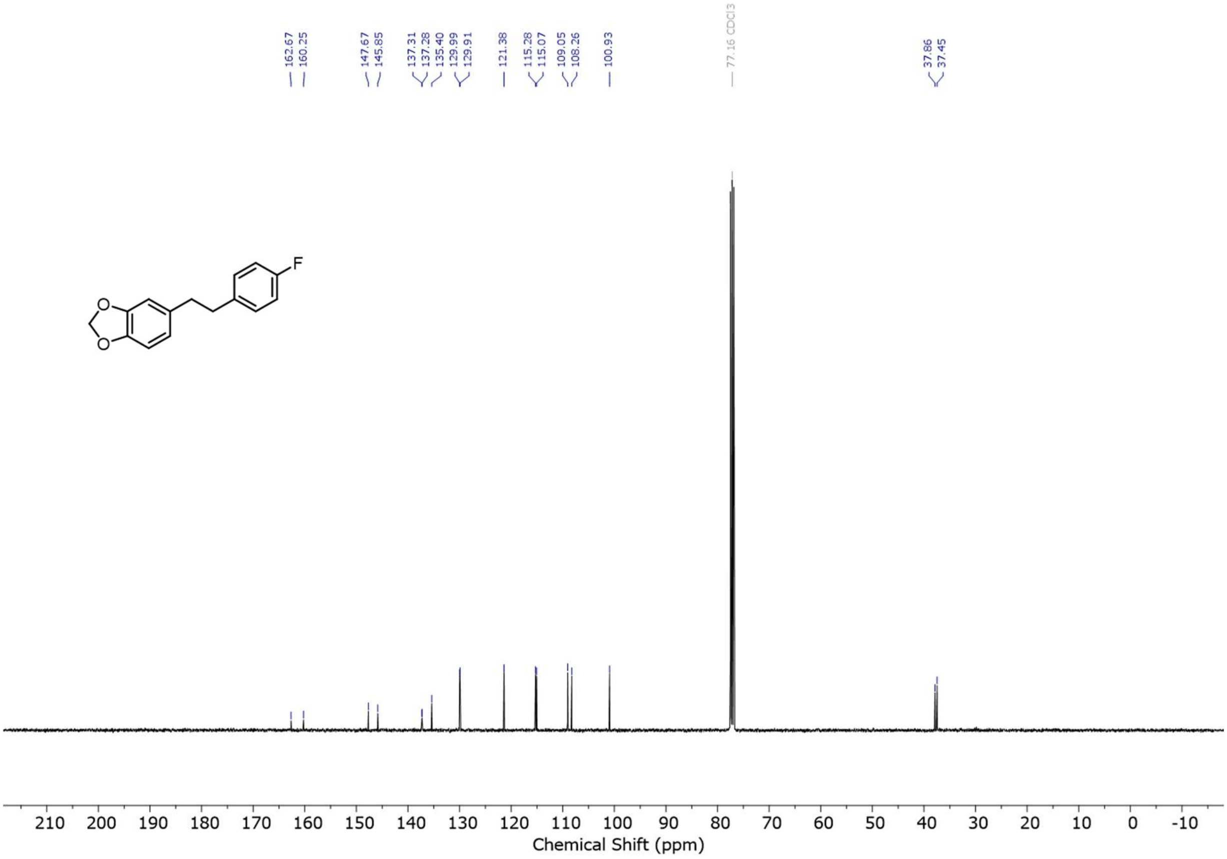

5-(4-Fluorophenethyl)benzo[*d*][1,3]dioxole (15) ^{19}F NMR (376 MHz, CDCl$_3$)

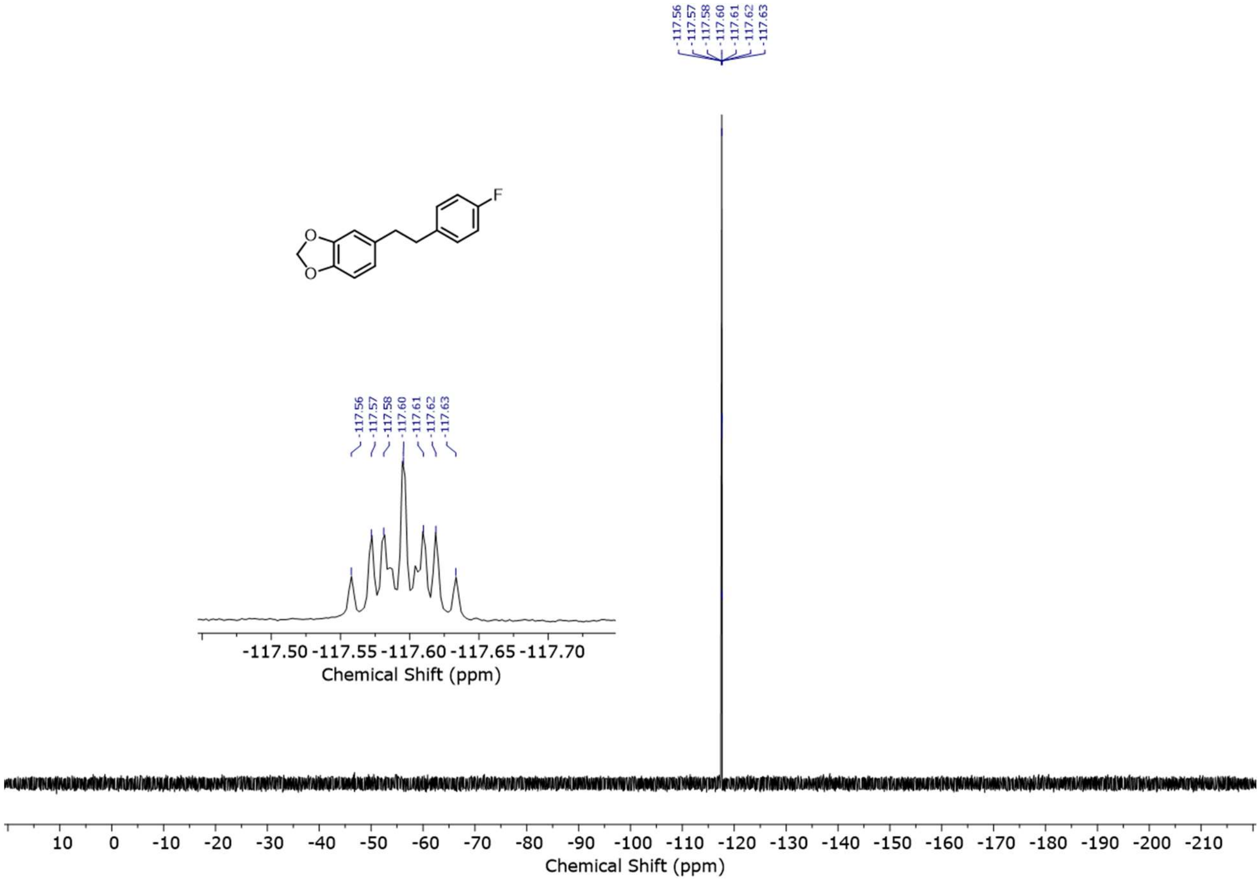

1-(2-(Cyclohex-1-en-1-yl)ethyl)-4-fluorobenzene (16) ^{1}H NMR (400 MHz, $CDCl_3$)

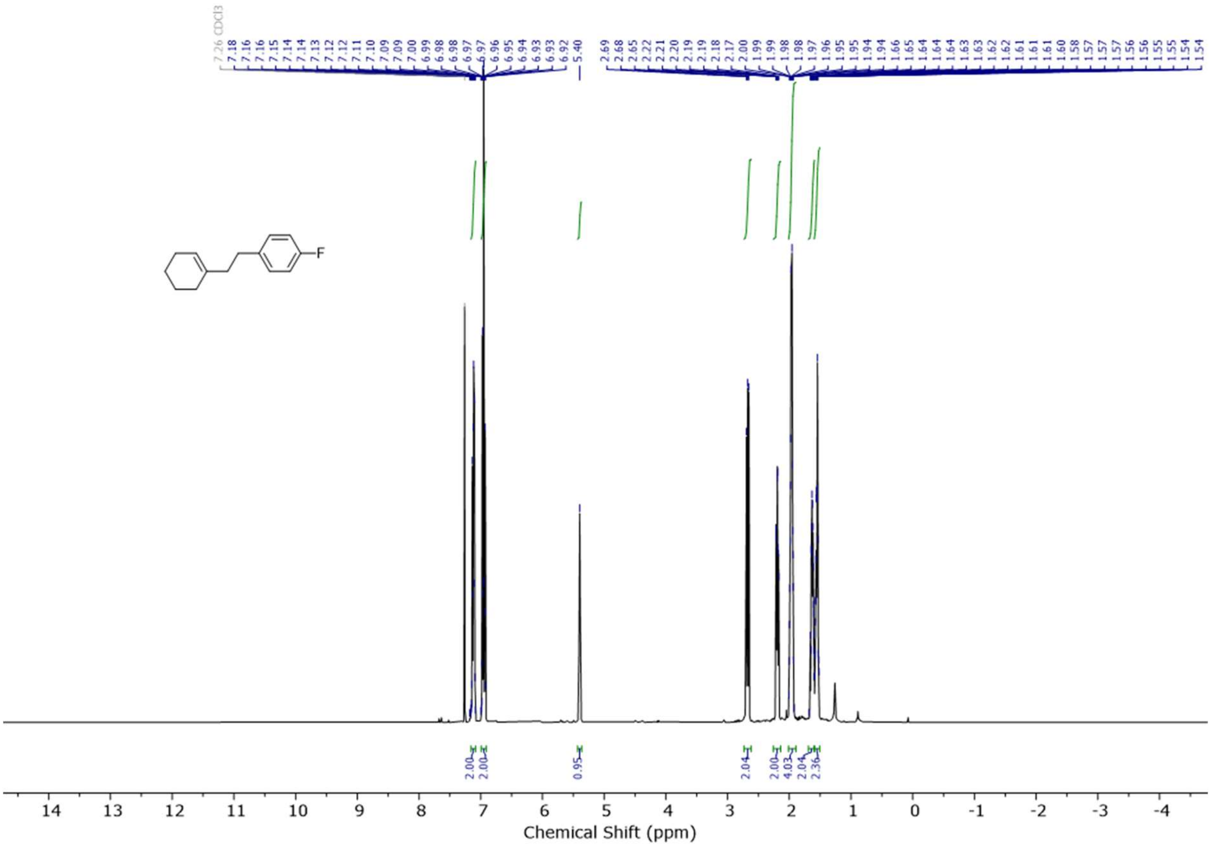

1-(2-(Cyclohex-1-en-1-yl)ethyl)-4-fluorobenzene (16) ^{13}C NMR (101 MHz, $CDCl_3$)

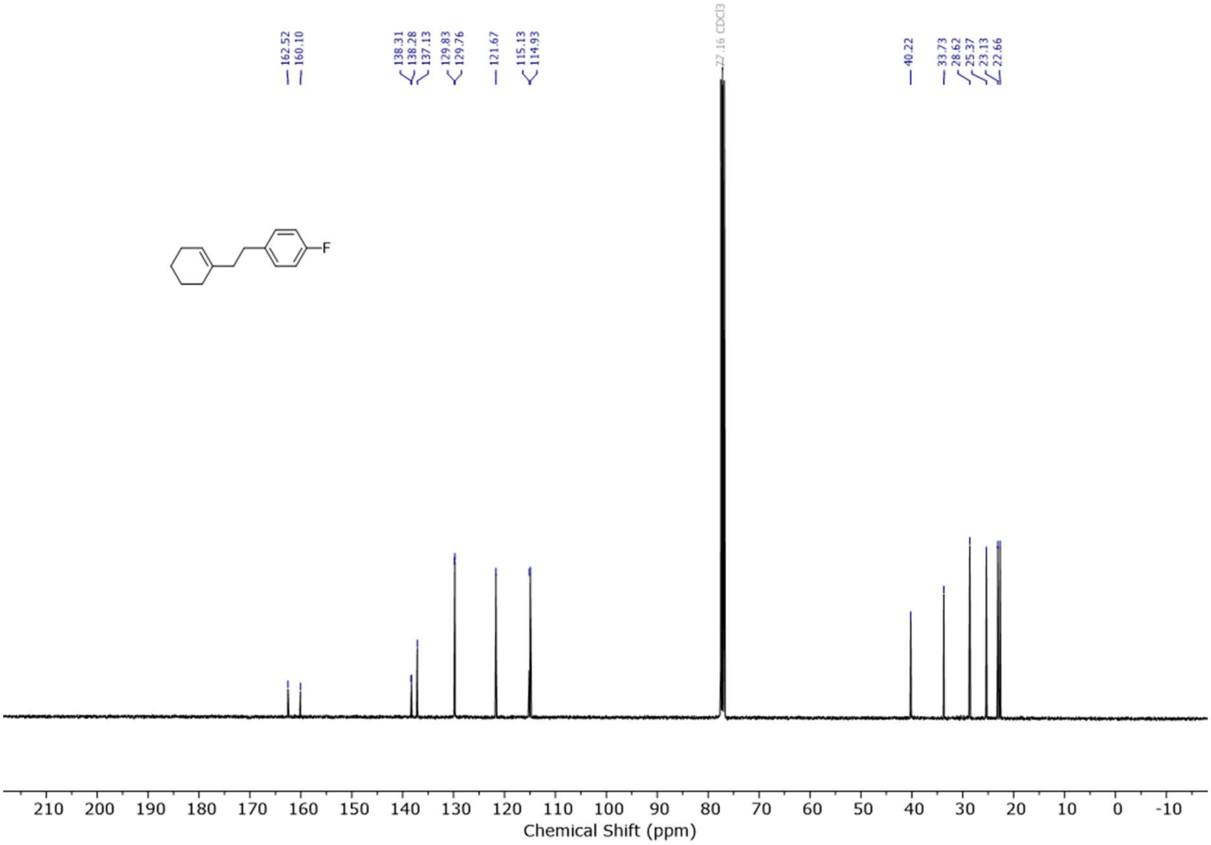

1-(2-(Cyclohex-1-en-1-yl)ethyl)-4-fluorobenzene (16) ^{19}F NMR (376 MHz, $CDCl_3$)

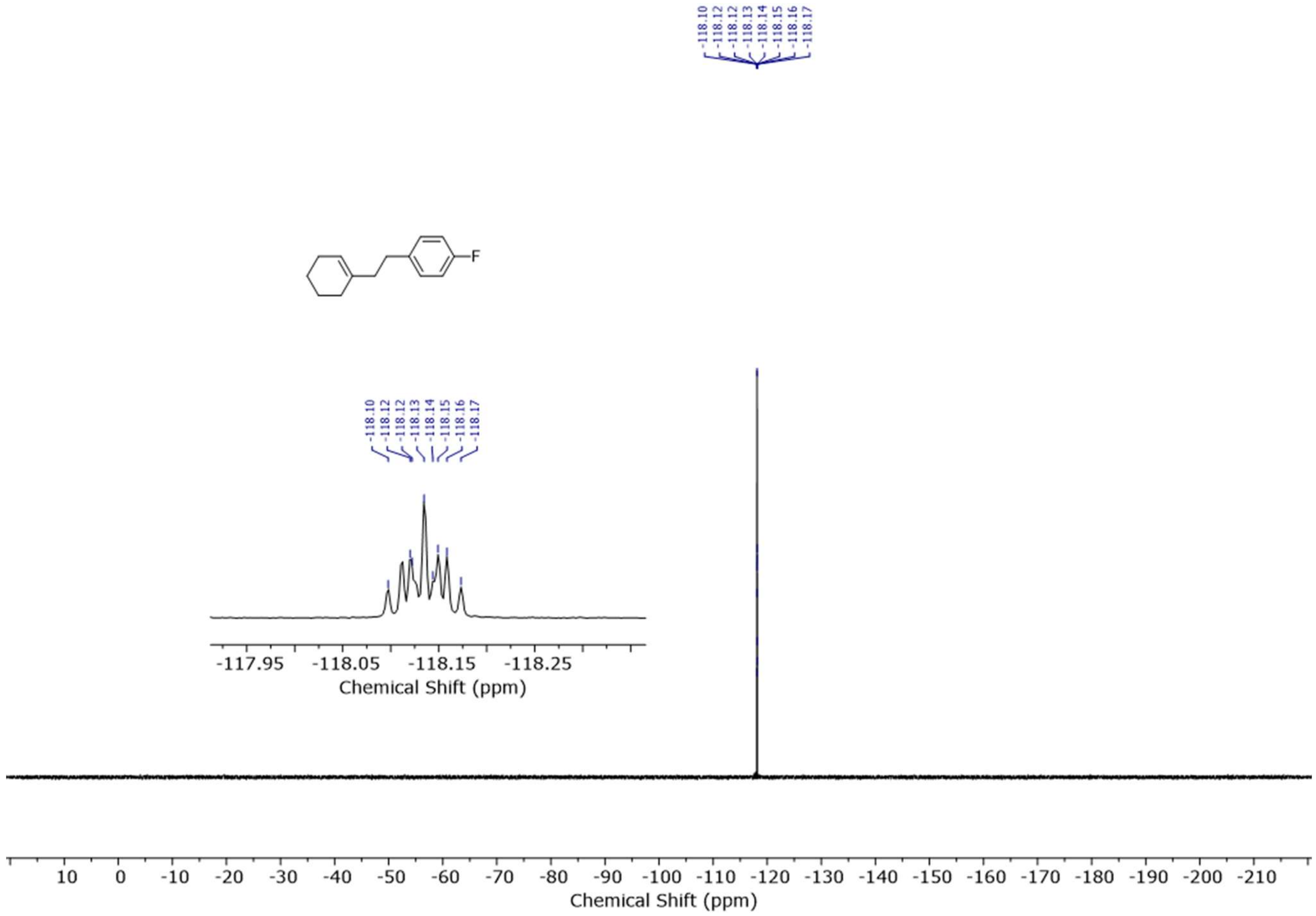

(*1S,2S,5S*)-2-(4-Fluorobenzyl)-6,6-dimethylbicyclo[3.1.1]heptane (17) ^{1}H NMR (400 MHz, CDCl$_3$)

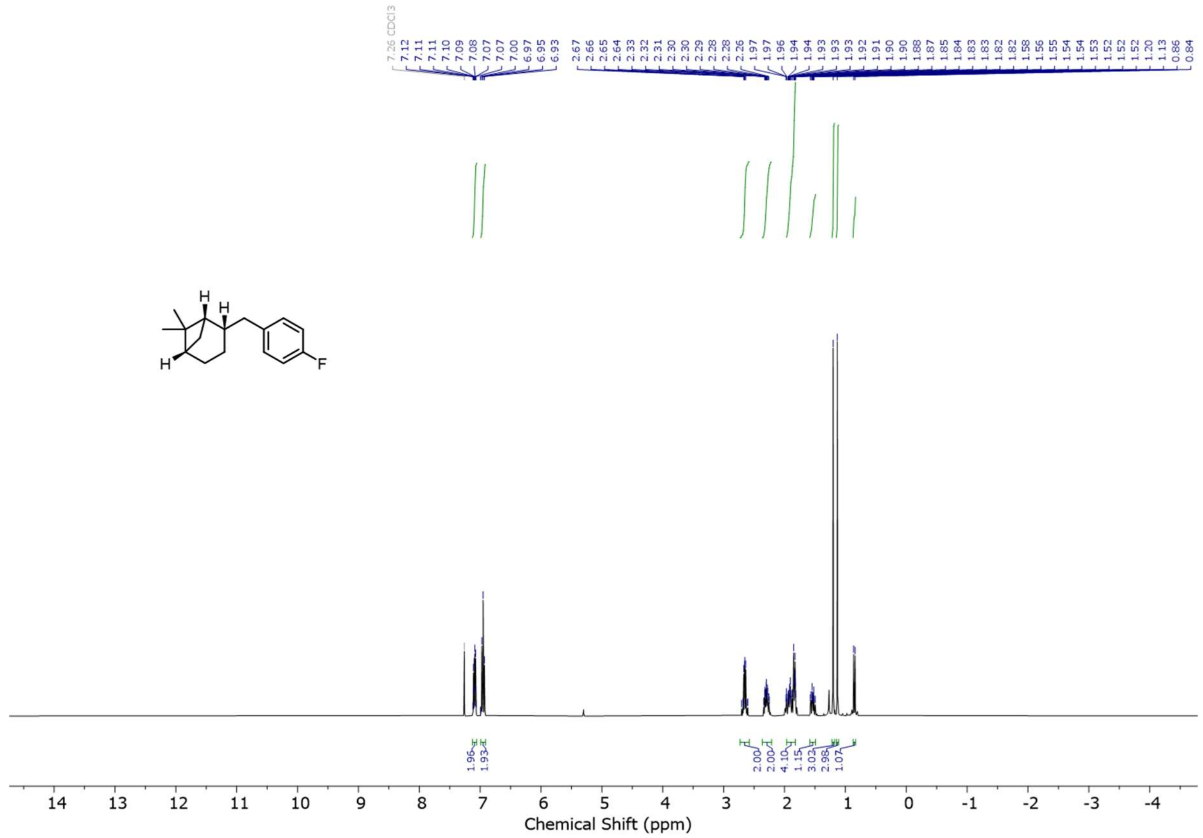

(*1S,2S,5S*)-2-(4-Fluorobenzyl)-6,6-dimethylbicyclo[3.1.1]heptane (17) ^{13}C NMR (101 MHz, CDCl$_3$)

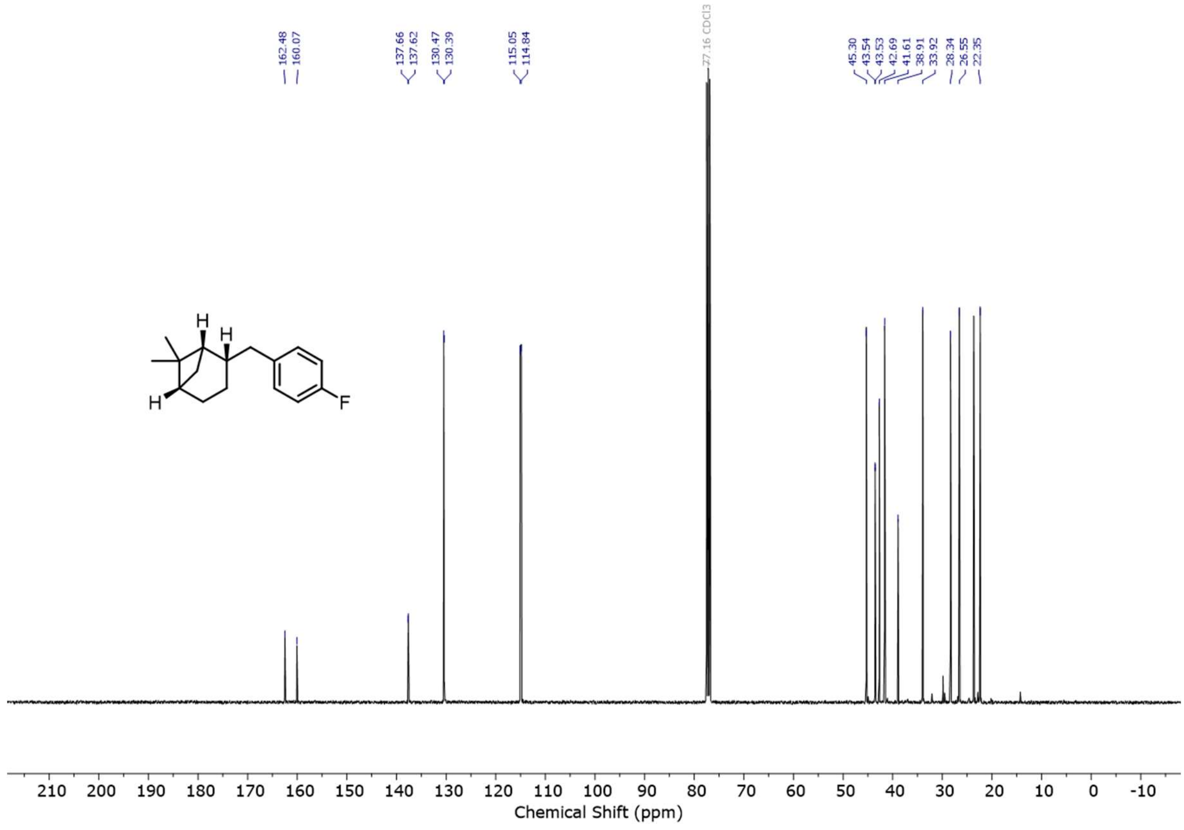

(*1S,2S,5S*)-2-(4-Fluorobenzyl)-6,6-dimethylbicyclo[3.1.1]heptane (17) ^{19}F NMR (376 MHz, $CDCl_3$)

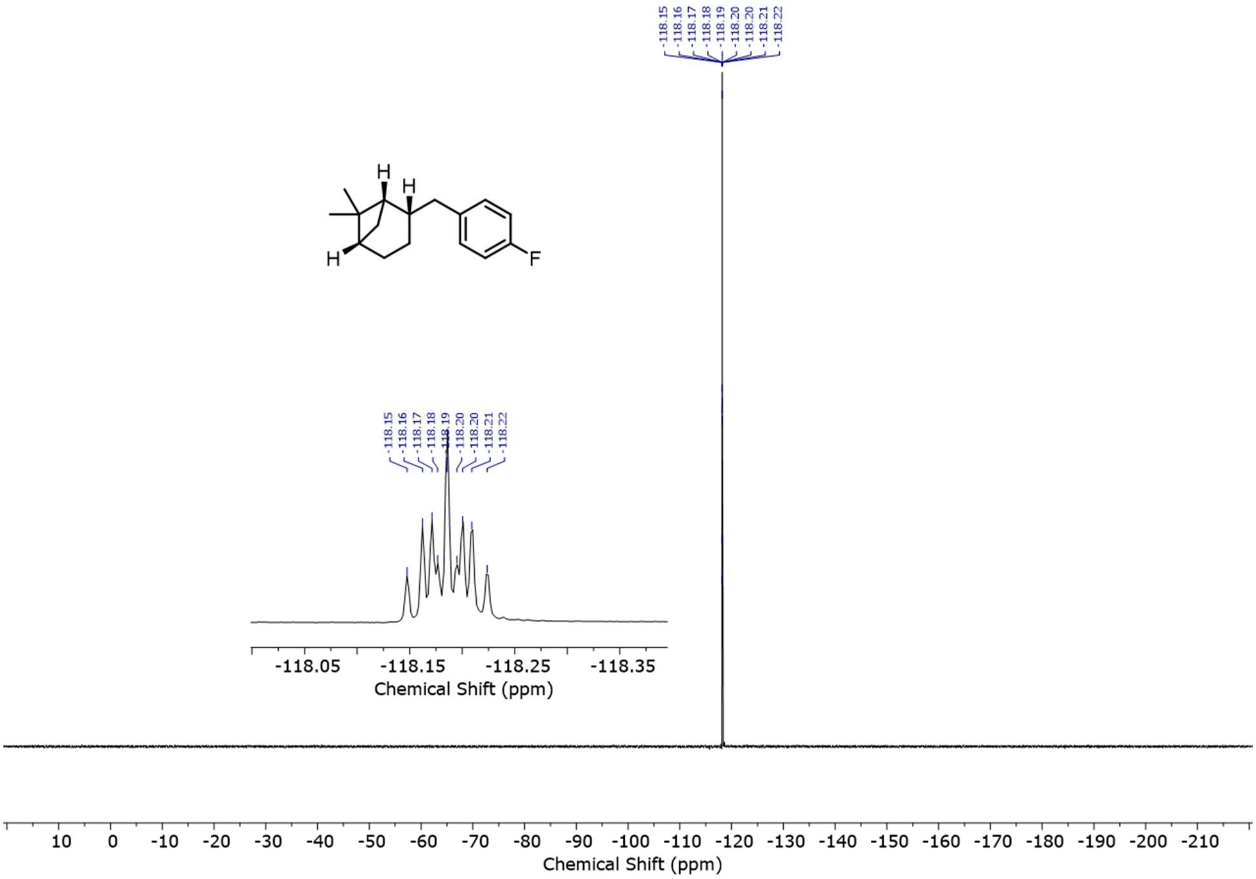

***tert*-Butyl 2-((*4R,6R*)-6-(4-fluorophenethyl)-2,2-dimethyl-1,3-dioxan-4-yl)acetate (18) ^{1}H NMR (400 MHz, $CDCl_3$)**

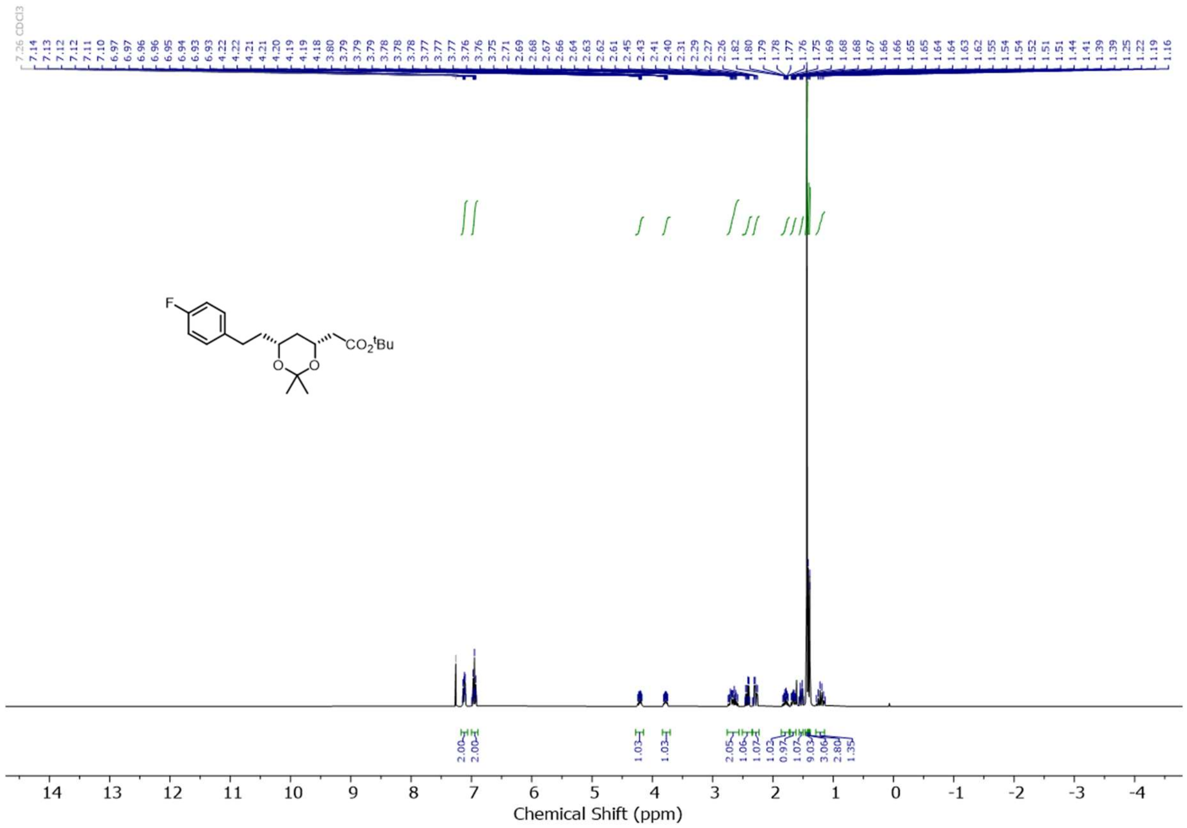

***tert*-Butyl 2-((*4R,6R*)-6-(4-fluorophenethyl)-2,2-dimethyl-1,3-dioxan-4-yl)acetate (18) ^{13}C NMR (101 MHz, $CDCl_3$)**

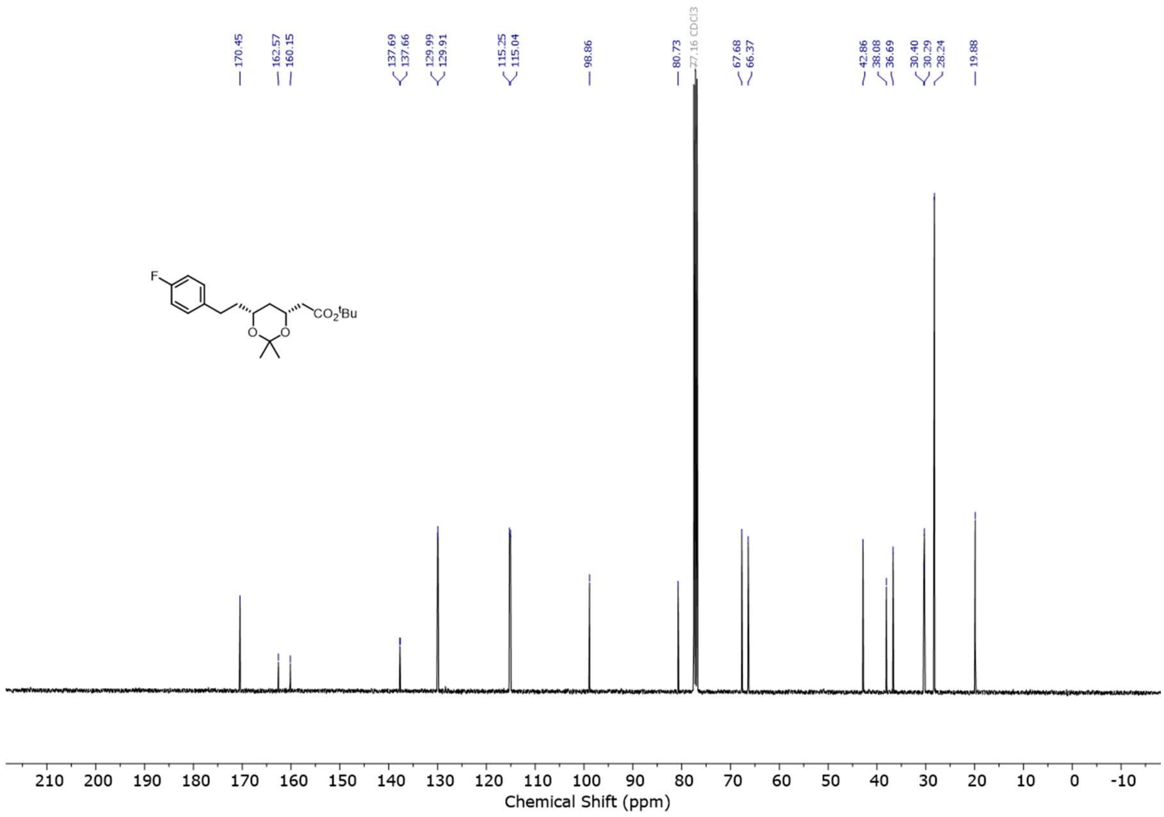

***tert*-Butyl 2-((4*R*,6*R*)-6-(4-fluorophenethyl)-2,2-dimethyl-1,3-dioxan-4-yl)acetate (18) ^{19}F NMR (376 MHz, $CDCl_3$)**

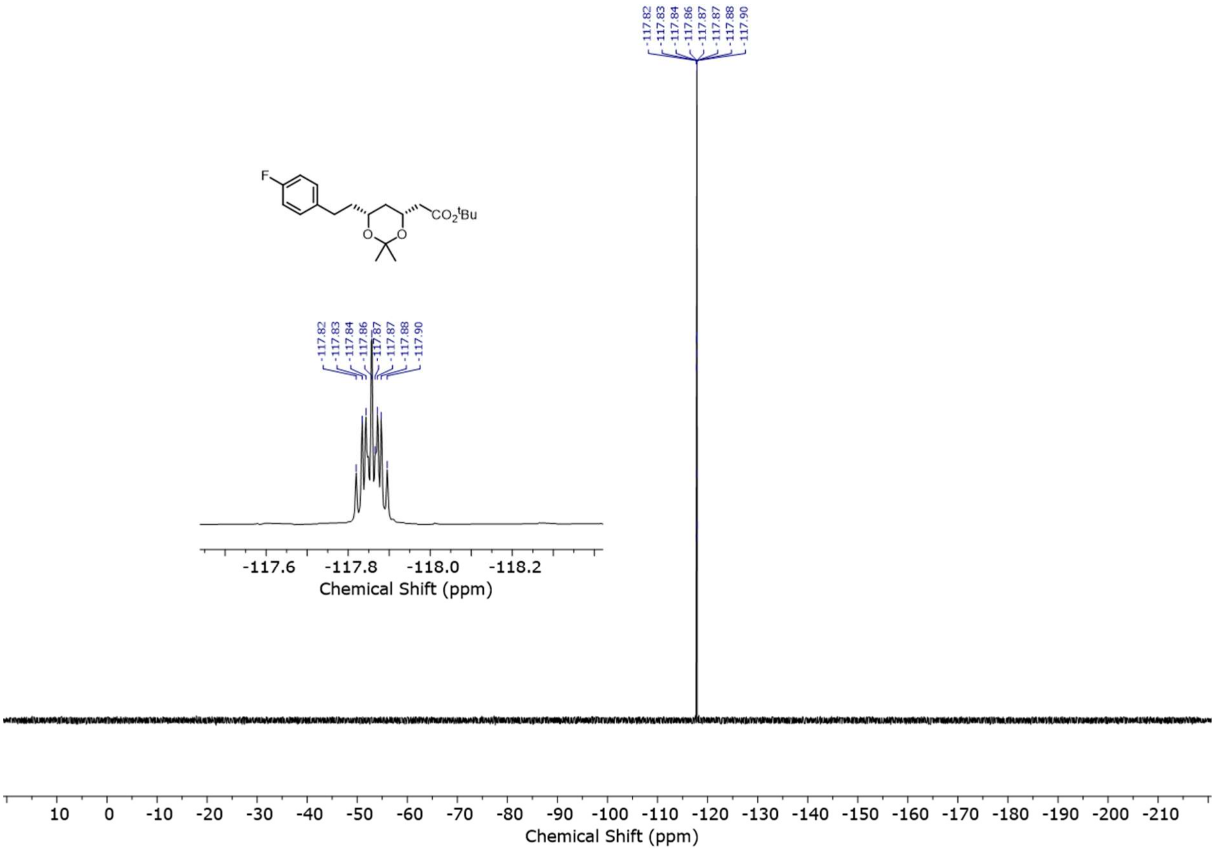

(*E*)-5-Methoxy-1-(4-(trifluoromethyl)phenyl)pentan-1-one *O*-(4-fluorophenethyl) oxime (19) ^{1}H NMR (500 MHz, $CDCl_3$)

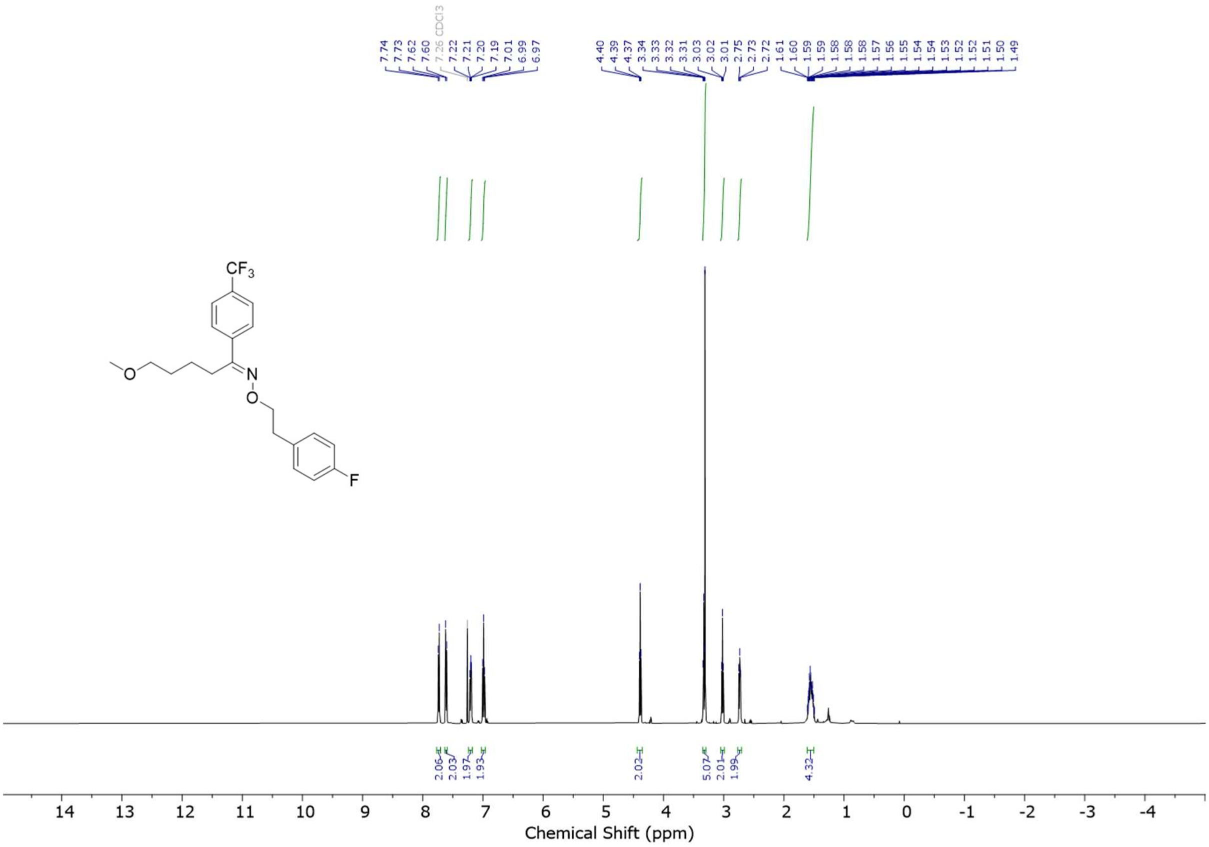

(*E*)-5-Methoxy-1-(4-(trifluoromethyl)phenyl)pentan-1-one *O*-(4-fluorophenethyl) oxime (19) {^{19}F}^{13}C NMR (126 MHz, $CDCl_3$)

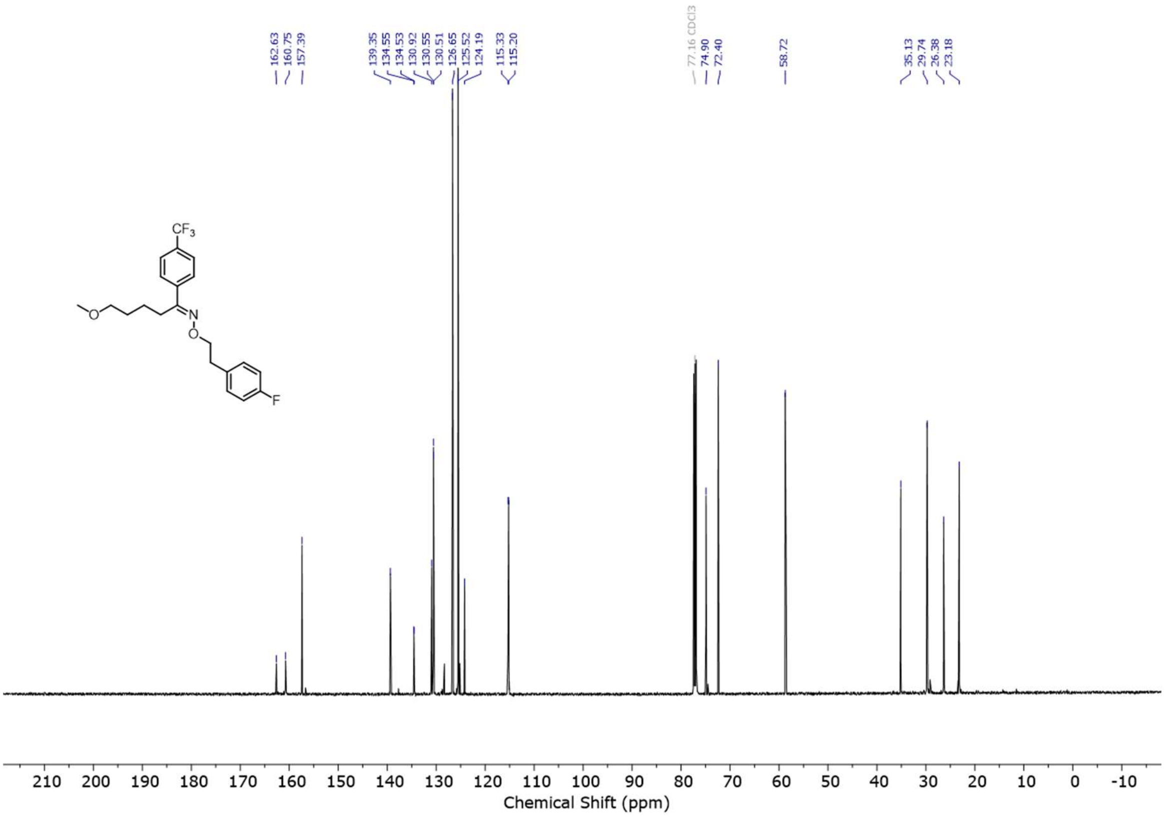

(*E*)-5-Methoxy-1-(4-(trifluoromethyl)phenyl)pentan-1-one *O*-(4-fluorophenethyl) oxime (19) ^{19}F NMR (471 MHz, $CDCl_3$)

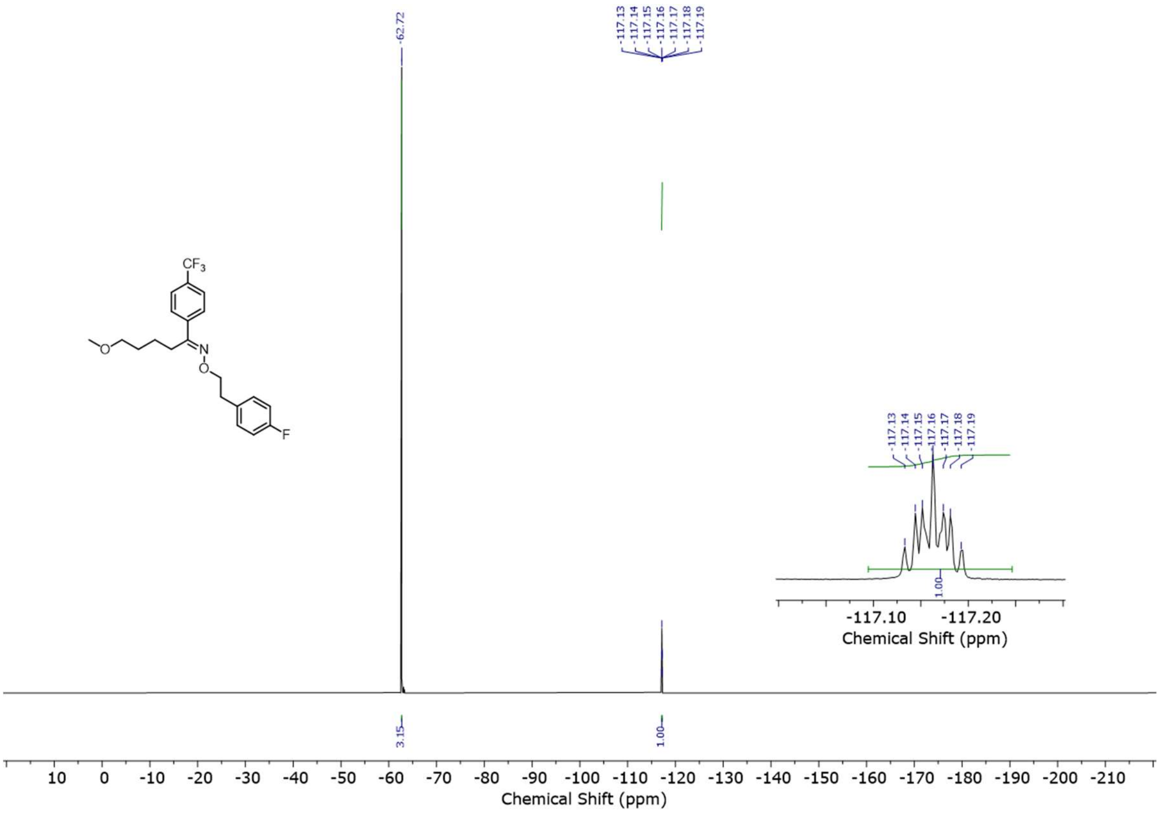

***N*-(5-(4-Fluorophenyl)pentan-2-yl)-6-methoxyquinolin-8-amine (20) ^{1}H NMR (400 MHz, $CDCl_3$)**

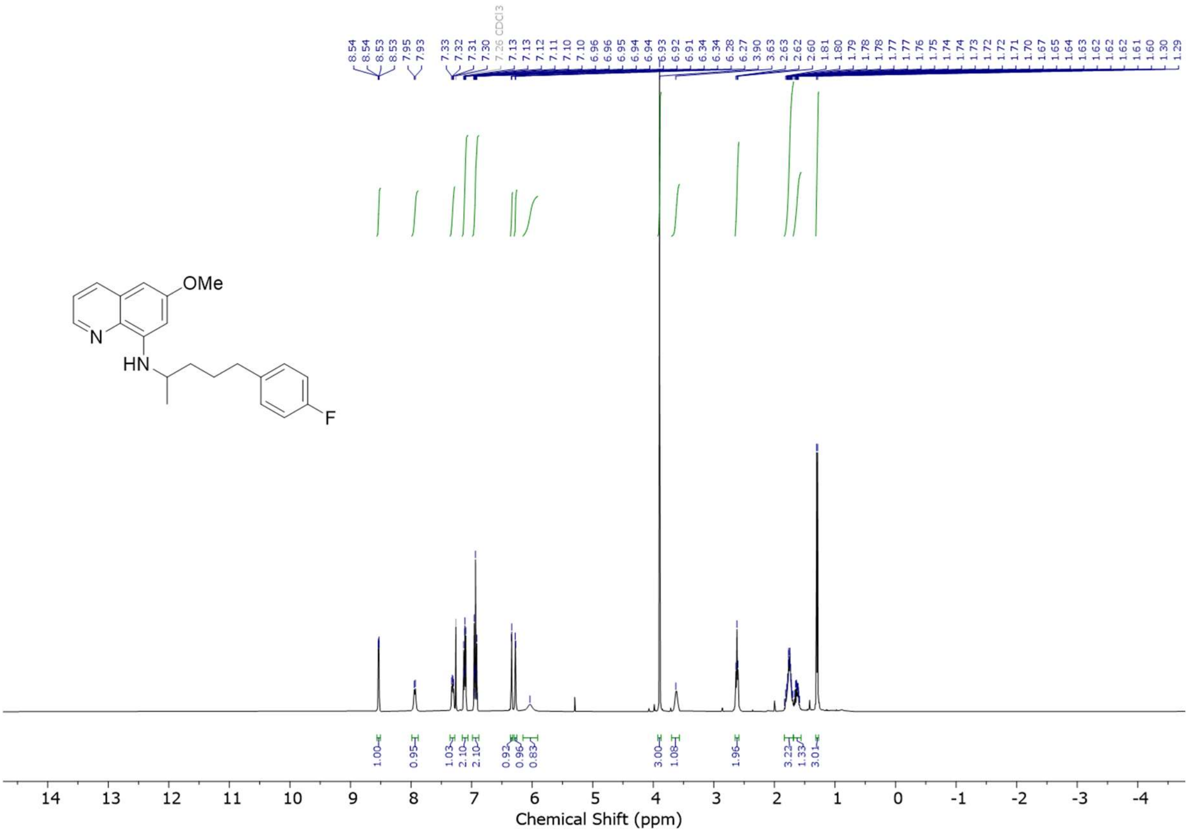

***N*-(5-(4-Fluorophenyl)pentan-2-yl)-6-methoxyquinolin-8-amine (20) ^{13}C NMR (101 MHz, $CDCl_3$)**

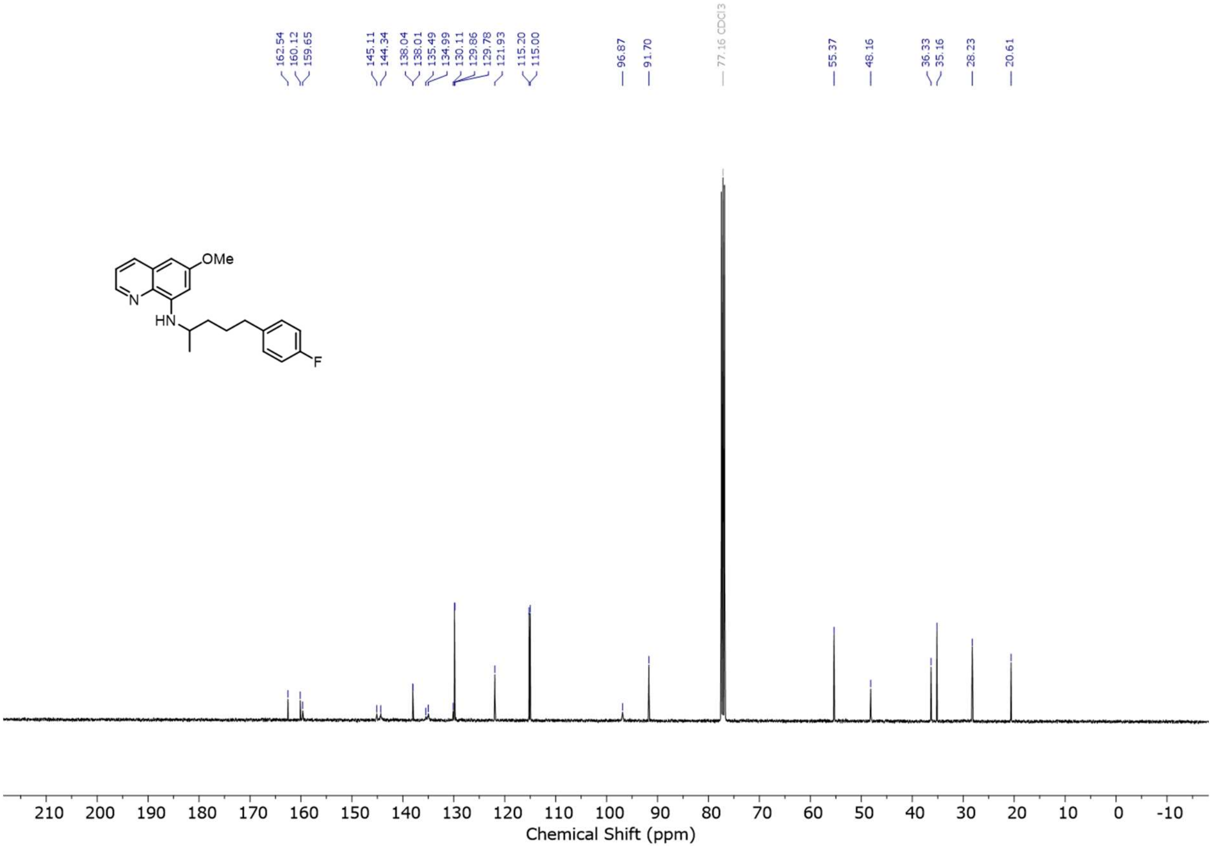

_N_-(5-(4-Fluorophenyl)pentan-2-yl)-6-methoxyquinolin-8-amine (20) ^{19}F NMR (377 MHz, $CDCl_3$)

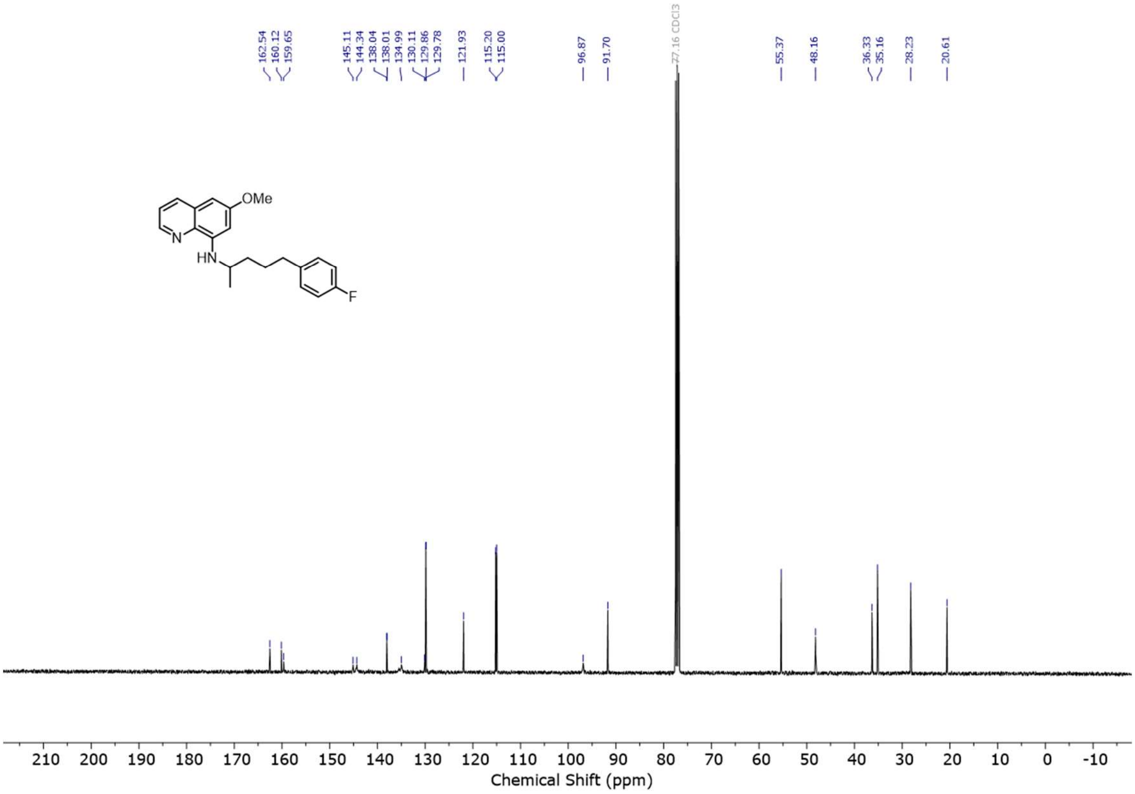

3-Ethyl 5-methyl 2-((4-fluorophenethoxy)methyl)-6-methyl-4-(o-tolyl)-1,4-dihydropyridine-3,5-dicarboxylate (21) ^{1}H NMR (400 MHz, $CDCl_3$)

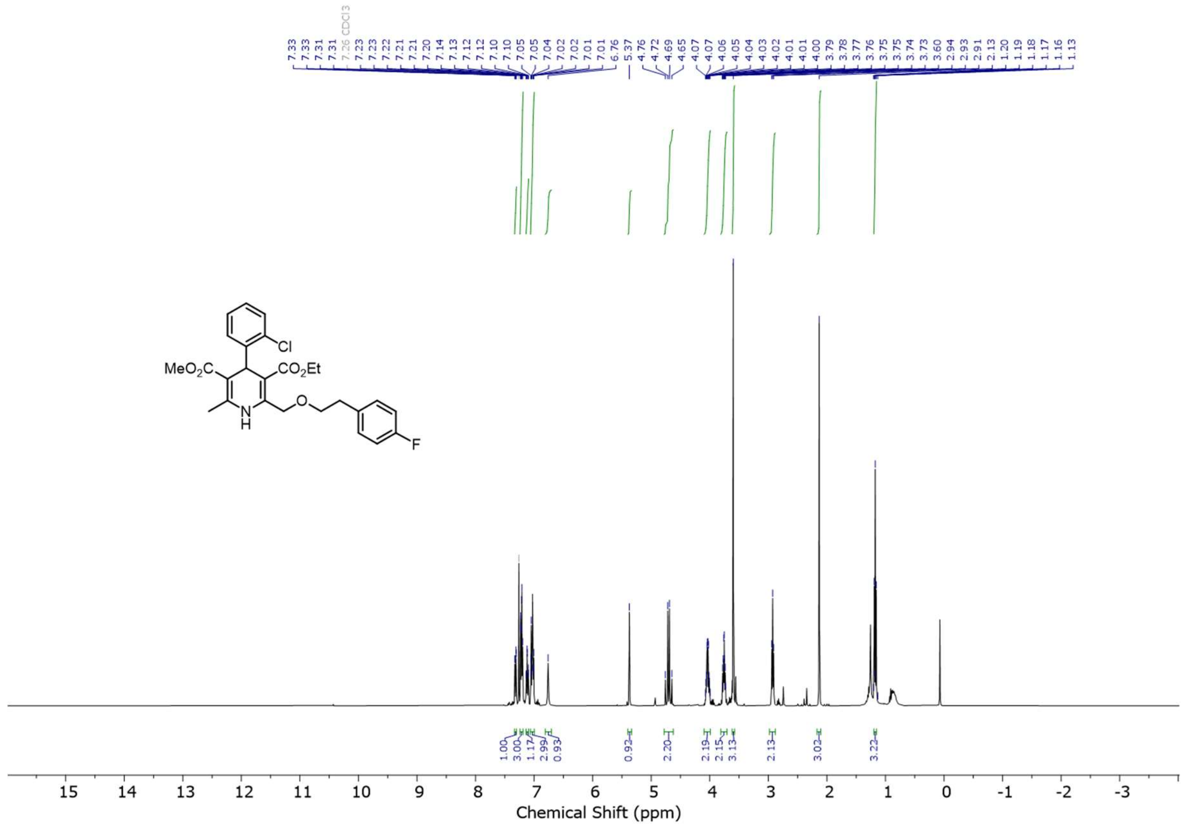

3-Ethyl 5-methyl 2-((4-fluorophenethoxy)methyl)-6-methyl-4-(o-tolyl)-1,4-dihydropyridine-3,5-dicarboxylate (21) ^{13}C NMR (101 MHz, $CDCl_3$)

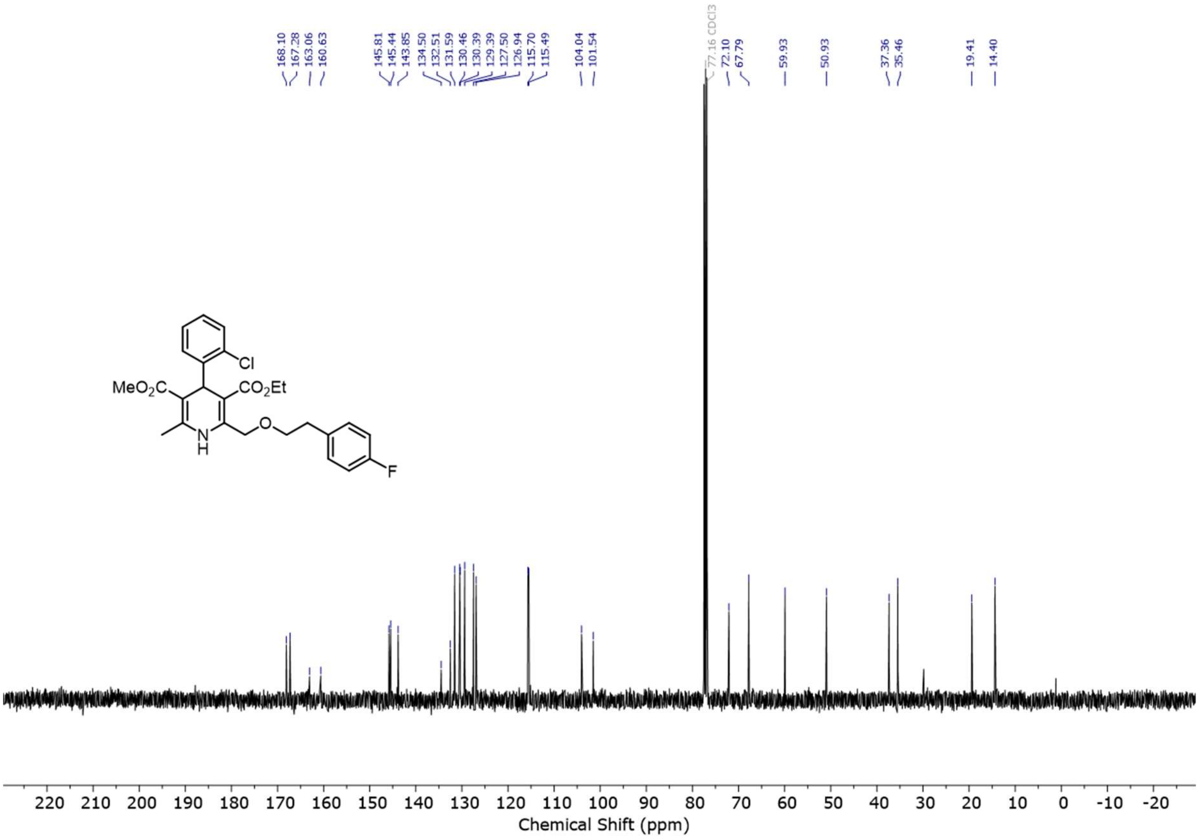

3-Ethyl 5-methyl 2-((4-fluorophenethoxy)methyl)-6-methyl-4-(o-tolyl)-1,4-dihydropyridine-3,5-dicarboxylate (21) ^{19}F NMR (377 MHz, $CDCl_3$)

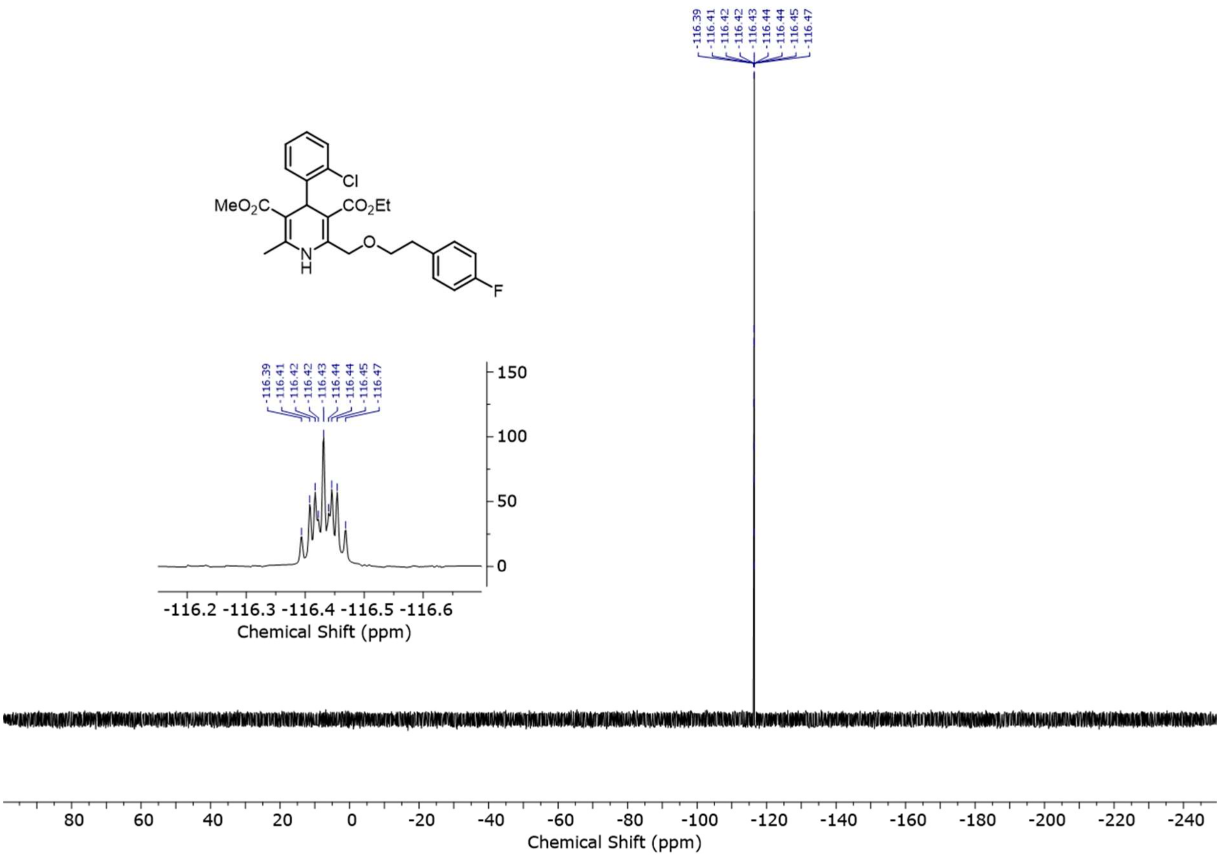

1-(2-(4-Fluorophenyl)-1-(4-methoxyphenyl)ethyl)cyclohexan-1-ol (22) ^{1}H NMR (400 MHz, $CDCl_3$)

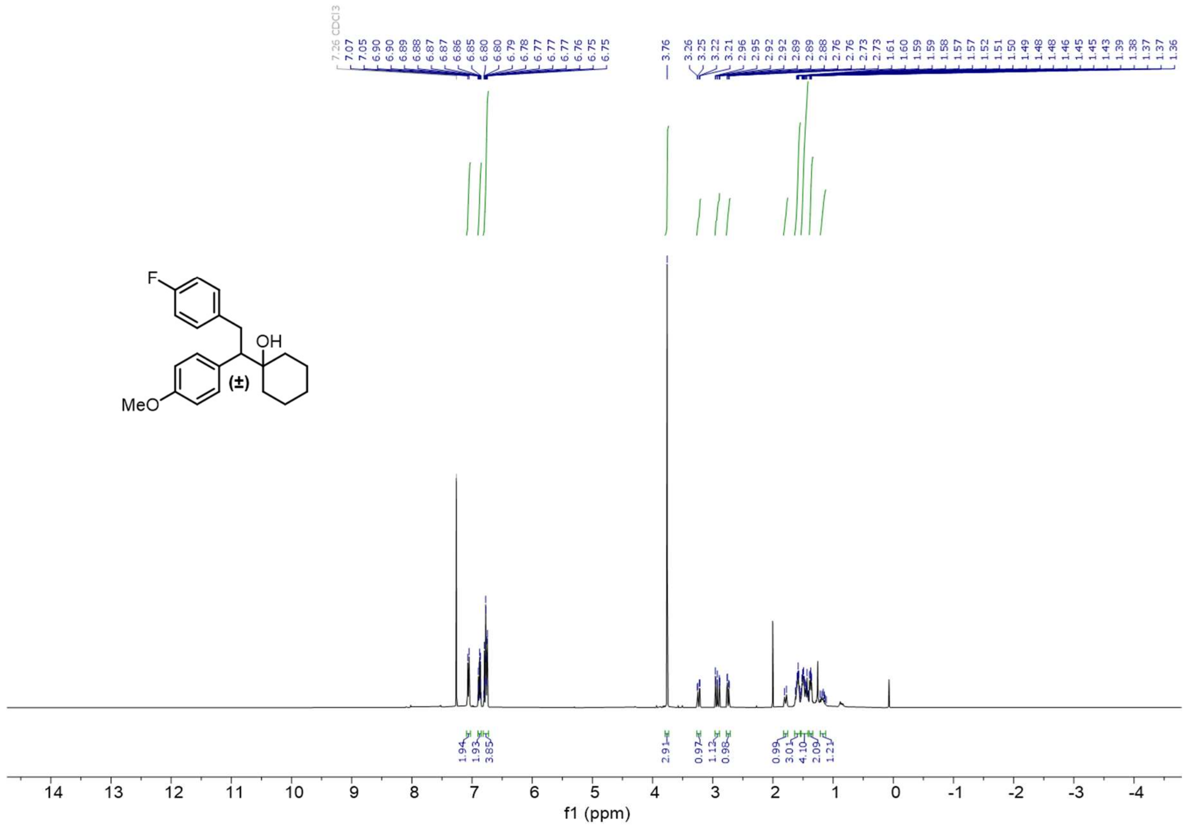

1-(2-(4-Fluorophenyl)-1-(4-methoxyphenyl)ethyl)cyclohexan-1-ol (22) ^{13}C NMR (101 MHz, $CDCl_3$)

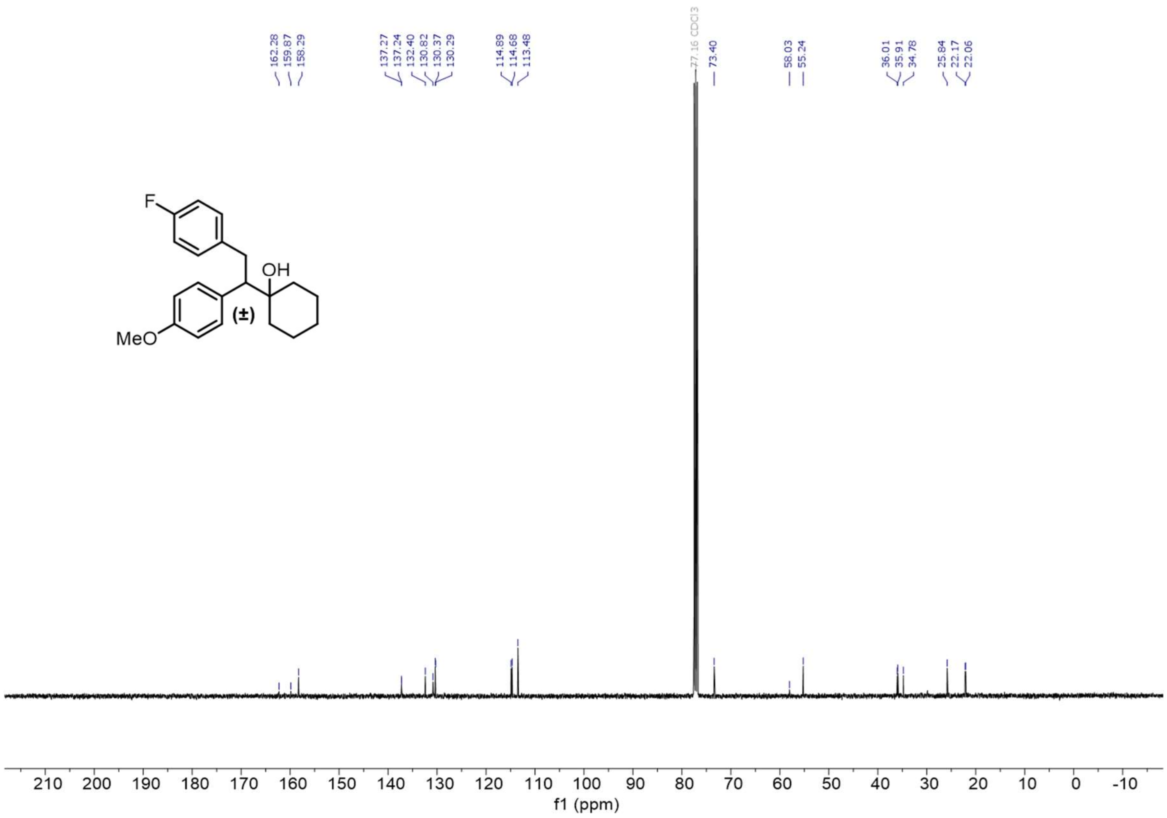

1-(2-(4-Fluorophenyl)-1-(4-methoxyphenyl)ethyl)cyclohexan-1-ol (22) {^{1}H}^{19}F NMR (376 MHz, $CDCl_3$)

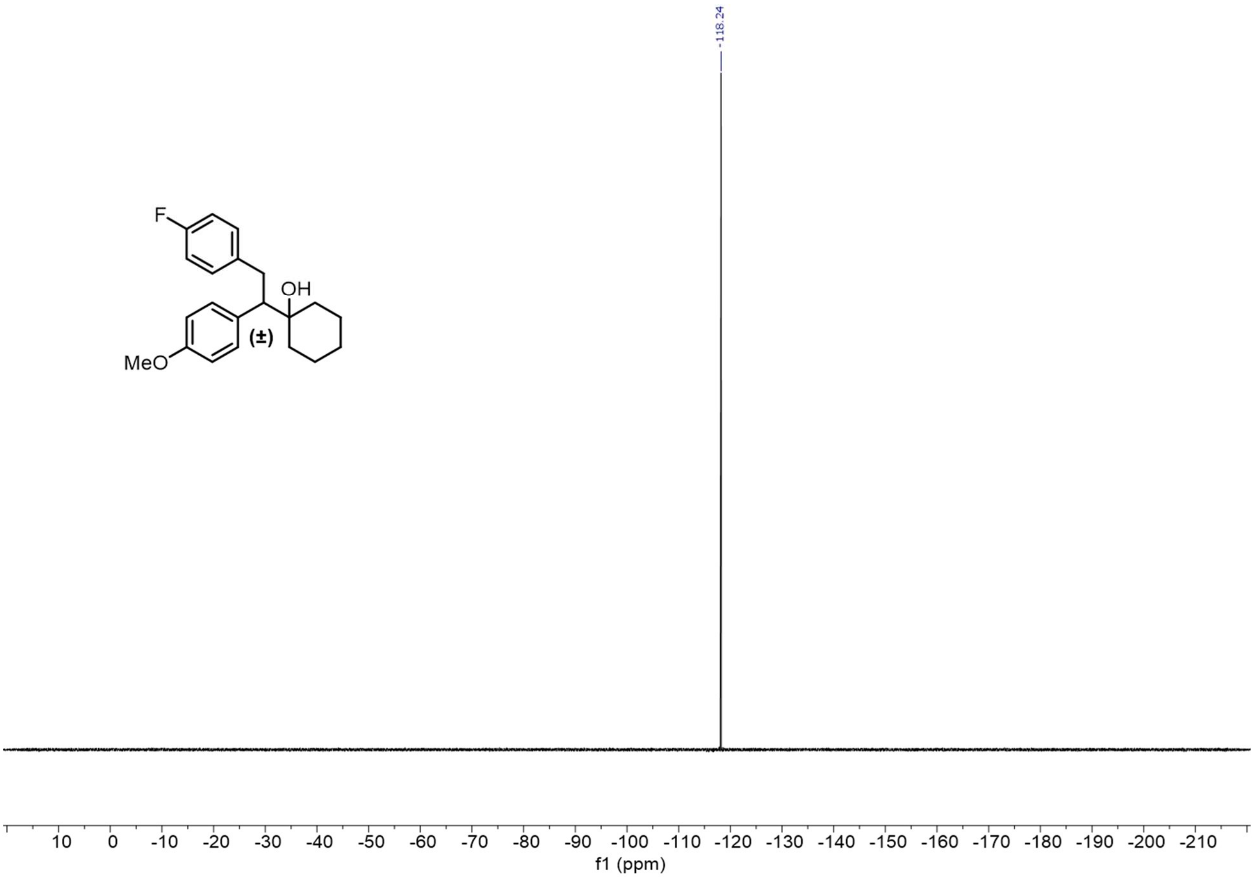

7-Methyl (*S*)-2-((*tert*-butoxycarbonyl)amino)-6-(4-fluorophenyl)hexanoate (23) ^{1}H NMR (600 MHz, $CDCl_3$)

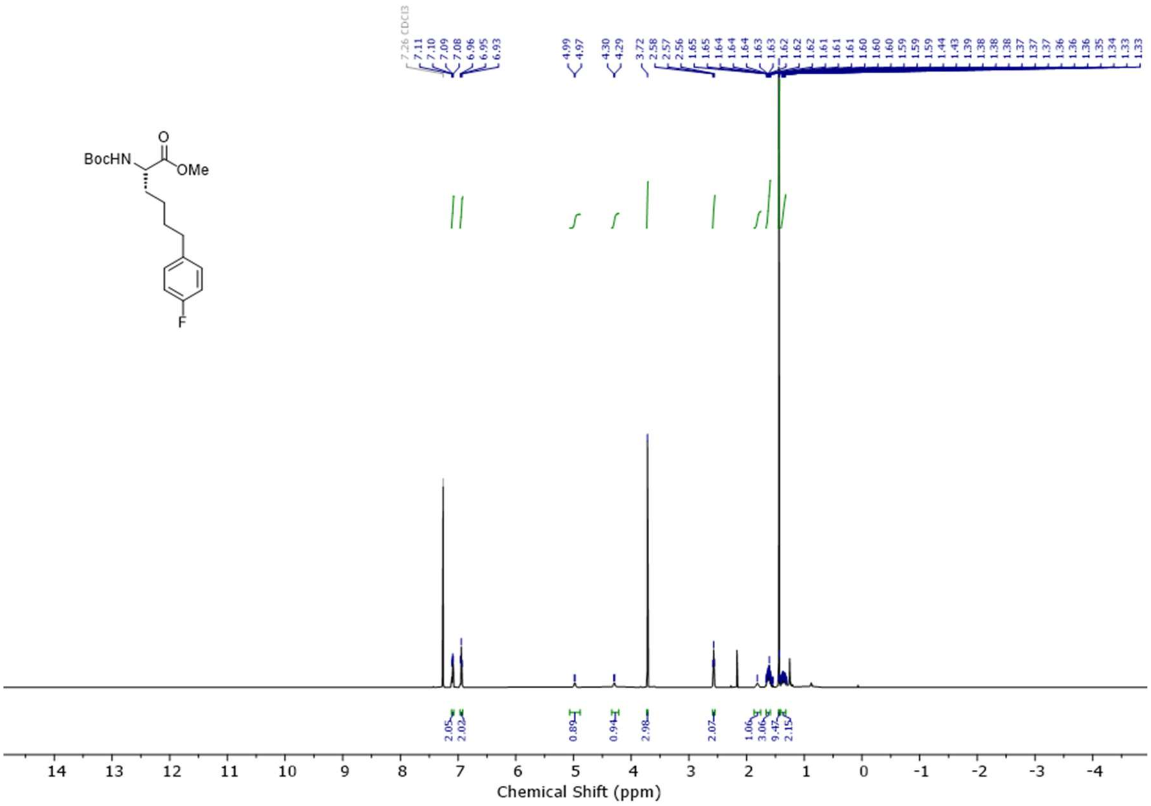

Methyl (*S*)-2-((*tert*-butoxycarbonyl)amino)-6-(4-fluorophenyl)hexanoate (23) {^{1}H}^{19}F NMR (565 MHz, $CDCl_3$)

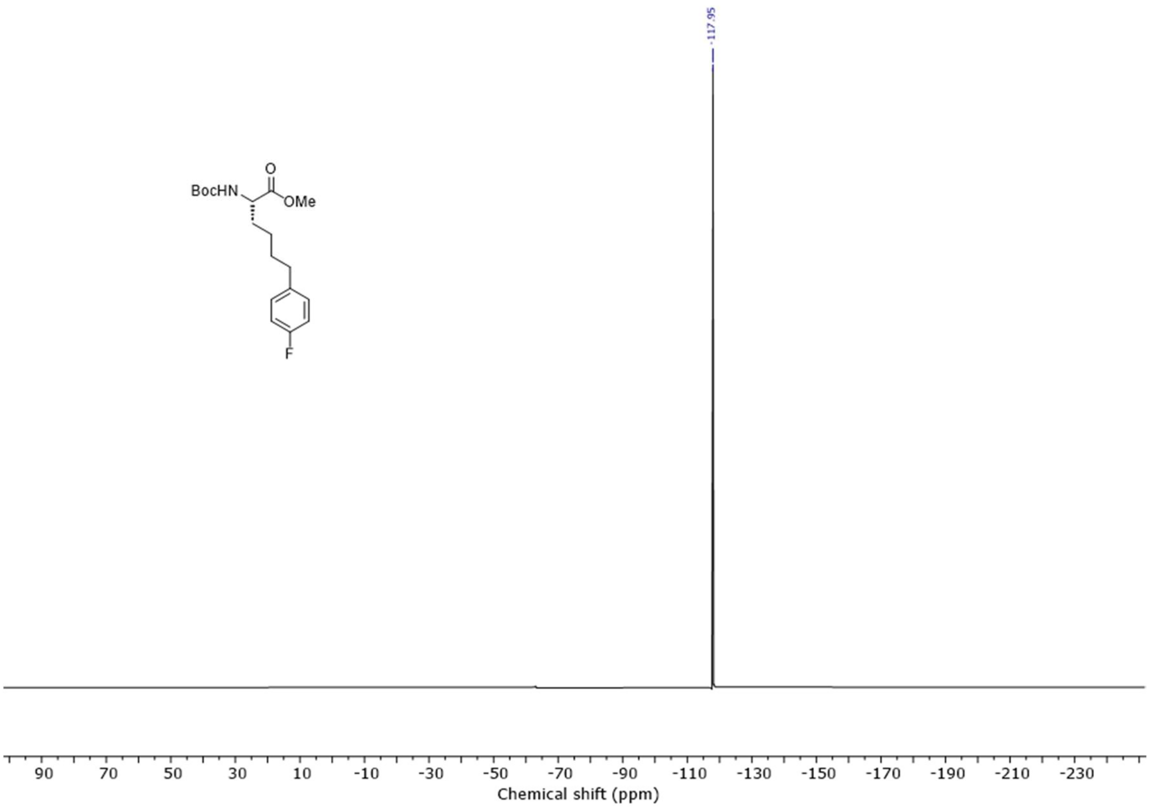

Methyl (*S*)-2-((*tert*-butoxycarbonyl)amino)-6-(4-fluorophenyl)hexanoate (23) ^{13}C NMR (151 MHz, $CDCl_3$)

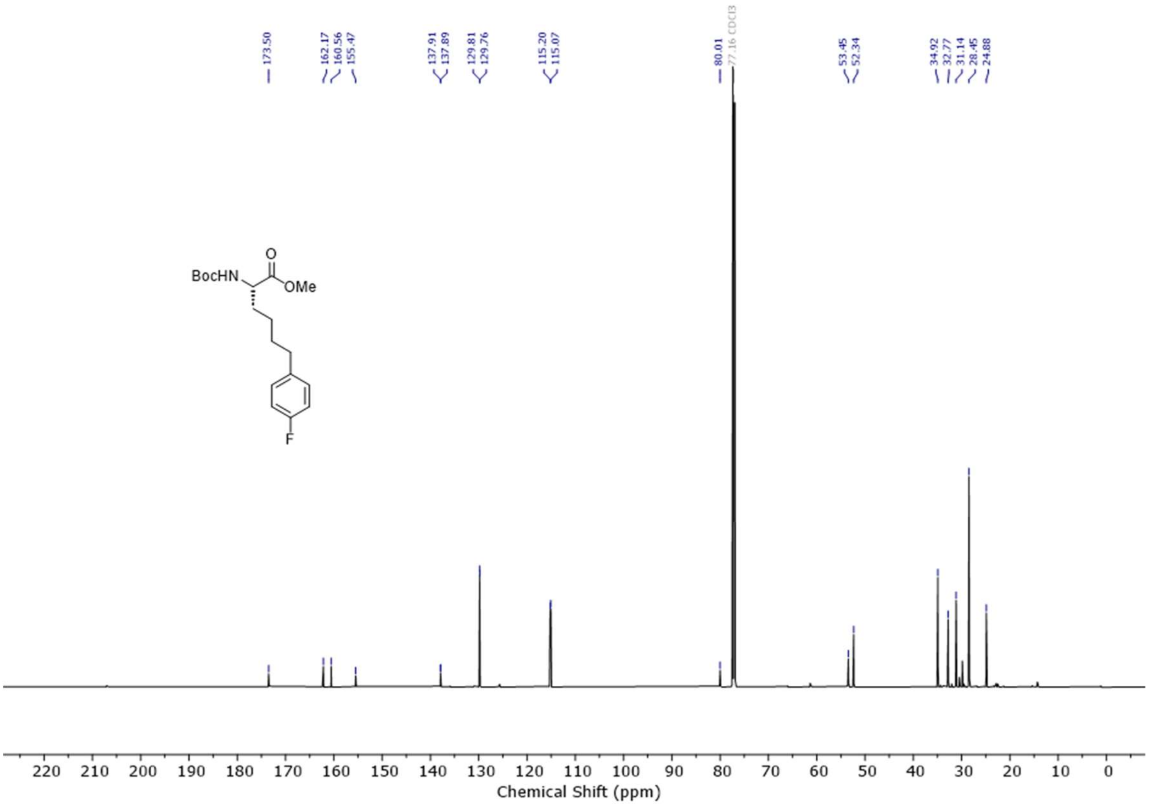

Di-*tert*-butyl (((*S*)-1-(*tert*-butoxy)-6-(4-fluorophenyl)-1-oxohexan-2-yl)carbamoyl)-*L*-glutamate (24) ^{1}H NMR (500 MHz, $CDCl_3$)

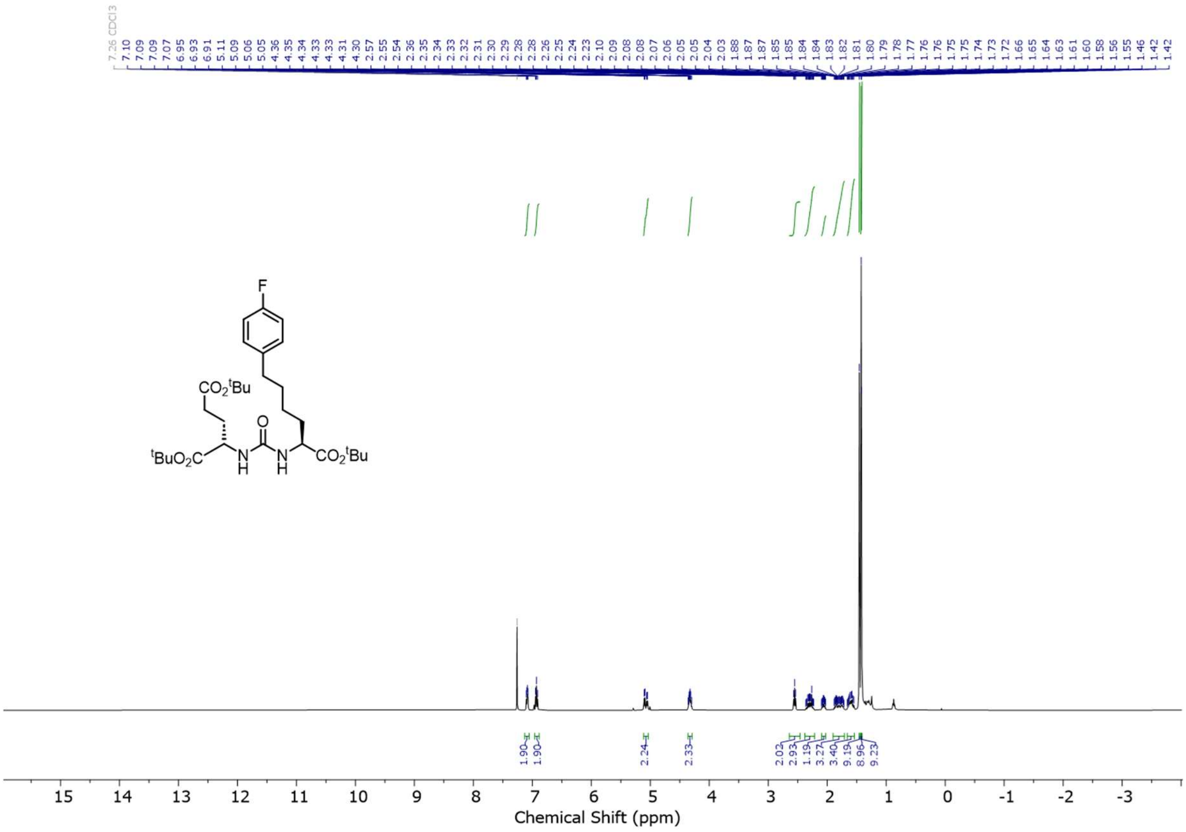

Di-*tert*-butyl (((*S*)-1-(*tert*-butoxy)-6-(4-fluorophenyl)-1-oxohexan-2-yl)carbamoyl)-*L*-glutamate (24) ^{13}C NMR (126 MHz, $CDCl_3$)

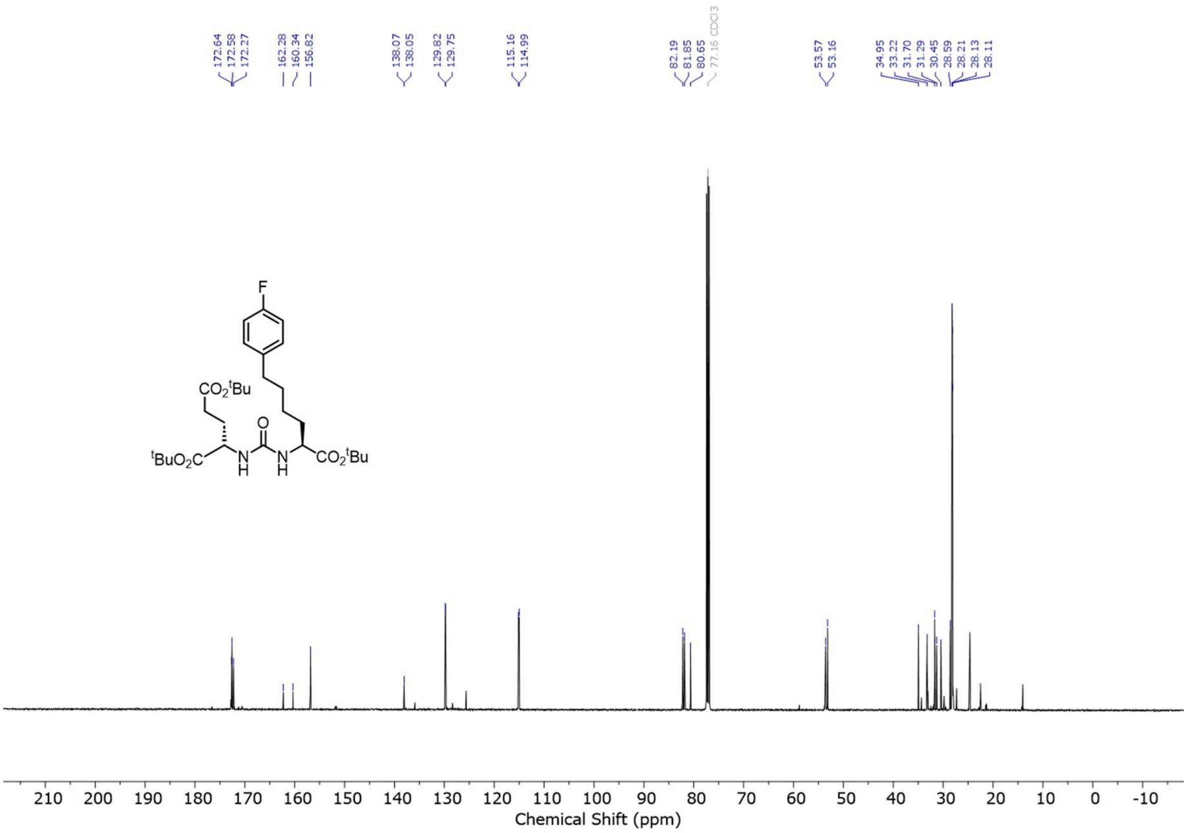

Di-*tert*-butyl (((*S*)-1-(*tert*-butoxy)-6-(4-fluorophenyl)-1-oxohexan-2-yl)carbamoyl)-*L*-glutamate (24) ^{19}F NMR (470 MHz, CDCl$_3$)

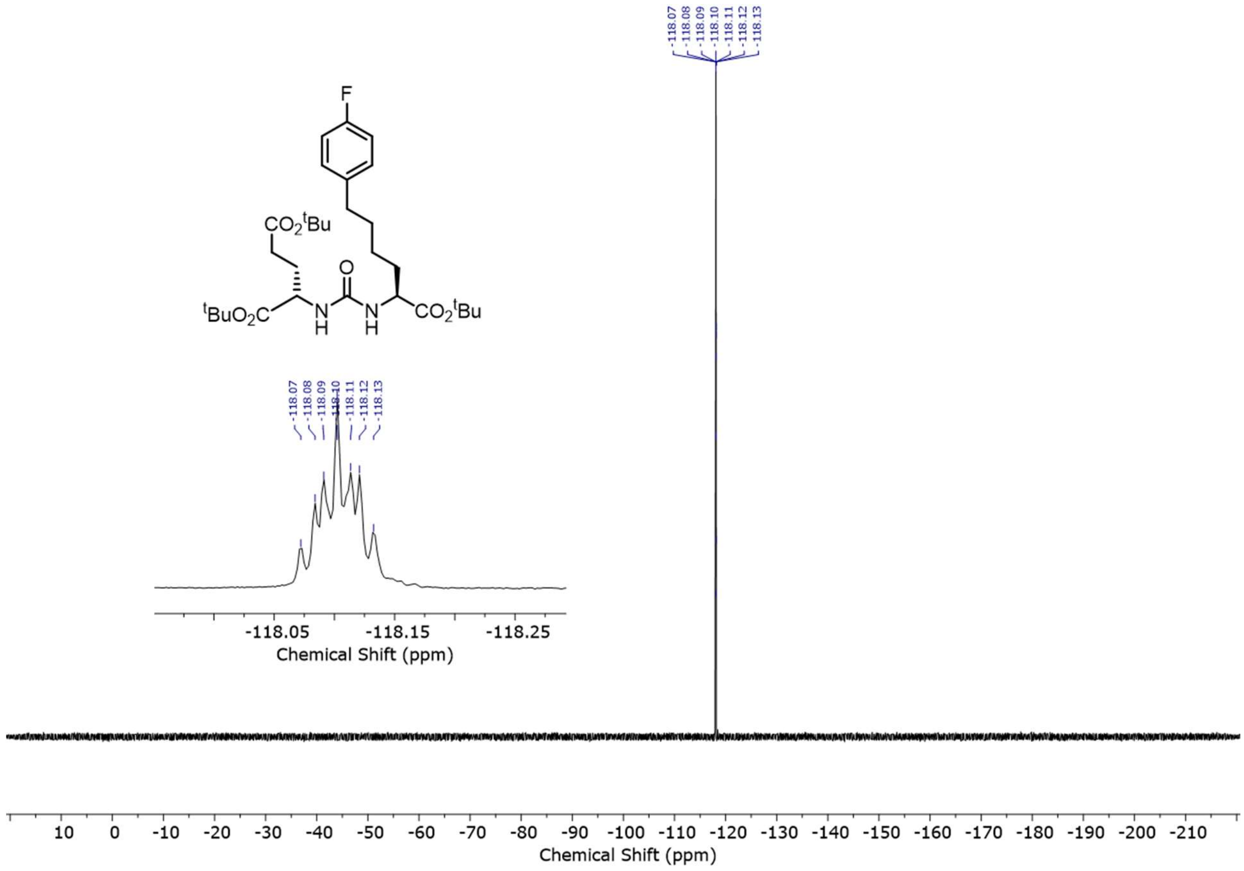

***N*-(4-Fluorophenethyl)-4,6-dimethoxypyrimidin-2-amine (25) ^{1}H NMR (400 MHz, $CDCl_3$ + 0.03% TMS)**

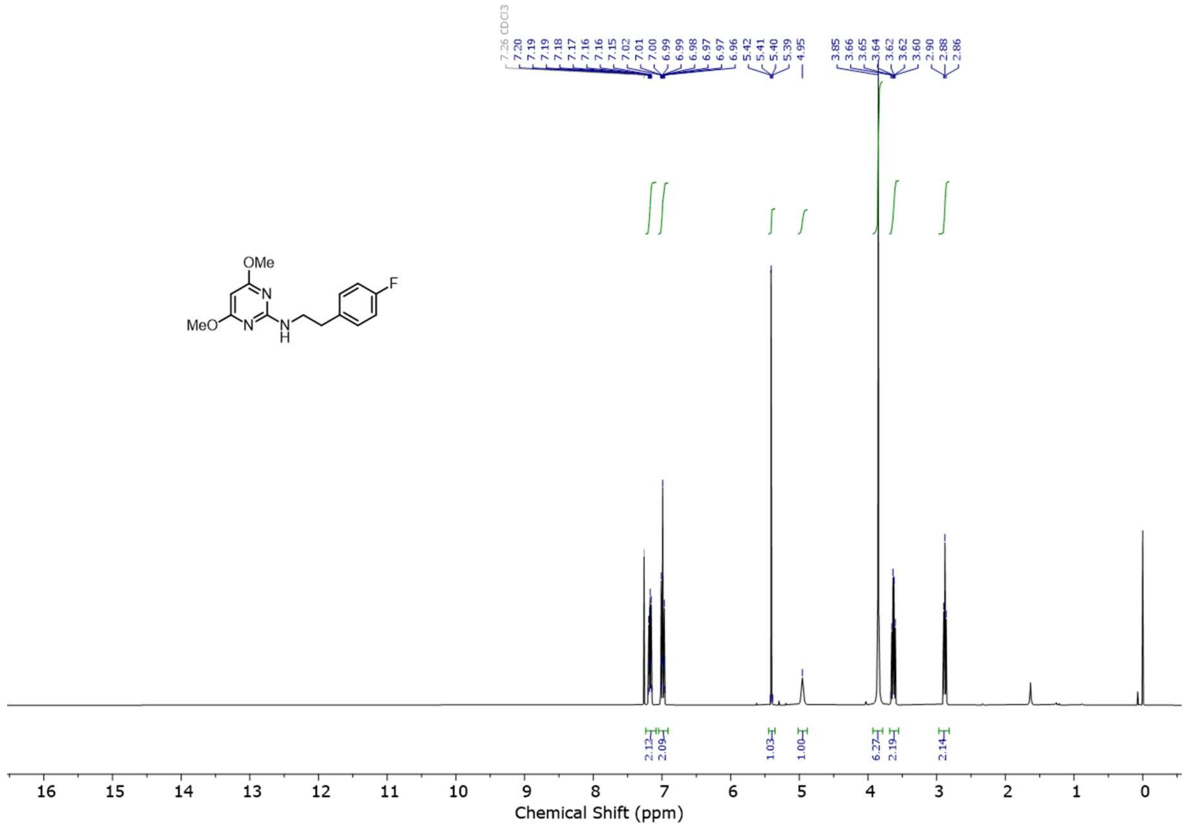

***N*-(4-Fluorophenethyl)-4,6-dimethoxypyrimidin-2-amine (25) ^{13}C NMR (101 MHz, $CDCl_3$)**

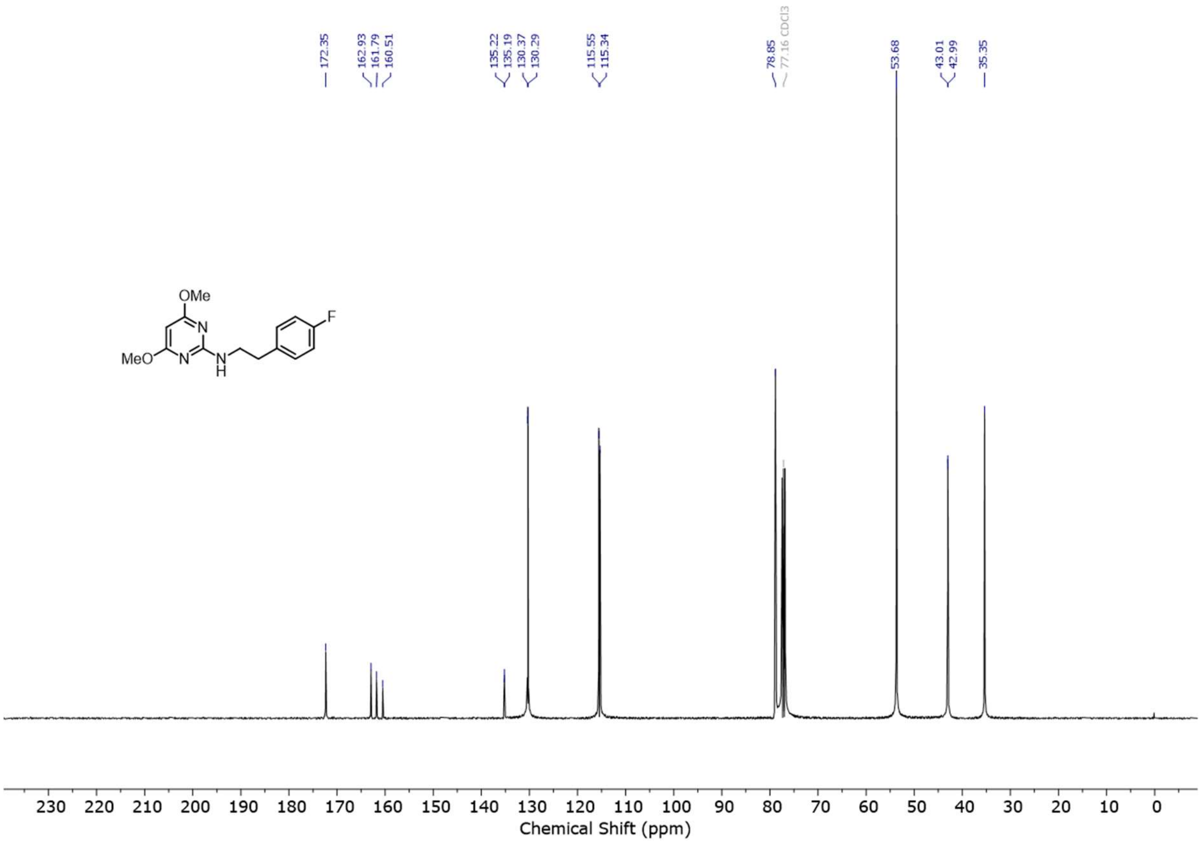

***N*-(4-Fluorophenethyl)-4,6-dimethoxypyrimidin-2-amine (25) ^{19}F NMR (377 MHz, $CDCl_3$)**

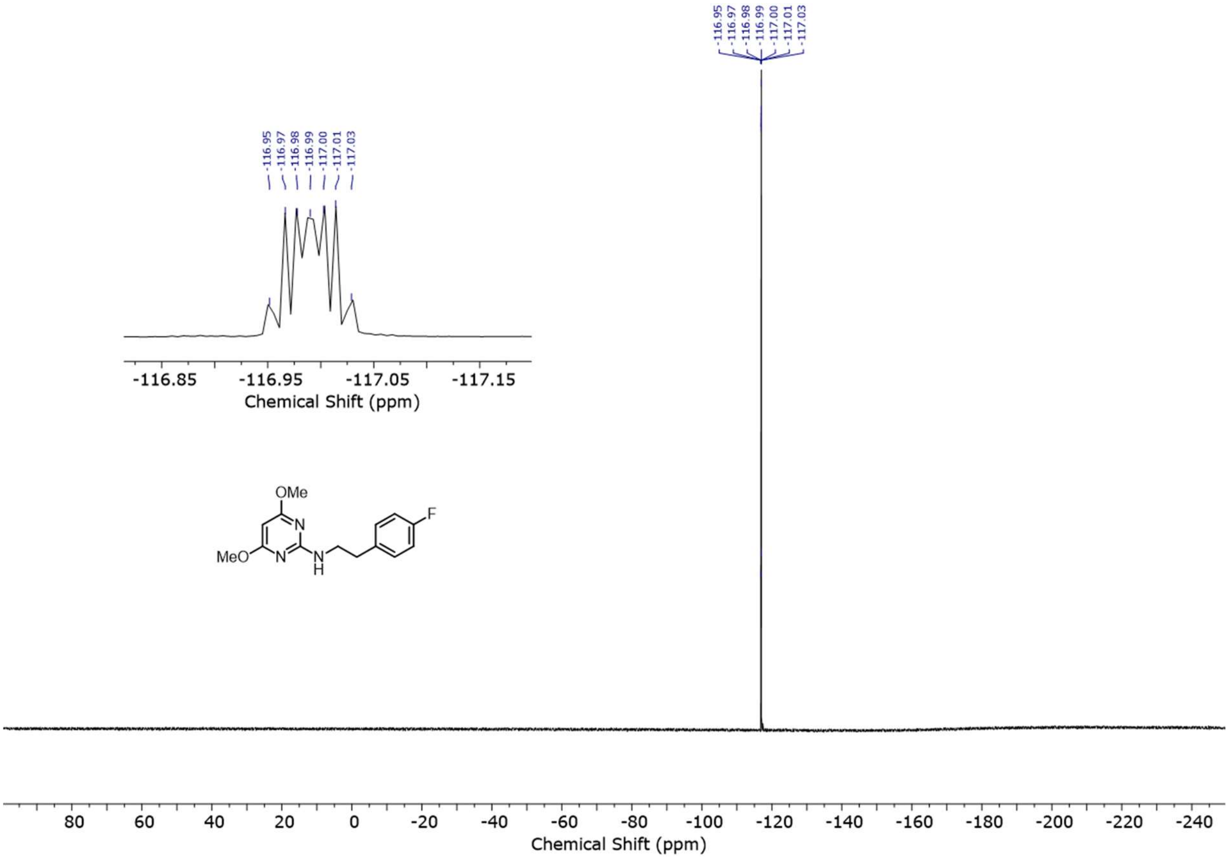

1-(2-(Benzhydryloxy)ethyl)-4-(3-(4-fluorophenyl)propyl)piperazine (26) ^{1}H NMR (500 MHz, $CDCl_3$)

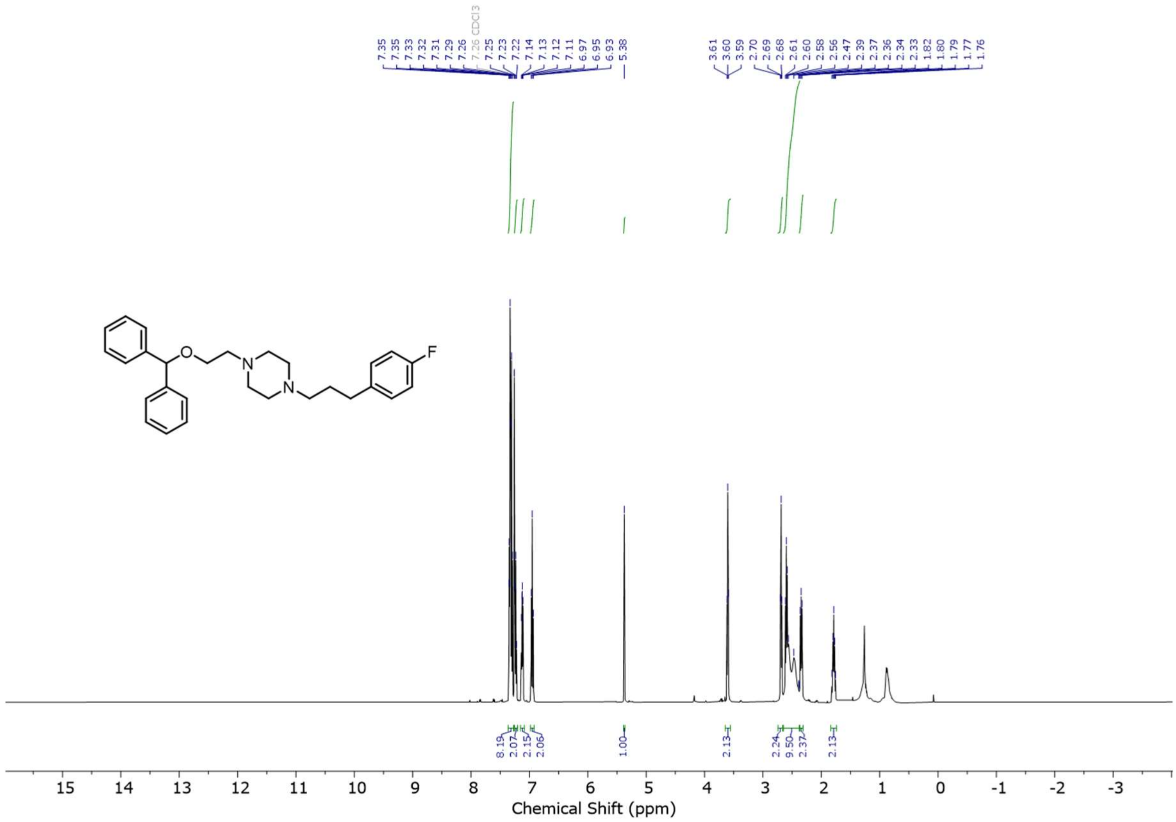

1-(2-(Benzhydryloxy)ethyl)-4-(3-(4-fluorophenyl)propyl)piperazine (26) ^{13}C NMR (126 MHz, $CDCl_3$)

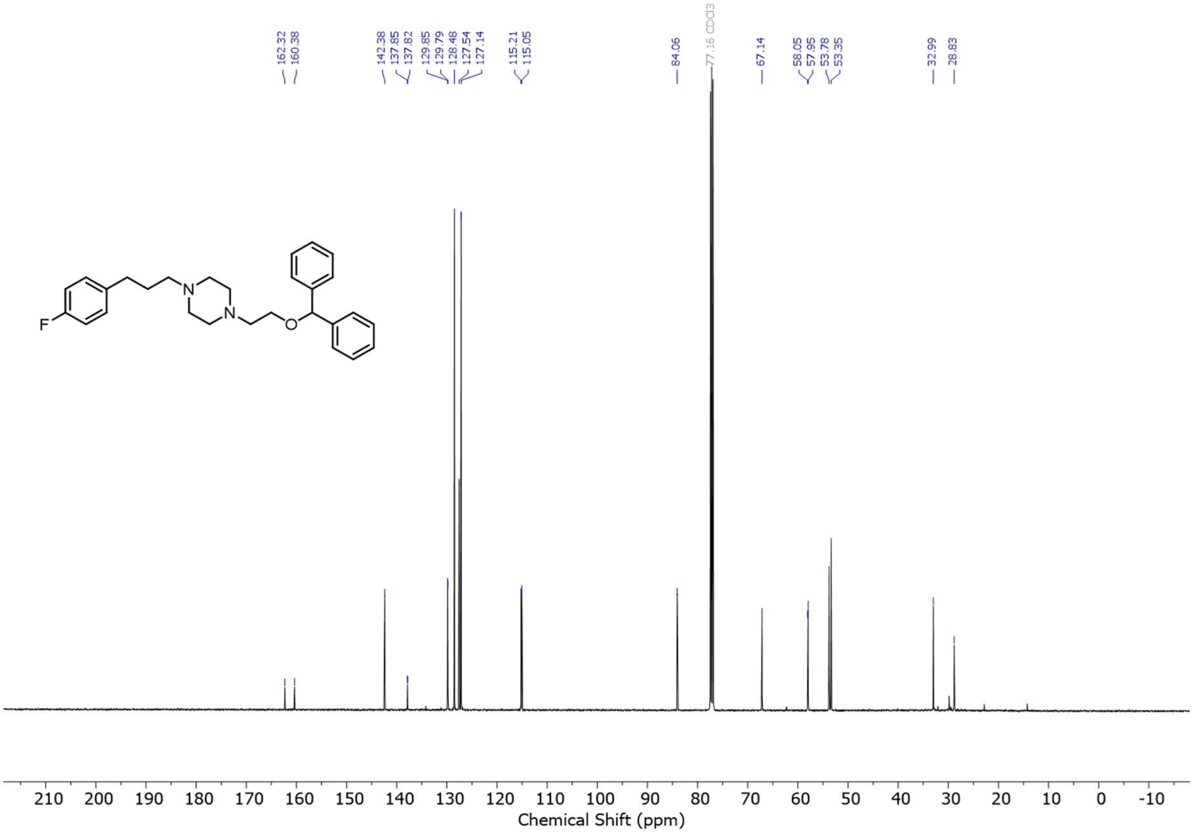

1-(2-(Benzhydryloxy)ethyl)-4-(3-(4-fluorophenyl)propyl)piperazine (26) ^{19}F NMR (470 MHz, $CDCl_3$)

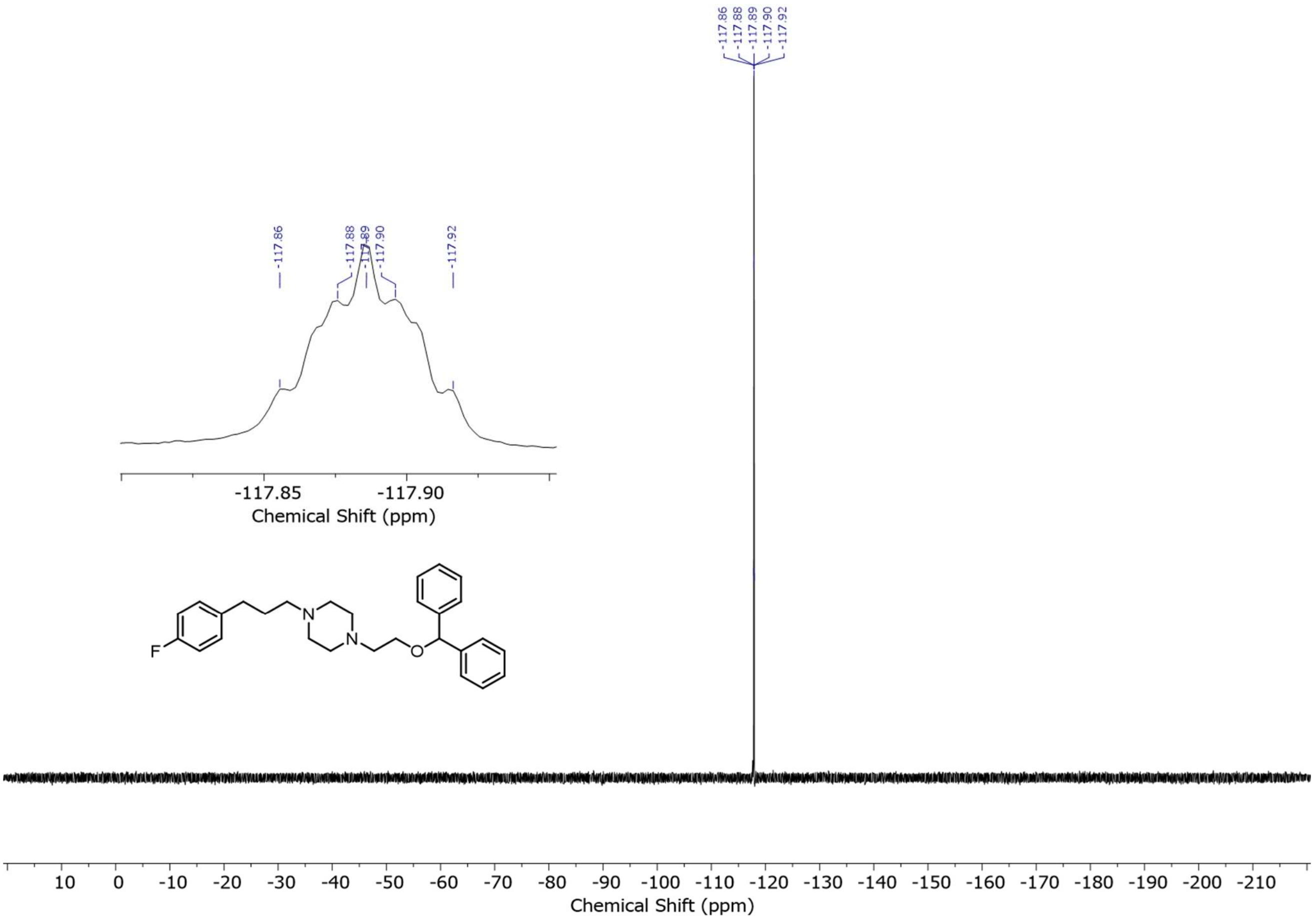

7-(4-(4-Fluorophenethyl)piperazine-1-carbonyl)-1*H*-indole-3-carbonitrile (27) ^{1}H NMR (400 MHz, CDCl$_3$)

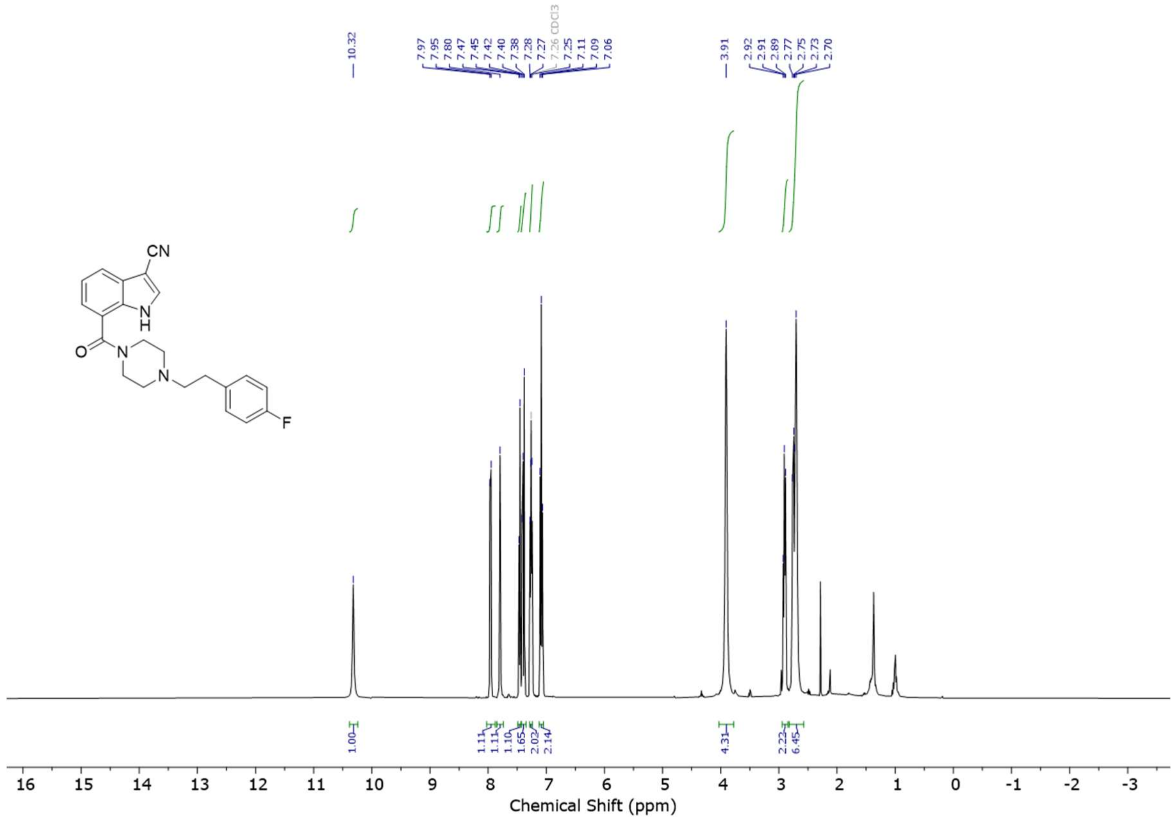

7-(4-(4-Fluorophenethyl)piperazine-1-carbonyl)-1*H*-indole-3-carbonitrile (27) ^{13}C NMR (126 MHz, CDCl$_3$)

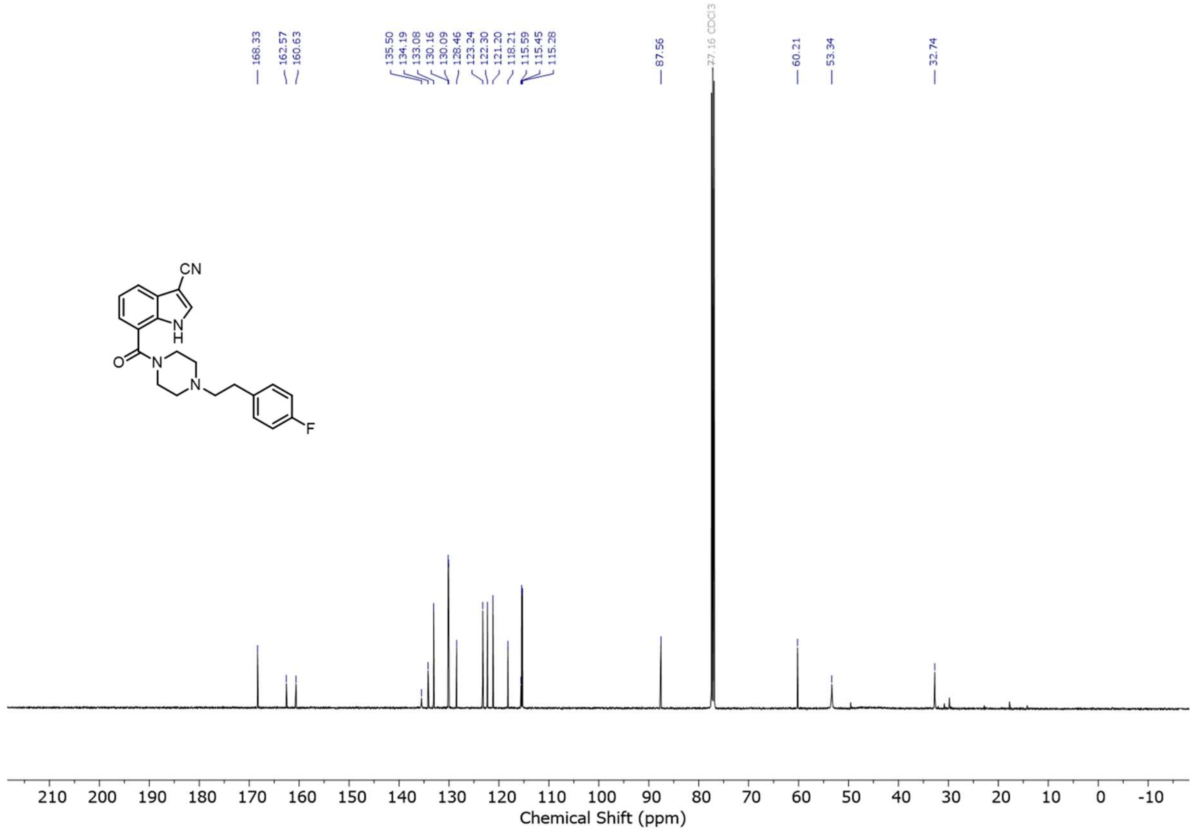

7-(4-(4-Fluorophenethyl)piperazine-1-carbonyl)-1*H*-indole-3-carbonitrile (27) $\{^{1}H\}^{19}F$ NMR (377 MHz, $CDCl_3$)

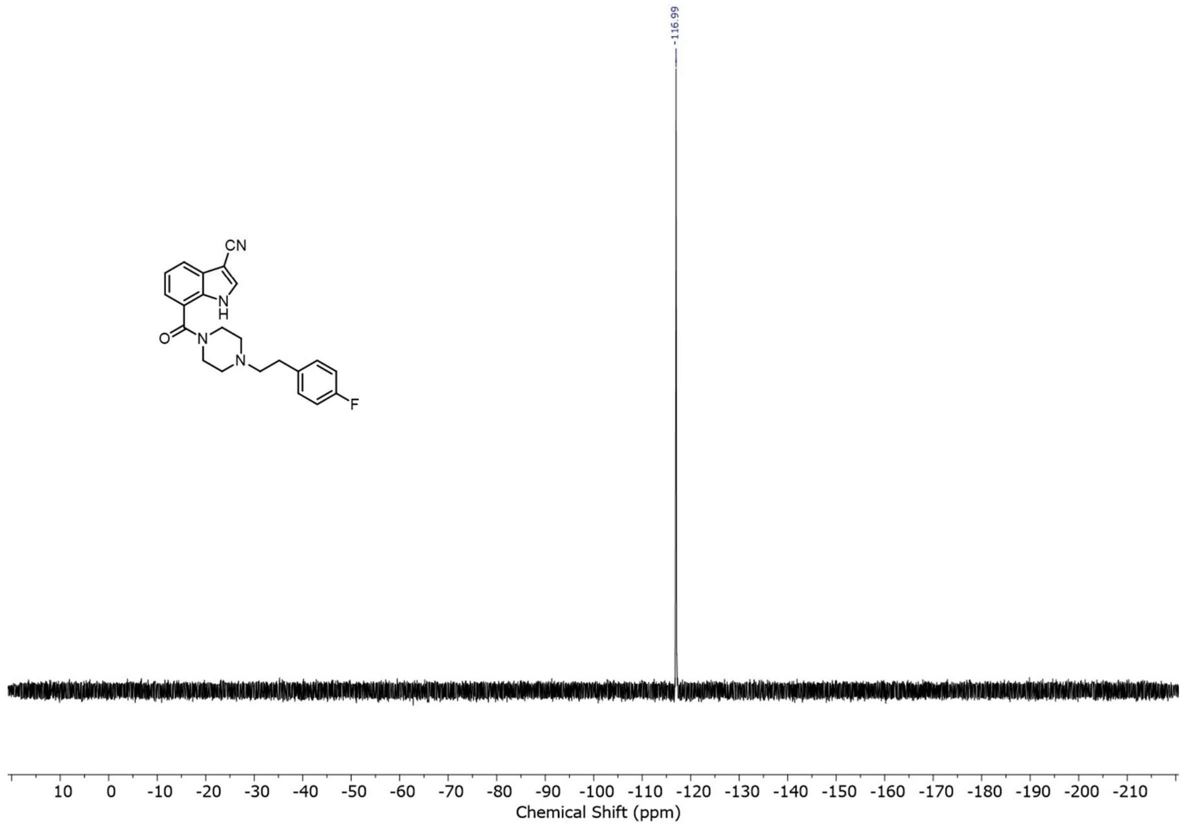

***N*-(3-(4-Fluorophenyl)propyl)-7-hydroxy-2-oxo-*2H*-chromene-3-carboxamide (28) ^{1}H NMR (400 MHz, DMSO-*d*$_6$)**

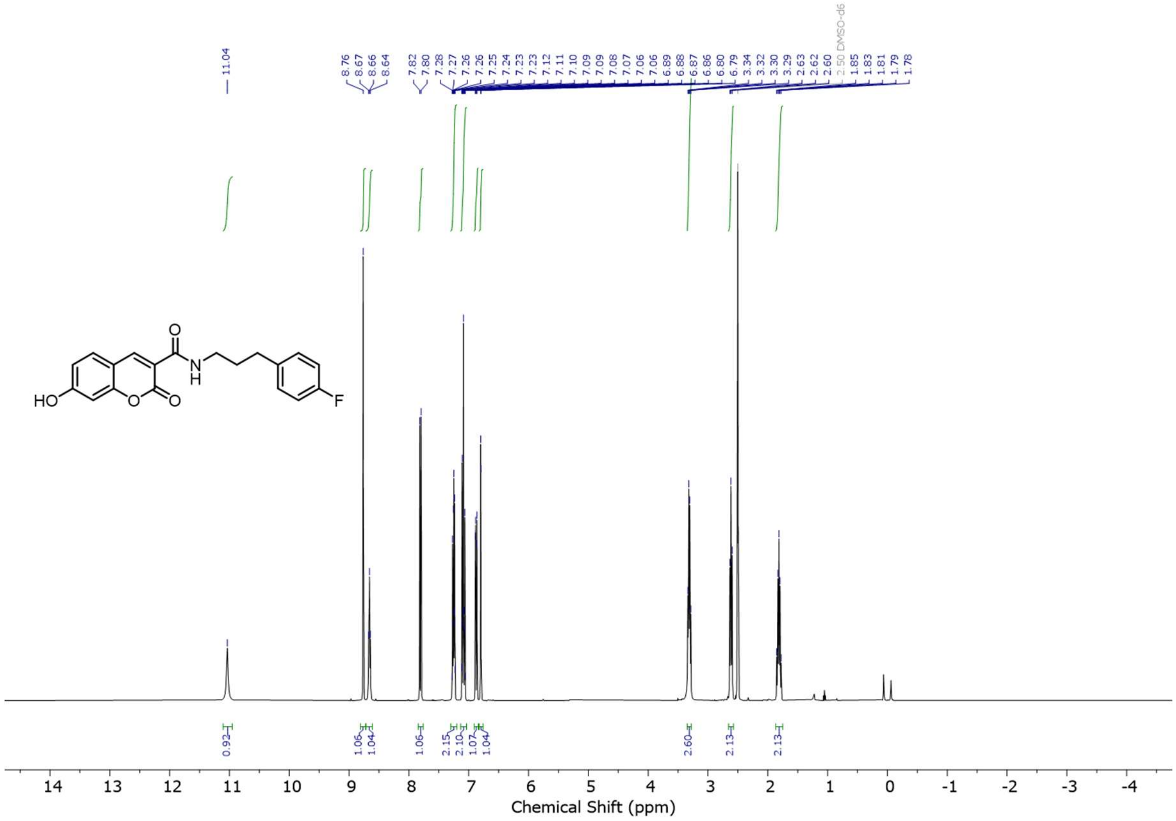

***N*-(3-(4-Fluorophenyl)propyl)-7-hydroxy-2-oxo-*2H*-chromene-3-carboxamide (28) ^{13}C NMR (101 MHz, DMSO-*d*$_6$)**

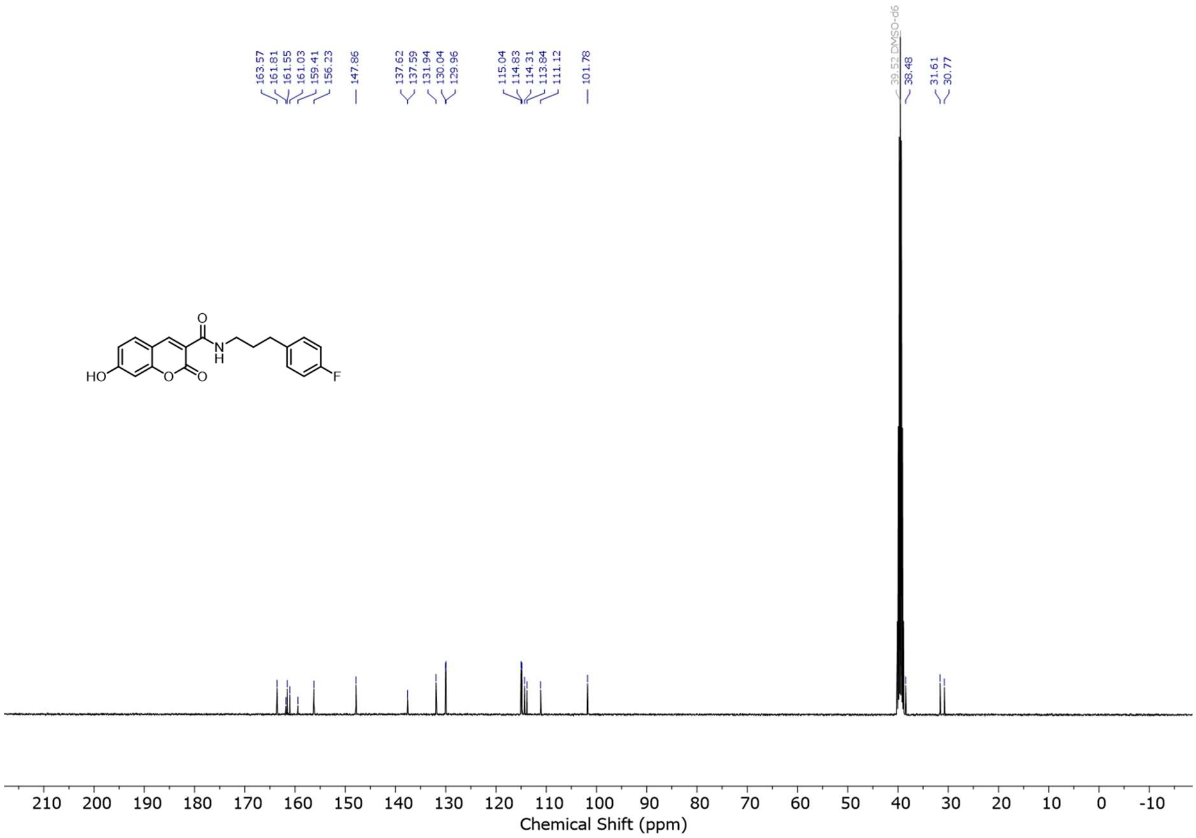

N-(3-(4-Fluorophenyl)propyl)-7-hydroxy-2-oxo-2H-chromene-3-carboxamide (28) ^{19}F NMR (376 MHz, DMSO-d_6)

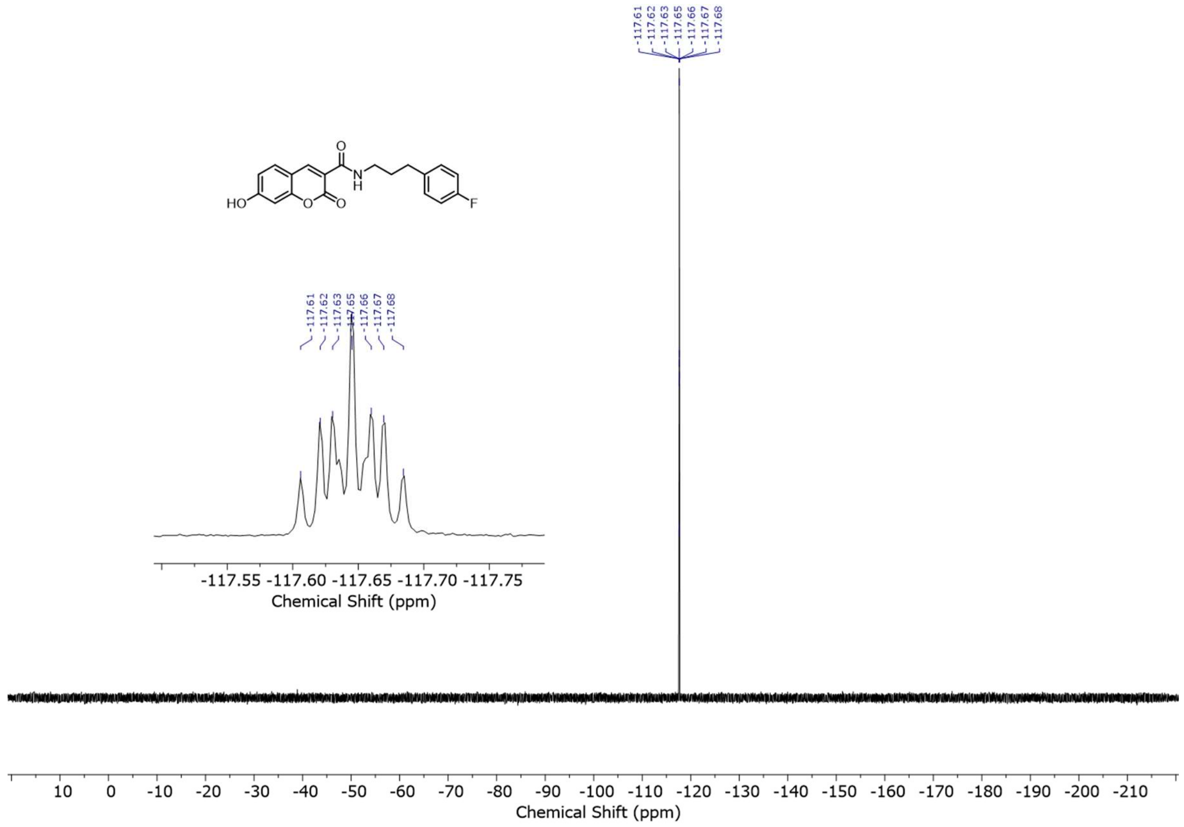

2-(4-(4-Fluorophenyl)piperidin-1-yl)pyrimidine (32) ^{1}H NMR (400 MHz, $CDCl_3$ + 0.03% TMS)

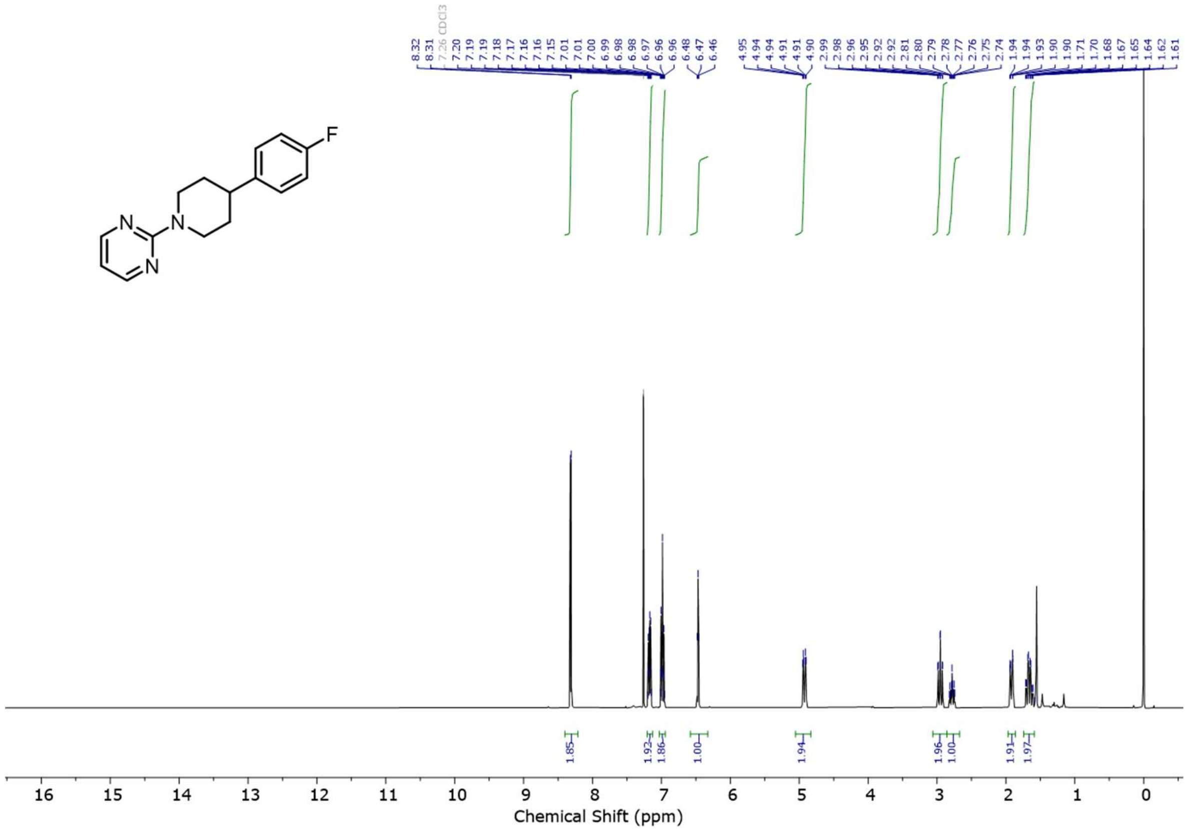

2-(4-(4-Fluorophenyl)piperidin-1-yl)pyrimidine (32) ^{13}C NMR (101 MHz, $CDCl_3$ + 0.03% TMS)

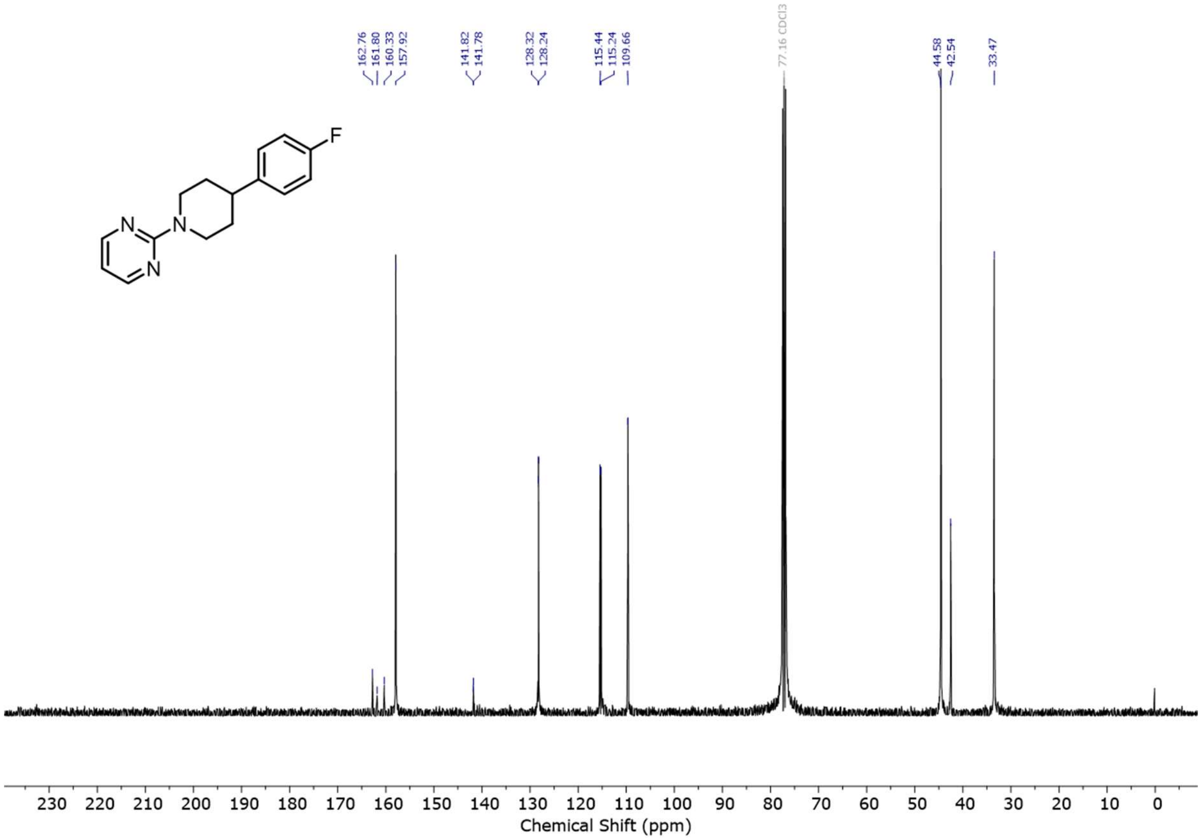

2-(4-(4-Fluorophenyl)piperidin-1-yl)pyrimidine (32) ^{19}F NMR (377 MHz, $CDCl_3$ + 0.03% TMS)

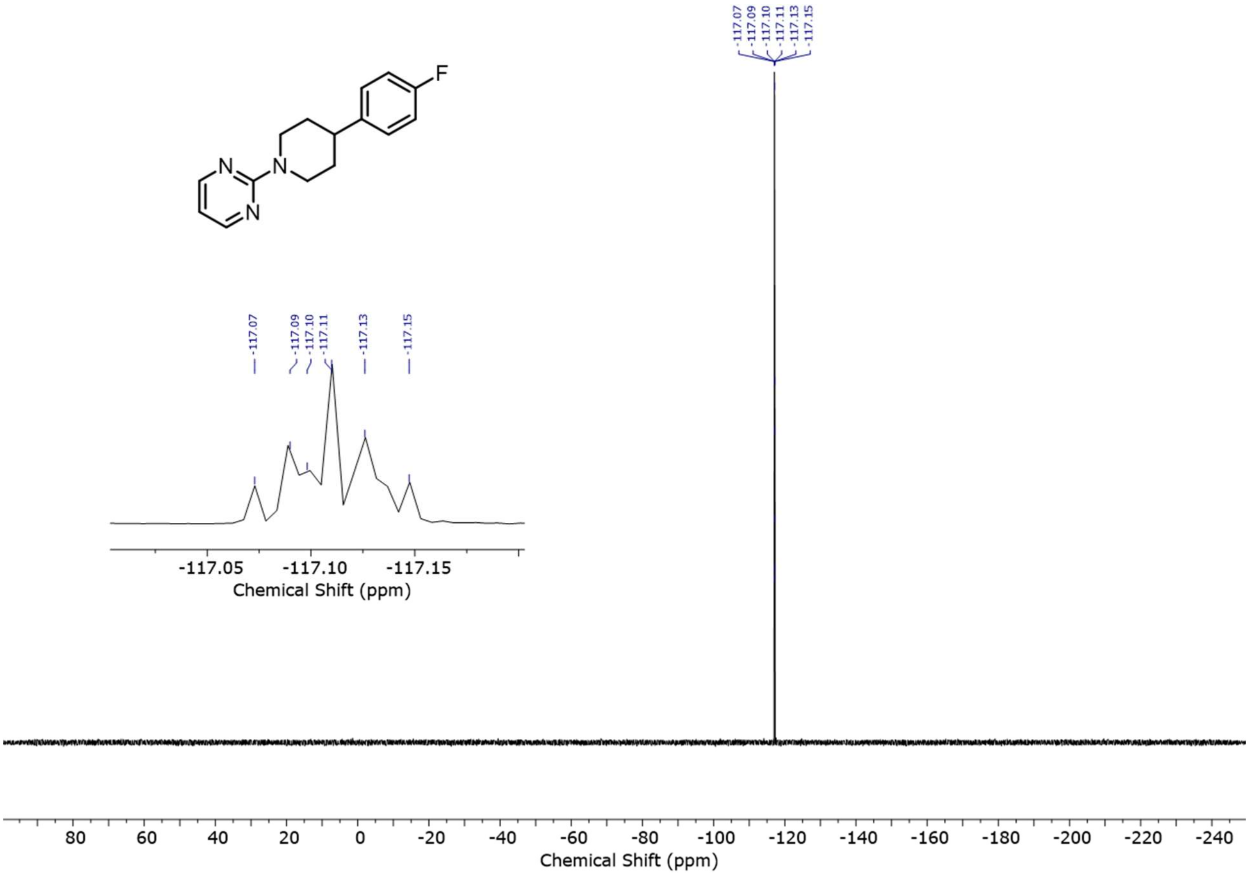

1-(3-(5-(3-Chloro-4-methoxyphenyl)oxazole-4-carboxamido)propyl)-2,4,6-triphenylpyridin-1-ium Tetrafluoroborate (33) ^{1}H NMR (400 MHz, CDCl$_3$)

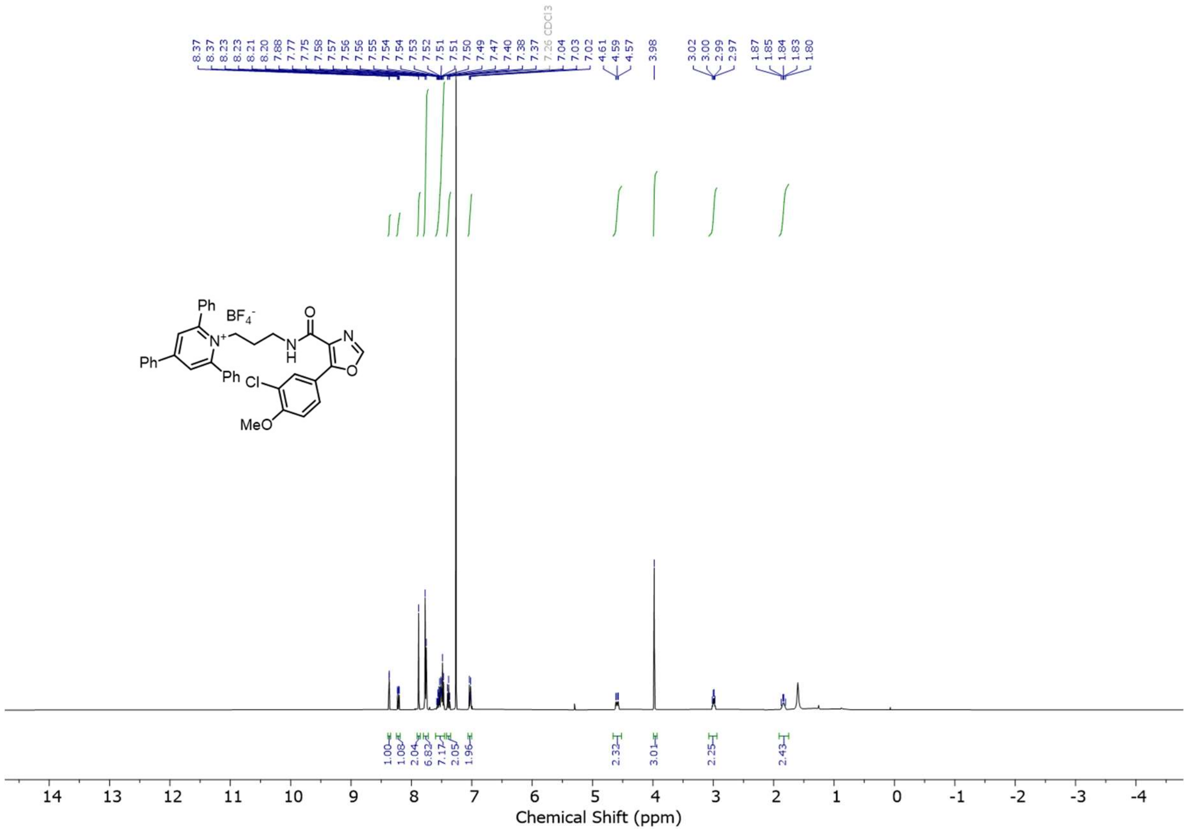

1-(3-(5-(3-Chloro-4-methoxyphenyl)oxazole-4-carboxamido)propyl)-2,4,6-triphenylpyridin-1-ium Tetrafluoroborate (33) ^{13}C NMR (101 MHz, CDCl$_3$)

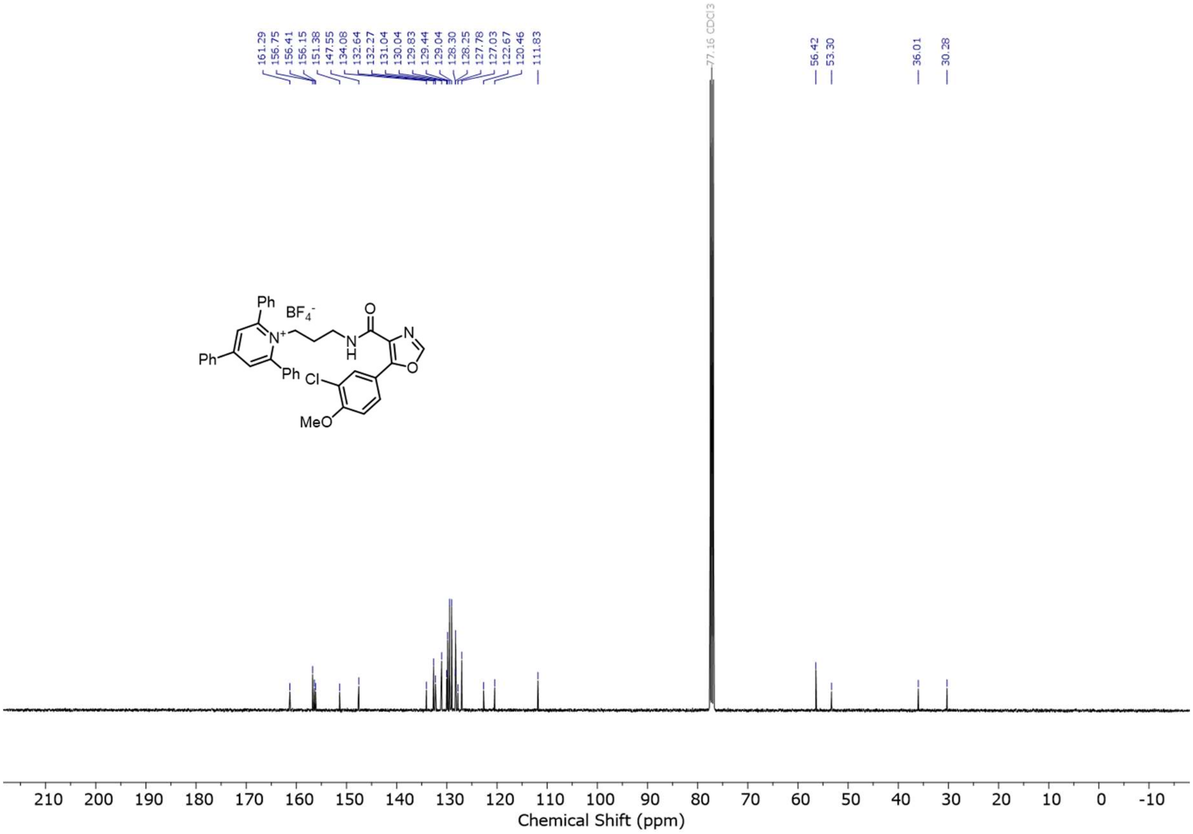

1-(3-(5-(3-Chloro-4-methoxyphenyl)oxazole-4-carboxamido)propyl)-2,4,6-triphenylpyridin-1-ium Tetrafluoroborate (33) ^{19}F NMR (376 MHz, $CDCl_3$)

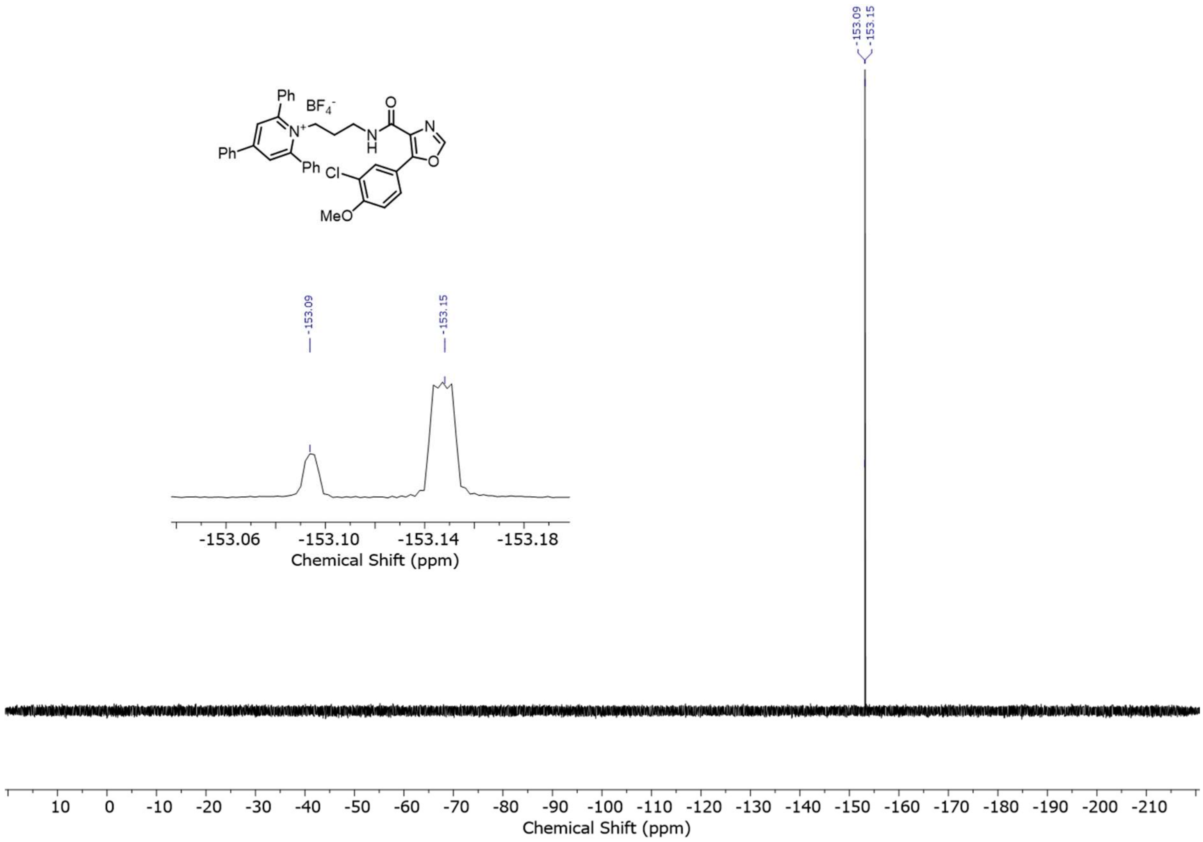

5-(3-Chloro-4-methoxyphenyl)-*N*-(3-(4-(trifluoromethyl)phenyl)propyl)oxazole-4-carboxamide (39) ^{1}H NMR (400 MHz, $CDCl_3$)

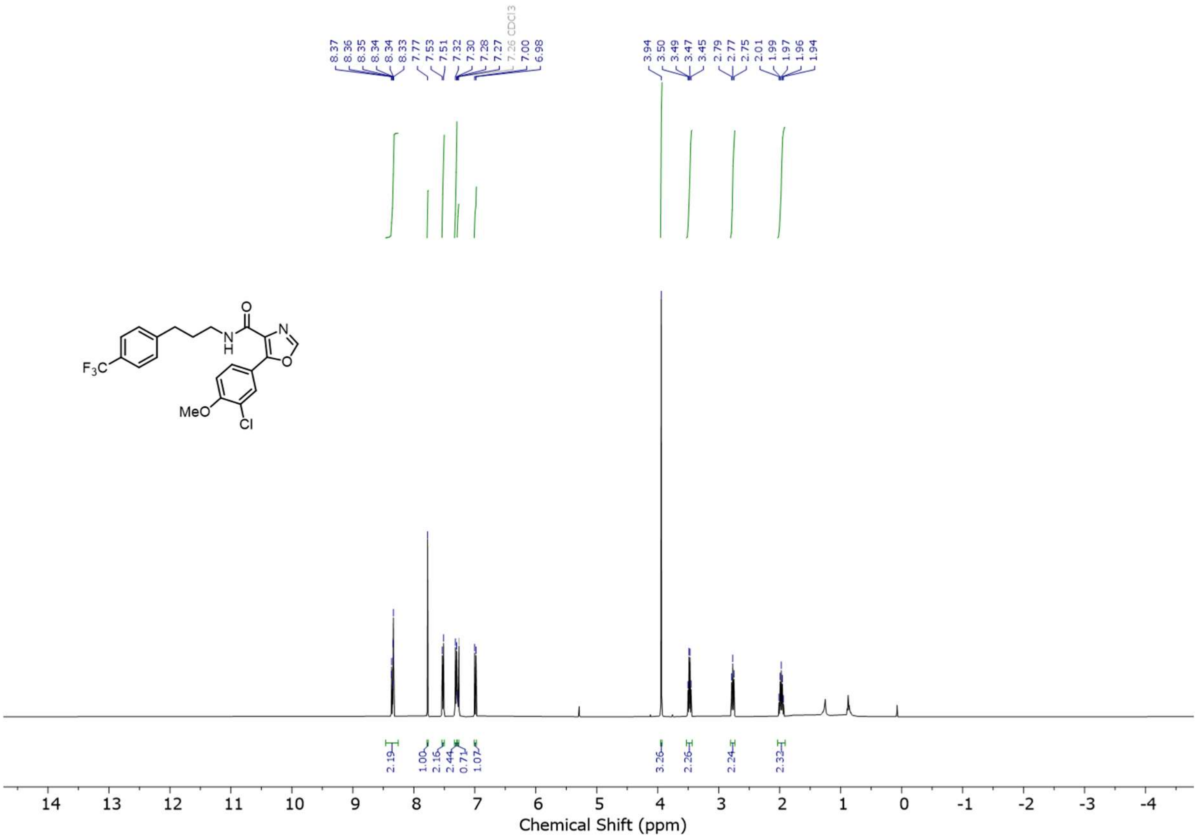

5-(3-Chloro-4-methoxyphenyl)-*N*-(3-(4-(trifluoromethyl)phenyl)propyl)oxazole-4-carboxamide (39) ^{13}C NMR (101 MHz, $CDCl_3$)

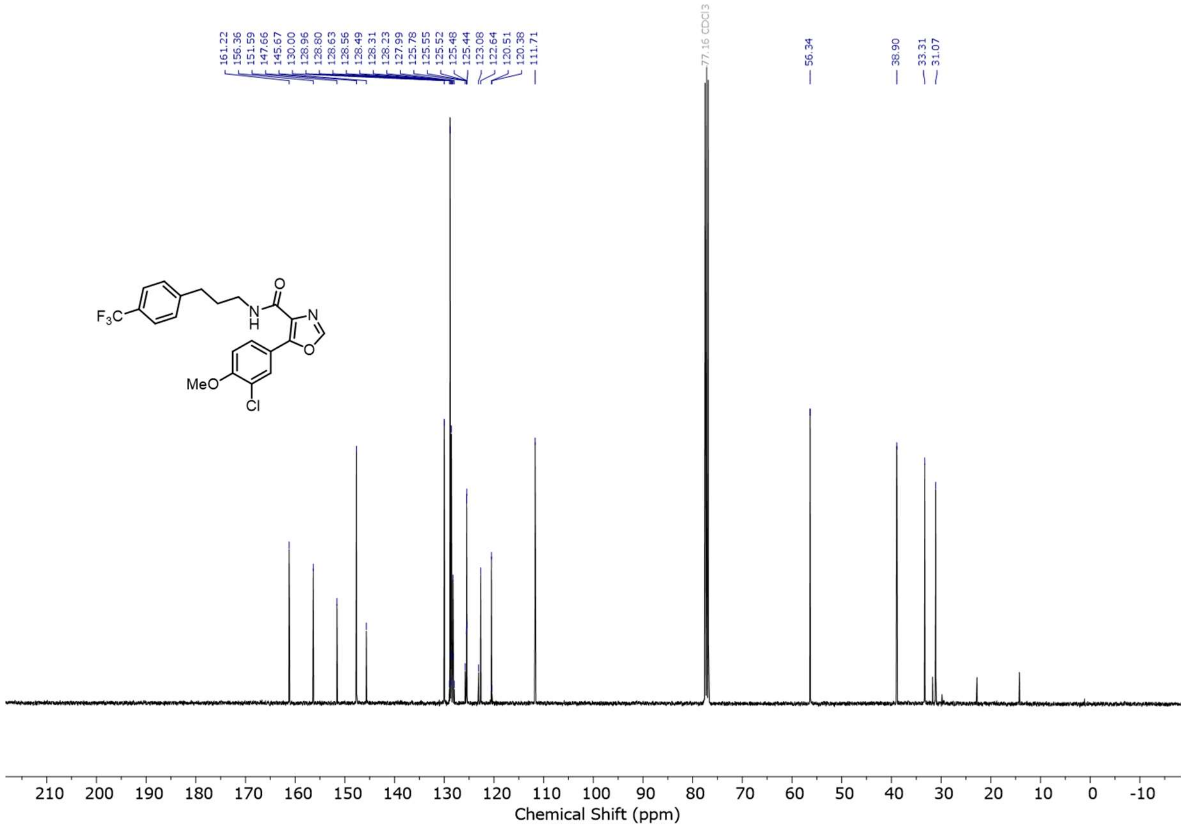

5-(3-Chloro-4-methoxyphenyl)-*N*-(3-(4-(trifluoromethyl)phenyl)propyl)oxazole-4-carboxamide (39) ^{19}F NMR (376 MHz, $CDCl_3$)

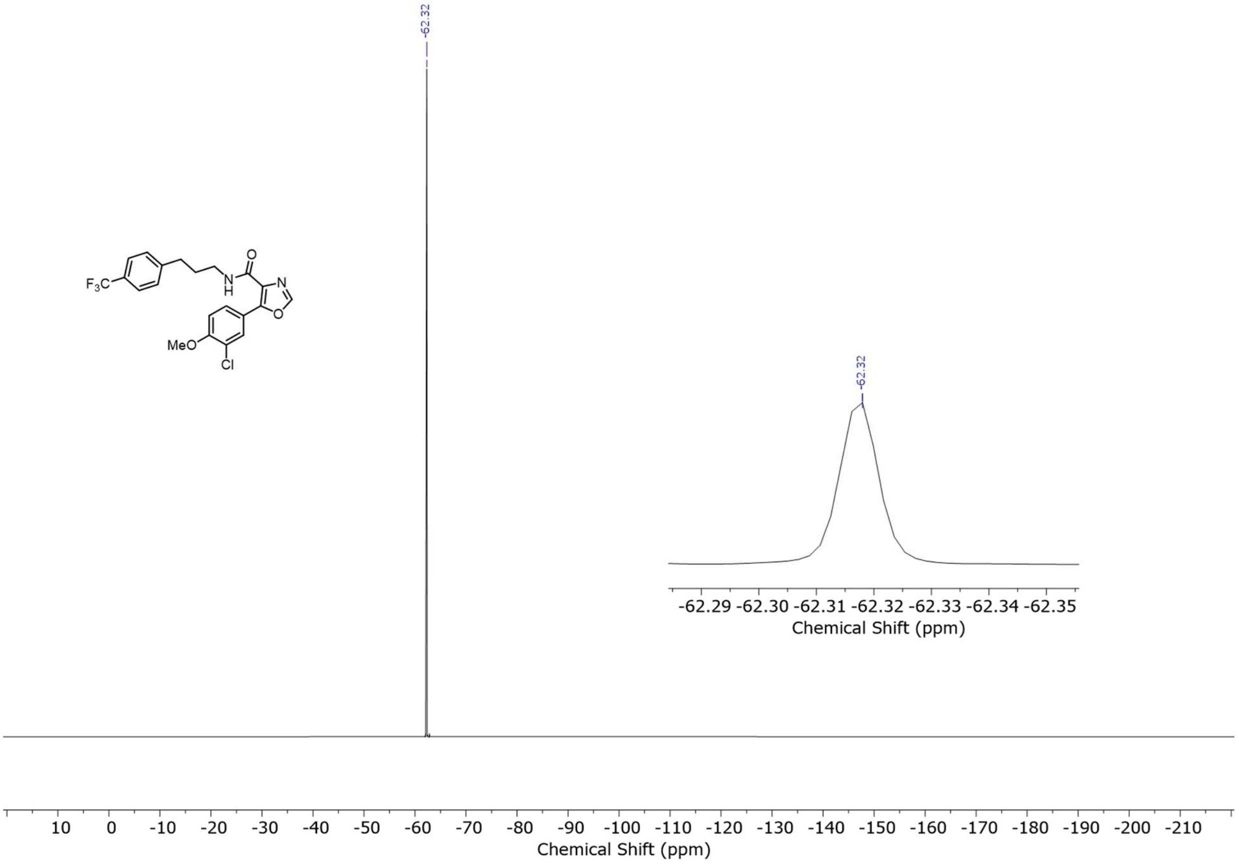

5-(3-Chloro-4-methoxyphenyl)-*N*-(3-(4-(2-fluoroethoxy)phenyl)propyl)oxazole-4-carboxamide (40) ^{1}H NMR (400 MHz, $CDCl_3$)

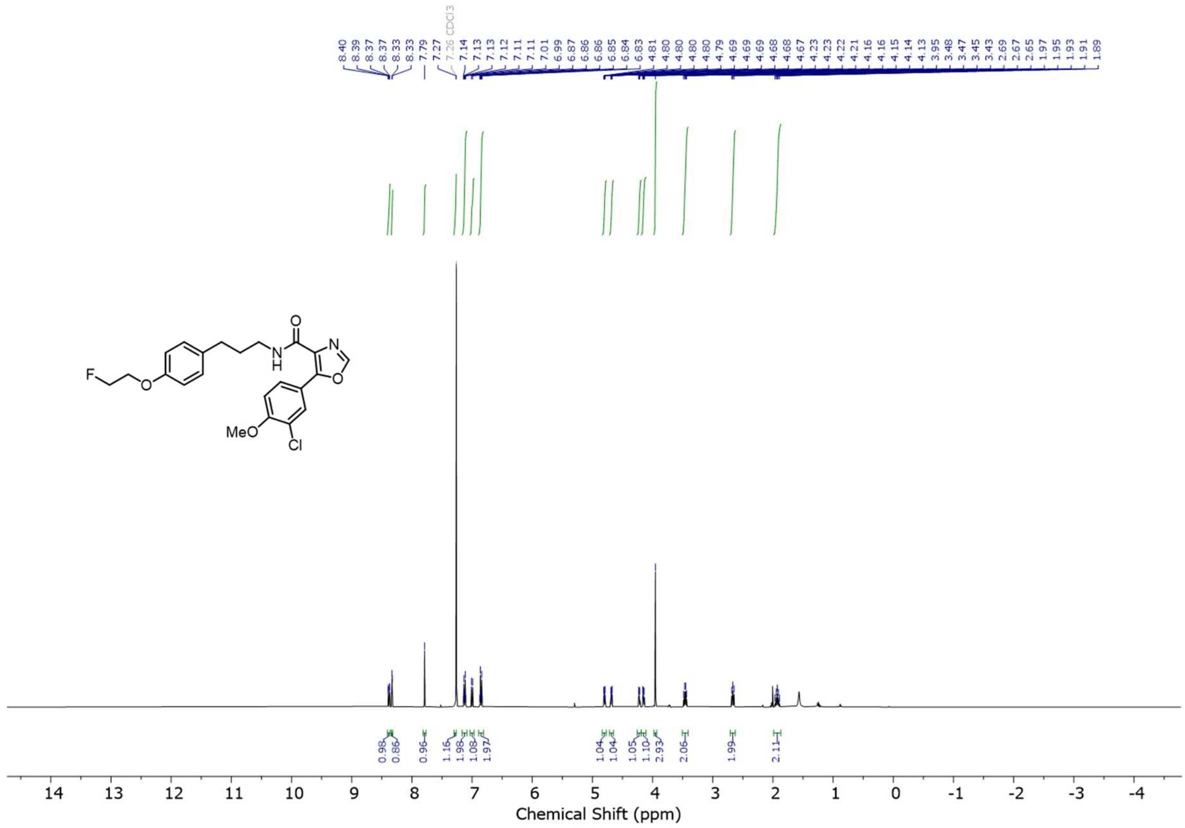

5-(3-Chloro-4-methoxyphenyl)-*N*-(3-(4-(2-fluoroethoxy)phenyl)propyl)oxazole-4-carboxamide (40) ^{13}C NMR (101 MHz, $CDCl_3$)

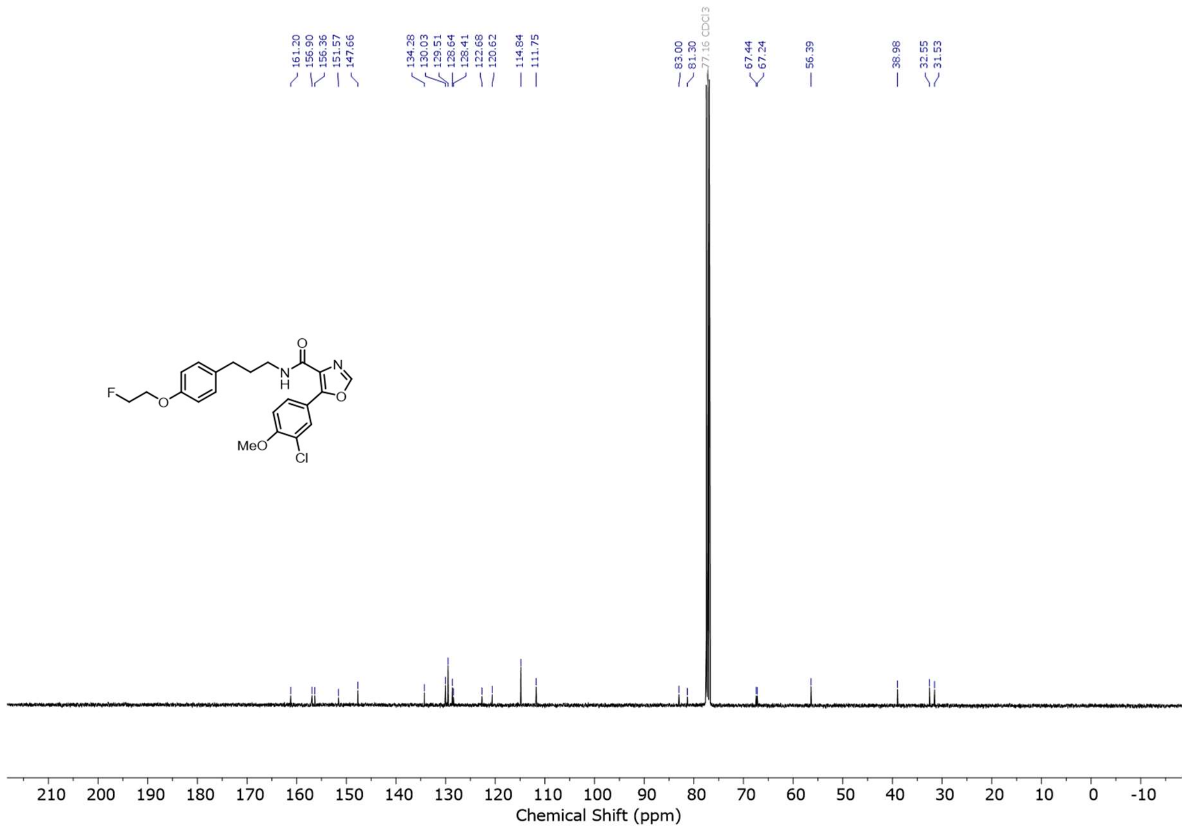

5-(3-Chloro-4-methoxyphenyl)-*N*-(3-(4-(2-fluoroethoxy)phenyl)propyl)oxazole-4-carboxamide (40) ^{19}F NMR (377 MHz, $CDCl_3$)

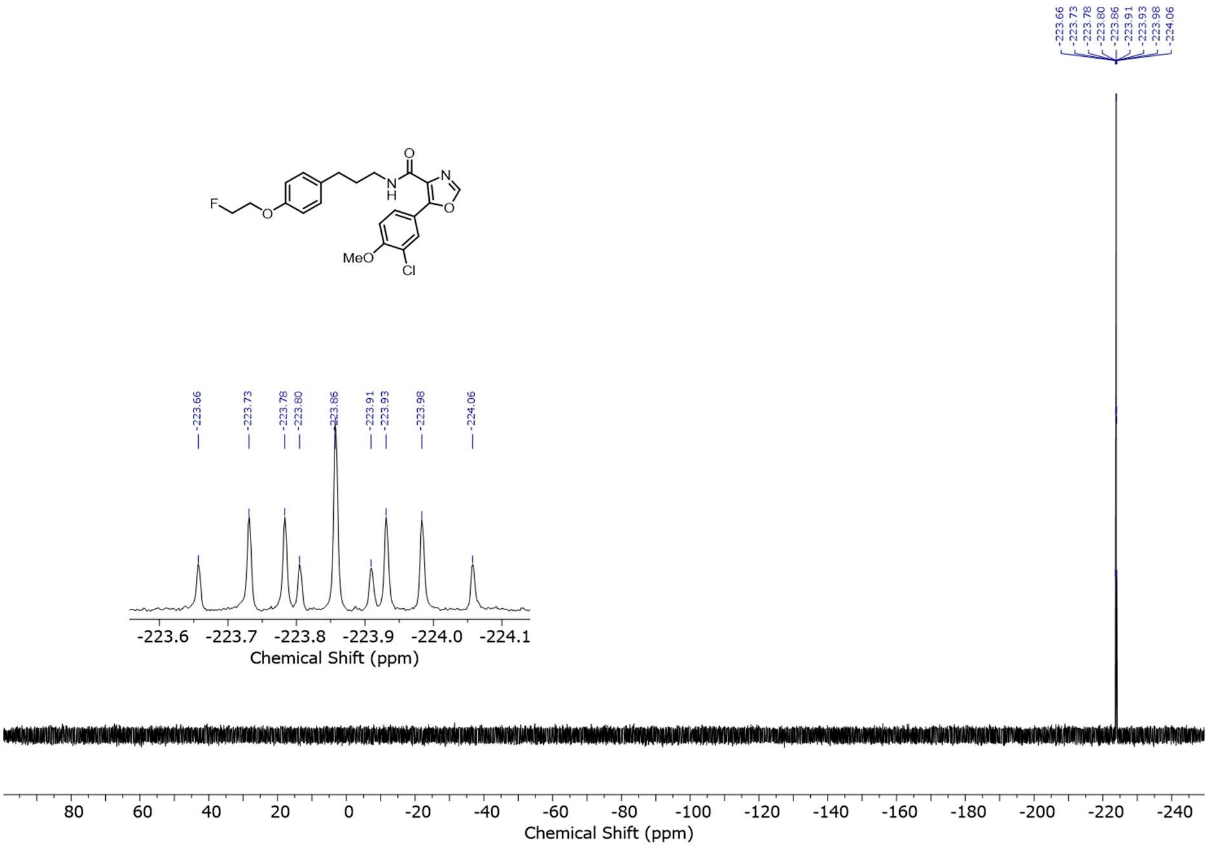

5-(3-Chloro-4-methoxyphenyl)-*N*-(3-(4-fluorophenyl)propyl)oxazole-4-carboxamide (41) ^{1}H NMR (500 MHz, $CDCl_3$)

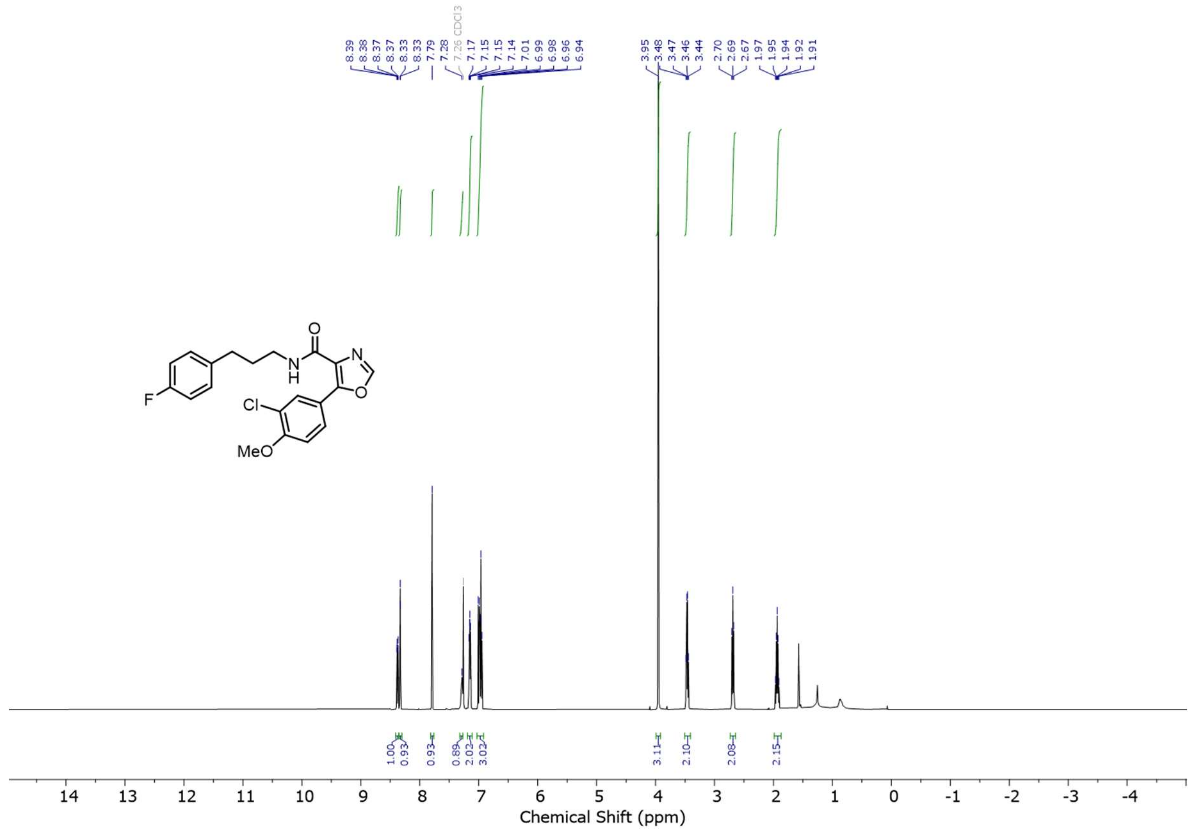

5-(3-Chloro-4-methoxyphenyl)-*N*-(3-(4-fluorophenyl)propyl)oxazole-4-carboxamide (41) ^{13}C NMR (126 MHz, $CDCl_3$)

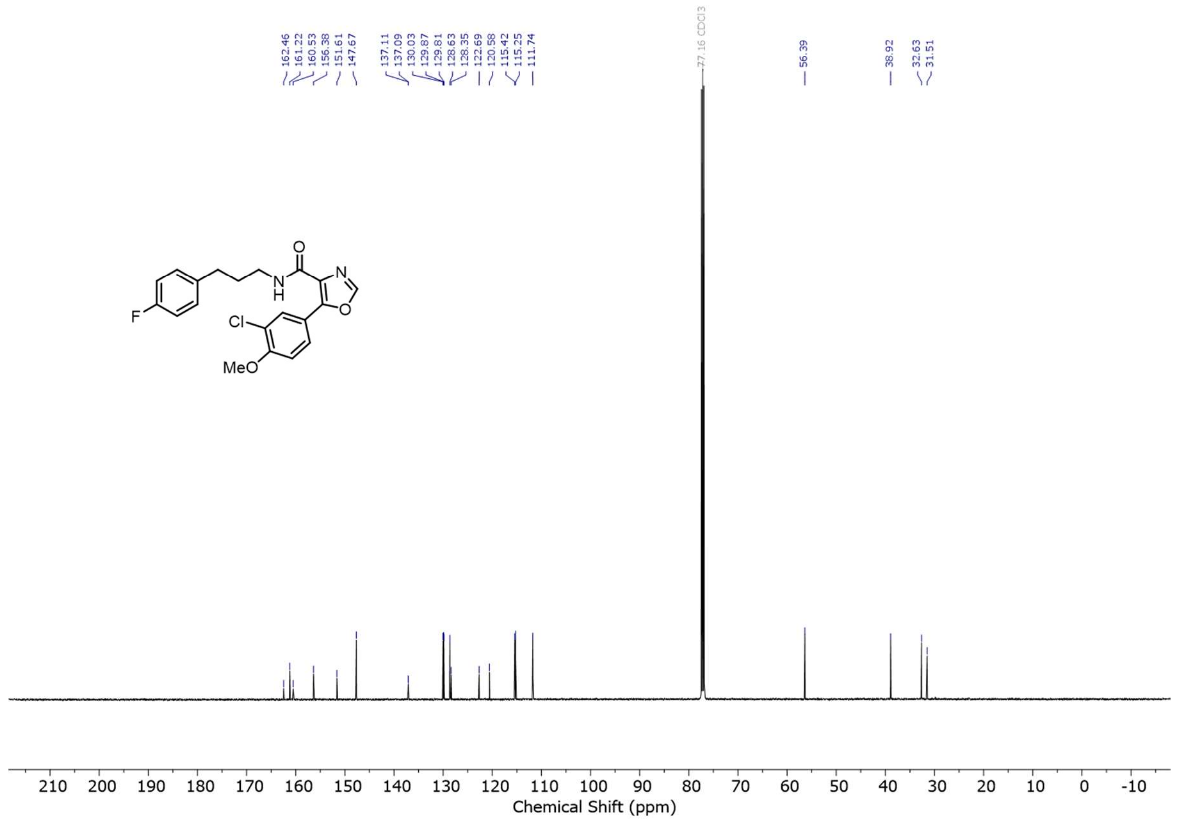

5-(3-Chloro-4-methoxyphenyl)-*N*-(3-(4-fluorophenyl)propyl)oxazole-4-carboxamide (41) ^{19}F NMR (471 MHz, $CDCl_3$)

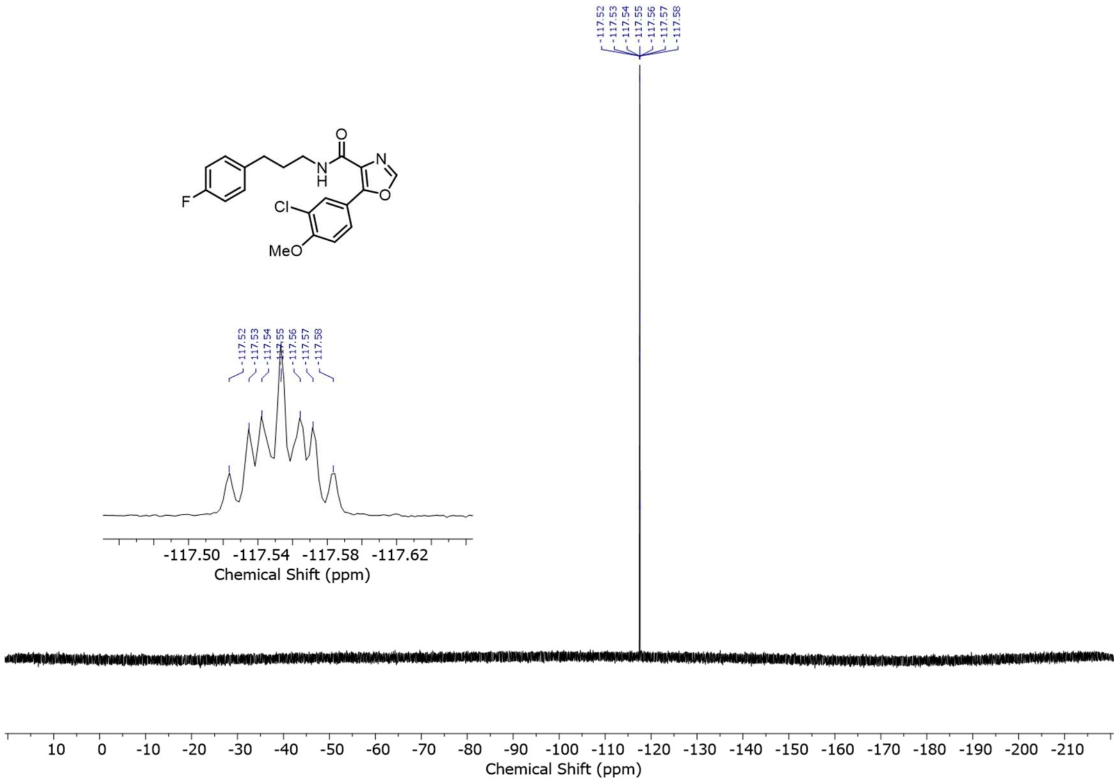

5-(3-Chloro-4-methoxyphenyl)-*N*-(3-(2-fluorophenyl)propyl)oxazole-4-carboxamide (42) ^{1}H NMR (400 MHz, $CDCl_3$)

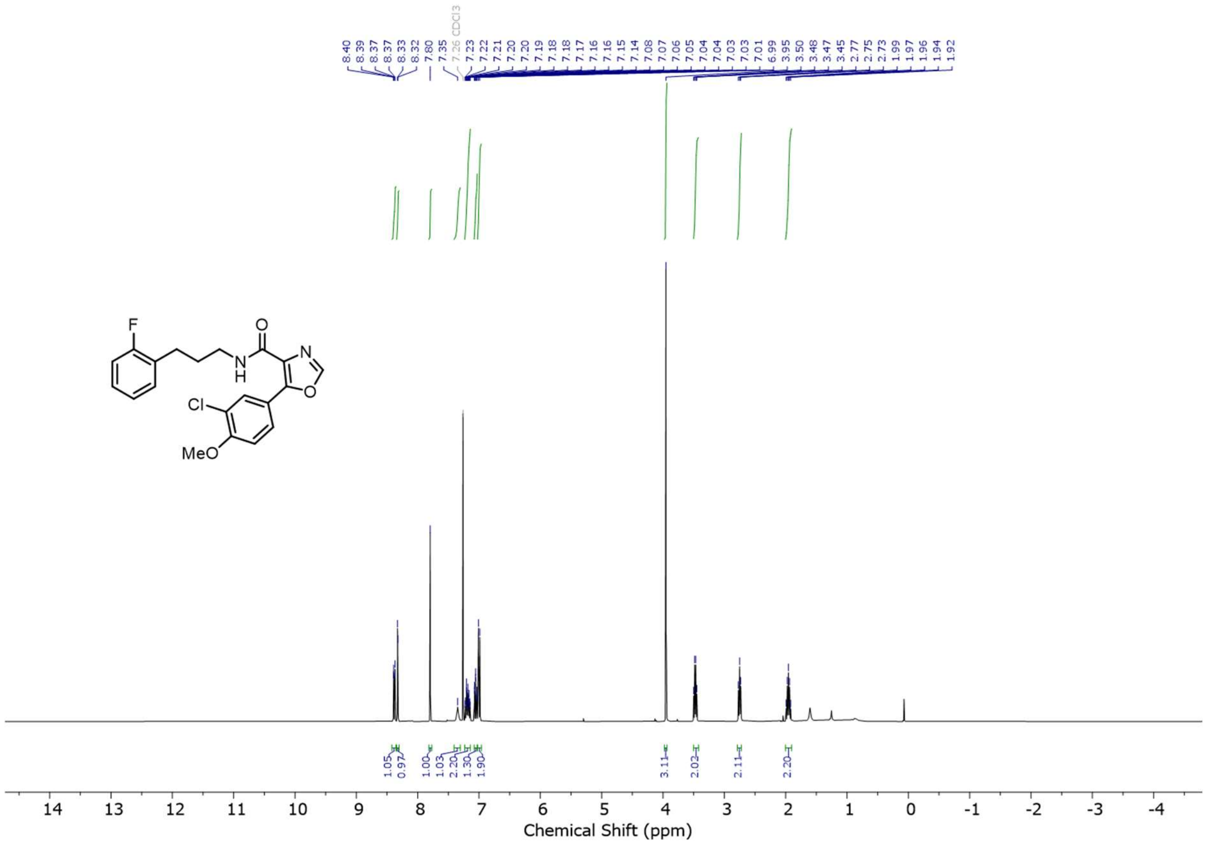

5-(3-Chloro-4-methoxyphenyl)-*N*-(3-(2-fluorophenyl)propyl)oxazole-4-carboxamide (42) ^{13}C NMR (101 MHz, $CDCl_3$)

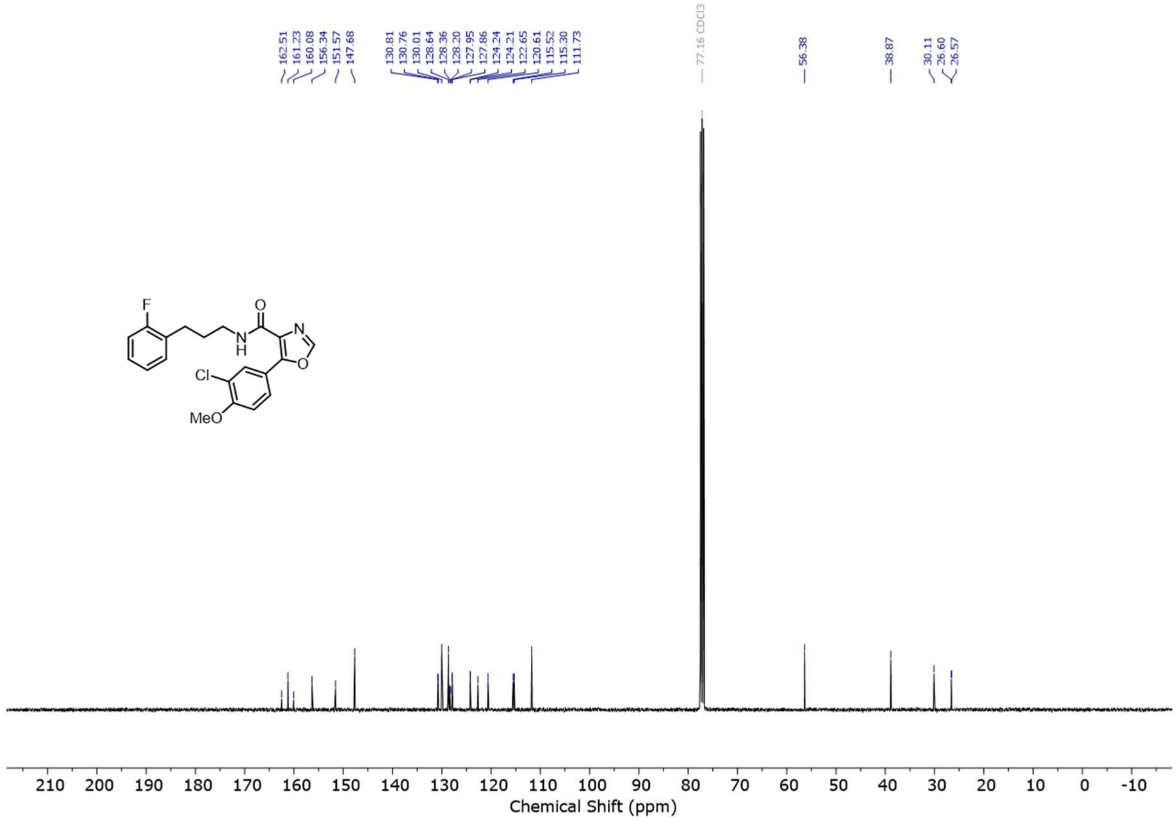

5-(3-Chloro-4-methoxyphenyl)-*N*-(3-(2-fluorophenyl)propyl)oxazole-4-carboxamide (42) ^{19}F NMR (376 MHz, $CDCl_3$)

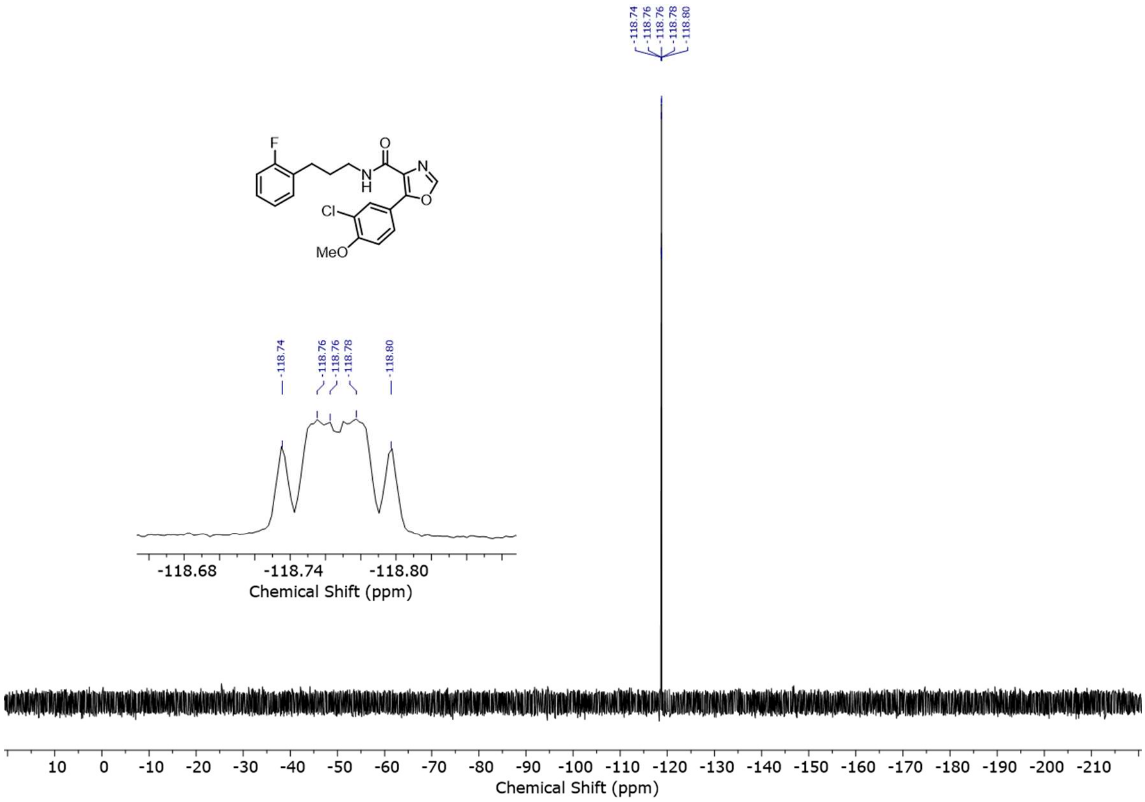

5-(3-Chloro-4-methoxyphenyl)-*N*-(3-(3-chloro-5-fluorophenyl)propyl)oxazole-4-carboxamide (43) ^{1}H NMR (500 MHz, $CDCl_3$)

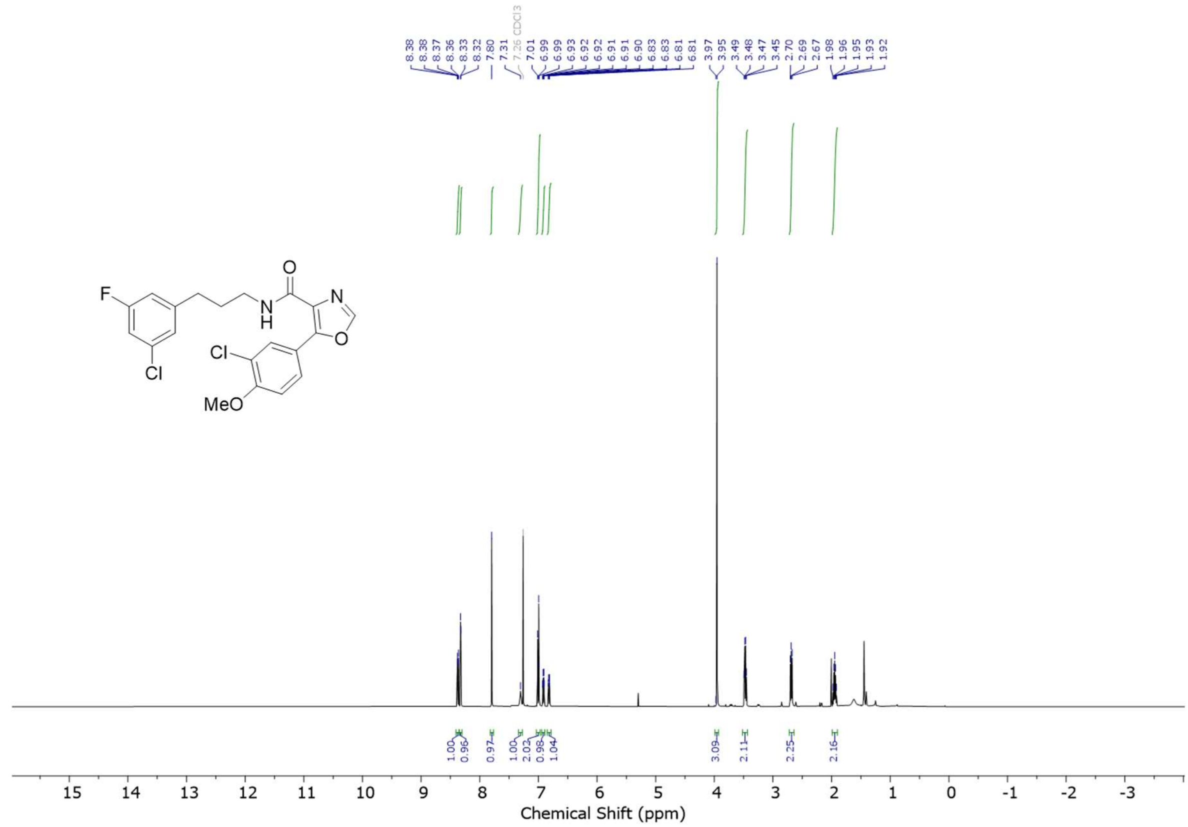

5-(3-Chloro-4-methoxyphenyl)-*N*-(3-(3-chloro-5-fluorophenyl)propyl)oxazole-4-carboxamide (43) ^{13}C NMR (126 MHz, $CDCl_3$)

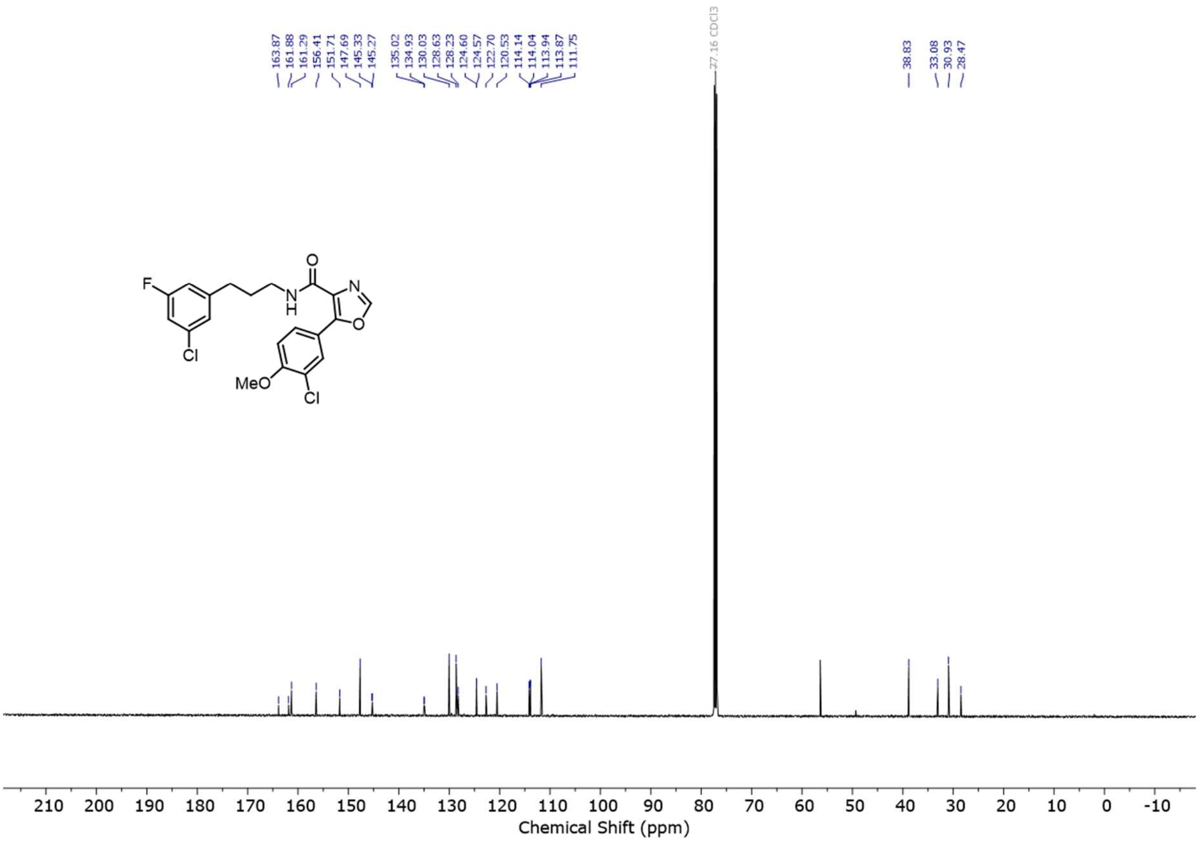

5-(3-Chloro-4-methoxyphenyl)-*N*-(3-(3-chloro-5-fluorophenyl)propyl)oxazole-4-carboxamide (43) ^{19}F NMR (470 MHz, $CDCl_3$)

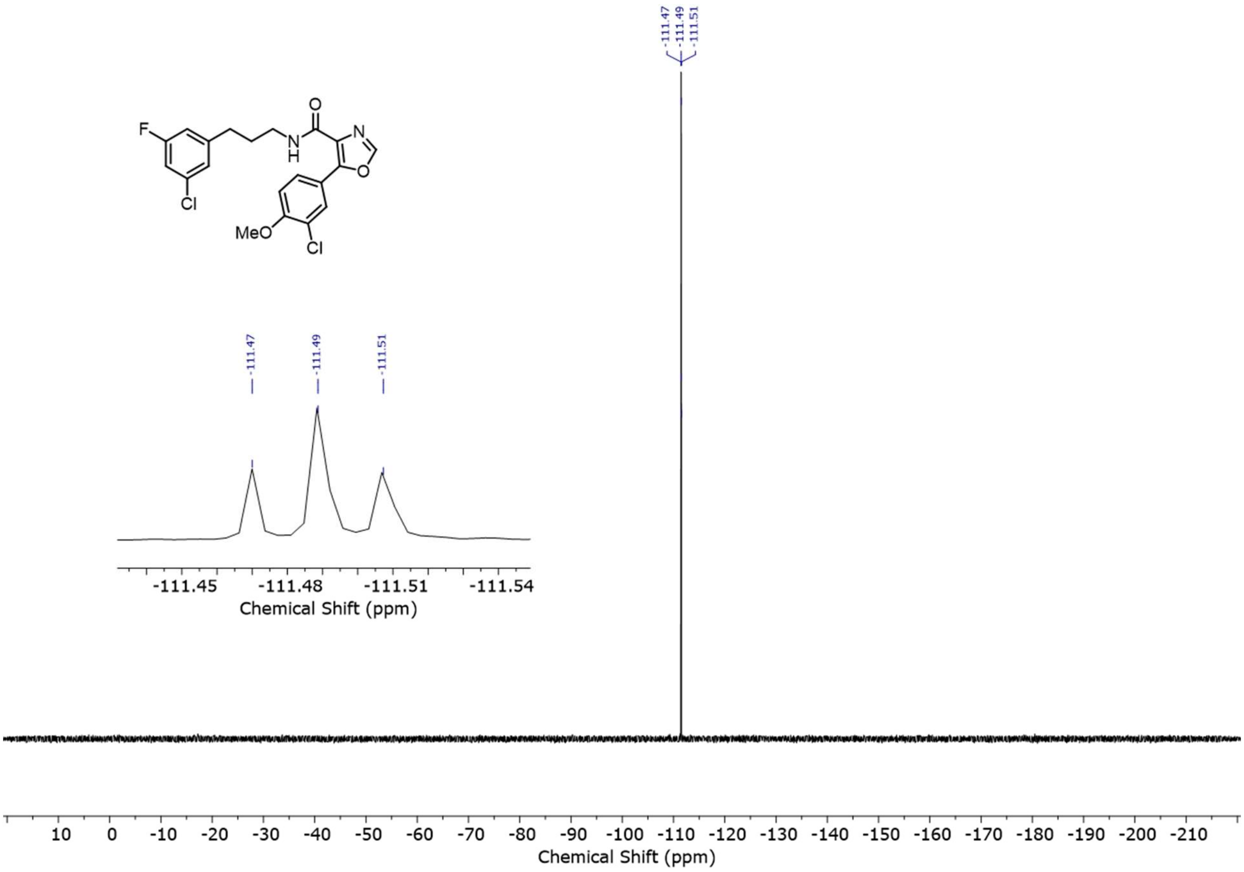

1-((6-Chloropyridin-3-yl)methyl)-2,4,6-triphenylpyridin-1-ium Tetrafluoroborate (S-2) 1H NMR (500 MHz, $CDCl_3$)

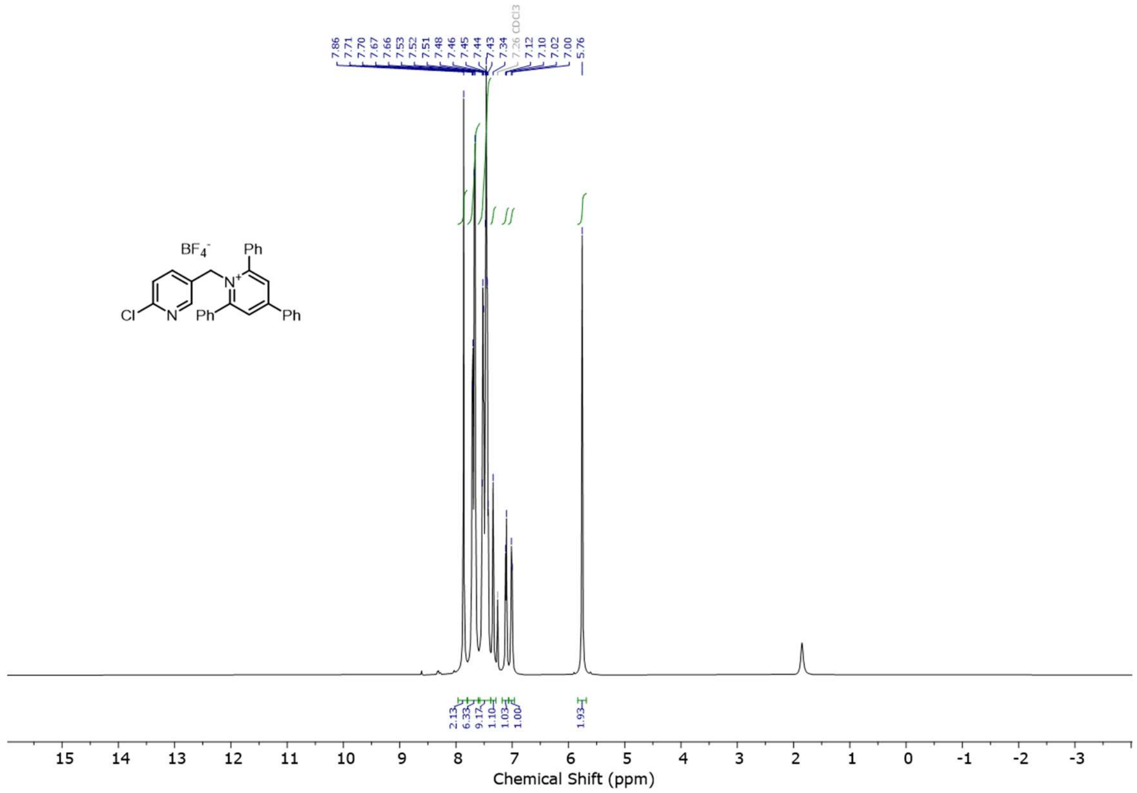

1-((6-Chloropyridin-3-yl)methyl)-2,4,6-triphenylpyridin-1-ium Tetrafluoroborate (S-2) ^{13}C NMR (126 MHz, $CDCl_3$)

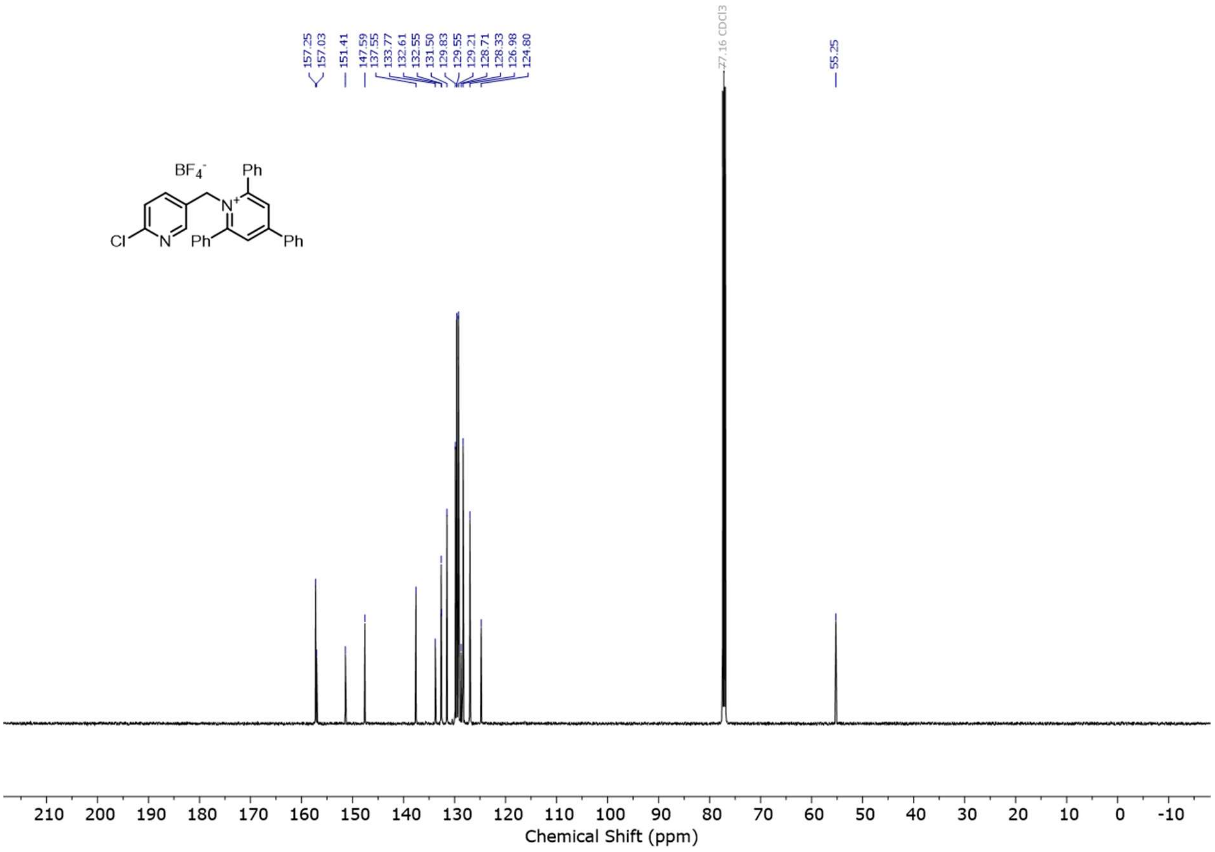

1-((6-Chloropyridin-3-yl)methyl)-2,4,6-triphenylpyridin-1-ium Tetrafluoroborate (S-2) ^{19}F NMR (470 MHz, $CDCl_3$)

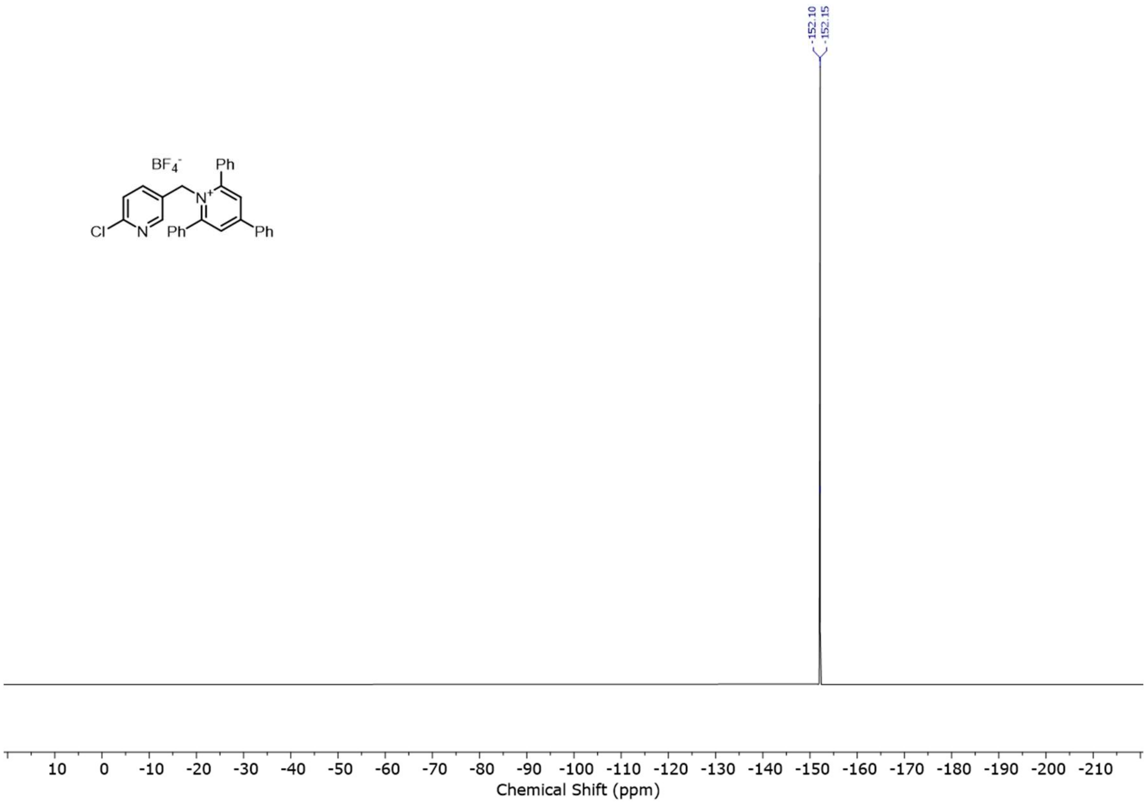

1-(1-(Oxazol-2-yl)azetidin-3-yl)-2,4,6-triphenylpyridin-1-ium Tetrafluoroborate (S-4) ^{1}H NMR (500 MHz, $CDCl_3$)

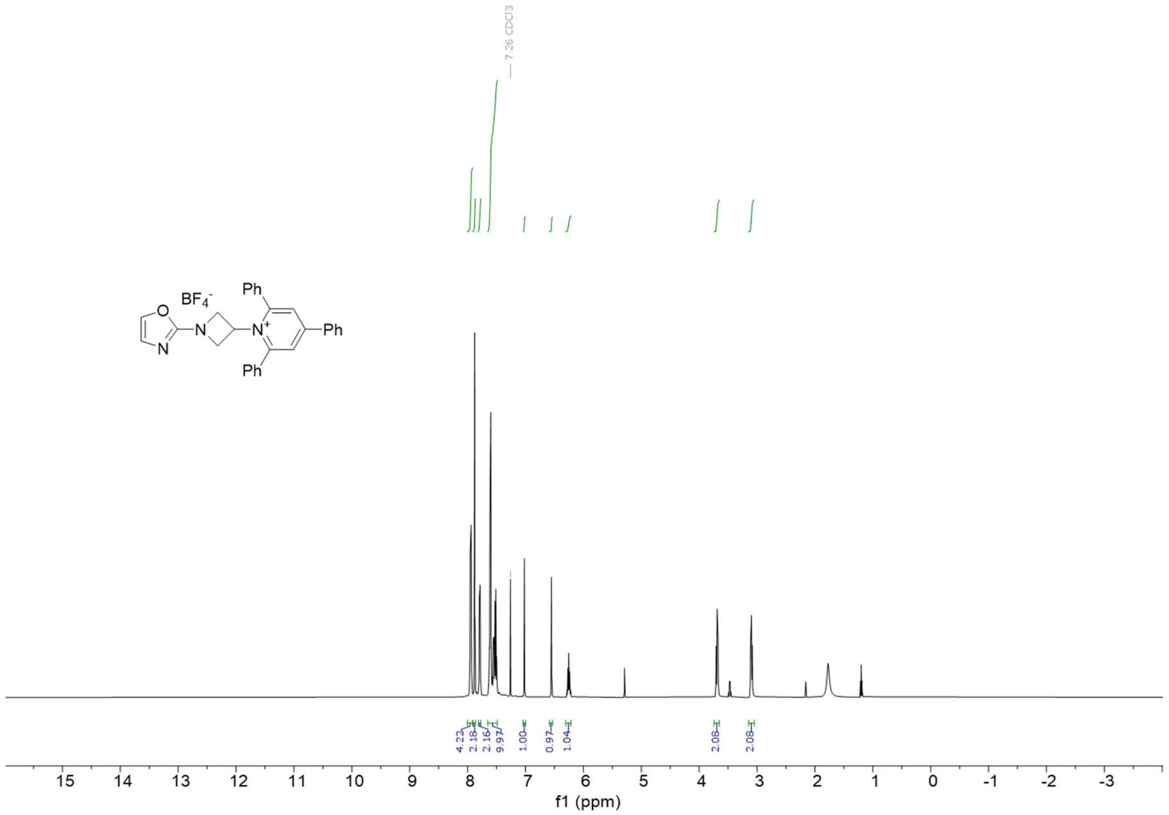

1-(1-(Oxazol-2-yl)azetidin-3-yl)-2,4,6-triphenylpyridin-1-ium Tetrafluoroborate (S-4) ^{13}C NMR (126 MHz, $CDCl_3$)

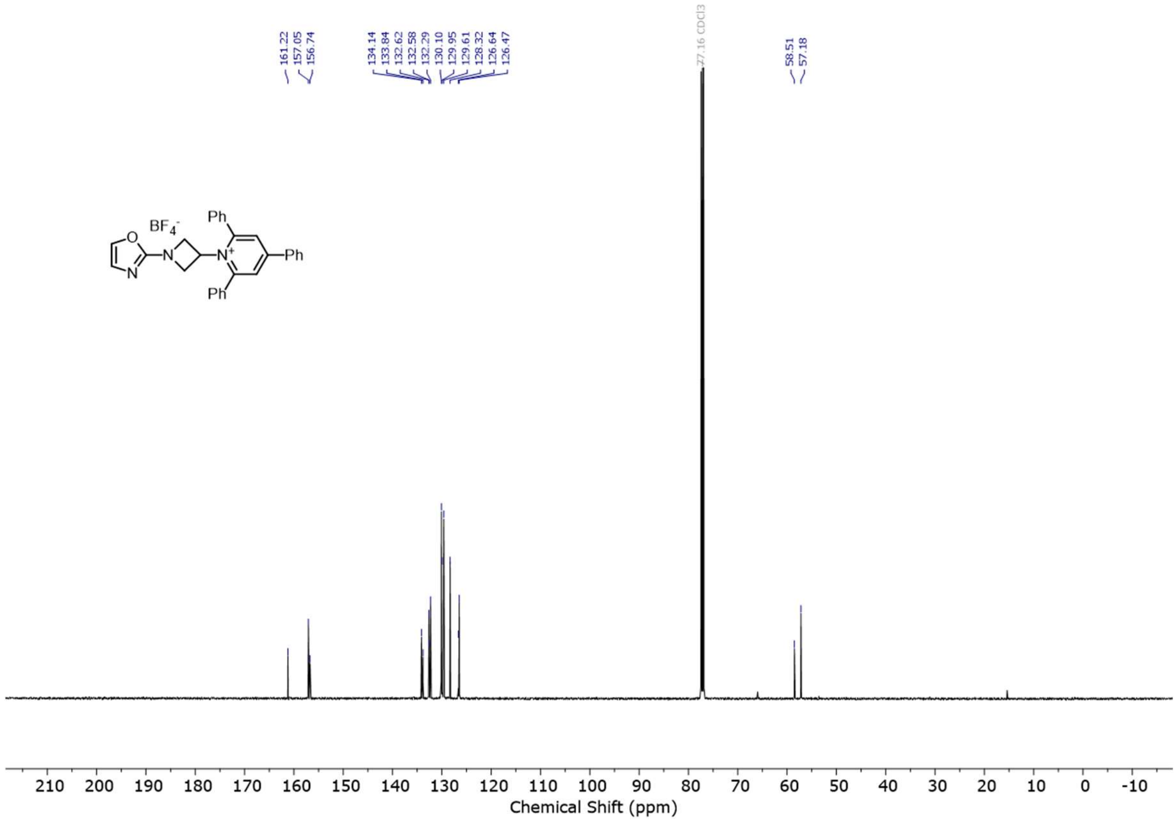

1-(1-(Oxazol-2-yl)azetidin-3-yl)-2,4,6-triphenylpyridin-1-ium Tetrafluoroborate (S-4) ^{19}F NMR (470 MHz, $CDCl_3$)

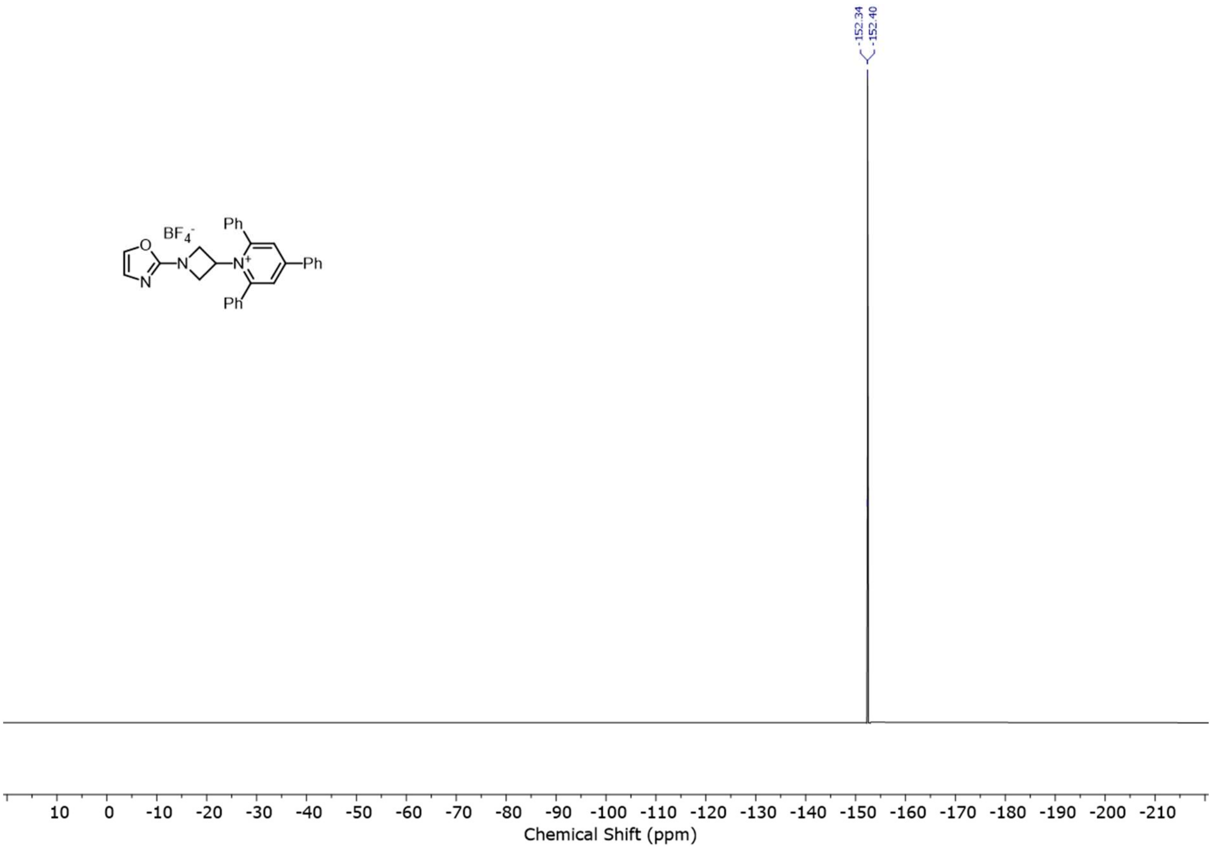

1-(4-Nitrophenethyl)-2,4,6-triphenylpyridin-1-ium tetrafluoroborate (S-5) ^{1}H NMR (400 MHz, $CDCl_3$)

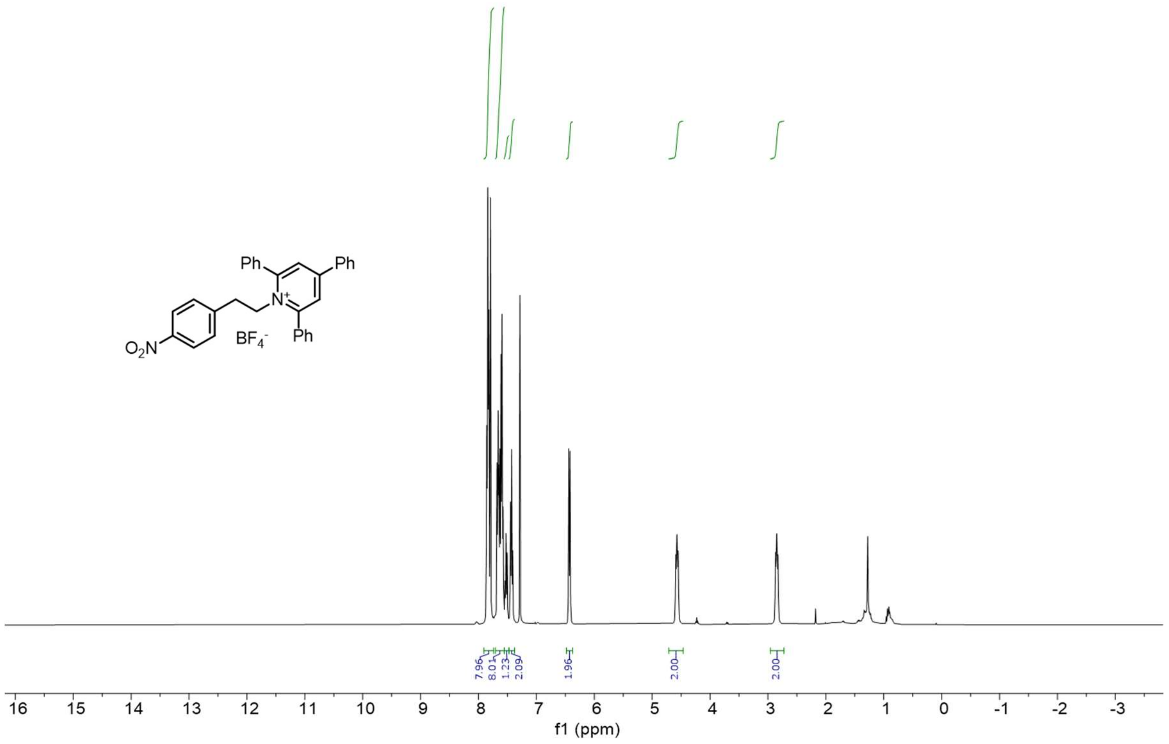

1-(4-Nitrophenethyl)-2,4,6-triphenylpyridin-1-ium tetrafluoroborate (S-5) ^{13}C NMR (101 MHz, CD_3CN)

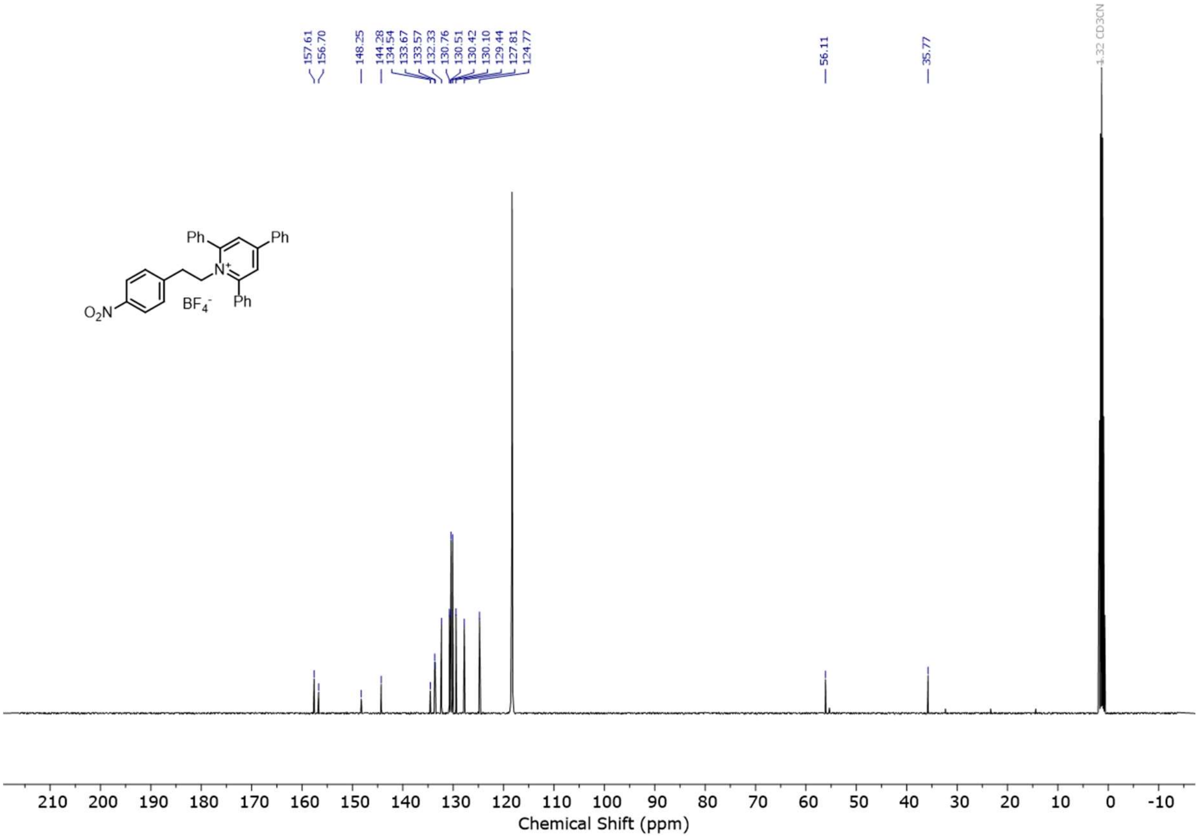

1-(4-Nitrophenethyl)-2,4,6-triphenylpyridin-1-ium tetrafluoroborate (S-5) ^{19}F NMR (376 MHz, CD_3CN)

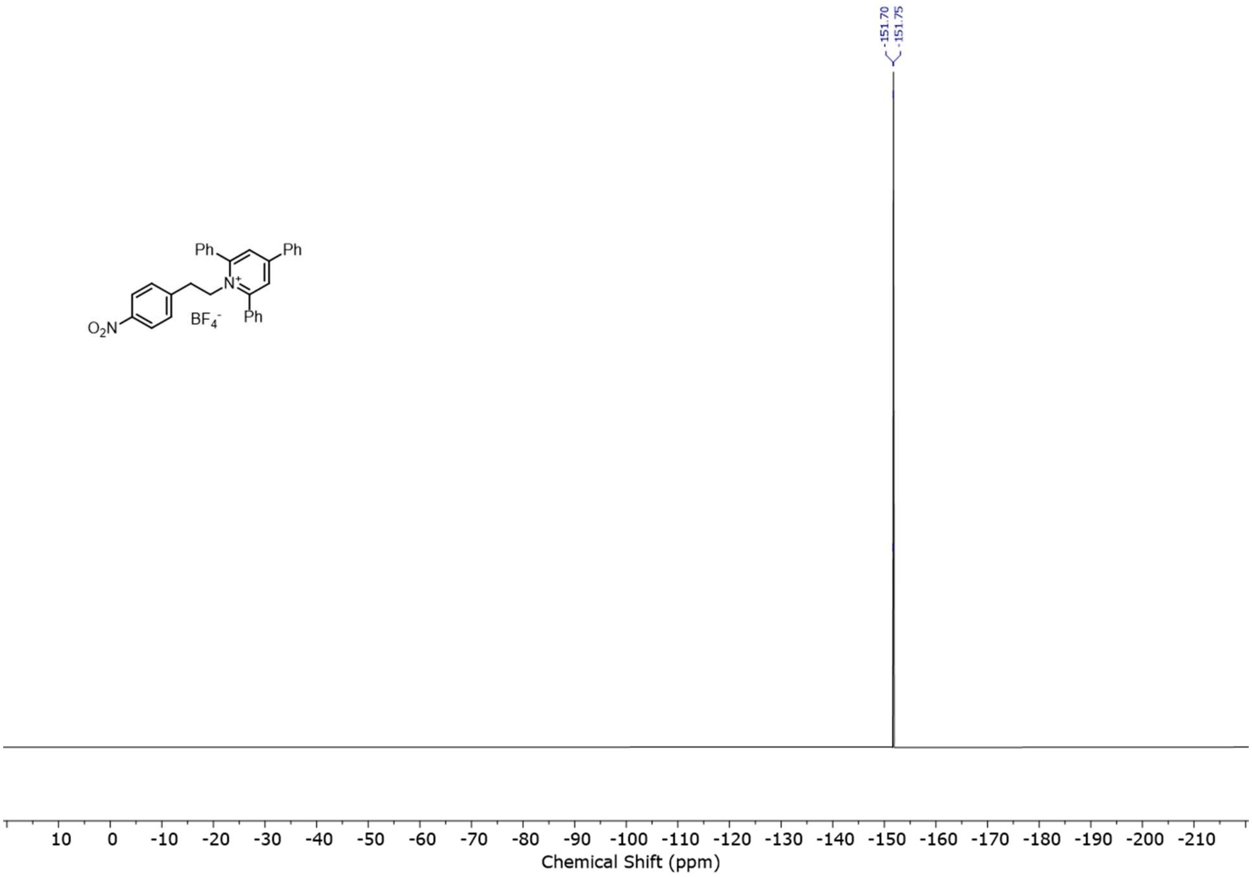

2,4,6-Triphenyl-1-(2-(2-(prop-2-yn-1-yloxy)ethoxy)ethyl)pyridin-1-ium tetrafluoroborate (S-6) ^{1}H NMR (400 MHz, $CDCl_3$)

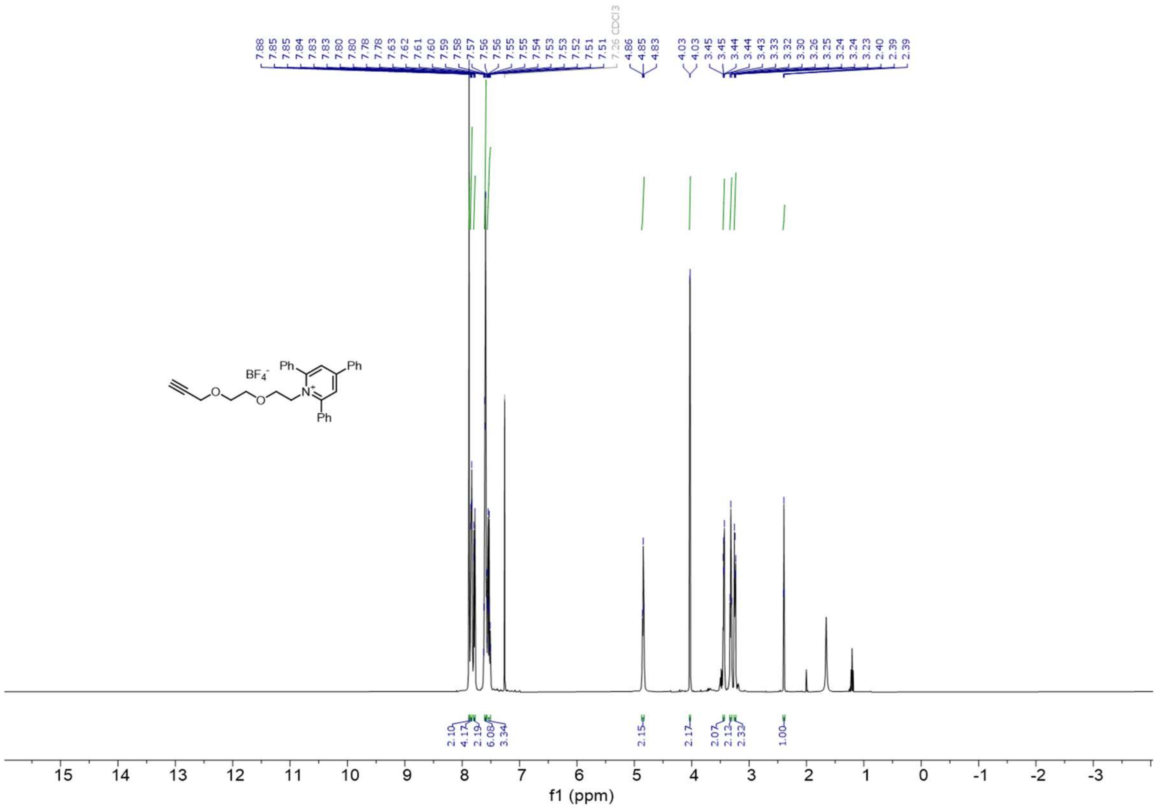

2,4,6-Triphenyl-1-(2-(2-(prop-2-yn-1-yloxy)ethoxy)ethyl)pyridin-1-ium tetrafluoroborate (S-6) ^{13}C NMR (101 MHz, $CDCl_3$)

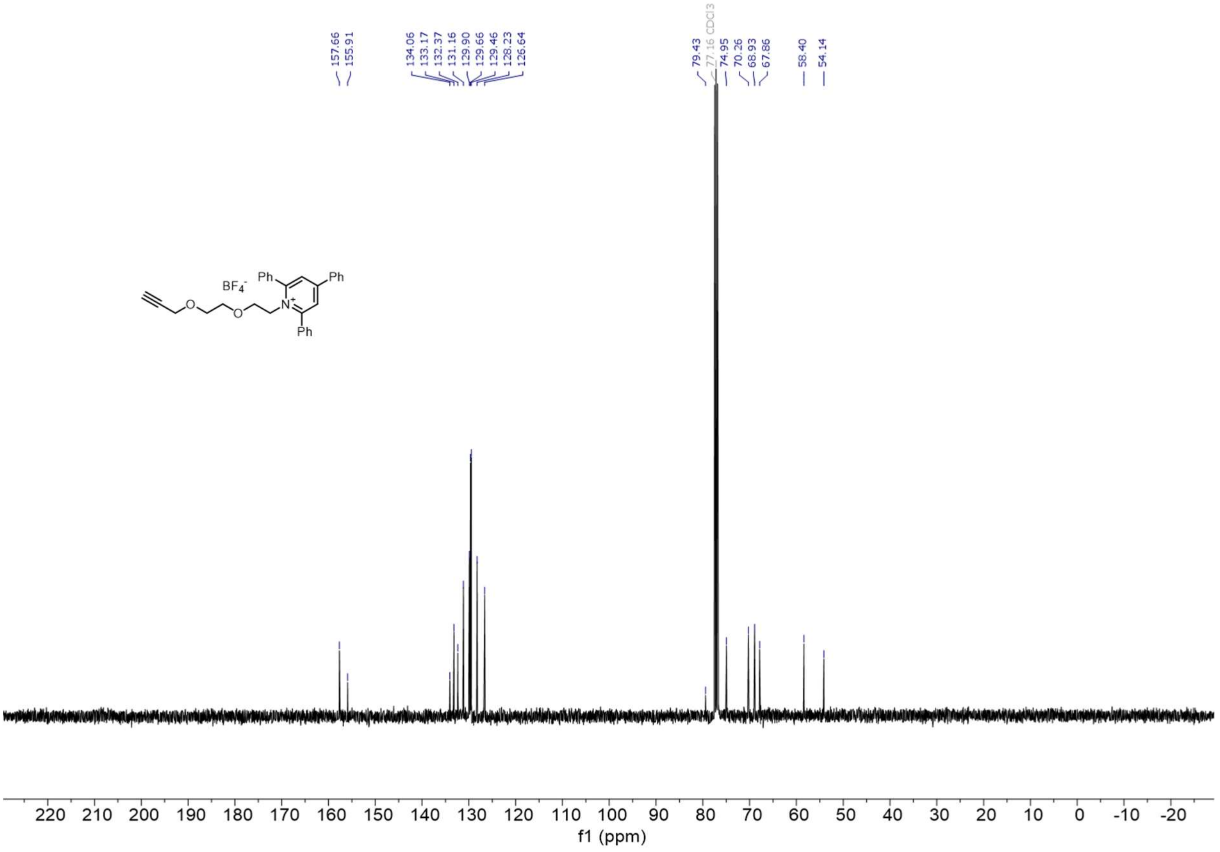

2,4,6-Triphenyl-1-(2-(2-(prop-2-yn-1-yloxy)ethoxy)ethyl)pyridin-1-ium tetrafluoroborate (S-6) ^{19}F NMR (377 MHz, $CDCl_3$)

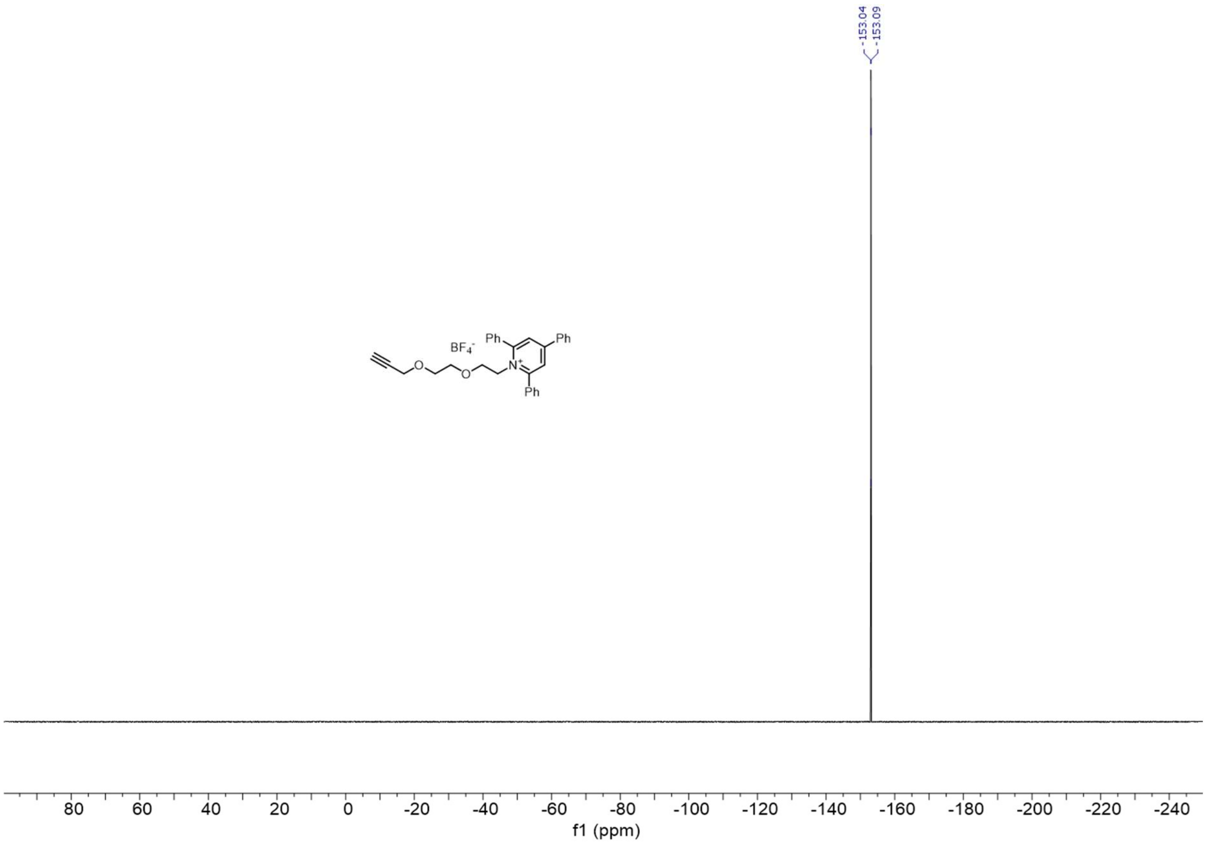

1-(2-(2,3-Bis(*tert*-butoxycarbonyl)guanidino)ethyl)-2,4,6-triphenylpyridin-1-ium tetrafluoroborate (S-8) ^{1}H NMR (500 MHz, $CDCl_3$)

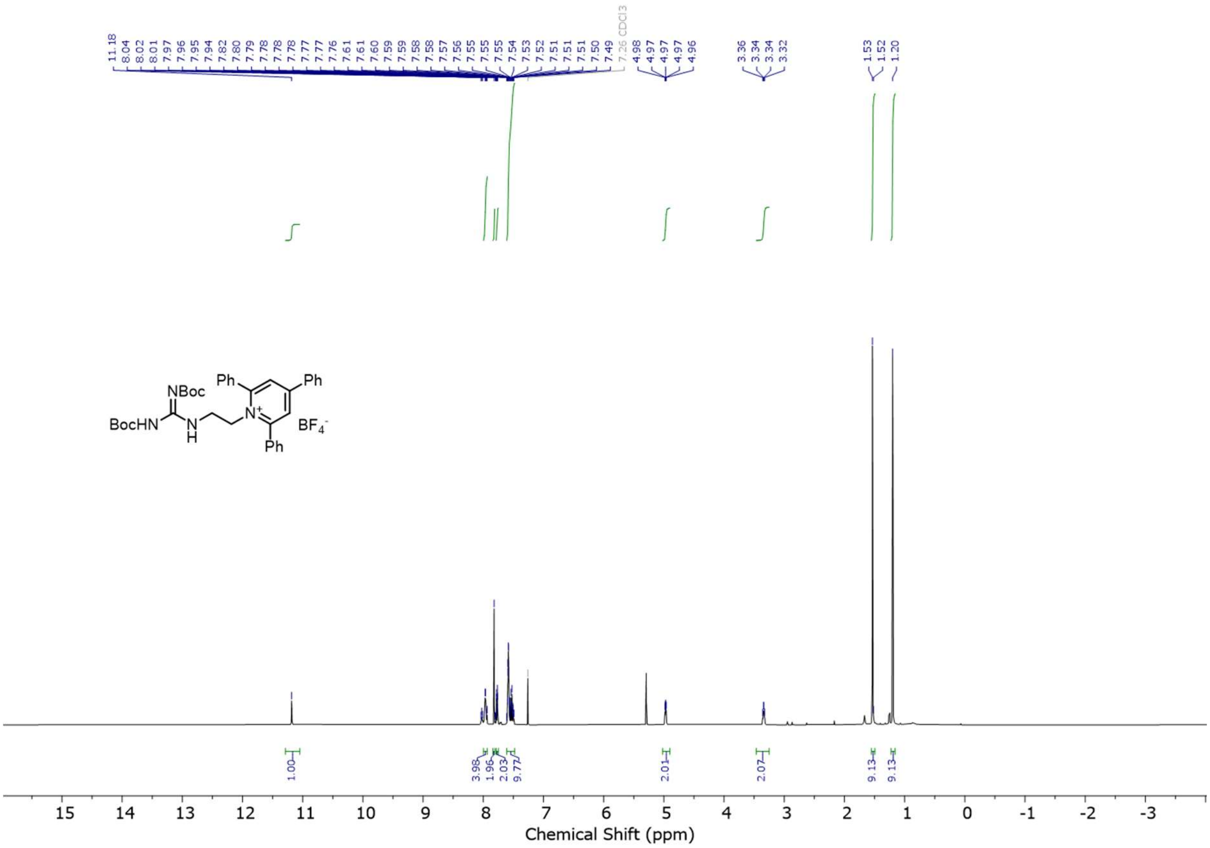

1-(2-(2,3-Bis(*tert*-butoxycarbonyl)guanidino)ethyl)-2,4,6-triphenylpyridin-1-ium tetrafluoroborate (S-8) ^{13}C NMR (126 MHz, $CDCl_3$)

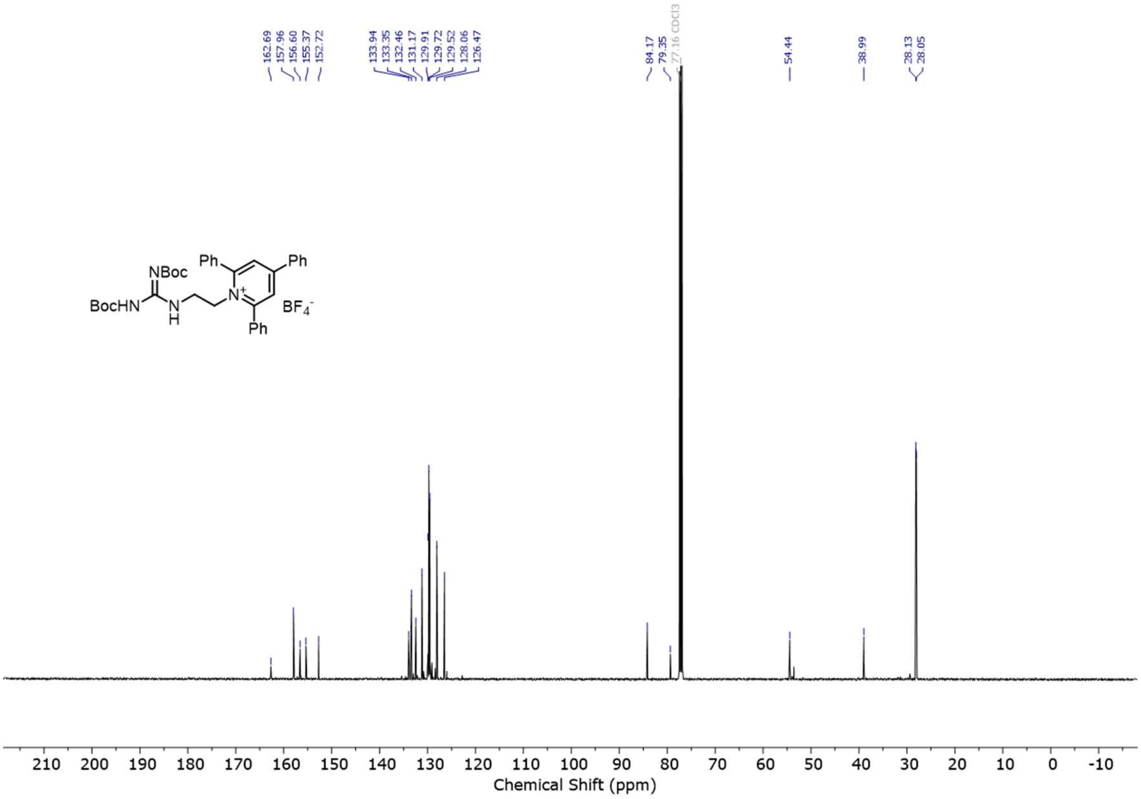

1-(2-(2,3-Bis(*tert*-butoxycarbonyl)guanidino)ethyl)-2,4,6-triphenylpyridin-1-ium tetrafluoroborate (S-8) ^{19}F NMR (470 MHz, $CDCl_3$)

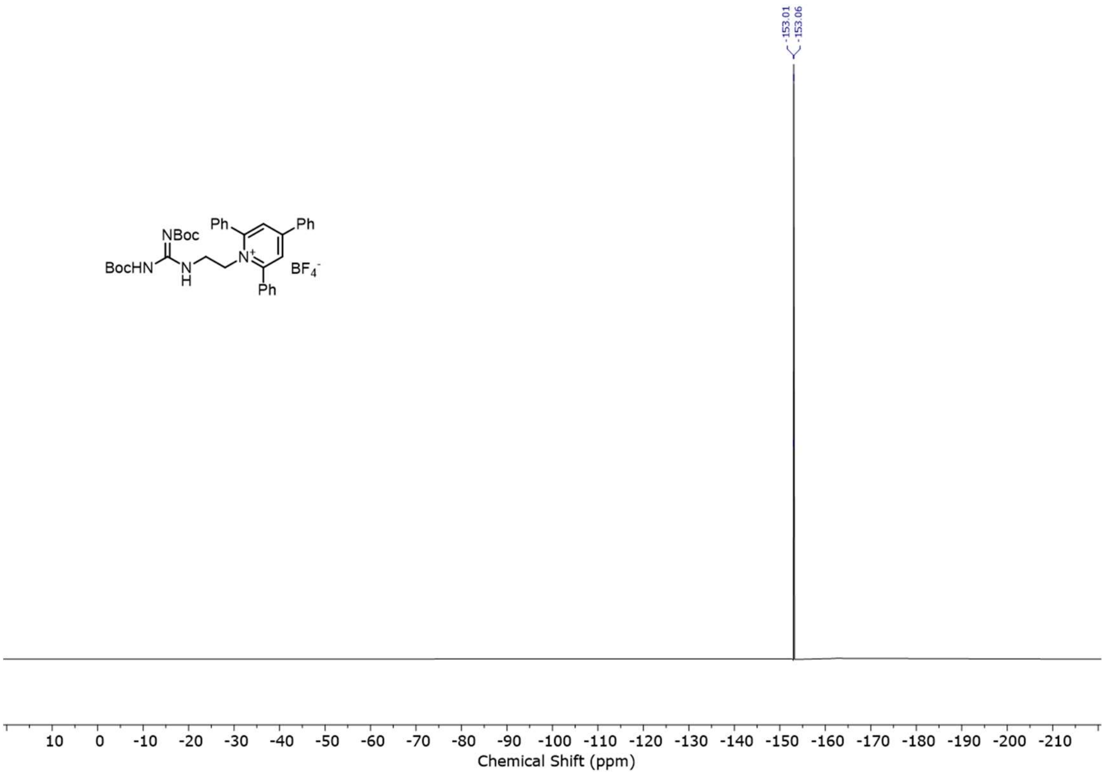

1-(2-(*1H*-Imidazol-5-yl)ethyl)-2,4,6-triphenylpyridin-1-ium tetrafluoroborate (S-9) ^{1}H NMR (500 MHz, DMSO-d_6)

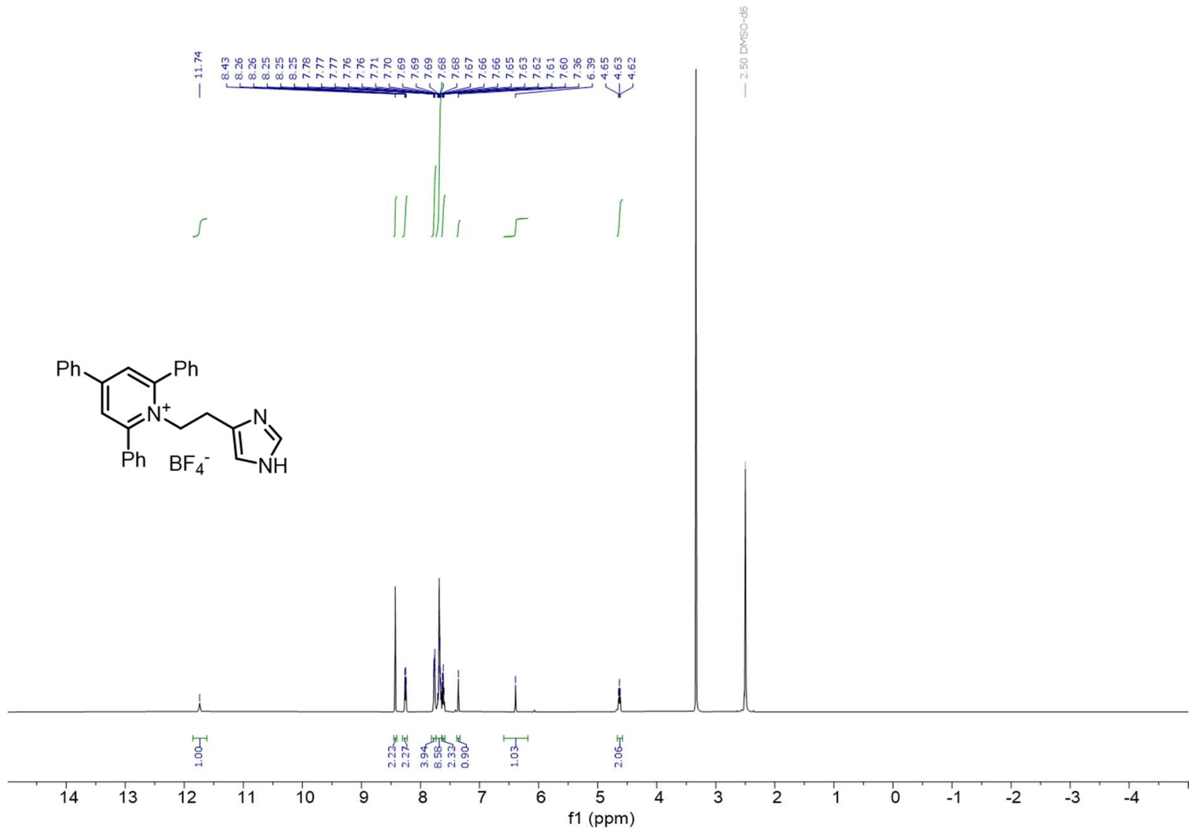

1-(2-(*1H*-Imidazol-5-yl)ethyl)-2,4,6-triphenylpyridin-1-ium tetrafluoroborate (S-9) ^{13}C NMR (126 MHz, DMSO-d_6)

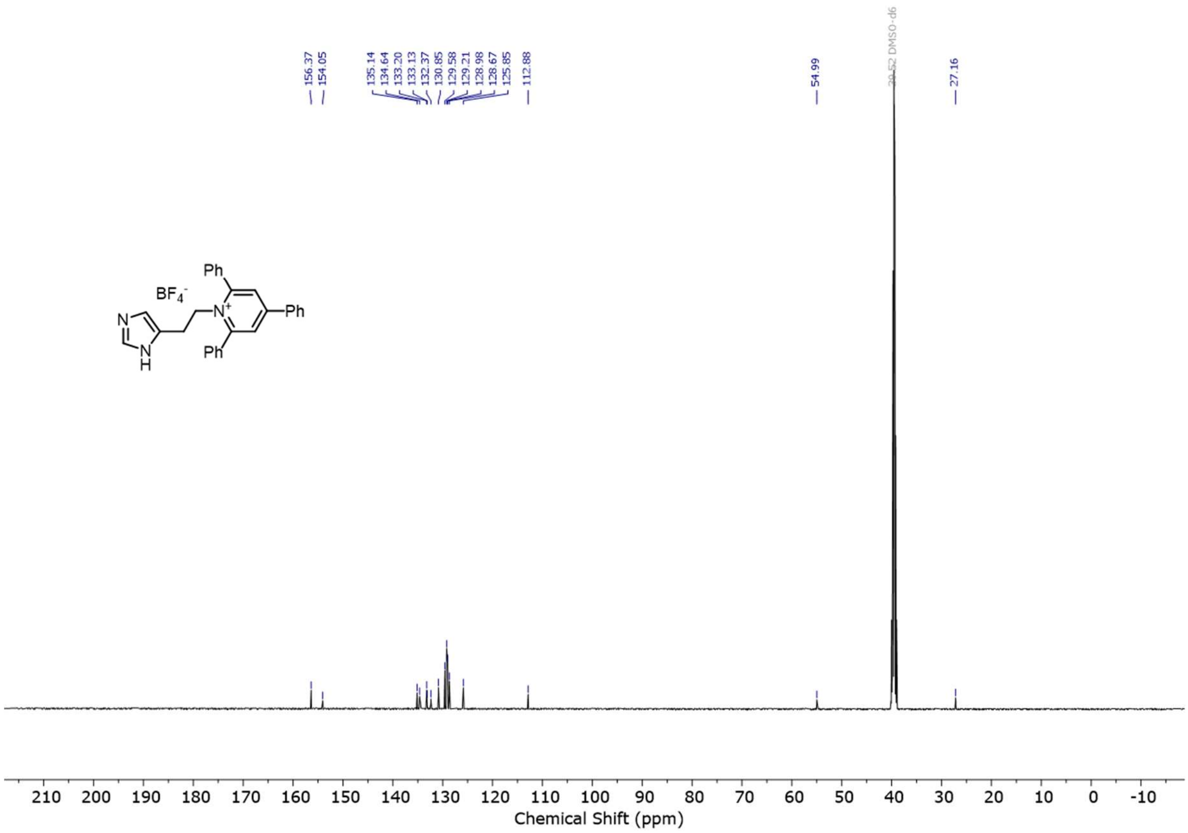

1-(2-(*1H*-Imidazol-5-yl)ethyl)-2,4,6-triphenylpyridin-1-ium tetrafluoroborate (S-9) ^{19}F NMR (470 MHz, DMSO-d_6)

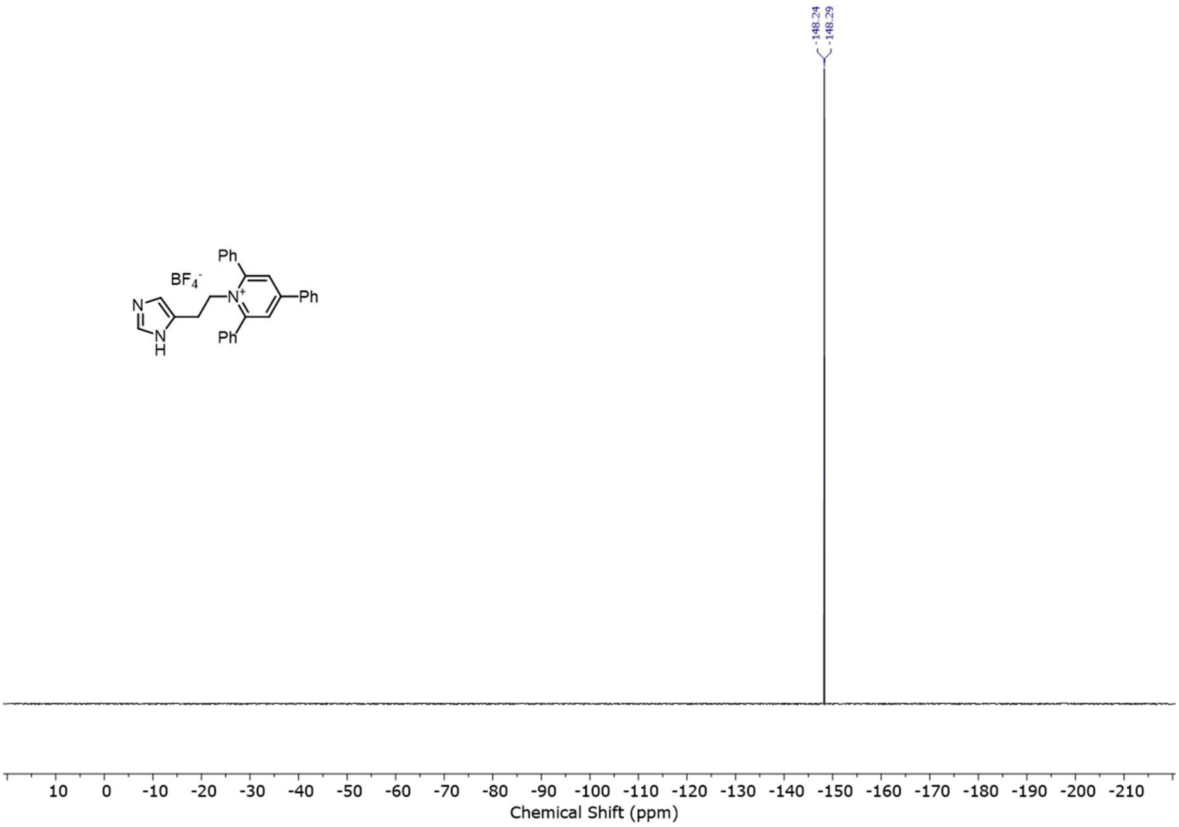

2-(4-Iodophenoxy)ethyl 4-methylbenzenesulfonate (S-11) ^{1}H NMR (400 MHz, $CDCl_3$)

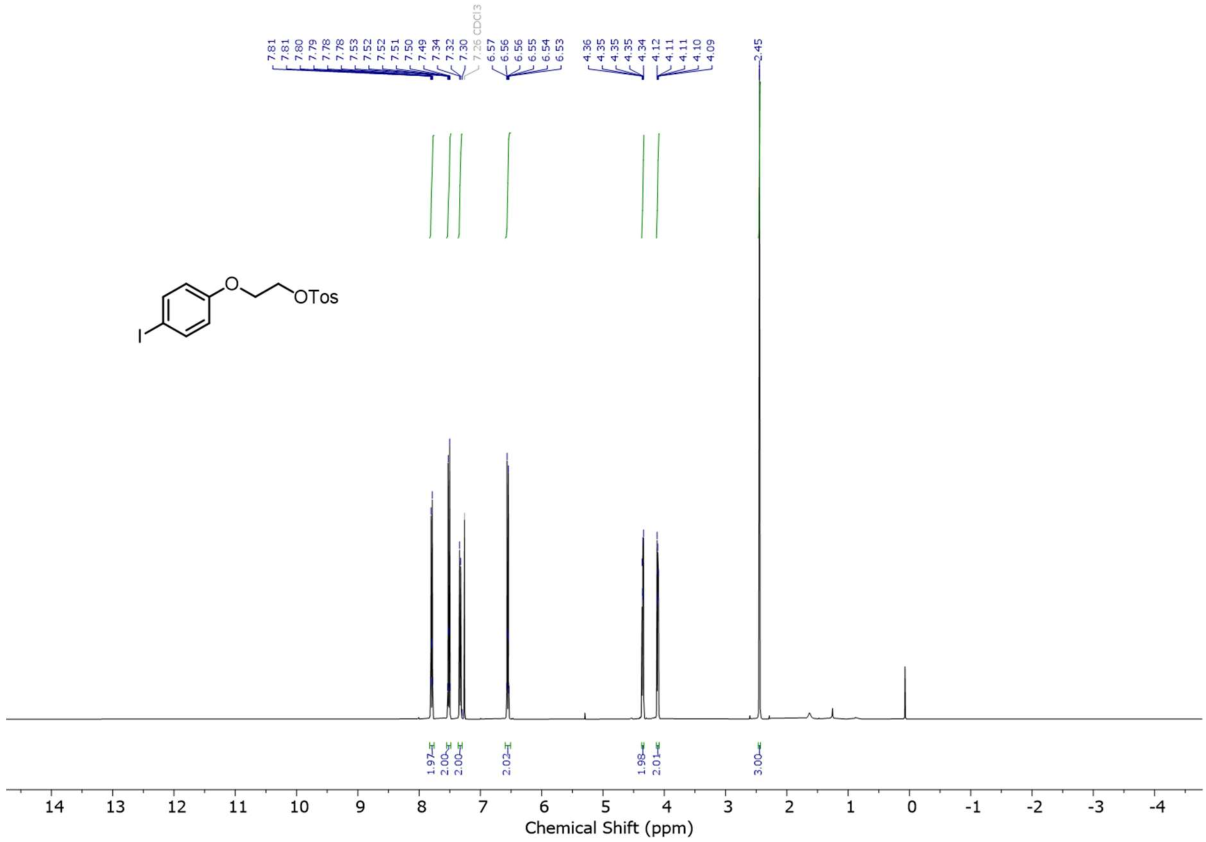

2-(4-Iodophenoxy)ethyl 4-methylbenzenesulfonate (S-11) ^{13}C NMR (101 MHz, $CDCl_3$)

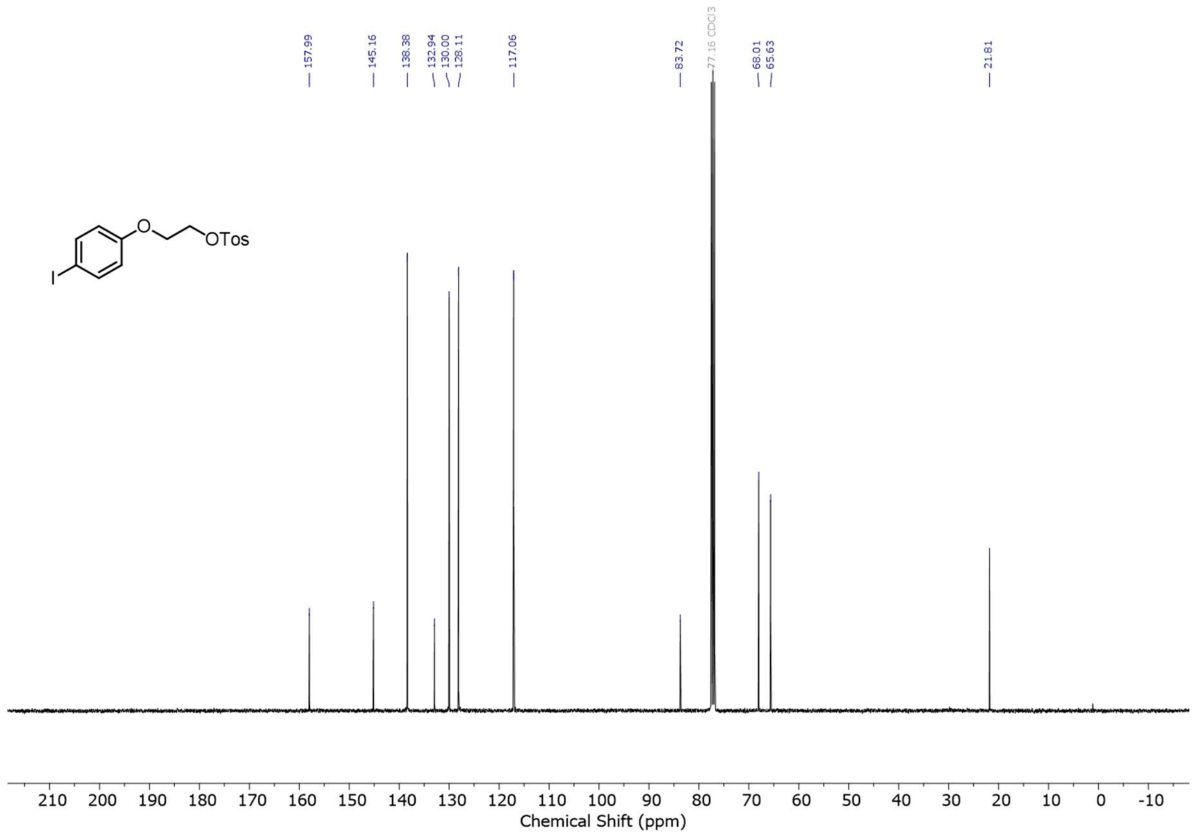

5-Iodo-*N,N,N*-trimethylpyridin-2-aminium Triflate (S-12) ^{1}H NMR (500 MHz, $CDCl_3$)

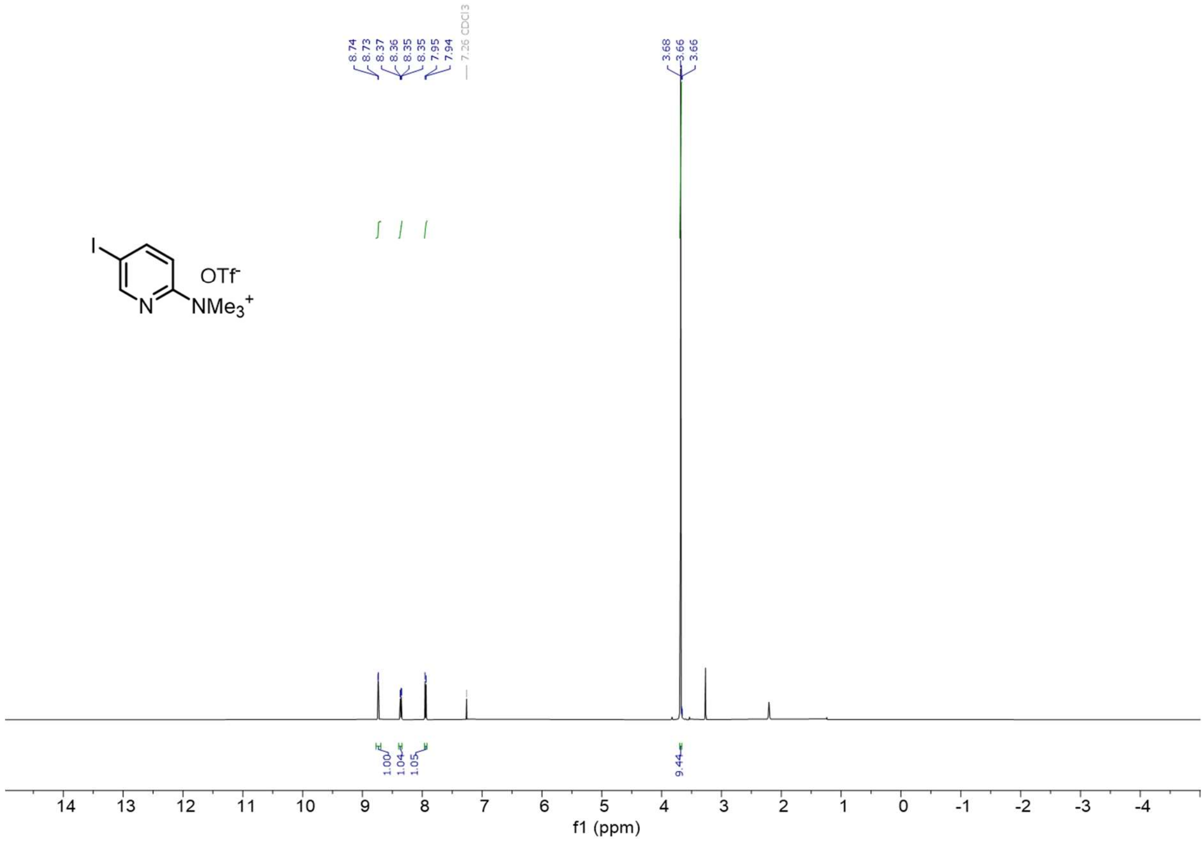

5-Iodo-*N,N,N*-trimethylpyridin-2-aminium Triflate (S-12) {^{19}F}^{13}C NMR (126 MHz, $CDCl_3$)

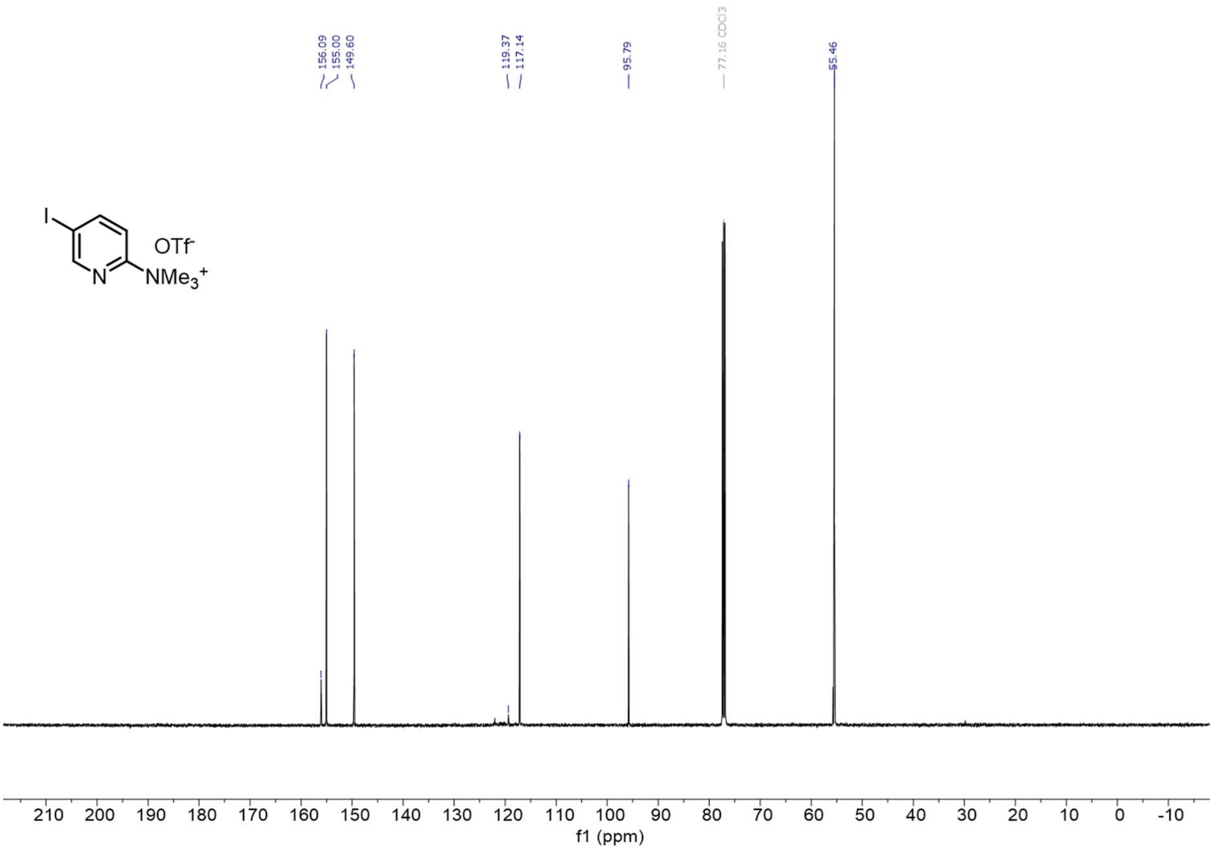

5-Iodo-*N,N,N*-trimethylpyridin-2-aminium Triflate (S-12) ^{19}F NMR (471 MHz, $CDCl_3$)

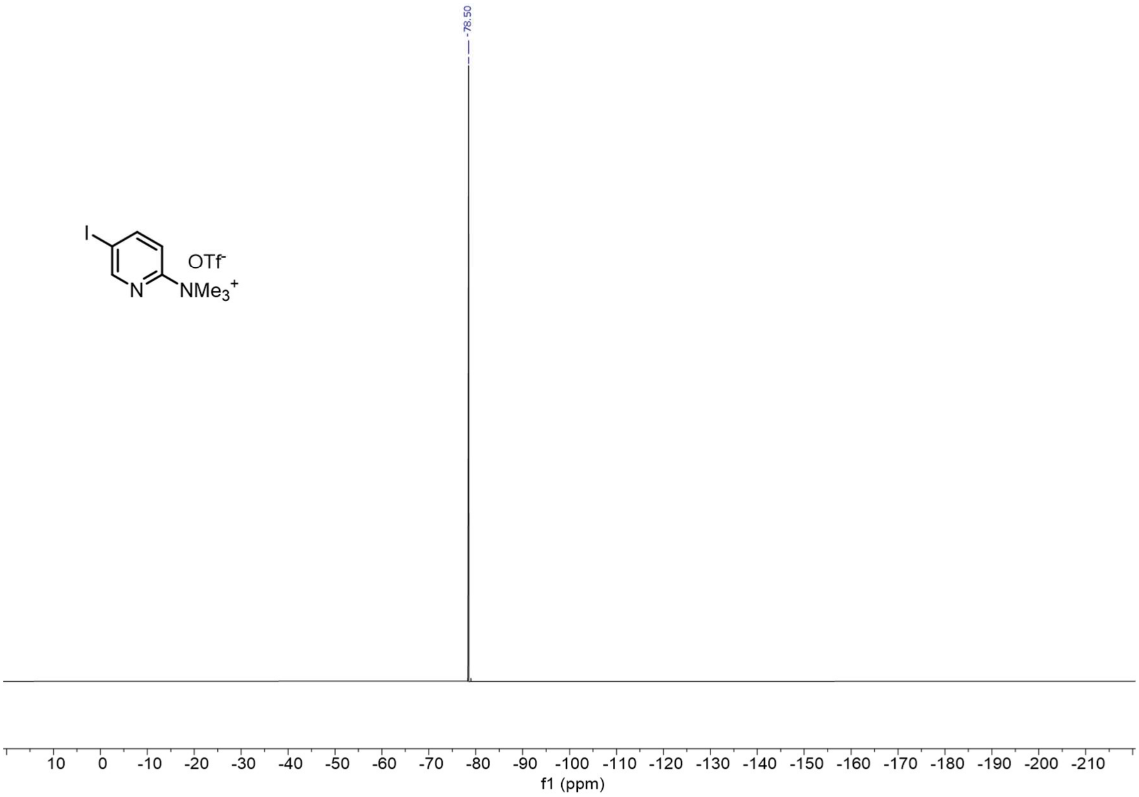

2-Fluoro-5-phenethylpyridine (S-14) ^{1}H NMR (400 MHz, $CDCl_3$)

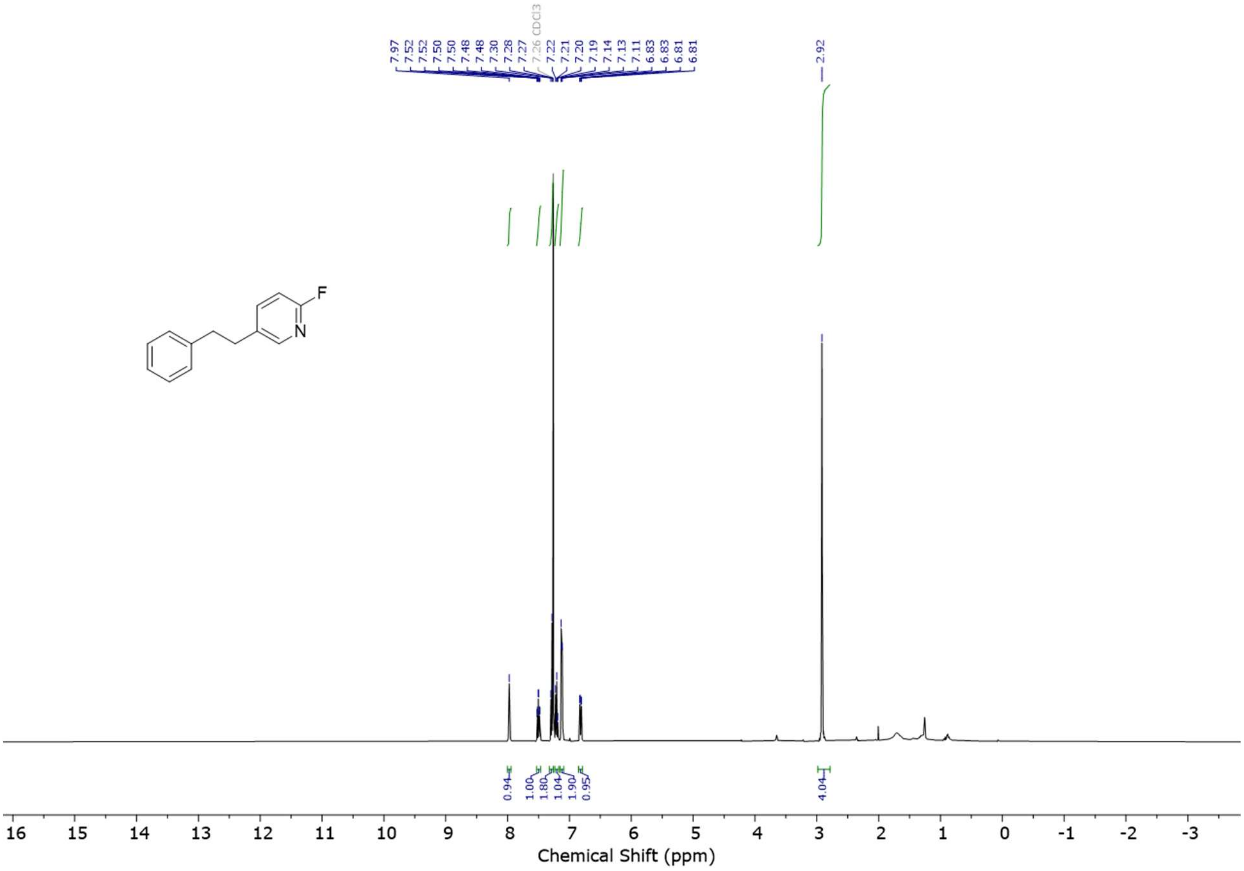

2-Fluoro-5-phenethylpyridine (S-14) ^{13}C NMR (101 MHz, $CDCl_3$)

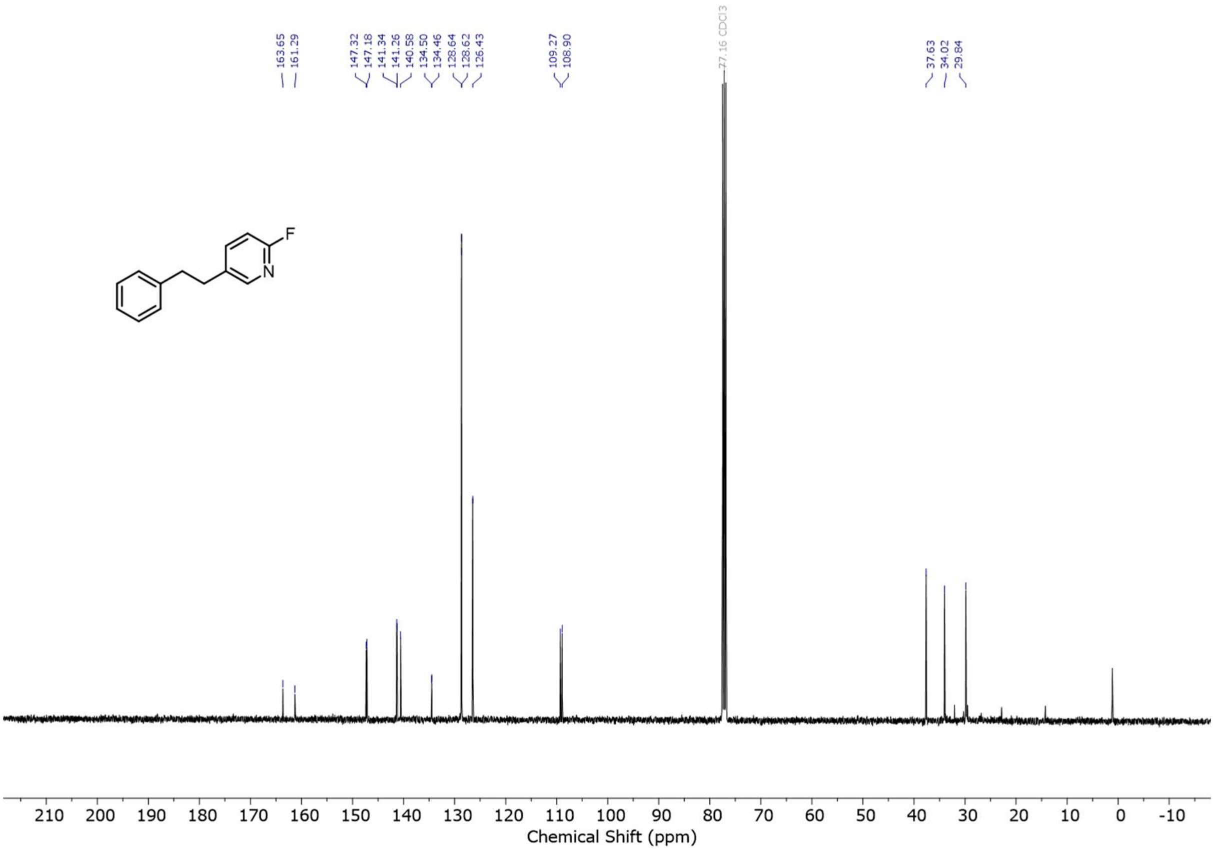

2-Fluoro-5-phenethylpyridine (S-14) ^{19}F NMR (376 MHz, $CDCl_3$)

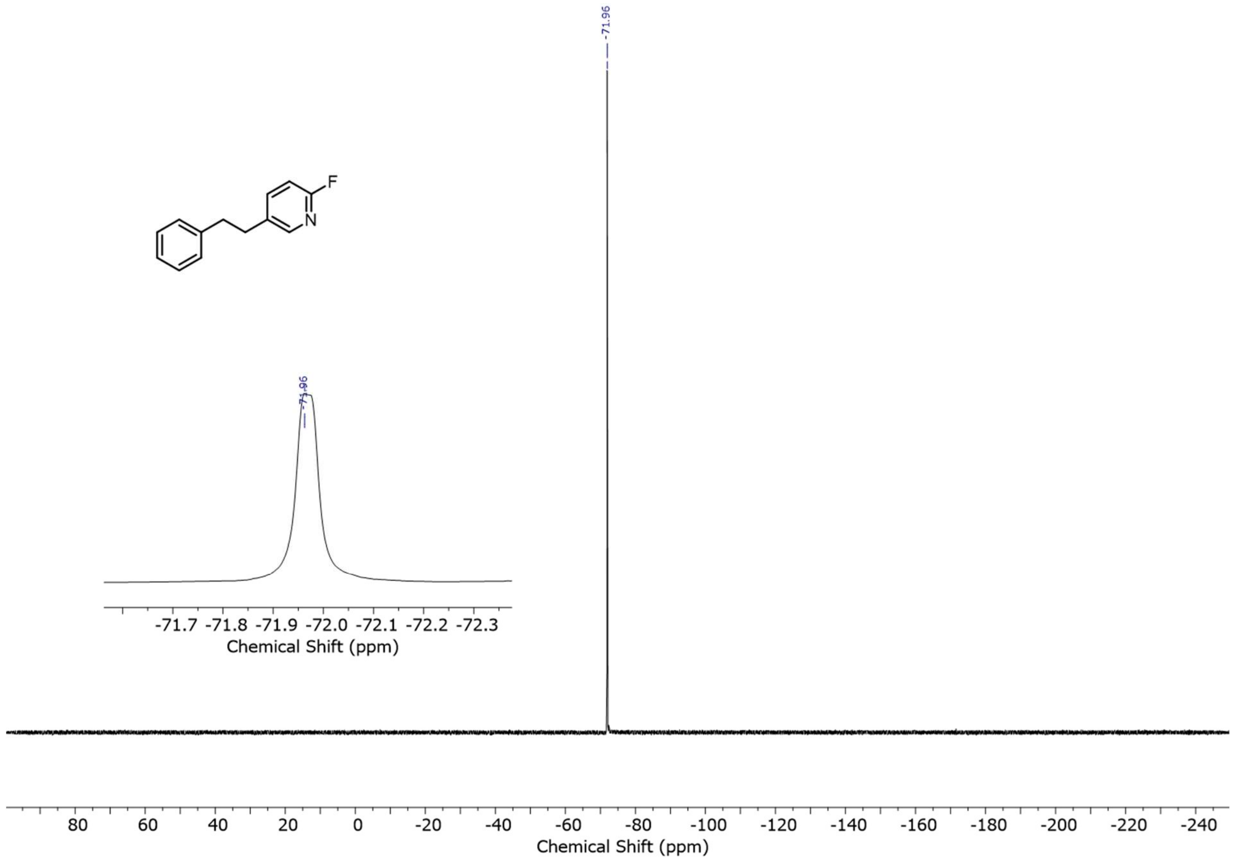

1-(2-Fluoroethoxy)-4-phenethylbenzene (S-16) ^{1}H NMR (500 MHz, $CDCl_3$)

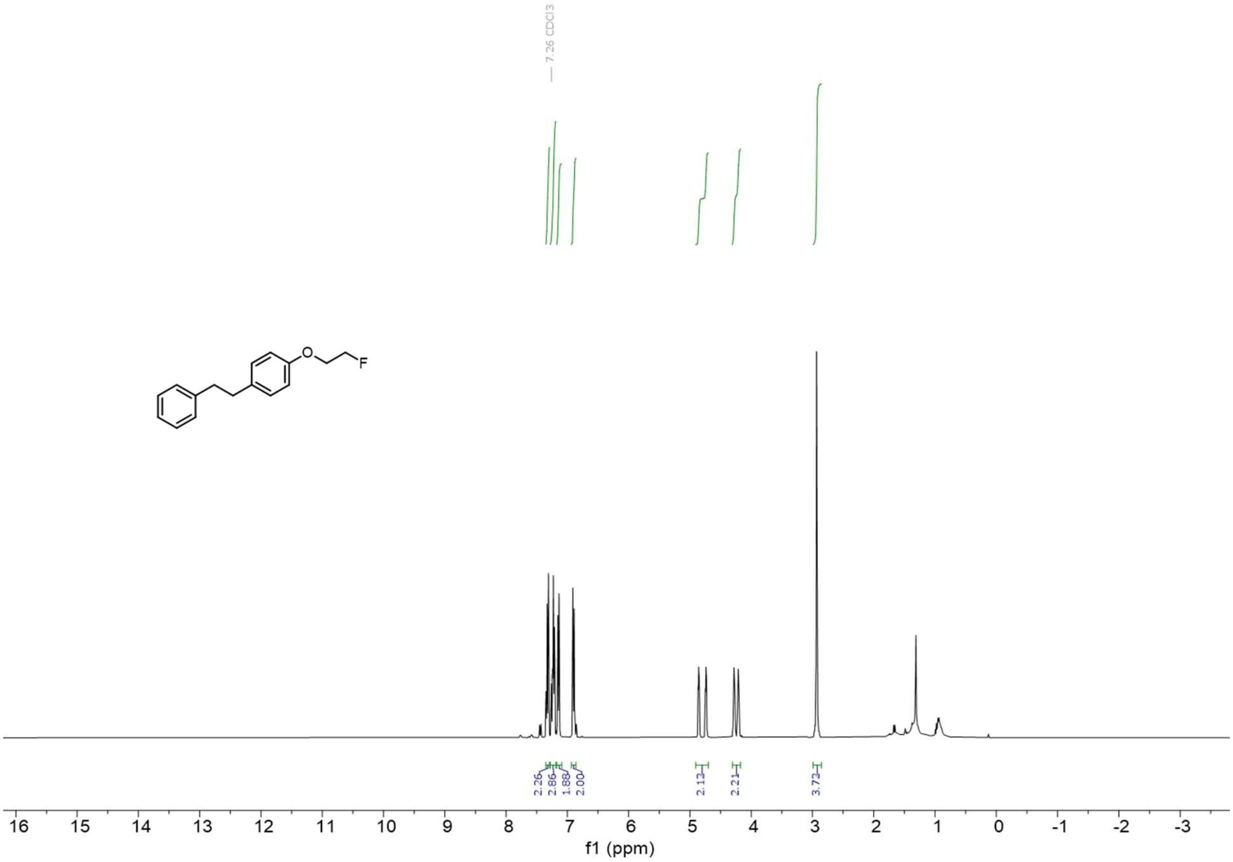

1-(2-Fluoroethoxy)-4-phenethylbenzene (S-16) ^{13}C NMR (126 MHz, $CDCl_3$)

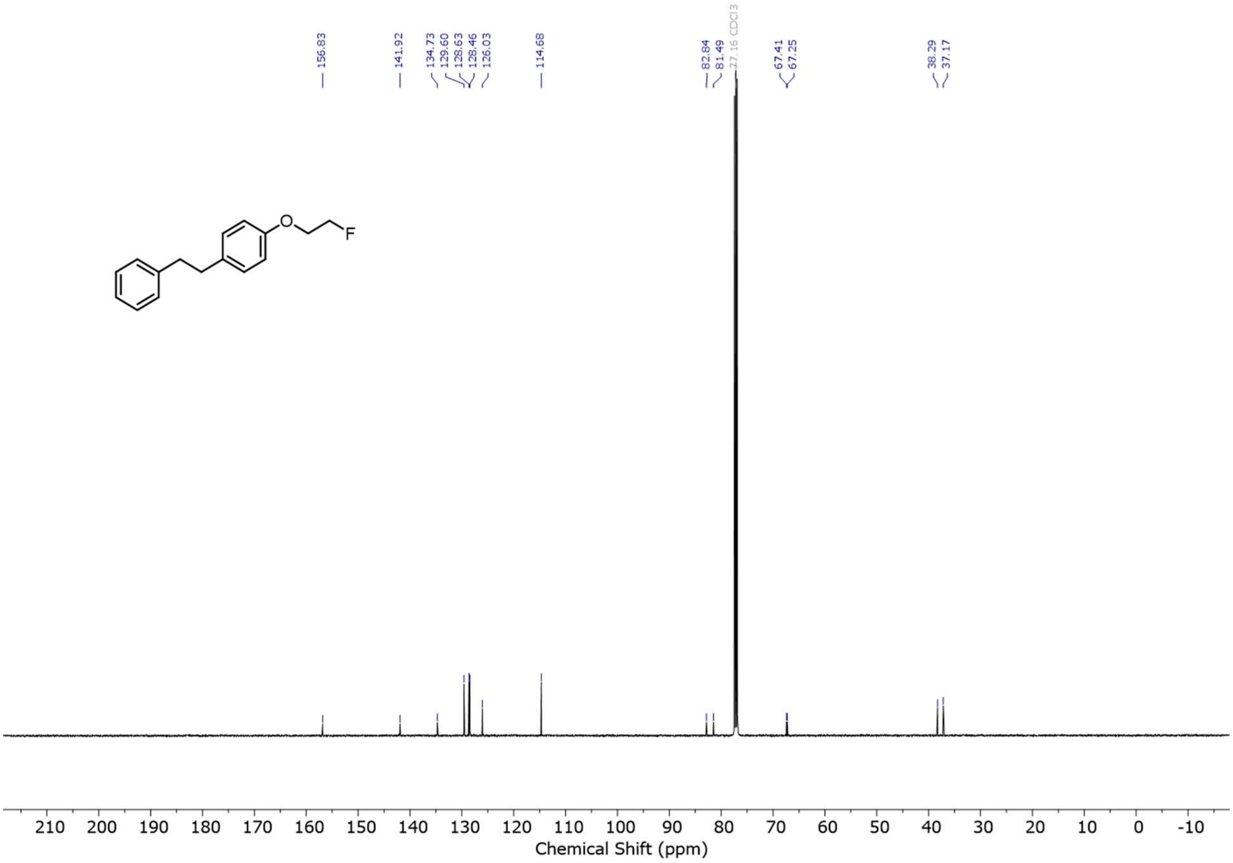

1-(2-Fluoroethoxy)-4-phenethylbenzene (S-16) ^{19}F NMR (377 MHz, $CDCl_3$)

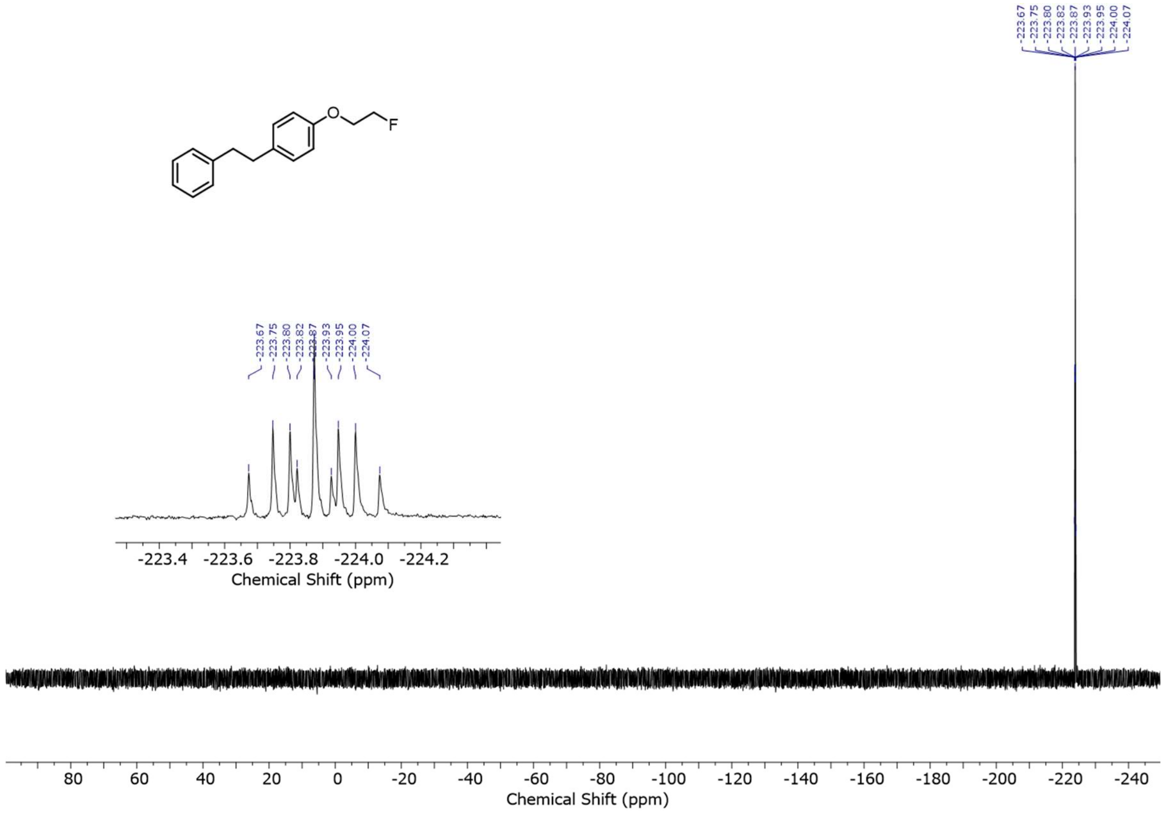

1-Fluoro-2-phenethylbenzene (S-17) ^{1}H NMR (400 MHz, $CDCl_3$)

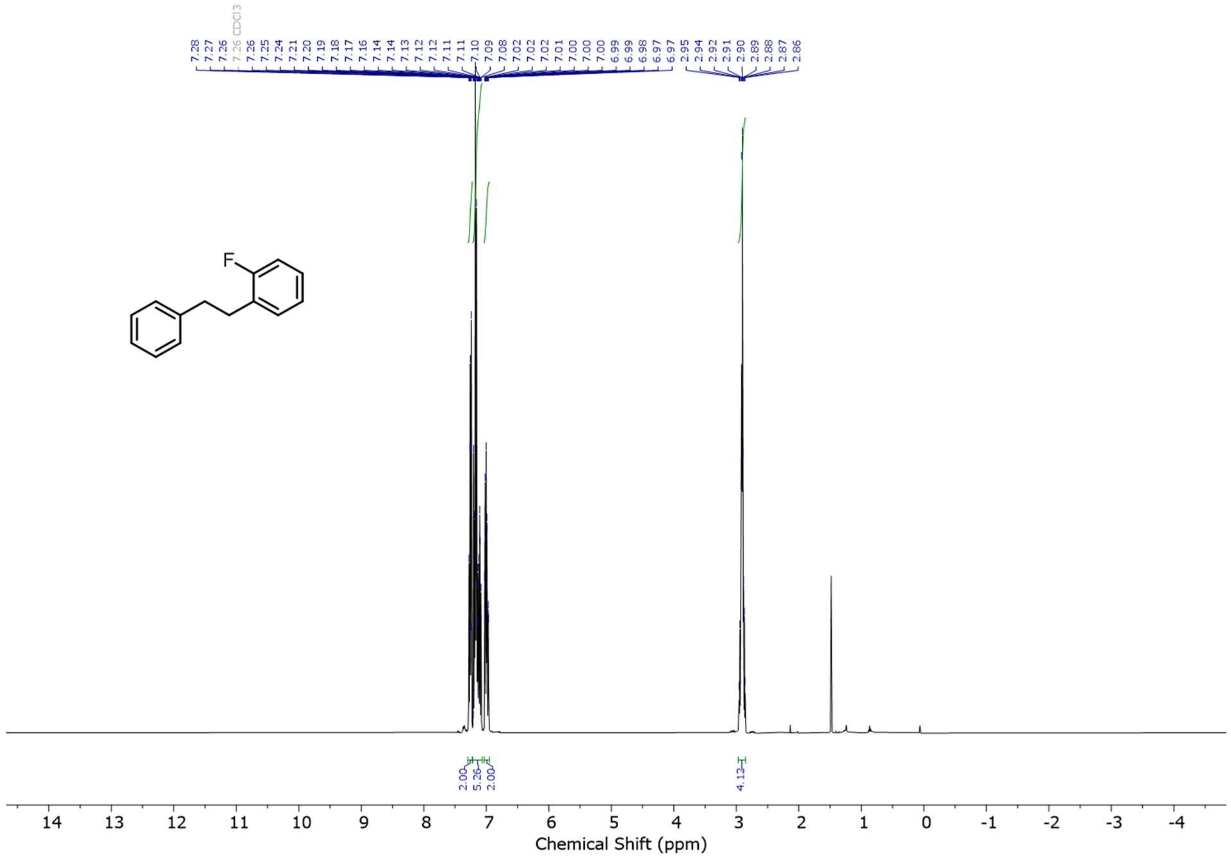

1-Fluoro-2-phenethylbenzene (S-17) ^{13}C NMR (101 MHz, $CDCl_3$)

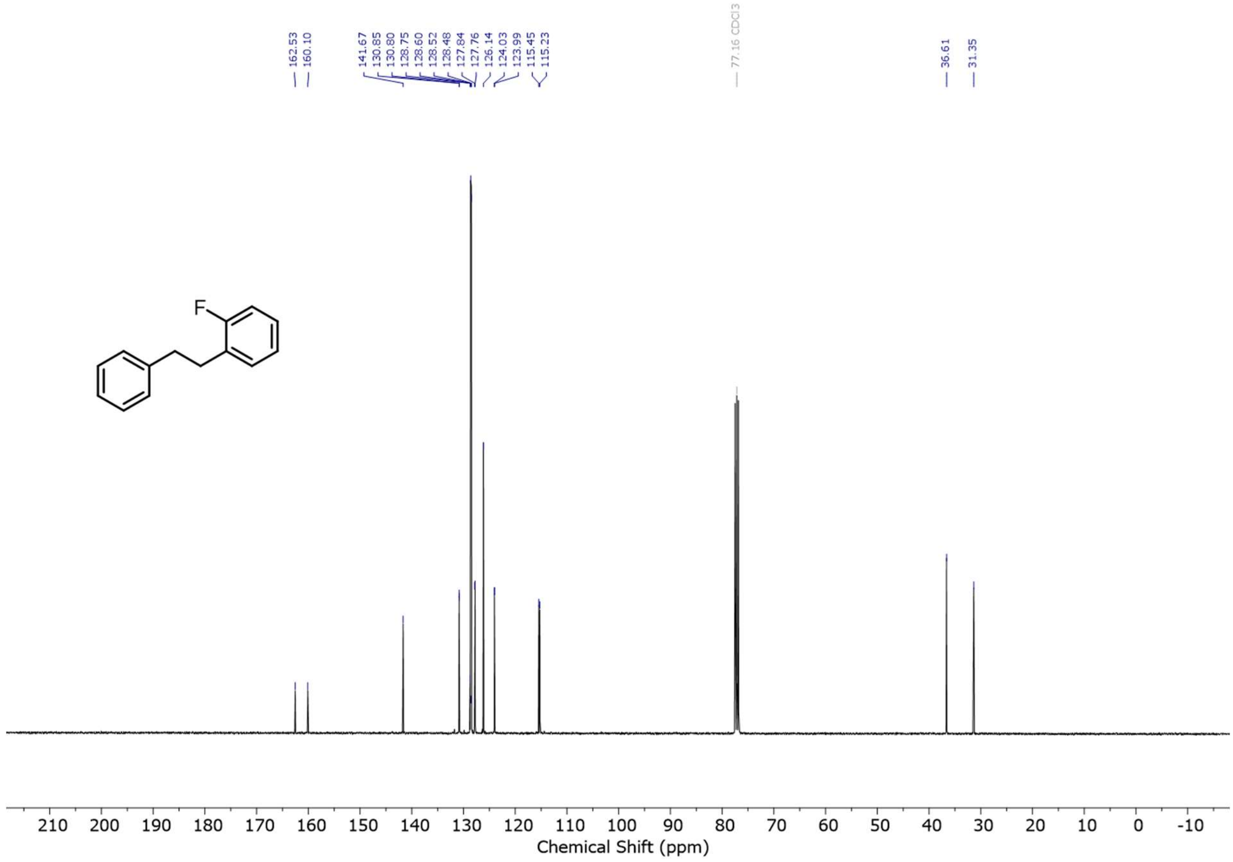

1-Fluoro-2-phenethylbenzene (S-17) ^{19}F NMR (376 MHz, $CDCl_3$)

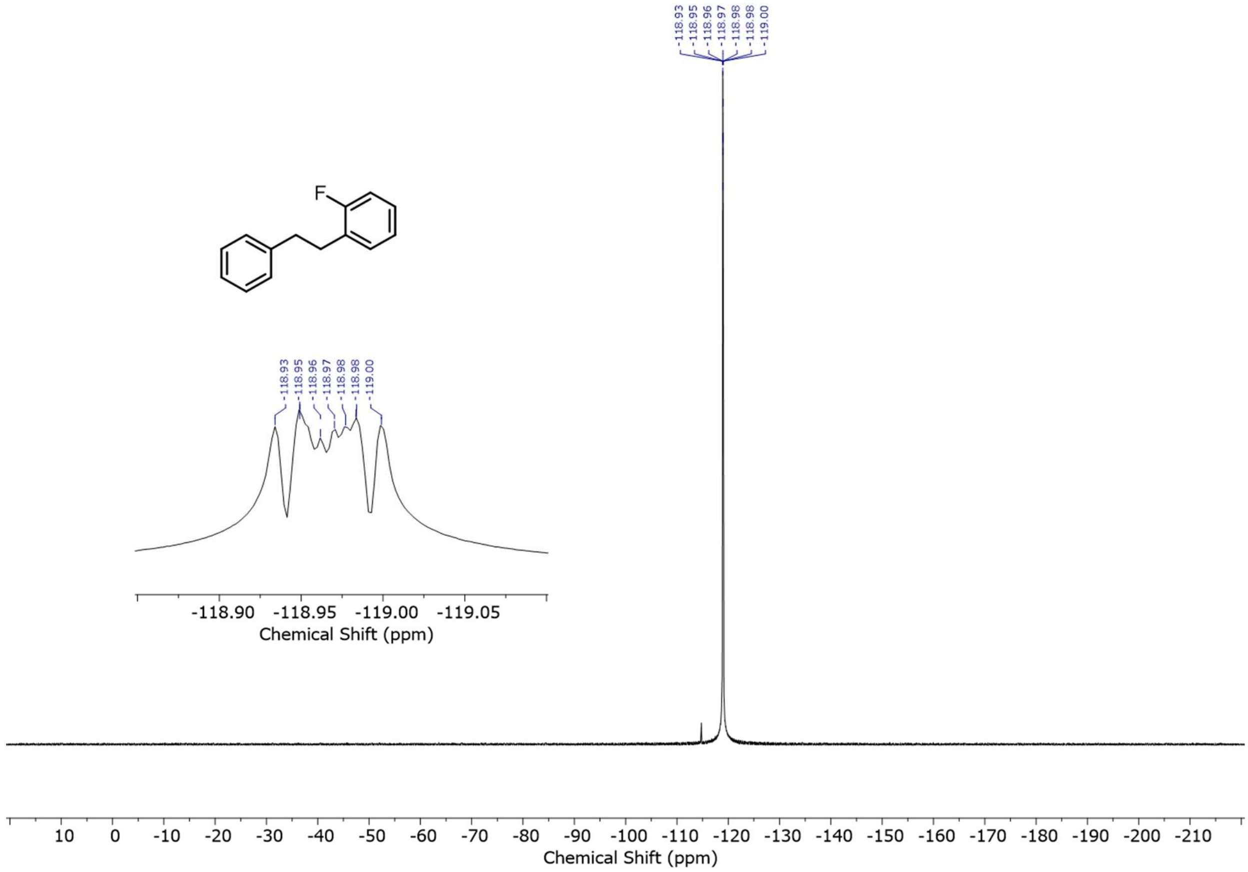

1-(2-(4-Ethyl-1*H*-1,2,3-triazol-1-yl)ethyl)-2,4,6-triphenylpyridin-1-ium Tetrafluoroborate (S-34) ^{1}H NMR (400 MHz, DMSO-*d*$_6$)

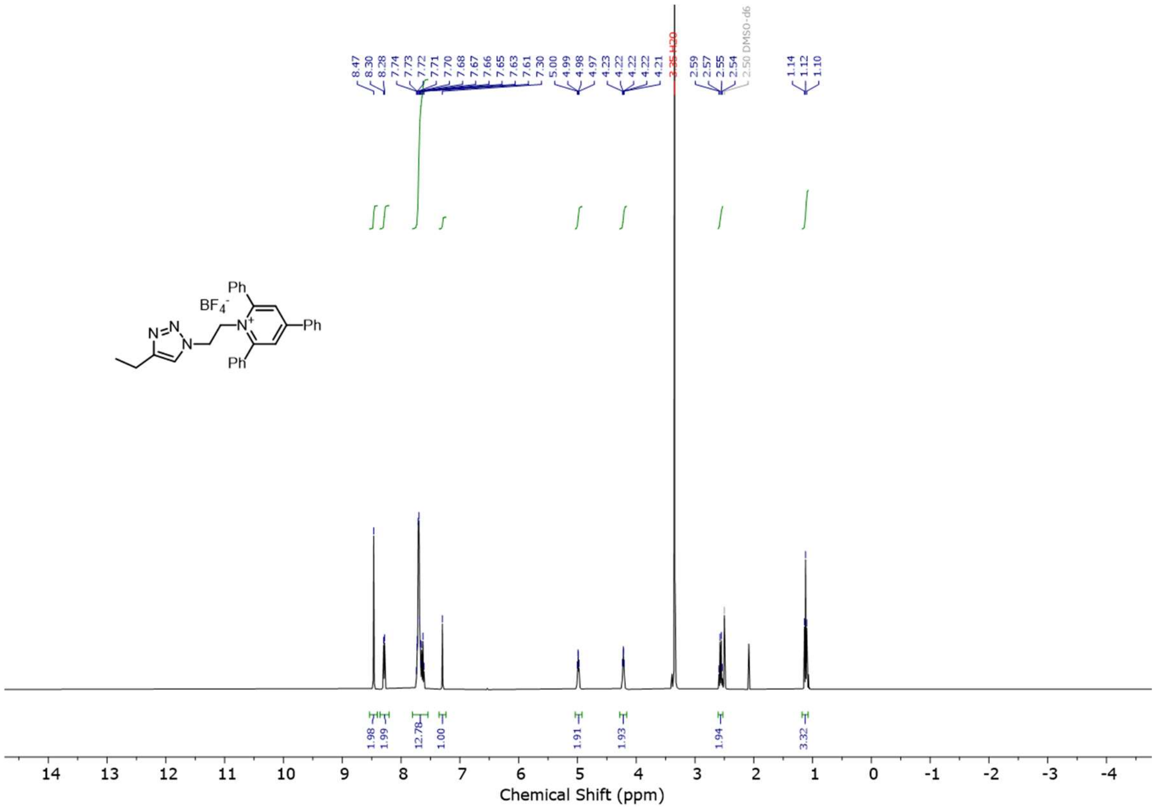

1-(2-(4-Ethyl-1*H*-1,2,3-triazol-1-yl)ethyl)-2,4,6-triphenylpyridin-1-ium Tetrafluoroborate (S-34) ^{13}C NMR (101 MHz, DMSO-*d*$_6$)

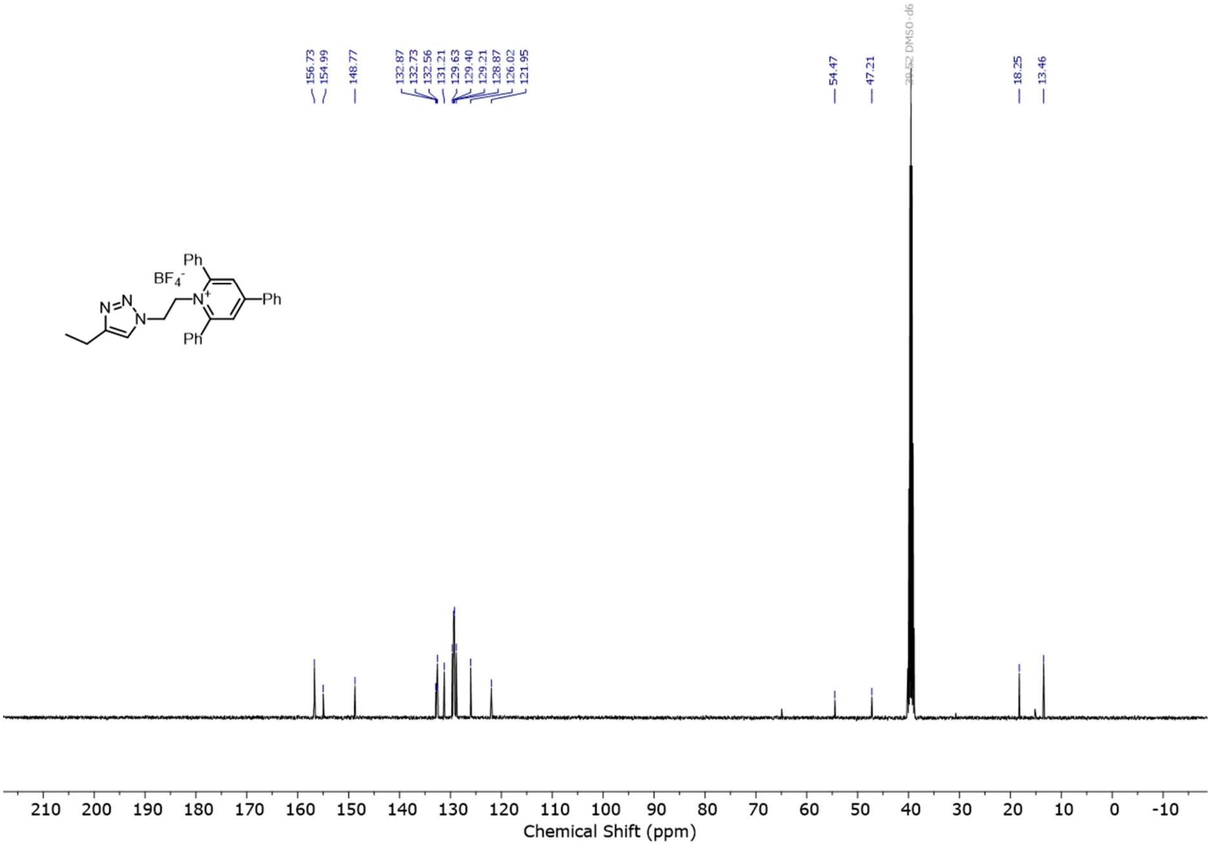

1-(2-(4-Ethyl-1*H*-1,2,3-triazol-1-yl)ethyl)-2,4,6-triphenylpyridin-1-ium Tetrafluoroborate (S-34) ^{19}F NMR (470 MHz, DMSO-d_6)

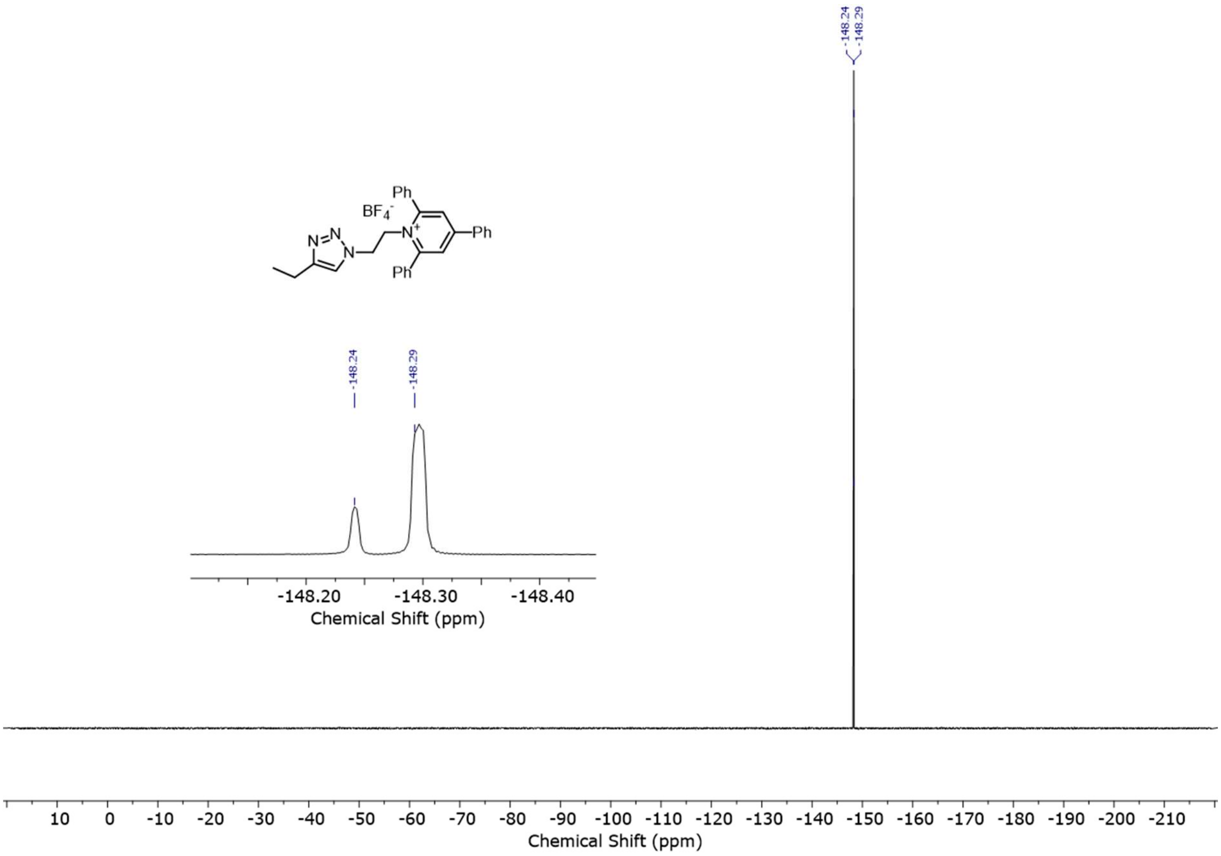

1-((*S*)-6-(*tert*-Butoxy)-5-(3-((*S*)-1,5-di-*tert*-butoxy-1,5-dioxopentan-2-yl)ureido)-6-oxohexyl)-2,4,6-triphenylpyridin-1-ium Tetrafluoroborate (S-35) ^{1}H NMR (500 MHz, DMSO-d_6)

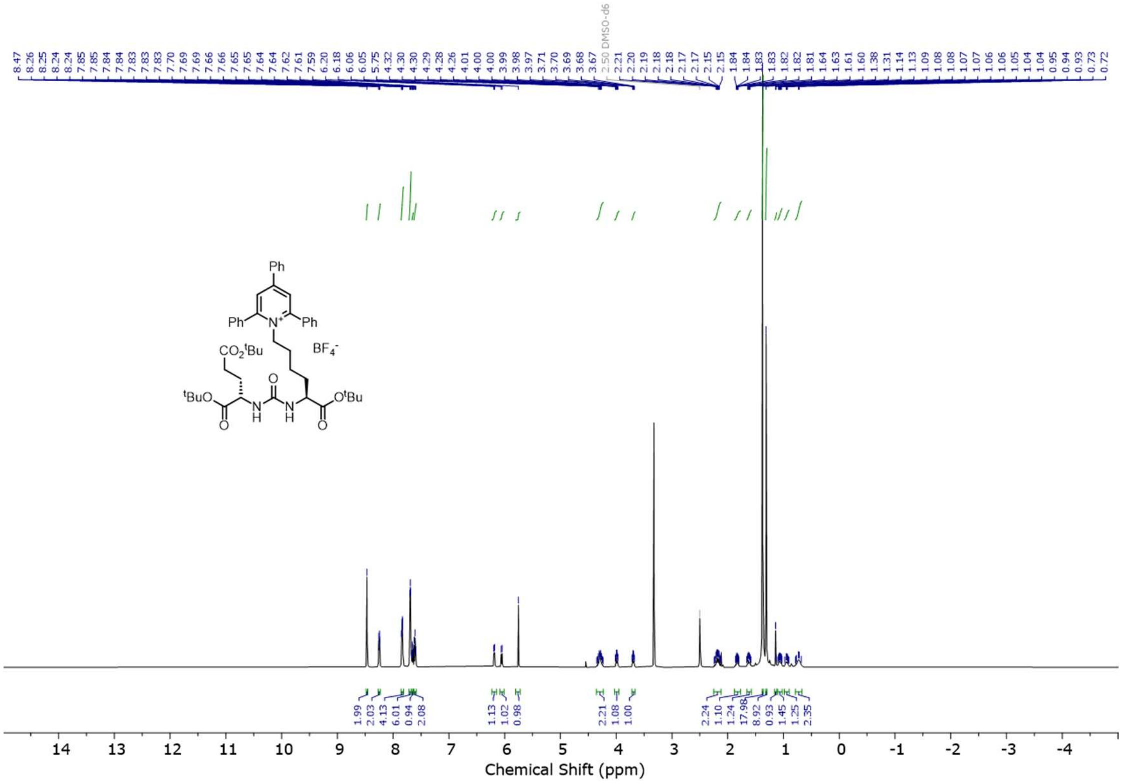

1-((*S*)-6-(*tert*-Butoxy)-5-(3-((*S*)-1,5-di-*tert*-butoxy-1,5-dioxopentan-2-yl)ureido)-6-oxohexyl)-2,4,6-triphenylpyridin-1-ium Tetrafluoroborate (S-35) ^{13}C NMR (126 MHz, DMSO-d_6)

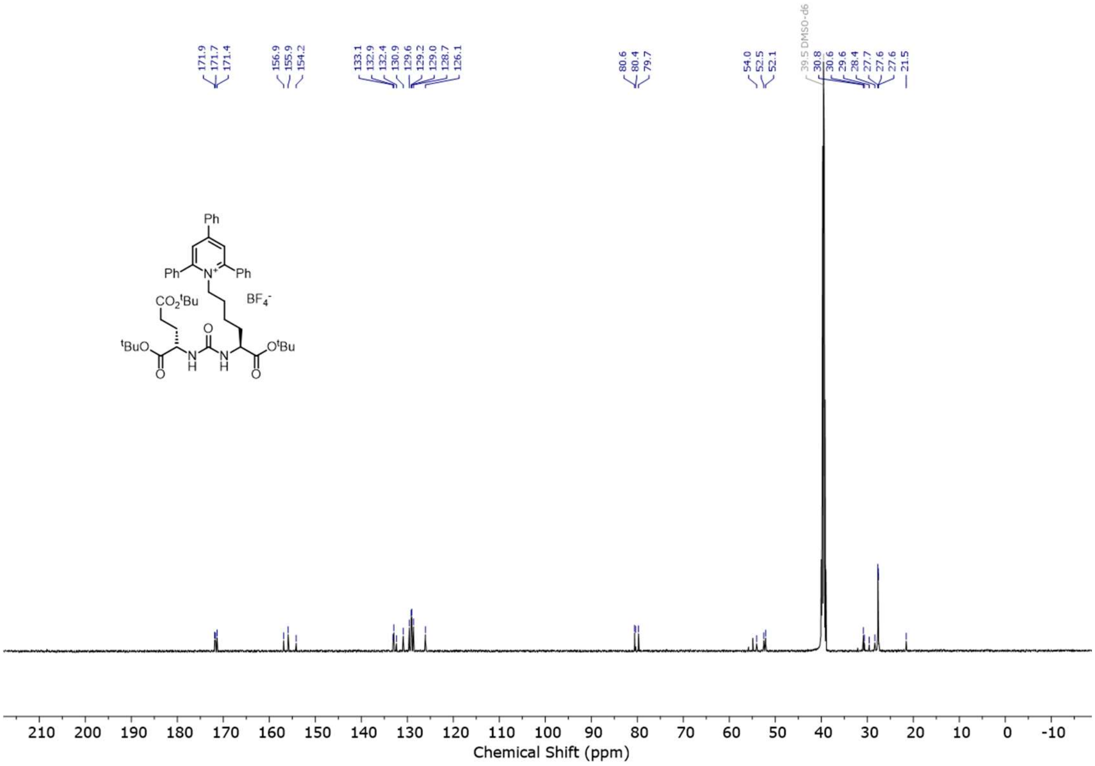

1-((*S*)-6-(*tert*-Butoxy)-5-(3-((*S*)-1,5-di-*tert*-butoxy-1,5-dioxopentan-2-yl)ureido)-6-oxohexyl)-2,4,6-triphenylpyridin-1-ium tetrafluoroborate (S-35) ^{19}F NMR (470 MHz, DMSO-d_6)

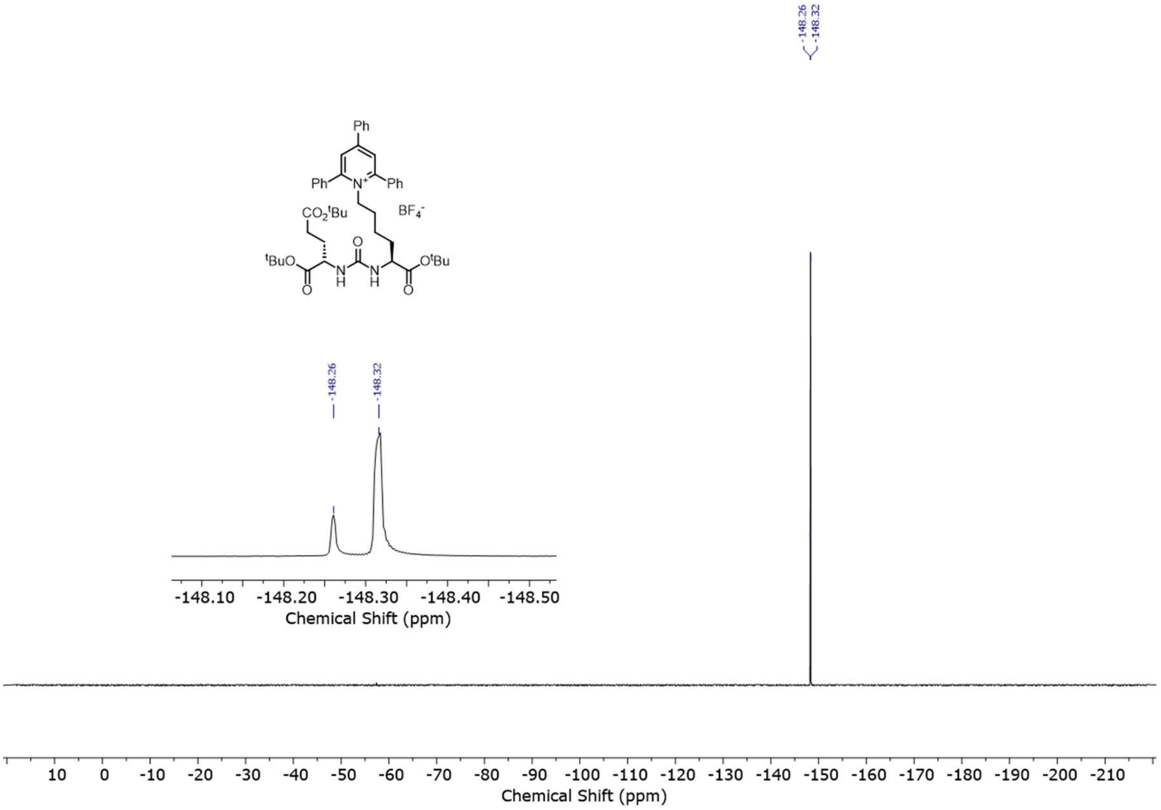

2,4,6-Triphenyl-1-(piperidin-2-ylmethyl)pyridin-1-ium Tetrafluoroborate (S-36) ^{1}H NMR (400 MHz, CD_3CN)

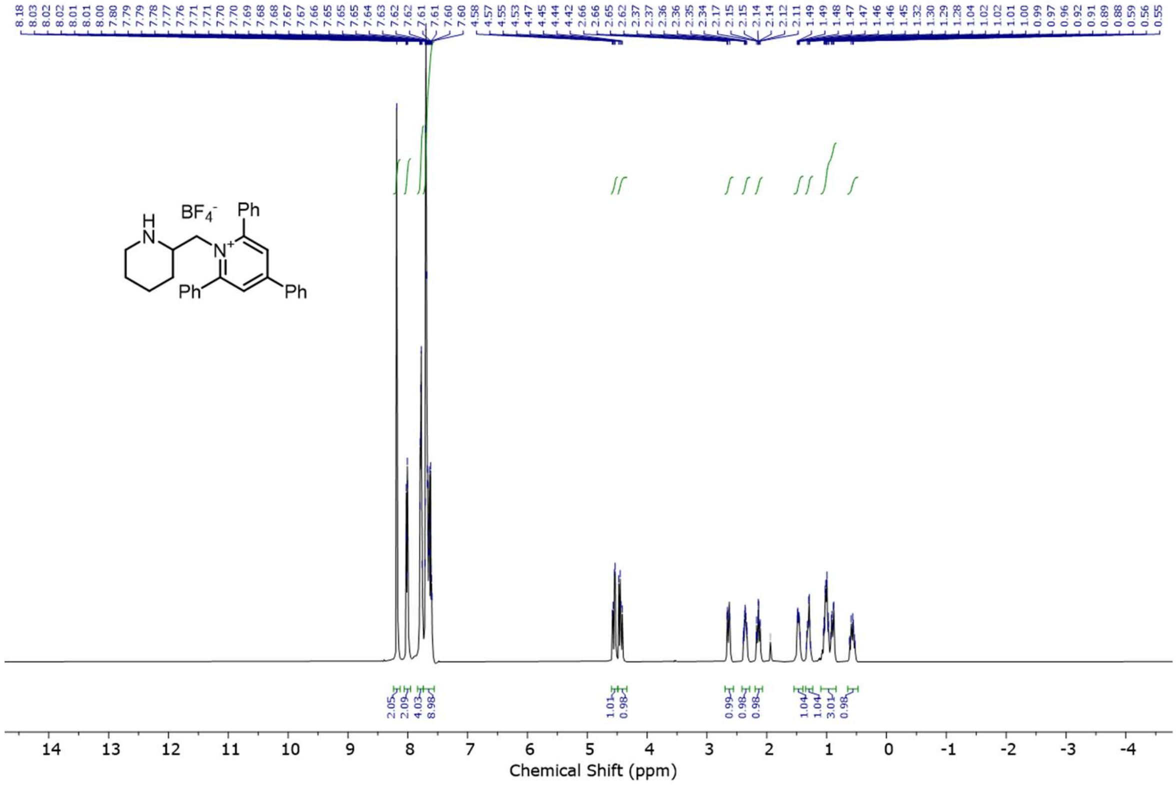

2,4,6-Triphenyl-1-(piperidin-2-ylmethyl)pyridin-1-ium Tetrafluoroborate (S-36) ^{13}C NMR (101 MHz, CD_3CN)

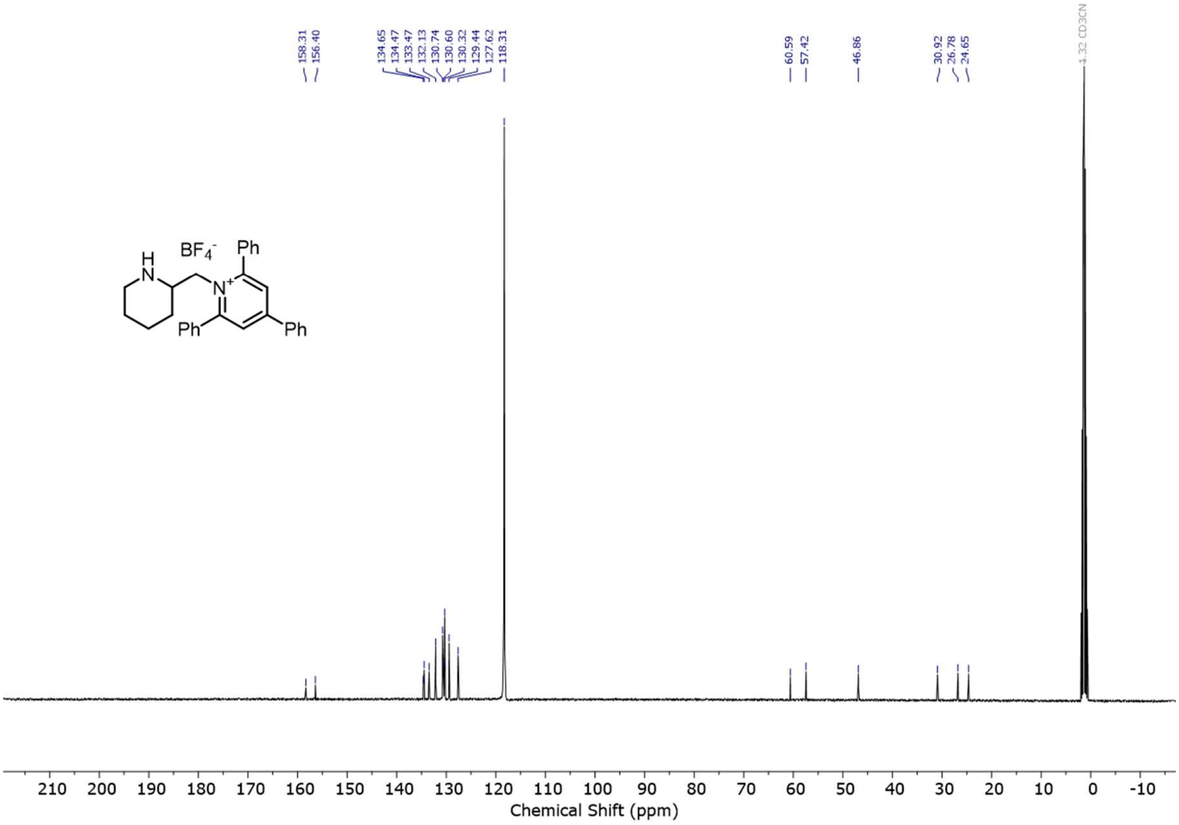

2,4,6-Triphenyl-1-(piperidin-2-ylmethyl)pyridin-1-ium Tetrafluoroborate (S-36) ^{19}F NMR (376 MHz, CD_3CN)

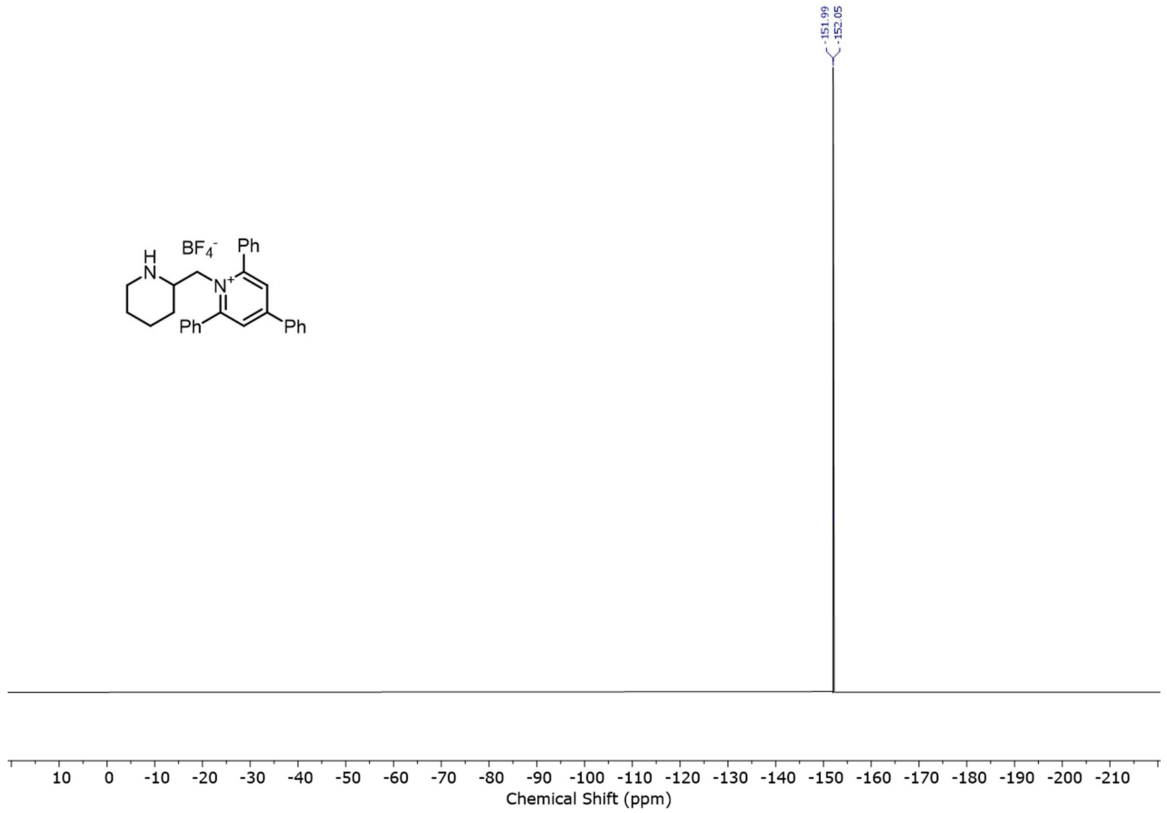

1-(2-(1-Hydroxycyclohexyl)-2-(4-methoxyphenyl)ethyl)-2,4,6-triphenylpyridin-1-ium Tetrafluoroborate (S-37) ^{1}H NMR (600 MHz, DMSO-d_6, T = 373K)

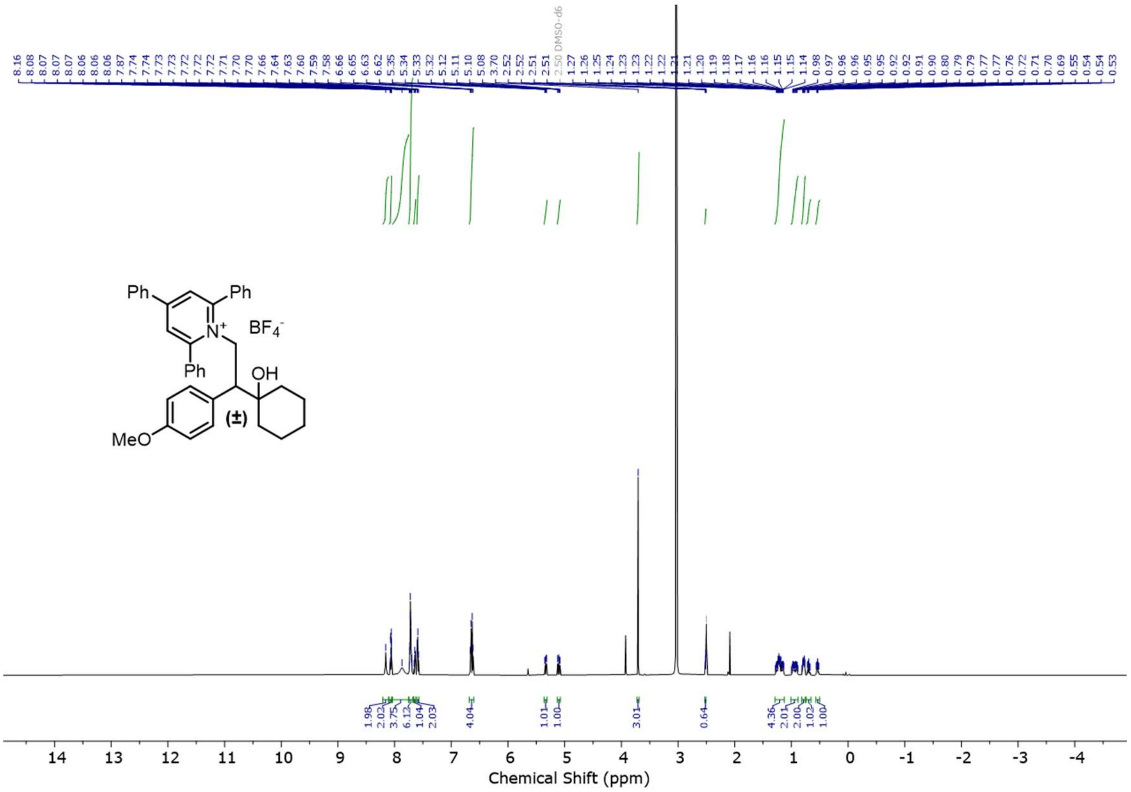

1-(2-(1-Hydroxycyclohexyl)-2-(4-methoxyphenyl)ethyl)-2,4,6-triphenylpyridin-1-ium Tetrafluoroborate (S-37) ^{13}C NMR (151 MHz, DMSO-d_6, T = 373K)

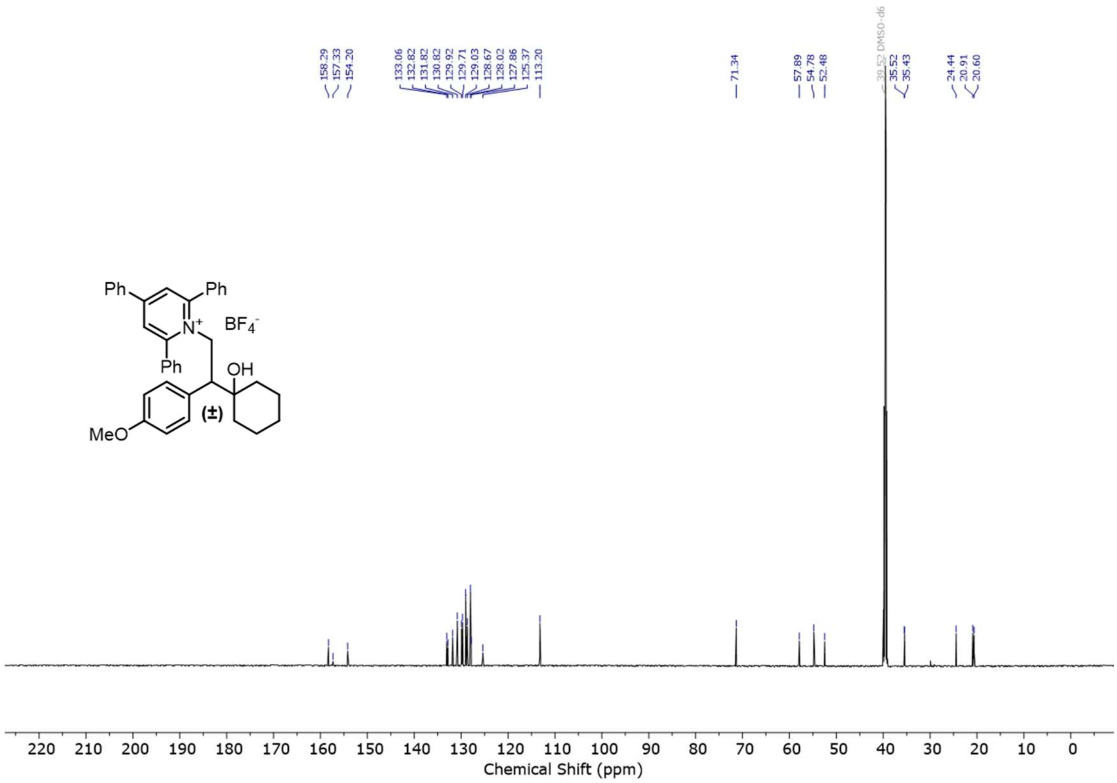

1-(2-(1-Hydroxycyclohexyl)-2-(4-methoxyphenyl)ethyl)-2,4,6-triphenylpyridin-1-ium Tetrafluoroborate (S-37) ^{19}F NMR (376 MHz, DMSO-d_6)

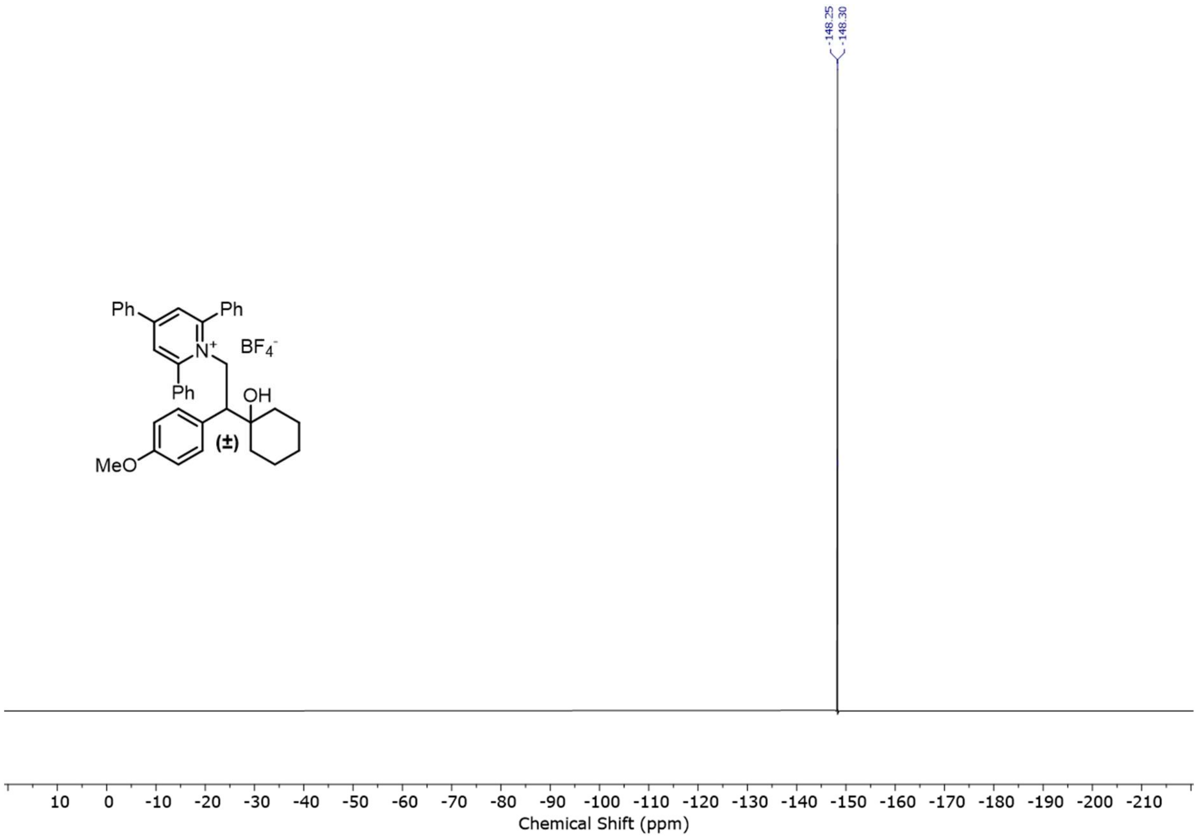

2,4,6-Triphenyl-1-(2-(pyrrolidin-1-yl)ethyl)pyridin-1-ium Tetrafluoroborate (S-38) ^{1}H NMR (400 MHz, CD_3CN)

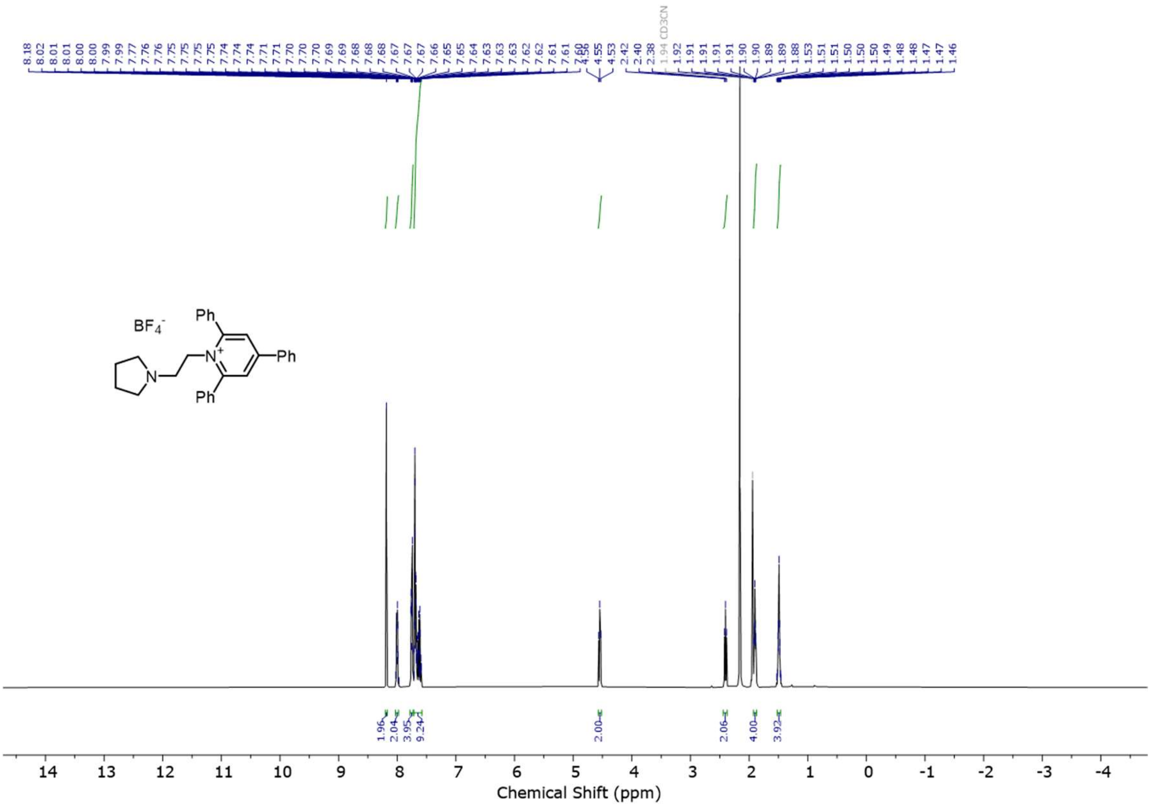

2,4,6-Triphenyl-1-(2-(pyrrolidin-1-yl)ethyl)pyridin-1-ium Tetrafluoroborate (S-38) ^{13}C NMR (101 MHz, CD_3CN)

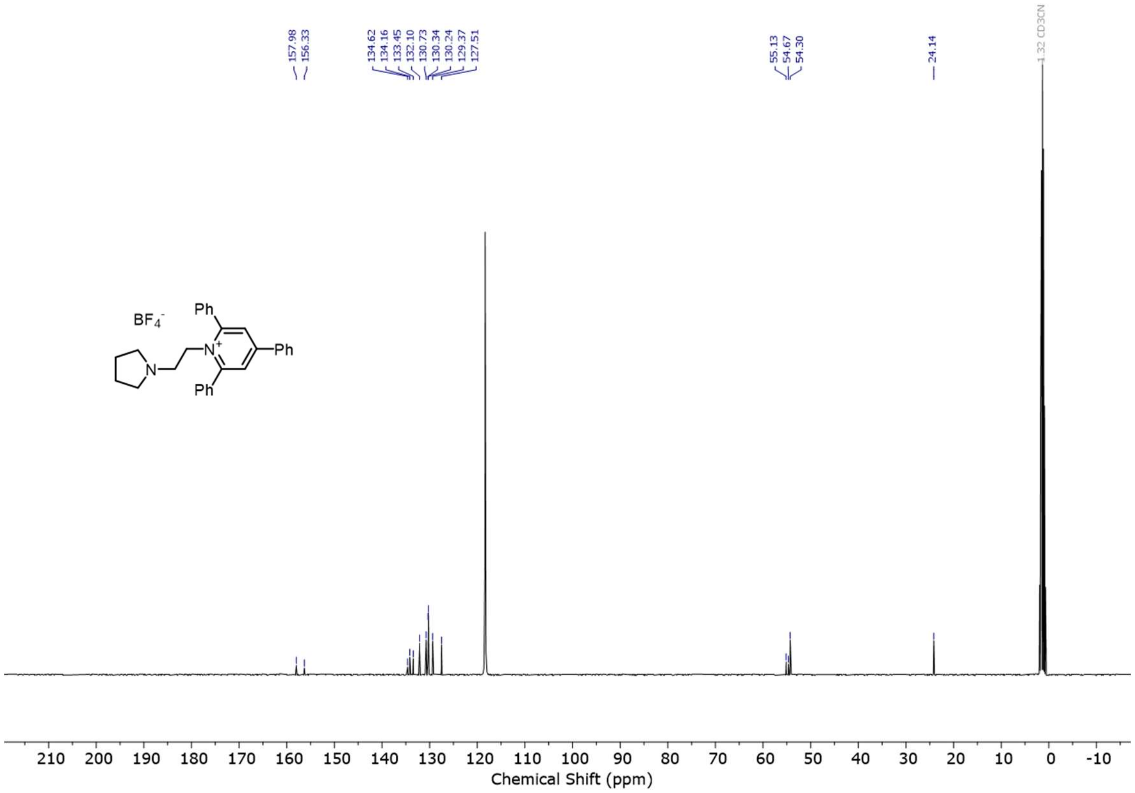

2,4,6-Triphenyl-1-(2-(pyrrolidin-1-yl)ethyl)pyridin-1-ium Tetrafluoroborate (S-38) ^{19}F NMR (376 MHz, CD_3CN)

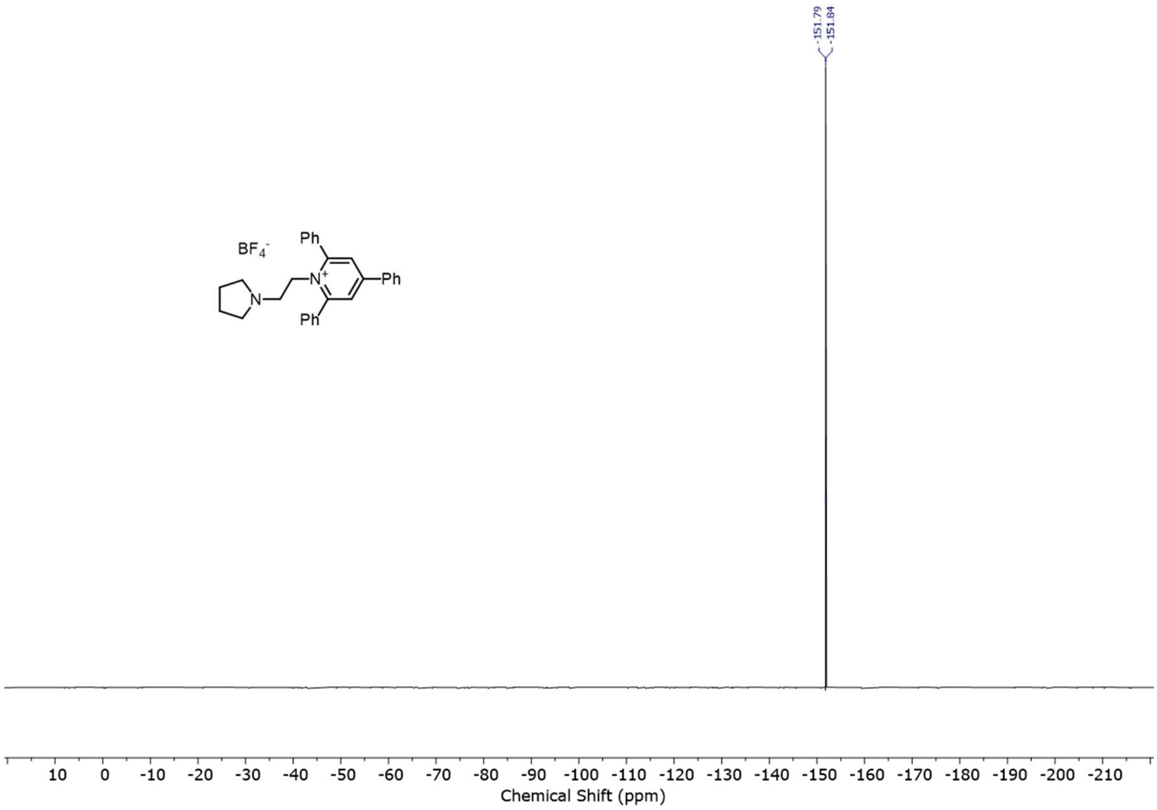

1-(2-((4,6-Dimethoxypyrimidin-2-yl)amino)ethyl)-2,4,6-triphenylpyridin-1-ium tetrafluoroborate (S-40) ^{1}H NMR (400 MHz, $CDCl_3$ + 0.03% TMS)

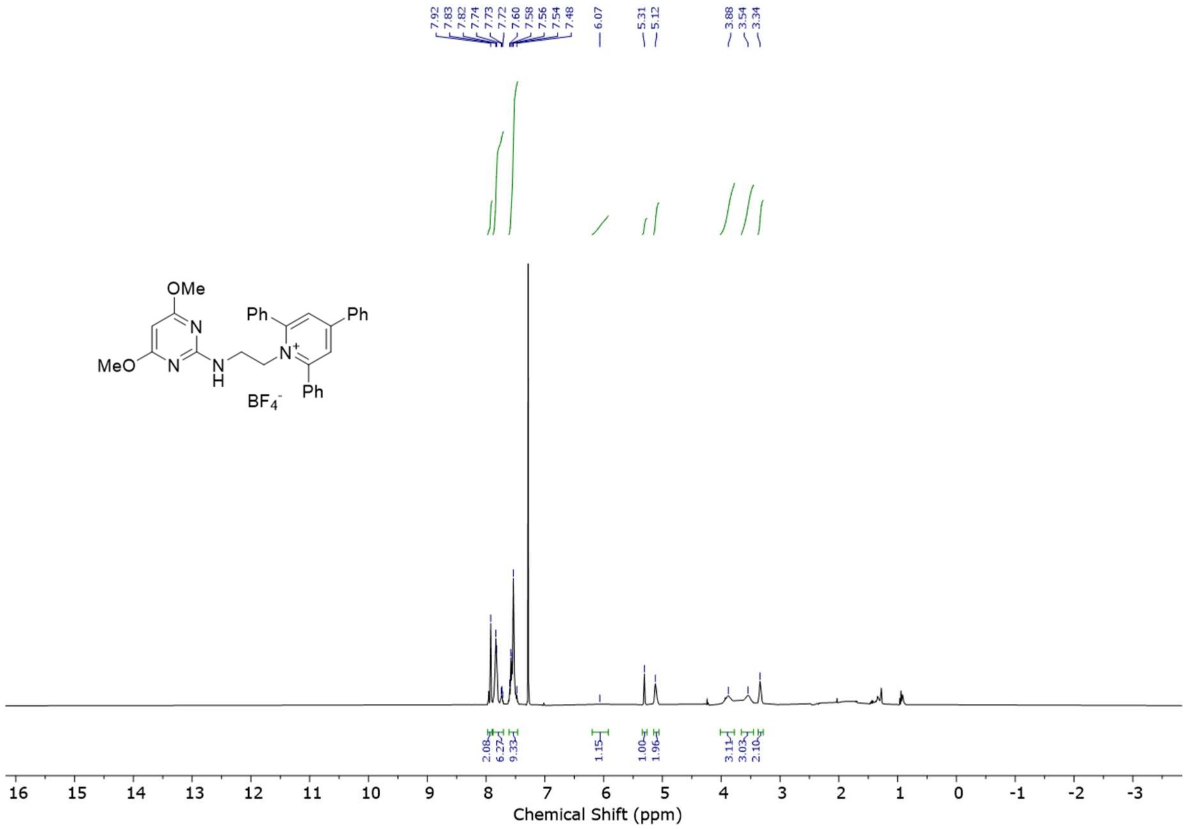

1-(2-((4,6-Dimethoxypyrimidin-2-yl)amino)ethyl)-2,4,6-triphenylpyridin-1-ium tetrafluoroborate (S-40) ^{13}C NMR (101 MHz, $CDCl_3$)

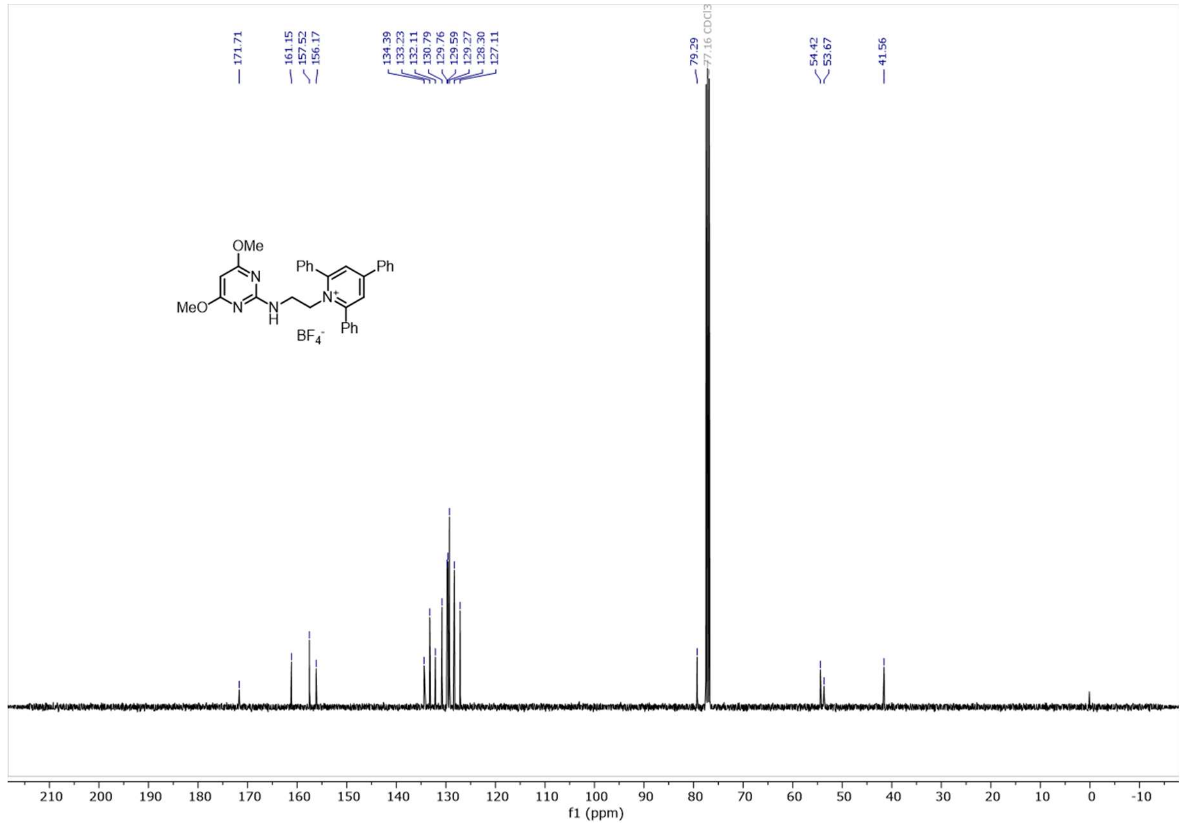

1-(2-((4,6-Dimethoxypyrimidin-2-yl)amino)ethyl)-2,4,6-triphenylpyridin-1-ium Tetrafluoroborate (S-40) ^{19}F NMR (377 MHz, $CDCl_3$)

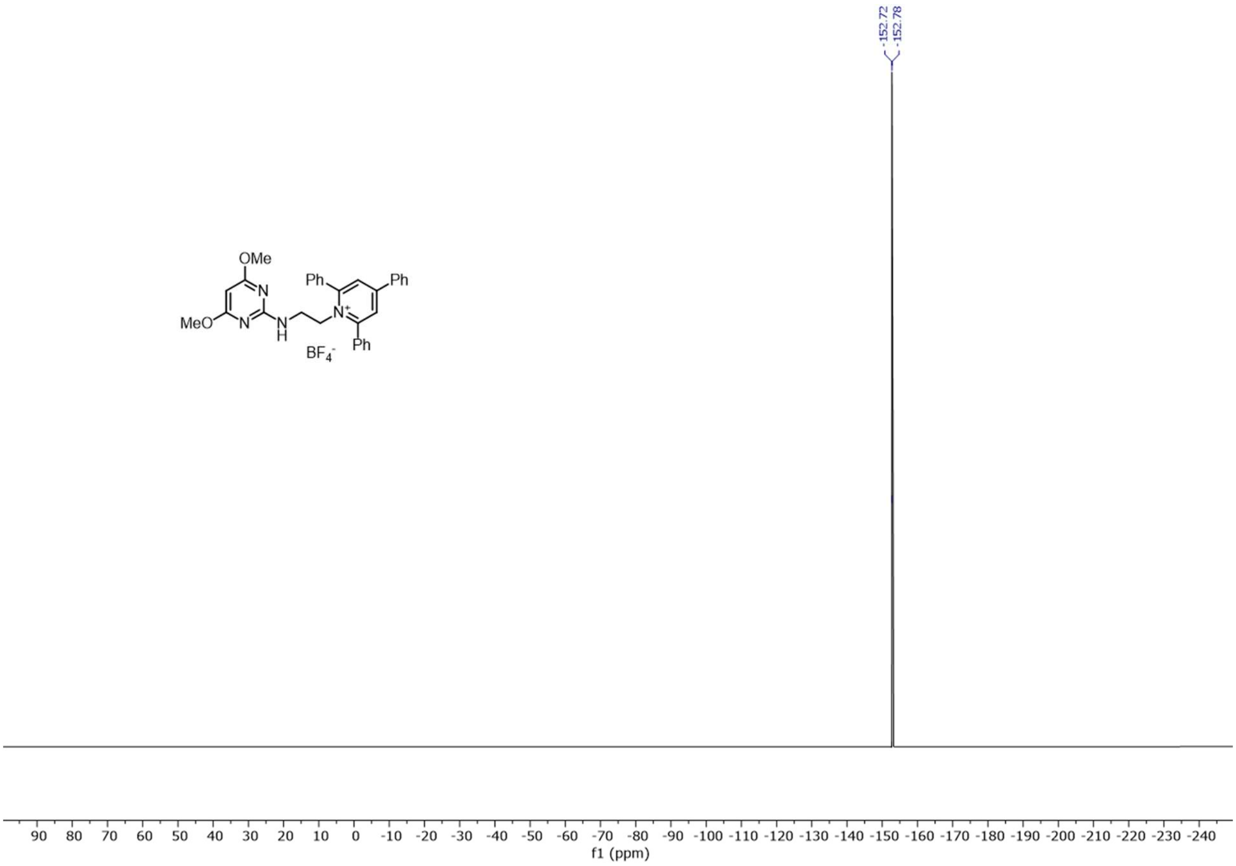

2,4,6-Triphenyl-1-(1-(pyrimidin-2-yl)piperidin-4-yl)pyridin-1-ium tetrafluoroborate (S-42) ¹H NMR (500 MHz, $CDCl_3$)

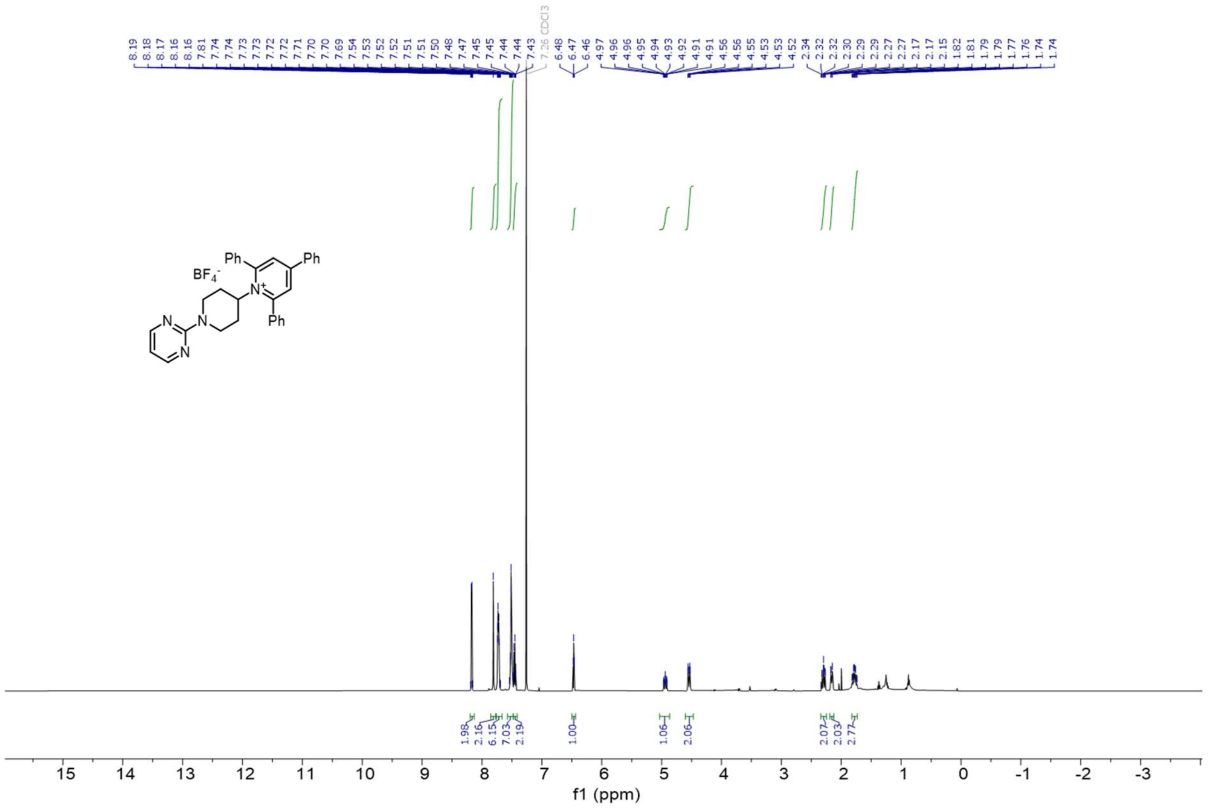

2,4,6-Triphenyl-1-(1-(pyrimidin-2-yl)piperidin-4-yl)pyridin-1-ium tetrafluoroborate (S-42) ¹³C NMR (126 MHz, $CDCl_3$)

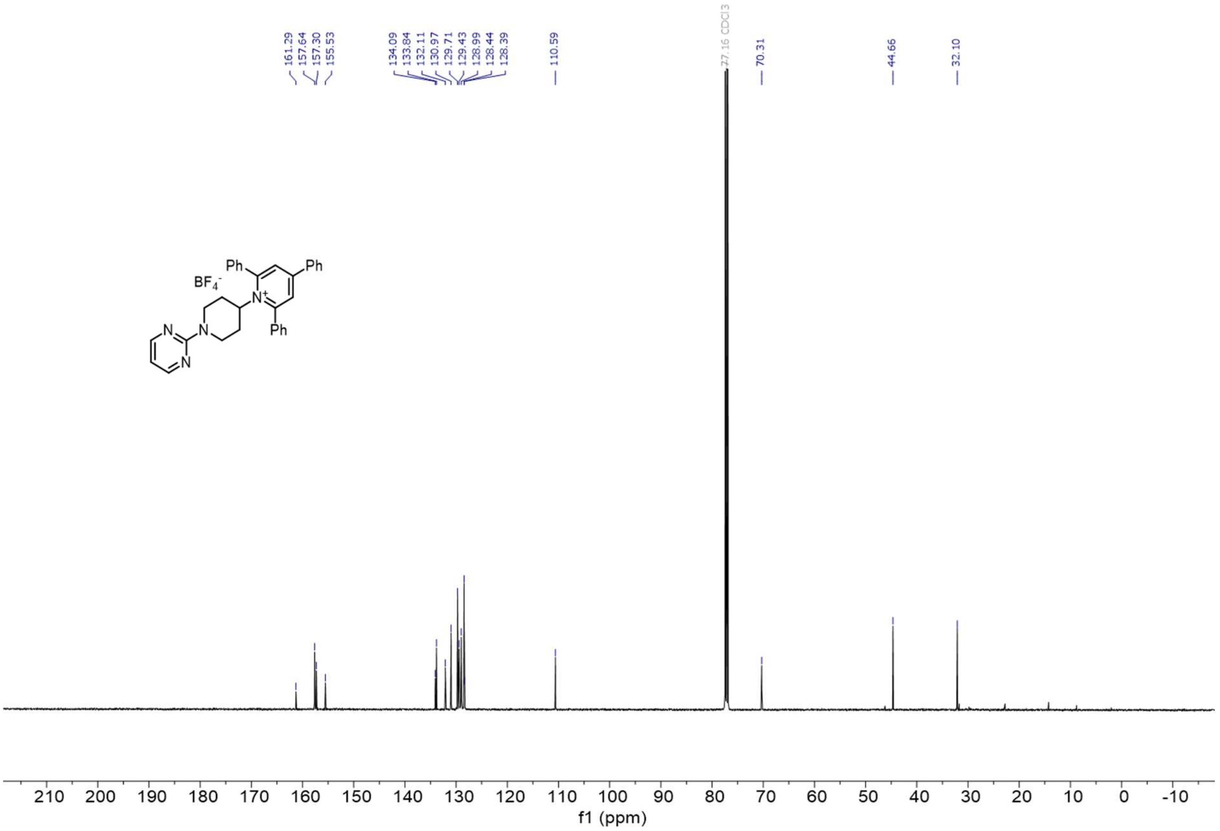

2,4,6-Triphenyl-1-(1-(pyrimidin-2-yl)piperidin-4-yl)pyridin-1-ium tetrafluoroborate (S-42) ^{19}F NMR (470 MHz, $CDCl_3$)

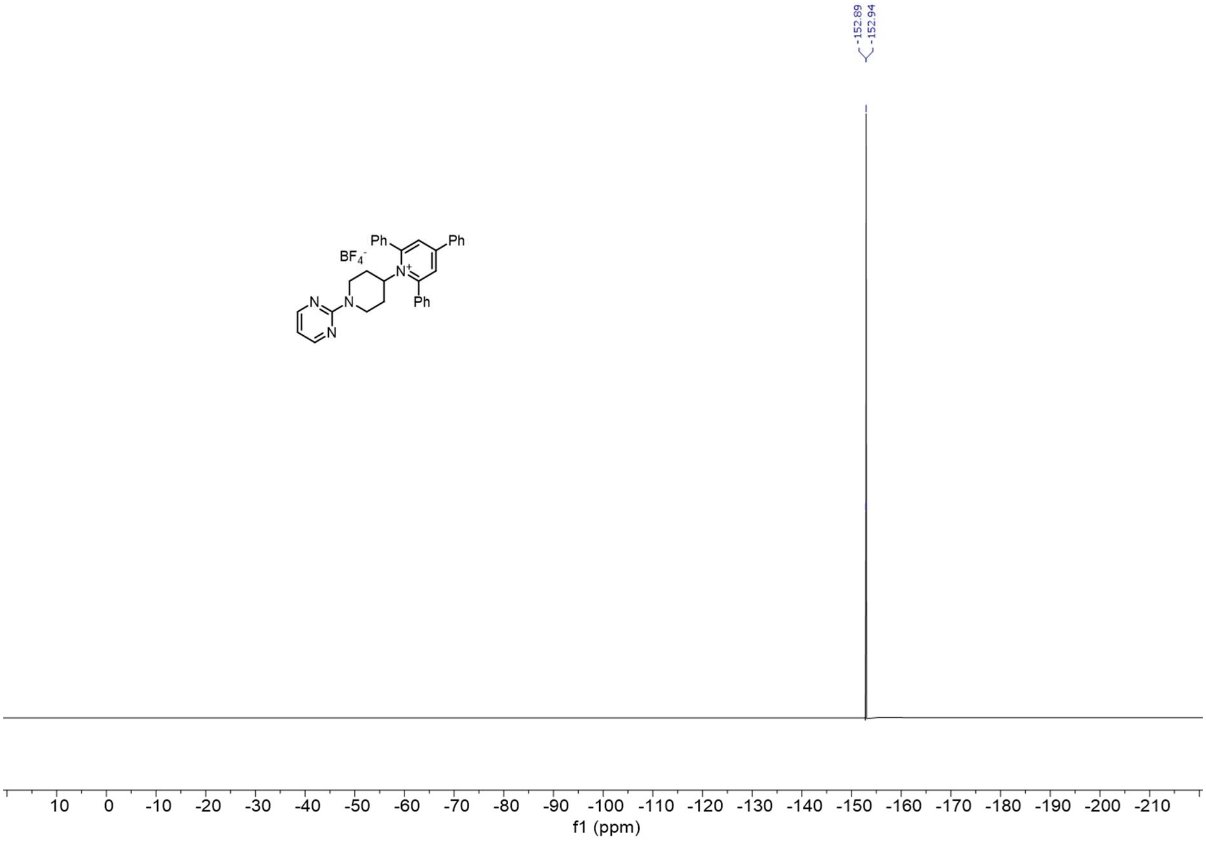

tert-Butyl (2-(4-(3-cyano-_1H_-indole-7-carbonyl)piperazin-1-yl)ethyl)carbamate (S-44) ^{1}H NMR (500 MHz, $CDCl_3$)

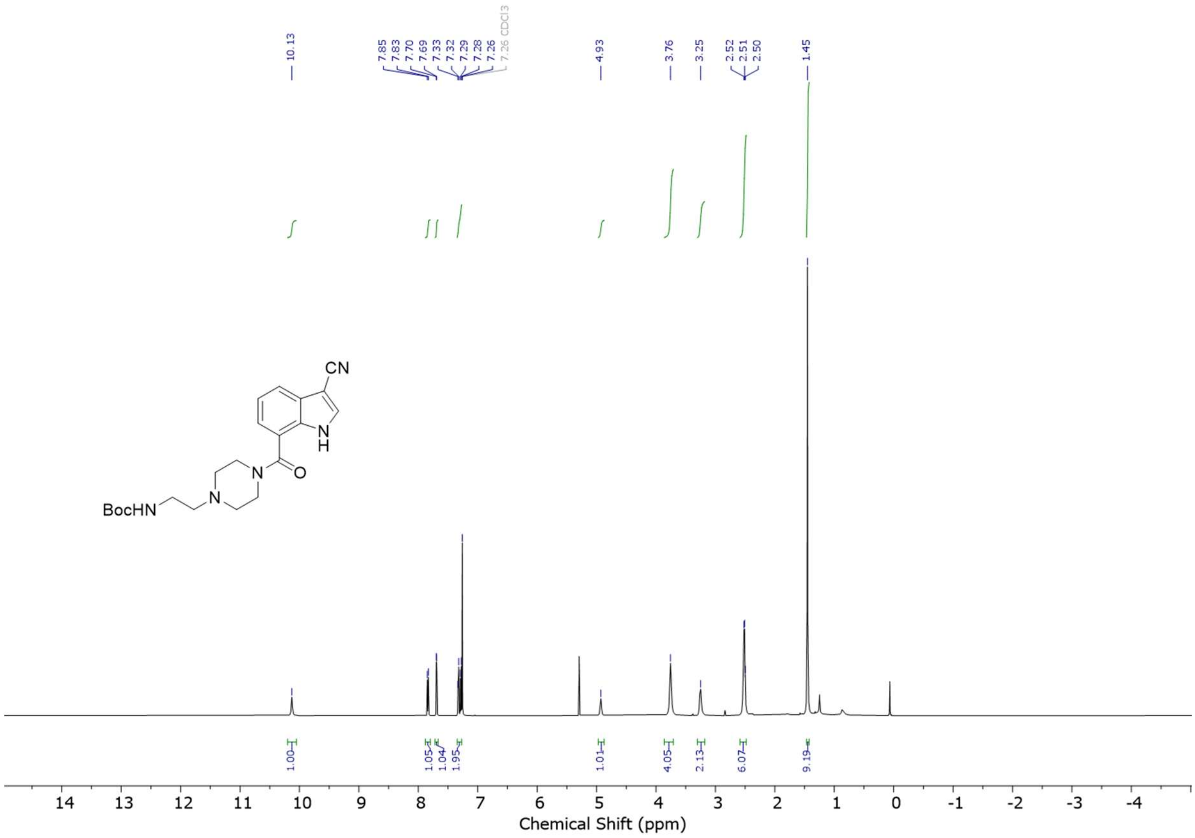

tert-Butyl (2-(4-(3-cyano-_1H_-indole-7-carbonyl)piperazin-1-yl)ethyl)carbamate (S-44) ^{13}C NMR (126 MHz, $CDCl_3$)

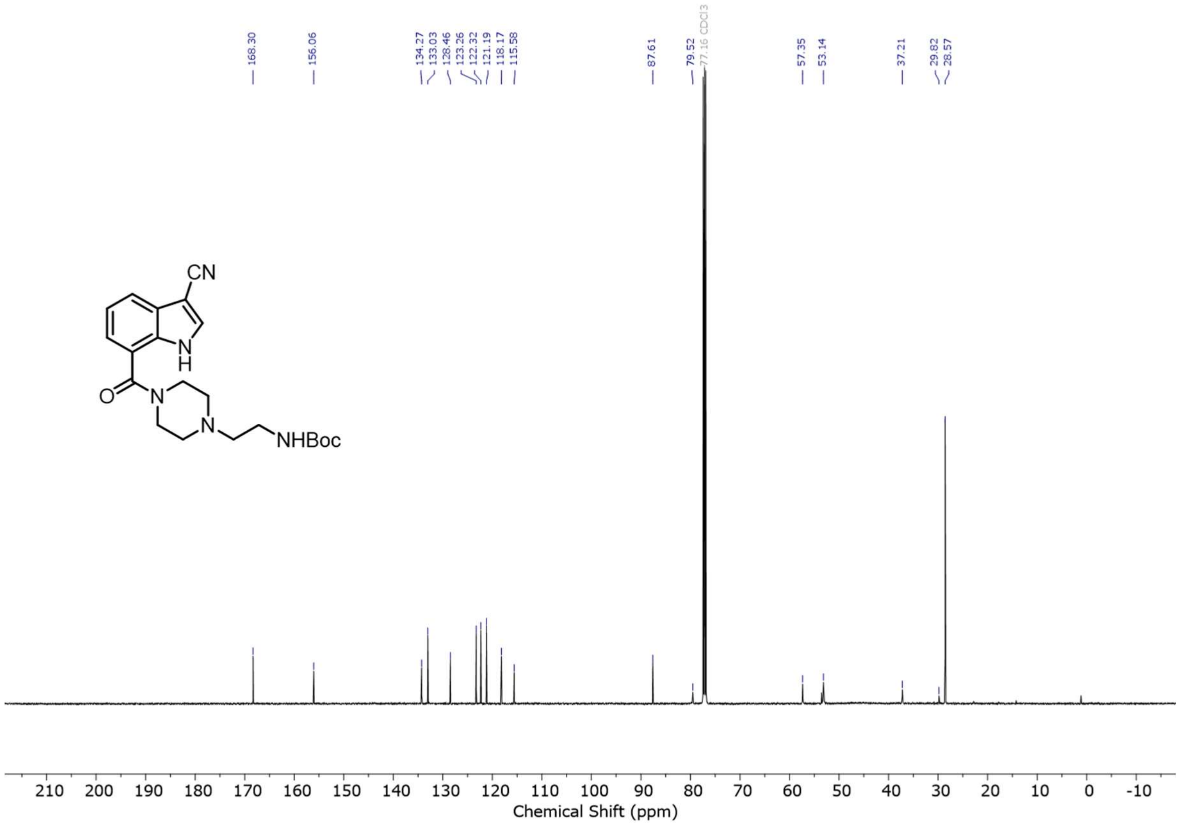

1-(2-(4-(3-Cyano-1*H*-indole-7-carbonyl)piperazin-1-yl)ethyl)-2,4,6-triphenylpyridin-1-ium Tetrafluoroborate (S-45) ^{1}H NMR (600 MHz, DMSO)

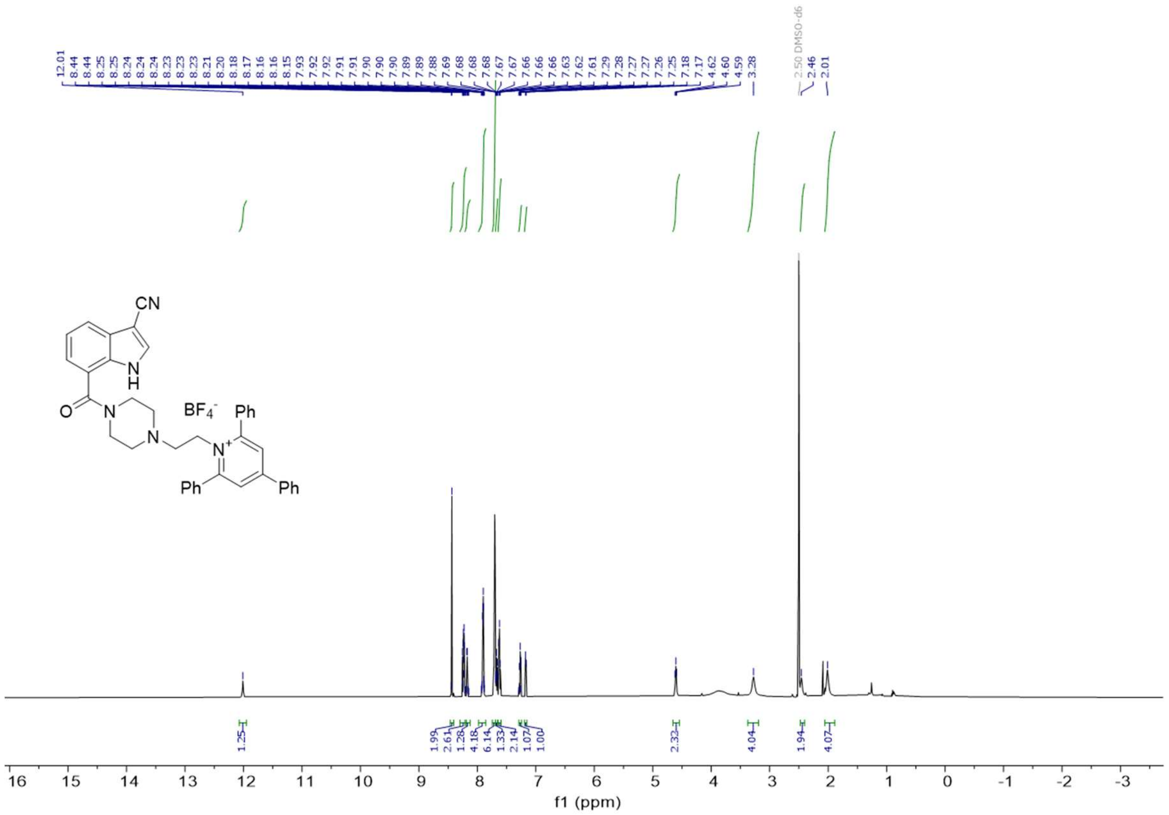

1-(2-(4-(3-Cyano-1*H*-indole-7-carbonyl)piperazin-1-yl)ethyl)-2,4,6-triphenylpyridin-1-ium Tetrafluoroborate (S-45) ^{13}C NMR (151 MHz, DMSO, T = 353K)

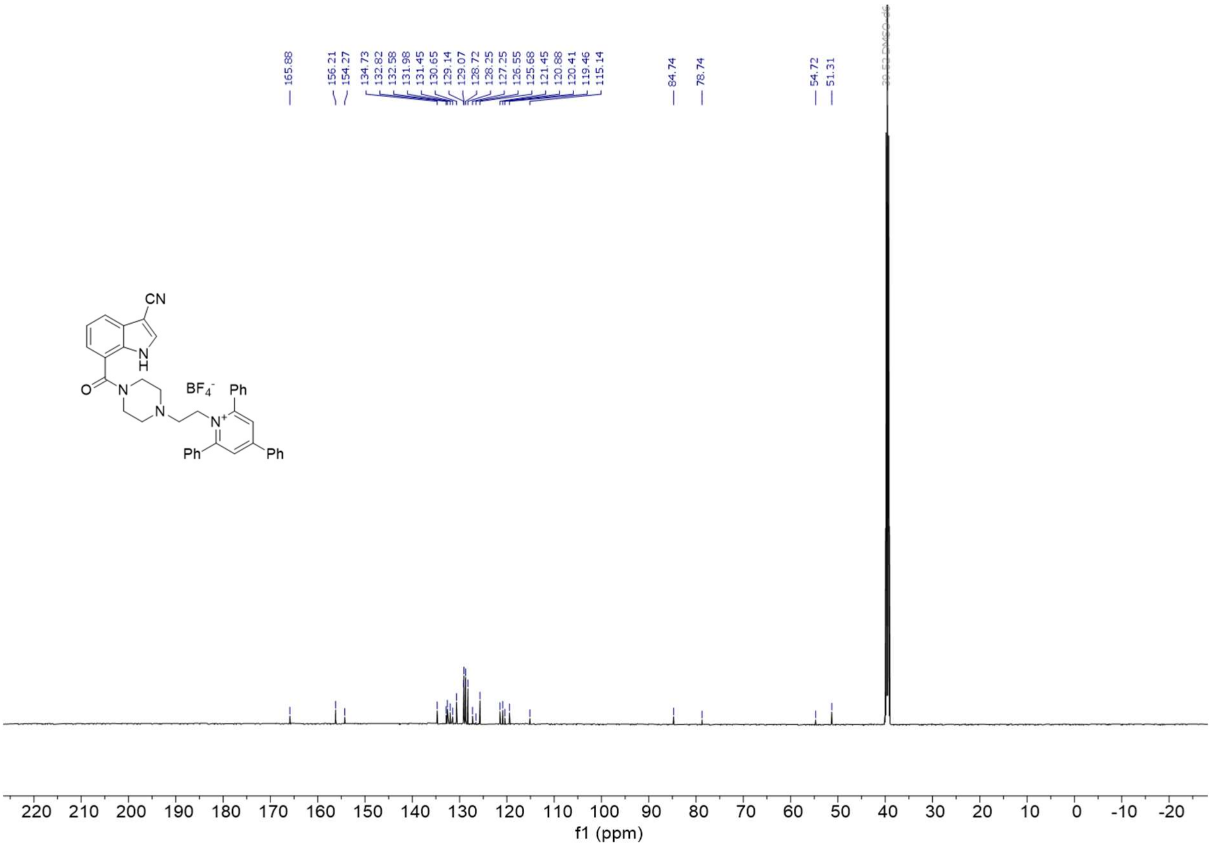

1-(2-(4-(3-Cyano-1*H*-indole-7-carbonyl)piperazin-1-yl)ethyl)-2,4,6-triphenylpyridin-1-ium Tetrafluoroborate (S-45) ^{19}F NMR (471 MHz, $CDCl_3$)

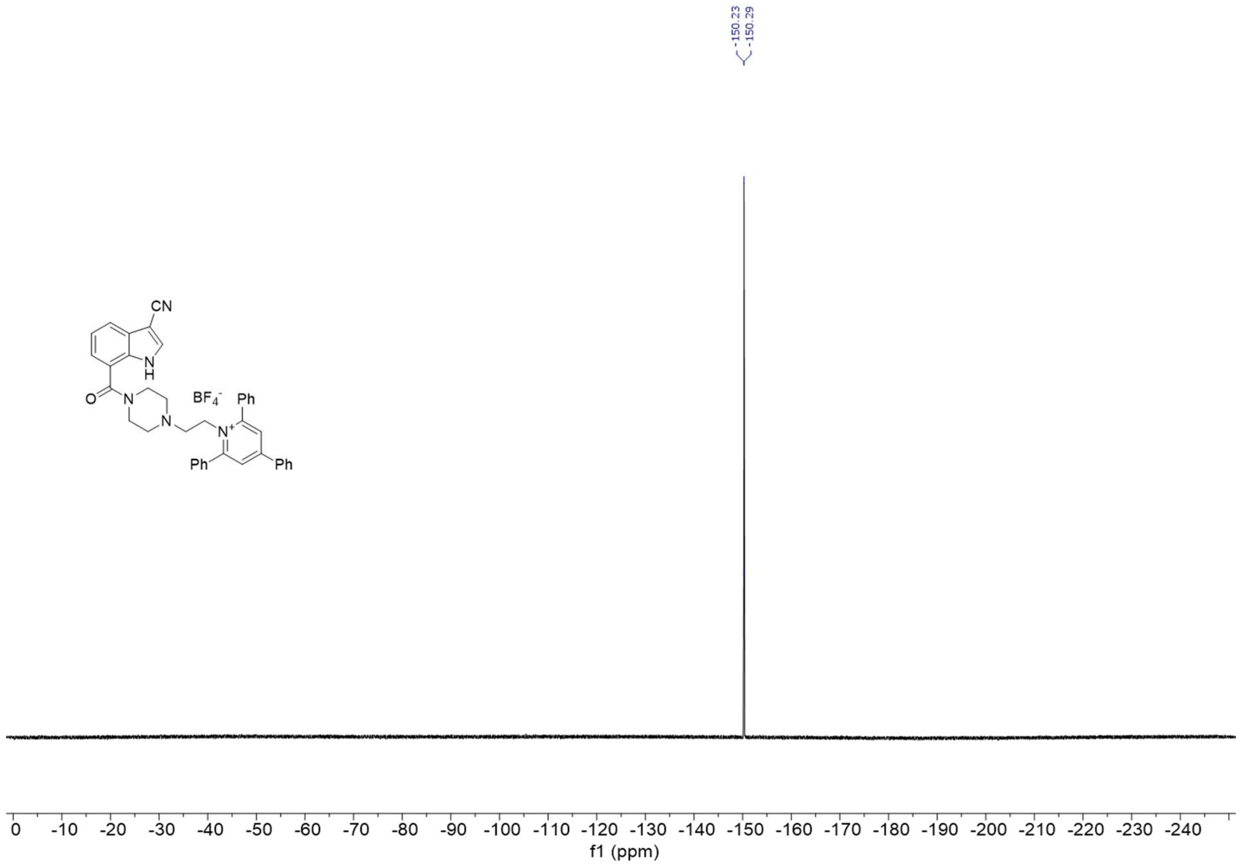

***tert*-Butyl (3-(5-(3-chloro-4-methoxyphenyl)oxazole-4-carboxamido)propyl)carbamate (S-47) ^{1}H NMR (400 MHz, $CDCl_3$)**

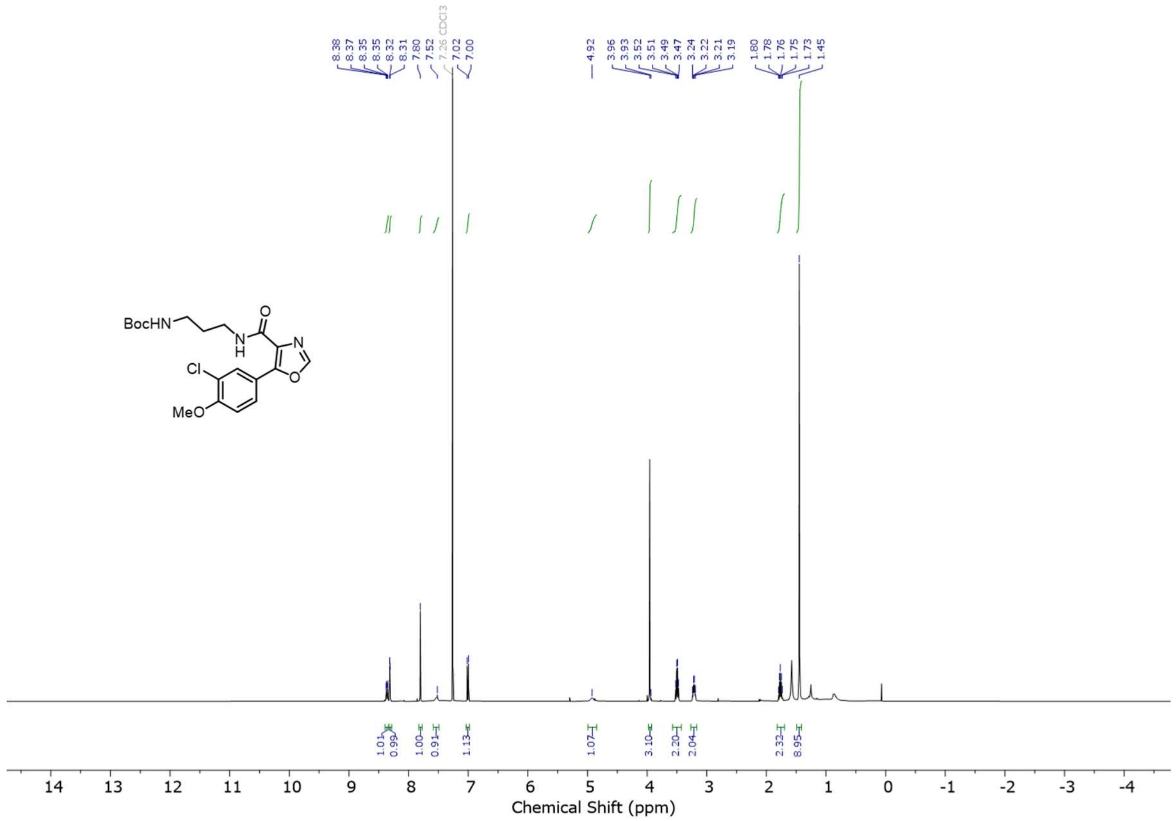

***tert*-Butyl (3-(5-(3-chloro-4-methoxyphenyl)oxazole-4-carboxamido)propyl)carbamate (S-47) ^{13}C NMR (101 MHz, $CDCl_3$)**

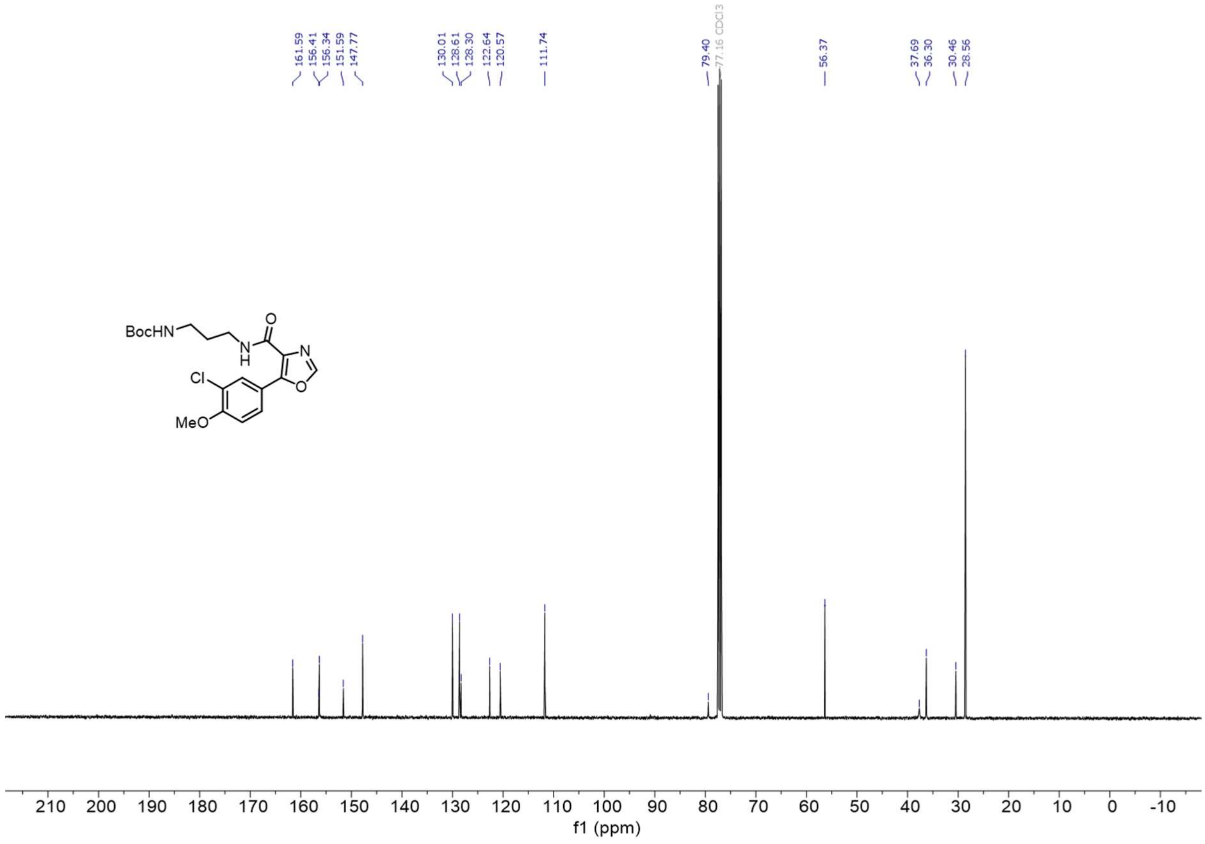

***tert*-Butyl (3-(7-hydroxy-2-oxo-*2H*-chromene-3-carboxamido)propyl)carbamate (S-48)**
^{1}H NMR (400 MHz, DMSO-d_6)

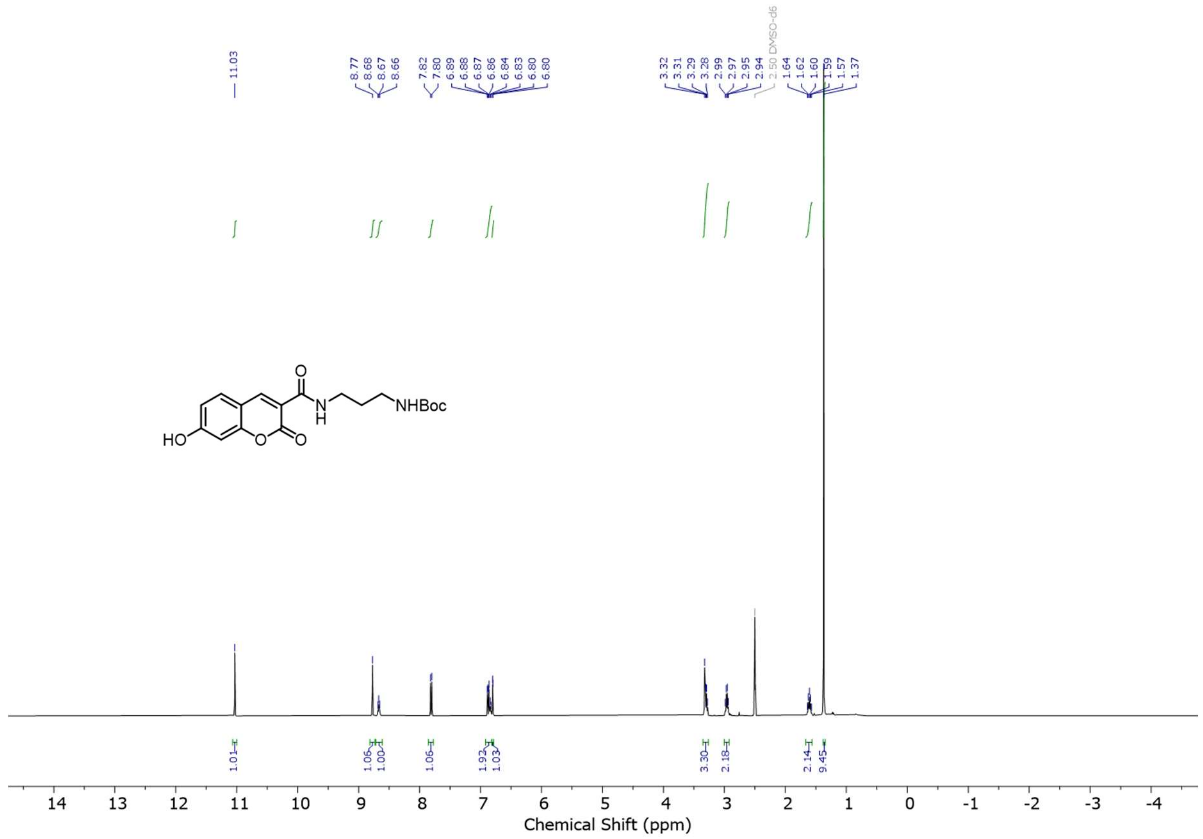

***tert*-Butyl (3-(7-hydroxy-2-oxo-*2H*-chromene-3-carboxamido)propyl)carbamate (S-48)**
^{13}C NMR (101 MHz, DMSO-d_6)

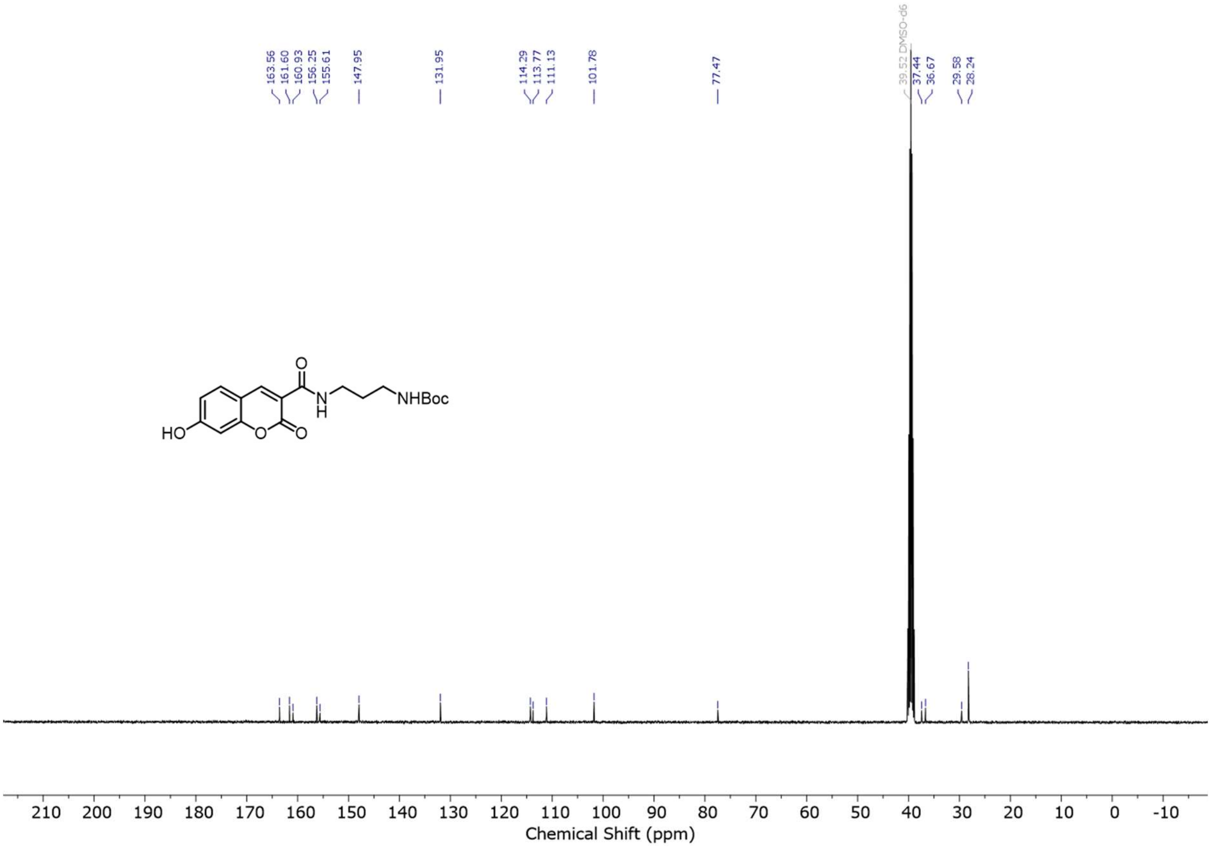

1-(3-(7-Hydroxy-2-oxo-*2H*-chromene-3-carboxamido)propyl)-2,4,6-triphenylpyridin-1-ium Tetrafluoroborate (S-49) ^{1}H NMR (600 MHz, DMSO-d_6)

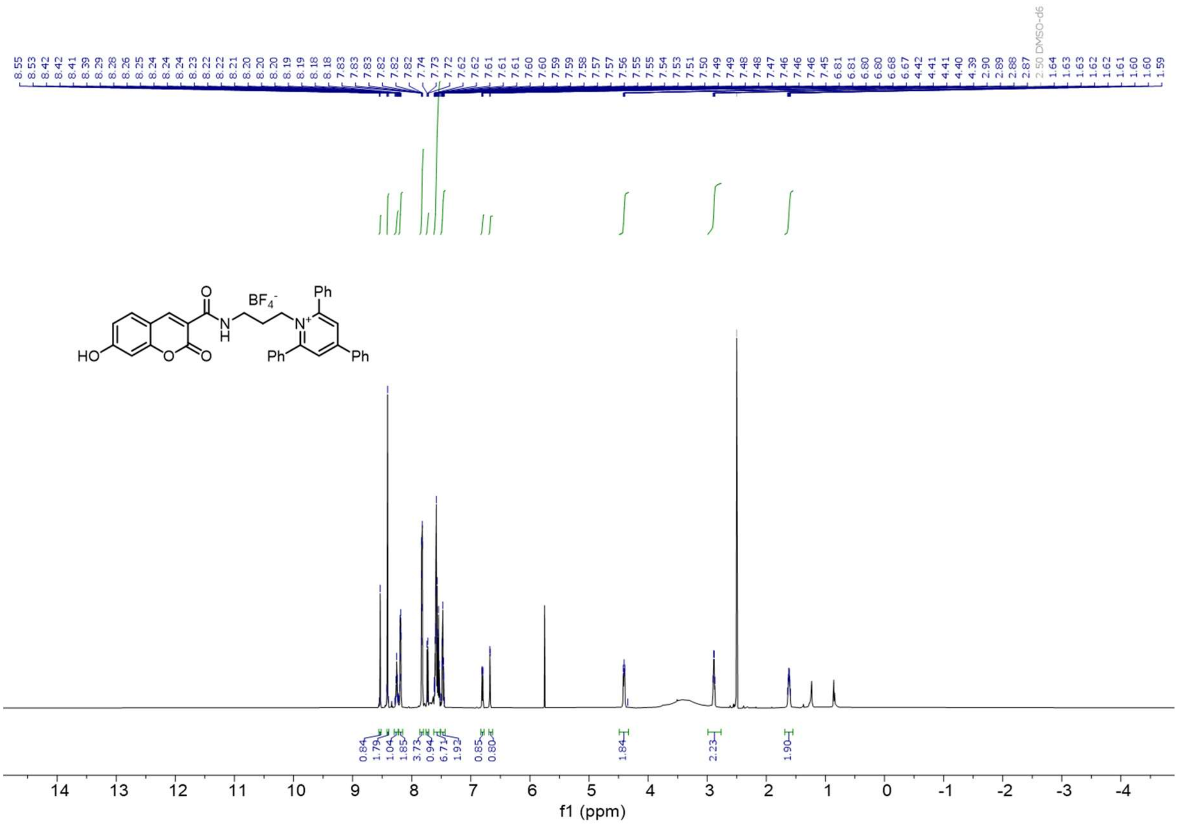

1-(3-(7-Hydroxy-2-oxo-*2H*-chromene-3-carboxamido)propyl)-2,4,6-triphenylpyridin-1-ium Tetrafluoroborate (S-49) ^{13}C NMR (151 MHz, DMSO-d_6)

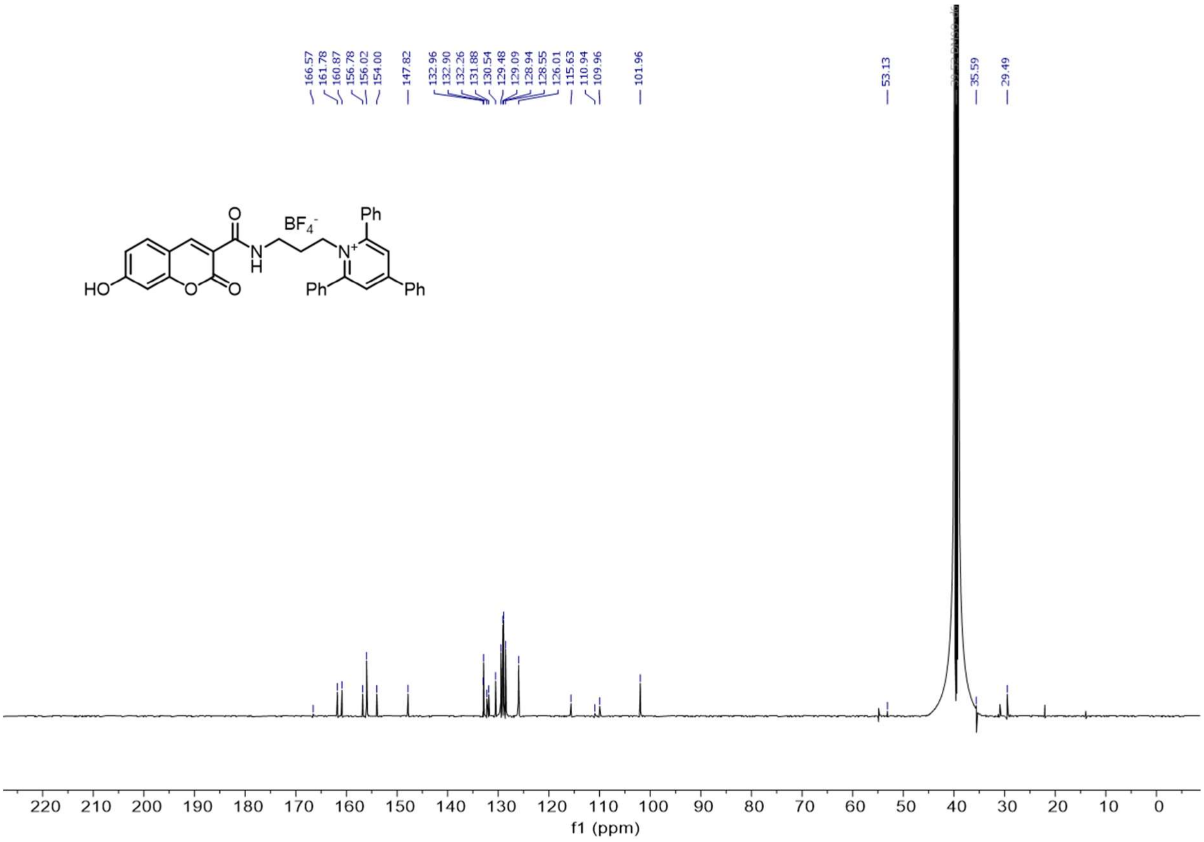

1-(3-(7-Hydroxy-2-oxo-*2H*-chromene-3-carboxamido)propyl)-2,4,6-triphenylpyridin-1-ium Tetrafluoroborate (S-49) ^{19}F NMR (471 MHz, DMSO-d_6)

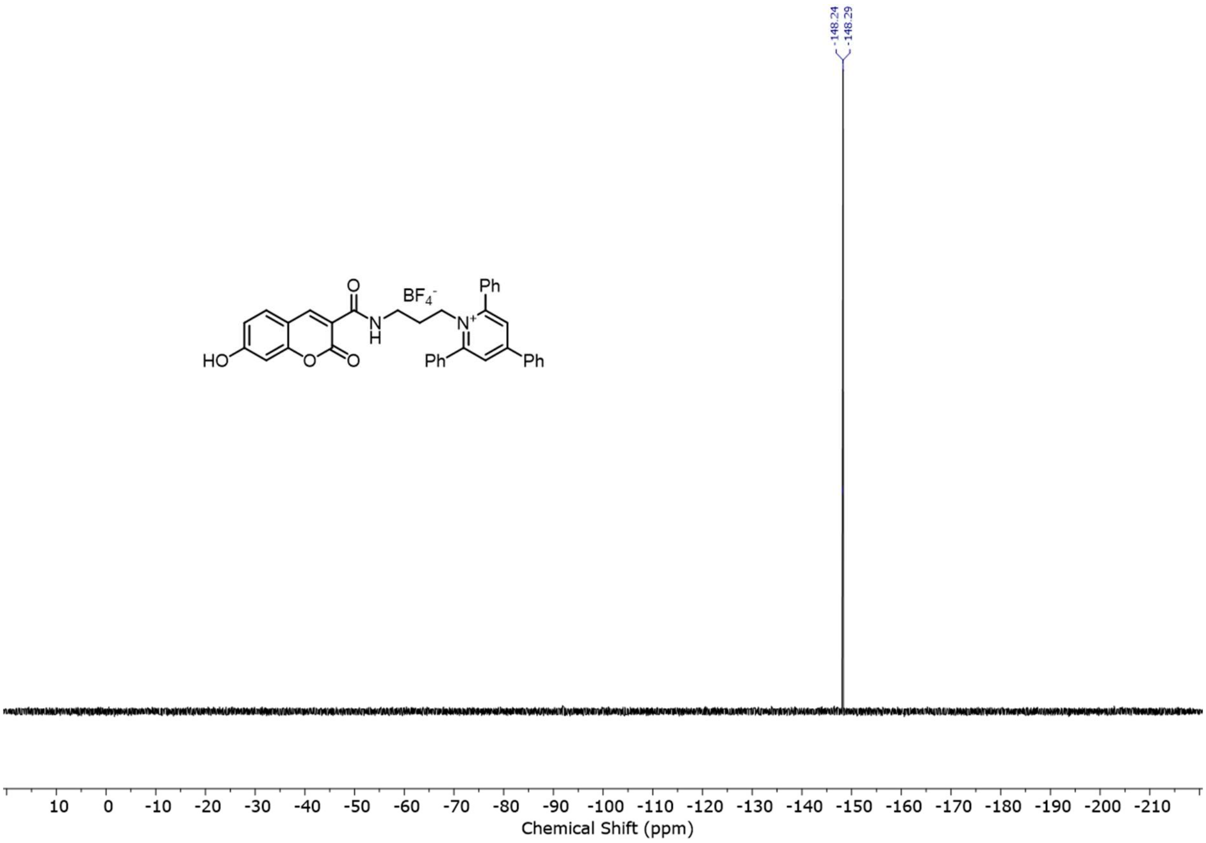

***tert*-Butyl (3-(4-(2-(benzhydryloxy)ethyl)piperazin-1-yl)propyl)carbamate dibenzene (S-51) ^{1}H NMR (400 MHz, $CDCl_3$)**

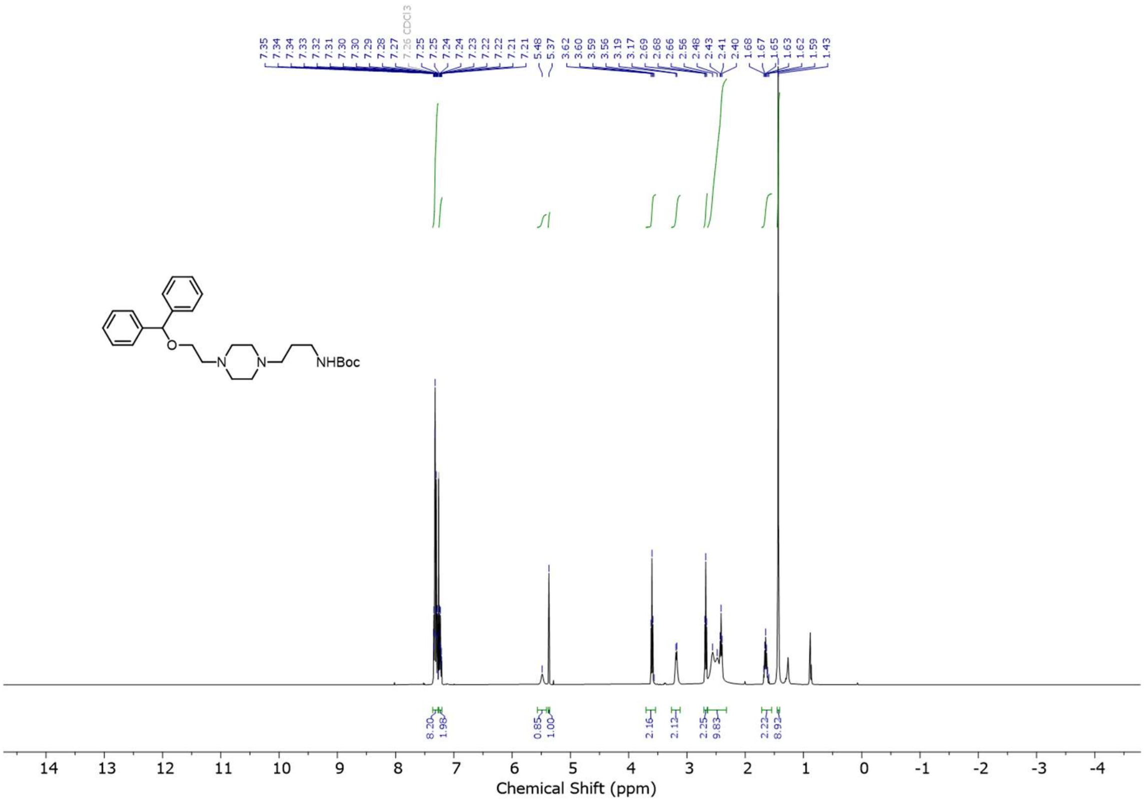

***tert*-Butyl (3-(4-(2-(benzhydryloxy)ethyl)piperazin-1-yl)propyl)carbamate dibenzene (S-51) ^{13}C NMR (101 MHz, $CDCl_3$)**

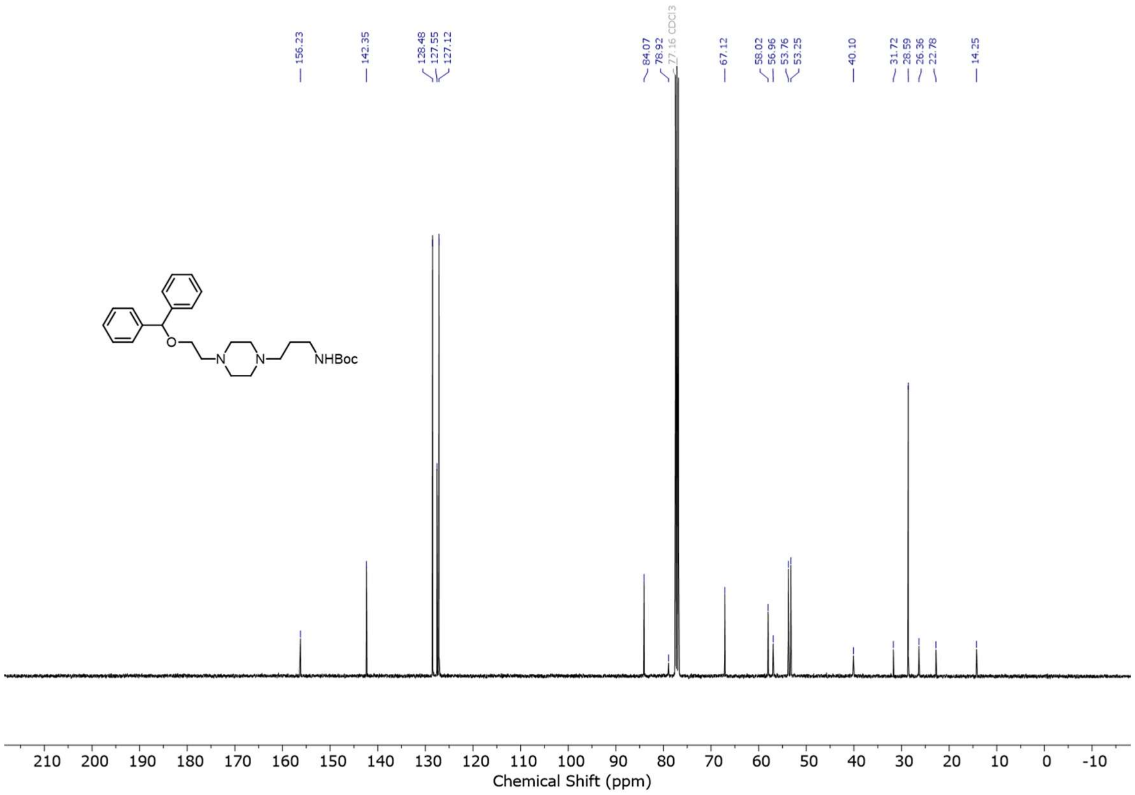

1-(3-(4-(2-(Benzhydryloxy)ethyl)piperazin-1-yl)propyl)-2,4,6-triphenylpyridin-1-ium Tetrafluoroborate (S-52) ^{1}H NMR (500 MHz, $CDCl_3$)

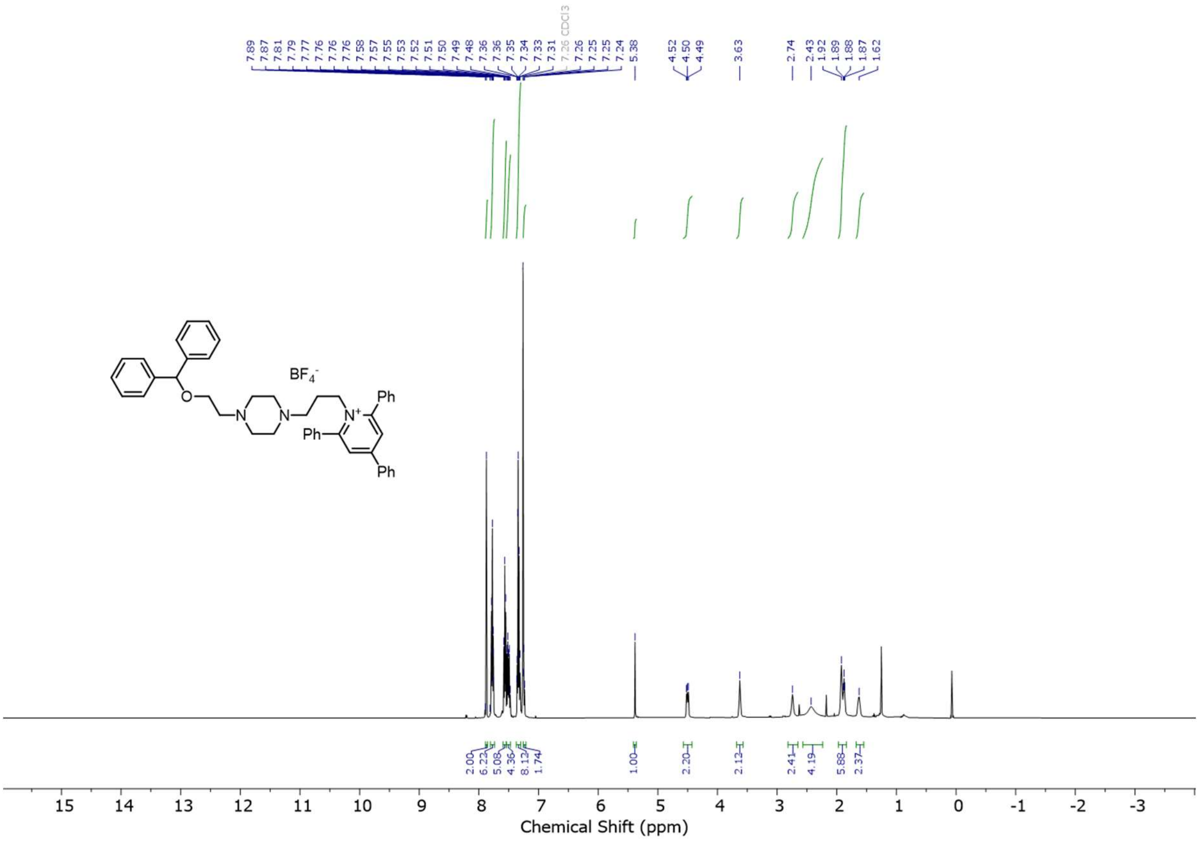

1-(3-(4-(2-(Benzhydryloxy)ethyl)piperazin-1-yl)propyl)-2,4,6-triphenylpyridin-1-ium Tetrafluoroborate (S-52) ^{13}C NMR (126 MHz, $CDCl_3$)

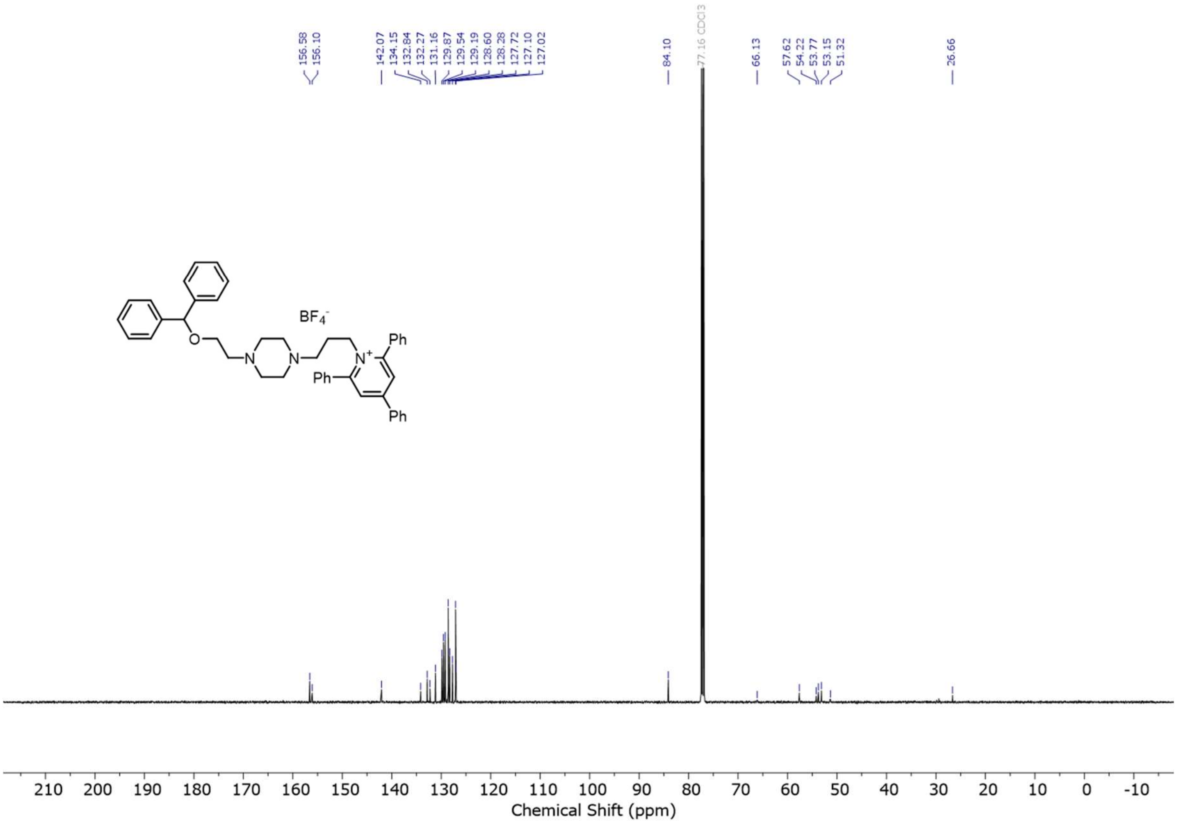

1-(3-(4-(2-(Benzhydryloxy)ethyl)piperazin-1-yl)propyl)-2,4,6-triphenylpyridin-1-ium Tetrafluoroborate (S-52) ^{19}F NMR (470 MHz, $CDCl_3$)

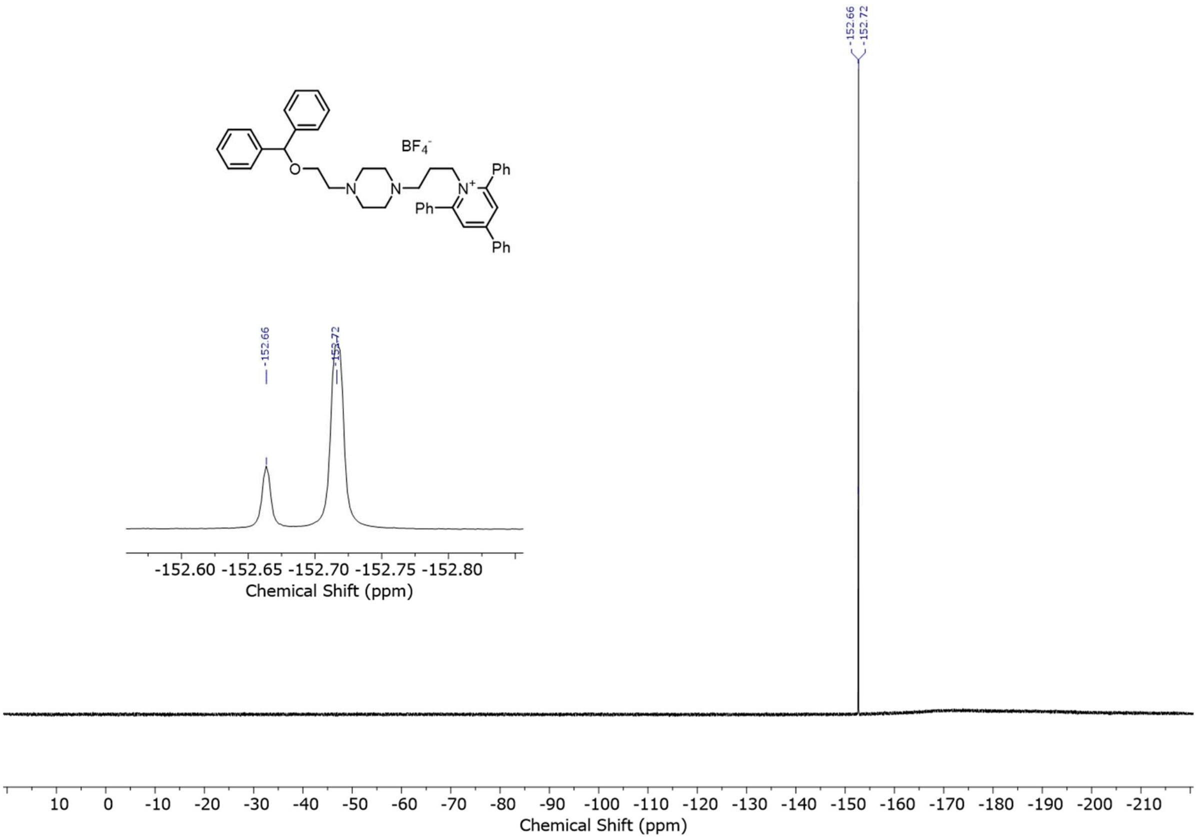

***tert*-Butyl (3-(4-(2-fluoroethoxy)phenyl)propyl)carbamate (S-56) ¹H NMR (400 MHz, CDCl₃)**

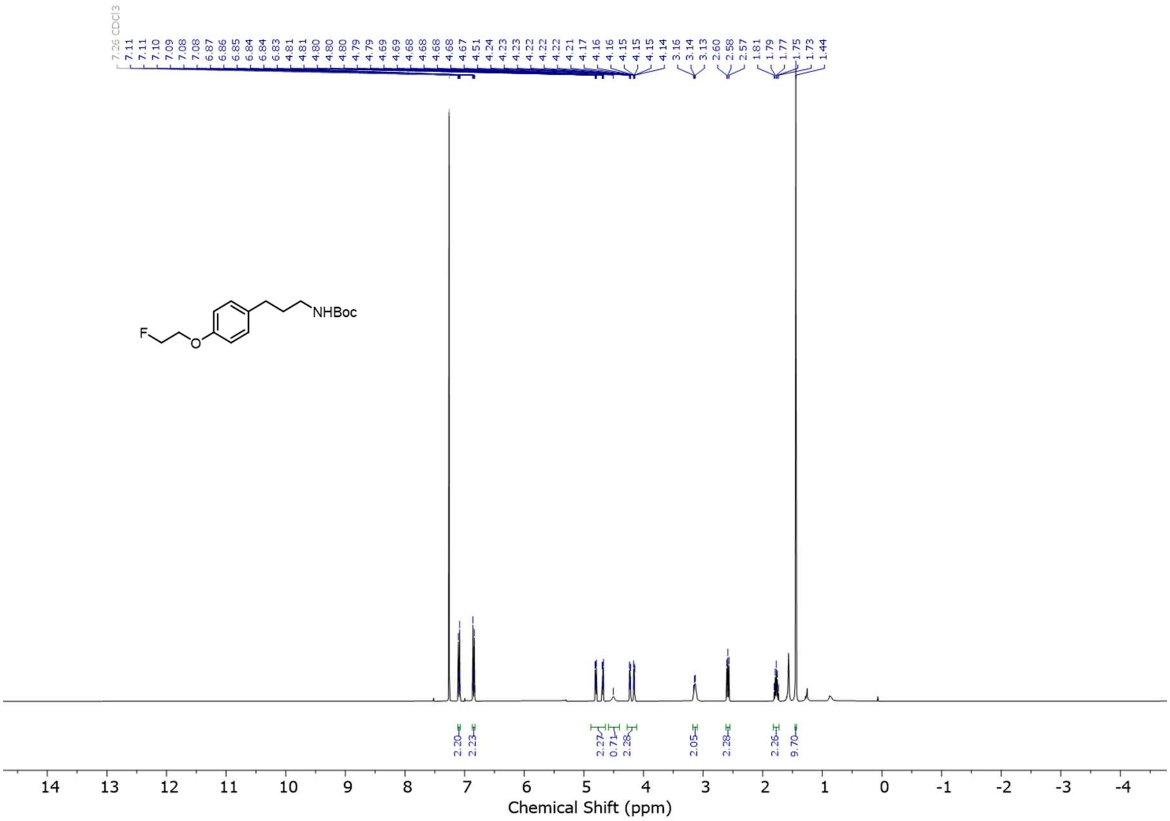

***tert*-Butyl (3-(4-(2-fluoroethoxy)phenyl)propyl)carbamate (S-56) ¹³C NMR (101 MHz, CDCl₃)**

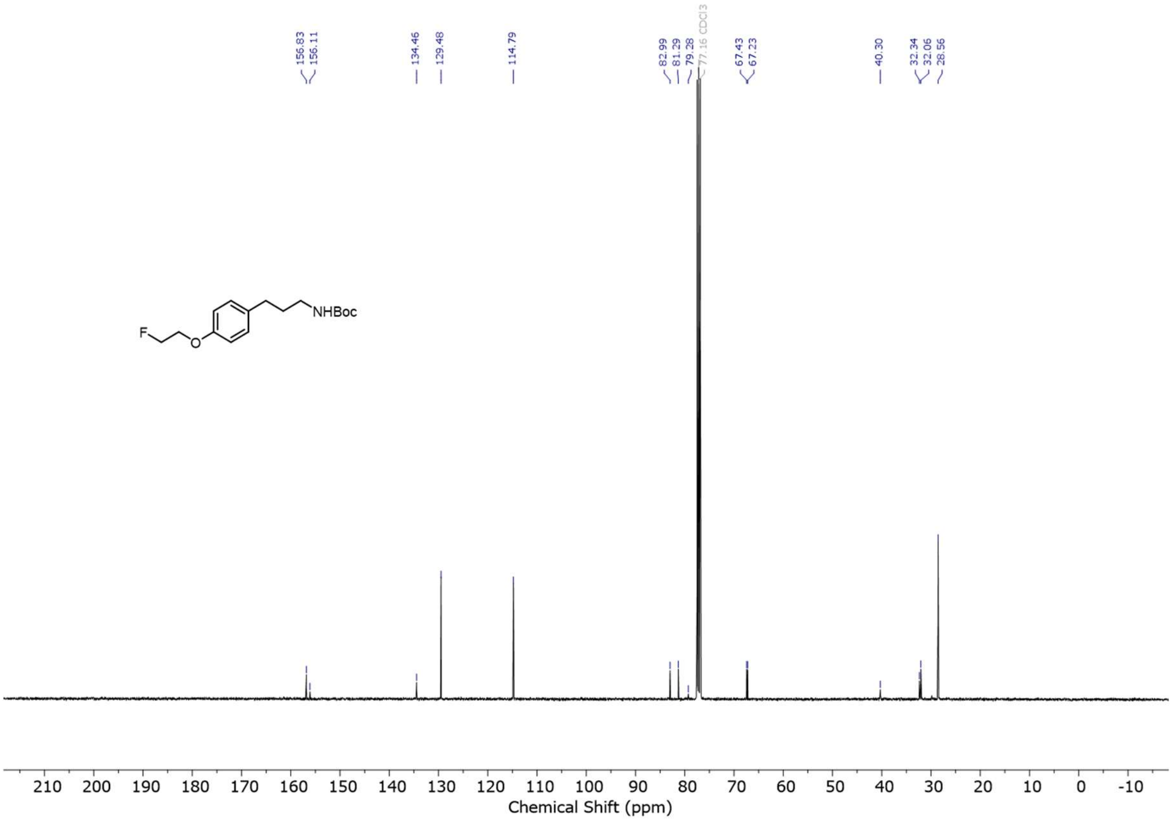

***tert*-Butyl (3-(4-(2-fluoroethoxy)phenyl)propyl)carbamate (S-56) ^{19}F NMR (377 MHz, $CDCl_3$)**

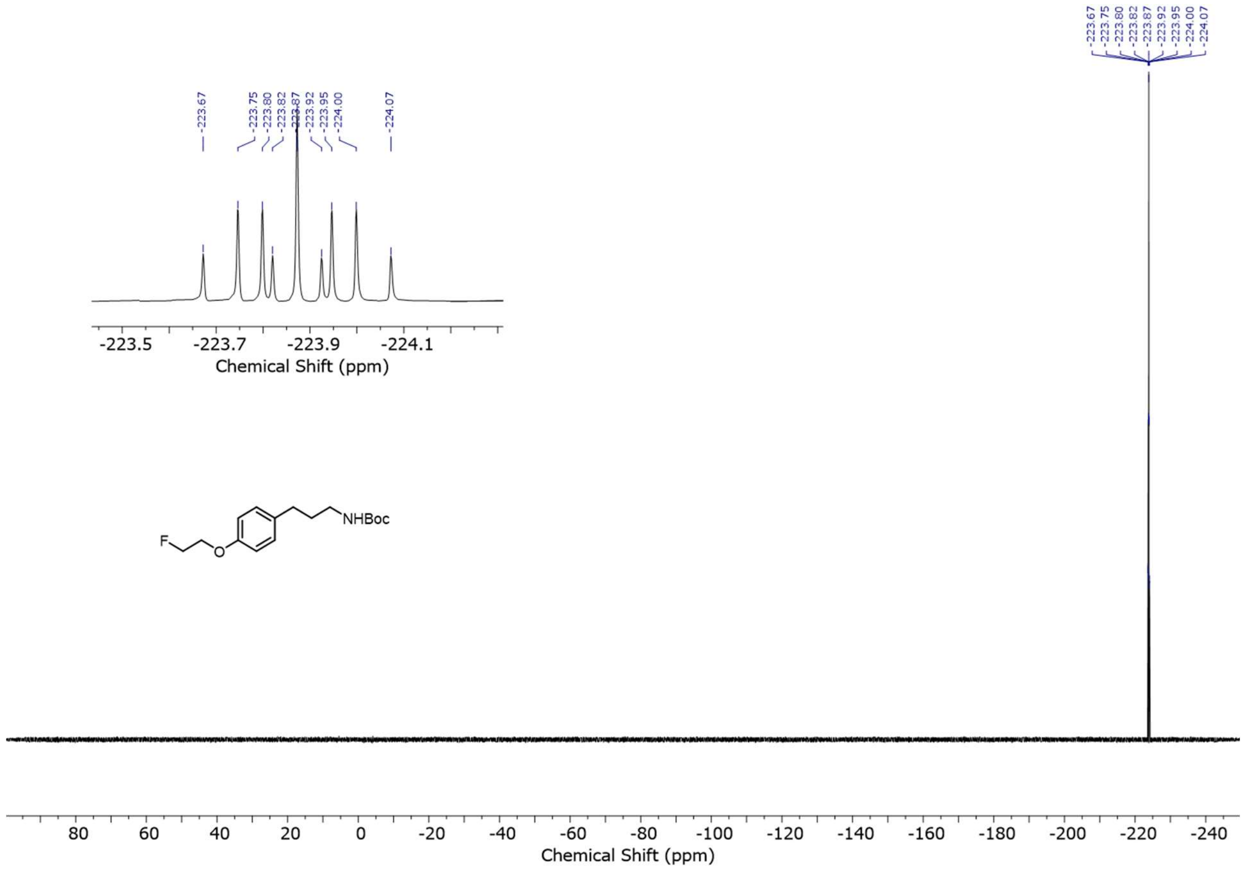

tert-Butyl (3-(6-fluoropyridin-3-yl)propyl)carbamate (S-57) ^{1}H NMR (500 MHz, CDCl$_3$)

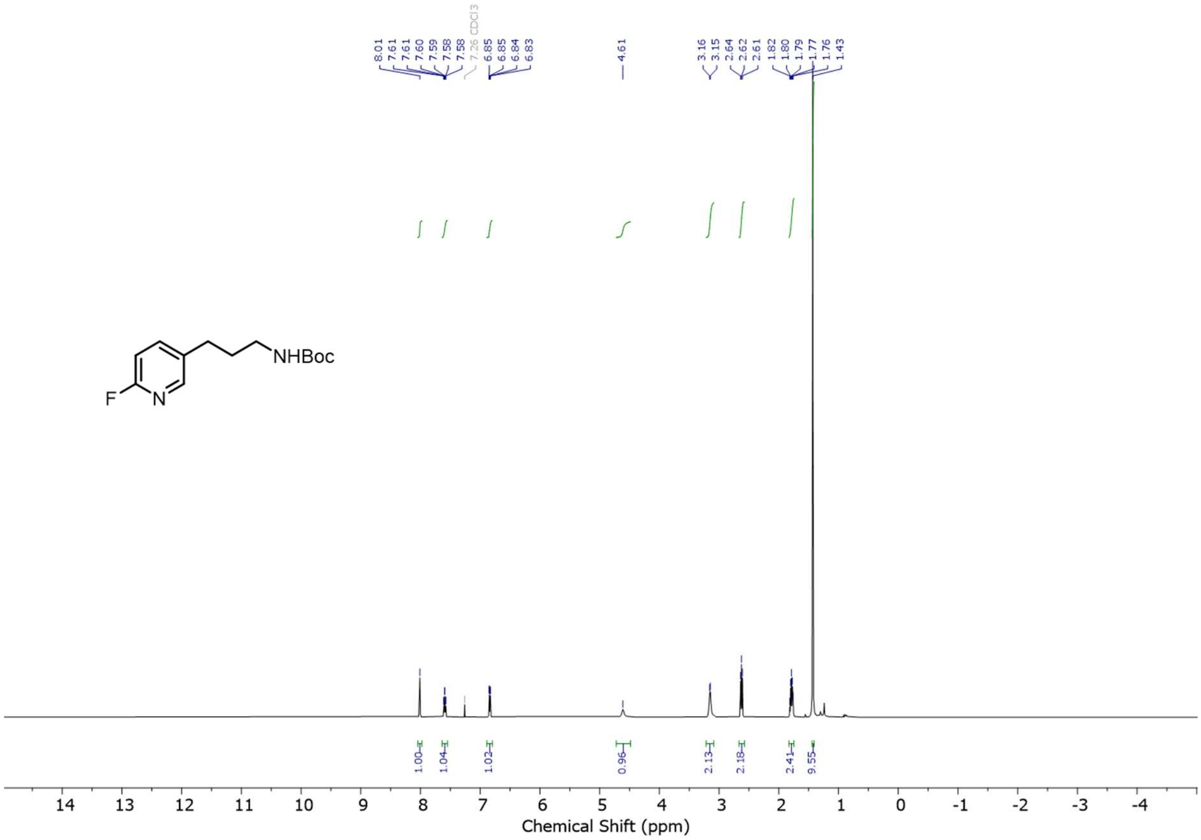

tert-Butyl (3-(6-fluoropyridin-3-yl)propyl)carbamate (S-57) ^{13}C NMR (126 MHz, CDCl$_3$)

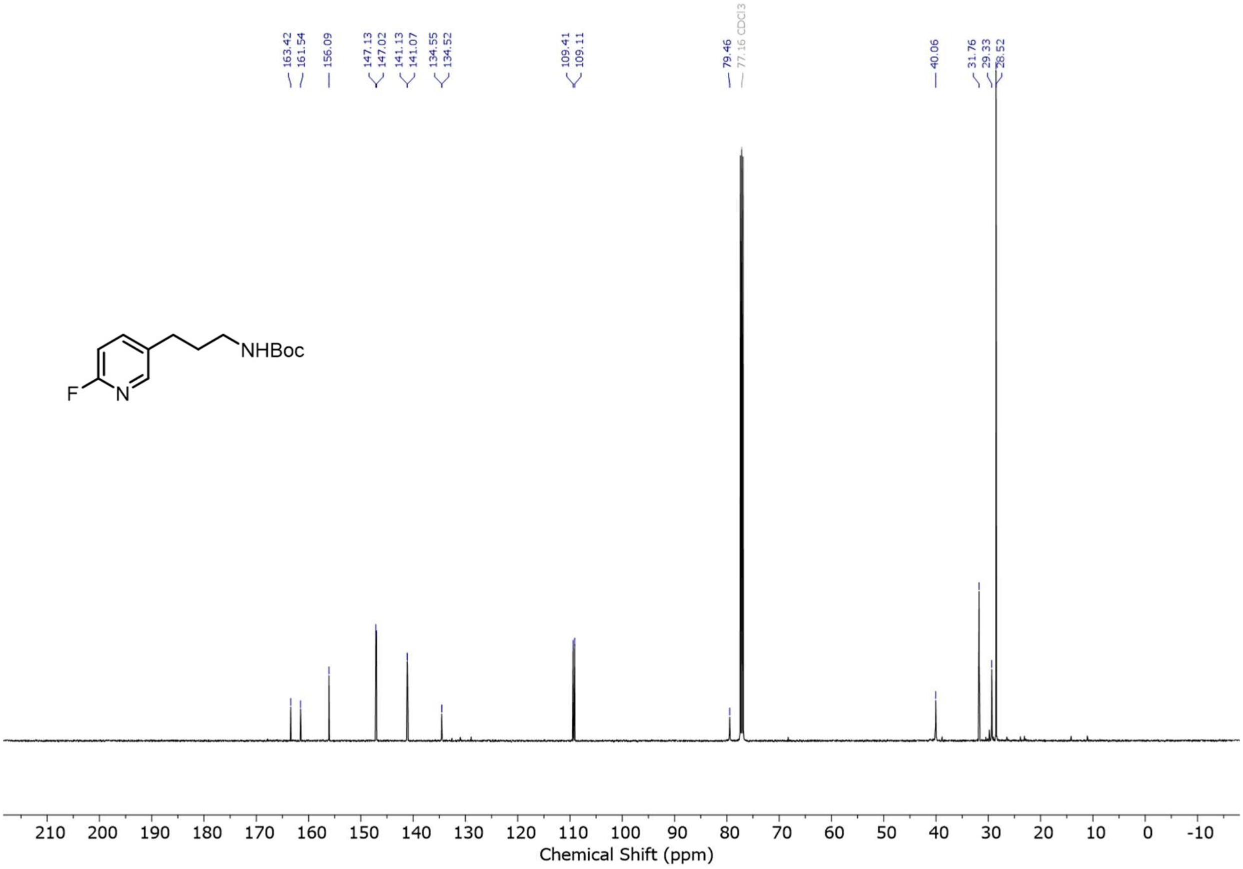

***tert*-Butyl (3-(6-fluoropyridin-3-yl)propyl)carbamate (S-57) ^{19}F NMR (471 MHz, $CDCl_3$)**

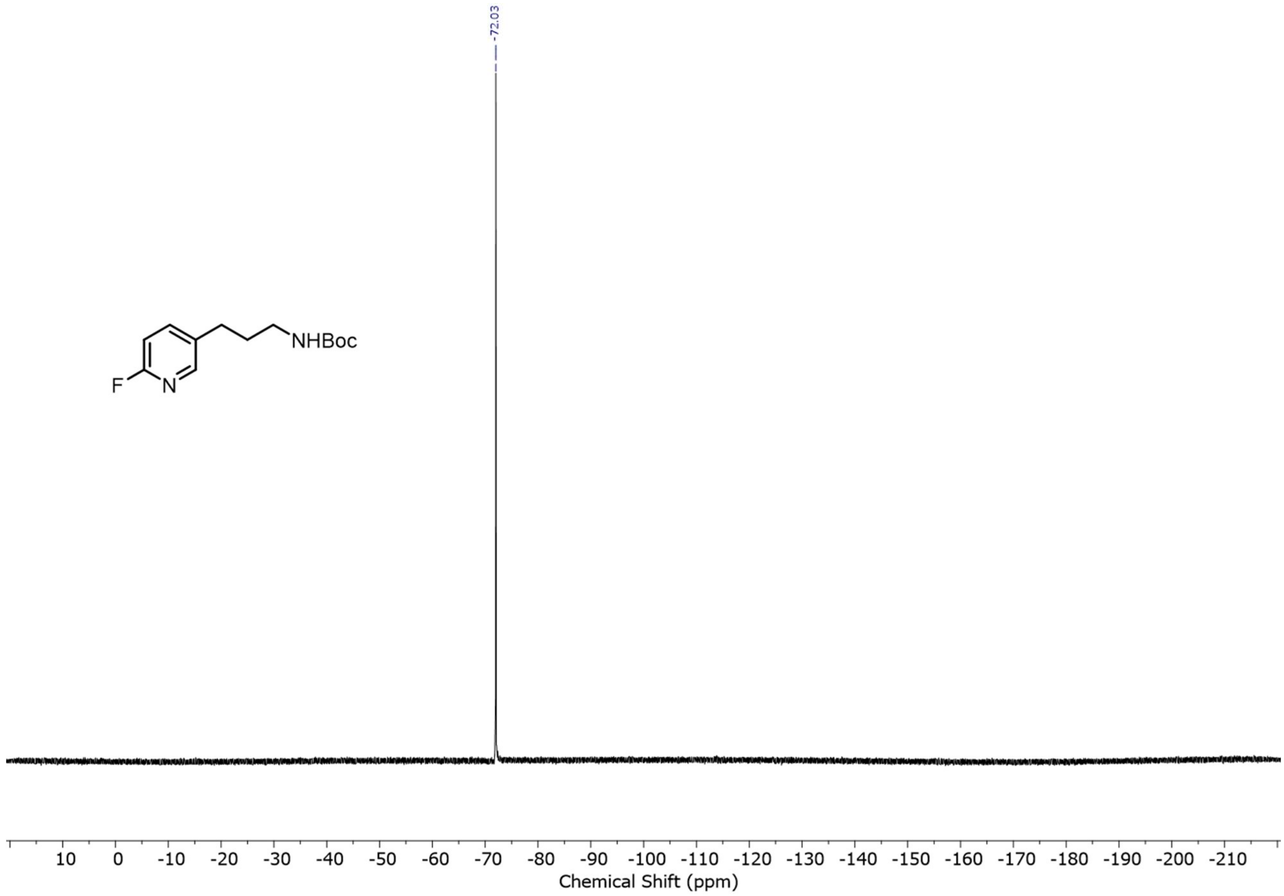

***tert*-Butyl 4-(3-(4-fluorophenyl)propyl)piperazine-1-carboxylate (S-58) ^{1}H NMR (500 MHz, $CDCl_3$)**

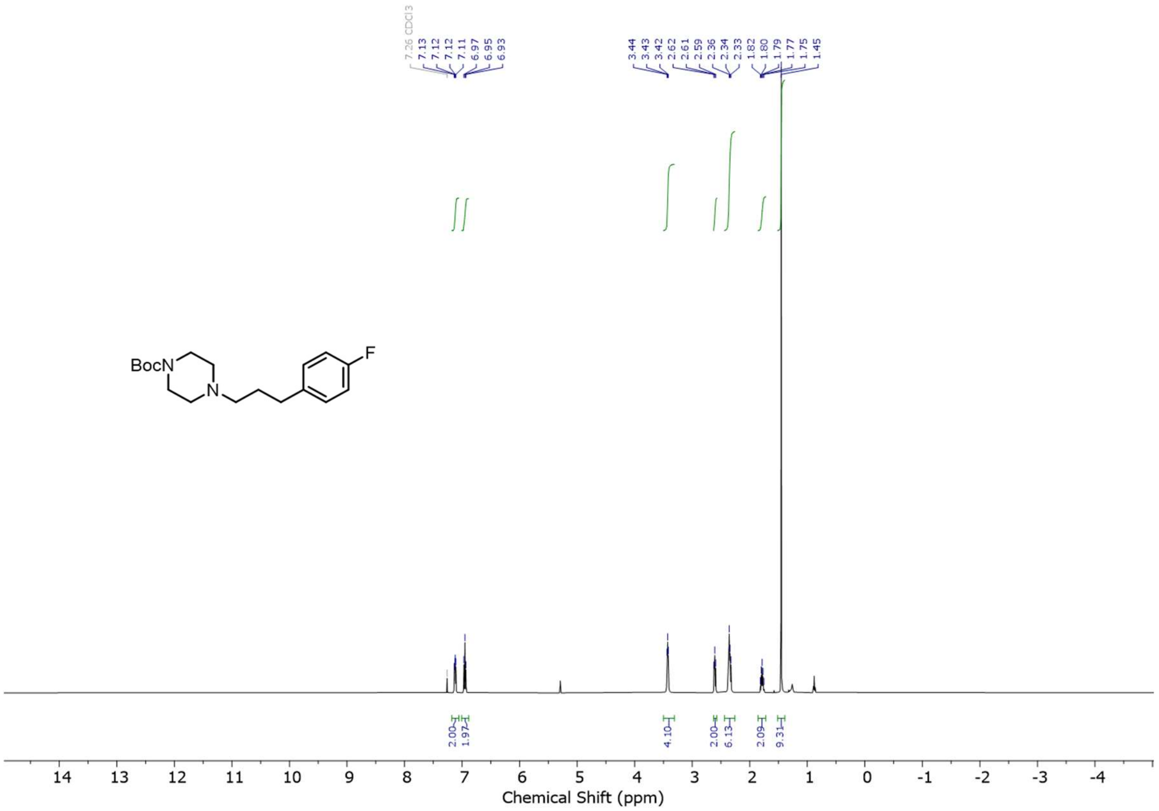

***tert*-Butyl 4-(3-(4-fluorophenyl)propyl)piperazine-1-carboxylate (S-58) ^{13}C NMR (126 MHz, $CDCl_3$)**

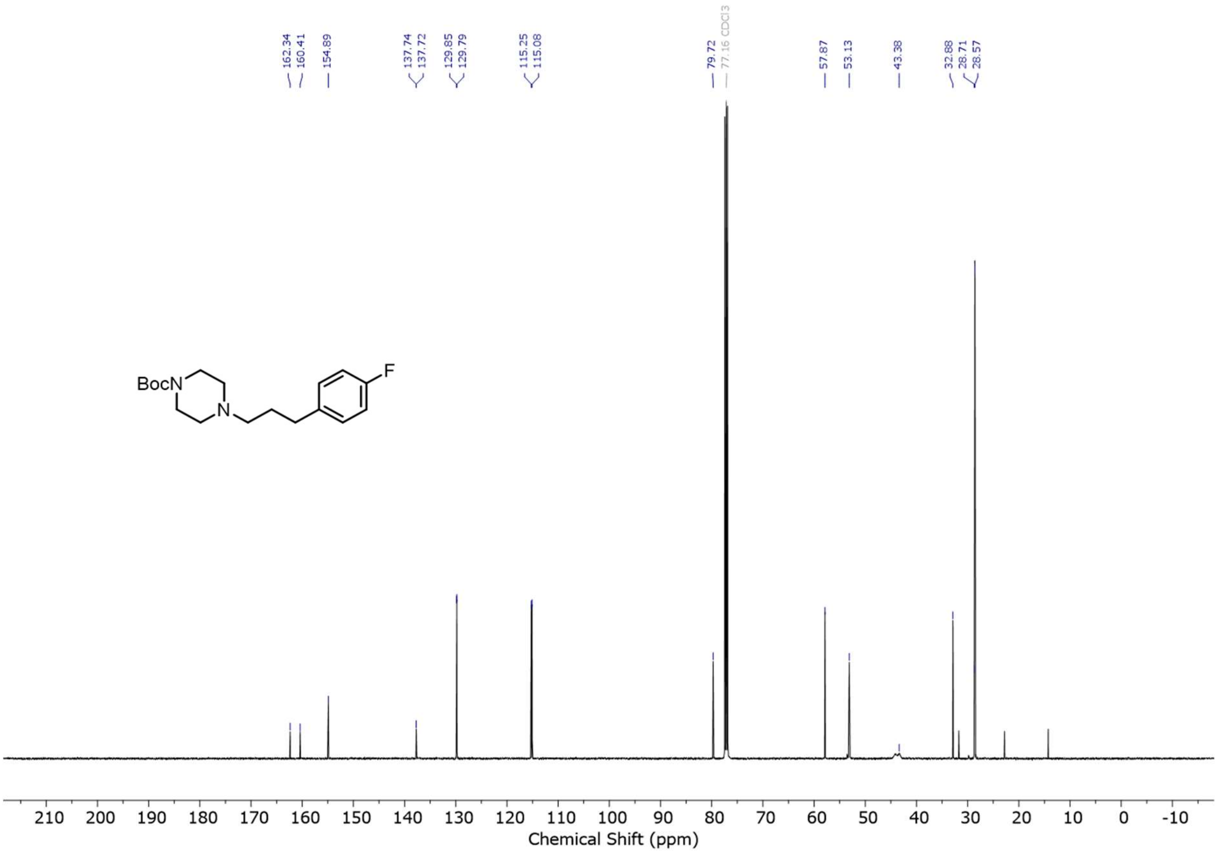

tert-Butyl 4-(3-(4-fluorophenyl)propyl)piperazine-1-carboxylate (S-58) ^{19}F NMR (471 MHz, CDCl$_3$)

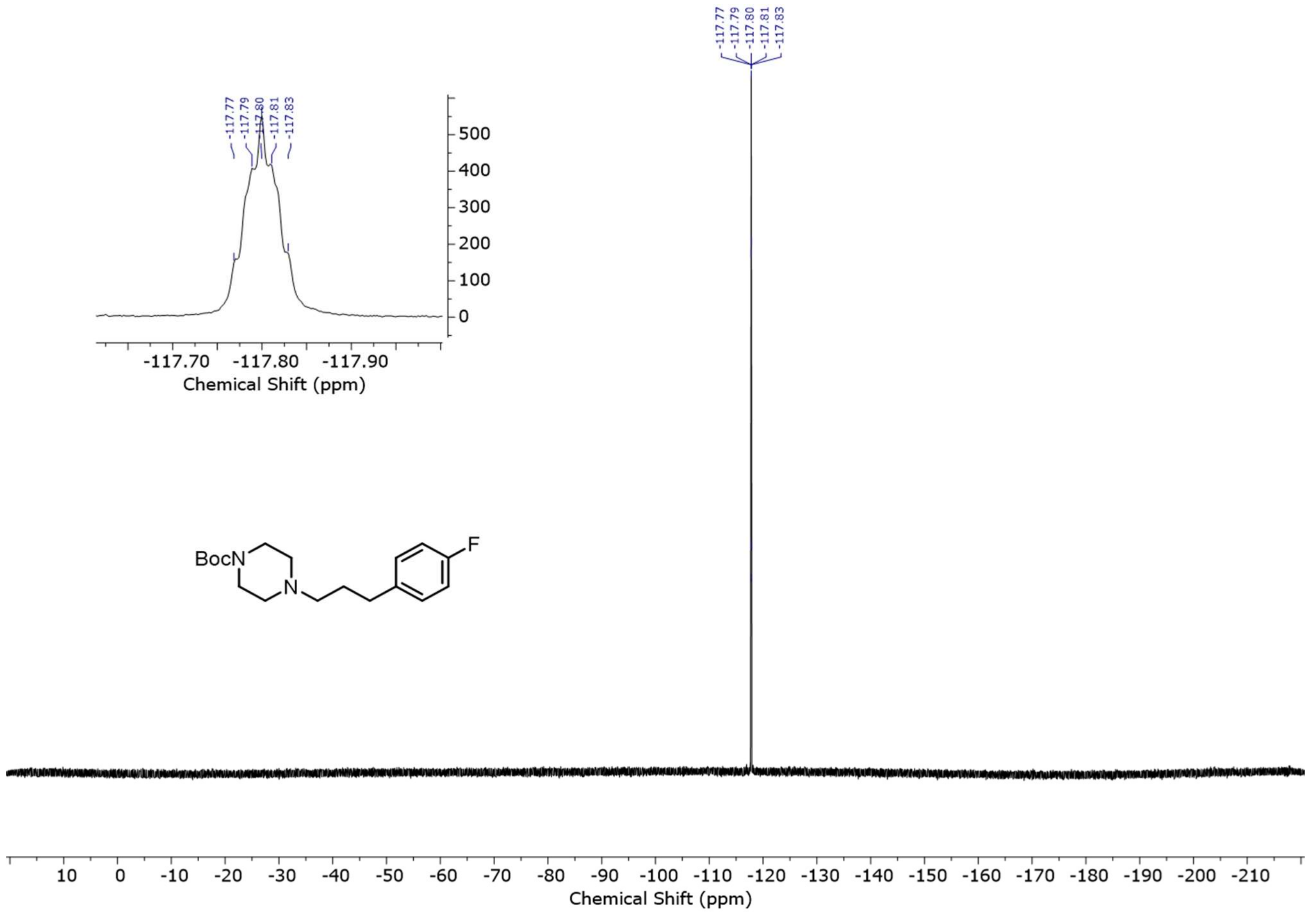

5-(3-chloro-4-methoxyphenyl)-*N*-(3-(3,4-difluorophenyl)propyl)oxazole-4-carboxamide (S-60) ^{1}H NMR (400 MHz, $CDCl_3$)

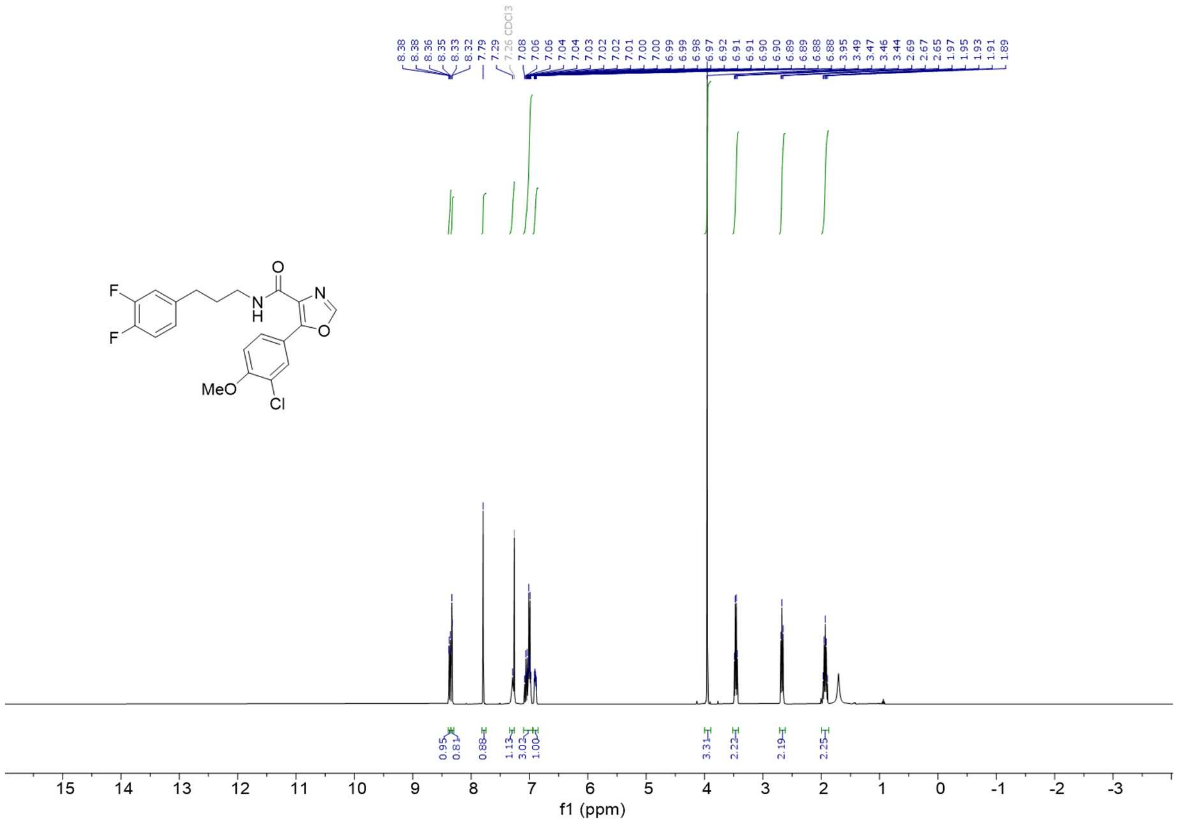

5-(3-chloro-4-methoxyphenyl)-*N*-(3-(3,4-difluorophenyl)propyl)oxazole-4-carboxamide (S-60) ^{19}F NMR (377 MHz, $CDCl_3$)

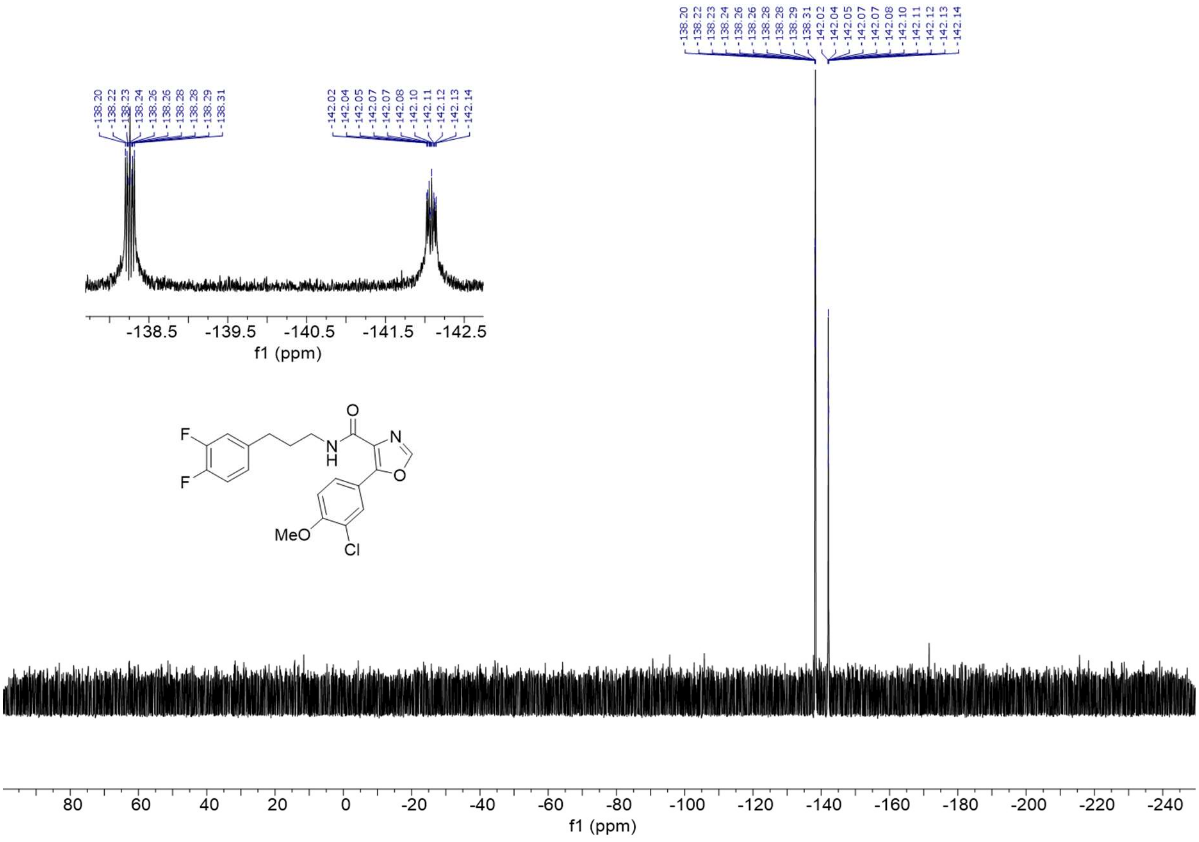

5-(3-chloro-4-methoxyphenyl)-*N*-(3-(3,5-difluorophenyl)propyl)oxazole-4-carboxamide (S-61) ^{1}H NMR (400 MHz, $CDCl_3$)

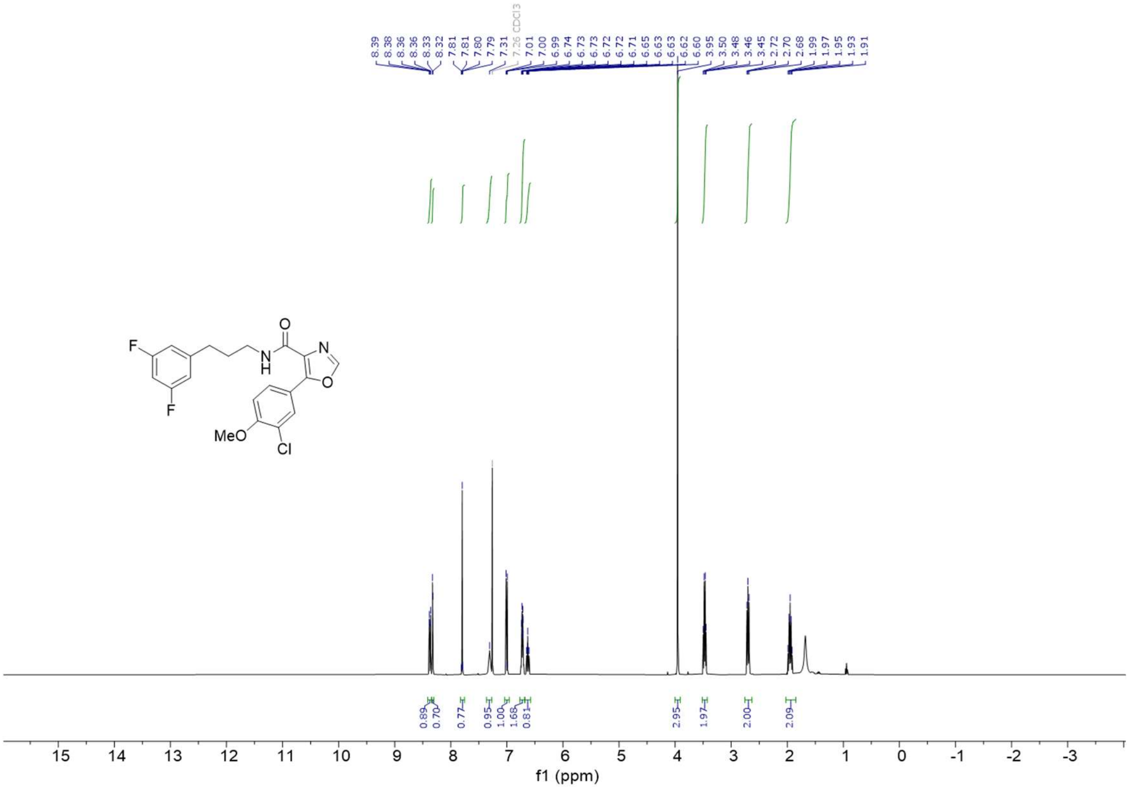

5-(3-chloro-4-methoxyphenyl)-*N*-(3-(3,5-difluorophenyl)propyl)oxazole-4-carboxamide (S-61) ^{19}F NMR (377 MHz, $CDCl_3$)

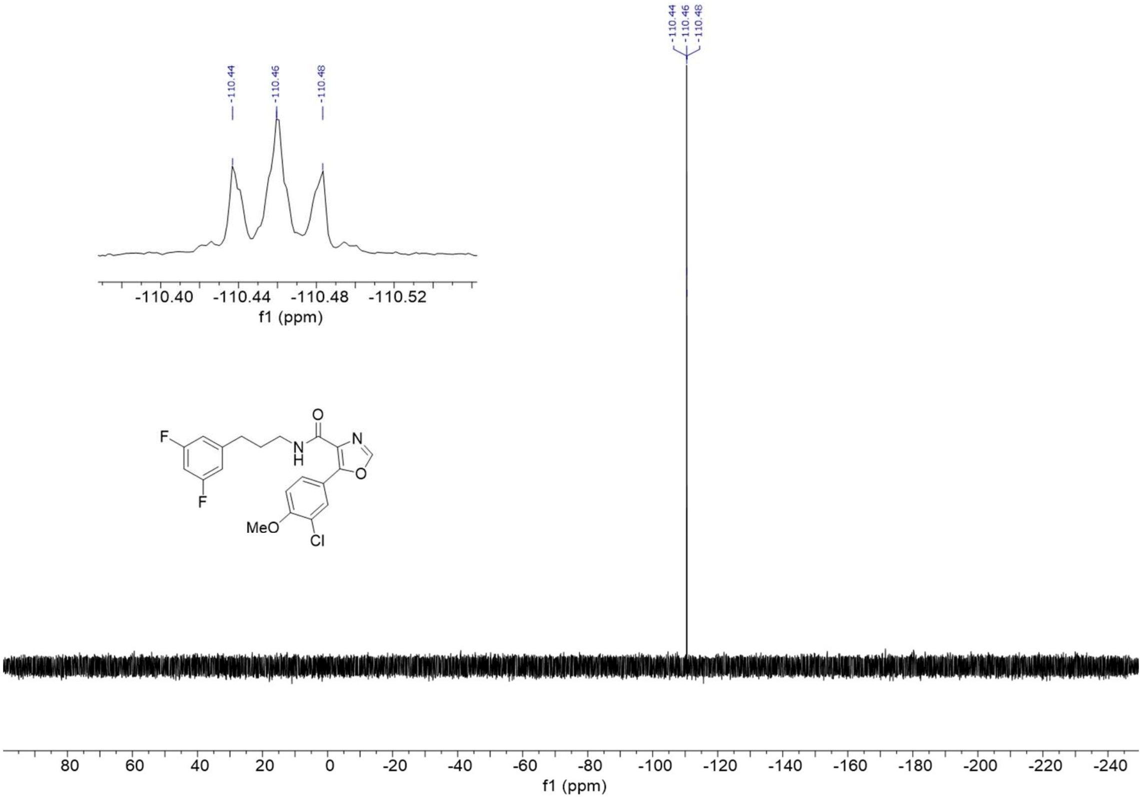

5-(3-chloro-4-methoxyphenyl)-*N*-(3-(3-fluoro-4-methoxyphenyl)propyl)oxazole-4-carboxamide (S-62) ^{1}H NMR (400 MHz, CDCl$_3$)

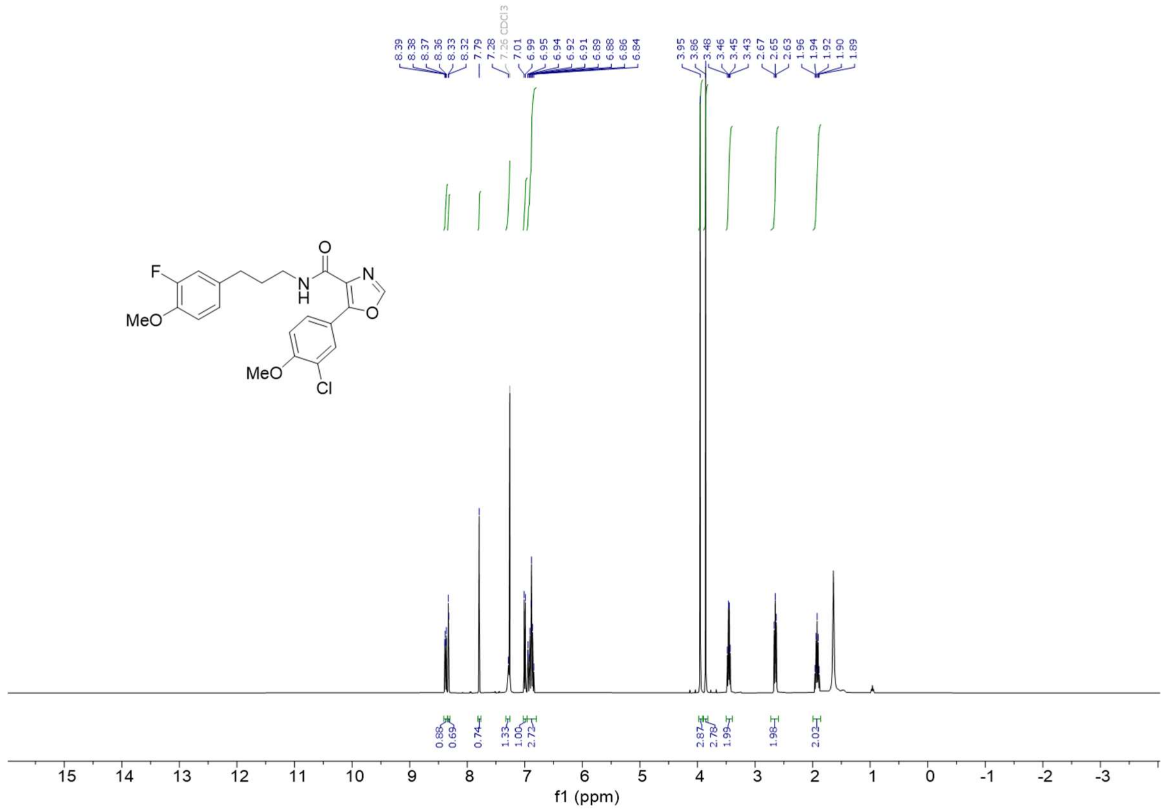

5-(3-chloro-4-methoxyphenyl)-*N*-(3-(3-fluoro-4-methoxyphenyl)propyl)oxazole-4-carboxamide (S-62) ^{19}F NMR (377 MHz, CDCl$_3$)

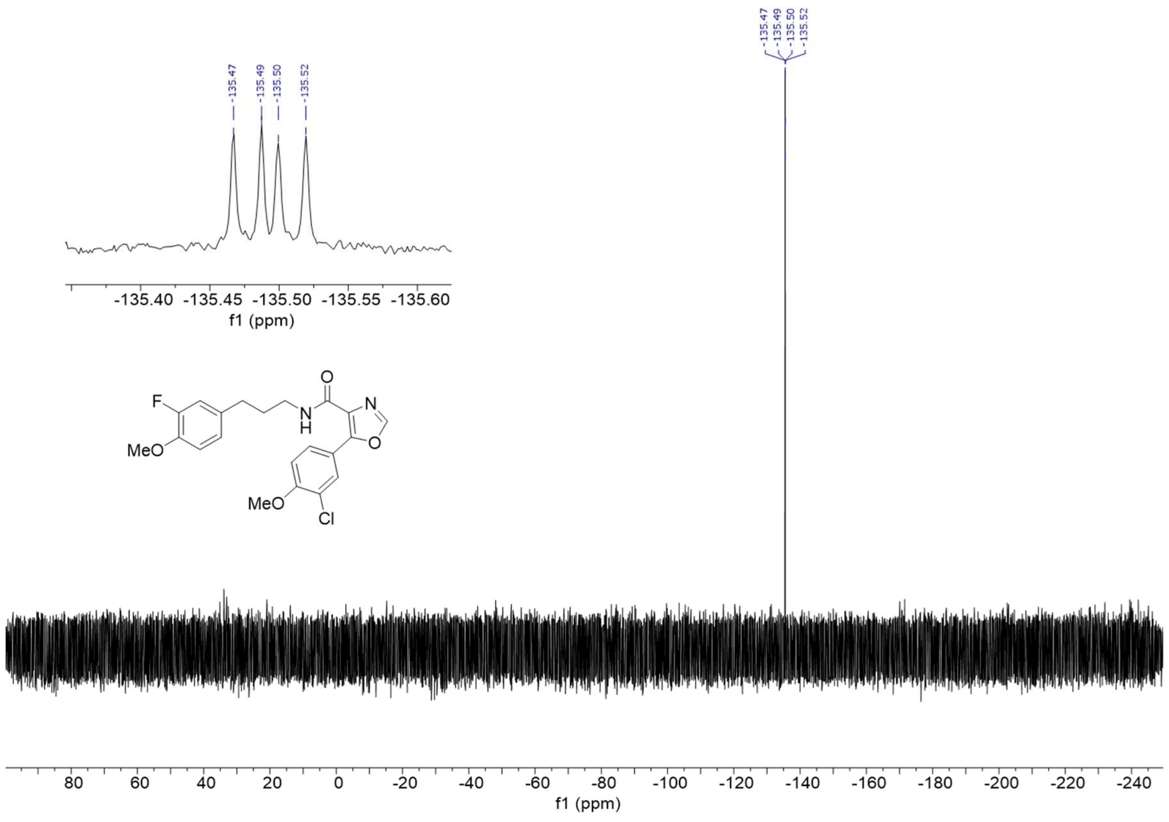

5-(3-chloro-4-methoxyphenyl)-*N*-(3-(4-fluoro-3-methoxyphenyl)propyl)oxazole-4-carboxamide (S-63) ^{1}H NMR (400 MHz, CDCl$_3$)

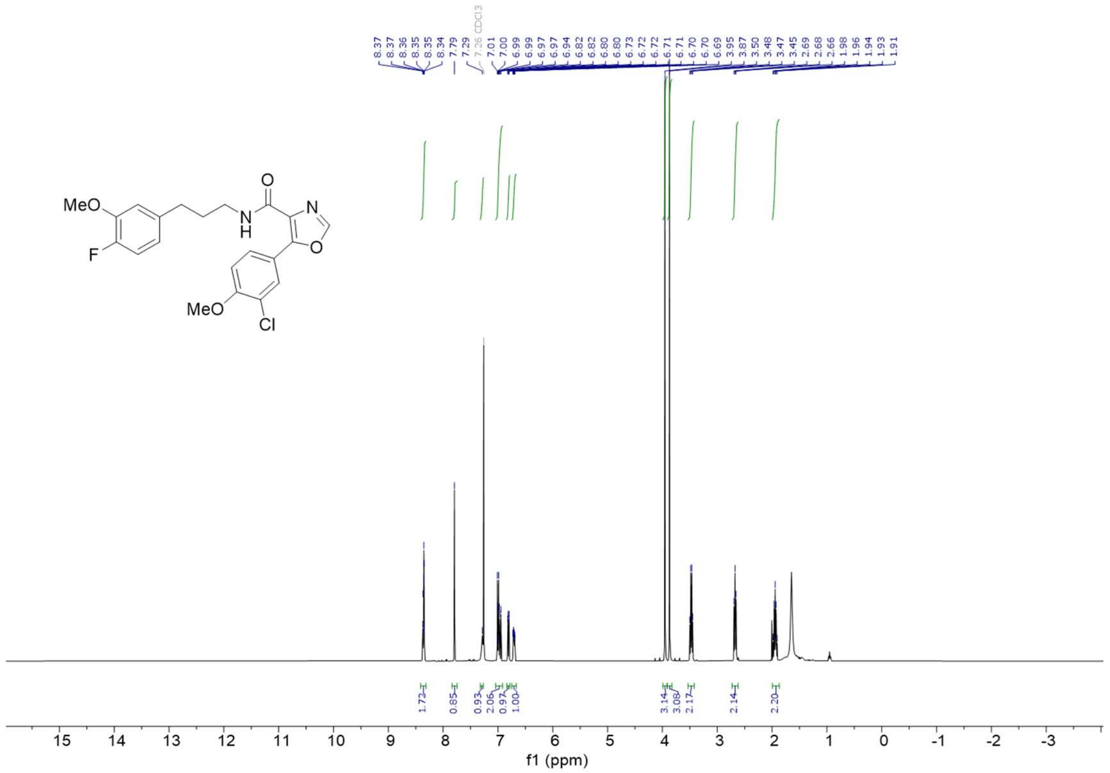

5-(3-chloro-4-methoxyphenyl)-*N*-(3-(4-fluoro-3-methoxyphenyl)propyl)oxazole-4-carboxamide (S-63) ^{19}F NMR (377 MHz, CDCl$_3$)

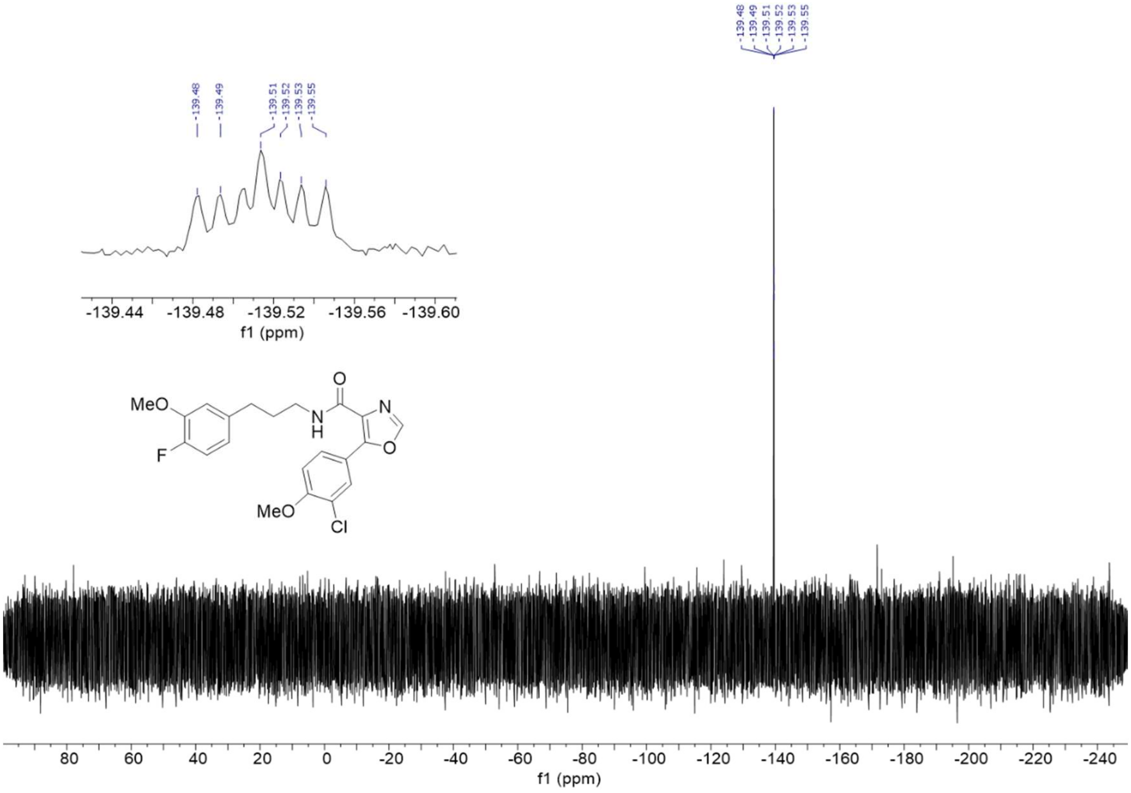

5-(3-chloro-4-methoxyphenyl)-*N*-(3-(3-cyano-2-fluoropyridin-4-yl)propyl)oxazole-4-carboxamide (S-64) ^{1}H NMR (500 MHz, $CDCl_3$)

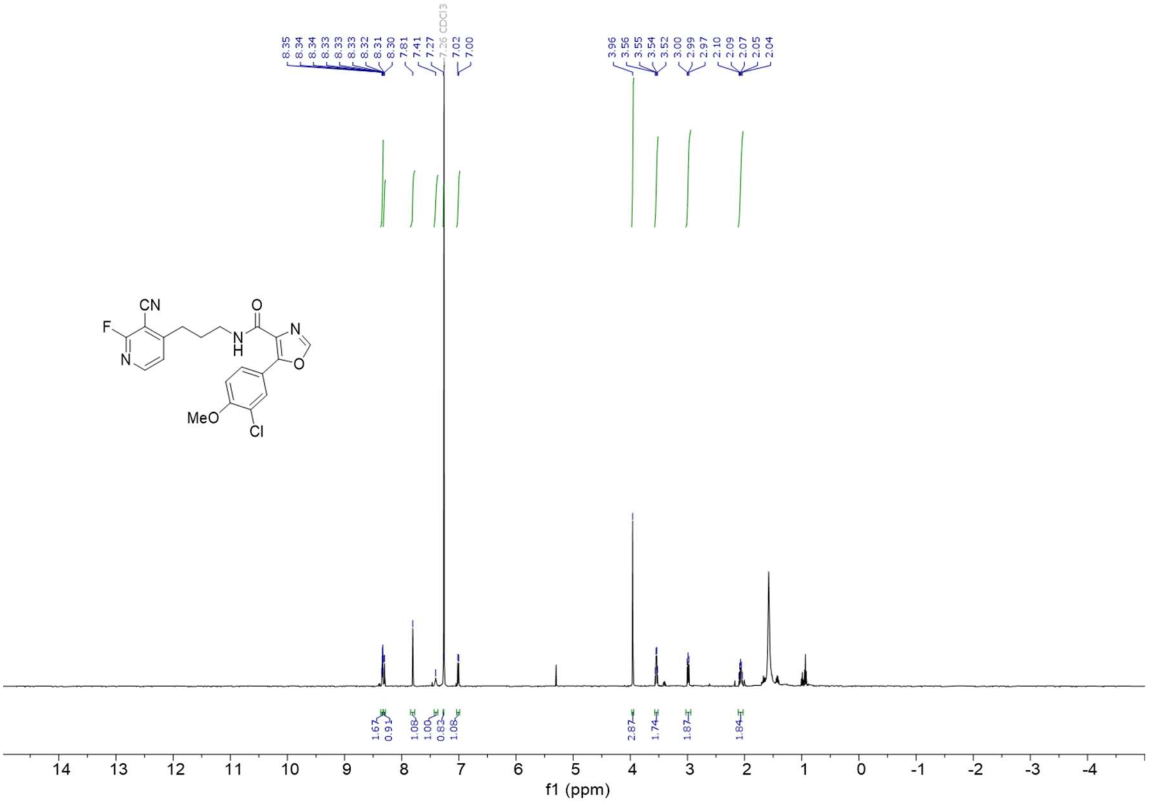

5-(3-chloro-4-methoxyphenyl)-*N*-(3-(3-cyano-2-fluoropyridin-4-yl)propyl)oxazole-4-carboxamide (S-64) ^{19}F NMR (471 MHz, $CDCl_3$)

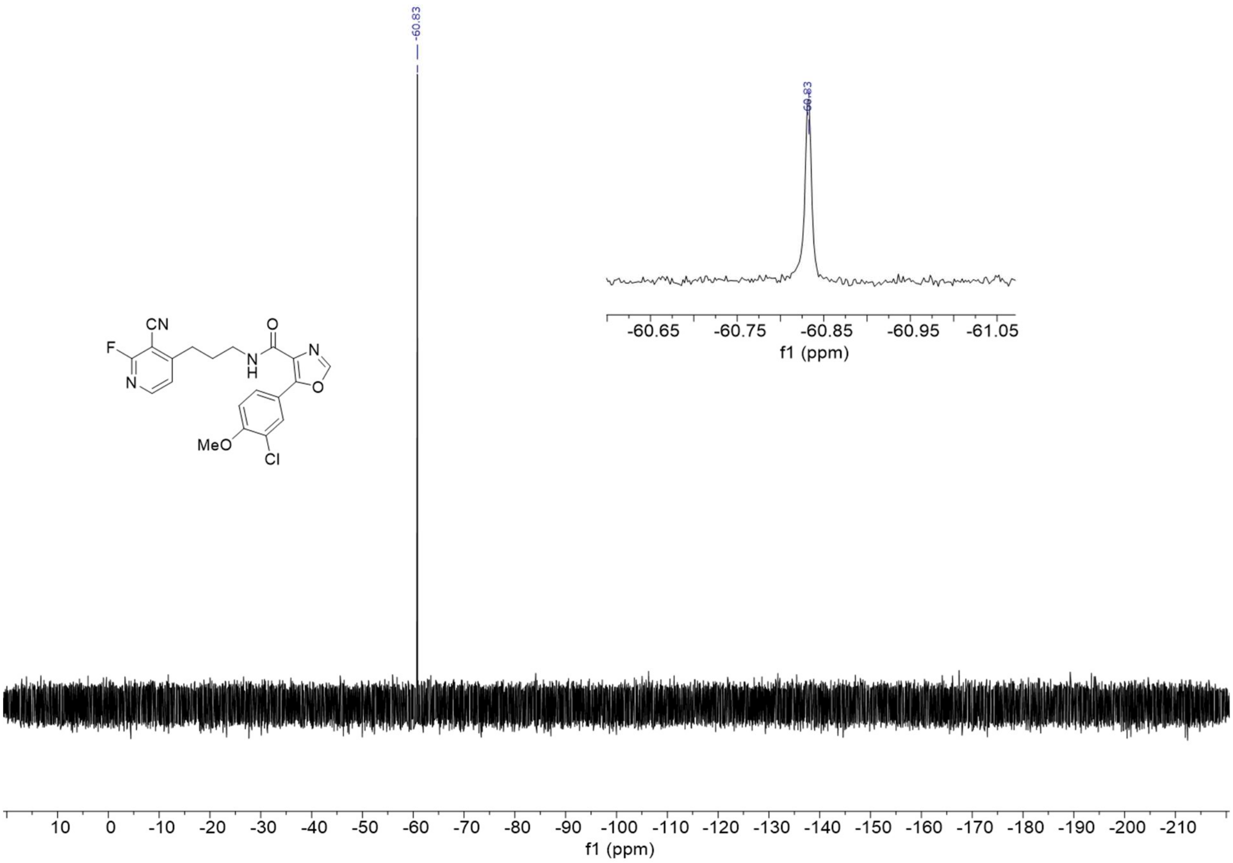

Supplement: Supplementary file 1 — Supporting Information [file ANIE-65-e22650-s001.pdf]
